# The Diagnostic and Prognostic Values of HOXA Gene Family in Kidney Clear Cell Renal Cell Carcinoma

**DOI:** 10.1155/2022/1762637

**Published:** 2022-03-16

**Authors:** Xiaofang Zong, Juexiu Fu, Ziyu Wang, Qianying Wang

**Affiliations:** ^1^Department of Otorhinolaryngology, The First Affiliated Hospital of Chongqing Medical University, Chongqing 400016, China; ^2^Center of Neuroscience, College of Basic Medicine, Chongqing Medical University, Chongqing 400016, China; ^3^School of Medicine, Qingdao University, Qingdao 266011, China

## Abstract

Kidney renal clear cell carcinoma (KIRC) is one of the most common cancers with high mortality worldwide. As members of the homeobox (HOX) family, homeobox-A (HOXA) genes have been reported to play an increasingly important role in tumorigenesis and the progression of multiple cancers. However, limited studies have investigated the potential diagnostic and prognostic roles of HOXA genes in KIRC. In this research, we explored the expression pattern of the HOXA gene family in KIRC progression by differential analysis of expression profiles from The Cancer Genome Atlas (TCGA). By using univariate Cox analysis and lasso regression analysis, we comprehensively evaluated the prognostic value of HOXA genes and eventually identified a prognostic risk model consisting of five HOXA genes (HOXA2, HOXA3, HOXA7, HOXA11, and HOXA13). The risk model was further validated as a novel independent prognostic factor for KIRC patients based on the calculated risk score by Kaplan–Meier analysis, univariate and multivariate Cox regression analyses, and time-dependent receiver operating characteristic (ROC) curve analysis. Moreover, to explore the potential mechanism of tumorigenesis and clinical application of KIRC, we also developed the HOXA-based competing endogenous RNA (ceRNA) regulatory network and machine learning classification model. Valproic acid and tretinoin were predicted to be the most promising small molecules to adjuvant treatment of KIRC by mining the CMAP and DGIdb drug database. Subsequently, pathway and functional enrichment analyses provided us with new ways to search for a possible mechanism of action of drugs. Taken together, our study demonstrated the nonnegligible role of HOXA genes in KIRC and constructed an effective prognostic and diagnostic model, which offers novel insights into KIRC prognosis.

## 1. Introduction

Homeobox (HOX) gene family, as pivotal regulatory factors in mammalian development, strongly correlates with body repair and various homeostatic cellular processes [1, 2]. Currently, there are arrayed 4 gene clusters with 9 to 11 genes per cluster named HOXA, HOXB, HOXC, and HOXD, respectively [3]. The HOXA cluster is composed of 11 genes (HOXA1, HOXA2, HOXA3, HOXA4, HOXA5, HOXA6, HOXA7, HOXA9, HOXA10, HOXA11, and HOXA13), which encodes highly conserved DNA-binding transcription factors [4]. Homologous fragments at the end of the HOXA genes can specifically recognize and bind to the TAAT or TTAT box in the promoter region of the target gene and further play a regulatory role by activation and inhibition of transcription of downstream genes. The biological functions of the HOXA gene family cover a wide spectrum of life regulatory processes, including cell differentiation, cell proliferation, and cell death [5]. Noteworthily, the HOXA gene family also controls the early patterns of embryo segmentation, as well as postdevelopmental events. To date, some literature have found that the expression of some HOXA genes was dysregulated in certain types of cancers, which might lead to carcinogenesis [6–9]. For example, the upregulation expression of the HOXA1 gene in breast cells would conduce to increased cell proliferation and drug resistance in clinical [10, 11]. HOXA gene expression showed tissue specificity in colorectal carcinoma, which presented a more expression level of HOXA13 in normal colons than in malignant colons [12]. Costa et al. reported the abnormal expression of HOXA9 and HOXA10 and estimated their prognostic value in glioblastoma multiforme [13, 14]. Therefore, a comprehensive exploration of the biological functions of the HOXA gene family in a wider range of tumors will bring us light in the field of prognosis and early screening of tumors.

Renal cell carcinoma (RCC) with approximately 175,000 deaths occurred in 2020 is one of the most frequent malignancies of the urinary system [15, 16]. Kidney clear cell renal cell carcinoma (KIRC) is the commonest subtype of RCC, representing roughly 75% [17]. Since KIRC lacked sensitivity to chemotherapy and radiotherapy, the primary treatment for KIRC is still surgery. However, 30% of patients experience metastasis or recurrence after radical nephrectomy [18]. With the improvement of medical science, the five-year overall survival rate (OS) of early-stage KIRC could reach about 80%–95%, whereas the five-year survival rate of patients with advanced stages drops less than 10% sharply [19]. Therefore, there is still an urgent need to study new and sensitive KIRC tumor prognostic markers to reduce the number of KIRC patients who are not diagnosed before the onset of the invasive disease [20].

Considering the accuracy of the prognosis and diagnostic model, the discovery of the potential value of the HOXA gene family in KIRC will be more valuable than simple genes. We comprehensively analyzed HOXA gene family expression profiles and identified five significantly prognostic-related genes based on the TCGA-KIRC cohort. A subsequent corresponding prognostic model was constructed and further verified its reproducibility in the internal and external test set. Besides, we developed the ceRNA regulatory network based on the HOXA gene family and an efficient machine-learning classification model in order to achieve the early screening of KIRC patients. According to differential expression profiles from the TCGA-KIRC cohort, we also carefully screened out possible underlying small molecular drugs by mining public drug databases. The complete workflow of the study is displayed in Supplementary Figure 1.

## 2. Materials and Methods

### 2.1. Data Collection and Preprocessing

Gene expression quantification data (FPKM format) for KIRC were downloaded from TCGA (https://portal.gdc.cancer.gov/). Then 72 normal samples and 539 KIRC samples were obtained. The RNA expression matrix was extracted separately by annotations using the Gencode (GENCODE v 26) GTF file and normalized. For repetitive gene expression data, the average value was utilized as the expression level. The corresponding clinical information (*n* = 537) of KIRC also were obtained from the TCGA database. Patients with missing clinical information (including TNM, grade, clinic stage, etc.) or with OS less than 30 days were excluded from this study to reduce statistical bias. Ultimately, we obtained a TCGA-KIRC cohort consisting of gene expression profiles and corresponding matching clinical information for 487 patients. Sample information of collected TCGA-KIRC is shown in Table 1. Additionally, to increase the robustness, the external validation cohort set (E-MTAB-3033) used to validate the prognostic model was downloaded from the ArrayExpress (https://www.ebi.ac.uk/arrayexpress/) database, acquired using the Illumina Hiseq platform. The data set included 91 KIRC samples and corresponding clinical data. The external validation set (GSE151419) used to validate the machine learning model was obtained from the GEO (https://www.ncbi.nlm.nih.gov/geo) database. The data set consisted of 58 KIRC tumor samples and 17 normal adjacent kidney tissue samples, which provided full RNA expression profiles based on the Illumina Hiseq platform. Overall, we collected a training set from the TCGA-KIRC cohort and two external validation sets from the remaining two databases. The “caret” package in the R environment was utilized to achieve data partitioning in order to develop subsequent different models in our analysis.

### 2.2. Comparison of the mRNA Expression of HOXA Gene Family in KIRC and Normal Tissues and Correlation Analysis

In order to obtain HOXA gene family members with prognostic significance rather than simply differential gene identification, we firstly utilized Wilcoxon test between 539 KIRC samples and 72 normal controlled samples to preliminarily filter potential differential expression genes (DEGs). *P* values obtained from the Wilcoxon test and fold change calculation were adjusted using the BH16 method [21]. Thresholds of |log2FC| > 1.0 and an adjusted *P* value of <0.05 were selected (Supplementary Table S1). The mRNA expression levels of the HOXA gene family were obtained from the whole genome mRNA expression by utilizing Perl software (version 4.26; https://www.perl.org/). Thereafter, we utilized the “corrplot” package in R to explore the correlation between the HOXA gene family expression in KIRC.

### 2.3. Construction and Evaluation of the Risk Score

To establish the prognostic signature of the HOXA gene family, univariate Cox regression analysis was performed for these genes in the TCGA discovery set, and *P* < 0.005 was set for screening condition. To further minimize the risk of overfitting, significantly, HOXA genes were then subjected to lasso regression analysis using the package “glmnet” [22] in R. The coefficients (*β*) obtained from the previous step were used to generate the following risk score formula: risk score = gene_1_ expression ^*∗*^*β*_1_ + gene_2_ expression ^*∗*^*β*_2_ + … + gene_n_ expression ^*∗*^*β*_n_. Finally, the risk score of each patient was calculated, and according to the cutoff value of the risk score, samples were stratified into low- and high-risk score groups. The optimal cutoff for risk grouping of different cohorts was determined by the “survminer” package in R.

### 2.4. ceRNA Regulatory Network and Machine Learning Model Construction

miRTarbase [23] (http://mirtarbase.mbc.nctu.edu.tw/), an online platform containing more than thousands of miRNA-mRNA target interactions by experimental verification, was used to predict the miRNA interacting with the HOAX gene family. For putative miRNA, only the miRNA-mRNA interaction pairs overlapped with the TCGA differentially expressed miRNA were ultimately selected for downstream analysis. In addition, the possible lncRNAs interacting with filtered miRNA were predicted by retrieving the LncBase database [24] (http://carolina.imis.athena-innovation.gr/). The obtained lncRNAs were also overlapped with TCGA differentially expressed lncRNA to finally establish lncRNA-miRNA-mRNA ceRNA regulatory network. Cytoscape 3.6 provided the visualization of the network, and then we labeled its position in the human reference genome HG19 by “circlize” package [25]. Besides, random forest (RF) is an ensemble algorithm of decision trees, which belong to the branch of ensemble learning. We used the ceRNA network-related genes as classification features to establish a machine learning classification model for predicting possible KIRC patients, which to some degree assisted clinicians to achieve the early screening of KIRC.

### 2.5. Identification of Small Molecule Drugs and Function Annotations in the Treatment of KIRC

To predict the small molecular agents that could attenuate or reverse the influence brought by KIRC, the CMAP database [26] and the DGIdb database [27] were used to mine the putative molecular drugs for the treatment of KIRC. The former database contains whole genomic expression profiles for small active molecular inferences, and the latter covers more than thousands of the correlative information between specific genes and their interacting drugs. Of note, Dr. Insight [28] provides a novel systematic connectivity mapping method to connect drugs in the CMAP data set with query data. Hence, we applied Dr. Insight to establish the mapping relation between DEGs and potential drugs (*P* < 0.05 and FDR <0.1 as the significance threshold), instead of the CMAP database. Based on the provided gene interacted information, the drug that interacted with DEGs in the above two databases would be obtained. Moreover, enrichment analysis with GO terms and KEGG pathways was performed by utilizing the “clusterProfiler” package [29] in R. The top pathways of GO and KEGG enrichment analysis were visualized using the “ggplot2” package.

### 2.6. Statistical Analysis

All statistical tests and packages were implemented by R software (version 3.6.1; https://www.r-project.org/). The difference comparison of two groups was performed by Wilcoxon test, while the difference comparison of multiple groups was conducted by Kruskal test. To investigate whether prognostic signature may be an independent prognostic factor, univariate, multivariate, lasso regression, and Kaplan–Meier analyses were used to construct and evaluate the risk signature by using the R packages “glmnet” and “survival.” ROC curve analysis by using the R package “survivalROC” was conducted to test the performance of the prognostic signature. *P* < 0.05 indicated statistical significance.

## 3. Results

### 3.1. Expression Status and Correlation of the Expression of HOXA Gene Family in the TCGA-KIRC Cohort

**Figure 1:**
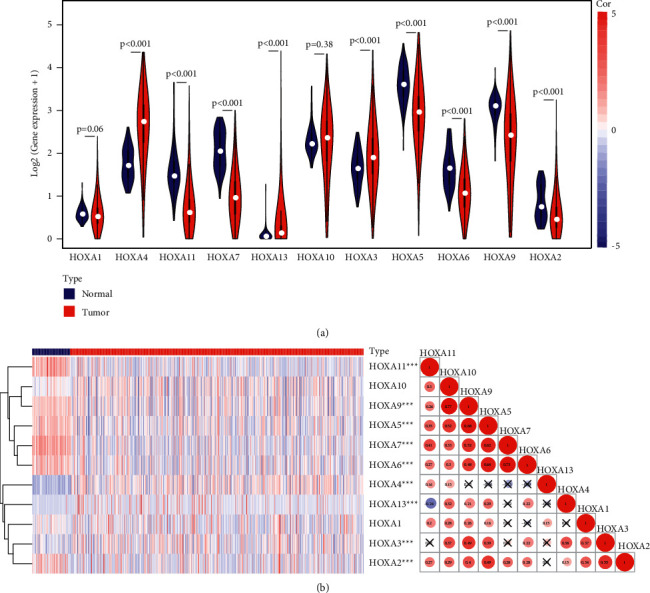
Differentially expressed HOXA genes and correlation analysis results in the TCGA data set: (a) the relative expression levels of HOXA genes across KIRC and normal samples and (b) the heatmap of HOXA genes between KIRC and normal samples is shown on the left, corresponding to Spearman correlation analysis of the HOXA genes on the right. “X” and different colors represent *P* > 0.05 and correlation coefficients, respectively.

We firstly explored the expression patterns of each HOXA gene in the TCGA-KIRC expression profile. As shown in Figure 1(a), almost all of HOXA genes were expressed aberrantly in KIRC samples compared to normal samples. Among these genes, HOXA4, HOXA13, and HOXA3 were expressed higher in KIRC samples than in normal samples, while HOXA11, HOXA7, HOXA5, HOXA5, HOXA6, HOXA9, and HOXA2 were expressed lower (*P* < 0.001). Unlike the above genes, the expression levels of HOXA1 and HOXA10 were of no statistical difference between normal and KIRC samples (*P* > 0.05). Besides, Spearman correlation analysis was further performed to explore the interaction among the HOXA gene family. Most of the HOXA genes were correlated with each other positively, whereas the expression of HOXA13 showed a significant negative correlation with HOXA11 (Figure 1(b)), which might be related to the target action of HOXA13 with other HOX genes [30]. These findings demonstrated that the inconsistent expression level of the HOXA gene family possessed important roles in the KIRC occurrence and development.

### 3.2. Establishment and Verification of the HOXA Prognostic Model

**Figure 2:**
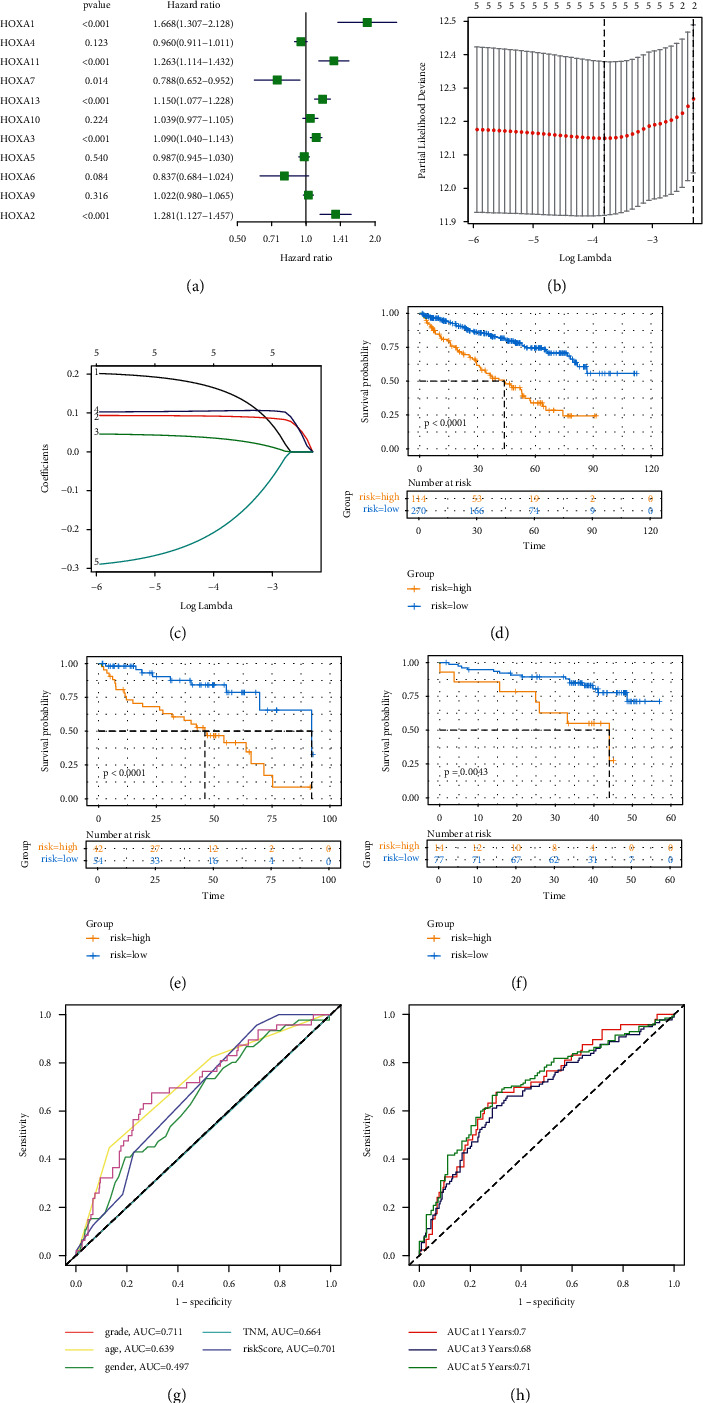
Establishment and verification of the prognostic signature of HOXA genes in the TCGA database: (a) univariate Cox regression analysis of HOXA genes; (b) lasso model with tenfold cross-validation; (c) lasso coefficient profiles of the six HOXA genes; (d–f) Kaplan–Meier curves of prognostic value by the risk score in the discovery set, the test set, and the validation set, respectively; (g) the AUC for risk model score and clinical features according to the ROC curves (clinical features: age, clinical grade, gender, and TNM stage); and (h) the risk score is measured by the time-dependent receiver-operating characteristic curves at 1, 3, and 5 years.

Considering the potential clinical application of the HOXA gene family in the progression of KIRC, we attempted to uncover the prognostic and diagnostic value of HOXA genes. Firstly, we matched the HOXA gene family expression profiles with clinical information to obtain a complete prognostic model input profile consisting of 480 patients. The filtered TCGA-KIRC patients were split at random into the discovery set (*n* = 384; 70%) and test set (*n* = 96; 30%). Secondly, we conducted the univariate Cox regression analysis in the TCGA discovery set, and the results showed that six of these genes were clearly related to OS (*P* < 0.05; Figure 2(a)). Among these OS-related genes, HOXA7 acted as protective roles with HR < 1, while HOXA1, HOXA11, HOXA13, HOXA3, and HOXA2 played as risky factors with HR > 1. Subsequently, after removing the insignificantly HOXA1 gene between KIRC samples and normal samples, five OS-related genes were subjected to the lasso regression model to reduce the risk of overfitting (Figures 2(b) and 2(c)). Finally, a five-gene prognostic signature consisting of HOXA11, HOXA13, HOXA7, HOXA3, and HOXA2 was constructed. Applying the coefficients obtained from the lasso regression, the risk score for each patient was calculated, and the formula for the risk score was as follows: Exp_(HOXA11)_ ^*∗*^ 0.1544 − Exp_(HOXA7)_^*∗*^ 0.1823 + Exp_(HOXA13)_ ^*∗*^ 0.0912 + Exp_(HOXA3)_ ^*∗*^ 0.0326 + Exp_(HOXA2)_ ^*∗*^ 0.1055. According to the optimate cutoff of risk score, patients were stratified into low- and high-risk score groups.

We further evaluated the performance of the prognosis model in TCGA-KIRC discovery and test sets, as well as in external validation sets (consisting of 91 KIRC patients). Either one of the sets showed all-right survival prediction ability (Figures 2(d)–2(f)). Specifically, the survival risk curve showed that patients in the high-risk score group had an obviously shorter overall survival time compared with patients in the low-risk score group (*P* < 0.005). Moreover, to compare our model with the existing model in predicting the survival rate, we constructed the ROC curve of the HOXA signature, TNM stage, age, gender, and clinical grade. The area under the curve (AUC) of the HOXA signature was 0.701 (Figure 2(g)), which showed that our prognostic signature had reliable predictive power and was similar to the AUC of clinical grade. Then we analyzed the time-dependent ROC curve of the HOXA signature. We found that the area under the HOXA signature curve: 1 year: 0.70, 3 years: 0.68, and 5 years: 0.71 (Figure 2(h)). Of note, clinical grade and TNM stage were known as the prognostic criteria in KIRC. Compared with clinical-grade ROC (AUC at 1, 3, and 5 years were 0.71, 0.69, and 0.67, respectively), the HOXA prognostic model had similar prognostic performance but had a more prominent prognostic capability at 5 years. Compared with TNM stage ROC (AUC at 1, 3, and 5 years were 0.56, 0.56, and 0.55, respectively), the HOXA prognostic model consistently delivered better prognostic performance (Supplementary Figure 2). These all indicated that our model had a good predictive ability for patients with 1-, 3-, and 5-year survival.

### 3.3. Prognostic Signature, as an Independent Prognostic Factor, Correlated with Disease Progression

**Figure 3:**
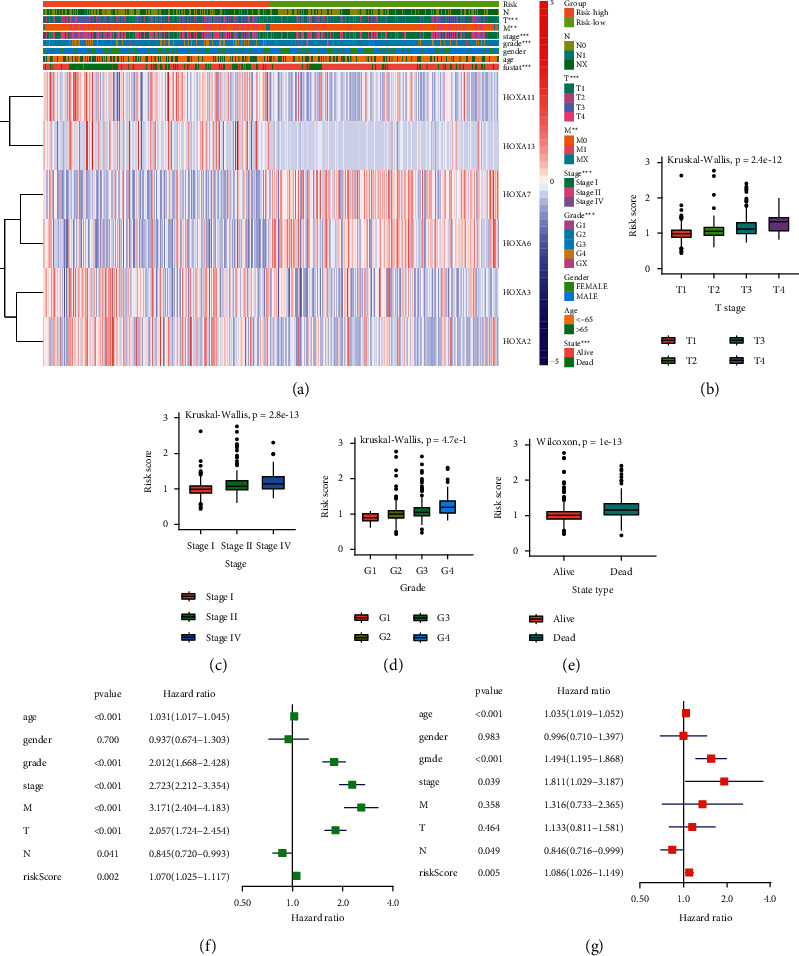
The correlation of prognostic signature with clinical variables and further verification of the six-gene prognostic signature. (a) The heatmap between prognostic signature and clinical variables of the low- and high-risk score group. (b–e) The distribution of risk score in each clinical variable: (b) T stage, (c) stage type, (d) grade type, and (e) status type. (f, g) Forest plot for the prognostic signature and clinical variables by the univariate and multivariate Cox regression analysis. ^*∗∗∗*^*P* < 0.001 and ^*∗∗*^*P* < 0.01.

For observing the association between the risk score and clinicopathological features, we quantitatively examined the expression levels of prognostic signatures in high-/low-risk score groups (Figure 3(a)). We found that the HOXA score was clearly correlated with survival status, stage, grade, and T stage (*P* < 0.001; Figures 3(b)–3(e)). Specifically, as the HOXA score gradually increased, the patient's disease state became more severe. To investigate whether the above prognostic signature could be used as an independent prognostic factor, we implemented univariate and multivariate Cox regression analyses of the risk score and relevant clinical variables in the TCGA-KIRC cohort (Figures 3(f) and 3(g)). In the univariate analysis, we found that the risk score was significantly correlated with the overall survival (OS; HR = 1.070, 95% CI = 1.025–1.117, *P* < 0.005). Multivariate analysis shows that the risk score is an effective independent prognostic predictor of OS (HR = 1.086, 95% CI = 1.026–1.149, *P* < 0.005). Besides, the Kaplan–Meier survival curves were also applied to evaluating single prognostic roles of the five prognosis-related HOXA genes (Supplementary Figure 3). These results reflected that the prognostic signature based on the HOXA gene family was an independent prognostic predictor for KIRC and significantly correlated with disease progression.

### 3.4. Constructing the ceRNA Regulatory Network and Machine Learning Model

**Figure 4:**
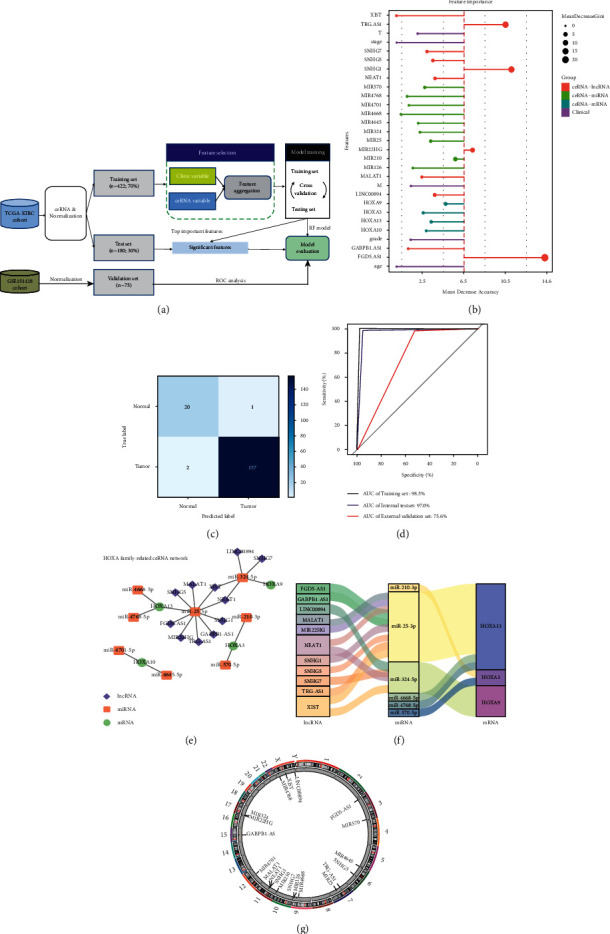
The construction of ceRNA regulatory network and machine learning classifier. (a) Flowchart of developing RF machine learning model. Based on the ceRNAs and clinicopathological features as the input of the model, the model was trained with cross-validation using a training set (*n* = 422) after normalization. And then the trained model was further applied to assessing generalization and performance using the independent testing set (*n* = 180) and the external validation set (*n* = 75). (b) The important features ranked by RF model. Mean decrease accuracy is shown along the horizontal axis, the value of which means the average impact of features in the model. The stacked bar indicates the different classes of features. (c) Confusion matrix for classifying the KIRC sample in the independent testing set. (d) Density plot of ROC for classifying the KIRC sample in the training set, the testing set, and the external validation set. (e) The HOXA genes related to ceRNA network in KIRC. Red squares represent miRNAs; green ellipses represent HOXA mRNAs; and blue diamonds represent lncRNAs. (f) Sankey diagram for the ceRNA network in KIRC. Each rectangle represents a gene, and the connection degree of each gene is visualized based on the size of the rectangle. (g) The relative position of ceRNAs in the human reference genome HG19.

To establish the HOXA-associated ceRNA regulatory network, we predicted the interactions among DE-miRNAs, DE-lncRNAs, and DE-mRNAs by using bioinformatics tools. miRNAs that interacted with the HOXA gene family were obtained from the miRTarbase database. After we discarded miRNAs that did not include in DE-miRNAs, eight miRNAs were selected as the ceRNA-miRNAs for follow-up research. Then, we utilized the LncBase database to predict the miRNA-LncRNA interaction pairs and then found the eleven lncRNAs that were predicted to interact with the above miRNAs. Ultimately, eleven lncRNAs, eight miRNAs, and four HOXA genes were included in the ceRNA network. Based on the above findings, we systematically constructed and visualized the ceRNA regulatory network using Cytoscape 3.6. Figures 4(e)–4(f) show that four miRNAs, two mRNAs, and eleven lncRNAs are involved in one ceRNA network; only two single HOXA-miRNA pairs were involved in another ceRNA network. The position of the ceRNA elements in the human reference genome HG19 was labeled in Figure 4(g).

Moreover, we applied the classifier RF to establish a machine learning model based on multiple related features, aiming at unsupervised early diagnosis of KIRC patients. Briefly, we randomly stratified samples for the training set (70%) and the independent test set (30%) (Figure 4(a) and Supplementary Table S2). The ceRNAs expression level and clinicopathological features were regarded as the input feature of the model and then normalized. We selected the optimal hyperparameters by tenfold cross-validation, trained the final model, and evaluated the model in an internal and external test set. A more detailed model construction process was provided in Supplementary Figure 4. Our training results showed strong generalizable discrimination among the two classes, with a training set AUC of 0.985 and a test set AUC of 0.970 (Figures 4(c) and 4(d)). The confusion matrix showed that almost all patients in the test set were correctly identified, except for two normal patients who were predicted to have the tumor. Furthermore, by introducing an external validation data set from the GEO database, our model still achieved good classification performance even when clinical input features are completely missing, with a validation set AUC of 0.756. The importance of different features in the model was prioritized and ranked by average decrease accuracy, and we further found that several lncRNAs and miRNAs (such as XIST, TRG-AS1, SNHG5, etc.) showed the ability of better classification than other features (Figure 4(b)). In general, these results demonstrated that early screening models based on HOXA-ceRNA regulatory networks had good predictive power and suggested that some important HOXA-related biomarkers deserved attention in the KIRC progression.

### 3.5. Mining of the Small Molecule Drug and Function Annotations

**Figure 5:**
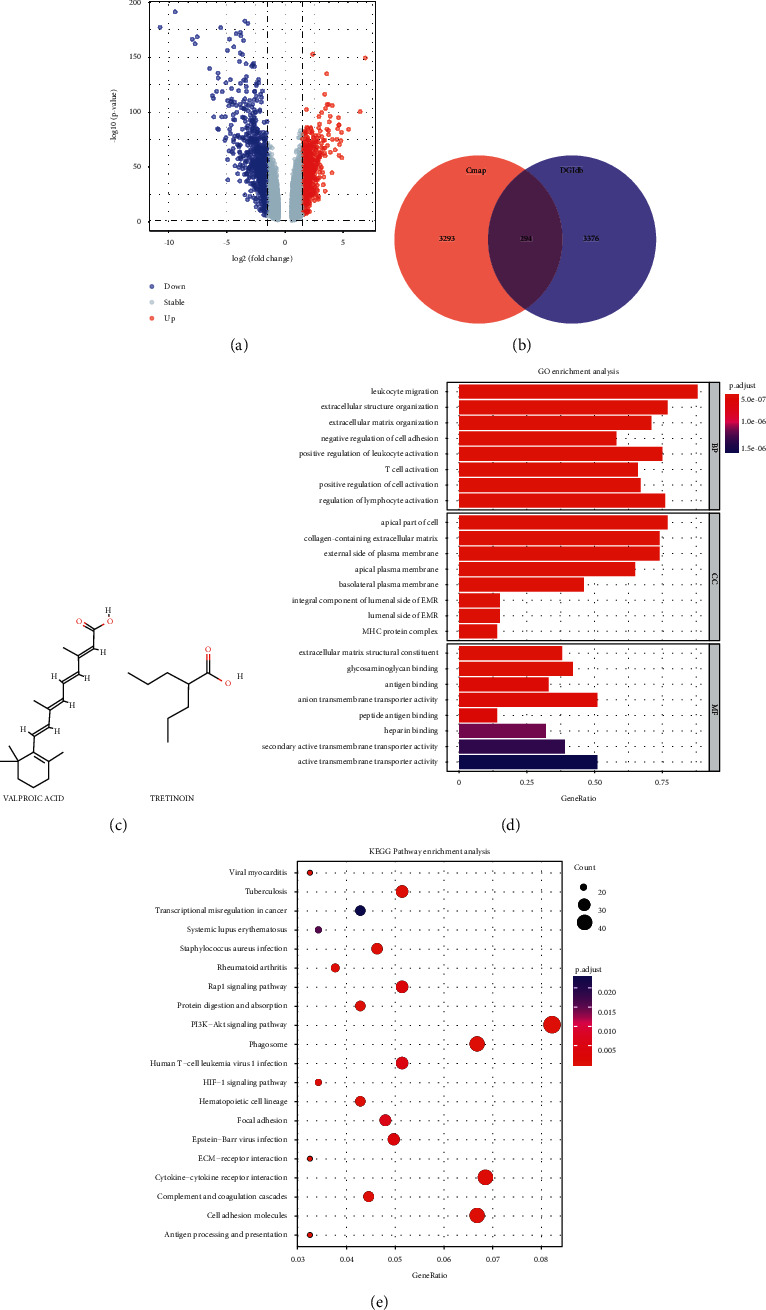
The discovery of small molecule drugs and functional annotations. (a) Volcano plot. According to the standard of LogFC ≥ 1.0 and *P* < 0.05, red represented upregulated genes, and blue represented downregulated genes. (b) The intersection of putative small molecules for the treatment of KIRC, small molecule obtained from CMAP and DGIdb database. (c) The chemical structures of valproic acid and tretinoin were retrieved from the PubChem database. (d) GO enrichment entries of DEGs between KIRC samples and normal samples. (e) KEGG pathway enrichment entries of DEGs in the top 20.

To predict the potential small molecule drug in the treatment of KIRC, a total of 1,373 DEGs (669 upregulated and 704 downregulated) were identified between 539 tumor samples and 72 normal controlled samples, as shown in the volcano (Figure 5(a)). The DEGs were submitted to the CMAP database and the DGIdb database, obtaining 3,587 and 3,670 types of putative drugs, respectively. The intersection of 2 databases showed 294 type possible small molecule drugs (Figure 5(b) and Supplementary Table S3). After filtering by the threshold of *P* value and FDR, only 2 possible small molecule drugs, named valproic acid and tretinoin, were finally identified. Their chemical structure was retrieved from the PubChem database and shown in Figure 5(c). Detailed annotated information about these small molecule drugs is listed in Table 2. Moreover, in order to explore insight into the potential molecular mechanisms of 2 drugs, enrichment analysis (GO terms and KEGG pathways) of DEGs was carried out. The results indicated that DEGs were notably associated with many signaling pathways related to cancer and immunity, such as cytokine-cytokine receptor interaction and PI3K-Akt signaling pathway (Figures 5(d) and 5(e)). Abovementioned results provided possible molecular targets and signaling pathways for subsequent researches of drug interventions in KIRC progression.

## 4. Discussion

In addition to the HOXA gene family role in regulating embryonic development and cell fate, gene members also play an important role in tumor genesis, progression, and patient prognoses, such as hepatocellular carcinoma [31], epithelial ovarian cancer [32], leukemia [33, 34], and gastric cancer [35]. KIRC is a heterogeneous disease with complex biological characteristics, the incidence of which is increasing rapidly worldwide. Advances in surgical techniques and comprehensive treatment techniques have improved the local control rate and quality of life of KIRC patients. Still, in recent decades, the survival rate has not increased significantly. KIRC still needs to find accurate biomarkers for early diagnosis and a more accurate prognosis. Hence, a comprehensive and in-depth exploration of the role of the HOXA gene family in KIRC development is necessary to be performed.

In our study, we aimed at investigating the expression pattern of the HOXA gene family to uncover the association between these gene members and prognosis and clinical application in KIRC. By analyzing the TCGA-KIRC expression profile from the open-access TCGA database, a total of 1,373 DEGs showed a considerable difference between the two groups, which included 9 HOXA gene family members. By utilizing univariate Cox regression analysis, 5 HOXA genes were found to be associated with the prognosis significantly, including HOXA2, HOXA3, HOXA7, HOXA11, and HOXA13. Currently, numerous studies have demonstrated that the above HOXA genes played a critical role in multiple cancers. For instance, the results of Heller and Eoh et al. identified HOXA2 as potential prognostic markers and therapeutic targets for non-small cell lung cancer and cervical cancer patients [36, 37]. Li et al. found that epigenomic modifications of two HOXA gene family members (HOXA2 and HOXA5) were closely associated with the clinical manifestations of non-small cell lung cancer patients [38]. HOXA7 significantly enhanced proliferation, migration, invasion *in vitro*, and tumor growth and metastasis *in vivo* in liver cancer. The activation of *Snail* molecules was an important mechanism for HOXA7 to perform its oncogenic characteristics for liver cancer cells [39]. Moreover, HOXA11 regulated RCC cells apoptosis by inhibiting Wnt signaling in renal cell carcinoma, and its function was regarded as a tumor suppressor in RCC [40]. HOXA13, as a novel oncogenic gene in KIRC, was proved to accelerate cancer cell proliferation in a p53-dependent way [41]. A more detailed summary of the different roles of HOXA family members in KIRC was illustrated in Supplementary Table S4. Some HOXA genes whose functions have been researched, such as HOXA3, HOXA9, and HOXA13, were mostly confirmed to have significant prognostic values in our research and then introduced to the subsequent establishment of the diagnostic model. Such evidences also efficiently improve the interpretability of our machine learning model. In clinical observation aspects, we found the expression of the aforementioned genes was changed as the disease stage and ages at clinic diagnosis developed, indicating the expression levels of these genes are highly correlated with malignant progression of KIRC. Collectively, our study is the first to link these genes to the prognosis and clinical features of KIRC, providing clues for further study of the molecular mechanisms involved.

Prognostic-associated HOXA genes were identified as independent prognostic factors for KIRC patients by multivariate Cox regression analysis. We classified KIRC patients into high- and low-risk score groups to precisely predict clinical outcomes. And we further verified that risk score, age, tumor T stage, and clinical grade were independent prognostic factors, which provided additional pathway options for the prognosis of KIRC patients. Moreover, stratification analysis demonstrated that the high-risk subgroup had worse OS compared to the low-risk subgroup, which was almost consistent with the trend observed in clinical. It was worth noting that we found HOXA13 and HOXA3 were upregulated in tumor samples compared with normal samples. HOXA13 and HOXA3 also were risk factors (hazard ratio >1), which were upregulated in the high-risk subgroup. The Kaplan–Meier survival curves showed that higher expression of HOXA13 and HOXA3 were linked to poorer survival outcomes. These results suggested that they might act as tumor suppressors in KIRC. On the contrary, HOXA7 was downregulated in tumor tissues and regarded as a protective factor (hazard ratio <1), which were upregulated in the low-risk subgroup. And the lower expression of HOXA7 was linked to poorer survival outcomes. These results suggested that it might act as a tumor promoter in KIRC. Cai et al. demonstrated similar results in their study, which presented the negative prognostic role of HOXA13 in KIRC [41]. The roles of the remaining three HOXA genes in tumors have not been reported. Furthermore, verifying HOXA gene members' function and mechanism by molecular biology experiments methods was necessary to be performed.

The ceRNA hypothesis provided a novel guiding theory and suggested valuable strategies for the diagnosis and treatment of malignancies. Herein, we constructed a HOXA-related lncRNAs-miRNAs-mRNAs ceRNA regulatory network based on the TCGA-KIRC expression profile. By integrating the ceRNAs and clinical features as input variables, we successfully constructed an RF classifier to achieve the early screening of KIRC patients and performed well in the test and validation set. According to the results of the prediction model, partial lncRNAs and miRNAs have better categorization than the other features. These features such as FGD5-AS1, SNHG1, TRG-AS1, and MIR22HG have higher mean accuracy in model evaluation and thus may potentially yield crucial diagnostic biomarkers for early screening KIRC patients. Currently, numerous studies have described the functional role of these genes in the pathogenesis of KIRC. For example, Yang et al. found that FGD5-AS1 expression was significantly lower in the KIRC sample than in adjacent normal tissues, and increased expression of FGD5-AS1 was associated with longer OS. They believed that FGD5-AS1 could serve as a valuable diagnostic and prognostic marker in KIRC [42]. Zhao et al. reported that SNHG1 promoted KIRC progression and metastasis by negatively regulating miR-137 [43]. MIR22HG was first reported by Tani et al. [44] and commonly downregulated in tumor tissues and participated in the inhibition of cell proliferation. Abnormal expression of MIR22HG was associated with many tumors, including lung cancer, breast cancer, and thyroid cancer [45, 46]. Using these features, we could optimize existing approaches to improve the accuracy and sensitivity of auxiliary detection and prevent progression in time to minimize individual, medical, and social costs.

Based upon the DEGs and drug interactions from the CMAP and DGIdb databases, we reported several putative small molecule drugs that displayed anticancer activity. Valproic acid, which was used as an anticonvulsant drug for years, was related to inhibitions on cell proliferation and differentiation, cell cycle control, and cell apoptosis in KIRC. Valproic acid could activate ERK and AKT proteins and perform antitumor effects by regulating cell apoptotic signal pathways [47, 48]. Recent research reported that the combination strategy of valproic acid and metformin enhanced the antitumor activity and surprisingly overcame the resistance induced by the long-term use of metformin in KIRC cells [49]. Tretinoin, a derivative of vitamin A, functions via targeting the retinoic acid receptor alpha (RAR*α*) mediated signals to regulate cell apoptosis [50]. Shi et al. reported that tretinoin could be a novel promising drug to overcome RCC resistance by regulating TR4/lncTASR/AXL signaling pathway while also aiding in enhancing sunitinib efficacy in RCC treatment [51]. Subsequently, the outcomes of enrichment analysis revealed that the PI3K-Akt signaling pathway might be the most probably putative action mechanisms of future drug development. Considering this, further researches were still required to investigate their suitability as broad-spectrum antitumor auxiliary drugs in KIRC.

As far as we know, this work is the first to research the prognostic value and clinical application of the HOXA gene family in KIRC. We identified a novel risk prognostic model consisting of five HOXA gene family members (HOXA2, HOXA3, HOXA7, HOXA11, and HOXA13) in KIRC. Furthermore, based on HOXA-ceRNA regulatory network, we constructed an efficiently machine learning early screening model for KIRC patients. These developments may open a new chapter in KIRC prognosis and diagnosis.

## Figures and Tables

**Table 1:** Clinicopathological variables of patients in TCGA discovery and test sets.

Variables	Type	Total No. (%)
No. of patients	≤60	487 249 (51.12)
Age	>60	238 (48.88)
Gender	Female	169 (34.70)
	Male	318 (65.30)
Grade	G1-2	211 (43.32)
	G3-4	271 (55.64)
	Unknown	5 (1.04)
Stage	Stage I	241 (49.49)
	Stage II	164 (33.67)
	Stage IV	82 (16.84)
T stage	T1-2	311 (63.86)
	T3-4	176 (36.14)
M stage	M0	400 (82.13)
	M1	80 (16.43)
	Unknown	7 (1.44)
N stage	N0	216 (44.35)
	N1	16 (3.29)
	Unknown	255 (52.36)

Abbreviations: T, tumor size; M, metastasis.

**Table 2:** Antineoplastic drugs targeting DEG based on the CMAP database and the DGIdb database.

Name	Cell line	*P* value	FDR	Description
Valproic acid	HL60	4.88E-08	5.84E-05	Histone deacetylases and nitric oxide synthase inhibitor
Tretinoin	MCF7	0.0017	0.087129	Regulators of cell reproduction, proliferation, and differentiation

## Data Availability

The data used to support the findings of this study are available from the corresponding author upon request.

## Abbreviations

KIRC: Kidney renal clear cell carcinoma
HOXA: Homeobox-A
TCGA: The Cancer Genome Atlas
ceRNAs: Competing endogenous RNAs
ROC: Receiver operating characteristic
AUC: Area under curve
OS: Overall survival rate
DEGs: Differentially expressed genes
mRNA: Messenger RNA
miRNA: Micro RNA
lncRNA: Long non-coding RNA
RF: Random forest
GO: Gene ontology
KEGG: Kyoto encyclopedia of genes and genomes.

## Supplementary Materials

Supplementary Materials
*Supplementary Figure 1*. Flow chart of data collection and subsequent model establishment in our study; *Supplementary Figure 2*. The TNM stage and clinical grade measured by the time-dependent receiver-operating characteristic curves at 1, 3, and 5 years. *Supplementary Figure 3*. The Kaplan–Meier survival curves of evaluating single prognostic roles of the prognosis-related HOXA genes. *Supplementary Figure 4*. A more detailed machine learning model construction process for early screening classification. *Supplementary Figure 5*. Custom scripts for prognostic model and machine learning model construction in this study; Table S1: differentially expressed genes between KIRC samples and paired normal samples, including the differential expression HOXA genes; Table S2: the training set, the test set, and the external validation set for machine learning model; Table S3: small molecule drugs by querying the CMAP and DGIdb databases; and Table S4: a detailed summary of the different roles of HOXA family members in KIRC.
