# Supplementary material for: The Diagnostic and Prognostic Values of HOXA Gene Family in Kidney Clear Cell Renal Cell Carcinoma
Source: J Oncol. 2022 Mar 16;2022:1762637. doi: 10.1155/2022/1762637 (PMC8942704; doi:10.1155/2022/1762637)
Supplement: Supplementary Materials — Supplementary Figure 1. Flow chart of data collection and subsequent model establishment in our study; Supplementary Figure 2. The TNM stage and clinical grade measured by the time-dependent receiver-operating characteristic curves at 1, 3, and 5 years. Supplementary Figure 3. The Kaplan–Meier survival curves of evaluating single prognostic roles of the prognosis-related HOXA genes. Supplementary Figure 4. A more detailed machine learning model construction process for early screening classification. Supplementary Figure 5. Custom scripts for prognostic model and machine learning model construction in this study; Table S1: differentially expressed genes between KIRC samples and paired normal samples, including the differential expression HOXA genes; Table S2: the training set, the test set, and the external validation set for machine learning model; Table S3: small molecule drugs by querying the CMAP and DGIdb databases; and Table S4: a detailed summary of the different roles of HOXA family members in KIRC. [file 1762637.f1.zip › 1762637.f1/Supplementary Figure 4 (1).pdf]

SUPPLEMENTARY MATERIALS

Data Preprocessing for Machine Learning

Random Forest (RF)[1], an ensemble algorithm of decision trees, was developed to distinguish kidney renal clear cell carcinoma (KIRC) patients and normal patients based on mRNA transcripts (n=24, 4 HOXA gene family members, 9 miRNA, and 11 LncRNA, all derived from elements in the ceRNA regulatory network) and clinical features (n=7, including age, grade, clinic stage, gender, and TNM) from TCGA-KIRC cohort. The open-sourced R package ("randomForest", version = 4.6) was used to perform this progress. We employed random stratified sampling to select 422 patients (70% of patient cohort) as the training set (tumor samples = 370, and normal samples = 52), while the remaining 180 patients were used as the independent testing set (**Figure 4A)**. Moreover, to ensure reproducible results, we obtained an external validation set from the GEO database (**GSE151428**), consisting of 17 normal and 58 KIRC samples. A fixed random number seed was also used to ensure reproducibility of the results. The data in three feature sets was normalized by Log2 transformation.

Feature Selection

Due to irrelevant features were trained and thus may decrease model's performance, we firstly pre-trained all the original features based on the training set. And then based on the ranking of the features importance with average reduced accuracy in model, we filtered out the variables with weaker discriminatory power, including gender and T stage. We finally trained our early screening model using features containing 5 clinically relevant variables and 24 HOXA-ceRNA elements' variables. The importance ranking of the output of the final trained model is shown in **Figure 4B**. Besides, in addition to clinically relevant features, the functions of these genes included in our model are described individually in the discussion section of the text.

Model Training and Model Evaluation

We performed 10-fold cross validation to optimize RF parameters. Two core model parameters were identified as follows: the number of trees was set to 120, and the number of features randomly sampled as candidates was set to 8. To evaluate overall performance of our model, ROC curves with AUROC values for each class and confusion matrices (predicted label as the index of maximum value of the predicted probability vector) were generated for evaluating the performance in the training set, the test set and the validation set, respectively (**Figure 4C,D)**. Of note, even when the validation set is missing clinical features and only genetic features are retained, our model can still achieve good classification results, with a validation set AUC of 0.756. (**Figure 4D**).

Data Visualization

All the figures in our manuscript were plotted using R packages of ggplot2 (version 0.9.0).

Data and source code availability

The data that support the findings of this study, including the training set, the test set, and the validation set have been deposited in the **Supplementary Table S2**. Custom scripts for early screening model in this study were present in **Supplementary Figure 5**.

References

1. Svetnik, V.; Liaw, A.; Tong, C.; Culberson, J. C.; Sheridan, R. P.; Feuston, B. P., Random forest: a classification and regression tool for compound classification and QSAR modeling. *J Chem Inf Comput Sci* **2003,** *43* (6), 1947-58.
