# Supplementary material for: The Diagnostic and Prognostic Values of HOXA Gene Family in Kidney Clear Cell Renal Cell Carcinoma
Source: J Oncol. 2022 Mar 16;2022:1762637. doi: 10.1155/2022/1762637 (PMC8942704; doi:10.1155/2022/1762637)
Supplement: Supplementary Materials — Supplementary Figure 1. Flow chart of data collection and subsequent model establishment in our study; Supplementary Figure 2. The TNM stage and clinical grade measured by the time-dependent receiver-operating characteristic curves at 1, 3, and 5 years. Supplementary Figure 3. The Kaplan–Meier survival curves of evaluating single prognostic roles of the prognosis-related HOXA genes. Supplementary Figure 4. A more detailed machine learning model construction process for early screening classification. Supplementary Figure 5. Custom scripts for prognostic model and machine learning model construction in this study; Table S1: differentially expressed genes between KIRC samples and paired normal samples, including the differential expression HOXA genes; Table S2: the training set, the test set, and the external validation set for machine learning model; Table S3: small molecule drugs by querying the CMAP and DGIdb databases; and Table S4: a detailed summary of the different roles of HOXA family members in KIRC. [file 1762637.f1.zip › 1762637.f1/Supplementary Figure 5 (1).pdf]

```
#################prognostic model###################
library("glmnet")
library("survival")

setwd("D:\\Documents\\Desktop\\Mai\\new\\21.lasso")
#Discovery set or test set of prognostic model
rt=read.table("ENSG_risk.txt",header=T,sep="\t",row.names=1)
rt$futime=rt$futime/36

#gene list
gene=read.table("gene.txt",header=F)
rt=rt[,c("futime","fustat",as.vector(gene[,1]))]

x=as.matrix(rt[,c(3:ncol(rt))])
y=data.matrix(Surv(rt$futime,rt$fustat))

fit <- glmnet(x, y, family = "cox", maxit = 1000)
pdf("lambda.pdf")
plot(fit, xvar = "lambda", label = TRUE)
dev.off()

cvfit <- cv.glmnet(x, y, family="cox", maxit = 1000)
pdf("cvfit.pdf")
plot(cvfit)
abline(v=log(c(cvfit$lambda.min,cvfit$lambda.1se)),lty="dashed")
dev.off()

coef <- coef(fit, s = cvfit$lambda.min)
index <- which(coef != 0)
actCoef <- coef[index]
lassoGene=row.names(coef)[index]
geneCoef=cbind(Gene=lassoGene,Coef=actCoef)
write.table(geneCoef,file="geneCoef2.txt",sep="\t",quote=F,row.names=F)

riskScore=predict(cvfit, newx = x, s = "lambda.min",type="response")
outCol=c("futime","fustat",lassoGene)
risk=as.vector(ifelse(riskScore>median(riskScore),"high","low"))
outTab=cbind(rt[,outCol],riskScore=as.vector(riskScore),risk)
write.table(cbind(id=rownames(outTab),outTab),
```

```
    file="ENSG_riskscore.txt",
    sep="\t",
    quote=F,
    row.names=F)

#################machine-learning early screening model####################
library("devtools")
library("dplyr")
library("pROC")
library("randomForest")
set.seed(8989)
runif(6)
setwd("E:/final_version_mai/RF")
expr_file <- "readforml.csv"
expr_mat <- read.csv(expr_file,header = TRUE, row.names = 1)
metadata <- as.data.frame(expr_mat[,"y"])
rownames(metadata) <- rownames(expr_mat)
colnames(metadata) <- "class"
metadata$class <- as.factor(metadata$class)
######################################Remove label######################################################
 <- which(colnames(expr_mat)== "y")
expr_mat <- expr_mat[,-k]
SAMPLE_NUM <- length(expr_mat[,1])
######################################normalization: log2(x+1)##########################################
expr_mat[,6:29] <- log2(expr_mat[,6:29]+0.1)
numberize <- function(){
  expr_mat$age[c(grep(">65",expr_mat$age))] <- 1
  expr_mat$age[c(grep("<=65",expr_mat$age))] <- 0
  expr_mat$gender[c(grep("FEMALE",expr_mat$gender,fixed = TRUE))] <- 0
  expr_mat$gender[c(grep("MALE",expr_mat$gender,fixed = TRUE))] <- 1
  expr_mat$grade[c(grep("G1",expr_mat$grade))] <- 0
  expr_mat$grade[c(grep("G2",expr_mat$grade))] <- 2
  expr_mat$grade[c(grep("G3",expr_mat$grade))] <- 3
  expr_mat$grade[c(grep("G4",expr_mat$grade))] <- 4
  expr_mat$grade[c(grep("GX",expr_mat$grade))] <- 1
  expr_mat$stage[c(grep("Stage I",expr_mat$stage))] <- 1
  expr_mat$stage[c(grep("Stage II",expr_mat$stage))] <- 2
  expr_mat$stage[c(grep("Stage IV",expr_mat$stage))] <- 3
  expr_mat$M[c(grep("M0",expr_mat$M))] <- 1
```

```
  expr_mat$M[c(grep("M1",expr_mat$M))] <- 2
  expr_mat$M[c(grep("MX",expr_mat$M))] <- 3
  expr_mat$T[c(grep("T1",expr_mat$T))] <- 0
  expr_mat$T[c(grep("T2",expr_mat$T))] <- 1
  expr_mat$T[c(grep("T3",expr_mat$T))] <- 2
  expr_mat$T[c(grep("T4",expr_mat$T))] <- 3
  #expr_mat$N[c(grep("N0",expr_mat$N))] <- 0
  #expr_mat$N[c(grep("N1",expr_mat$N))] <- 1
  #expr_mat$N[c(grep("NX",expr_mat$N))] <- 2
  write.csv(expr_mat,file = "expr_mat_numerized.csv")
}
numberize()
expr_mat <- read.csv("expr_mat_numerized.csv",header = TRUE, row.names = 1)


########################################Dividing the dataset into Trainset and Testset###################
ratio = 3/10
generate_training_set_and_test_set <- function(ratio){
  NORMAL_SEQ <- grep("normal",metadata$class)
  TUMOR_SEQ <- grep("tumor",metadata$class)
  TEST_NUM <- floor(SAMPLE_NUM*ratio)
  TEST_NUM_NORMAL <- floor(TEST_NUM*length(NORMAL_SEQ)/SAMPLE_NUM)
  TEST_NUM_TUMOR <- TEST_NUM - TEST_NUM_NORMAL
  TEST_NORMAL_SEQ <- sample(NORMAL_SEQ, TEST_NUM_NORMAL, replace = FALSE, prob = NULL)
  TEST_TUMOR_SEQ <- sample(TUMOR_SEQ, TEST_NUM_TUMOR, replace = FALSE, prob = NULL)
  test_set <- rbind(expr_mat[TEST_NORMAL_SEQ,],expr_mat[TEST_TUMOR_SEQ,])
  i = 1
  test_set_list <-c()
  for (i in 1:length(test_set[,1])){
    rownames(test_set[i,])
    a <- which(rownames(expr_mat) == rownames(test_set[i,]))
    test_set_list <- append(test_set_list,a)
  }
  training_set <- expr_mat[-c(test_set_list),]
  write.csv(training_set,file = "training_set.csv")
  write.csv(test_set,file = "test_set.csv")

}
```

```r
generate_training_set_and_test_set(ratio = ratio)

#################################### 10 fold Cross validation   #######################################################
K = 10
m = nrow(expr_mat)
kfold <- sample(rep(1:K, length.out=m), size=m, replace=F)

randomForest2 <- function(x, y, xtest, ytest, type="response", ...){
  model <- randomForest(x, y, ntree = 120 ,mtry = 8, importance = TRUE, ...)
  preds <- predict(model, xtest, type=type)
  return(data.frame(preds, real=ytest))
}

randomForest3 <- function(x, y, xtest, ytest, type="response", ...){
  model <- randomForest(x, y, ntree = 120 ,mtry = 8, importance = TRUE, ...)
  preds <- predict(model, xtest, type=type)
  return(data.frame(preds, real=ytest))
}

rf2 <- lapply(1:K, function(x, ...){
  training_set2 = expr_mat[kfold != x,]
  metadata_training_set2 = metadata$class[kfold!=x]

  test_set2 = expr_mat[kfold == x,]
  metadata_test_set2 = metadata$class[kfold==x]

  randomForest2(x=training_set2, y=metadata_training_set2, xtest=test_set2, ytest=metadata_test_set2, ...)
})

kfold_estimate <- do.call(rbind, rf2)
write.csv(kfold_estimate,"kkk.csv")

######################################Start From Now!!!####################################################################

training_set <- read.csv("training_set.csv",header = TRUE, row.names = 1)
test_set <- read.csv("test_set.csv",header = TRUE, row.names = 1)

training_set_list <- c()
test_set_list <- c()
```

```
for (i in 1:length(training_set[,1])){
  rownames(training_set[i,])
  a <- which(rownames(metadata) == rownames(training_set[i,]))
  training_set_list <- append(training_set_list,a)
}
for (i in 1:length(test_set[,1])){
  rownames(test_set[i,])
  a <- which(rownames(metadata) == rownames(test_set[i,]))
  test_set_list <- append(test_set_list,a)
}
metadata_training_set <- as.data.frame(metadata[training_set_list,1],row.names = rownames(expr_mat[training_set_list,]))
metadata_test_set <- as.data.frame(metadata[test_set_list,1],row.names = rownames(expr_mat[test_set_list,]))
colnames(metadata_test_set) <- "class"
colnames(metadata_training_set) <- "class"
######################################Train model########################################################################
rf <- randomForest(training_set, metadata_training_set[["class"]], ntree = 120 ,mtry = 8, importance = TRUE)
best_mtry = 8
best_ntree = 120
oob = data.frame()

# Loop over each value of mtry and store result in a data frame
#for (i in mtry) {
#
#  rf1 <- randomForest(training_set, metadata_training_set[["class"]], ntree = 120 ,mtry = i, importance = TRUE)
#
#  result = data.frame(mtry=i,
#                      OOB=rf1[["err.rate"]][nrow(rf1[["err.rate"]]),"OOB"])
#  oob = rbind(oob, result)
#}

# Loop over each value of ntree and store result in a data frame
#for (i in ntree) {
#
#  rf1 <- randomForest(training_set, metadata_training_set[["class"]], ntree = i ,mtry = 8, importance = TRUE)
#
#  result = data.frame(mtry=i,
#                      OOB=rf1[["err.rate"]][nrow(rf1[["err.rate"]]),"OOB"])
#  oob = rbind(oob, result)
#}
```

```
#plot(oob)

#feature importance plot
importance <- varImpPlot(rf, main = "variable importance")

##Testset prediction
pre_ran <- predict(rf,newdata=test_set)
obs_p_ran = data.frame(prob=pre_ran,obs=metadata_test_set$class)

#ROC plot with Testset
ran_roc <- roc(metadata_test_set$class,as.numeric(pre_ran))
ROCcurve <- plot(ran_roc, print.auc=TRUE, auc.polygon=TRUE, grid=c(0.3, 0.4),grid.col=c("grey", "grey"), max.auc.polygon=TRUE,auc.polygon.col="white", print.thres=TRUE,main='ROC curve, mtry=8, ntree=120')
lines(smooth(ROCcurve, method = "logcondens"), # method=c("binormal", "density", "fitdistr", "logcondens","logcondens.smooth")
      col = "#008600")


#bbplot
p <- ggplot(plotdata_importance, aes(x = feature, y = changed_MeanDecreaseAccuracy, color = group)) +
  geom_segment(aes(x = feature, y = changed_MeanDecreaseAccuracy, xend = feature, yend = changed_MeanDecreaseAccuracy)) +
  geom_point(aes(size=MeanDecreaseGini))+
  geom_col(position = "dodge", aes(fill = group),width = 0.05)+theme_set(theme_bw())
p <- p +geom_hline(aes(yintercept=0), colour="#BB0000", linetype="dashed")+ylab("MeanDecreaseAccuracy")+
  xlab("Features") + ggtitle("Feature importance") + theme(plot.title = element_text(hjust = 0.5))+theme(panel.grid.major=element_line(colour=NA))+coord_flip()
print(p)


#external validation prediction
set.seed(9811)
runif(14)
rf <- randomForest(training_set[,6:29], metadata_training_set[["class"]], ntree = 120 ,mtry = 8, importance = TRUE)
test_set2 <- read.csv("geo_test.csv",header = T,row.names = 1)
pre_ran <- predict(rf,newdata=log2(test_set2[,6:29]+0.1))
obs_p_ran = data.frame(prob=pre_ran,obs=test_set2$y)
ran_roc <- roc(test_set2$y,as.numeric(pre_ran))
ROCcurve <- plot(ran_roc, print.auc=TRUE, auc.polygon=TRUE, grid=c(0.3, 0.4),grid.col=c("white", "white"), max.auc.polygon=TRUE,auc.polygon.col="white", print.thres=TRUE,main='ROC curve, mtry=8, ntree=120')
```
