# Supplementary material for: The Diagnostic and Prognostic Values of HOXA Gene Family in Kidney Clear Cell Renal Cell Carcinoma
Source: J Oncol. 2022 Mar 16;2022:1762637. doi: 10.1155/2022/1762637 (PMC8942704; doi:10.1155/2022/1762637)
Supplement: Supplementary Materials — Supplementary Figure 1. Flow chart of data collection and subsequent model establishment in our study; Supplementary Figure 2. The TNM stage and clinical grade measured by the time-dependent receiver-operating characteristic curves at 1, 3, and 5 years. Supplementary Figure 3. The Kaplan–Meier survival curves of evaluating single prognostic roles of the prognosis-related HOXA genes. Supplementary Figure 4. A more detailed machine learning model construction process for early screening classification. Supplementary Figure 5. Custom scripts for prognostic model and machine learning model construction in this study; Table S1: differentially expressed genes between KIRC samples and paired normal samples, including the differential expression HOXA genes; Table S2: the training set, the test set, and the external validation set for machine learning model; Table S3: small molecule drugs by querying the CMAP and DGIdb databases; and Table S4: a detailed summary of the different roles of HOXA family members in KIRC. [file 1762637.f1.zip › 1762637.f1/Supplementary Table S1 (2).pdf]

Gene symbol	logFC	AveExpr	t	P.Value	adj.P.Val	B
AQP2	-9.446940693	3.36527669	-43.97941052	4.8948E-192	3.4254E-188	428.9365983
LINC02437	-3.448514347	2.240454136	-42.16840981	1.3408E-183	4.6914E-180	409.5279165
PRR35	-3.187961167	2.216687137	-41.7085582	1.9873E-181	4.6357E-178	404.5400159
UMOD	-10.7491672	3.755398766	-40.95278746	7.7825E-178	1.3615E-174	396.3276386
DUSP9	-5.524154151	2.919771048	-40.91351557	1.1986E-177	1.6775E-174	395.85308
AP005432.2	-3.839841576	2.513996669	-40.00760037	2.6789E-173	3.1245E-170	385.8740727
SLC9A4	-4.205586247	2.438442765	-39.76720756	3.8861E-172	3.8849E-169	383.2060681
ACPP	-3.791645153	2.742045754	-39.36745435	3.3724E-170	2.95E-167	378.751117
SLC12A1	-7.561834939	3.267201746	-39.16302036	3.3309E-169	2.5899E-166	376.4813882
KCNJ10	-4.779946195	2.889066048	-38.7406048	3.8438E-167	2.6899E-164	371.7243447
KNG1	-7.992584518	3.303109857	-38.70552049	5.7078E-167	3.6312E-164	371.3522443
TMEM207	-3.555243638	2.255922998	-38.47743944	7.4871E-166	4.3662E-163	368.7734999
FXYD4	-7.74743337	3.276204902	-37.86528797	7.7281E-163	4.1601E-160	361.8595416
MFSD4A	-4.404039853	4.461081518	-37.30482969	4.6188E-160	2.3087E-157	355.4565943
MUC15	-4.967415121	2.570877073	-36.64992792	8.5001E-157	3.9656E-154	347.9682853
HS6ST2	-3.843684937	2.447358043	-36.19861105	1.5564E-154	6.8074E-152	342.7748384
PVT1	2.385694176	4.131655483	35.99702065	1.6074E-153	6.617E-151	340.449932
ESRRB	-3.656379641	2.521948322	-35.94064026	3.091E-153	1.2017E-150	339.7927319
CA9	6.898889294	8.480081944	35.32662243	3.9158E-150	1.4422E-147	332.6743019
HRG	-3.91854546	2.3846855	-34.68249005	7.3715E-147	2.5793E-144	325.1412638
ATP12A	-2.69991143	2.151979172	-34.36344955	3.1399E-145	1.0463E-142	321.3939073
ELF5	-3.350093705	2.263251291	-34.30163777	6.5035E-145	2.0687E-142	320.6707574
AC073172.1	-2.771735604	2.177341739	-34.11188154	6.0964E-144	1.8549E-141	318.4350118
TMEM238L	-2.855185184	2.223114462	-33.86315234	1.1524E-142	3.3602E-140	315.5028427
GPC5	-2.626136049	2.25211251	-33.78606755	2.8694E-142	8.0321E-140	314.5915772
KCNJ1	-6.478535281	3.298157269	-33.44521487	1.6328E-140	4.3948E-138	310.5618812
SFRP1	-5.769075649	4.030566481	-32.62844783	2.753E-136	7.1353E-134	300.8476676
SPAG4	3.586078428	6.53086515	32.51116121	1.1199E-135	2.7988E-133	299.446093
IRX2	-3.319055482	2.643977136	-31.99398835	5.5339E-133	1.3354E-130	293.2587769
SERPINA5	-5.751105062	3.94426048	-31.77448552	7.7575E-132	1.8096E-129	290.6232416
AL031726.1	-2.26107276	2.200304015	-31.53282365	1.427E-130	3.2213E-128	287.7169453
CLDN16	-4.702385217	2.941808888	-31.46637241	3.1812E-130	6.9569E-128	286.9172211
NELL1	-3.551365958	2.380655655	-31.42763581	5.0773E-130	1.0767E-127	286.4510864
LINC02121	-3.363862695	2.227683712	-31.32874692	1.6758E-129	3.4492E-127	285.2593909
EGF	-5.085599546	3.720726558	-30.94431144	1.7533E-127	3.5056E-125	280.6184441
GP2	-2.256931855	2.087682712	-30.94153581	1.8133E-127	3.5248E-125	280.5849364
LINC00982	-3.826188584	2.582084345	-30.8803865	3.8039E-127	7.1945E-125	279.8454317
LINC00864	-2.436439339	2.193395983	-30.79920272	1.0177E-126	1.8742E-124	278.8635956
LINC01762	-2.472987778	2.398295743	-30.48251869	4.7552E-125	8.5326E-123	275.0273344
TFAP2B	-3.341208798	2.331963422	-30.29518812	4.6393E-124	8.1165E-122	272.7531741
CASR	-4.587223571	2.933262362	-29.9556416	2.9019E-122	4.9531E-120	268.62681
RANBP3L	-3.991596873	2.835250159	-29.56485688	3.4255E-120	5.7075E-118	263.8649286
AC016526.1	-1.940124969	2.034107224	-29.51701544	6.1479E-120	1.0005E-117	263.2821615
RALYL	-2.6075732	2.191560645	-29.49317433	8.2287E-120	1.3087E-117	262.9908765
SCNN1G	-5.88971901	3.158468194	-29.48546661	9.0421E-120	1.4061E-117	262.8958835
PTGER1	-3.304967427	2.521035606	-29.47267366	1.0573E-119	1.6085E-117	262.7389544
CLCNKA	-5.412387947	3.225141945	-29.42971563	1.7882E-119	2.6625E-117	262.2163264
AC087045.3	-2.593673075	2.19972104	-29.41889563	2.0413E-119	2.976E-117	262.0842311
AC007993.2	-2.097749005	2.108767605	-28.99199498	3.8092E-117	5.4402E-115	256.866773
GABRD	3.419155009	5.118641805	28.97303669	4.8063E-117	6.7269E-115	256.5466989
CLDN8	-6.24793398	3.003804883	-28.70436755	1.2998E-115	1.7836E-113	253.340519
NHLRC4	-2.462088046	3.101736628	-28.65280016	2.449E-115	3.2958E-113	252.7111038
AC092078.2	-2.027840022	2.059223953	-28.42410064	4.0728E-114	5.3776E-112	249.9063895
CLCNKB	-6.173761635	3.581874719	-28.28081378	2.3742E-113	3.0768E-111	248.1442265
PRR15	-3.453123872	3.256808762	-28.19407459	6.9064E-113	8.7875E-111	247.0812581
SCNN1B	-4.597495801	3.191126519	-28.16130638	1.0339E-112	1.292E-110	246.6791519
CA10	-2.657448327	2.207085606	-28.04291623	4.4445E-112	5.4566E-110	245.2221091

NRK	-3.027661045	2.378676376	-27.92414838	1.9209E-111	2.3177E-109	243.7590812
AC124017.1	-2.405049986	2.113484435	-27.91873731	2.0535E-111	2.4356E-109	243.6960319
GGT6	-4.937232897	3.009709558	-27.85942771	4.2664E-111	4.9761E-109	242.9657961
CLDN19	-4.620406346	3.049602492	-27.78528258	1.0646E-110	1.2213E-108	242.05362
WNK4	-3.550772014	3.565512883	-27.76119665	1.4329E-110	1.6174E-108	241.7592261
CALB1	-4.674928304	2.643393825	-27.64567408	5.9605E-110	6.6209E-108	240.3323428
LINC01983	-2.024621943	2.05434197	-27.64351822	6.1212E-110	6.6932E-108	240.3083728
MCCD1	-4.341263175	2.82726145	-27.63422942	6.8648E-110	7.3907E-108	240.1919588
ATP1A1	-2.775609151	9.523119047	-27.27030317	6.148E-108	6.5187E-106	235.6980907
ADH1C	-3.853377311	2.784770404	-27.26141046	6.8622E-108	7.1674E-106	235.5951031
IRX1	-3.318925828	2.646228392	-27.23239205	9.8228E-108	9.9907E-106	235.2358178
TREM2	3.735068717	6.477220369	27.23216216	9.8508E-108	9.9907E-106	235.2331349
SPTBN2	-4.529027428	3.408390424	-27.2287376	1.0277E-107	1.0274E-105	235.1971589
LINC00887	3.619374042	5.268361543	27.2268298	1.0522E-107	1.0371E-105	235.0571853
FAM3B	-3.164018839	2.618970482	-27.08727437	5.9085E-107	5.7427E-105	233.4452288
GPC3	-4.180629538	4.409485715	-27.06689673	7.602E-107	7.2875E-105	233.2016646
FABP6	4.059784789	5.672620502	27.04942794	9.4355E-107	8.9229E-105	232.8676729
TMEM52B	-4.833194968	3.528371236	-27.01815299	1.3892E-106	1.2962E-104	232.5981792
TYRP1	-2.843662742	2.265698833	-26.79742672	2.133E-105	1.964E-103	229.8677382
RASL11B	-3.749117914	3.107085333	-26.65662945	1.2192E-104	1.108E-102	228.1304243
DGCR5	3.484740419	5.441924657	26.56518218	3.784E-104	3.3949E-102	226.9246467
NAT8L	-3.542916975	2.608615213	-26.54661151	4.7627E-104	4.2189E-102	226.7657122
TMEM45B	-3.525888262	3.552275106	-26.53376831	5.5841E-104	4.8847E-102	226.6153478
HSPA2	-3.426182438	4.762909951	-26.40411026	2.7837E-103	2.405E-101	225.0144955
CDKN2A	1.82929342	3.482215215	26.36535168	4.5001E-103	3.8405E-101	224.3871733
LINC01543	-2.639573879	2.197814921	-26.32689975	7.2476E-103	6.1107E-101	224.0528549
AP000696.1	-2.161530476	2.155782381	-26.29705489	1.0492E-102	8.7407E-101	223.6861718
AC104237.3	-3.160491801	2.440874325	-26.153178	6.2444E-102	5.141E-100	221.8993356
LINC01571	-2.150573129	2.07685633	-26.06391628	1.8889E-101	1.537E-99	220.8015406
NDUFA4L2	6.447529281	10.55943747	26.02568949	3.0345E-101	2.4409E-99	220.3312285
SIM2	-2.521864695	2.555643064	-25.92162556	1.1032E-100	8.7298E-99	219.0371173
PIK3C2G	-2.552257036	2.19940277	-25.92111547	1.1102E-100	8.7298E-99	219.0321161
EHF	-4.142749061	2.800511496	-25.89627537	1.5109E-100	1.17483E-98	218.7224044
NOL3	3.171392784	7.416275723	25.85926026	2.3915E-100	1.83911E-98	218.2745211
AL049838.1	-2.783263505	3.280073783	-25.72288394	1.2988E-99	9.87953E-98	216.5782192
TMPRSS2	-4.464599179	2.981888695	-25.48119168	2.60793E-98	1.9624E-96	213.5845826
AL139280.1	-2.982526189	2.289233442	-25.43254466	4.77043E-98	3.55144E-96	212.9773938
STC2	3.883899337	6.511803789	25.21778841	6.8626E-97	5.05521E-95	210.3123986
CDCA2	2.920330983	4.72157482	25.18722204	1.00303E-96	7.3117E-95	209.8146538
ATP6V1B1	-5.136323581	3.596869569	-25.14120937	1.776E-96	1.28128E-94	209.3742972
SLC4A1	-5.453197428	3.157166195	-25.12439937	2.18823E-96	1.56258E-94	209.1627657
TMEM213	-6.135955965	3.191912178	-25.12177219	2.26079E-96	1.59809E-94	209.1258923
S100A2	-3.655271006	4.446955458	-25.09096487	3.31435E-96	2.31938E-94	208.7576911
OVCH2	-2.682209225	2.353867735	-25.04653088	5.75475E-96	3.9873E-94	208.1965833
AC007906.2	-3.561343423	3.692186754	-24.99485662	1.09322E-95	7.50035E-94	207.5648863
APOC1	4.607815642	7.846132359	24.96691302	1.54672E-95	1.05087E-93	207.2235247
ARMH4	-2.61597732	3.086142138	-24.96266481	1.63051E-95	1.09714E-93	207.1586574
GATA3	-3.419641227	4.192423932	-24.94035382	2.15105E-95	1.43362E-93	206.8919903
PROM2	-4.418917558	3.465002132	-24.69080684	4.77034E-94	3.14932E-92	203.7955041
SMIM5	-2.995606118	2.90075158	-24.63284601	9.79843E-94	6.40836E-92	203.0689206
BSND	-4.317520113	2.58222308	-24.6052045	1.38114E-93	8.94928E-92	202.7246197
ERVE-1	-2.492516332	2.198500802	-24.52645517	3.67248E-93	2.3578E-91	201.7540648
LINC01055	-2.875526129	2.285546042	-24.51724211	4.11763E-93	2.61956E-91	201.6359842
SOST	-2.8252719	2.239344302	-24.48880072	5.8619E-93	3.69564E-91	201.2842144
TMEM178A	-2.808694938	3.535399792	-24.38262872	2.19095E-92	1.36895E-90	199.9747012
PLPPR1	-3.419933353	2.550301532	-24.37319647	2.4632E-92	1.52544E-90	199.8474534
TRPV6	-1.559324912	2.048316629	-24.34391751	3.54322E-92	2.17504E-90	199.4852532
ERVMER34-1	-2.643709837	2.387756721	-24.30386198	5.82649E-92	3.54554E-90	198.992996

HSF4	3.815262237	5.779851341	24.27695686	8.13759E-92	4.90921E-90	198.5748139
AC090709.1	-1.796256056	2.003186341	-24.21846337	1.68236E-91	1.00625E-89	197.9355576
CXCR4	2.968869636	8.750359734	23.91609313	7.18038E-90	4.25833E-88	194.1965401
TNNC1	-2.680625578	2.757217446	-23.90293397	8.45437E-90	4.97174E-88	194.0255817
MTURN	-2.926171838	5.376400648	-23.86016952	1.43745E-89	8.38274E-88	193.5066809
PRRG2	-2.611665107	3.259587749	-23.78532992	3.63884E-89	2.10451E-87	192.5726886
HILPDA	4.645211417	8.514553374	23.77494742	4.13922E-89	2.37429E-87	192.4558702
CD70	4.649865184	6.361772402	23.74316111	6.14078E-89	3.49375E-87	191.9266143
SLC52A3	-2.666232116	3.250772835	-23.71871639	8.31679E-89	4.69362E-87	191.7476998
TFCP2L1	-4.639530551	3.771065236	-23.70991542	9.27647E-89	5.19334E-87	191.6446455
GSTM3	-3.213103059	5.485998661	-23.60791691	3.28838E-88	1.82636E-86	190.3811435
LILRB1	1.880791955	4.154238746	23.33972205	9.15279E-87	5.0434E-85	186.970393
AC010655.2	2.223329154	3.850001202	23.29320224	1.62941E-86	8.90831E-85	186.3052005
LINC00645	-3.304595285	2.533835391	-23.25958488	2.47184E-86	1.34092E-84	186.0572398
ODF3B	2.897999263	5.665002876	23.25239905	2.70212E-86	1.45457E-84	185.9649057
TMEM74B	1.964386491	3.970526231	23.24645917	2.90858E-86	1.55376E-84	185.7770023
EPN3	-2.767902949	2.479310523	-23.18046077	6.59089E-86	3.49417E-84	185.0824728
DDN	-2.767419001	2.296117753	-23.16878842	7.61673E-86	4.00766E-84	184.9390543
MPP7	-2.570930968	3.669571168	-23.14461196	1.02775E-85	5.32756E-84	184.6481077
F11	-3.196369349	2.742512592	-23.14487114	1.02446E-85	5.32756E-84	184.6404405
DGCR9	2.225890879	4.147768516	23.12596943	1.29484E-85	6.66273E-84	184.2789503
TNFAIP6	4.544069645	6.498547221	23.12064291	1.38319E-85	7.06538E-84	184.2561045
RHCG	-5.802783091	3.237990076	-23.09755012	1.84142E-85	9.33785E-84	184.0579847
TCF21	-2.814291707	3.361844698	-23.09423045	1.91874E-85	9.65995E-84	184.0221104
AC104237.2	-2.393091534	2.265278087	-23.09337232	1.93925E-85	9.69347E-84	184.0107114
AC107057.1	-2.387555139	2.151369794	-23.0683134	2.64529E-85	1.31289E-83	183.7016419
ADGRF1	-3.665405811	2.611744715	-23.05612779	3.07637E-85	1.51609E-83	183.5416789
ANGPTL4	5.455187428	9.951084388	23.04218938	3.65622E-85	1.78925E-83	183.3822865
SCARB1	3.609440312	7.05561149	23.02986539	4.2593E-85	2.0699E-83	183.2409738
AL035661.1	-4.74849557	3.068268451	-23.00483815	5.80739E-85	2.80277E-83	182.9170198
ATP6V0A4	-5.723469789	3.222657114	-22.99592235	6.48551E-85	3.1086E-83	182.8019001
TTC21B-AS1	3.746930852	5.117662502	22.8568098	3.63191E-84	1.72899E-82	180.8936288
SLC16A3	3.020939069	7.180521976	22.76080407	1.19201E-83	5.59844E-82	179.9144674
PARVG	1.733465309	3.950052943	22.75980718	1.20681E-83	5.63016E-82	179.7875437
SLC4A11	-2.740866036	2.973788662	-22.73822027	1.57641E-83	7.30576E-82	179.6189434
AP000757.1	-2.832462549	3.254266587	-22.71315504	2.14973E-83	9.89724E-82	179.312346
ITGAX	2.3262601	4.679743604	22.64495209	4.999E-83	2.28647E-81	178.3977218
REEP6	-2.339356014	3.480069633	-22.63146839	5.90648E-83	2.684E-81	178.3068921
DDB2	1.857102051	5.982553594	22.58188419	1.0907E-82	4.92432E-81	177.7070873
PRDM16	-2.395296279	2.664001623	-22.54661371	1.68714E-82	7.52013E-81	177.2555061
AC019069.1	1.752340963	3.750710573	22.54859133	1.64638E-82	7.38548E-81	177.1396517
GATA3-AS1	-1.870300278	2.084443196	-22.51892534	2.37604E-82	1.04576E-80	176.9160285
SIGLEC8	2.174913439	3.843450374	22.53252826	2.00817E-82	8.8944E-81	176.9021049
COL23A1	4.87177387	7.556864548	22.51400142	2.5252E-82	1.10446E-80	176.8525975
FAM222A	-2.166795391	3.130998843	-22.4748254	4.09884E-82	1.7816E-80	176.3731696
EMX1	-2.416704342	4.30123067	-22.45909361	4.97883E-82	2.15073E-80	176.1862078
FAM167A	-2.782151012	2.964325417	-22.41696922	8.38052E-82	3.59797E-80	175.654071
BMPR1B-DT	-1.615695997	2.057644504	-22.40933853	9.2094E-82	3.92972E-80	175.5575151
LHX1-DT	-3.121079057	2.474260088	-22.40204221	1.00785E-81	4.27451E-80	175.4650076
C1orf226	-2.256431417	3.201148069	-22.36858675	1.52391E-81	6.4243E-80	175.0623305
HLA-F	2.311120399	7.484566835	22.35488139	1.80515E-81	7.56432E-80	174.8978597
AL031123.1	-3.234971446	2.875512155	-22.32912873	2.48146E-81	1.03364E-79	174.5690639
DOK3	1.72849503	4.215985639	22.30371459	3.39679E-81	1.40655E-79	174.1989496
NPHS2	-4.705848704	2.688806126	-22.28579417	4.23851E-81	1.72448E-79	174.0367428
SLC15A2	-2.448751603	3.118604912	-22.2831094	4.38143E-81	1.77233E-79	174.0067591
TMEM30B	-3.328234554	3.669106551	-22.18942737	1.39345E-80	5.60421E-79	172.8603968
CD68	1.561699525	3.594973534	22.12486103	3.0922E-80	1.23653E-78	171.916919
LINC01976	-1.74379027	2.061138061	-22.11295578	3.58168E-80	1.42412E-78	171.908832

LINC00462	3.753916193	5.298312336	22.09630775	4.39869E-80	1.7391E-78	171.5235407
TSPAN8	-4.046823899	3.313317811	-22.01974488	1.1314E-79	4.44807E-78	170.7679318
TYMP	2.904378668	7.169319721	21.99411024	1.55223E-79	6.06844E-78	170.4616865
AC079760.2	2.608178777	4.264744189	21.96452248	2.23589E-79	8.64461E-78	169.9005851
CD300LF	1.807809971	3.970534349	21.92872173	3.4769E-79	1.33689E-77	169.5276871
LINC02343	-1.916094641	2.10305436	-21.88993685	5.60886E-79	2.14485E-77	169.1679783
FGF1	-3.113644762	3.739855795	-21.88718697	5.80227E-79	2.20676E-77	169.1402694
SCNN1A	-4.321131112	4.892762985	-21.87286793	6.92246E-79	2.61856E-77	168.9604817
SLC26A7	-4.08793968	2.89369541	-21.85419686	8.71393E-79	3.2785E-77	168.7236633
KLRG2	-2.521705826	2.28063323	-21.80714141	1.55623E-78	5.8238E-77	168.1468997
RNF150	-2.633101179	3.36268735	-21.77089152	2.4325E-78	9.00668E-77	167.7038689
LINC02348	3.175189532	4.738727364	21.77564637	2.29409E-78	8.53939E-77	167.5681203
FOXCUT	-2.062473384	2.442558734	-21.75312557	3.02765E-78	1.11513E-76	167.4875196
HOXB9	-3.631063389	3.82808295	-21.67443193	7.98037E-78	2.92391E-76	166.5280598
SDS	2.43224658	4.3940597	21.67045871	8.3805E-78	3.05452E-76	166.3264445
CLUL1	-1.648131614	2.361414457	-21.64653242	1.12514E-77	4.07964E-76	166.1785507
NR0B2	-3.90004319	2.603794045	-21.63051614	1.37036E-77	4.91783E-76	165.9689266
ATP6V0D2	-5.081078138	3.381815428	-21.61028494	1.75787E-77	6.25624E-76	165.7318219
LINC00379	-2.900455979	2.611616895	-21.61013173	1.76119E-77	6.25624E-76	165.7203948
COL4A6	-2.159260646	2.224472179	-21.59707994	2.0681E-77	7.23627E-76	165.5700504
RASAL3	1.923849331	4.493757674	21.59920862	2.01462E-77	7.08458E-76	165.5429939
COL5A3	2.470715356	4.897415829	21.59982433	1.99941E-77	7.0666E-76	165.5361304
INPP5J	-3.476770657	3.096072048	-21.56179459	3.19266E-77	1.10605E-75	165.1323665
SUSD4	-3.067354675	3.057471713	-21.51620902	5.59399E-77	1.92841E-75	164.5714155
TYROBP	2.778604186	8.72104923	21.51093106	5.96922E-77	2.04768E-75	164.5164631
SLC2A12	-2.288489631	2.522255646	-21.51010105	6.03048E-77	2.0586E-75	164.5000563
CHL1	-2.824735874	2.821903915	-21.49661659	7.11844E-77	2.41819E-75	164.3287686
ESRRG	-2.833616759	3.43780799	-21.49251871	7.48641E-77	2.53091E-75	164.2864637
SLC13A2	-3.63184336	2.649963246	-21.48370646	8.34341E-77	2.80708E-75	164.1667841
ABHD17C	-1.831840568	4.292197143	-21.46167438	1.09399E-76	3.66304E-75	163.9128876
LINC01020	-1.673250065	2.039508365	-21.43822724	1.45956E-76	4.86382E-75	163.6137812
RNU1-106P	1.512937987	3.250831071	21.38556279	2.78859E-76	9.20498E-75	162.7733309
AC138207.5	1.943263604	4.782319942	21.36240902	3.70651E-76	1.21775E-74	162.6709408
EPB41L4B	-2.456469806	2.442098712	-21.35424243	4.09778E-76	1.33378E-74	162.5865739
PIK3R5	1.747938981	3.980919054	21.35985839	3.82453E-76	1.25066E-74	162.547935
ATP6V1C2	-2.504634468	3.276117954	-21.34372279	4.66323E-76	1.5108E-74	162.4597241
CEL	-2.21371992	2.488322746	-21.33769315	5.02184E-76	1.61948E-74	162.3870339
SEMA3B-AS1	-2.495537976	2.984899844	-21.32563738	5.82359E-76	1.86942E-74	162.2359177
FAM81A	-1.78598402	2.988585289	-21.31848251	6.35866E-76	2.03187E-74	162.1563559
EGLN3	4.074554866	8.207992561	21.31007963	7.05012E-76	2.23243E-74	162.0601688
CHGB	-2.928528379	2.675154832	-21.31152025	6.92645E-76	2.20324E-74	162.0571908
F11-AS1	-1.814106097	2.202759507	-21.30509429	7.49538E-76	2.3579E-74	161.9881767
NNMT	4.475335081	9.752247505	21.30489494	7.51375E-76	2.3579E-74	161.9867827
RAB25	-4.411428335	3.198064127	-21.29459429	8.52723E-76	2.664E-74	161.8602642
PDE1A	-2.928197996	4.60716802	-21.22266963	2.06251E-75	6.41485E-74	160.9895022
SYT7	-2.690668082	2.730600607	-21.21102479	2.37947E-75	7.36794E-74	160.8283708
FGF9	-3.499476779	2.56141796	-21.18803445	3.15531E-75	9.72724E-74	160.5411874
FCGR3A	3.404016148	7.685196958	21.18391325	3.31901E-75	1.0187E-73	160.5145832
HLA-B	1.963595733	12.49038654	21.1624601	4.3187E-75	1.31975E-73	160.2218325
SCD	3.292880607	8.49880128	21.1544859	4.76268E-75	1.4491E-73	160.1458444
NPHS1	-3.052605787	2.485608319	-21.15046613	5.00349E-75	1.51578E-73	160.0828607
LAPTM5	2.708302295	8.838299134	21.13798834	5.8313E-75	1.75894E-73	159.9467903
SLC12A3	-5.170788051	2.941340036	-21.1359794	5.97682E-75	1.7951E-73	159.9125704
MTCP1	1.912031269	4.138284748	21.11117038	8.10314E-75	2.42332E-73	159.5024505
SLC4A9	-2.823038915	2.401696651	-21.09817163	9.50389E-75	2.83014E-73	159.445785
NPTX2	4.820596786	6.582537057	21.09681955	9.66282E-75	2.86527E-73	159.2997157
DANCR	-1.71221868	5.465004936	-21.08238044	1.1535E-74	3.40598E-73	159.2704153
ACSF2	-2.428598469	4.475745399	-21.04168869	1.89988E-74	5.58627E-73	158.7722571

KLK6	-2.698739982	2.326217322	-21.01700633	2.57123E-74	7.52866E-73	158.4548458
ENPP6	-2.391137756	2.472467949	-20.9693798	4.60946E-74	1.33846E-72	157.8757725
KLHL14	-2.414471959	2.697373229	-20.96578834	4.81685E-74	1.39291E-72	157.8305111
LINC02188	2.33602353	4.371128642	20.97524936	4.28956E-74	1.25076E-72	157.8097609
ARL4D	-3.003702888	4.83105637	-20.93895037	6.69225E-74	1.92726E-72	157.5181075
SLC7A8	-3.255864893	4.919561013	-20.92455988	7.98241E-74	2.28938E-72	157.3406905
VIM	2.666943908	11.58150175	20.91959132	8.48334E-74	2.42312E-72	157.2629614
C16orf89	-3.232929236	3.367692759	-20.90772573	9.81039E-74	2.79078E-72	157.1256277
PCED1B-AS1	1.772880168	4.154098946	20.90547964	1.0084E-73	2.857E-72	157.0110176
SLC16A5	-2.660416322	4.349640905	-20.86715279	1.6124E-73	4.53155E-72	156.6395508
TACSTD2	-4.420068825	5.559364919	-20.86828294	1.59024E-73	4.48729E-72	156.6381302
SAP30	2.284982999	6.257294194	20.85670144	1.83251E-73	5.12955E-72	156.5186271
ADM	2.813278523	8.467255803	20.85583452	1.85206E-73	5.16363E-72	156.4950024
EFHD1	-2.807174471	6.735509763	-20.82499585	2.70152E-73	7.50207E-72	156.1112229
TNFSF9	2.4843983	4.768142786	20.79883495	3.721E-73	1.02923E-71	155.7027565
NETO2	2.715296149	5.572133644	20.77654014	4.88808E-73	1.34672E-71	155.5102985
MECOM	-2.608539134	5.026423061	-20.75274758	6.53962E-73	1.79468E-71	155.244331
LIPH	-3.046649891	2.985497038	-20.73549842	8.07579E-73	2.199E-71	155.0156762
AC114803.1	2.096330125	3.658540303	20.75031979	6.73675E-73	1.84155E-71	154.9888931
AC112243.1	-1.700300574	1.977239366	-20.72946811	8.69394E-73	2.35815E-71	154.9388268
PSMB8	1.602114088	8.629940994	20.72038361	9.71558E-73	2.62508E-71	154.8479804
HK2	3.133879751	6.184938546	20.62897434	2.97015E-72	7.96363E-71	153.7275437
FOLR3	-2.441681355	2.466952956	-20.59884617	4.29191E-72	1.14637E-70	153.3525652
FLRT1	-1.798373319	2.218048824	-20.57859894	5.49627E-72	1.45693E-70	153.1099916
AC026369.2	2.422310996	4.729719088	20.58078861	5.35121E-72	1.42387E-70	153.0466402
COBLL1	-2.285740032	4.998384661	-20.44523558	2.79974E-71	7.39342E-70	151.4967438
AQP6	-4.100394273	2.628039224	-20.43132141	3.3177E-71	8.72829E-70	151.3052432
TMSB10	2.144667523	13.60238506	20.42973162	3.38267E-71	8.8328E-70	151.2695301
P2RX7	1.896351298	4.42921548	20.43052652	3.35003E-71	8.78033E-70	151.243052
KLK7	-2.155374241	2.150033554	-20.39720277	5.02995E-71	1.30369E-69	150.9019
SLC6A3	4.700053367	6.297885913	20.40964635	4.32172E-71	1.12429E-69	150.8765723
FATE1	1.682408358	3.577272423	20.38856997	5.58834E-71	1.44307E-69	150.6163626
HSD11B2	-3.859021845	6.813429457	-20.34380349	9.64507E-71	2.48148E-69	150.2399834
FAM78A	1.654118626	4.222039682	20.3344947	1.08039E-70	2.76944E-69	150.0762747
SUCLG1	-1.600457037	7.12828002	-20.32649734	1.19099E-70	3.04181E-69	150.0442329
AP1M2	-2.915277098	5.254769693	-20.3257934	1.20125E-70	3.05686E-69	150.0420128
PLCL1	-2.512439193	4.402781408	-20.32026794	1.28493E-70	3.25794E-69	149.975547
PLA2G4F	-3.205528788	2.472606129	-20.31379792	1.39033E-70	3.51247E-69	149.8741307
ERBB4	-2.73918258	2.495250119	-20.30659571	1.51786E-70	3.80717E-69	149.7910844
GNLY	2.100293441	4.404704573	20.2960412	1.72616E-70	4.29881E-69	149.574577
AC007326.1	2.512122507	4.047096475	20.29643104	1.71798E-70	4.29372E-69	149.4590214
L1CAM	-3.979095523	3.315610458	-20.27701112	2.17652E-70	5.40117E-69	149.4425797
ESM1	3.654962526	8.55269759	20.25014028	3.01923E-70	7.46593E-69	149.1173876
KCNK13	-1.91917498	3.257895175	-20.24266899	3.30681E-70	8.14827E-69	149.0259767
TMIGD3	2.015633798	4.383761677	20.23044547	3.83751E-70	9.42276E-69	148.7853842
MYO1F	1.949405613	4.690113836	20.21270776	4.7625E-70	1.16531E-68	148.6247229
SIGLEC10	2.147627126	4.558636975	20.18672	6.53451E-70	1.59333E-68	148.2619776
LRRC2	-2.287245023	2.556072893	-20.16484257	8.52777E-70	2.07213E-68	148.075824
TRPM2	1.624352874	3.883581297	20.16104043	8.93156E-70	2.16274E-68	147.9143046
IL2RB	2.320403097	4.91543705	20.12578435	1.3715E-69	3.30958E-68	147.5547622
TNFRSF4	2.260098814	4.744938418	20.11737798	1.51915E-69	3.65326E-68	147.434043
COL26A1	-2.250454153	2.284845292	-20.07865978	2.43258E-69	5.79021E-68	147.0320164
VTCN1	-3.958083204	2.950571286	-20.05183179	3.37052E-69	7.99557E-68	146.7019937
CAV2	1.932330507	7.017060746	20.04817017	3.52391E-69	8.33119E-68	146.6682712
TJP3	-2.379934826	2.843124824	-20.00423731	6.00982E-69	1.4113E-67	146.1270955
SFTA1P	1.706864259	3.357807454	19.98251442	7.82449E-69	1.8313E-67	145.6461924
FCER1G	2.551668883	7.887942693	19.95704296	1.06609E-68	2.48682E-67	145.5624485
ST8SIA4	2.690379298	5.239735414	19.95645935	1.07367E-68	2.49619E-67	145.5013888

TNNI1	-1.684373959	2.224190535	-19.948431	1.1836E-68	2.74265E-67	145.4536667
AL096799.1	2.753468503	4.473196054	19.94533462	1.22894E-68	2.83832E-67	145.220477
DCXR	-1.953605181	6.62204027	-19.89055331	2.3894E-68	5.48229E-67	144.7580835
ENO2	3.134336928	7.624263945	19.87512303	2.88133E-68	6.58939E-67	144.5769557
WNT9B	-1.865975785	2.321641506	-19.86609489	3.21482E-68	7.30433E-67	144.4599731
DGCR10	1.685705808	3.545174348	19.87154991	3.00897E-68	6.85889E-67	144.3345977
PROX1	-2.447135821	2.570131302	-19.85124744	3.8492E-68	8.68925E-67	144.2732445
RASD2	2.143529275	4.551106157	19.84871648	3.96919E-68	8.93131E-67	144.1666938
LINC01094	1.570457712	3.682285991	19.8535418	3.74356E-68	8.47814E-67	144.1578844
FER1L4	2.037369678	3.667096714	19.83861781	4.48632E-68	1.00626E-66	143.9081998
INHBB	3.439009322	6.502837156	19.81563211	5.92834E-68	1.31703E-66	143.8549061
GRIK3	2.25177905	3.914178398	19.82918383	5.03006E-68	1.12461E-66	143.8006902
CD300A	2.444485417	5.392745259	19.80943214	6.39112E-68	1.41535E-66	143.7641792
PAPPA	-2.263654664	2.839064168	-19.76962567	1.03541E-67	2.28574E-66	143.2888598
VEGFA	3.299601138	8.953039204	19.76163845	1.14063E-67	2.5101E-66	143.1981598
GABRA2	-2.058394686	2.115795396	-19.74654323	1.36953E-67	3.00438E-66	143.0129435
AC097534.2	1.920954769	4.396297637	19.74108614	1.46313E-67	3.19968E-66	142.8692099
ATP6V1G3	-4.468032087	2.748562801	-19.7285887	1.70226E-67	3.71104E-66	142.7879907
MAN1C1	-2.199742858	3.736936618	-19.72004271	1.8879E-67	4.10296E-66	142.6956513
AVPR2	-2.51338793	3.058718811	-19.70776476	2.19056E-67	4.74598E-66	142.539544
SSC4D	-1.64148682	2.958481451	-19.70208765	2.34644E-67	5.06803E-66	142.4828954
GMPR	-2.420959955	4.580087383	-19.6840158	2.92035E-67	6.28819E-66	142.2679794
DNASE1	-2.021236002	3.40418661	-19.68175982	3.00121E-67	6.44247E-66	142.2324478
AL161668.4	-1.569247428	2.143996985	-19.67450782	3.27659E-67	7.01211E-66	142.1353165
MYO3B	-2.65920201	2.551282032	-19.65499626	4.1494E-67	8.8529E-66	141.8987941
CD247	1.747902109	4.135755775	19.6535084	4.22479E-67	8.98635E-66	141.7967842
GRHL2	-2.706976528	2.424599127	-19.58750104	9.38857E-67	1.98493E-65	141.0844126
ITLN1	-1.792476677	2.27115033	-19.58506338	9.66944E-67	2.03816E-65	141.0644239
VGLL1	-2.287890363	2.138156714	-19.5782778	1.04963E-66	2.19919E-65	140.9811329
OSCAR	1.596532964	3.962893453	19.57196517	1.13288E-66	2.36654E-65	140.8024075
AC019080.1	-1.791767929	2.976549894	-19.5520115	1.44194E-66	3.00319E-65	140.6696803
AHNAK2	2.669639089	4.903407647	19.53470307	1.77747E-66	3.69102E-65	140.3487474
SOWAHA	-2.527333851	2.897474256	-19.5152788	2.24772E-66	4.65371E-65	140.216347
CRHBP	-2.950505771	3.167171978	-19.51334398	2.30089E-66	4.74973E-65	140.1941152
C3	4.318628063	9.405545985	19.49749075	2.7866E-66	5.71866E-65	140.0092686
LINP1	1.679762442	3.584889933	19.50140077	2.65804E-66	5.47086E-65	139.8678245
SIRLNT	-1.681764972	2.033122612	-19.47008476	3.88001E-66	7.89311E-65	139.6687979
FCGR1A	1.80516962	4.035888938	19.47524479	3.6456E-66	7.43788E-65	139.6157308
LZTS3	-2.269205256	4.518608743	-19.45898655	4.43644E-66	8.99891E-65	139.5533708
GMFG	1.80531094	7.017503391	19.43360864	6.02692E-66	1.21897E-64	139.2431939
MIR210HG	2.664757513	5.697759908	19.43000338	6.29499E-66	1.26952E-64	139.1950995
ERMP1	-2.255809415	5.926326183	-19.42624888	6.58682E-66	1.32456E-64	139.1576715
RHBDF2	1.737305171	5.535985051	19.40246811	8.77635E-66	1.7598E-64	138.8825836
HLA-A	1.702844821	12.11959768	19.39730854	9.3401E-66	1.86749E-64	138.781042
AC079310.1	-1.671106198	1.974768597	-19.39596111	9.49318E-66	1.89269E-64	138.7723387
LAIR1	2.097670432	4.872162195	19.37583307	1.21023E-65	2.40603E-64	138.5124903
CD40	1.801561865	6.943059217	19.33028799	2.09604E-65	4.14352E-64	138.000584
COLGALT1	1.695610372	7.336352625	19.32112251	2.34091E-65	4.61456E-64	137.8897509
PRKAR2B	-1.839984377	3.876087531	-19.31292536	2.58402E-65	5.0511E-64	137.7912663
MAL	-4.816579393	6.584669413	-19.31373493	2.55893E-65	5.01607E-64	137.7787456
LILRB4	2.026777155	4.326298427	19.3173782	2.44898E-65	4.81404E-64	137.7326183
AC104984.4	-2.388637223	2.493324374	-19.30648179	2.7927E-65	5.44381E-64	137.7056141
KLHL13	-1.94091748	3.920199815	-19.28609825	3.57028E-65	6.94022E-64	137.4688117
SHMT2	1.9492696	7.922910161	19.27515105	4.07365E-65	7.89678E-64	137.3384543
LY86	2.326465839	5.701492049	19.26054972	4.85705E-65	9.38941E-64	137.1715938
PSMB9	2.041107035	6.930241952	19.2542282	5.24132E-65	1.01043E-63	137.0885031
GCGR	-2.930055796	2.419838043	-19.25242676	5.35628E-65	1.02976E-63	137.0485451
FBXO2	-2.513602413	3.775168564	-19.23806712	6.36748E-65	1.22081E-63	136.8914518

AL021328.1	1.852872283	3.733980904	19.20547956	9.42662E-65	1.80239E-63	136.3084471
SLC9A2	-2.593847715	2.473419484	-19.18462439	1.21161E-64	2.31031E-63	136.2388466
GAL3ST4	2.148339864	4.806565647	19.18227166	1.2464E-64	2.3702E-63	136.1712147
IGSF6	2.122603304	4.999053451	19.17928113	1.29207E-64	2.45039E-63	136.1624948
TRPV2	1.792261273	5.413557504	19.17550571	1.35212E-64	2.55734E-63	136.1544913
FOXI1	-4.584232074	2.803721152	-19.16297284	1.57218E-64	2.95568E-63	135.9795923
CD1D	1.800040628	4.62318033	19.16280239	1.57541E-64	2.95568E-63	135.9529225
MYO1G	1.552534212	3.759161062	19.16523077	1.53005E-64	2.88606E-63	135.8787373
DMRT2	-3.904095129	2.661611238	-19.14387223	1.97827E-64	3.70158E-63	135.7440929
HOXB-AS3	-2.335713828	3.077859743	-19.13489941	2.20369E-64	4.11239E-63	135.646559
RGS19	1.597579515	5.545482156	19.13033044	2.32816E-64	4.33311E-63	135.613469
SEMA6D	-2.354796711	3.607988749	-19.12915324	2.36135E-64	4.38323E-63	135.5823351
MAPK4	-2.148083495	2.215160738	-19.12588228	2.45608E-64	4.547E-63	135.5415924
LAMA4	2.56175794	6.276846049	19.08904033	3.82466E-64	6.99299E-63	135.1193098
ANKRD2	-2.930504755	2.973481734	-19.08767646	3.88787E-64	7.08524E-63	135.0751047
PNCK	3.907833683	5.517907979	19.10388706	3.19956E-64	5.89224E-63	135.0745277
EPCAM	-3.236292244	6.898329711	-19.07918003	4.30584E-64	7.82656E-63	134.9719106
RGS1	3.119039027	6.927765564	19.07047268	4.78078E-64	8.66733E-63	134.8967342
LINC02048	1.524756736	3.106555892	19.08898391	3.82726E-64	6.99299E-63	134.8542721
LST1	2.009566183	5.461101442	19.05479887	5.77142E-64	1.04363E-62	134.7046377
SPI1	2.226005326	6.32829131	19.04675371	6.35704E-64	1.14656E-62	134.6102304
IDO1	2.988689832	5.510027841	19.03914593	6.96532E-64	1.25304E-62	134.4513341
DIO1	-3.294122254	2.927970964	-19.03421877	7.38995E-64	1.32602E-62	134.4335024
XPNPEP2	-3.876129897	3.299512481	-19.02631126	8.12614E-64	1.45439E-62	134.3496856
LGALS9	1.967793872	5.663562952	19.00973962	9.91521E-64	1.77007E-62	134.1687263
PCCB	-1.655712644	4.919549316	-19.0060012	1.03704E-63	1.84661E-62	134.1126308
TMEM61	-3.166650276	2.685467419	-18.9882689	1.28304E-63	2.27885E-62	133.8796318
GAL3ST3	-2.143963433	2.264945984	-18.98411261	1.34866E-63	2.38935E-62	133.843067
FREM1	-2.490246049	2.719833628	-18.96151796	1.76872E-63	3.12563E-62	133.5658478
TNFRSF14	1.937248181	6.867524467	18.94923723	2.04949E-63	3.6036E-62	133.43234
FECH	-1.75012184	5.693825467	-18.92784811	2.64891E-63	4.59976E-62	133.1811103
CSF3R	1.624247537	3.880841257	18.93865698	2.32683E-63	4.08099E-62	133.1746089
STAMBPL1	1.575927503	4.037354305	18.93541658	2.41905E-63	4.23212E-62	133.1713423
E2F1	1.504824493	4.088654751	18.93306614	2.48821E-63	4.34227E-62	133.1595506
NXPH4	2.99223195	5.279035562	18.92997631	2.58215E-63	4.495E-62	133.1028502
SEMA5B	3.417248091	6.755858048	18.90077961	3.66459E-63	6.33205E-62	132.8637553
PLEKHB1	-2.151534085	3.476506419	-18.89463075	3.94492E-63	6.79964E-62	132.7740632
AC092813.2	-1.963929924	2.19748981	-18.87595671	4.93451E-63	8.46365E-62	132.5500988
MT1H	-4.467024483	3.763833503	-18.87489419	4.99775E-63	8.55116E-62	132.5441667
CYP2B6	-2.14903567	2.255108699	-18.83878156	7.70338E-63	1.31484E-61	132.1056734
TUBB2B	-2.312351501	3.260250638	-18.83644606	7.92192E-63	1.34885E-61	132.0759362
MFSD6L	-2.044997657	2.326493907	-18.79455237	1.30826E-62	2.21675E-61	131.5783048
ANXA9	-1.890157656	5.424279544	-18.78969012	1.38666E-62	2.34392E-61	131.5306291
DOC2A	2.555084925	4.183348781	18.80338	1.17705E-62	1.99928E-61	131.4609565
CNTN1	-2.655789794	2.658656732	-18.76118681	1.95036E-62	3.28091E-61	131.169822
RN7SL138P	1.680183283	4.408785077	18.74760376	2.29451E-62	3.85059E-61	130.9763812
CPAMD8	-2.043569356	3.254399594	-18.72475618	3.01559E-62	5.02455E-61	130.7454724
LAT2	1.671112382	4.52859867	18.71583754	3.35498E-62	5.57675E-61	130.6104488
SASH3	2.282406108	5.61120108	18.7050406	3.81728E-62	6.33017E-61	130.5222683
HADH	-1.635679516	6.838246401	-18.68409047	4.9036E-62	8.09325E-61	130.2647288
DNER	-3.228798001	3.033387816	-18.66308523	6.30274E-62	1.0378E-60	130.0018637
RAB11FIP4	-1.502446109	3.418487737	-18.65821489	6.68038E-62	1.0974E-60	129.9602165
GZMH	2.363133077	5.127110688	18.64372262	7.94308E-62	1.30177E-60	129.7515387
TTC36	-1.652715624	2.667821614	-18.63805413	8.4995E-62	1.38971E-60	129.7175437
TNFAIP8L2	1.910198872	5.078661091	18.61229195	1.15612E-61	1.88151E-60	129.4075756
AL031710.1	-2.930090589	3.250494632	-18.60913975	1.20045E-61	1.94914E-60	129.3633663
NKG7	3.10482873	6.837030914	18.60692169	1.23267E-61	1.9922E-60	129.3611915
GAS6-AS1	1.676566018	3.492044629	18.62516008	9.9145E-62	1.61729E-60	129.3456572

NUPR2	-3.265162353	2.59268158	-18.60795053	1.21762E-61	1.97243E-60	129.3379863
AC007406.3	2.004001816	4.193994389	18.57592005	1.78462E-61	2.8776E-60	128.8393483
AL353152.1	-1.904185723	2.244064382	-18.536376	2.86036E-61	4.5805E-60	128.5022874
RBP2	-1.549529604	2.23582285	-18.53596706	2.87434E-61	4.59239E-60	128.492232
GPSM3	1.872534473	6.604228844	18.53203849	3.01221E-61	4.77992E-60	128.4588593
UPP2	-2.371608853	2.740729006	-18.53304226	2.97637E-61	4.73378E-60	128.4573311
ENPP3	4.649358744	7.848623754	18.53117274	3.04347E-61	4.8186E-60	128.4567373
SLA2	1.560805929	3.671143988	18.53331422	2.96673E-61	4.7292E-60	128.3063967
AC106772.2	-1.888114989	2.137845844	-18.51579552	3.65593E-61	5.77522E-60	128.25588
MIR4435-2HG	1.544367404	4.626213607	18.50465651	4.17512E-61	6.58051E-60	128.1178013
KCTD1	-1.801623498	3.793251112	-18.48306976	5.40012E-61	8.49214E-60	127.872283
SLC16A1	2.202979067	6.537264929	18.47804228	5.73351E-61	8.97609E-60	127.8226681
IGFBP3	3.453787797	11.00070127	18.4734237	6.05787E-61	9.46272E-60	127.7576027
SLC15A4	1.723122005	6.611140025	18.47142974	6.20351E-61	9.66864E-60	127.7367497
LINC01077	2.71285809	4.226694685	18.48061236	5.56059E-61	8.72489E-60	127.6021456
SLC22A8	-3.601175332	2.691873183	-18.45476744	7.56559E-61	1.17653E-59	127.5176749
AIF1L	-3.556640547	7.201722009	-18.41478312	1.21792E-60	1.8898E-59	127.0421274
GAS2L3	2.385732049	4.97636128	18.38953923	1.64475E-60	2.54645E-59	126.7037212
MRAP2	-2.034499222	2.470932585	-18.38049674	1.83158E-60	2.82945E-59	126.6509862
TNNT2	-1.689523397	2.118361069	-18.37371235	1.98555E-60	3.06054E-59	126.564582
ZNF503	-1.819316896	5.237251261	-18.36441001	2.21788E-60	3.40366E-59	126.4699335
C1QB	2.987422346	9.430105289	18.32236005	3.65669E-60	5.58723E-59	125.9686978
SELPLG	2.104623212	6.061909801	18.30405518	4.54539E-60	6.89993E-59	125.7633032
ARHGAP9	1.637066378	4.100031295	18.31244685	4.11396E-60	6.27222E-59	125.7537385
BATF	1.878310923	4.22639603	18.31146086	4.16245E-60	6.33236E-59	125.725042
RUNX3	2.171079933	4.881190158	18.29819419	4.87325E-60	7.3816E-59	125.6334588
ANGPTL1	-2.538355796	3.249346712	-18.28608919	5.62703E-60	8.50496E-59	125.5276671
TMEM91	2.898142655	6.046217054	18.2733063	6.54976E-60	9.85704E-59	125.3882843
PCP4	-3.809622216	3.064511401	-18.26964188	6.84113E-60	1.02734E-58	125.3313052
ERP27	-3.046315307	3.949745576	-18.26594974	7.14779E-60	1.06881E-58	125.3018082
CCL5	2.986109488	7.463042203	18.25929004	7.73605E-60	1.1543E-58	125.2246901
BHLHE41	2.817886606	7.416801907	18.24192467	9.50746E-60	1.4156E-58	125.0176341
SLC37A2	1.803086823	4.207915557	18.23991013	9.73758E-60	1.44678E-58	124.8841883
CD37	1.969203815	5.255518087	18.22298856	1.19037E-59	1.76487E-58	124.7938023
MARVELD2	-2.256277672	4.168277271	-18.2175535	1.26968E-59	1.87848E-58	124.7266836
PLA2G7	2.207903978	4.342905616	18.18288307	1.9157E-59	2.82234E-58	124.1636238
SLC16A7	-2.415267287	3.738475469	-18.16616677	2.33572E-59	3.42671E-58	124.11524
AC009542.2	1.566417439	3.32397644	18.17440787	2.11827E-59	3.11421E-58	123.9819158
HS6ST1	-1.856105613	5.80662252	-18.13349931	3.44032E-59	5.02617E-58	123.7371933
TAP1	1.84101985	7.611001292	18.12819534	3.6635E-59	5.32997E-58	123.6680137
FABP7	4.900099165	6.264101997	18.13042258	3.56807E-59	5.20195E-58	123.4600899
HEPACAM2	-3.850010606	2.633702842	-18.06849631	7.42978E-59	1.07203E-57	122.9434138
LINC01587	1.836198541	3.546623406	18.0848244	6.12375E-59	8.87247E-58	122.9219838
SELENBP1	-1.698648851	6.200143964	-18.0569808	8.5148E-59	1.22104E-57	122.8324087
IKBIP	1.73727098	6.249395235	18.05614935	8.59901E-59	1.23059E-57	122.8250532
FMNL1	1.789185772	4.782608642	18.02909954	1.18429E-58	1.68792E-57	122.4817638
CCDC181	-1.69765753	2.477720072	-18.02515368	1.24088E-58	1.76498E-57	122.4507316
ESRP1	-3.121829522	2.777346104	-18.02336559	1.26741E-58	1.79541E-57	122.413133
NIPAL1	-2.137158599	2.797292084	-17.9795786	2.12709E-58	2.99504E-57	121.9080231
HCLS1	1.938077683	6.452890746	17.97566008	2.22793E-58	3.13073E-57	121.8758332
BTK	1.520919707	3.999290822	17.98186611	2.07034E-58	2.92102E-57	121.8461695
SLC1A4	1.665217595	5.059358255	17.95043805	3.00154E-58	4.20938E-57	121.5793872
CALML3	-1.698820451	2.114142535	-17.92265629	4.16735E-58	5.83263E-57	121.2342146
HPD	-4.052064099	4.047635826	-17.92008566	4.2958E-58	6.0004E-57	121.2201409
AC083862.2	1.521826148	4.416830664	17.91216153	4.71714E-58	6.57581E-57	121.0905637
PHKA2	2.069163265	6.431269936	17.89744607	5.6121E-58	7.80785E-57	120.9571091
B4GALNT3	-2.174851684	3.158257685	-17.8837191	6.59914E-58	9.16285E-57	120.780037
SLC9A3	-3.797935055	4.633264773	-17.87965711	6.92317E-58	9.59373E-57	120.74299

LILRB2	1.675907443	4.372216878	17.87809837	7.05169E-58	9.75251E-57	120.6626728
RASSF10	-2.0322542	2.715701829	-17.86144499	8.5827E-58	1.18232E-56	120.5195671
C1QC	2.856589656	9.435768349	17.83641078	1.15306E-57	1.57908E-56	120.2335617
TAPBP	1.593627135	8.853994363	17.82301606	1.35032E-57	1.84561E-56	120.0778007
FAM83B	-2.245219084	2.327445612	-17.81223599	1.53328E-57	2.0916E-56	119.939033
HSPB7	-2.769026162	3.37785911	-17.8119085	1.53921E-57	2.09561E-56	119.9349494
ZNF395	2.470763495	8.158020801	17.79377423	1.90594E-57	2.57984E-56	119.7275961
LINC01612	-2.348027181	2.306379744	-17.78828525	2.03327E-57	2.74688E-56	119.6565766
ITGB2	2.394341652	6.800736857	17.78371164	2.14583E-57	2.89336E-56	119.6191771
GALNT3	-2.307665643	3.560572067	-17.78106685	2.21374E-57	2.97919E-56	119.5755993
MT1G	-4.937756559	5.938495144	-17.76574035	2.65171E-57	3.55492E-56	119.3789044
CSTA	1.553761395	4.061683037	17.77095404	2.49379E-57	3.34962E-56	119.3674136
CWH43	-2.792407096	2.672094811	-17.755254	3.00023E-57	3.99917E-56	119.2600207
PRF1	2.382473404	5.624518303	17.75105409	3.15232E-57	4.19391E-56	119.232383
AC104072.1	-1.97313664	2.116715839	-17.74990447	3.19528E-57	4.24299E-56	119.2079766
RPS6KA6	-2.264178755	2.983094791	-17.73718166	3.71155E-57	4.90991E-56	119.0557189
CORO1A	2.318479602	6.089200296	17.71514302	4.81054E-57	6.32785E-56	118.8217123
CTH	-1.507634633	3.835863172	-17.70674334	5.31023E-57	6.97204E-56	118.7091111
ADAP2	1.711051562	5.377083732	17.69943287	5.7871E-57	7.56975E-56	118.6363928
SLC47A2	-2.375566295	2.77095841	-17.69986529	5.75774E-57	7.54544E-56	118.6164418
CAV1	2.258904941	8.340649659	17.68503371	6.85504E-57	8.94992E-56	118.453795
C1orf162	2.181999188	5.877792456	17.66481064	8.6951E-57	1.12474E-55	118.2313276
LINC01606	-1.998540555	2.140081474	-17.66540684	8.63437E-57	1.11895E-55	118.2174921
PTCSC3	-2.379078233	2.551753543	-17.65992515	9.20906E-57	1.18902E-55	118.1478874
DTX1	-1.807186776	3.432533965	-17.65719565	9.50931E-57	1.22553E-55	118.1259079
KISS1R	2.670565116	4.22113874	17.6735381	7.84719E-57	1.02262E-55	118.0723027
LCP2	1.930781198	5.355095844	17.6402986	1.15981E-56	1.48652E-55	117.9387542
C1QA	2.716149686	9.574541249	17.63975311	1.16727E-56	1.49062E-55	117.9269452
SIM1	-2.799299508	3.316899599	-17.64012746	1.16215E-56	1.48679E-55	117.9185659
HIF1A-AS2	2.495779016	4.60425934	17.64543394	1.0919E-56	1.40461E-55	117.8303059
FMO5	-2.014923801	3.614580685	-17.628043	1.33944E-56	1.70736E-55	117.7826655
AC103563.7	-2.318350994	2.805665155	-17.62616485	1.36932E-56	1.74227E-55	117.7536748
DEF6	1.850869076	4.92661438	17.57842326	2.39863E-56	3.04639E-55	117.1951471
GZMA	2.784594435	6.333023122	17.57249901	2.57133E-56	3.24805E-55	117.1505072
LDHB	-1.577102231	10.00177283	-17.57273406	2.56425E-56	3.24496E-55	117.1060687
ANGPT2	2.839187974	6.31896747	17.56140508	2.92884E-56	3.69297E-55	117.0198789
RNU6-796P	1.989082962	3.884076346	17.57460195	2.50865E-56	3.18035E-55	116.9609119
SOSTDC1	-3.396034193	3.387355704	-17.55357051	3.21081E-56	4.03398E-55	116.9095364
FERMT3	1.954517942	5.761690301	17.54031094	3.75114E-56	4.69527E-55	116.774321
COL4A5	-2.072373212	3.511392128	-17.53523536	3.98121E-56	4.96622E-55	116.6958562
CGN	-2.499709314	4.225529596	-17.52463796	4.508E-56	5.61334E-55	116.5794626
VAV1	1.689138595	4.359744761	17.5206155	4.7257E-56	5.87397E-55	116.4670305
FOXC1	-2.077329451	5.139487457	-17.5049425	5.67882E-56	7.03369E-55	116.3521507
MAP6	-1.6901984	3.01786413	-17.50149167	5.91321E-56	7.31107E-55	116.3061413
TMC8	1.741782333	4.373279452	17.50531597	5.65401E-56	7.01539E-55	116.2832524
KCNQ1	-1.947818609	5.400998021	-17.49566521	6.33108E-56	7.81392E-55	116.2438681
HSPA7	2.162659163	4.22012282	17.48520323	7.15679E-56	8.81746E-55	115.9482273
GPAT3	-2.801742516	4.95467452	-17.4695658	8.59559E-56	1.05715E-54	115.9401509
ARRDC2	1.922296172	6.663883527	17.45823881	9.81497E-56	1.20289E-54	115.8039462
LAD1	-3.278473543	4.376503422	-17.45689825	9.97026E-56	1.21979E-54	115.7931117
TMPRSS4	-2.211725815	2.303533132	-17.44808756	1.10538E-55	1.34999E-54	115.6750741
RAP1GAP	-2.729215948	5.663318399	-17.44242009	1.18121E-55	1.44009E-54	115.6169961
PAQR7	-1.526210337	5.916179069	-17.42933834	1.37667E-55	1.67547E-54	115.4693431
CYFIP2	-2.139267896	7.002849594	-17.41759041	1.57957E-55	1.91574E-54	115.3185874
MLKL	1.557707419	4.808646109	17.39145754	2.14442E-55	2.57846E-54	115.02146
LINC01871	1.506301818	3.837708568	17.39602246	2.03293E-55	2.44861E-54	114.9461236
AL109615.3	1.94824537	4.55452052	17.38872472	2.21407E-55	2.65764E-54	114.9190742
PLXDC1	2.067317575	4.864180389	17.35367106	3.33557E-55	3.98333E-54	114.5418142

AC008760.2	2.422731481	4.227214415	17.36098096	3.06243E-55	3.66339E-54	114.4484231
AL162724.2	1.521434281	3.526712422	17.34205694	3.82044E-55	4.55458E-54	114.2542349
ALDH6A1	-2.636847938	6.035048703	-17.318812	5.01236E-55	5.95527E-54	114.1729224
FAM169A	-1.921190173	2.3787511	-17.31829278	5.04285E-55	5.98133E-54	114.1652106
FRMD7	-1.560926861	2.03488576	-17.30478646	5.90436E-55	6.95602E-54	113.9905554
YBX3	1.506336081	7.618212094	17.30123093	6.15463E-55	7.23867E-54	113.9716128
LINC02038	-1.787082883	2.443683661	-17.29849689	6.35425E-55	7.46091E-54	113.9359222
NUSAP1	1.916549716	5.292943117	17.272128	8.6442E-55	1.01327E-53	113.6398064
LGALS1	2.073583242	10.17827701	17.25702025	1.03103E-54	1.20655E-53	113.4514958
ALB	-3.419110426	3.343991845	-17.25061327	1.11104E-54	1.298E-53	113.3757563
LINC02381	-2.2403621	5.493418628	-17.24775042	1.14876E-54	1.33984E-53	113.354732
BMP7	-2.353251402	2.347721421	-17.24139479	1.23714E-54	1.43813E-53	113.2654775
LSP1	2.112874285	5.63139338	17.1987539	2.03368E-54	2.3446E-53	112.792194
ST6GAL1	-2.189392668	6.480669688	-17.18826609	2.29799E-54	2.64061E-53	112.6547407
PFKFB4	1.883681184	4.812085801	17.1893041	2.27037E-54	2.61316E-53	112.6430422
CA8	-2.015285086	2.730689145	-17.18047777	2.51621E-54	2.8819E-53	112.5620589
PFKFB2	-1.920864912	3.983884162	-17.1707041	2.81955E-54	3.2188E-53	112.4525944
ABAT	-2.236519318	4.328733975	-17.16948814	2.85976E-54	3.25938E-53	112.442199
BIN2	1.576138154	4.704697785	17.14801687	3.6719E-54	4.17142E-53	112.1821934
TBXAS1	1.523334596	4.91292693	17.13755474	4.14736E-54	4.70393E-53	112.0761522
SFXN2	-1.677092033	4.035009185	-17.13171297	4.43909E-54	5.02666E-53	112.0007312
RHBG	-2.922058004	2.495729396	-17.12464309	4.81968E-54	5.44881E-53	111.9001129
LPCAT1	2.306696931	7.602960092	17.10776811	5.86499E-54	6.60921E-53	111.7218075
RNASET2	2.848139305	6.938184804	17.10625849	5.96887E-54	6.70468E-53	111.7189271
MPC1	-1.63969813	7.512773604	-17.10710764	5.91021E-54	6.64946E-53	111.7079698
SNHG12	1.915368306	4.521423872	17.10413828	6.11787E-54	6.86104E-53	111.6129325
OXGR1	-2.101061764	2.29503894	-17.09370063	6.90734E-54	7.73401E-53	111.5551307
ARHGDIB	1.686699705	9.424605206	17.08943676	7.25838E-54	8.11408E-53	111.5119142
PRDM1	1.912046113	5.095177375	17.07917273	8.1782E-54	9.12776E-53	111.3901305
KRBA1	2.313137557	5.908818694	17.05694048	1.05891E-53	1.1781E-52	111.1496736
GRIK5	-2.34034239	2.625844683	-17.05886619	1.03548E-53	1.15387E-52	111.1462645
S100A14	-2.2890648	4.188667907	-17.05016079	1.14568E-53	1.26859E-52	111.0581032
PPM1H	-1.996974605	4.199425983	-17.04398956	1.23083E-53	1.36026E-52	110.9852275
LTB4R	1.573210571	4.132617497	17.05058459	1.14006E-53	1.26436E-52	110.9763086
AC148477.4	-2.859262862	2.587847569	-17.04296963	1.24549E-53	1.37259E-52	110.9541378
NLRC5	1.685750398	4.761535461	17.0335037	1.39022E-53	1.52968E-52	110.8519344
AC130371.2	-1.938222665	3.049662804	-17.01870815	1.65079E-53	1.80503E-52	110.6885754
PHYHD1	-2.527657224	4.176277797	-17.0147225	1.72896E-53	1.88756E-52	110.6494676
COL4A4	-2.081427309	4.348702698	-16.9903031	2.2954E-53	2.49042E-52	110.3655624
CAVIN3	2.108634064	7.192097253	16.96388102	3.11856E-53	3.37305E-52	110.057928
CLDN14	-1.775171252	2.730895678	-16.95596133	3.41848E-53	3.68606E-52	109.9660012
AC108463.1	1.737669678	4.420233861	16.95844637	3.3214E-53	3.5869E-52	109.9309086
GAS1	-1.943462643	3.576263702	-16.94911703	3.70077E-53	3.97819E-52	109.8849945
AL590666.2	1.83011858	4.233623303	16.95567054	3.43003E-53	3.69282E-52	109.8498777
CLIC5	-2.722031653	3.829215378	-16.93659053	4.27905E-53	4.58572E-52	109.7432695
HOXB6	-2.009035574	4.785580956	-16.93101227	4.56479E-53	4.88447E-52	109.6828451
SCN1B	1.587005856	4.663862557	16.91937493	5.2237E-53	5.57247E-52	109.5326258
MS4A6A	2.295456016	6.140609877	16.90586166	6.1088E-53	6.49687E-52	109.404018
ASF1B	1.542347909	4.223059431	16.91129685	5.7361E-53	6.10978E-52	109.3825508
PAPPA2	-2.717224761	2.65646262	-16.90037789	6.50933E-53	6.90187E-52	109.3077874
CLEC2B	1.843077269	5.248707112	16.88883454	7.44017E-53	7.8769E-52	109.2008066
HMOX1	2.721256477	9.104118639	16.86773189	9.49865E-53	9.99572E-52	108.9506273
TYMS	2.012391521	6.034916037	16.86620696	9.66775E-53	1.01584E-51	108.9451913
APOBEC3G	1.799995093	4.922991407	16.86282742	1.00533E-52	1.05319E-51	108.884532
SLC13A3	-3.850086544	3.427350556	-16.85800549	1.06301E-52	1.11029E-51	108.8344651
CD52	2.279547026	6.866399697	16.85614604	1.08613E-52	1.13275E-51	108.8203695
GZMB	1.839513609	4.497869055	16.85830429	1.05934E-52	1.10812E-51	108.7782477
CST7	2.69752295	6.09729475	16.84463866	1.24077E-52	1.28446E-51	108.6944983

CDH16	-2.694287295	8.645711531	-16.84777343	1.19659E-52	1.24239E-51	108.6899795
C1QTNF6	1.540491885	4.309426109	16.84599922	1.2214E-52	1.26628E-51	108.6461479
BMPR1B	-2.802793992	2.768693053	-16.83754235	1.3469E-52	1.39226E-51	108.582965
AL109615.1	2.693279792	4.223754858	16.85077429	1.15577E-52	1.20358E-51	108.4967162
IYD	-1.985311467	2.440477512	-16.81920749	1.66496E-52	1.71344E-51	108.3845164
MS4A7	2.329130787	6.168709551	16.8117268	1.81533E-52	1.85999E-51	108.318749
RAB42	3.010912124	5.338117277	16.81526631	1.74256E-52	1.78804E-51	108.2439991
HCK	1.916351712	5.493151903	16.79799073	2.12763E-52	2.1736E-51	108.158551
ACOT11	-1.623980526	3.355645174	-16.79476446	2.20844E-52	2.25287E-51	108.1091728
LRRN2	-2.372181131	3.186513337	-16.78958768	2.34455E-52	2.38824E-51	108.0387151
TAGLN3	-1.930789757	2.277000077	-16.78560658	2.4549E-52	2.497E-51	107.9972697
LINC01428	1.916908999	3.782244934	16.80252051	2.01913E-52	2.06577E-51	107.9805122
ILDR1	-2.036893514	4.305701174	-16.7763581	2.73166E-52	2.77046E-51	107.8969148
AC107021.2	1.536643436	3.81949453	16.78027964	2.61069E-52	2.65162E-51	107.7970776
HLX	1.787706128	4.550756218	16.76583211	3.08472E-52	3.11949E-51	107.7273171
WAS	1.919057642	5.368067528	16.74050926	4.13206E-52	4.13678E-51	107.494071
MYBL2	1.658979022	3.700129047	16.75646917	3.43687E-52	3.4656E-51	107.4751156
AC015910.1	-3.634977832	2.73103661	-16.7408642	4.11516E-52	4.12578E-51	107.4665612
THRB	-1.744789777	3.818994441	-16.73437011	4.4354E-52	4.4215E-51	107.4117205
MPPED2	-1.787999822	2.854269311	-16.73323697	4.49377E-52	4.47332E-51	107.3984496
PLK2	2.107425703	6.962753116	16.73225089	4.54519E-52	4.51808E-51	107.3901743
APBB1IP	2.453483113	6.147963973	16.71759902	5.3822E-52	5.33494E-51	107.2359538
P2RY1	1.568693268	3.932327865	16.71084314	5.81835E-52	5.75097E-51	107.0185555
CD7	1.682416557	4.045903099	16.69307303	7.14144E-52	7.00923E-51	106.8174833
PPP1R18	1.648142171	6.721344308	16.6693993	9.38168E-52	9.19509E-51	106.6660006
CARD16	1.600624089	5.137146846	16.66631466	9.7211E-52	9.51444E-51	106.6431947
KDF1	-2.082697242	3.973668101	-16.66573811	9.78589E-52	9.56448E-51	106.6231066
AL049555.1	-2.30516307	2.437592868	-16.66145352	1.02811E-51	1.00344E-50	106.5655174
SH3GL2	-2.942158143	2.802402717	-16.65304139	1.13271E-51	1.104E-50	106.4598148
C7	-4.300928578	6.108292324	-16.65280011	1.13587E-51	1.10554E-50	106.4562253
BTN3A2	1.828000953	6.581675105	16.62937657	1.48753E-51	1.43781E-50	106.2103106
THEMIS2	1.942331864	5.529492634	16.62814889	1.5087E-51	1.45626E-50	106.2069142
LGI2	-1.972470378	3.059004587	-16.61579664	1.73919E-51	1.67643E-50	106.0461123
GABARAPL1	-1.73678547	7.529538838	-16.61224159	1.81182E-51	1.74403E-50	106.0008508
DNAH11	1.946191685	3.688975704	16.63064839	1.46591E-51	1.41887E-50	105.980683
C1orf116	-2.701599372	2.933451864	-16.60942301	1.87155E-51	1.79905E-50	105.9626524
PLEKHO1	1.611591996	5.509760644	16.60558236	1.95611E-51	1.87776E-50	105.9496437
GBP2	1.870793946	6.944157583	16.60083332	2.06596E-51	1.97508E-50	105.8792886
HOMER1	-1.642888913	3.362484408	-16.5958363	2.1882E-51	2.08058E-50	105.823293
CTSW	2.354854255	5.224485826	16.58009826	2.62239E-51	2.48665E-50	105.6170899
PLPP4	-2.185178454	2.728929573	-16.57823321	2.67924E-51	2.53712E-50	105.6123013
PP7080	-2.329151214	5.838872575	-16.56858543	2.99354E-51	2.82328E-50	105.5128063
EDA2R	1.847072076	4.947483307	16.56681352	3.05514E-51	2.87751E-50	105.4798637
JAK3	1.792484042	4.692367453	16.52922112	4.70577E-51	4.39666E-50	105.0282549
PTP4A3	1.756517753	6.733389314	16.50486591	6.22423E-51	5.79218E-50	104.7813017
CD2	2.665000699	5.849848887	16.48233998	8.06043E-51	7.48102E-50	104.5264889
OLFML2B	2.49268755	6.456450274	16.46773769	9.53031E-51	8.83353E-50	104.371186
LDHA	1.761308678	10.57579991	16.44979661	1.17073E-50	1.07942E-49	104.137561
TRIM9	1.51029064	3.654456653	16.45900631	1.05341E-50	9.75096E-50	104.0840149
SLC36A2	-2.991612503	2.556652411	-16.44077181	1.29834E-50	1.19549E-49	104.0267676
HNF4A-AS1	-1.624210122	2.23541805	-16.43524196	1.38329E-50	1.27038E-49	103.9760718
PROZ	-1.864739124	2.321230582	-16.4300957	1.46733E-50	1.34579E-49	103.9215688
COL9A2	-2.008612318	3.612121875	-16.41072831	1.83186E-50	1.67355E-49	103.701796
PPP1R1A	-3.97647502	4.267559999	-16.40348369	1.99035E-50	1.80889E-49	103.6281269
SLC5A2	-2.339774009	2.526504183	-16.4041653	1.97487E-50	1.79716E-49	103.6195601
AP000439.2	3.127918919	4.733148449	16.41888255	1.6685E-50	1.52629E-49	103.551723
CGREF1	2.414875649	5.094254638	16.39777377	2.12484E-50	1.92612E-49	103.5113402
ZFAS1	1.545962464	7.879455304	16.39054786	2.30812E-50	2.08685E-49	103.4773274

FAM171A1	-2.073866546	5.824197528	-16.38024021	2.5972E-50	2.3409E-49	103.3620921
HSPB8	2.345958786	8.654275586	16.38017487	2.59914E-50	2.3409E-49	103.358292
CXCR3	1.795853613	4.000881588	16.39095617	2.29736E-50	2.07981E-49	103.3275081
ITGAL	2.069104201	5.10769402	16.36639709	3.04304E-50	2.72666E-49	103.1874401
PDLIM1	1.722769674	8.884657802	16.36115504	3.23114E-50	2.8915E-49	103.1448634
CBLC	-1.922375587	3.112984743	-16.34378278	3.94146E-50	3.51368E-49	102.9387815
CYP4F2	-2.587557899	2.51557085	-16.34468255	3.90111E-50	3.48659E-49	102.9369879
EBI3	1.775445481	4.678591009	16.3445047	3.90906E-50	3.48923E-49	102.92055
TMEM72	-3.374787188	5.377890607	-16.33659147	4.27929E-50	3.80515E-49	102.8581429
PTTG1	1.588684975	4.299005631	16.34010169	4.11093E-50	3.66009E-49	102.8354768
TMCC1	1.660377355	6.233117583	16.32247793	5.0286E-50	4.46009E-49	102.7052535
ADH1B	-3.106546864	3.921522009	-16.32065285	5.1346E-50	4.54835E-49	102.682051
RNU6-339P	2.100332479	4.11575135	16.32878632	4.67873E-50	4.15505E-49	102.5884086
DGKD	1.71189154	4.935661822	16.30745397	5.97057E-50	5.26886E-49	102.5269164
RASD1	-2.785152071	6.8420189	-16.30397555	6.21265E-50	5.47558E-49	102.4751449
PTP4A2P2	1.608606016	3.729466648	16.31047131	5.76822E-50	5.10316E-49	102.3887495
LHX1	-2.259493537	2.332784295	-16.29492256	6.88959E-50	6.04936E-49	102.3767667
ETV7	1.816677554	4.482606868	16.29205748	7.1188E-50	6.23496E-49	102.2867133
GGTA1P	1.707823161	4.904764899	16.28087713	8.08837E-50	7.06647E-49	102.2229279
STRA8	1.797362894	3.866654153	16.29392814	6.9683E-50	6.1108E-49	102.1930907
MISP3	-1.58181114	4.321872559	-16.27148926	9.00354E-50	7.84642E-49	102.1180515
OR51E1	1.590489573	3.879452731	16.28183109	8.00073E-50	6.99864E-49	102.1013099
DEPP1	2.330619119	9.199496397	16.2619638	1.00378E-49	8.73685E-49	102.0149184
NR3C2	-2.052870786	4.355757996	-16.26176403	1.00607E-49	8.74591E-49	102.0095168
NCF4	1.741394027	5.225394917	16.25582465	1.07663E-49	9.34772E-49	101.9518449
AC087468.1	1.64665453	3.435050464	16.27505867	8.64399E-50	7.54248E-49	101.9184179
CYTH4	1.603535334	4.669462019	16.25487385	1.08838E-49	9.43799E-49	101.9163472
NTNG1	-1.528071954	2.116348634	-16.22911534	1.46017E-49	1.25996E-48	101.618519
MIR200CHG	-2.132857449	2.30073932	-16.20389526	1.94664E-49	1.66943E-48	101.3442004
UBE2C	1.991353187	4.512686979	16.20814031	1.85468E-49	1.59253E-48	101.306787
FABP1	-3.06728514	2.960260705	-16.19957563	2.04487E-49	1.74939E-48	101.2842807
NPM1P25	-1.552930362	3.896408096	-16.19711879	2.10294E-49	1.79687E-48	101.273655
LOXL2	2.628253828	6.492710445	16.1587708	3.25486E-49	2.77099E-48	100.8543465
AC005082.1	-2.49381864	2.906699094	-16.16066278	3.1855E-49	2.71524E-48	100.8459354
EPB41L5	-1.667589567	4.785821002	-16.1562854	3.34828E-49	2.84705E-48	100.8115133
B4GALNT2	-2.318973418	2.616244407	-16.15540323	3.38208E-49	2.8723E-48	100.7889062
TRBV28	2.317777088	5.102991028	16.14922895	3.62834E-49	3.07771E-48	100.6936259
MIR210	2.014198654	4.043640071	16.14906712	3.63503E-49	3.07965E-48	100.5407773
DPEP1	-3.647395	4.226463949	-16.11962117	5.08184E-49	4.259E-48	100.4022255
FCGR2C	1.510809982	3.433050556	16.13839755	4.10436E-49	3.47308E-48	100.3835545
CAPN12	2.085478421	4.53922424	16.11718779	5.22445E-49	4.37329E-48	100.2742753
ZNF37CP	1.87042154	3.722498407	16.12729723	4.65691E-49	3.92167E-48	100.2535665
AC019197.1	-1.723776011	2.454386896	-16.10634713	5.90993E-49	4.93528E-48	100.2412091
VWF	3.036620133	8.785978274	16.09978492	6.36774E-49	5.31126E-48	100.167072
HOXD8	-1.760927532	6.414118811	-16.09212698	6.94693E-49	5.78745E-48	100.0849929
EMP3	1.637720385	7.666751451	16.08520511	7.51553E-49	6.25371E-48	100.0048143
AC104794.4	-1.91326884	3.709450483	-16.08148062	7.84046E-49	6.51634E-48	99.95986556
MSC	2.644847535	6.132004573	16.07993337	7.97954E-49	6.62406E-48	99.9593195
GLDC	-2.400790172	4.499201451	-16.07574889	8.3681E-49	6.93839E-48	99.9027091
STAP1	-2.955660852	3.064548635	-16.06412035	9.54992E-49	7.89957E-48	99.75098142
FXYD5	1.773455175	7.234697865	16.06018292	9.98671E-49	8.25112E-48	99.71935059
MAL2	-3.142193928	6.181189935	-16.02582726	1.47507E-48	1.21299E-47	99.32276705
POU5F1	2.583917811	5.5817653	16.00141228	1.94581E-48	1.59634E-47	99.04696154
PLA2R1	-1.94327973	3.672272426	-15.97245723	2.70185E-48	2.20883E-47	98.72695546
SLC30A2	-2.553537868	2.860809202	-15.95653763	3.23591E-48	2.63927E-47	98.53560465
TMC4	-2.735985671	4.933657763	-15.9544778	3.31231E-48	2.69843E-47	98.53488631
PALD1	1.738168599	5.494108791	15.94581696	3.6537E-48	2.97309E-47	98.44512375
LYPD6B	-2.229753822	2.367547691	-15.9360074	4.08294E-48	3.31467E-47	98.31027908

CASP1	1.620096948	5.582564594	15.92961078	4.38959E-48	3.55536E-47	98.26199281
AC144831.1	-1.703646979	2.838291088	-15.91317264	5.28716E-48	4.27246E-47	98.06119676
ARHGAP25	1.53472329	5.208907946	15.8893475	6.9226E-48	5.55554E-47	97.80779154
MCF2L-AS1	-1.721035949	2.617362329	-15.88817594	7.01492E-48	5.61675E-47	97.77849643
MUC3A	1.515109942	3.517092996	15.90460193	5.82554E-48	4.69127E-47	97.753546
THSD4	-1.718813534	3.545527342	-15.87207471	8.41552E-48	6.70749E-47	97.59737332
NUDT4	-1.582945149	5.039285019	-15.87055593	8.56124E-48	6.81587E-47	97.58321282
PADI2	-2.106609938	4.167407756	-15.86939146	8.67466E-48	6.89833E-47	97.56811614
CSPG4	2.48780046	6.434910035	15.85284393	1.04583E-47	8.28849E-47	97.39706636
AC073218.2	2.014681396	3.715714118	15.85529129	1.01731E-47	8.08075E-47	97.1572124
TRIB3	2.495011075	6.074309493	15.82155052	1.48917E-47	1.17488E-46	97.04516497
CORO2B	-1.879856137	3.477447957	-15.78803022	2.17374E-47	1.70536E-46	96.64993969
GJC1	1.945372134	4.702250305	15.79074352	2.10823E-47	1.65582E-46	96.6305557
DACH1	-1.923793248	3.578988035	-15.78262331	2.31042E-47	1.80652E-46	96.58881
EHD2	2.276745464	8.761879018	15.77133103	2.62414E-47	2.04269E-46	96.46810835
AC018553.1	1.857914854	4.401516018	15.77323579	2.56839E-47	2.00598E-46	96.39415809
HAPLN1	1.781308412	3.771022304	15.78321957	2.29494E-47	1.79642E-46	96.38630719
CGNL1	-2.396457693	6.188724345	-15.75747553	3.06771E-47	2.37476E-46	96.30745143
FKBP10	2.544611245	7.6706195	15.74967958	3.34939E-47	2.58664E-46	96.22015895
SLFN13	1.707563572	4.849698079	15.74959693	3.35251E-47	2.58664E-46	96.21319136
AC023024.2	1.893873153	4.121002138	15.7619018	2.91843E-47	2.2617E-46	96.2075658
HOXB8	-2.462713136	4.464901913	-15.73325019	4.03035E-47	3.0892E-46	96.0431255
HAPLN3	1.565188702	4.148655464	15.73661852	3.88031E-47	2.98073E-46	95.98808514
CMTM4	-1.999021657	5.672276998	-15.72489898	4.42778E-47	3.3864E-46	95.95023558
EYA4	-1.652162379	2.472522869	-15.72160349	4.59516E-47	3.50293E-46	95.90448535
BTG2	-1.848662228	8.230550458	-15.70731509	5.397E-47	4.0919E-46	95.72739176
TPX2	1.738855634	4.43917037	15.71138351	5.15544E-47	3.91724E-46	95.72510696
CES4A	2.447687649	4.748755033	15.70784245	5.36507E-47	4.0721E-46	95.63251778
SHISA3	-3.124007734	4.590138357	-15.69022868	6.54107E-47	4.95394E-46	95.56408074
MAB21L4	-2.244681922	2.52983596	-15.67463122	7.79536E-47	5.89113E-46	95.37114149
SIGLEC1	1.696475032	4.15065428	15.66413249	8.77202E-47	6.60782E-46	95.15666663
DEGS2	-1.899112919	3.140146343	-15.6408243	1.13988E-46	8.5497E-46	94.99844621
DNASE1L3	-2.146394596	4.10229592	-15.63308415	1.24342E-46	9.3064E-46	94.91547421
RASSF4	1.758486349	6.782733576	15.63127896	1.26889E-46	9.48688E-46	94.8953558
CCDC160	-1.861502804	3.292384	-15.62899757	1.30183E-46	9.72271E-46	94.86674978
SDHAP3	2.163617916	6.105454103	15.62313453	1.39043E-46	1.03734E-45	94.81999127
RNA5SP18	1.538816075	3.381033533	15.63356113	1.23678E-46	9.26659E-46	94.67639031
C4orf47	1.820426886	4.793688154	15.6136296	1.54702E-46	1.15171E-45	94.67566419
GPD1L	-1.743583453	5.397131829	-15.60467018	1.71069E-46	1.26682E-45	94.60353566
ASPHD1	2.051150705	4.760741253	15.60490085	1.70627E-46	1.26488E-45	94.54814108
NCKAP1L	1.877871444	4.793743506	15.5949037	1.90884E-46	1.41057E-45	94.45960104
RNF43	-1.724230559	3.012134385	-15.59100375	1.99422E-46	1.47211E-45	94.44422019
SLC29A2	-1.874558364	3.760376402	-15.58818019	2.05841E-46	1.51629E-45	94.41122635
CD86	1.831986239	4.848449801	15.58056146	2.24206E-46	1.6481E-45	94.30998055
SYNE4	-1.680494121	2.670838818	-15.57339236	2.42977E-46	1.77861E-45	94.24821122
SLC1A3	1.845079228	4.329560191	15.56304866	2.72856E-46	1.99316E-45	94.03169172
PRSS22	-1.854247149	2.270075885	-15.55274885	3.06248E-46	2.23475E-45	94.01193108
ITGA5	1.949269518	7.171368983	15.53954069	3.551E-46	2.58047E-45	93.86868821
KCCAT198	1.662239047	3.77945521	15.55060152	3.13708E-46	2.2868E-45	93.81451436
NEK6	2.226094606	7.234442798	15.53431291	3.76518E-46	2.72761E-45	93.81174294
CALCA	-2.525955392	2.272477561	-15.53174498	3.87506E-46	2.80431E-45	93.77062197
HPCAL4	-1.717240289	2.273922394	-15.52741731	4.06752E-46	2.93751E-45	93.72833127
CD84	1.850536241	4.275032739	15.53436105	3.76315E-46	2.72761E-45	93.69860374
CD48	1.948479186	5.235760112	15.51155039	4.85836E-46	3.49422E-45	93.56244855
DEPTOR	-1.552415689	6.267251514	-15.49675132	5.73358E-46	4.11524E-45	93.39816879
CD3D	2.450438673	5.539430267	15.49662312	5.74181E-46	4.11692E-45	93.3872146
NAP1L2	-2.241733817	3.682961747	-15.4934601	5.94867E-46	4.25652E-45	93.35242724
SLAMF6	1.743571688	4.22734538	15.47407298	7.38933E-46	5.26048E-45	93.04017216

BMP1	1.654146606	5.42483412	15.45913749	8.73231E-46	6.19763E-45	92.99040337
C5orf46	2.967067933	4.462043458	15.48082495	6.85187E-46	4.8878E-45	92.94656545
CALHM6	2.393866288	5.588813446	15.45403539	9.24478E-46	6.54146E-45	92.91907092
SH2D2A	1.503510747	3.822142938	15.45878548	8.76673E-46	6.21577E-45	92.82508394
PGF	3.254638002	6.19505977	15.44104778	1.06888E-45	7.53274E-45	92.76413375
DDIT4	2.207006894	9.358558549	15.43267358	1.1737E-45	8.24656E-45	92.68397067
PTH1R	-2.896852117	5.552047213	-15.43219843	1.17995E-45	8.28213E-45	92.67511064
BTN3A1	1.691508267	6.371415682	15.42692201	1.25158E-45	8.76731E-45	92.62017445
TNFSF13B	1.923394634	4.778896368	15.4278763	1.23831E-45	8.68305E-45	92.58871796
MIR155HG	1.616429634	3.721506981	15.43348025	1.16317E-45	8.1808E-45	92.50352476
PYGL	1.915681173	6.396261104	15.40042313	1.68244E-45	1.17268E-44	92.32872614
SLC7A13	-2.329767176	2.240500984	-15.39904592	1.7085E-45	1.18848E-44	92.29644448
CYP2J2	4.046640329	6.965514107	15.39769608	1.73443E-45	1.20532E-44	92.28866376
SLC14A2	-2.150704319	2.204229928	-15.39475712	1.79226E-45	1.24427E-44	92.25109756
RAPGEF3	-1.594656822	4.210049849	-15.39039231	1.8817E-45	1.30378E-44	92.20865647
HOXB5	-1.695706481	3.929632161	-15.38637536	1.96795E-45	1.3595E-44	92.16376537
CNKSR1	-1.828636892	2.650771418	-15.3792942	2.12969E-45	1.46689E-44	92.08406029
ADA	1.598857813	4.628931902	15.37424333	2.2531E-45	1.54884E-44	92.01115605
RF00601	-1.549825256	2.039793039	-15.36794717	2.41698E-45	1.65986E-44	91.93283067
RAC2	2.073095566	6.304978777	15.36388413	2.52898E-45	1.73508E-44	91.92710195
SIRPG	1.523518236	3.511639596	15.38133424	2.08178E-45	1.43672E-44	91.89354682
TRNP1	-2.363657885	3.852583102	-15.3310528	3.64614E-45	2.49177E-44	91.54879658
SHISA2	-1.98961608	2.862697837	-15.32794645	3.77449E-45	2.57697E-44	91.51080561
TSPAN33	-1.696855525	7.359122426	-15.28254695	6.25611E-45	4.24227E-44	91.00499185
ITGB6	-2.481274431	4.391350116	-15.27596494	6.73125E-45	4.56005E-44	90.94502264
AC023421.1	-1.966253587	2.18105038	-15.27483773	6.81616E-45	4.6042E-44	90.92085177
MCAM	2.09968416	8.230847879	15.26033697	8.00858E-45	5.38885E-44	90.76750908
PPFIA4	1.859937871	4.059140521	15.26267548	7.80308E-45	5.25562E-44	90.64134726
FCGR2A	1.858194823	5.823923428	15.23787437	1.02791E-44	6.88357E-44	90.53434033
DCN	-3.303967274	4.773987481	-15.23570435	1.05299E-44	7.04477E-44	90.50137858
RASSF2	1.979994977	5.688914528	15.22395674	1.19973E-44	8.00355E-44	90.38087165
LDHD	-2.379980886	5.037567344	-15.22077231	1.24291E-44	8.27582E-44	90.33704575
CNTD2	-1.581593576	2.225802285	-15.21874006	1.27127E-44	8.45663E-44	90.2939651
EOMES	1.522950572	3.484065444	15.23446257	1.06761E-44	7.13578E-44	90.2578294
SHROOM3	-1.890584444	3.939643967	-15.20951057	1.40842E-44	9.35119E-44	90.20306484
GLOD5	-1.680181431	2.90877279	-15.19782392	1.60345E-44	1.06159E-43	90.07657772
TCEAL2	-2.781439442	2.747818188	-15.19835642	1.59401E-44	1.05633E-43	90.06172683
CADPS2	-1.500123519	5.507846794	-15.19261418	1.69886E-44	1.12369E-43	90.02299637
PRR15L	-3.265732049	4.086023438	-15.18530558	1.84233E-44	1.21743E-43	89.94453649
BSPRY	-2.380847382	4.125575242	-15.18157609	1.92013E-44	1.26765E-43	89.8977032
IL10RA	1.970663674	5.390468632	15.17600769	2.04243E-44	1.34585E-43	89.84505306
PVALB	-4.075834975	3.20758952	-15.17707074	2.01849E-44	1.33133E-43	89.83915025
MAGI3	-1.557566572	3.773212101	-15.17536555	2.05702E-44	1.35419E-43	89.82806646
TRBC2	2.559605111	6.037205147	15.16289625	2.36196E-44	1.55202E-43	89.70444998
GBP5	1.967228039	4.254831228	15.1606345	2.42192E-44	1.58993E-43	89.52464142
KRT7	-3.315938821	4.636409552	-15.14409274	2.90912E-44	1.90084E-43	89.49045594
LINC02041	1.971436482	4.141689689	15.15617986	2.54449E-44	1.66882E-43	89.45489024
COL4A3	-1.883060643	4.193246646	-15.1306381	3.3766E-44	2.20013E-43	89.33351533
SLC25A25	-1.67279629	4.702039035	-15.12043545	3.78039E-44	2.45638E-43	89.22251345
RPS24P17	-1.94420958	2.361813066	-15.11415244	4.05266E-44	2.62841E-43	89.1471911
PFKP	2.059630732	9.110067599	15.11247407	4.12865E-44	2.67027E-43	89.13884411
CAPS	-1.601025567	4.076813141	-15.10944149	4.26956E-44	2.75631E-43	89.09961667
RDH10	-1.650880049	5.789524767	-15.10249032	4.61092E-44	2.96846E-43	89.03030577
CHST11	1.591917801	5.328173756	15.09498043	5.01036E-44	3.22266E-43	88.95739895
DEFB1	-3.493569319	9.096889072	-15.0915621	5.20343E-44	3.34376E-43	88.86160804
CCL4	2.126904601	5.24709587	15.07564603	6.20474E-44	3.96537E-43	88.72311508
BDH1	-1.714871534	3.103697027	-15.0615849	7.24803E-44	4.61526E-43	88.57371359
CYP27B1	-1.736539475	2.765867613	-15.05614245	7.69728E-44	4.88798E-43	88.51340568

DIRAS1	-1.937820122	2.508090805	-15.04725428	8.49142E-44	5.38251E-43	88.41169489
APLN	2.390056556	7.191859977	15.04176121	9.02254E-44	5.714E-43	88.35735966
HS3ST2	1.677779618	3.413741229	15.06043289	7.34089E-44	4.67014E-43	88.2991225
HLA-DQB1	2.350113908	8.230506112	15.02919588	1.03654E-43	6.54074E-43	88.21575939
BCAM	-1.777699399	8.249213264	-15.03052247	1.02147E-43	6.45729E-43	88.21204131
SLAMF7	1.861405098	4.368946242	15.03608941	9.60573E-44	6.07784E-43	88.19830622
CCR5	2.044201692	4.726085153	15.02640757	1.06894E-43	6.73913E-43	88.12386712
VASH1	1.902368282	5.589687427	15.01073251	1.27082E-43	7.94742E-43	88.03048076
GDF6	2.139354047	4.231943753	15.01256069	1.24544E-43	7.79568E-43	87.86027604
MRGPRF	-1.936409282	3.373223208	-14.990612	1.5866E-43	9.85183E-43	87.78940608
TDGF1	-2.415680822	2.35499434	-14.98971418	1.60238E-43	9.93222E-43	87.77113359
PLCB2	1.542863415	4.353446395	14.99128933	1.57479E-43	9.78721E-43	87.75122356
NXPH2	-1.80281651	2.129827797	-14.98044453	1.7748E-43	1.09718E-42	87.67180977
HLA-H	1.712166173	8.174312679	14.97577278	1.86859E-43	1.15312E-42	87.63365942
SIT1	1.711767767	4.106249719	14.98608318	1.66784E-43	1.03288E-42	87.62257338
ABCA4	-2.009147429	2.323309699	-14.95280328	2.40671E-43	1.47868E-42	87.37240457
ANK2	-2.373354693	4.590234249	-14.9492303	2.50331E-43	1.53668E-42	87.34499196
AL365181.3	1.557287769	3.945616285	14.95315109	2.39751E-43	1.47432E-42	87.25031734
CD4	1.813292727	6.867657211	14.93006916	3.09123E-43	1.89426E-42	87.12872086
HLA-DPB1	2.046157364	9.742669334	14.92333564	3.32901E-43	2.0364E-42	87.05516443
CLSTN2	-2.030889256	2.924428522	-14.92098475	3.41625E-43	2.08794E-42	87.02278851
MST1L	-2.201124522	3.014790662	-14.9007212	4.26926E-43	2.6002E-42	86.79800777
SPARC	1.945947047	11.45956798	14.89503609	4.54465E-43	2.76312E-42	86.72119078
ABCC3	2.315298235	6.540511477	14.89046988	4.77862E-43	2.90284E-42	86.70754887
NUAK2	-2.061855423	5.516304773	-14.88977163	4.81544E-43	2.92267E-42	86.69459464
EVI2A	1.8119525	5.267801295	14.86746844	6.15285E-43	3.72149E-42	86.45488129
ERICH4	-1.817116225	2.744182838	-14.86534204	6.29826E-43	3.80615E-42	86.41858568
CLDN10	-2.370235118	5.730105711	-14.85508013	7.04964E-43	4.24921E-42	86.31181822
ZDHHC2	-1.898774853	5.178674985	-14.85460653	7.08639E-43	4.26769E-42	86.30886551
LMO7-AS1	-1.673156282	2.931899507	-14.84872648	7.55899E-43	4.54449E-42	86.23974873
IGFBP2	-2.418594974	5.120223766	-14.84675988	7.72397E-43	4.63968E-42	86.2251016
RGS10	1.522235682	6.603782153	14.81609105	1.08141E-42	6.45709E-42	85.88162448
PTPN13	-1.754117139	5.189236878	-14.81220199	1.12853E-42	6.7327E-42	85.8445115
PDK1	1.584479469	4.885572624	14.80180389	1.26481E-42	7.5265E-42	85.72885915
APOBEC3C	1.960840421	6.900531098	14.79431106	1.37308E-42	8.16382E-42	85.64520193
ELMO1	1.505948174	4.922453757	14.78812251	1.46943E-42	8.72928E-42	85.58560638
AQP3	-2.318482787	7.839698682	-14.78368099	1.54271E-42	9.14907E-42	85.508424
IL32	2.278278176	8.547355135	14.75305612	2.15743E-42	1.27622E-41	85.19602503
DOK2	1.602550392	4.787023889	14.7487188	2.26231E-42	1.33488E-41	85.14246416
EVI2B	1.965844724	5.990673654	14.74596017	2.33165E-42	1.37348E-41	85.13278354
SH3BP2	1.591545737	6.201301109	14.72793397	2.83995E-42	1.6617E-41	84.92690999
SLAMF8	2.021813559	4.965889431	14.72946796	2.7927E-42	1.63542E-41	84.91395269
TRIM2	-2.02879063	5.540767741	-14.71284395	3.34942E-42	1.9549E-41	84.76348547
BTC	-1.506911042	3.463166375	-14.70793	3.53424E-42	2.05591E-41	84.70619238
HSD3B7	1.932858033	7.116650859	14.70686871	3.57548E-42	2.07645E-41	84.69012031
CD3E	2.302237709	5.503622638	14.7028986	3.73403E-42	2.16673E-41	84.65032334
ACADSB	-1.81851215	5.872763166	-14.69767669	3.9533E-42	2.29206E-41	84.59767963
LIMD2	1.553567188	4.764251426	14.6923599	4.18974E-42	2.42513E-41	84.53090798
AOAH	1.944357616	5.089640285	14.69101127	4.25192E-42	2.45909E-41	84.51359419
IFI16	1.578897688	6.861370477	14.68219986	4.68148E-42	2.70082E-41	84.42133294
FAM151A	-3.064531058	4.190954851	-14.66087794	5.90849E-42	3.38637E-41	84.19892093
CDH3	-2.190133424	2.807929028	-14.66207186	5.832E-42	3.34802E-41	84.1948387
PEG3	-1.535320994	2.906615725	-14.62559048	8.68224E-42	4.94775E-41	83.81085375
AFM	-2.47889328	2.724783365	-14.62682735	8.56596E-42	4.88546E-41	83.8065615
PPP1R3C	1.82766265	7.175088021	14.62323602	8.90796E-42	5.07224E-41	83.78063732
LINC00472	-1.627488236	3.481249172	-14.61612854	9.62544E-42	5.47188E-41	83.70635627
TTYH3	2.012473295	7.797238832	14.61282198	9.97858E-42	5.65884E-41	83.66819328
SCIN	-2.713376938	4.704807854	-14.60649895	1.06903E-41	6.05262E-41	83.60984995

AC009084.1	1.676361124	3.335232344	14.6305398	8.22619E-42	4.70702E-41	83.58901132
PALM	-1.588125549	5.220509073	-14.57819525	1.45492E-41	8.19771E-41	83.2980922
LOX	3.338170765	7.372241145	14.57342931	1.53238E-41	8.61334E-41	83.25664245
CLEC7A	1.698112795	4.403291482	14.57375949	1.52689E-41	8.59625E-41	83.17973478
TENT5B	-1.779061568	3.24332988	-14.55310874	1.9115E-41	1.07099E-40	83.02139875
OLFM4	-2.095775918	2.256189227	-14.54117039	2.17645E-41	1.21652E-40	82.88673518
RRM2	1.524956687	3.984293627	14.53601239	2.30196E-41	1.28565E-40	82.71613766
SLC14A1	-2.131881164	3.739585034	-14.50234391	3.31848E-41	1.83869E-40	82.46999451
AL731533.2	1.653569084	4.648952097	14.49471026	3.60519E-41	1.9944E-40	82.36743778
CHAC1	-1.555471397	3.261337767	-14.48857617	3.85337E-41	2.12833E-40	82.32815036
LHFPL2	1.742915172	5.362895098	14.48353141	4.07019E-41	2.24277E-40	82.28565319
MYEOV	2.474078733	4.044969114	14.50480979	3.23081E-41	1.79154E-40	82.22755445
CXCL9	2.956496307	6.509935951	14.47345223	4.54049E-41	2.49798E-40	82.17886516
TOP2A	1.669803282	4.32509021	14.46647875	4.8972E-41	2.69E-40	82.00478982
CYP17A1	-2.721042861	2.915866681	-14.46032683	5.23501E-41	2.87105E-40	82.00222726
PLEK	2.069552145	5.801673497	14.44822125	5.96906E-41	3.26595E-40	81.90622283
BCL2A1	1.648409999	4.328305608	14.4522565	5.71363E-41	3.13109E-40	81.86313584
EIF4EBP1	1.77138613	7.55007003	14.44062508	6.48118E-41	3.53785E-40	81.80572179
CD8B	1.860193855	4.078518641	14.45065445	5.81371E-41	3.18344E-40	81.7591408
OLFML2A	2.374949425	6.74374197	14.43345253	7.00487E-41	3.80886E-40	81.73949652
UCHL1	-3.055141177	4.942097756	-14.42465206	7.70538E-41	4.18326E-40	81.6420019
PNMA2	2.586303262	6.03247154	14.42289074	7.85375E-41	4.26051E-40	81.63045415
AC009084.2	1.649879286	3.270439882	14.43537819	6.86026E-41	3.73895E-40	81.46750471
CD8A	2.486533595	5.193291561	14.40603892	9.42547E-41	5.10127E-40	81.38557527
CD27	2.192704333	4.701762613	14.40568279	9.46187E-41	5.11701E-40	81.34333439
PYCARD	1.681376644	5.606089823	14.38611917	1.16921E-40	6.30367E-40	81.23667389
ARHGAP30	1.793830575	5.366507708	14.38238281	1.21742E-40	6.55854E-40	81.19433093
ANXA4	1.735252896	8.64516808	14.38137597	1.23075E-40	6.62523E-40	81.1744901
SGK2	-1.718707199	4.928769281	-14.36818615	1.41937E-40	7.61132E-40	81.0296591
PLIN2	2.823222495	9.57398619	14.33430351	2.04668E-40	1.0925E-39	80.66656684
BICDL1	-2.092708967	5.52971955	-14.31737385	2.45698E-40	1.30753E-39	80.48754376
OVOL1	-1.688094315	2.976117011	-14.3123281	2.59444E-40	1.37963E-39	80.4267576
ABTB2	-1.703062263	4.39441933	-14.2775992	3.7727E-40	1.99708E-39	80.05346177
THSD7A	-1.822487882	4.375235086	-14.26725875	4.21727E-40	2.22232E-39	79.94306366
DOCK2	1.561226906	4.145559822	14.27481271	3.88768E-40	2.05328E-39	79.93241069
TPD52L1	-1.622345781	4.003168182	-14.25388628	4.87046E-40	2.56267E-39	79.7987541
DUSP15	-1.939938868	3.231571306	-14.23781644	5.79009E-40	3.03969E-39	79.62356352
LZTS1	1.968897529	5.369299694	14.23109915	6.22403E-40	3.2626E-39	79.56544614
HLA-J	1.910806995	5.031833585	14.22889925	6.37307E-40	3.33823E-39	79.52522077
BX547991.1	-1.862433236	2.448665948	-14.22597337	6.57683E-40	3.44238E-39	79.49652478
KCNE3	1.96882239	6.912559373	14.2165435	7.27876E-40	3.79558E-39	79.4004122
FOXJ1	-1.835294262	2.370693749	-14.15850994	1.3576E-39	6.98053E-39	78.7745603
HIGD1A	-1.571141978	7.051334103	-14.1451084	1.56751E-39	8.04799E-39	78.63295908
ABLIM3	2.239912973	6.375707625	14.13486627	1.74947E-39	8.96909E-39	78.54112715
ARHGAP24	-1.600727603	5.913466793	-14.13366876	1.77208E-39	9.07169E-39	78.52076001
C5orf38	-1.824551364	2.607809954	-14.13274405	1.78973E-39	9.15538E-39	78.50179049
DLL4	2.136247067	6.546137711	14.12608005	1.92226E-39	9.81177E-39	78.44237592
ITGAM	1.602469857	4.706848521	14.12177313	2.01306E-39	1.02678E-38	78.37483358
GAL3ST1	2.606048591	7.672625718	14.11917463	2.0699E-39	1.05423E-38	78.35770147
CXCL11	1.965907211	4.257761254	14.11275886	2.21719E-39	1.12843E-38	78.14813806
ACKR3	2.07332975	7.561741218	14.08009027	3.14562E-39	1.59054E-38	77.94039466
HPGD	-2.173724544	3.90672274	-14.06952773	3.52194E-39	1.77825E-38	77.82854354
ALDH1A2	-2.247021164	3.387494714	-14.06715308	3.61254E-39	1.82268E-38	77.79753478
CTSS	1.894925789	7.59181878	14.05791971	3.98744E-39	2.00894E-38	77.70501607
CCL18	2.598918714	4.448205853	14.07823489	3.20869E-39	1.62126E-38	77.69718561
ANXA3	-2.184892748	3.758933956	-14.04317846	4.66795E-39	2.34841E-38	77.54667995
CDK18	2.147209009	7.80161184	14.03491477	5.09885E-39	2.55967E-38	77.45991341
CD53	1.775783945	7.334571922	14.02731441	5.53006E-39	2.77216E-38	77.37921374

HCST	1.741922427	5.545327784	14.01281315	6.45616E-39	3.22026E-38	77.2447879
SORD	-1.60544242	4.605641968	-14.01438679	6.34861E-39	3.16887E-38	77.24416607
ALDOB	-4.919967124	6.636959314	-14.00916688	6.71239E-39	3.34567E-38	77.15660815
HOGA1	-2.046800351	5.135955955	-13.98836514	8.38036E-39	4.16518E-38	76.9746089
CXCL10	2.78027174	6.729469975	13.97701141	9.45889E-39	4.68791E-38	76.86395744
MRO	-1.669936972	3.251149149	-13.97207241	9.97023E-39	4.93435E-38	76.79562497
GAS5	1.509094793	7.91177029	13.95702402	1.17043E-38	5.77622E-38	76.63844398
TIMP1	2.1101179	10.23671233	13.9522512	1.23148E-38	6.06465E-38	76.57820159
DIRAS3	-1.661864353	3.367270063	-13.90686992	1.99606E-38	9.70029E-38	76.10468592
PDGFRA	-2.235796076	3.375175596	-13.90606655	2.01318E-38	9.77672E-38	76.08774487
P4HA1	1.63085466	8.152283865	13.89357657	2.29904E-38	1.11418E-37	75.9674089
SLC34A1	-3.198286031	3.179376763	-13.86638799	3.06889E-38	1.47602E-37	75.66284212
BIRC7	2.407416564	4.023878052	13.88074637	2.63484E-38	1.27075E-37	75.55617786
PAQR5	-1.839520885	5.666339429	-13.83772616	4.15982E-38	1.98978E-37	75.38037235
ADAMDEC1	1.625652898	3.425669228	13.86247306	3.19913E-38	1.53549E-37	75.37382161
GACAT2	1.592182933	3.324374759	13.86256899	3.19587E-38	1.53498E-37	75.36207461
IRF7	1.508555991	5.811220507	13.82523855	4.74878E-38	2.2684E-37	75.25388543
HMGCS2	-3.882389319	5.451772854	-13.79367226	6.63485E-38	3.15212E-37	74.89985682
PPARGC1A	-2.060183028	4.680052066	-13.78137094	7.55767E-38	3.57114E-37	74.78332971
OCLN	-1.797737659	3.431852435	-13.75789454	9.68837E-38	4.55334E-37	74.52992014
LINC01358	1.77428451	3.519114617	13.7688908	8.62462E-38	4.06705E-37	74.37968925
FA2H	-1.796870333	2.848260181	-13.73186046	1.27571E-37	5.97153E-37	74.25747569
PLAT	-1.785740289	5.727120693	-13.70225157	1.7439E-37	8.13587E-37	73.95422896
NDNF	-3.323682552	3.590454043	-13.70092852	1.76842E-37	8.23927E-37	73.93217361
AL049629.1	-1.547418445	2.489605346	-13.68583219	2.07369E-37	9.64875E-37	73.77187309
MOXD1	-2.314885848	4.035725599	-13.66792602	2.50453E-37	1.16148E-36	73.58721794
TCIM	-2.149240061	7.227917585	-13.66705525	2.52762E-37	1.17141E-36	73.56580087
GGACT	-1.722201979	4.089412729	-13.64447942	3.20618E-37	1.47805E-36	73.34025256
ATG16L2	1.502444053	4.235650911	13.63311389	3.61367E-37	1.66371E-36	73.15971991
FPR3	2.188472876	5.998706746	13.6198599	4.15444E-37	1.90766E-36	73.10154945
PROCR	1.554192064	7.216587555	13.61105337	4.55761E-37	2.08731E-36	72.98951034
AC073115.2	1.722599358	3.28405918	13.63063286	3.70926E-37	1.7066E-36	72.90305246
ARNT2	-1.930761166	4.945272718	-13.5949895	5.39603E-37	2.45843E-36	72.82833186
MIR1270	1.879937009	4.101845416	13.59972132	5.13425E-37	2.34221E-36	72.71315553
SMPDL3A	1.796298006	7.819054978	13.57958464	6.34396E-37	2.87347E-36	72.66244936
AC073115.1	1.640960075	3.257518884	13.60231629	4.9961E-37	2.28216E-36	72.61082209
MAN1A1	-1.512247832	7.175901372	-13.56732695	7.21535E-37	3.26182E-36	72.53027796
MYH14	-2.091995312	4.062541774	-13.55142103	8.52611E-37	3.83949E-36	72.36757889
SORT1	-1.521099691	6.539322698	-13.51461528	1.25408E-36	5.61847E-36	71.98777306
FABP5	1.526935326	4.778457927	13.49423931	1.55236E-36	6.93704E-36	71.77077027
PACRG	-1.889155194	3.031629108	-13.48134456	1.77663E-36	7.89387E-36	71.63465403
TOX3	-2.237723427	3.39279319	-13.46361615	2.13856E-36	9.47191E-36	71.44552779
C3AR1	1.877013403	5.94456777	13.46065854	2.20571E-36	9.76318E-36	71.43858947
STAB1	1.649858086	6.253840652	13.45613697	2.31246E-36	1.02099E-35	71.38208107
OGDHL	-2.64147093	6.12339116	-13.42626739	3.1592E-36	1.38609E-35	71.06090529
FYB2	-1.718620718	2.571164914	-13.42187414	3.30745E-36	1.44841E-35	71.01818228
PHLDA3	1.521204577	6.577585327	13.42009484	3.36945E-36	1.47371E-35	71.0008672
PALM3	-1.971864405	4.748008328	-13.41668498	3.49151E-36	1.52424E-35	70.96973286
TUBAL3	-1.545530703	2.174818474	-13.41684177	3.4858E-36	1.5227E-35	70.95143815
AC011352.3	1.932120065	3.496721302	13.44244869	2.66803E-36	1.17575E-35	70.94337488
CD74	1.625493306	12.73136599	13.41452513	3.5711E-36	1.55801E-35	70.90109906
SNORD99	1.989765353	4.396705252	13.41346507	3.61081E-36	1.57436E-35	70.8173662
SMIM22	-1.983917977	2.630540666	-13.39903951	4.19715E-36	1.8232E-35	70.77799673
CADM4	-2.081555886	4.924483612	-13.38389186	4.91513E-36	2.1206E-35	70.63179727
PTHLH	2.646450028	4.481542547	13.39625328	4.32088E-36	1.87229E-35	70.51750283
NCF2	1.593224384	5.232758723	13.34156594	7.63734E-36	3.25494E-35	70.20358207
AL031429.2	-2.123450523	3.705476753	-13.33577752	8.11135E-36	3.45065E-35	70.12340681
CCND1	2.094152586	9.688868549	13.33472151	8.20093E-36	3.48664E-35	70.11749818

PTGER3	-2.8025122	5.267811299	-13.32958588	8.65086E-36	3.67124E-35	70.06894471
TSPYL5	-1.559562819	4.878653309	-13.31672473	9.88842E-36	4.18881E-35	69.93149534
BARX2	2.197117624	5.9343729	13.30399806	1.12865E-35	4.77525E-35	69.81690441
PLAU	-1.929956038	6.552180952	-13.294901	1.24048E-35	5.23893E-35	69.70329878
HLA-DQA1	2.065173892	7.474685972	13.27046351	1.59862E-35	6.69887E-35	69.44994463
AFAP1L2	-1.597573885	5.161447244	-13.26340447	1.72006E-35	7.19483E-35	69.38315793
LINC01187	-2.964011672	2.525619906	-13.25737096	1.83111E-35	7.64564E-35	69.29366514
HPCAL1	1.653393426	7.561232022	13.25188166	1.93833E-35	8.08853E-35	69.26014324
SEZ6L2	2.29774466	6.440078799	13.24671163	2.04504E-35	8.51855E-35	69.22209256
PDIA5	1.55228128	6.595720105	13.23914956	2.21173E-35	9.18011E-35	69.12935535
VAT1L	-2.071217131	2.868284618	-13.22717059	2.5039E-35	1.03438E-34	68.9985116
LCP1	1.882861955	7.288461811	13.22039175	2.68596E-35	1.10893E-34	68.93399526
PSAT1	-2.695718447	5.010008135	-13.21838538	2.74233E-35	1.13087E-34	68.92426777
PTPRO	-1.762852604	3.632269496	-13.20910833	3.01869E-35	1.24263E-34	68.81880307
WNT7B	-1.76758714	2.234169119	-13.20298503	3.21612E-35	1.32313E-34	68.74899134
IL4I1	1.743138904	5.013493509	13.19291552	3.56914E-35	1.46578E-34	68.65549171
GPM6B	-1.515296142	3.156186732	-13.18776148	3.7645E-35	1.5451E-34	68.60429578
PNMA8A	-2.052525027	4.534499061	-13.15966312	5.03261E-35	2.05354E-34	68.31482304
UGT8	-1.848007778	5.503262303	-13.13633674	6.40256E-35	2.60041E-34	68.07961651
AC011297.1	-1.503279757	2.313153314	-13.11771689	7.7579E-35	3.14723E-34	67.87088132
KCNJ13	-2.036013014	2.499629421	-13.11631599	7.87073E-35	3.19116E-34	67.86097492
RNASE6	1.706602137	6.668584399	13.08636428	1.07155E-34	4.31455E-34	67.56090305
AP005233.2	1.883787468	3.441050656	13.1097044	8.42575E-35	3.41223E-34	67.50494481
AL355102.4	-1.730410797	2.540375972	-13.08001638	1.1439E-34	4.60058E-34	67.49319295
OSTM1-AS1	1.658795999	3.150167449	13.10209991	9.11245E-35	3.68394E-34	67.4144879
TGFB1	1.523643238	7.849475821	13.07163882	1.24689E-34	5.00614E-34	67.41209264
AC073486.1	3.289173407	7.412250012	13.05908357	1.41876E-34	5.68318E-34	67.29437967
GZMK	2.414154755	5.198113494	13.05662367	1.45511E-34	5.82209E-34	67.21581103
NPNT	-2.082553893	5.802559997	-13.05070939	1.54632E-34	6.17647E-34	67.20098793
HES4	1.844629721	5.389552955	13.04819806	1.58676E-34	6.32714E-34	67.18492878
GRB14	-1.803884699	4.529558473	-13.04304614	1.67303E-34	6.65978E-34	67.11818202
TEX11	1.511747165	3.312642162	13.06848386	1.28802E-34	5.16537E-34	67.10854038
LAG3	1.656772101	3.858164519	13.05745042	1.44279E-34	5.77611E-34	67.08457027
LRRC25	1.709062136	5.215448357	13.0200847	2.11799E-34	8.39282E-34	66.89617088
AC084880.1	1.889097695	5.147352556	13.01676273	2.19146E-34	8.67903E-34	66.85179147
TBX3	-1.537914735	3.903399678	-13.00921847	2.36787E-34	9.35123E-34	66.771847
LINC01230	-2.00452666	2.241204048	-13.00182965	2.55435E-34	1.00706E-33	66.68979316
NT5DC3	1.6702183	4.756692086	13.00162645	2.55968E-34	1.00859E-33	66.67993567
CXCL13	1.879788048	3.75762503	13.00607522	2.44548E-34	9.65228E-34	66.49965651
STMN3	1.895551583	7.062762323	12.97072542	3.5136E-34	1.37749E-33	66.37786756
HLA-G	2.762313041	6.031288143	12.96649647	3.66914E-34	1.43445E-33	66.34554559
SLC44A4	-2.573124429	5.197245519	-12.95933434	3.94836E-34	1.54103E-33	66.27109711
MS4A4A	1.830917257	5.616274043	12.95705737	4.04149E-34	1.57387E-33	66.25823268
C21orf62	-1.990952362	3.322793135	-12.9577817	4.01163E-34	1.56398E-33	66.24185901
MYH10	-1.83786997	5.971185256	-12.91884619	5.97446E-34	2.31118E-33	65.85719037
ARG2	-2.623458706	5.215011082	-12.90948486	6.57422E-34	2.53619E-33	65.7637301
FBP1	-2.175795397	7.177001631	-12.90364335	6.97848E-34	2.68326E-33	65.68486857
SLC2A3	1.989911683	6.646265687	12.89897683	7.31913E-34	2.80961E-33	65.65475271
ADAMTS16	-1.689105799	2.806964216	-12.89564724	7.57225E-34	2.9036E-33	65.61539461
OXCT1	-1.990954768	5.71851801	-12.89259926	7.81161E-34	2.9921E-33	65.59146615
COL6A2	1.938696737	8.633552929	12.88939023	8.07175E-34	3.08667E-33	65.55723635
CYGB	1.766662502	6.281986605	12.88148178	8.75027E-34	3.34249E-33	65.48004333
CTXN3	-2.698908781	2.819204678	-12.84807499	1.23016E-33	4.67354E-33	65.11368502
BIRC3	2.034493704	6.756019206	12.84301343	1.29526E-33	4.91021E-33	65.085987
MNDA	1.763375931	5.442713487	12.83901539	1.3491E-33	5.10601E-33	65.0589306
MISP	-2.054187797	3.342729181	-12.82687833	1.52657E-33	5.76523E-33	64.91310126
SAMHD1	1.510611971	7.063571693	12.79498608	2.11162E-33	7.91916E-33	64.59367565
EGFR-AS1	2.009671099	3.755957534	12.8172911	1.68304E-33	6.33903E-33	64.55482685

MAP3K7CL	1.657220636	5.180474926	12.7876067	2.27609E-33	8.51315E-33	64.53547298
AP006284.1	1.78887148	3.944739656	12.79300049	2.15468E-33	8.07197E-33	64.39523516
LINC01235	1.834856955	4.063039123	12.77287309	2.64359E-33	9.86132E-33	64.20005926
DIRAS2	2.12935315	4.993147541	12.72251111	4.40622E-33	1.62459E-32	63.82682081
ANO5	-1.82385452	2.753788202	-12.71203286	4.89966E-33	1.80462E-32	63.75685
LRRC19	-2.31493433	4.691004306	-12.69433805	5.86092E-33	2.14962E-32	63.58769323
C6orf223	2.567860839	5.688599444	12.66457007	7.91969E-33	2.88807E-32	63.28035053
ASS1	-1.993884843	8.183989702	-12.66226553	8.1063E-33	2.95458E-32	63.23599955
FAM193B	1.62265544	5.362833644	12.64183216	9.9645E-33	3.61304E-32	63.0715171
PCSK6	2.159761502	4.963677709	12.64453309	9.69644E-33	3.51766E-32	63.03592848
SERPINA4	-2.001910448	2.549004198	-12.60601505	1.43011E-32	5.15872E-32	62.68843108
NRP2	1.562822012	5.092520949	12.59741643	1.55956E-32	5.61988E-32	62.62203164
MFAP3L	-1.67105189	5.182430834	-12.58869666	1.70274E-32	6.12638E-32	62.52522914
ALOX5	1.99323942	5.772934405	12.55645662	2.35538E-32	8.41395E-32	62.21678318
PLXND1	1.517878855	7.221347119	12.54629953	2.60862E-32	9.2996E-32	62.09489475
TMEM45A	2.099623339	4.844941898	12.53673439	2.87182E-32	1.02223E-31	61.94199284
COX4I2	2.165439062	6.107156454	12.51531344	3.56107E-32	1.26371E-31	61.80520535
CA2	-1.690119544	8.352931063	-12.5110975	3.71498E-32	1.31766E-31	61.72328947
SLC2A1	1.893967582	8.184155785	12.50290846	4.0331E-32	1.42905E-31	61.66565799
CYS1	-2.487894272	7.435618039	-12.50138127	4.09536E-32	1.45037E-31	61.62962677
CD200	1.515189646	6.274046203	12.48924072	4.62557E-32	1.63566E-31	61.5312728
AMBP	-1.764457059	3.240457831	-12.4718218	5.50774E-32	1.93977E-31	61.35271609
SLFN11	1.542299032	5.783845803	12.46531048	5.87889E-32	2.06736E-31	61.30301429
MCHR1	1.914070932	3.611690634	12.48172642	4.98742E-32	1.76095E-31	61.17359892
ADTRP	-1.544200927	3.01033317	-12.43594246	7.88729E-32	2.75563E-31	61.00096288
SEMA6A	1.750100499	5.790822618	12.42947691	8.41402E-32	2.93526E-31	60.94965878
AL353801.1	-1.789108436	3.435985594	-12.42831771	8.51209E-32	2.96799E-31	60.91909623
AC073218.1	2.471982304	5.795318306	12.42164497	9.09915E-32	3.16953E-31	60.86527368
PSORS1C3	2.469171515	5.603872623	12.4165431	9.57503E-32	3.32867E-31	60.80300743
KCNE4	1.794559356	5.559885525	12.39316626	1.20925E-31	4.18513E-31	60.59066386
GPRC5A	-2.130320081	3.605658003	-12.39059173	1.24072E-31	4.28769E-31	60.54177699
AXL	1.548246979	6.989620583	12.38496528	1.31236E-31	4.52633E-31	60.48847336
AC093496.1	-1.651776428	2.449107715	-12.38275844	1.34157E-31	4.62252E-31	60.46546562
TFAP2A	-1.778984525	2.700291329	-12.37684825	1.42303E-31	4.88874E-31	60.40900925
KIAA0895L	1.561123233	4.97461093	12.36758518	1.56071E-31	5.35385E-31	60.32854666
COL4A1	1.861308378	9.665346076	12.36657367	1.57653E-31	5.40281E-31	60.30756334
IRF6	-2.058061669	4.927719771	-12.36111052	1.66474E-31	5.70233E-31	60.26064663
AC022144.1	1.746956039	4.83721497	12.35740544	1.72735E-31	5.91099E-31	60.20706101
GRB10	1.510611308	6.658940722	12.34927879	1.87299E-31	6.39686E-31	60.13613391
MSR1	1.819802241	5.329441481	12.3313937	2.23799E-31	7.61373E-31	59.97460447
RHOBTB3	-1.796226322	4.922636463	-12.27606655	3.87824E-31	1.3048E-30	59.41771902
AL080317.1	1.940687101	5.3723792	12.25411451	4.82166E-31	1.60906E-30	59.20986029
NTRK2	-1.910819798	3.735499306	-12.25127646	4.95923E-31	1.65261E-30	59.16671411
SEMA3B	-1.634375727	4.765947777	-12.2402297	5.53294E-31	1.84203E-30	59.06215771
USP2	-1.996409379	5.066569621	-12.23896683	5.60259E-31	1.86256E-30	59.05505037
SCD5	-2.428623776	5.422211663	-12.23330065	5.92602E-31	1.96821E-30	58.99960166
CD14	1.638435015	8.215784603	12.2318578	6.0113E-31	1.99465E-30	58.98319241
NMB	1.915415778	6.578485177	12.2304881	6.09339E-31	2.02093E-30	58.97032824
CSF1R	1.675707491	6.814184623	12.22674247	6.32361E-31	2.0953E-30	58.92701839
SLC5A3	-1.880243871	5.884346162	-12.2064381	7.73079E-31	2.55189E-30	58.73482697
SLC22A7	-2.201069759	3.051908687	-12.16948559	1.1138E-30	3.63543E-30	58.35584792
ADAMTS4	1.968580073	5.272932226	12.15597496	1.27267E-30	4.14432E-30	58.23853962
EXOC3L4	1.568762119	4.395723865	12.14963427	1.35481E-30	4.40362E-30	58.12781576
CHRDL1	-2.437656442	3.210084557	-12.14051282	1.48232E-30	4.80689E-30	58.06949964
PTPRC	1.812152516	5.68807185	12.12976064	1.64803E-30	5.33931E-30	57.99490773
COL5A2	1.823932873	6.240048882	12.10184511	2.1694E-30	6.99283E-30	57.71340896
HLA-DQB2	2.381633413	5.930023707	12.09939975	2.22223E-30	7.15982E-30	57.69662142
CTSL3P	1.626888365	3.847143117	12.11211353	1.96087E-30	6.33232E-30	57.63810788

LGI4	2.744989275	5.496071238	12.08117661	2.65837E-30	8.54538E-30	57.46010493
HLA-DRB1	1.586895989	11.5991383	12.0742366	2.84601E-30	9.13597E-30	57.40148856
HLA-DPA1	1.762049801	9.11613018	12.05743646	3.3566E-30	1.07405E-29	57.27422887
PCSK1N	-2.63154923	4.65793901	-12.05475426	3.44616E-30	1.09969E-29	57.25242577
MUC1	-2.096068932	6.647771811	-12.0221173	4.74637E-30	1.50841E-29	56.920483
ACAN	1.702696612	4.299569388	12.00918597	5.38744E-30	1.70826E-29	56.70748751
HLA-DRA	1.655543225	12.41176696	11.99128184	6.41953E-30	2.02725E-29	56.58479051
RIMKLA	1.545868777	4.365119905	11.97317408	7.66336E-30	2.40917E-29	56.39608713
SLC29A4	1.741684706	5.327675965	11.94914091	9.6916E-30	3.03453E-29	56.2324891
SUCNR1	-1.98156208	4.606986523	-11.9346068	1.11688E-29	3.48458E-29	56.07826809
GPR4	1.736618891	6.523759559	11.91790583	1.31445E-29	4.09186E-29	55.91661462
FBLN5	-2.110011118	6.080578588	-11.88873012	1.74652E-29	5.39862E-29	55.63342186
CTHRC1	2.347164474	5.559229249	11.86757199	2.14571E-29	6.60319E-29	55.4278719
GPR27	-1.517741142	3.231810013	-11.86125954	2.28151E-29	7.01494E-29	55.36963799
SOD3	-1.660537973	6.72850394	-11.85870895	2.33877E-29	7.1784E-29	55.34062224
KCNMA1	1.539838677	4.157131509	11.86226317	2.25936E-29	6.94989E-29	55.28361991
QRFPR	2.010300952	4.890671713	11.8557493	2.40702E-29	7.37816E-29	55.27521557
HHLA2	2.79673976	5.947383413	11.84015633	2.80065E-29	8.55103E-29	55.16625819
RTP4	1.503013414	5.836007252	11.83356371	2.98576E-29	9.09239E-29	55.11085799
AGR2	-2.1114206	2.406652573	-11.83451526	2.95831E-29	9.02451E-29	55.09968493
ALDOC	2.14576913	7.080305864	11.82976924	3.09776E-29	9.42937E-29	55.0624899
WLS	-1.557675703	6.74558516	-11.82713961	3.17782E-29	9.66467E-29	55.03712822
PGGHG	2.444589838	6.03913021	11.79733562	4.24253E-29	1.28137E-28	54.76781646
CDH4	1.878510002	3.928047798	11.81258122	3.65978E-29	1.10919E-28	54.68025742
KLK1	-2.943473488	2.714808623	-11.79195619	4.46947E-29	1.34584E-28	54.67451564
PRSS8	-1.96156055	6.261233768	-11.7658008	5.757E-29	1.72169E-28	54.44809883
UNC5B	1.67230532	6.461128869	11.76437051	5.83719E-29	1.74418E-28	54.43580332
ABCB1	-2.046890698	5.34361737	-11.76066925	6.04989E-29	1.80465E-28	54.4048939
CP	3.452865981	7.032081563	11.74490611	7.04555E-29	2.09223E-28	54.26576059
MAPK8IP3	1.576131083	5.134398227	11.74174239	7.26421E-29	2.15311E-28	54.23079988
PRSS35	-1.569323149	3.148862233	-11.73638992	7.64962E-29	2.26064E-28	54.16659021
CXorf36	1.677735606	5.773894225	11.70392696	1.04634E-28	3.07917E-28	53.86939395
CYP3A5	1.667580038	4.585253393	11.70124479	1.07374E-28	3.15451E-28	53.79546202
COL8A1	1.908758443	5.822590889	11.69466397	1.14405E-28	3.35542E-28	53.78274778
LYZ	2.436075638	8.613661357	11.67808591	1.34211E-28	3.92645E-28	53.60624576
MYC	1.692445004	7.412117419	11.67131914	1.43243E-28	4.18196E-28	53.53906995
MT1F	-2.081027963	5.798042302	-11.6614765	1.57471E-28	4.58776E-28	53.45268675
VWCE	1.501510104	3.830115607	11.66437588	1.5314E-28	4.4653E-28	53.32074461
C16orf74	1.543764559	3.989302529	11.65602647	1.65945E-28	4.83263E-28	53.26927712
KITLG	-1.645783683	5.490742331	-11.64095413	1.91814E-28	5.56285E-28	53.25746295
ALDH4A1	-1.90125805	6.512260962	-11.62148051	2.31258E-28	6.69289E-28	53.06474145
IL20RB	2.139368277	3.964964268	11.64807975	1.79119E-28	5.2098E-28	53.05983189
ENAM	-1.589407607	3.347493147	-11.60467011	2.71729E-28	7.82857E-28	52.90686232
CYP39A1	-1.502410763	3.431609121	-11.60355862	2.74641E-28	7.90594E-28	52.89778473
BMP6	-1.611860846	4.574178984	-11.60130946	2.80628E-28	8.07497E-28	52.87304806
FAM107A	-1.653897649	5.351986092	-11.58119776	3.40272E-28	9.74713E-28	52.68741417
DAO	-2.010927963	3.341620826	-11.56972295	3.79785E-28	1.08523E-27	52.56721642
CRABP1	-2.001649124	2.35368281	-11.5579815	4.24941E-28	1.20884E-27	52.4549796
SEL1L3	1.552440588	6.826536285	11.55244189	4.4806E-28	1.27254E-27	52.40633345
HLA-DQB1-AS1	1.592661475	4.51154401	11.51851559	6.19577E-28	1.74619E-27	52.04983635
GUCA2B	1.580164701	3.248344241	11.53017038	5.54331E-28	1.56672E-27	51.89777458
C2orf40	-1.89427548	3.920410648	-11.46437735	1.03794E-27	2.88233E-27	51.57220413
AC087379.2	-2.139941331	2.967024271	-11.46342026	1.04743E-27	2.90755E-27	51.5555181
P2RY8	1.564238614	5.316617908	11.41509214	1.65798E-27	4.55715E-27	51.12815356
TPSAB1	1.693440461	4.587943323	11.38459191	2.214E-27	6.0451E-27	50.7855667
COL1A1	2.564039742	8.20804227	11.37684604	2.38255E-27	6.49932E-27	50.74617459
NTN4	-1.77439914	7.454796468	-11.37374956	2.45344E-27	6.68581E-27	50.70874529
CDH1	-1.697719439	6.796188025	-11.36965531	2.5504E-27	6.93924E-27	50.67997031

DPT	-1.895941498	3.591869311	-11.36864481	2.57492E-27	7.00321E-27	50.66898221
ANGPTL3	-2.284634066	3.684658077	-11.34684023	3.16478E-27	8.56423E-27	50.46279985
SPINK13	1.829898515	3.869198803	11.36685114	2.619E-27	7.12035E-27	50.43057731
AC093583.1	-1.810325587	3.694097909	-11.33436575	3.56076E-27	9.6135E-27	50.34835613
PLOD2	1.53073188	7.678252846	11.33303031	3.60597E-27	9.73179E-27	50.33906279
AHSA2P	1.524577311	4.85519282	11.33021153	3.70327E-27	9.98669E-27	50.31704517
LINC02061	-1.898928082	2.831286902	-11.32968948	3.72157E-27	1.00283E-26	50.30212364
CDKN1C	-1.520120054	5.865956416	-11.32046514	4.06025E-27	1.09157E-26	50.22753668
PXDN	1.603485186	6.008792621	11.30135931	4.86226E-27	1.30219E-26	50.05213322
GPR183	1.560240302	5.557467972	11.30025032	4.91337E-27	1.31487E-26	50.04911673
OSMR	1.507318384	7.098117609	11.30085755	4.88532E-27	1.30786E-26	50.03412337
AC092535.4	1.631404953	4.217900494	11.28695176	5.56951E-27	1.48591E-26	49.8086486
ASPDH	-1.732538752	3.728995005	-11.25936184	7.22143E-27	1.9135E-26	49.64751872
ST14	-1.785628915	6.186223942	-11.25502922	7.5218E-27	1.99158E-26	49.61269524
KIT	-1.776215582	4.541330236	-11.24362348	8.37323E-27	2.20783E-26	49.50258569
CHST13	1.718783139	4.852817268	11.23368396	9.1929E-27	2.42214E-26	49.40309142
NECTIN4	-1.520068944	2.567568021	-11.2116984	1.13002E-26	2.96286E-26	49.20293142
CREB3L3	2.137978277	4.659148344	11.22039107	1.0415E-26	2.73487E-26	49.1996612
HRH2	2.026594854	4.642322598	11.19642496	1.30403E-26	3.39494E-26	48.97943245
WDR72	-1.980017583	5.423158901	-11.16668121	1.72291E-26	4.45727E-26	48.79340014
IL2RG	1.610425614	5.961207579	11.1614775	1.80886E-26	4.67445E-26	48.7492977
CDH6	1.994534072	6.864632439	11.15622352	1.89997E-26	4.90627E-26	48.69022792
GLIS1	1.513580123	4.536379278	11.15819276	1.8653E-26	4.81852E-26	48.68695581
FBLN1	-1.768751309	4.863192879	-11.15399763	1.93993E-26	5.00576E-26	48.67081176
PMEPA1	1.579780928	6.966952915	11.14945609	2.02407E-26	5.21326E-26	48.62309639
AC078864.1	1.998814469	5.185379343	11.14767356	2.05808E-26	5.29306E-26	48.60737886
AC022509.2	1.686978443	4.615366691	11.12278014	2.59679E-26	6.6541E-26	48.34579767
COX7A2P1	-1.640592736	3.005457439	-11.09046844	3.50982E-26	8.94129E-26	48.08087019
SLC34A3	-1.691952709	2.928360639	-11.08576891	3.66687E-26	9.33118E-26	48.03680683
OR2I1P	2.671123758	6.438516966	11.07368459	4.1035E-26	1.04044E-25	47.94425555
CDHR1	1.609215846	3.598686076	11.09058709	3.50594E-26	8.93466E-26	47.84360673
SLC17A4	2.524169217	5.145789985	11.07103015	4.20612E-26	1.06531E-25	47.83890409
SEMA3G	-1.805109936	5.207092048	-11.04262059	5.47761E-26	1.37886E-25	47.64362327
FREM2	-1.543168802	3.718534832	-11.04071467	5.57545E-26	1.40147E-25	47.62099858
UBD	2.367384792	6.341482339	11.00538287	7.73846E-26	1.92993E-25	47.31296877
SLC28A1	2.327227718	7.463382308	11.00634424	7.6698E-26	1.91349E-25	47.3017092
MYO15B	1.596275589	5.71286595	11.00014207	8.12358E-26	2.02309E-25	47.26335477
IL1RL1	-1.900111849	3.463364301	-10.9958314	8.45458E-26	2.10178E-25	47.2030407
HAVCR2	2.030016659	6.504850141	10.98461539	9.37995E-26	2.32934E-25	47.11315625
MMP9	2.143183418	4.771315938	10.98050254	9.74392E-26	2.41772E-25	46.98742608
NEBL	-1.553442829	5.024595538	-10.90656844	1.92863E-25	4.71085E-25	46.39084995
PIGR	-3.441873201	6.13910544	-10.90485199	1.95938E-25	4.78427E-25	46.35774546
TRAC	1.685571094	6.369027386	10.8839561	2.37506E-25	5.78713E-25	46.18745508
SLCO2B1	1.573704965	5.839442467	10.88090584	2.44265E-25	5.9477E-25	46.16839402
SNORD104	1.769070042	4.959213537	10.87208182	2.64916E-25	6.43709E-25	46.07170358
C10orf99	2.297561538	4.063610141	10.89276993	2.18999E-25	5.34178E-25	45.9908968
CCL4L2	1.753655228	4.604739002	10.86441461	2.84261E-25	6.88564E-25	45.95999805
CD36	2.014498319	6.059764882	10.84598228	3.36703E-25	8.1306E-25	45.8532087
GJA1	1.715963546	8.130576636	10.81966321	4.28642E-25	1.02762E-24	45.59964739
PCDH17	1.628093758	5.034080239	10.80339763	4.97516E-25	1.1903E-24	45.45799901
RGS5	2.267783626	10.37973196	10.7719463	6.63365E-25	1.57953E-24	45.15572184
KCNJ16	-1.788135926	7.488293754	-10.76449532	7.10096E-25	1.68793E-24	45.08331959
KSR1	1.578166235	5.715113763	10.74921145	8.16431E-25	1.93412E-24	44.97365527
LINC01874	-1.98785884	2.634324258	-10.73600058	9.20991E-25	2.17446E-24	44.83181633
DCDC2	-1.891882681	5.444427523	-10.70837883	1.18452E-24	2.7807E-24	44.59580816
PIPOX	-1.925234924	4.392114868	-10.64934655	2.02524E-24	4.69292E-24	44.05712366
CD248	1.610966327	7.071847374	10.63460366	2.3148E-24	5.34797E-24	43.92096448
EPS8L1	-1.551397344	3.336360157	-10.62297147	2.57199E-24	5.92064E-24	43.82261611

C4B	1.884066337	5.693028343	10.60948096	2.90596E-24	6.68286E-24	43.71754029
CNTN3	-1.565243143	2.456479523	-10.59101228	3.43403E-24	7.86623E-24	43.52825166
SPATA18	1.516652942	5.736318668	10.58320754	3.68487E-24	8.42428E-24	43.47775274
PCK1	-3.010416238	6.056642488	-10.57011967	4.14698E-24	9.45295E-24	43.33671102
SFTA2	-1.669085873	2.38815667	-10.56474879	4.35287E-24	9.90936E-24	43.29207182
WT1	-1.789705066	3.003989643	-10.5553008	4.73998E-24	1.07591E-23	43.21137544
SLC39A14	1.596193855	7.809618047	10.55414146	4.78977E-24	1.08686E-23	43.20413798
AC087482.1	2.141963797	5.869161766	10.55128472	4.91471E-24	1.11341E-23	43.19654566
MIR140	1.883623201	4.460466345	10.55605459	4.70788E-24	1.06897E-23	43.13714988
GBP1	1.561086513	6.623929801	10.52819991	6.05042E-24	1.36495E-23	42.97022598
KCCAT333	2.901285929	5.097522138	10.54568118	5.16923E-24	1.16955E-23	42.9613344
ETNK2	-1.532166734	5.357421593	-10.50166884	7.68047E-24	1.71993E-23	42.73909106
SLC6A8	1.512524162	8.527536293	10.47327477	9.90986E-24	2.20506E-23	42.48852962
C2	1.910976057	5.048608299	10.4681191	1.03787E-23	2.30352E-23	42.43001628
ANO1	1.550091106	5.538642281	10.4620766	1.09563E-23	2.43017E-23	42.40080108
CHST15	1.510343217	6.287283335	10.45908345	1.12541E-23	2.49385E-23	42.35906356
PROC	-1.676766004	3.163864145	-10.40144316	1.88432E-23	4.11179E-23	41.84524803
RF00154	1.618248886	4.507264076	10.37842675	2.31368E-23	5.02831E-23	41.59897837
BDKRB2	-1.633435505	4.219643042	-10.37250713	2.43899E-23	5.29571E-23	41.58760668
APOD	-1.655298573	4.400186677	-10.35861539	2.76014E-23	5.97816E-23	41.46546707
NGF	1.549730491	5.15182473	10.33241143	3.4844E-23	7.50733E-23	41.25219972
G6PC	-2.355639349	3.661958324	-10.32730243	3.64618E-23	7.8366E-23	41.18490036
RGCC	1.549646228	8.69742713	10.31084069	4.21988E-23	9.02804E-23	41.05190063
PLVAP	1.75020362	10.62467389	10.29775291	4.73921E-23	1.01174E-22	40.91200975
RERGL	-1.516311043	3.779877544	-10.27628779	5.73165E-23	1.21693E-22	40.74284901
PHGDH	-1.748598789	5.066042532	-10.26278919	6.45872E-23	1.36881E-22	40.62786733
LARGE2	-1.575882772	4.386621252	-10.22558042	8.97168E-23	1.88145E-22	40.29674945
PLG	-2.702803777	3.375460975	-10.22408557	9.09076E-23	1.90585E-22	40.27384222
TGFBI	2.790775785	8.518856567	10.22164314	9.28872E-23	1.9456E-22	40.26000268
FLT1	1.738550105	7.682371038	10.20541802	1.07168E-22	2.23269E-22	40.12083881
TPSB2	1.666349709	4.601967232	10.19220172	1.20394E-22	2.49782E-22	39.97556403
DSP	-1.873308315	5.829788288	-10.17983954	1.34226E-22	2.7774E-22	39.9053927
LAMC2	-1.806860384	3.970306027	-10.16637926	1.51085E-22	3.11518E-22	39.77929454
ALPK2	1.713207756	5.518521477	10.15302383	1.69886E-22	3.49152E-22	39.68460307
KRT223P	2.039292576	4.167607362	10.14896785	1.76043E-22	3.61592E-22	39.43131285
AC021744.1	2.390279522	5.255972911	10.11218241	2.43016E-22	4.94799E-22	39.28517656
LINC01426	1.581034077	4.111458888	10.11483375	2.37441E-22	4.84152E-22	39.204757
PTGDS	-2.129118573	5.064392319	-10.08472839	3.0896E-22	6.26153E-22	39.08025998
PAH	-2.501391774	3.888517367	-9.988644611	7.13281E-22	1.41725E-21	38.24250112
C4A	1.883730798	5.732684196	9.984354051	7.40337E-22	1.46975E-21	38.22621983
CYBB	1.727555383	6.487520567	9.985827519	7.30932E-22	1.45191E-21	38.22296056
GPR34	1.561466895	5.429688753	9.957937898	9.30827E-22	1.83543E-21	37.99946443
SLC38A4	-1.73537781	2.918926096	-9.955512597	9.50582E-22	1.87227E-21	37.9581049
CPVL	-1.509753438	7.115053383	-9.926753489	1.21904E-21	2.38426E-21	37.70865078
F2RL3	1.866332632	5.270166243	9.923833866	1.25019E-21	2.44312E-21	37.69887743
SCGN	2.870221167	5.658539605	9.90291591	1.4976E-21	2.91036E-21	37.47699301
TEK	-1.567178256	5.762995824	-9.886754635	1.72149E-21	3.33066E-21	37.3781441
ADH6	-1.544952728	3.61117076	-9.872878439	1.94E-21	3.74514E-21	37.25436214
LINC02384	1.587558099	4.251052905	9.783483517	4.17737E-21	7.88596E-21	36.41557877
SLPI	-3.228110594	5.559656115	-9.773776963	4.53881E-21	8.53604E-21	36.40655433
SLC2A5	1.963588852	6.101881894	9.736085595	6.26098E-21	1.16838E-20	36.10860703
ADSSL1	1.837776737	5.757089489	9.677608151	1.02949E-20	1.9024E-20	35.61909112
F3	-1.547318172	4.156854336	-9.61885856	1.69305E-20	3.10075E-20	35.10618403
C1QL1	2.526239469	5.586502556	9.579541054	2.35901E-20	4.27677E-20	34.77329185
C1orf210	-1.836087715	5.241254237	-9.57709938	2.40803E-20	4.36112E-20	34.76509157
TUBA3D	1.691001697	3.544966739	9.604802579	1.90643E-20	3.48333E-20	34.71311999
FN1	1.651394372	8.850883478	9.557950133	2.82916E-20	5.10139E-20	34.60516502
CLDN7	-1.61433771	6.233912334	-9.52517621	3.72582E-20	6.68375E-20	34.33220655

ACTG2	1.726901012	4.962765434	9.519658072	3.90233E-20	6.98963E-20	34.2778539
RRAD	1.605710123	7.341544884	9.501826953	4.5313E-20	8.07899E-20	34.13073153
FSTL3	1.56869799	7.247814818	9.490151108	4.99656E-20	8.88813E-20	34.03370904
WFDC2	-2.11418327	7.21585883	-9.48957443	5.02073E-20	8.92885E-20	34.01795951
CYP1B1	-1.701813813	5.459393232	-9.473989557	5.71959E-20	1.01485E-19	33.90921722
CKMT2	-1.832669864	3.699517157	-9.465365332	6.1469E-20	1.08901E-19	33.8283727
APOLD1	1.821115008	7.249212205	9.44155811	7.49769E-20	1.32363E-19	33.63157868
MELTF	-1.500595442	3.02956067	-9.417077258	9.19339E-20	1.61241E-19	33.43764317
ARL4C	1.535696836	6.699702938	9.411411303	9.63709E-20	1.68896E-19	33.38431501
PDGFD	1.563111106	6.782497912	9.406149849	1.00681E-19	1.76317E-19	33.34061105
CA4	-1.814384845	4.312428012	-9.397320731	1.08346E-19	1.89599E-19	33.26972468
GJB1	-1.626886046	5.465363634	-9.370307167	1.35575E-19	2.35598E-19	33.05476887
CD163	1.714990384	6.117265471	9.352211196	1.57503E-19	2.73027E-19	32.91122317
AC136475.3	2.106985667	4.859446144	9.335256685	1.81221E-19	3.12669E-19	32.69939929
UGT3A1	-2.057733276	4.365393695	-9.318822209	2.07579E-19	3.57001E-19	32.62851229
VCAN	1.876773781	6.55932277	9.311052182	2.21329E-19	3.80089E-19	32.56839878
KCNK3	2.041931251	5.663849128	9.280582458	2.84518E-19	4.86098E-19	32.33371171
PROM1	-2.532155611	4.77273394	-9.272173571	3.04905E-19	5.19787E-19	32.2558376
SORCS2	-1.65754093	3.81291419	-9.225605475	4.46935E-19	7.55471E-19	31.86728222
HLA-DRB6	1.514194359	7.359680895	9.200436192	5.49227E-19	9.23251E-19	31.6630443
NGFR	1.546668263	5.237853259	9.111200422	1.13669E-18	1.87696E-18	30.96434793
LY6E	1.567421149	8.866301337	9.05681476	1.76623E-18	2.89125E-18	30.51523802
APOL1	1.532239903	8.516415895	9.054266305	1.803E-18	2.95006E-18	30.49605144
AC104031.1	1.526875457	4.769701518	9.038189797	2.05306E-18	3.34824E-18	30.35938813
LHFPL3-AS2	-1.611283636	4.411015713	-8.992101496	2.9764E-18	4.80925E-18	29.99261083
C19orf33	1.836374201	7.240663119	8.938452684	4.57791E-18	7.31087E-18	29.56509146
CYP4F3	-1.508391245	2.975331652	-8.910963341	5.7036E-18	9.07957E-18	29.35577618
MIR647	1.550170715	4.798912213	8.903386594	6.05937E-18	9.63058E-18	29.29137477
CPA3	1.569006551	4.891850885	8.881479871	7.21626E-18	1.14356E-17	29.12295014
SPON2	1.592132509	5.78155846	8.861317639	8.47287E-18	1.33814E-17	28.97570237
AC007406.2	1.670391326	5.103531177	8.852876187	9.06118E-18	1.42816E-17	28.90598533
CCL20	2.127456785	5.183547083	8.818659453	1.1889E-17	1.8617E-17	28.61080429
APCDD1L	-1.518953059	2.836719197	-8.782729932	1.57994E-17	2.46026E-17	28.34732704
BTNL9	1.643268873	5.597254665	8.762310141	1.85634E-17	2.87786E-17	28.20424189
CCDC8	-1.640169136	4.792511252	-8.702986572	2.96056E-17	4.54841E-17	27.72482697
SERPINE1	1.934185404	8.347353573	8.696348961	3.11882E-17	4.78735E-17	27.67524589
CLRN3	1.952609943	7.17414206	8.696816186	3.10741E-17	4.77088E-17	27.67351536
SPINK1	-1.793892902	3.828105461	-8.654274409	4.33561E-17	6.6188E-17	27.34467172
RHEX	1.514683351	6.176519308	8.652237561	4.40517E-17	6.71913E-17	27.33710216
TSPAN1	-1.948368126	8.046265372	-8.653299852	4.36875E-17	6.66649E-17	27.31626648
FLRT3	-1.75985392	5.119843967	-8.601729168	6.53089E-17	9.8775E-17	26.94725624
LUM	-2.287298799	6.358450773	-8.598581376	6.69276E-17	1.01136E-16	26.91399626
MUC20	-1.560298558	4.921558903	-8.583713093	7.51254E-17	1.13181E-16	26.80545031
COL5A1	1.559676049	5.487483104	8.577486367	7.88466E-17	1.18609E-16	26.77619471
APOH	-1.612670446	3.028711113	-8.523314164	1.19939E-16	1.78962E-16	26.34563025
SLC9A3-AS1	1.819319863	5.365020492	8.427342509	2.50921E-16	3.68045E-16	25.63028878
POSTN	1.733055659	6.333784358	8.329156011	5.30429E-16	7.66298E-16	24.88205299
CAPN6	-1.901735912	4.265001765	-8.321131196	5.63726E-16	8.13392E-16	24.81443947
VCAM1	1.694396321	8.718965453	8.283030194	7.52203E-16	1.07889E-15	24.53697452
KL	-1.61088915	6.616278563	-8.173348254	1.71577E-15	2.41443E-15	23.71847148
HAVCR1	2.090722273	6.370123514	8.149074427	2.05691E-15	2.88289E-15	23.55181065
AC015722.2	1.519272505	3.816445694	8.162711483	1.85778E-15	2.60849E-15	23.45921221
CYP24A1	-1.684874514	3.285742802	-8.116219009	2.6274E-15	3.66193E-15	23.29749691
LINC02294	-1.658237693	3.423360473	-8.093566331	3.10905E-15	4.32118E-15	23.13157499
APLNR	1.535153364	6.408186309	8.065897679	3.81681E-15	5.28284E-15	22.93066507
RNU1-14P	1.799785688	5.202384811	8.048786536	4.33179E-15	5.97435E-15	22.81561048
LCN2	-1.653704442	2.826332295	-8.031600137	4.91794E-15	6.76277E-15	22.68010681
CSDC2	-1.722163943	3.907331558	-8.020438877	5.33983E-15	7.32996E-15	22.59585685

NAPSA	-1.735467645	4.863822332	-8.007915527	5.85583E-15	8.02881E-15	22.50949106
COL1A2	1.511541387	8.227557415	7.941471734	9.53481E-15	1.29487E-14	22.03242125
SLC22A6	-2.485343399	4.879411518	-7.893632708	1.35174E-14	1.82475E-14	21.69209937
KCNJ15	-1.531132691	6.276622547	-7.819376844	2.31608E-14	3.09193E-14	21.15827161
CPN2	-1.767694314	2.824656626	-7.783715336	2.99532E-14	3.98276E-14	20.89785408
LIX1	-1.589240173	3.05815191	-7.655708787	7.48213E-14	9.7979E-14	19.99898812
MT1X	-1.532795132	6.830112629	-7.534812895	1.75673E-13	2.26485E-13	19.15750873
AC111000.2	1.526866395	5.126427486	7.52136066	1.93046E-13	2.48425E-13	19.08268892
HLA-DQA2	1.949099657	6.685327412	7.466001084	2.84174E-13	3.64021E-13	18.68877983
TMEM174	-2.011138234	4.898665301	-7.398660405	4.5343E-13	5.74734E-13	18.2312102
IGF2	-1.854456743	4.727432348	-7.353633315	6.18546E-13	7.78663E-13	17.92272566
SLC16A12	-1.744522176	6.565594156	-7.299536612	8.96442E-13	1.12123E-12	17.55564711
BEX1	-1.666818074	3.552960962	-7.273066138	1.07405E-12	1.33979E-12	17.37712594
CES3	1.610209209	5.597813591	7.110784258	3.2156E-12	3.93405E-12	16.32053594
HAO2	-1.908162175	5.563104821	-7.100303544	3.44921E-12	4.20955E-12	16.2399824
SLC6A19	-2.156480573	4.487141152	-7.040055549	5.15339E-12	6.24366E-12	15.84148326
MFAP4	-1.657997104	5.96616368	-6.746533696	3.50124E-11	4.10895E-11	13.96618597
IGHA1	1.981168651	9.263113712	6.708701768	4.46023E-11	5.21254E-11	13.7264491
LTF	-1.775785177	4.623853298	-6.676116372	5.48936E-11	6.39709E-11	13.51943675
SLC22A12	-2.066031895	5.222650378	-6.66386989	5.93355E-11	6.90783E-11	13.45036926
IGLC2	1.907751175	7.941298228	6.661988936	6.00482E-11	6.98732E-11	13.43002763
IGLC3	1.916529079	7.190002873	6.623172121	7.67799E-11	8.90611E-11	13.18683163
IGHA2	1.740902067	6.550008537	6.49934195	1.66853E-10	1.91196E-10	12.43304406
IGHM	1.768247728	6.855579561	6.48048434	1.87588E-10	2.14816E-10	12.31418844
REG1A	2.11785392	5.416563489	6.382310421	3.43603E-10	3.8984E-10	11.73133634
MMP7	-1.784030076	6.929208596	-6.379481101	3.49609E-10	3.96526E-10	11.70096252
IGKC	1.915497054	8.623791877	6.262609588	7.11252E-10	7.99958E-10	11.01823928
SLC17A3	1.951188536	7.294636858	6.257887284	7.31789E-10	8.22661E-10	10.98162758
DES	-1.50530766	4.081473071	-6.238964152	8.20065E-10	9.20419E-10	10.87269584
PRIMA1	1.550403776	4.644292895	6.238197502	8.23852E-10	9.24522E-10	10.85113329
STAC2	-1.633849734	3.351424938	-6.187425192	1.11668E-09	1.24653E-09	10.57307887
IGHG3	1.747181035	6.600210461	6.100964953	1.86536E-09	2.06776E-09	10.07368054
CDHR5	1.518078171	7.287713978	5.926087584	5.16917E-09	5.66988E-09	9.07550093
FGG	1.514608661	3.613893222	5.820813198	9.43331E-09	1.03019E-08	8.257492876
IGHG1	1.84011679	7.981775892	5.733143874	1.54597E-08	1.6781E-08	8.012049178
IGLV1-40	1.633219393	6.359382994	5.560355468	4.0175E-08	4.31999E-08	7.088141495
IGHG2	1.605498656	8.06279881	5.429890141	8.12783E-08	8.68773E-08	6.403598616
IGLV3-19	1.542132288	6.095433354	5.164504577	3.26086E-07	3.44289E-07	5.063414355
RBP4	-1.810558447	5.947750173	-5.139149397	3.71216E-07	3.91525E-07	4.936103783
CYP4A11	-1.664780567	5.477092537	-5.013831836	6.98852E-07	7.33768E-07	4.325488548
