# Supplementary material for: The Diagnostic and Prognostic Values of HOXA Gene Family in Kidney Clear Cell Renal Cell Carcinoma
Source: J Oncol. 2022 Mar 16;2022:1762637. doi: 10.1155/2022/1762637 (PMC8942704; doi:10.1155/2022/1762637)
Supplement: Supplementary Materials — Supplementary Figure 1. Flow chart of data collection and subsequent model establishment in our study; Supplementary Figure 2. The TNM stage and clinical grade measured by the time-dependent receiver-operating characteristic curves at 1, 3, and 5 years. Supplementary Figure 3. The Kaplan–Meier survival curves of evaluating single prognostic roles of the prognosis-related HOXA genes. Supplementary Figure 4. A more detailed machine learning model construction process for early screening classification. Supplementary Figure 5. Custom scripts for prognostic model and machine learning model construction in this study; Table S1: differentially expressed genes between KIRC samples and paired normal samples, including the differential expression HOXA genes; Table S2: the training set, the test set, and the external validation set for machine learning model; Table S3: small molecule drugs by querying the CMAP and DGIdb databases; and Table S4: a detailed summary of the different roles of HOXA family members in KIRC. [file 1762637.f1.zip › 1762637.f1/Supplementary Table S2 (2).pdf]

The Discovery set

Id	age	grade	stage	M	T	MIR324	XIST	MIR210	MIR25	GABPB1.AS1	LINC00894	SNHG7	FGD5.AS1	MIR22HG
TCGA-3Z-A93Z-01A-11R-A37O-07	1	2	1	1	0	0.612994625	0.017067153	3.225795816	2.570791284	0.743989965	0.135492261	3.899170055	4.145603507	3.200293686
TCGA-A3-3306-01A-01R-0864-07	1	3	1	1	0	0.88577595	0.005145434	0.281897603	2.60562447	0.977179296	0.391317923	1.441174126	4.360450706	2.776280333
TCGA-A3-3307-01A-01R-0864-07	1	3	1	1	2	0.484714312	0.004847677	1.011797781	2.466104779	1.182134566	0.580251633	2.640142256	4.704912301	4.30062154
TCGA-A3-3311-01A-02R-1325-07	0	2	1	1	0	0.181635462	0.003259461	0.348477879	1.281629773	0.506645685	0.337764557	2.349022868	4.28629044	2.498248126
TCGA-A3-3316-01A-01R-0864-07	0	3	1	1	1	0.318189979	0.003995546	1.173933342	2.569322713	2.13836557	0.600056476	1.418402138	4.331072939	4.034135136
TCGA-A3-3322-01A-02R-1325-07	0	2	1	1	0	1.436572454	0.016813401	2.882044796	3.506785875	2.038529096	0.642447871	4.203742106	4.108666845	2.77547061
TCGA-A3-3324-01A-02R-1325-07	0	3	1	1	0	1.070503483	0.005563705	1.872002034	2.994166784	1.757966131	0.407530454	2.236170111	4.159969709	2.709265411
TCGA-A3-3325-01A-01R-0864-07	0	2	1	1	0	1.564626783	0.003337968	2.986442091	3.427029174	2.192708363	0.780208414	2.899234695	4.544274306	3.884063664
TCGA-A3-3326-01A-01R-0864-07	0	0	1	1	0	0.811778007	0.003058685	2.489194856	3.401639195	2.050318401	0.744755886	3.096730379	4.010027102	2.35283203
TCGA-A3-3328-01A-01R-0864-07	1	2	1	1	0	0.162490348	0.001931713	1.556875941	1.888406987	1.081957349	1.142674252	2.90086614	6.051657241	5.001901716
TCGA-A3-3329-01A-01R-0864-07	1	2	1	1	0	0.715568508	0	4.221315867	3.049190828	0.795570848	0.628501044	3.795103959	4.493731857	3.973617836
TCGA-A3-3331-01A-02R-1325-07	1	2	1	1	0	0.839902193	4.276446755	1.524404398	2.173381601	1.431317191	0.651998986	2.413291504	4.423857058	3.901148586
TCGA-A3-3335-01A-01R-0864-07	0	4	1	1	1	1.074695993	0.01005463	1.349812207	1.074695993	1.661221394	0.664864528	2.900347554	3.597536036	1.900514427
TCGA-A3-3343-01A-01R-0864-07	1	3	1	1	1	0.372642314	0.002387353	2.498569602	2.514892678	1.300531513	0.54020439	3.63085585	4.336509501	2.818106371
TCGA-A3-3346-01A-01R-1766-07	1	3	1	1	0	0.374345468	0.003598101	2.384858827	1.834918089	1.139656113	0.44858756	1.70672591	3.948594154	1.870165575
TCGA-A3-3347-01A-02R-1325-07	1	2	1	1	0	1.147750238	4.422461737	2.289887409	3.222819834	1.44294522	0.592229977	1.512641097	4.88114337	3.498082763
TCGA-A3-3349-01A-01R-1188-07	0	2	1	1	0	0.761124026	2.296947881	2.002391016	3.223071373	1.347489107	0.683590811	3.515640566	4.618519963	2.590553847
TCGA-A3-3351-01A-02R-1325-07	0	2	1	1	1	0.632657476	0.022140609	3.56497564	3.219932327	2.162229568	1.356109531	3.033614183	4.461891409	2.748192051
TCGA-A3-3352-01A-01R-0864-07	1	3	1	1	2	1.201110179	0.004852942	2.370684618	2.721679819	1.01271579	0.745199779	3.101184112	4.270679917	2.302202596
TCGA-A3-3359-01A-01R-0864-07	1	2	1	1	0	1.111658238	3.904797427	2.868736082	2.034158266	1.008416597	0.846844204	2.585563961	4.410656634	3.691176217
TCGA-A3-3362-01A-02R-1325-07	0	2	1	1	0	0.827033403	3.339759142	2.65986119	1.732095958	1.026468089	0.773591872	2.80026231	4.375406672	3.19277564
TCGA-A3-3363-01A-01R-0864-07	0	2	1	1	1	0.484500009	0.10701751	0.858151766	3.01705373	0.862262497	0.889887091	2.354737178	5.431375747	2.647157491
TCGA-A3-3365-01A-01R-0864-07	0	2	1	1	0	0.230286292	0.008393958	0.234151056	2.758926466	1.724728195	0.505873413	2.491012747	5.237677123	4.749241757
TCGA-A3-3367-01A-02R-1420-07	1	3	1	1	0	0.548657118	0.002810756	0.436537679	2.531437994	0.878063741	0.594756831	3.816034373	4.201271172	1.949998431
TCGA-A3-3370-01A-02R-1420-07	0	2	1	1	0	0.629053046	4.546922515	2.291428992	2.708930724	1.028402557	0.90254606	2.981932338	4.372988402	3.003469631
TCGA-A3-3372-01A-02R-1325-07	0	2	1	1	2	0.884609178	0.018180215	1.782972359	2.511333662	2.348491201	0.775702778	2.57859596	4.62067451	2.735664437
TCGA-A3-3373-01A-02R-1420-07	0	3	1	1	0	0.742059926	2.927208034	1.312498555	0.67611675	0.925156268	0.254105705	1.655224791	4.558682345	3.400664357
TCGA-A3-3374-01A-02R-1325-07	0	2	1	1	0	0.508855161	0.126832068	0	1.617115936	0.305919126	0.402856725	2.687029058	5.361132257	3.76615494
TCGA-A3-3378-01A-02R-1325-07	0	3	1	1	0	0.511484573	0.010312119	1.107916357	2.138857283	1.450944361	0.740549762	1.37725409	4.354650317	2.58262656
TCGA-A3-3380-01A-01R-0864-07	0	2	1	1	0	1.061215601	0.010970501	2.796421778	2.910758375	1.711536749	0.866219181	4.074754043	4.476725339	2.876421668
TCGA-A3-3382-01A-02R-1325-07	1	3	1	1	0	0.712210919	0.005165583	2.193630532	2.206988983	0.936181763	0.768781454	3.302832174	4.475608227	2.003859915
TCGA-A3-3383-01A-02R-1325-07	0	2	1	1	0	1.149579478	0.001645461	1.961564829	3.007227286	0.626830268	0.388862309	4.127562061	3.180763726	2.228591483
TCGA-A3-3385-01A-02R-1420-07	0	2	1	1	0	0.90433916	4.279462467	1.536979135	2.410592353	1.528644054	0.484645215	3.517049678	4.730541686	1.171061287
TCGA-A3-3387-01A-01R-1541-07	0	2	1	1	0	0.615831135	0.002156652	2.088388553	2.850360742	2.169902981	0.629481015	3.102031087	4.644681465	2.953302186
TCGA-A3-A6NI-01A-11R-A33J-07	0	3	1	1	0	1.210963064	0.004730081	4.105569525	2.774859591	1.349823322	0.42396146	3.815907235	3.950723858	2.844287077
TCGA-A3-A6NJ-01A-12R-A33J-07	0	0	1	1	0	1.21795398	3.547577122	2.383944423	1.778756997	0.721113738	0.32483724	2.829070612	4.403901691	3.901135882
TCGA-A3-A6NL-01A-11R-A33J-07	0	2	1	1	0	1.50357892	3.929955013	3.175675357	3.102076308	0.865595169	0.649801673	3.529893052	4.360605598	3.000273959
TCGA-A3-A6NN-01A-12R-A33J-07	1	2	1	1	0	0.981925585	0	2.450942053	2.681948747	1.465168943	0.464652728	4.295234184	4.669329602	2.459078033
TCGA-A3-A8CQ-01A-11R-A37O-07	0	2	1	1	0	0.95113009	3.876517783	4.526587607	2.164993148	0.794009572	0.548859763	3.047193208	4.527130089	3.588335653
TCGA-A3-A8OU-01A-11R-A37O-07	1	0	1	1	0	1.514209502	3.405251202	4.224883002	3.255620916	0.757857562	0.416633262	2.558184581	4.064363515	3.59352012
TCGA-A3-A8OV-01A-11R-A37O-07	1	2	1	1	0	1.404647628	0.029380211	4.28187242	3.290842008	0.827462637	0.464017561	4.23546378	3.83694755	2.192335485
TCGA-A3-A8OW-01A-11R-A37O-07	0	2	1	1	2	0.889628532	0.015128743	3.69835146	2.967462321	1.269593769	0.514957706	3.89084499	4.530803159	2.89929886
TCGA-A3-A8OX-01A-11R-A37O-07	0	3	1	1	0	0.358478593	2.596949273	2.327924363	2.307144123	0.61231732	0.271844341	2.701348244	4.908668274	2.883582397
TCGA-AK-3425-01A-02R-1277-07	1	2	1	1	0	0	0.001459974	0.793949979	1.287721889	0.235576875	0.01830093	2.662608811	4.239021898	2.38511619
TCGA-AK-3426-01A-02R-1325-07	0	3	1	1	2	0.325617157	0.020382451	1.939544308	1.749836251	1.196951275	1.100080549	2.293652318	4.165988796	2.546842407
TCGA-AK-3427-01A-01R-0864-07	0	1	1	1	0	0.206772216	0.007476537	0	0.692795624	0.266573354	0.142382823	4.524214376	5.809809463	4.705517541
TCGA-AK-3428-01A-02R-1277-07	0	2	1	1	2	0	0.397446676	0	0	0.365209649	0.084870754	3.187808406	4.388389656	2.99483367
TCGA-AK-3431-01A-02R-1277-07	0	3	1	1	1	0	1.61042399	0	0.898695337	0.39724553	0.106420988	2.936669268	4.825936593	2.839975092
TCGA-AK-3433-01A-02R-1277-07	0	1	1	1	1	0.277383815	0.670901299	0	1.574644339	0.632346323	0.517865829	4.145648793	4.790146186	3.053034715
TCGA-AK-3440-01A-02R-1277-07	0	3	1	1	0	0.885093069	0.009125397	1.197502139	1.527052271	0.250435039	0.698253384	2.966838366	5.403758456	5.072280319
TCGA-AK-3443-01A-02R-1325-07	0	1	1	1	1	0.437682455	0.005735305	0.163872647	0.959872666	0.989237569	0.943258415	1.718124425	5.126696029	4.647945023
TCGA-AK-3445-01A-02R-1277-07	1	3	1	1	2	1.853600255	0.0388292	2.014982366	1.166565305	0.973872216	0.379581648	2.682423129	3.944784708	1.733406498
TCGA-AK-3450-01A-02R-1277-07	1	2	1	1	0	1.070985658	2.605061941	2.227403627	1.034529796	0.615546842	0.360802441	1.914092173	4.095925241	2.635652538
TCGA-AK-3451-01A-02R-1188-07	0	3	1	1	1	0	0	0	0.342313661	0.155642906	0.040485916	3.323832946	3.522415464	2.563563571
TCGA-AK-3453-01A-02R-1277-07	0	2	1	1	1	0	0.540606529	3.130792606	3.006190963	0.89374321	1.031066832	2.753161622	3.606085964	2.843675363
TCGA-AK-3454-01A-02R-1277-07	1	3	1	1	0	2.122224466	0.559238926	1.648069191	3.047230441	1.453238127	0.665947353	2.839815997	4.091693369	2.973711644

TCGA-AK-3455-01A-01R-0864-07	1	3	1	1	2	0.239181111	1.028320044	1.303815105	1.423852472	1.126102092	0.955355464	3.162362947	4.149272586	2.597510204
TCGA-AK-3456-01A-02R-1325-07	0	3	1	1	1	0	0.001524229	0	1.002351855	0.832869067	0.337119156	1.629813577	5.139685931	1.633486492
TCGA-AK-3458-01A-01R-1503-07	0	3	1	1	0	0.26267265	0.055579584	2.742704936	1.872889883	1.330085026	0.864455168	4.449753522	4.290539994	1.868527923
TCGA-AK-3465-01A-02R-1325-07	1	1	1	1	0	0.49516194	1.410903263	0.874874112	2.156339112	1.210052124	0.828701961	3.747043489	5.685847879	3.973491545
TCGA-AS-3778-01A-01R-A32Z-07	0	0	1	1	0	1.676032457	0.003555393	3.691770793	2.404189837	1.482318566	0.646331805	4.087508707	4.201578997	3.125043269
TCGA-B0-4691-01A-01R-1277-07	0	3	1	2	1	1.066457636	0.049823588	2.60724704	2.116019731	0.60289818	0.638374526	2.592864693	3.918399011	3.298125705
TCGA-B0-4693-01A-01R-1277-07	1	4	1	1	2	1.622325633	4.049374919	1.958338539	1.592501564	1.235114774	0.750907614	2.268722514	4.148516032	4.025199305
TCGA-B0-4697-01A-01R-1277-07	0	4	1	2	2	2.586790188	3.692786743	2.191896546	2.323682719	1.928915014	1.415523562	3.164178955	4.174945746	2.354286995
TCGA-B0-4698-01A-01R-1503-07	1	4	1	1	3	1.006196411	0.012213433	1.107898882	1.422778864	0.952628053	0.969412088	3.84822797	5.374507206	2.541883068
TCGA-B0-4699-01A-01R-1277-07	1	4	1	2	3	0.999814464	0.011741149	1.463740342	1.72353147	1.229260155	1.023990302	2.95931446	5.465216616	3.606612567
TCGA-B0-4700-11A-01R-1541-07	0	4	1	2	3	1.173711779	0.002906147	0.242057741	1.419296926	0.949420613	0.670035038	1.169083084	5.040116929	3.575573458
TCGA-B0-4700-01A-02R-1541-07	0	4	1	2	3	0.866784453	0.436786838	1.59099184	1.069100728	0.657875384	0.23988225	1.558085998	4.143126492	2.752678245
TCGA-B0-4701-01A-01R-1277-07	1	3	1	2	2	1.964330139	4.229498098	2.991524391	1.899790611	1.150498432	0.908411313	3.615539194	4.157683445	3.291623428
TCGA-B0-4706-01A-01R-1503-07	0	4	1	1	2	2.010914841	0.007002252	3.358456001	2.675214597	1.635704744	1.24336188	4.610597719	4.199402512	2.041823042
TCGA-B0-4707-01A-01R-1277-07	0	4	1	1	2	0.774127771	0.401173266	2.54113108	2.806709948	1.102105564	0.765734008	3.553266347	3.852998454	1.841477441
TCGA-B0-4710-01A-01R-1503-07	1	3	1	1	2	2.820169397	4.251437685	2.493009276	2.497709632	1.426374544	0.810960414	2.770824401	3.920260793	3.283561272
TCGA-B0-4712-01A-01R-1503-07	1	3	1	2	2	0	0.001169909	1.304456842	1.548376689	0.361976545	0.224994942	2.247117269	4.672651177	2.620492079
TCGA-B0-4712-11A-02R-1503-07	1	3	1	2	2	0	0	0.155386172	0.200276643	0.565462146	0.069645782	2.030783144	5.828314292	3.324135643
TCGA-B0-4714-01A-01R-1277-07	1	3	1	2	2	1.605440365	0.040089504	3.277732686	4.272327069	1.541935909	1.486824642	3.135656557	4.156690426	2.395927886
TCGA-B0-4718-01A-01R-1277-07	0	2	1	1	2	1.132686837	0.152188829	4.089959659	2.679594458	1.201694561	1.358009421	3.088728869	3.899446619	3.159627533
TCGA-B0-4810-01A-01R-1503-07	0	3	1	1	2	1.160532164	0.007768842	3.071659823	1.973924375	1.426437214	0.753313251	3.087660596	4.338214081	2.802725819
TCGA-B0-4811-01A-01R-1503-07	0	3	1	1	2	2.555608744	0.093468	3.714513598	3.95808118	1.66601883	0.986358532	3.925370587	3.764393403	2.998561301
TCGA-B0-4813-01A-01R-1277-07	1	3	1	1	2	1.514656257	0.163530525	3.386174026	3.775219207	1.347031779	1.488163146	3.540314221	3.963474787	3.644244326
TCGA-B0-4814-01A-01R-1277-07	0	3	1	2	3	2.079346457	0.024567721	2.099232808	2.406412322	1.305222341	1.081228361	3.189572486	3.994275818	1.535014538
TCGA-B0-4815-01A-01R-1503-07	0	4	1	1	2	1.590689608	0.005813579	3.004227154	3.237404132	1.172236495	0.795485755	2.840031683	4.378521916	3.118788402
TCGA-B0-4816-01A-01R-1503-07	0	3	1	1	1	0.673997429	0.02203803	2.095620425	3.081245031	1.451753548	0.430908223	2.816543832	4.496634122	3.377117995
TCGA-B0-4818-01A-01R-1503-07	1	3	1	1	1	0.637534696	4.007703015	3.329460262	1.917753546	0.844135898	0.791451036	2.912239481	4.432852367	2.744235979
TCGA-B0-4819-01A-01R-1277-07	0	4	1	2	2	1.667493521	2.703846543	1.016396636	2.813025752	1.565307337	1.025061946	2.210901026	4.509835894	3.705454856
TCGA-B0-4823-01A-02R-1420-07	1	2	1	1	0	0	0.009382424	0.198839404	0.255215985	0.214015523	0.058154248	3.765151232	4.380066043	2.825007553
TCGA-B0-4824-01A-01R-1277-07	0	3	1	1	0	0.866943928	3.775280105	2.906985806	1.795511935	1.647799395	1.061501465	2.833139472	4.223823421	2.707295408
TCGA-B0-4827-01A-02R-1420-07	1	4	1	1	2	0	0.980520136	0.321651408	0	0.156198365	0.009372324	2.529545933	3.7993921	2.080482308
TCGA-B0-4828-01A-01R-1277-07	1	3	1	2	1	1.429162885	0.120095612	2.980891194	3.107364601	1.063439052	1.051038566	3.306139144	3.924602526	3.489430937
TCGA-B0-4833-01A-01R-1305-07	1	2	1	1	0	0.964749564	3.314317576	2.355685093	3.106649416	1.893215831	1.141005909	3.399903842	4.166896407	2.601474387
TCGA-B0-4834-01A-01R-1305-07	0	3	1	1	0	0.204113129	0.00491998	0	1.009480808	0.35548443	0.779110675	3.143869334	5.22106084	4.276789894
TCGA-B0-4837-01A-01R-1305-07	0	3	1	1	0	0.210872842	0.017753695	2.228278602	1.814842099	1.167160674	0.357913745	2.903620134	3.954306724	2.078351224
TCGA-B0-4838-01A-01R-1305-07	1	3	1	1	0	1.925287422	4.150307033	1.003983148	2.025897369	2.105338733	0.732762823	1.870248475	4.409084208	3.478917962
TCGA-B0-4841-01A-01R-1277-07	0	3	1	2	1	0	0.561436229	1.319916238	0.477101901	0.334159127	0.260462869	2.437644324	3.947744438	2.678679046
TCGA-B0-4842-01A-02R-1420-07	1	4	1	1	2	0	0.190241779	0.694119889	0	0.343739691	0.035739346	2.56489162	3.491095607	1.728653156
TCGA-B0-4843-01A-01R-1277-07	0	3	1	1	2	1.161884757	0.054149442	2.948596243	2.357547747	0.8779195	0.560647507	3.133009378	3.897895812	3.200457551
TCGA-B0-4844-01A-01R-1277-07	0	3	1	2	2	0.474012747	0.045014427	1.915435541	2.211125136	0.91070181	0.753208614	2.064660713	3.790513124	2.471093783
TCGA-B0-4845-01A-01R-1277-07	1	2	1	2	2	2.991031437	0.032853642	3.656862347	3.885772828	2.494066199	1.054122445	2.08516735	4.336954142	2.981578158
TCGA-B0-4846-01A-01R-1277-07	0	2	1	2	2	1.015944124	3.722828599	1.130600896	1.897602728	1.047727287	0.621084223	2.088243891	4.525813924	4.114595975
TCGA-B0-4847-01A-01R-1277-07	0	3	1	2	2	1.390787625	0.014719997	3.075995979	3.529123405	0.837220168	1.029719037	2.888110188	3.593357749	3.01725437
TCGA-B0-4849-01A-01R-1277-07	0	3	1	1	2	1.082399564	0.10481931	2.882074943	2.859633476	2.2450794	1.095083694	3.39713198	4.387835844	4.33136322
TCGA-B0-4852-01A-01R-1503-07	1	2	1	1	1	1.187315577	4.217898982	2.087895181	1.829675971	1.253102554	0.919957865	2.487846681	4.283844185	3.343007288
TCGA-B0-4945-01A-01R-1420-07	1	2	1	1	0	0.452854969	3.435050932	2.460849543	1.568833002	1.824199822	1.261694175	1.918476338	4.470468546	4.482982985
TCGA-B0-5075-01A-01R-1334-07	1	2	1	1	2	0.995866441	3.611439872	1.930585598	1.389734573	0.571600265	0.744262075	2.117810219	3.933866943	2.868418675
TCGA-B0-5077-01A-01R-1334-07	1	3	1	1	0	1.049965504	0.002889921	2.742421877	0.964611614	1.12294792	0.891711585	3.4519181	4.067125841	3.00318358
TCGA-B0-5083-01A-02R-1420-07	0	3	1	1	0	0	0.223745518	0	0.957646665	0.122580383	0.040066709	3.074975679	5.082187587	3.113185335
TCGA-B0-5085-01A-01R-1334-07	1	3	1	1	2	2.22750294	2.473830991	4.34879937	2.835663702	0.980048281	1.06730275	3.408814092	3.348912989	3.901840044
TCGA-B0-5094-01A-01R-1420-07	0	2	1	2	2	1.336093536	0.010077917	2.133244734	3.131251138	0.966622207	0.618447181	2.371015199	3.86935751	2.487027791
TCGA-B0-5096-01A-01R-1420-07	1	1	1	1	2	0.232294218	1.723822514	1.37901155	1.517770928	0.502704704	0.164381832	1.707139869	4.06439021	2.156999135
TCGA-B0-5097-01A-01R-1420-07	0	2	1	1	2	0.837234847	2.651902187	1.287864999	2.255738816	0.523004172	0.338837529	1.824842214	4.379294993	3.814015398
TCGA-B0-5099-01A-01R-1420-07	1	3	1	1	2	1.980424072	4.053738549	2.961381596	1.516362556	0.811594351	0.825199673	3.203762044	4.050432156	4.896987289
TCGA-B0-5100-01A-01R-1420-07	1	3	1	1	2	0.567206223	0.010375346	3.247991372	1.321045799	0.730692685	0.265332826	3.57097047	4.723462256	3.195315299
TCGA-B0-5104-01A-01R-1420-07	1	2	1	1	0	1.392016492	2.172302712	1.718147766	1.736625307	0.760520889	0.679912748	2.326709321	4.197511262	3.916771212
TCGA-B0-5107-01A-01R-1420-07	0	4	1	2	1	1.294209433	3.0736529	2.823677757	3.752759547	1.04597469	0.864259239	3.061860616	4.082354755	1.654829317
TCGA-B0-5108-01A-01R-1420-07	0	2	1	1	2	1.183350637	0.001144476	3.223430427	1.862440685	0.658300825	0.403486662	2.104940811	4.316039647	2.681157727

TCGA-B0-5109-01A-02R-1420-07	1	4	1	1	2	1.126253749	0.010918508	0.229046276	1.598293051	0.567014969	0.357686519	4.12751317	4.167942179	2.256319646
TCGA-B0-5110-01A-01R-1420-07	1	2	1	1	0	1.087699369	4.897164907	2.348282304	2.215363423	0.831713403	0.825140986	2.655385034	4.556577166	4.289414322
TCGA-B0-5113-01A-01R-1420-07	1	2	1	1	2	0.184049813	4.732747994	2.245083814	1.293604278	0.698442138	0.880613822	2.283526751	4.719077674	2.635983988
TCGA-B0-5115-01A-01R-1420-07	0	3	1	2	1	1.285593068	0.016227514	2.391079643	2.435447844	2.073382767	0.982171273	3.402848146	4.349762606	2.143780698
TCGA-B0-5116-01A-02R-1420-07	0	3	1	1	2	0	0.00116876	1.386981768	1.547443745	0.212765518	0.061332671	3.048180659	4.277815102	2.190593753
TCGA-B0-5117-01A-01R-1420-07	0	2	1	1	0	0	0	0.22969739	2.341464509	0.379167938	0.92656804	3.5311565	4.831691087	3.997557181
TCGA-B0-5399-01A-01R-1503-07	0	2	1	1	0	0.366771262	0.002344794	2.587450225	2.33699544	0.954517024	0.74791824	3.800965328	4.297998901	2.72772217
TCGA-B0-5400-01A-01R-1503-07	0	4	1	1	2	0.375835156	1.29220883	0.540525126	1.131169307	0.33562342	0.34050336	1.852776848	3.635310465	2.802673689
TCGA-B0-5402-11A-01R-1503-07	0	4	1	1	3	0.310316062	0.005824807	0.315383732	0.400507797	0.268983079	0.097796685	2.015589261	4.994093785	3.830134868
TCGA-B0-5402-01A-01R-1503-07	0	4	1	1	3	0.291462585	0.002719953	1.662448926	0.922594905	0.297670903	0.204987435	2.715658042	4.176100796	2.688338633
TCGA-B0-5690-11A-01R-1541-07	0	0	1	1	0	0	1.766623315	0	0.608736138	0.394897945	0.049446138	2.639152484	5.536044469	3.131323955
TCGA-B0-5690-01A-11R-1541-07	0	0	1	1	0	0.712200913	3.696337271	1.781213364	1.543448668	0.66798021	0.290777709	3.374368804	4.521160859	2.779407797
TCGA-B0-5691-11A-01R-1541-07	1	3	1	1	0	0.149044085	2.866792363	0	1.011344571	0.568030406	0.17278789	2.175675564	5.371073438	3.730660959
TCGA-B0-5692-01A-11R-1541-07	1	3	1	1	2	0.411826669	0.566402454	1.81673048	1.345806763	0.25301446	0.272311401	2.746285803	3.919397973	1.895286556
TCGA-B0-5694-11A-01R-1541-07	1	3	1	1	2	0	0.02463905	0	0.980474355	0.402346877	0.17539887	2.259693193	5.236556414	3.885627707
TCGA-B0-5694-01A-11R-1541-07	1	3	1	1	2	0.510255188	0.015396843	0.518048308	1.936847355	0.372515629	0.154110901	2.671323161	4.024779873	2.128938335
TCGA-B0-5695-01A-11R-1541-07	0	2	1	1	0	0.168086925	2.727947656	1.253358274	0.866934864	0.312296837	0.169616801	3.360650089	4.358387827	2.912738122
TCGA-B0-5696-01A-11R-1541-07	1	4	1	1	2	0.344576318	0.402882872	0.867144681	0.622514396	0.207615599	0.037427692	2.546823468	3.860024528	2.505892785
TCGA-B0-5696-11A-01R-1541-07	1	4	1	1	2	0	0.002470746	0.208249861	0.68710689	0.32258459	0.094285996	2.510427372	5.302345123	3.315073257
TCGA-B0-5697-01A-11R-1541-07	0	2	1	1	0	0.16843633	1.900800995	1.400094079	0.993288847	0.381581341	0.195066281	1.413122487	5.003658098	2.694176432
TCGA-B0-5698-01A-11R-1672-07	1	3	1	1	0	0.733887441	0.002148763	0.744308426	1.043332673	0.497302479	0.126467285	2.955150818	4.221794151	4.013217205
TCGA-B0-5699-11A-01R-1541-07	0	2	1	1	0	0	0.00140504	0.436263407	0.944688526	0.36036027	0.102608085	2.433631652	5.248253766	3.636174136
TCGA-B0-5701-01A-11R-1541-07	0	4	1	1	2	0.540155248	0.126800242	2.001985298	1.493911641	0.390532382	0.095417197	2.019793911	3.573380837	1.829868176
TCGA-B0-5702-01A-11R-1541-07	1	2	1	1	0	0	0.033291259	0	0	0.233941786	0.021077074	3.418311197	1.586879907	1.456886466
TCGA-B0-5703-01A-11R-1541-07	1	3	1	1	0	0.244938877	0	0.810342652	0.57864588	0.422453858	0.143897186	3.370726727	4.126936939	2.261991988
TCGA-B0-5703-11A-01R-1541-07	1	3	1	1	0	0	0.002900631	0.241634289	0.967219125	0.560882627	0.184464637	3.143709452	5.707230767	3.854047135
TCGA-B0-5705-11A-01R-1541-07	0	2	1	1	0	0	2.321333063	0	0.752040441	0.560760044	0.161124879	2.146998463	5.534367844	3.35524009
TCGA-B0-5706-11A-01R-1541-07	0	2	1	1	1	0.227997089	0.002773317	0.231826438	0.752604119	0.836576903	0.382127483	2.350453694	5.703982168	3.716275951
TCGA-B0-5709-11A-01R-1541-07	0	3	1	1	2	0.46182652	3.439061579	0.328946837	0.740476758	0.497327571	0.282237918	1.997151491	5.321041271	4.004283415
TCGA-B0-5709-01A-11R-1541-07	0	3	1	1	2	0	1.707775932	1.680090184	0.886795438	0.304492449	0.103592812	1.812059787	4.68839724	2.631671003
TCGA-B0-5710-01A-11R-1672-07	0	2	1	1	0	0	0.002502948	0.39464905	1.021490764	0.964127398	0.397758239	2.580674556	4.538427534	2.117028525
TCGA-B0-5711-01A-11R-1672-07	0	3	1	1	2	0.194733741	0.00234125	0.527492034	0.470269017	0.641218423	0.157289267	2.904070502	4.223472927	2.258057565
TCGA-B0-5712-11A-01R-1672-07	1	3	1	2	1	0	1.950557121	0	0.449646034	0.319229042	0.146503124	2.357652207	5.389088031	3.839707375
TCGA-B0-5713-01A-11R-1672-07	1	3	1	1	2	0.765427095	3.251340887	0.776186647	1.317919183	0.776291473	0.209392635	2.40916542	4.366729353	3.343569063
TCGA-B2-3923-01A-02R-A277-07	0	2	1	1	1	0	0.03450683	0	1.103478011	1.548529776	1.663116888	3.791518849	5.686715353	3.785409852
TCGA-B2-3924-01A-02R-A277-07	1	2	1	1	0	0.520124397	0.014008189	2.315491213	2.161123794	1.616315969	0.835331144	3.78897386	4.572716092	2.903052538
TCGA-B2-3924-01A-02R-1325-07	1	2	1	1	0	0.212423538	0.001285474	2.285819798	1.537840141	1.003791424	0.189658993	3.595680318	4.360834076	2.765812727
TCGA-B2-4098-01A-02R-1325-07	1	2	1	1	0	1.340409408	1.072679309	1.356194777	2.346517606	1.433532307	1.686062956	2.257109506	4.759818072	2.844444536
TCGA-B2-4101-01A-02R-1277-07	0	3	1	1	1	1.228412531	0.022954511	2.251159394	3.029125624	2.004393476	1.06650539	3.317117596	4.362411902	3.013995774
TCGA-B2-4102-01A-02R-1325-07	0	2	1	1	0	1.338998047	0.059523927	2.914907262	3.458697447	0.927965131	1.072337008	1.919877337	4.541779693	3.026690157
TCGA-B2-5633-01A-01R-1541-07	0	2	1	1	0	0.988024857	0.149811368	1.849401667	1.2086984	1.257832174	0.179376089	2.772020539	4.309064862	2.280514851
TCGA-B2-5633-01A-01R-A277-07	0	2	1	1	0	0	0.01353137	0.250614823	1.453673058	1.460322682	0.467643077	3.258994089	4.962467879	2.325262163
TCGA-B2-5635-01A-01R-1541-07	1	2	1	1	0	0.207629894	0.147772895	3.071656472	2.172667352	1.003693677	0.442732564	2.266163544	4.438385844	2.450152531
TCGA-B2-5635-01B-04R-A277-07	1	2	1	1	0	1.264865578	0.042036337	2.301158848	3.002745887	3.063724921	3.022618298	1.977642566	4.990697313	2.720610799
TCGA-B2-5636-01A-02R-1541-07	1	2	1	1	0	0.85384624	0.006530134	3.152364497	2.152032394	0.780663287	0.103477648	3.210602417	5.003052415	2.627151382
TCGA-B2-5636-11A-01R-1541-07	1	2	1	1	0	0	0.002257374	0.511564001	1.201902691	0.83138616	0.220770476	2.287599476	5.082231838	3.663926819
TCGA-B2-5639-01A-01R-1541-07	0	3	1	2	2	0	0	1.253794074	0.44641885	0.168806931	0.041112243	2.263026976	3.991131381	1.777390019
TCGA-B2-5641-11A-01R-1541-07	1	3	1	1	0	0	0.001435104	0.783505951	1.273131878	1.321876167	0.158431832	2.033946793	5.793452778	3.443359759
TCGA-B4-5377-01A-01R-1503-07	1	3	1	2	2	0.287122498	3.71530934	3.411553207	0.666493898	0.450808523	0.314732763	2.376244498	4.370721579	3.471054322
TCGA-B4-5378-01A-01R-1503-07	0	2	1	1	0	0.590822868	0.074950717	3.267745505	1.426006232	0.63629536	0.297015217	4.105667828	5.021696197	2.703680617
TCGA-B4-5832-01A-11R-1672-07	0	2	1	1	2	0	0	0	0	0.357274976	0.034914484	0.672766198	5.033712897	2.70964867
TCGA-B4-5834-01A-11R-1672-07	0	0	1	1	0	0	0	1.664450066	0.222991612	0.497666937	0.080978242	3.95656133	4.477941897	1.762735663
TCGA-B4-5835-01A-11R-1672-07	0	2	1	1	0	0	2.061574025	0.15549787	0.674574783	0.273114125	0.24623391	1.758184395	4.531025405	1.169507961
TCGA-B4-5843-01A-11R-1672-07	0	2	1	1	0	0.436155529	0.001430163	0.238427459	1.122107417	0.481130129	0.209535194	2.774330579	4.351432561	1.772452665
TCGA-B8-4146-01B-11R-1672-07	0	2	1	1	0	0	3.421056033	0.229619793	0.293872811	1.0468732	0.246209558	1.935810705	4.32032937	2.357493003
TCGA-B8-4148-01A-02R-1325-07	0	3	1	1	0	1.78011977	4.880701907	4.144916221	2.579492942	1.354191405	0.861137283	2.51896454	4.670320464	4.114973396
TCGA-B8-4151-01A-01R-1188-07	0	2	1	1	2	0.941433002	1.819359429	2.408978644	0.986809298	0.657440913	0.249898756	3.597374454	4.280944297	2.702664711
TCGA-B8-4153-01B-11R-1672-07	1	3	1	1	2	0.639155971	0.003009539	1.790519613	1.576246278	0.994303524	0.337192785	3.301664617	3.996185927	3.097891567

TCGA-B8-4154-01A-01R-1188-07	1	2	1	1	0	1.558557481	1.965057271	1.767849206	1.415569424	0.618675872	0.151563059	3.680472539	4.606307067	2.880596962
TCGA-B8-4619-11A-01R-1758-07	0	2	1	1	0	0	0.001855183	0.552172271	1.15136562	0.52497076	0.165660927	1.808438432	5.199575224	3.93386676
TCGA-B8-4619-01A-02R-1325-07	0	2	1	1	0	0.398207704	0.003860314	0.404520735	1.908763147	0.769440809	0.922383031	2.298852087	5.567695118	4.413892374
TCGA-B8-4620-01A-02R-1325-07	1	2	1	1	2	0.923083911	3.287119889	2.761051476	2.67287455	0.786165618	0.921609401	2.667108147	4.251628537	3.57652723
TCGA-B8-4620-11A-01R-1758-07	1	2	1	1	2	0.308114949	1.478947416	0.78847981	0.965215947	0.534657147	0.26595314	2.161446781	5.45072223	4.126603345
TCGA-B8-4622-01A-02R-1277-07	0	3	1	2	2	1.135221551	0.02200343	1.852236783	2.588325057	0.615012506	0.450333318	2.338563866	4.337372704	2.098495071
TCGA-B8-4622-11A-01R-1758-07	0	3	1	2	2	0	0.001506862	0	0	0.191378663	0.074101159	2.375633603	5.300164621	3.323677014
TCGA-B8-5158-01A-01R-1420-07	0	4	1	1	2	0.443933337	0	0.688343085	0.400837119	0.501600723	0.15413179	1.505351866	4.043616928	1.842289812
TCGA-B8-5162-01A-01R-1420-07	0	2	1	1	1	0	0.002406927	0.931780408	0.481492327	0.432607232	0.08478233	2.17859168	4.672659975	2.436566821
TCGA-B8-5163-01A-01R-1420-07	0	3	1	1	2	0	2.252780903	0.865428188	0	0.226974385	0.113985847	1.540548645	4.615247061	2.621675145
TCGA-B8-5164-01A-01R-1420-07	0	3	1	1	2	0.162276034	0	2.174808097	0.84291819	0.437981139	0.206568194	1.447689287	4.77855451	2.902906953
TCGA-B8-5165-01A-01R-1420-07	0	2	1	1	0	0	0.003964336	1.319456365	1.205259703	0.673188838	0.117119797	3.436470444	4.658652719	2.707741291
TCGA-B8-5546-01A-01R-1541-07	0	2	1	1	0	0.261020451	1.35188694	0	0.338492507	0.466168862	0.135355163	3.204032203	4.969850287	1.744457313
TCGA-B8-5549-11A-01R-1541-07	0	3	1	1	0	0	0	0.50937489	0.63644689	0.635158203	0.17650086	2.266842394	6.049265957	3.085317077
TCGA-B8-5551-01A-01R-1541-07	0	3	1	1	0	0.229459304	3.017062323	0.908927925	1.103868992	0.56113525	0.177990977	2.106410014	4.596220443	2.641009927
TCGA-B8-5552-11A-01R-1672-07	0	2	1	1	0	0	2.282286599	0	1.30539839	0.510477199	0.108618891	1.326397746	5.649833218	3.727134916
TCGA-B8-5552-01B-11R-1672-07	0	2	1	1	0	0.48214988	2.806114934	1.230692058	0.612681047	0.767939542	0.147668415	2.558950434	4.826964179	3.13005326
TCGA-B8-A54D-01A-21R-A266-07	1	2	1	1	2	1.663081866	0.005843942	3.29649956	2.127887254	1.372583118	1.701238416	2.910072974	4.988093946	2.859649099
TCGA-B8-A54E-01A-11R-A266-07	0	3	1	1	0	0.475248531	0.734925416	0	0.832132819	0.550131624	0.177990762	3.076755867	4.334473869	3.164836271
TCGA-B8-A54F-01A-11R-A266-07	0	2	1	1	0	1.474649154	4.635912694	3.04370067	2.933987862	1.664067052	0.878360318	3.69196466	4.267576701	2.353543021
TCGA-B8-A54G-01A-11R-A266-07	0	3	1	1	0	1.006566239	0.024336654	2.568484961	3.047135101	1.468394594	1.363437454	3.007918518	4.488369283	2.515051906
TCGA-B8-A54H-01A-11R-A33J-07	1	3	1	1	1	1.505125611	3.962906614	3.26466202	2.392032577	1.311675239	0.761055018	3.109336356	4.304693752	2.089111876
TCGA-B8-A54I-01A-21R-A33J-07	0	3	1	1	0	1.502987633	0.001651356	4.313530881	2.264372595	0.829706034	0.63780215	1.673888923	3.530691496	2.703044131
TCGA-B8-A54K-01A-11R-A33J-07	0	0	1	1	0	1.04354973	0.002865143	4.059056434	3.055044805	1.328746838	0.89897835	4.641013371	4.969033338	1.918341227
TCGA-B8-A7U6-01A-12R-A37O-07	0	3	1	1	0	1.693930437	2.17582785	2.586699285	2.097119176	0.690240277	0.583905442	3.284928989	4.183918697	4.804230177
TCGA-BP-4159-01A-02R-1289-07	1	2	1	1	0	0.456625155	0.089245589	1.095784199	1.578092594	0.539956105	0.157579245	3.760072353	4.454941147	2.469562625
TCGA-BP-4162-01A-02R-1325-07	0	2	1	1	0	1.594995891	4.252383842	2.505013725	0.575238757	0.528055115	0.700281365	1.803640963	4.279119216	3.945727451
TCGA-BP-4165-01A-02R-1289-07	0	0	1	1	0	0.632064944	3.776825033	3.586039376	2.365666116	1.394233976	0.763763875	3.779344647	4.470746255	2.513125903
TCGA-BP-4166-01A-02R-1289-07	1	3	1	1	2	0	0.032274348	1.134591836	0.53338394	0.299544265	0.218372903	3.321651252	4.381419614	2.007225704
TCGA-BP-4167-01A-02R-1325-07	0	2	1	1	2	1.063023392	0.017554892	0.944303092	1.668396381	0.519251182	0.153875482	2.486692847	4.722806331	2.805811924
TCGA-BP-4169-01A-02R-1289-07	1	2	1	1	1	0.529696368	3.907922685	2.937804994	2.276423089	1.581129555	0.874164587	2.21033401	4.504204716	3.953170954
TCGA-BP-4170-01A-02R-1289-07	1	2	1	1	0	0.169239733	2.461468332	2.059431211	1.318223775	0.349677029	0.201530932	2.590009385	4.372876097	2.754231943
TCGA-BP-4173-01A-02R-1289-07	0	3	1	1	1	1.494378997	0.034713924	2.473789406	1.694583858	1.068594475	0.736503773	2.586276067	4.589543341	3.235148496
TCGA-BP-4174-01A-02R-1289-07	0	3	1	1	1	0	0.034725502	1.484581306	0.892477805	0.467949235	0.251638407	2.787034497	4.255709275	1.919324219
TCGA-BP-4177-01A-02R-1420-07	0	2	1	1	0	0.339422208	0.014974786	1.389806285	0.436872193	0.42574916	0.069472835	4.329119048	4.989349327	2.728183641
TCGA-BP-4325-01A-02R-1289-07	0	2	1	1	0	1.154921892	4.361654844	2.198793296	3.027840952	1.328589638	0.714582014	2.969762653	4.337893754	2.463351443
TCGA-BP-4327-01A-01R-1289-07	1	2	1	1	1	1.191885828	3.979374518	4.421924331	2.51345947	2.036958381	1.637059652	3.610234401	3.883265643	2.241206567
TCGA-BP-4331-01A-01R-1289-07	0	2	1	1	0	2.108353649	0.006697058	3.725114215	3.242445965	1.343969125	0.874632461	3.056084221	4.295717061	4.107915795
TCGA-BP-4332-01A-01R-1289-07	0	2	1	1	2	0.812244636	0.069394143	3.145806924	3.595400945	1.075813364	0.580562967	3.936431448	4.517472001	3.24370162
TCGA-BP-4337-01A-01R-1289-07	1	4	1	1	2	0.967685578	2.747783947	3.379400755	3.310321645	2.737687803	1.017330557	2.764112224	4.066701403	2.557559724
TCGA-BP-4338-01A-01R-1289-07	0	3	1	1	0	0	0.01046168	1.143255359	0.6559138	0.286333141	0.069984014	1.563529894	3.515267977	2.187231902
TCGA-BP-4341-01A-01R-1289-07	1	2	1	1	2	2.368558732	0.087328969	3.63964943	2.518475965	1.704613506	1.215522566	4.157733879	3.999222785	3.102638369
TCGA-BP-4342-01A-01R-1289-07	1	3	1	1	1	1.414946384	0.024729128	3.184664692	2.489265017	1.960411653	0.648622402	3.114842258	4.13809932	2.657260621
TCGA-BP-4343-01A-02R-1289-07	0	3	1	1	2	0	0.198708836	0.228377484	0	0.262891895	0.177967597	1.965295919	5.090792021	2.231519013
TCGA-BP-4345-01A-01R-1289-07	0	3	1	1	2	0.773530637	0.03858973	1.727489633	1.329691558	0.682564724	0.421475275	1.983758991	4.744802087	3.23890445
TCGA-BP-4346-01A-01R-1289-07	0	3	1	1	2	1.296871983	0.024722623	3.208106259	3.585937374	1.976018603	1.188332063	2.273469233	4.394763031	2.363782088
TCGA-BP-4347-01A-01R-1289-07	1	2	1	1	2	1.117860659	0.021402462	3.11948254	2.432237808	1.414309261	0.737331077	2.706743381	4.446259039	3.981151693
TCGA-BP-4349-01A-01R-1289-07	1	2	1	1	0	1.639258486	4.13449907	4.09419885	3.183362207	2.369105405	1.088429769	2.717102191	4.112209129	3.338087969
TCGA-BP-4351-01A-01R-1289-07	0	2	1	1	2	2.463891578	5.165860246	3.531197639	2.119670729	1.639179611	1.541695824	3.239344058	4.013574226	2.469068748
TCGA-BP-4352-01A-01R-1289-07	1	4	1	2	2	2.553850548	1.950380238	3.212199911	3.825015118	2.934298209	1.774478496	4.157788609	4.299851788	1.438641406
TCGA-BP-4353-01A-02R-1289-07	0	2	1	1	0	0.386819642	0.104562441	0.701502807	0.691548245	1.217969873	0.264879975	2.60898385	4.875096962	2.42375684
TCGA-BP-4354-01A-02R-1289-07	0	4	1	2	3	1.168262508	0.013421137	2.653527133	2.647737408	0.631290451	0.585761605	2.628114937	4.791925914	4.117719843
TCGA-BP-4355-01A-01R-1289-07	0	4	1	1	2	1.60534841	4.087421347	3.530963098	2.64100778	1.359714697	1.415312607	3.547049652	4.376645068	3.783011064
TCGA-BP-4761-01A-01R-1289-07	0	4	1	1	2	0.689924426	0.101950285	2.506553866	1.787765117	0.381652961	0.244458759	3.806608924	3.16576675	2.148206704
TCGA-BP-4762-01A-02R-1289-07	0	3	1	1	0	0	0.012933018	1.312235007	0.979060833	0.270345938	0.033121414	3.748929079	5.29592489	2.445671469
TCGA-BP-4763-01A-01R-1289-07	1	2	1	1	0	0.704087991	2.353242488	0.214296715	0.505291026	0.55242947	0.108363899	1.406009733	4.646473213	2.871471675
TCGA-BP-4766-01A-01R-1289-07	0	3	1	1	0	1.684957542	3.711660132	3.269993547	2.351182986	2.082433666	0.369740225	2.383859954	4.333211613	2.960008105
TCGA-BP-4768-01A-01R-1289-07	1	2	1	1	0	0.596292672	0.009306092	3.950601644	2.936165971	2.094008417	0.831617487	3.294220552	3.904621583	2.314389387

TCGA-BP-4769-01A-01R-1289-07	0	2	1	1	0	0.214467826	0.02703185	2.477500791	1.438084657	0.804984216	0.233908903	3.465114017	5.008822179	3.921961839
TCGA-BP-4770-01A-01R-1503-07	1	4	1	1	3	0	0.115498451	0.763004799	0.685725612	0.473312675	0.043085295	1.93373767	5.484153485	1.841911236
TCGA-BP-4771-01A-01R-1289-07	0	4	1	2	2	0.877373557	0.022443967	3.953096878	3.559291108	2.104850452	1.063322131	2.992445061	4.499744605	2.899611034
TCGA-BP-4774-01A-01R-1289-07	0	2	1	1	0	2.131427574	3.511517179	2.782780713	3.024805395	1.210697535	0.791808165	2.881715174	4.159891395	5.906239554
TCGA-BP-4777-01A-01R-1289-07	0	3	1	1	0	0.997867369	0.021774719	2.16369065	1.219954871	1.238479344	0.452440925	2.409806333	4.284873633	3.399500069
TCGA-BP-4781-01A-01R-1305-07	1	3	1	1	0	0.381001026	0.009768136	1.725982615	0.682115006	0.596776349	0.093453716	2.753986866	4.366488488	3.318849579
TCGA-BP-4784-01A-01R-1305-07	1	2	1	1	0	0.216212791	1.436291613	1.490989375	0.719595486	0.584613726	0.08425615	4.091294575	5.058474976	2.594424298
TCGA-BP-4787-01A-01R-1305-07	0	4	1	2	2	0.215811305	2.906080166	2.043176883	2.079680694	1.122451154	0.336795332	1.772530668	4.367322472	2.002867832
TCGA-BP-4789-01A-01R-1305-07	0	2	1	1	0	0.345854707	0.018548232	1.617636588	1.578517102	0.895403344	0.169620048	4.035408788	4.839155687	2.548917994
TCGA-BP-4790-01A-01R-1305-07	1	2	1	1	0	0.62494637	0.079796989	1.910753445	1.868105228	1.373273918	0.222131765	2.955911866	4.956381501	2.513745928
TCGA-BP-4795-01A-02R-1420-07	1	2	1	1	0	0	1.378547364	1.045384294	0.762656419	0.345730371	0.099929876	2.690250176	5.02445717	3.029019819
TCGA-BP-4797-01A-01R-1305-07	0	3	1	1	2	0	0.002830028	1.128244096	0.551785924	0.477773797	0.128083384	1.829846913	4.194867801	2.707276508
TCGA-BP-4799-01A-01R-1305-07	1	3	1	1	2	0	0.016889951	0.21925386	0.280877853	0.153744084	0.103315533	1.512596069	4.546268309	2.158520539
TCGA-BP-4804-01A-02R-1305-07	0	2	1	1	0	0.522286465	0.038386699	2.038832196	2.070051813	0.509707602	0.413876018	2.995894216	4.547302794	3.036956483
TCGA-BP-4807-01A-01R-1305-07	0	3	1	1	0	0	0.018248614	1.725276818	0.323882332	0.279938506	0.12026787	2.982712393	4.594876709	2.691322779
TCGA-BP-4959-01A-01R-1334-07	0	3	1	1	0	0	0.004551918	0.938202643	0.535943413	0.472905803	0.157754901	1.843567457	4.159166594	2.500139361
TCGA-BP-4960-01A-01R-1334-07	0	3	1	1	1	2.323957111	0.006482473	2.344797882	2.072321789	0.844721638	0.969521284	2.347728306	3.970290673	2.810835309
TCGA-BP-4961-01A-01R-1334-07	0	2	1	1	0	0	0.00174658	2.064397644	2.153518598	0.6173566	0.401353566	3.268714573	4.672239782	3.517876691
TCGA-BP-4962-01A-01R-1334-07	0	2	1	1	1	0	0.002959709	1.163707391	0.857676029	0.295301303	0.172882101	2.689555348	4.348832003	2.318719422
TCGA-BP-4963-01A-01R-1334-07	0	3	1	1	0	1.939517774	0.001149033	2.11117615	3.097958683	0.958891971	0.678300069	2.432100977	4.365907312	3.300842541
TCGA-BP-4964-01A-01R-1334-07	0	2	1	1	0	0.649716407	3.604388996	2.124121486	1.899379413	0.63399768	0.549431327	3.04534336	3.997076358	2.341084278
TCGA-BP-4965-01A-01R-1334-07	0	2	1	1	0	0.35074225	0.000743541	0.951638719	1.303238084	0.51242405	0.270675457	2.606181267	4.475367959	2.156454783
TCGA-BP-4967-01A-01R-1334-07	1	2	1	1	2	1.092015578	0.000917198	2.781715376	1.893796925	0.959344043	0.98714967	2.828383377	4.468971664	2.485801529
TCGA-BP-4969-01A-01R-1334-07	0	2	1	1	0	1.304671035	3.864633631	2.345607613	3.22898889	2.53447539	1.117411182	3.840974107	3.607149309	2.500155499
TCGA-BP-4970-01A-01R-1334-07	0	3	1	1	0	0	0.00232406	1.116680309	0.173142878	0.356086417	0.217381806	2.407115225	4.680613728	2.972934923
TCGA-BP-4972-01A-01R-1334-07	0	3	1	1	2	0.344986407	3.09722207	2.041677728	1.546456704	1.106738763	0.413334288	3.25599348	4.52259402	2.593554555
TCGA-BP-4973-01A-01R-1334-07	0	3	1	1	2	1.42443531	0.004260284	2.593825273	2.172656994	1.870685576	0.987352635	3.191253009	4.188726773	1.78068882
TCGA-BP-4974-01A-01R-1334-07	0	4	1	2	2	1.841982435	4.60782408	2.715397492	2.495640201	1.913236651	1.057498907	3.841408617	4.614925532	4.640054345
TCGA-BP-4977-01A-01R-1334-07	0	3	1	1	0	0.93019234	0.005495412	1.507915587	2.208554746	1.460824202	0.81054052	3.13590707	4.58749811	2.15984592
TCGA-BP-4983-01A-01R-1334-07	1	4	1	1	2	1.415052274	4.47551773	2.218803341	2.655175976	0.548994354	0.908110607	3.672623234	4.732338543	3.260595907
TCGA-BP-4986-01A-01R-1334-07	1	3	1	1	0	0	0.004707224	3.528616454	2.498926689	1.627591588	0.662725934	1.93973361	4.685920132	4.122451741
TCGA-BP-4987-01A-01R-1334-07	0	2	1	1	0	0.719155168	4.173861106	3.333416105	2.454292512	1.022446188	0.742485524	3.449932158	4.622503033	2.341198164
TCGA-BP-4989-01A-01R-1334-07	0	3	1	1	2	1.315170636	0.009021148	3.035279928	3.449180653	1.636372921	1.190922276	2.194541859	4.282500851	3.783438297
TCGA-BP-4992-01A-01R-1334-07	1	4	1	1	0	2.289295912	0.017707483	4.145359517	4.009158742	3.140370176	1.755985646	2.932108326	3.606548314	5.114552317
TCGA-BP-4993-01A-02R-1420-07	0	3	1	1	0	0.395124999	0.002551925	2.229288736	0.704972322	0.740404338	0.198980384	3.351822202	4.327969362	3.040236118
TCGA-BP-4995-01A-01R-1334-07	1	3	1	1	0	0.378417578	0.002429385	0.38445687	1.519108773	0.908149518	0.785978668	2.406244173	5.476011561	3.90126985
TCGA-BP-4998-01A-01R-1334-07	0	3	1	1	0	1.300317592	0.001823349	1.559596236	1.485636671	1.069171825	0.923665792	2.958427615	4.656961234	2.334390802
TCGA-BP-5000-01A-01R-1334-07	0	3	1	1	0	0.475893795	0.730124735	1.92036674	2.161435076	0.613462009	0.460575985	1.994570468	3.951902136	2.840168812
TCGA-BP-5001-01A-01R-1334-07	0	2	1	1	0	1.170515049	2.679336648	3.188972238	2.939522901	0.923798323	1.273869306	2.579607573	3.583391284	4.472996844
TCGA-BP-5004-01A-01R-1334-07	0	3	1	1	0	1.41897958	0.000847933	2.241343681	1.562160349	1.608022347	0.751745096	2.331064899	4.499813873	4.010784033
TCGA-BP-5006-01A-01R-1334-07	0	2	1	1	0	0.56808617	0.002931101	1.981113136	1.291407103	0.51226895	0.394100133	3.189426362	4.20686195	4.192975615
TCGA-BP-5008-01A-01R-1334-07	0	2	1	1	0	0.938700242	0.00278452	2.243022735	0.818850949	0.768765149	0.536692595	2.787041199	4.468270009	4.150884765
TCGA-BP-5009-01A-01R-1334-07	0	3	1	1	0	0.434590484	0.003794808	2.232197674	2.693364281	1.660462635	0.41143913	1.995370597	4.064586112	3.291977101
TCGA-BP-5169-01A-01R-1426-07	1	4	1	1	0	1.423012411	0.006799752	1.136463114	2.771678687	0.397049283	0.328996854	2.285342966	3.239039028	2.126923932
TCGA-BP-5170-01A-01R-1426-07	0	2	1	1	0	0	0.005462005	0.426148819	0	0.442189495	0.025645186	4.278960276	4.023340581	2.283154683
TCGA-BP-5173-01A-01R-1426-07	1	2	1	1	0	0	0.002210704	1.078225797	0.788875422	0.849215314	0.14894973	3.829256142	4.614869338	1.982973892
TCGA-BP-5174-01A-01R-1426-07	0	2	1	1	0	0.359684594	2.684916401	1.725238367	1.432356583	0.667163772	0.290642178	2.285561913	3.430376571	1.969114783
TCGA-BP-5175-01A-01R-1426-07	0	3	1	1	0	0.362087683	0.002310967	2.255841723	1.872889766	0.189571883	0.362340882	1.868296216	2.977252317	3.041519064
TCGA-BP-5176-01A-01R-1426-07	1	2	1	1	0	0	1.764780991	1.501791204	0.970550463	0.310431536	0.045261114	2.770198283	4.025501557	2.240385465
TCGA-BP-5180-01A-01R-1426-07	0	2	1	1	0	0	0.003118112	1.432323919	1.610467501	0.677272864	0.113489177	2.651534328	3.680276631	1.671541768
TCGA-BP-5181-01A-01R-1426-07	0	2	1	1	0	0	1.951297136	0.92254578	0.475991853	0.190845884	0.080138707	3.309323096	4.126634697	1.523452567
TCGA-BP-5183-01A-01R-1426-07	0	3	1	1	2	0.882595135	0.008184544	2.092311826	1.364508391	1.369686956	0.519427397	2.368065044	4.073321975	2.167888187
TCGA-BP-5184-01A-01R-1426-07	0	3	1	1	0	0	0.001484972	0.246829484	0.983612369	0.763639596	0.103883282	3.290803513	4.619756983	2.827248971
TCGA-BP-5185-01A-01R-1426-07	0	3	1	1	0	0.283902525	0.007035434	1.581564354	1.85192094	0.981195342	0.304500701	2.5329517	3.105193185	2.156643771
TCGA-BP-5186-01A-01R-1426-07	0	2	1	1	0	0.192915556	3.408322392	1.458420177	1.539838253	0.603645167	0.194656556	2.38172673	4.561920524	2.568445607
TCGA-BP-5187-01A-01R-1426-07	0	2	1	1	0	0.322469237	0.03609489	0.327713727	0.415713896	0.505554871	0.163225338	2.972712809	4.571471405	2.380955991
TCGA-BP-5189-01A-02R-1426-07	0	4	1	1	0	0	0.003643754	1.131808144	0.879738592	0.509608628	0.294411767	1.711910306	4.202151051	2.704043319
TCGA-BP-5191-01A-01R-1426-07	1	2	1	1	2	0.230884464	0.002811288	1.72125295	0.760618473	0.789676811	0.302919149	2.373752799	3.605478972	1.8676839

TCGA-BP-5194-01A-02R-1426-07	0	2	1	1	0	0.147432125	0.00435487	1.140829978	1.003049543	0.411097782	0.108545151	3.515938305	4.386233303	1.981904443
TCGA-BP-5195-01A-02R-1426-07	1	2	1	1	0	0.326541301	0.00308553	1.27644857	0.884932423	0.821079082	0.103311851	2.351044878	4.644757672	2.889832309
TCGA-BP-5196-01A-01R-1426-07	0	2	1	1	0	0	0.001244441	2.508625321	2.66075938	1.163162569	0.572314339	2.018624582	4.114193301	3.317361774
TCGA-BP-5199-01A-01R-1426-07	0	4	1	1	1	1.177767518	0.001022962	2.789100543	3.042115879	1.238483639	0.644721485	4.058888209	4.330480497	3.384799079
TCGA-BP-5200-01A-01R-1426-07	0	4	1	1	1	0.595027832	0.001654519	0.699320897	0.347402133	0.092972184	0.129669134	1.840689241	4.501102538	4.297318167
TCGA-BP-5202-01A-02R-1426-07	1	2	1	1	2	0.157284726	0.001866437	2.175817584	0.822043676	0.435312373	0.195173495	2.447233503	4.486669752	3.889582576
TCGA-CJ-4634-01A-02R-1325-07	0	2	1	1	0	1.359990555	4.09631153	2.914722156	1.893691223	1.00078	0.490776939	2.823419659	4.410348852	2.933751193
TCGA-CJ-4635-01A-02R-1305-07	0	3	1	1	0	0.496057878	0.050664794	4.180341777	3.57806773	2.165420375	0.706970775	2.940067289	4.418945894	2.650618375
TCGA-CJ-4636-01A-02R-1325-07	0	3	1	1	2	0.474855485	0.038968472	1.131324538	2.292130912	1.80180541	0.934595166	2.673623308	4.424289	2.446728043
TCGA-CJ-4638-01A-02R-1325-07	0	4	1	2	2	1.090106981	2.489462699	1.721961996	2.296755121	1.077161382	2.035422062	2.99497793	3.357621955	2.014439957
TCGA-CJ-4639-01A-02R-1325-07	0	2	1	1	1	0.562177255	4.366455987	0.78888533	0.709579795	1.768090217	0.669766622	3.894633596	4.443632876	2.097482295
TCGA-CJ-4641-01A-02R-1325-07	0	4	1	2	2	0.584515657	3.844626093	1.236532118	1.783319797	1.572210526	0.847434868	2.290709263	4.42500146	3.270549694
TCGA-CJ-4643-01A-02R-1325-07	1	3	1	1	1	1.783270945	4.73008894	2.50599034	1.803202384	1.533842331	1.078571963	2.47557793	4.816414542	2.963432184
TCGA-CJ-4644-01A-02R-1325-07	0	3	1	2	2	0	1.63859914	0.998378817	0.284234252	0.260690895	0.037082186	2.459541008	4.697158229	1.92350801
TCGA-CJ-4868-01A-01R-1305-07	0	3	1	2	2	0.455111029	0.00899236	1.707432186	1.689446699	0.557304781	0.17390381	2.778305735	4.128216924	2.672824156
TCGA-CJ-4869-01A-02R-1426-07	0	2	1	1	1	0.168019241	0	0.70381218	0.730093044	0.419438058	0.103512838	2.183830702	4.465071028	2.484339154
TCGA-CJ-4870-01A-01R-1305-07	0	2	1	1	2	0	1.577000613	2.465108623	0.286059898	0.2363493	0.089627875	4.618751221	4.399793679	2.567864455
TCGA-CJ-4871-01A-01R-1305-07	0	4	1	2	2	0	0.010240575	0.895669418	1.210770035	0.509272817	0.110813862	1.873783547	4.426735492	2.398905269
TCGA-CJ-4872-01A-01R-1305-07	0	4	1	1	0	0.78055106	0.01735265	0.36998012	1.721118017	0.739959895	0.375749198	2.556940717	4.562211029	2.214122317
TCGA-CJ-4873-01A-01R-1305-07	1	3	1	1	2	1.755922222	2.814782799	2.795265916	2.931297821	0.889465324	0.968702897	2.494457481	3.705865477	2.817124867
TCGA-CJ-4878-01A-01R-1305-07	1	2	1	1	2	2.775154283	4.201457606	4.082553709	2.941211982	1.427852641	1.040966796	3.511234102	4.218929958	3.358352509
TCGA-CJ-4882-01A-02R-1426-07	0	3	1	1	2	1.200809525	0.101620766	1.679798096	1.2886448	1.020575587	0.55736336	3.75225168	4.777912878	2.9429324
TCGA-CJ-4884-01A-01R-1305-07	1	3	1	1	2	0.7168189	3.731630407	3.036996515	2.355790393	1.441506974	0.769331224	2.555101519	4.308543477	3.10171201
TCGA-CJ-4886-01A-01R-1305-07	0	3	1	1	0	0.207326596	2.641810498	2.516191893	0.497867467	1.194526017	0.236263294	2.857277022	4.638912323	3.010869141
TCGA-CJ-4887-01A-01R-1305-07	0	3	1	2	2	1.749845531	0.005099992	2.690202095	3.167109116	1.729094641	0.472907128	2.74016315	3.925630942	2.880569047
TCGA-CJ-4888-01A-01R-1305-07	0	4	1	2	2	0.325479556	0.007163285	1.614219335	1.51395889	1.119779804	0.363902641	2.092567366	4.611958093	2.928205906
TCGA-CJ-4890-01A-01R-1305-07	1	4	1	2	2	0	0.009280381	1.117228287	1.239451618	1.2570863	0.275246907	2.177182835	4.659262273	2.95034074
TCGA-CJ-4891-01A-01R-1305-07	0	4	1	1	2	1.17201262	1.83645358	2.138416662	1.313444979	1.075592462	0.323761818	1.929991215	3.468205289	3.110385879
TCGA-CJ-4894-01A-01R-1305-07	0	3	1	1	2	0.186517586	0.005583598	2.1284737	2.108465033	1.710953451	0.868916474	3.111891005	4.394003897	2.255133461
TCGA-CJ-4900-01A-01R-1334-07	1	4	1	2	3	1.187883384	2.433469406	1.84913641	2.361453116	1.205351977	0.966533204	2.676709713	3.778475411	2.964923847
TCGA-CJ-4901-01A-01R-1426-07	0	3	1	1	2	0.377815782	0.007262797	0	1.675997888	0.527174872	0.636121748	2.639703671	3.920718391	2.629425996
TCGA-CJ-4903-01A-01R-1426-07	0	3	1	1	0	0.663101592	0	0.672734912	1.595857788	0.722899648	0.573206117	3.412762458	4.413932354	2.153531624
TCGA-CJ-4905-01A-02R-1426-07	0	2	1	1	0	0	2.672973968	1.816657901	0.958913689	0.59157728	0.135168212	3.627103775	4.529894838	2.327152745
TCGA-CJ-4912-01A-01R-1426-07	0	3	1	1	1	1.851041868	0.001509211	3.46612286	3.921776832	1.475187154	1.418295236	2.6521489	3.865846646	3.460906571
TCGA-CJ-4916-01A-01R-1426-07	1	3	1	1	2	0.988595558	3.485472331	1.249720428	1.857850886	1.547286784	0.739015378	3.334272157	3.998435461	2.832631718
TCGA-CJ-4918-01A-01R-1426-07	0	4	1	2	2	0	0.008965075	0.19052747	0.636579743	0.683999615	0.148046719	3.748653119	4.619996612	2.701006743
TCGA-CJ-5671-01A-11R-1541-07	0	3	1	1	0	0.208254172	0.002515685	0.396406669	1.025214544	0.26601824	0.06213623	2.581002556	4.229471527	2.31732142
TCGA-CJ-5672-11A-01R-1541-07	1	3	1	1	0	0.165953145	0.001975263	0.581586629	0.722615441	0.48273662	0.113774383	2.307415857	5.719964928	3.302486478
TCGA-CJ-5672-01A-11R-1541-07	1	3	1	1	0	0	0.028167088	1.478277944	0.664259508	0.359597276	0.218019458	2.503065193	3.841979622	2.381608296
TCGA-CJ-5676-01A-11R-1541-07	0	3	1	1	2	0.246040768	0	1.33034676	1.798799912	0.354824508	0.114157694	2.738783705	4.061057122	2.688665644
TCGA-CJ-5677-01A-11R-1541-07	0	4	1	2	2	0.454457648	1.273803053	0.646623124	1.310962635	0.371057006	0.143964704	1.962250668	4.085644488	2.339787113
TCGA-CJ-5677-11A-01R-1541-07	0	4	1	2	2	0.188897329	2.567955199	0.192112893	0.950553756	0.557311308	0.190604408	1.770782959	5.269796676	4.066250641
TCGA-CJ-5678-01A-11R-1541-07	0	3	1	2	1	0	0.001516625	0.96560343	2.390278496	0.447469651	0.1367983	3.894901677	3.863958622	2.655469905
TCGA-CJ-5679-01A-11R-1541-07	1	4	1	1	2	0.242025565	0.193702217	1.314860775	0.981227062	0.506254469	0.099197368	1.811247882	4.033656474	2.21559166
TCGA-CJ-5680-01A-11R-1541-07	0	4	1	2	2	0.374023423	2.34850174	1.410008471	0.670771472	0.543301289	0.177675712	2.623159375	4.336973886	1.701998666
TCGA-CJ-5680-11A-01R-1541-07	0	4	1	2	2	0.549103257	3.231726769	0.557382955	1.020967848	0.69514026	0.301534728	2.134459193	5.489360106	3.512505462
TCGA-CJ-5681-01A-11R-1541-07	0	3	1	2	2	0	0.357373008	2.453061061	1.38783765	0.374592454	0.394120248	1.144862654	5.022030678	2.686026279
TCGA-CJ-5681-11A-01R-1541-07	0	3	1	2	2	0.788588567	1.652071506	0.63733817	1.555663939	1.083498695	0.29712689	1.525891714	5.080083619	3.607155228
TCGA-CJ-5682-01A-11R-1541-07	0	4	1	2	2	0.25865236	0	1.137231381	0.607511076	0.236273989	0.049326277	3.136646177	4.904635809	2.229572327
TCGA-CJ-5686-01A-11R-1672-07	0	3	1	1	0	0.454345727	3.195917412	0.587386105	0.990662128	0.723153376	0.240500267	2.643361252	4.150377044	2.07128558
TCGA-CJ-5689-11A-01R-1541-07	1	4	1	1	0	0	0.001564971	0.478515337	0	0.494921262	0.081557425	1.955174598	5.335156968	3.913489643
TCGA-CJ-5689-01A-11R-1541-07	1	4	1	1	0	0	0.001223054	0.206231681	1.142357149	0.289692259	0.056723255	2.510861922	4.376426592	2.65894131
TCGA-CJ-6027-01A-11R-1672-07	1	4	1	1	0	0	0.004582638	0.365388813	0.81137638	0.723767028	0.13787752	2.081523819	4.234243633	2.331939233
TCGA-CJ-6030-11A-01R-1672-07	0	3	1	1	0	0.414700889	0.006285895	0.15448696	0	0.398829699	0.145585497	1.964018157	5.122001564	3.311356284
TCGA-CJ-6030-01A-11R-1672-07	0	3	1	1	0	0.336535254	0.00212832	0.849992444	0.766033497	0.621697623	0.149726971	1.972890603	4.377249316	1.699001258
TCGA-CJ-6031-01A-11R-1672-07	0	3	1	1	0	0	0.002161361	0.346705071	0.439076371	0.315440161	0.066618208	2.886722042	4.160058883	2.000790893
TCGA-CJ-6032-01A-11R-1672-07	0	3	1	1	1	0.311640506	2.851288931	2.10911951	0.71631653	0.560461944	0.098245346	1.74789661	4.034927941	4.053182177
TCGA-CJ-6033-01A-11R-1672-07	0	4	1	2	2	0	1.63766024	1.366111813	0.755913034	0.494830203	0.089558962	2.575213288	3.944922927	2.500661789

TCGA-CW-5583-01A-02R-1541-07	0	2	1	1	0	0.591676772	4.052905985	2.592899459	0.883617215	0.861111345	0.135909752	3.639381295	4.348544954	2.210209391
TCGA-CW-5584-01A-01R-1541-07	1	3	1	1	2	0.192034205	0	1.375501747	1.219597744	0.361725853	0.077900938	2.434982268	4.577493658	1.951469577
TCGA-CW-5584-11A-01R-1541-07	1	3	1	1	2	0.404722189	0	0	0.898059128	0.520970687	0.088273477	2.073673197	5.414428857	3.512936146
TCGA-CW-5585-01A-01R-1541-07	0	2	1	2	2	0.453036218	0	1.809073543	1.400339192	0.618761727	0.182756145	3.421634393	4.123308616	1.844879256
TCGA-CW-5587-11A-01R-1541-07	0	2	1	1	2	0	1.571991569	0	0.436951964	0.317859889	0.091925232	1.78300603	5.228986222	3.159641473
TCGA-CW-5587-01A-01R-1541-07	0	2	1	1	2	0.346023631	2.30351143	1.250903187	0.928660909	0.25092362	0.109988351	2.115276136	4.243064165	1.899086387
TCGA-CW-5588-01A-01R-1541-07	1	2	1	1	0	0	0.60992489	0.214415295	1.035024553	0.240744691	0.043572797	2.745788295	4.563847738	2.114089387
TCGA-CW-5589-11A-01R-1541-07	0	2	1	1	0	0.185941935	0.002228683	0	0.793812968	0.460241542	0.085309265	2.448138683	5.364664822	3.761130076
TCGA-CW-6087-11A-01R-1672-07	0	4	1	2	2	0	0.000957692	0	1.072392398	0.505766837	0.194626227	2.164018877	5.095042539	2.741296913
TCGA-CW-6087-01A-11R-1672-07	0	4	1	2	2	0.151007964	0.001788038	0	0.667670967	0.22534881	0.098101355	1.861500567	5.08952751	2.048674182
TCGA-CW-6088-01A-11R-1672-07	0	2	1	1	0	0.785253152	0	0.576310789	1.468999335	0.842466868	0.87074812	2.368613269	4.817564485	2.879362729
TCGA-CW-6088-11A-01R-1672-07	0	2	1	1	0	0.269066057	0.000830845	0.143233572	1.157150409	0.705685939	0.494224498	2.294421697	5.988953808	3.072029182
TCGA-CW-6093-01A-11R-1672-07	1	0	1	1	0	0.503878394	0	0.511590369	1.201952621	0.86850692	0.226881117	3.228409258	4.575059324	3.505734741
TCGA-CZ-4853-01A-01R-1426-07	1	2	1	1	0	0.898532584	0.00349869	2.518374178	1.287047587	1.192359564	0.469460273	3.170765857	3.809445526	1.583753112
TCGA-CZ-4854-01A-01R-1305-07	1	2	1	1	0	1.594589478	0.018442175	2.634731898	2.633973303	1.581207125	0.748339311	3.298398402	4.74470267	2.415231672
TCGA-CZ-4857-01A-01R-1305-07	0	3	1	2	2	0.915744436	0.004305919	2.365200269	2.686491416	1.41954548	0.556897921	2.062278362	4.203827139	2.46006717
TCGA-CZ-4858-01A-01R-1305-07	0	4	1	1	1	0.232962292	0.011321278	2.300270673	2.171498991	1.216019437	0.250176776	2.898241063	4.28278253	1.206682195
TCGA-CZ-4859-01A-02R-1426-07	0	2	1	1	0	0	2.206786583	1.923571825	0.525062627	0.183897405	0.049619526	3.406971091	4.046244417	2.410942269
TCGA-CZ-4860-01A-01R-1305-07	0	4	1	2	3	0.180669593	0.016132883	2.222667623	0.236192257	0.610780223	0.148872591	3.364873356	4.058671087	1.245551544
TCGA-CZ-4862-01A-01R-1305-07	0	2	1	1	0	0.841980037	0.676150593	2.079863236	1.510550172	1.58723527	0.652637972	2.646228844	5.207394631	2.708499148
TCGA-CZ-4863-01A-01R-1503-07	0	3	1	1	2	1.503437913	2.714654927	2.964737192	2.377036624	1.039452476	0.673020442	2.464927848	4.440680713	3.170640021
TCGA-CZ-4864-11A-01R-1503-07	1	3	1	1	1	0	0.003741124	0.304782526	0.944204305	0.339247896	0.198271018	1.916532843	5.086555085	3.91968712
TCGA-CZ-4865-01A-02R-1503-07	1	2	1	1	0	0	2.315539959	0.657458522	1.176312296	0.304197288	0.142452343	2.111062371	4.739300152	3.082064034
TCGA-CZ-5451-11A-01R-1503-07	1	3	1	1	1	0.154701058	0	0.546952013	0.380554893	0.275386258	0.156115581	2.254769203	5.25286714	3.903814253
TCGA-CZ-5451-01A-01R-1503-07	1	3	1	1	1	0	0.001461921	1.680046432	0.783811062	0.275661702	0.045383808	3.329132263	4.247439906	2.205918419
TCGA-CZ-5452-11A-01R-1503-07	1	2	1	1	1	0.187568304	0.085229357	0.771323437	0.945320792	0.486458936	0.128893418	2.897084834	5.953733253	3.43239732
TCGA-CZ-5452-01A-01R-1503-07	1	2	1	1	1	0.660294164	0.001175865	2.989820652	1.645310182	0.543856179	0.278967331	2.574299118	3.840758491	2.127274639
TCGA-CZ-5453-11A-01R-1503-07	1	2	1	1	1	0	0	0	0.543440759	0.720641992	0.096091088	1.985372512	6.093916086	3.539701754
TCGA-CZ-5454-01A-01R-1503-07	0	2	1	2	1	0	0	0.397994749	0.978689202	0.384930728	0.090143715	4.465488569	3.870178363	1.802720373
TCGA-CZ-5455-01A-01R-1503-07	0	4	1	2	2	0	0.001566229	1.188579217	1.194499176	0.540960388	0.195997822	2.094599107	4.496149454	2.732916062
TCGA-CZ-5455-11A-01R-1503-07	0	4	1	2	2	0	0.001878297	0.669146058	0.207340955	0.306317378	0.029322559	2.128775247	5.659366625	3.316300066
TCGA-CZ-5456-11A-02R-1503-07	0	3	1	1	1	0	0	1.005660863	0.611576806	0.702463982	0.097791982	1.773520112	5.667111996	3.770393492
TCGA-CZ-5456-01A-01R-1503-07	0	3	1	1	1	0.361545236	0.004610428	0.659887096	1.097074827	0.443836482	0.095051688	1.960679845	4.715898655	1.844180698
TCGA-CZ-5457-01A-01R-1503-07	0	4	1	1	2	0	0.001871947	1.041693854	0.823893606	0.264356898	0.037877467	4.655942687	4.651795441	1.997924637
TCGA-CZ-5457-11A-01R-1503-07	0	4	1	1	2	0	0	0.31974806	0.217174446	0.363744629	0.081709038	2.552876602	5.48312087	3.434549933
TCGA-CZ-5460-01A-01R-1503-07	0	2	1	2	2	0.178766139	0	2.544806623	1.791781101	0.515164411	0.278143617	2.785803433	4.099486278	2.034710673
TCGA-CZ-5461-11A-01R-1503-07	0	4	1	2	0	0	0.000817982	0	0	0.425262001	0.085128661	1.611277761	5.752895775	3.413729761
TCGA-CZ-5462-11A-01R-1503-07	1	3	1	2	0	0	0	0.547457101	0.884478234	0.334157005	0.013833121	2.560753271	6.030425309	2.962310236
TCGA-CZ-5462-01A-01R-1503-07	1	3	1	2	0	0.315643611	0	1.750875506	0.72436913	0.396711	0.167952718	1.596280675	4.407304996	3.291693254
TCGA-CZ-5463-11A-01R-1503-07	1	2	1	1	1	0	0.000925821	0.1587416	1.149595715	0.849694236	0.188552531	2.476169375	5.876228719	3.321652839
TCGA-CZ-5464-01A-01R-1503-07	1	2	1	2	2	0.566704864	0.016483052	1.37413546	1.288896144	0.33383074	0.258236527	2.676229565	4.202021302	2.157188131
TCGA-CZ-5465-01A-01R-1503-07	1	2	1	1	2	0.461977223	3.388320266	1.593366116	2.216757051	0.558821235	0.257673029	3.170245748	4.328608574	2.850251646
TCGA-CZ-5466-11A-01R-1503-07	1	2	1	1	2	0	0.001559612	0.13486527	0.174177041	0.300449807	0.036427042	2.21467347	5.395426139	3.449826987
TCGA-CZ-5467-01A-01R-1503-07	1	4	1	1	2	0.178257311	0.749659159	1.454595473	0.609816436	0.37671964	0.257835206	3.737301347	4.349519417	3.155825151
TCGA-CZ-5468-11A-01R-1503-07	1	4	1	2	2	0	0	0.773305846	0.63896746	0.400195844	0.129302776	2.79625606	5.610525597	3.50140077
TCGA-CZ-5469-11A-01R-1503-07	0	2	1	1	1	0	0	0.654969411	1.038015656	0.506799464	0.110987507	2.196679904	5.846802125	3.878492246
TCGA-CZ-5469-01A-01R-1503-07	0	2	1	1	1	0.521021815	0.007035494	1.749330713	0.826422139	0.627121161	0.3790476	3.453700149	4.143176883	1.705913959
TCGA-CZ-5470-11A-01R-1503-07	1	3	1	1	1	0	1.221150853	0.528076107	0.658843039	0.456464923	0.067720227	2.799392332	6.216355119	3.417958673
TCGA-CZ-5470-01A-01R-1503-07	1	3	1	1	1	0.30949645	2.268615535	1.972143542	1.692977938	0.689809159	0.188861825	2.778071494	4.038588237	2.158427511
TCGA-CZ-5982-11A-01R-1672-07	0	2	1	1	0	0	1.671329809	0.365416943	0.462026477	0.519791828	0.042767844	1.814616257	4.943770301	2.986137701
TCGA-CZ-5984-01A-11R-1672-07	0	3	1	1	0	0.463924825	0	0.471120035	2.012322802	0.480084185	0.272942945	2.371478305	4.01551022	2.154551114
TCGA-CZ-5985-11A-01R-1672-07	0	2	1	1	1	0.52320955	0	0	0.662538404	0.301327179	0.070844373	2.134796699	5.136875757	3.321006503
TCGA-CZ-5986-01A-11R-1672-07	0	3	1	1	0	1.094442525	0.003676907	0	1.641193184	1.059086952	0.353340393	2.754362991	4.305208785	3.492378521
TCGA-CZ-5986-11A-01R-1672-07	0	3	1	1	0	0	0	0.459845018	0.987811928	0.742471077	0.21412618	2.481688939	5.57071948	3.481714558
TCGA-CZ-5987-01A-11R-1672-07	0	2	1	2	2	0.827376009	0.002091335	0	0.755660551	0.878258566	0.108002988	1.9466186	3.897926274	1.822488073
TCGA-CZ-5987-11A-01R-1672-07	0	2	1	2	2	0	0.003697299	0	0.686037588	0.542990534	0.073924141	1.76106075	5.331192056	3.731265438
TCGA-CZ-5988-11A-01R-1672-07	0	2	1	1	0	0	0.00155854	0	0.597344649	0.722106729	0.149294359	2.444263157	5.471700303	3.546578527
TCGA-CZ-5988-01A-11R-1672-07	0	2	1	1	0	0.742847084	0.051836566	0	1.135724676	0.586896841	0.152300904	2.620617865	4.342156296	2.427221448

TCGA-CZ-5989-01A-11R-1672-07	0	2	1	1	1	0	0.001686318	0	0	1.580426325	0.225994342	1.180215377	5.077234774	2.806409725
TCGA-CZ-5989-11A-01R-1672-07	0	2	1	1	1	0	0.002388011	0.201736332	1.469271187	0.923423934	0.156890299	2.368570516	6.139048816	3.728117759
TCGA-DV-5565-01A-01R-1541-07	0	2	1	1	0	0.21598221	0.147948048	2.044108262	1.841534628	0.607181025	0.060612624	2.808781041	4.425221152	2.971746497
TCGA-DV-5566-01A-01R-1541-07	1	2	1	1	0	0.271369153	1.754775718	1.730257525	1.800509629	0.612498376	0.102074253	2.447411353	4.050232603	3.254967035
TCGA-DV-5568-01A-01R-1541-07	0	2	1	1	0	0	0	1.600216917	1.60728277	0.772792002	0.156011391	2.16035127	3.99952996	3.606646288
TCGA-DV-5569-01A-01R-1541-07	0	2	1	1	0	0.523540319	2.800421846	1.22422417	0.473911282	0.315376887	0.191647175	3.055040966	3.980531511	3.330246532
TCGA-DV-5574-01A-01R-1541-07	0	2	1	1	0	0	0	0.878411293	0.449900802	0.286989734	0.075158674	3.062489874	4.640030219	3.004685205
TCGA-DV-5575-01A-01R-1541-07	0	2	1	1	0	0.220976496	2.618437649	1.59568528	1.072779063	0.699321487	0.152394636	3.245848233	4.401346242	2.697837489
TCGA-DV-A4VX-01A-11R-A266-07	0	4	1	2	2	0.723864189	0.008777511	1.469465246	3.092477785	0.483272735	1.247004729	3.540021065	3.975491039	3.086626045
TCGA-DV-A4VZ-01A-11R-A266-07	0	2	1	3	0	2.088380126	0.001464249	4.134156494	4.09955147	0.827987646	1.635135047	3.712173224	4.500507705	5.2101618
TCGA-DV-A4W0-05A-11R-A266-07	0	3	1	1	0	0.692429347	0	3.312902543	2.839964437	1.547301342	0.736033706	3.426720316	5.064179235	3.023919801
TCGA-DV-A4W0-01A-11R-A266-07	0	3	1	1	0	1.437035702	0	1.110446794	1.712310845	1.85154233	0.620807155	2.682355712	4.167041085	3.971679566
TCGA-EU-5905-01A-11R-1672-07	1	3	1	1	0	0.304367909	1.71517762	2.19762	0.393053666	0.27840269	0.126350511	2.914321416	4.178191829	3.044485859
TCGA-EU-5906-01A-11R-1672-07	0	2	1	1	0	0.390061435	0.003351878	0.88199081	0.975147355	0.454588804	0.126510846	2.307575658	4.456642534	3.014323549
TCGA-EU-5907-01A-11R-1672-07	1	3	1	1	2	0.73041789	0	0.533466065	0.909831566	0.211091419	0.108134633	2.136845268	4.145196454	2.673842428
TCGA-G6-A5PC-01A-11R-A33J-07	0	4	1	2	0	1.063409676	0.253753693	2.500969914	2.42208753	0.431000315	0.401042021	3.540532969	2.376218773	2.849086739
TCGA-MW-A4EC-01A-11R-A266-07	1	2	1	3	0	1.252735135	3.115738581	4.203583951	2.079576474	0.802259196	0.729729965	3.156027609	4.137104392	4.341750671
TCGA-T7-A92I-01A-11R-A37O-07	0	0	1	3	0	0.601757592	3.431673819	0	1.697230435	1.175438996	0.795522389	1.189507153	5.907387812	3.619255126

MIR126	MALAT1	MIR4645	HOXA13	TRG.AS1	HOXA10	NEAT1	HOXA3	MIR570	SNHG1	MIR4668	HOXA9	MIR4701	SNHG5	MIR4768
3.968059001	3.747917597	1.726936923	0.057700495	0.601095375	2.598044821	4.385219424	1.781316517	2.558368131	3.493339112	0.509487556	2.397147302	0	5.547738685	4.250147372
1.41984578	4.401119755	2.23244844	0.013404145	0.562712392	1.875857936	5.096628277	1.195314169	3.925372324	3.821597708	0.746920277	1.449905101	0.461299521	5.915725461	3.75597297
2.870616418	4.697585329	1.112116554	0.092139141	0.831607512	2.672351106	4.70915583	1.304297378	3.868014716	2.951101252	0	2.586228437	1.472334667	5.081580443	1.143209465
0.913750017	4.763012775	2.350001864	0.149674128	0.614679468	2.666023931	3.930992519	2.08355336	3.227355734	2.520275991	1.051964341	3.000892874	1.132650651	4.665911172	2.945081595
3.083799185	6.491135786	0.442472668	0.169904218	0.605941323	2.236603884	5.114242111	1.973354101	3.983321776	2.995602952	1.127285762	2.501452831	0.908726808	4.806064638	0.457838749
3.614074236	5.787539827	2.436552739	0.035703844	0.533488123	2.932615546	5.905134481	2.737160207	2.484991103	4.168001138	0.933782216	3.647081638	0.743540489	5.695407577	0.896134874
2.840147078	5.908209079	3.000190686	0.233453563	0.958681117	1.828277025	4.349100753	2.379318949	2.878033703	3.564380248	1.214248801	2.233227498	1.563573534	5.192928041	1.717883665
3.105025885	5.87361583	2.29198141	0.009788688	0.849653327	2.784029492	5.264307684	2.664962082	4.180660614	3.554105153	0.220187569	3.44317842	2.103564963	4.360040717	0.9529552
2.316511444	6.020108717	1.404950831	0.058781465	1.143130555	1.848689531	5.989973403	2.26651707	3.405178475	3.708529864	0.681942846	1.949679808	0	5.826810209	0.652265262
0.836273631	6.018668711	0.604281598	0	0.094767535	0.03084678	8.077310853	0.013358797	4.516446831	3.016514968	0.466231185	0.037661994	0.710019606	3.429338593	0
3.479387271	4.602312339	1.116038271	0.064823024	0.791918566	2.432724311	4.776336716	1.923491707	5.071370605	3.764429091	0	2.903410891	0.892331633	5.438580346	2.665688361
2.633253736	5.808869049	1.543485433	0.198945765	0.955304469	2.705104041	5.650090683	2.048488342	4.365257102	2.570139216	1.981512021	2.830717064	1.635279267	4.463179618	2.317659004
0.629129572	5.347168604	0.681156393	0.627691867	0.41549747	2.183631054	5.513769844	1.993406777	3.783825541	3.573699007	1.779811059	2.051239973	0.317118332	5.13690264	1.527902503
1.73553827	5.025577248	1.050826398	0.027819922	0.720181484	2.323949393	5.356507981	2.364858488	2.9645743	3.762992846	0.557341598	3.222908303	0.836671679	5.28825331	3.174595301
0.66496703	3.572383149	0	0.721696467	0.647445644	2.906542267	3.304376969	2.117815046	3.402964279	3.291920715	0	3.592600673	0	5.310717513	0.291827718
1.613299092	5.076717826	2.870902513	0.919214324	1.26512407	2.507321645	5.63138298	2.840243312	3.985393759	3.411075305	0.637600901	2.946829554	0.945782377	4.641664944	2.380064296
2.797189021	5.091543926	2.471929031	0.17150128	0.615694592	2.599119126	5.131860838	2.235516859	4.003808242	3.787560827	1.256781898	3.200929178	1.660669848	5.578683274	2.869798591
2.419954674	5.935096591	1.976570439	0.112107499	1.379688286	2.440708494	5.713900611	2.33126972	4.165717952	3.9473798	1.465367732	3.045959275	1.715109897	5.294410684	3.221978343
1.572987384	5.838408607	1.85459443	0.098180621	0.74400662	0.480821006	5.773018017	2.045100475	4.459493242	3.609368292	1.286334348	0.445865612	0.438641244	5.303554544	3.098067208
3.580495836	6.888385951	2.460227632	0.140466171	1.179237559	0.637251549	6.24428017	1.814081157	5.154967577	3.054877016	0.947666956	0.557968127	1.90760408	5.175969874	3.795494255
3.726704648	5.735453557	2.034263171	0.117995367	0.723089364	2.325010152	5.38849661	2.185360204	4.168390335	3.61561611	0.94773379	2.665596408	1.768969697	5.584478516	3.634851908
0.23301847	3.997712295	0.471974998	0.012624013	0.37788826	1.353601218	4.492766533	3.5669726	3.573618705	3.402743287	1.434471041	2.176971008	0.960524272	4.521408268	4.257111581
1.987156434	4.99612224	1.004997069	0.100859443	0.596053923	2.53348489	4.696429671	1.633298228	3.458321372	3.19438712	1.075623921	2.418229905	0	4.857638392	3.453350613
0.937012467	6.00521743	1.817192048	0.239847645	0.565575374	1.894981357	4.922503906	1.904620189	3.352802016	3.981065724	1.310756184	2.428769483	1.61190372	6.227122762	3.121632409
2.201883789	5.836435951	2.267808283	0.051173121	1.166729147	2.032533373	5.731967322	1.551154481	4.317016402	3.25594591	1.262533475	2.39508779	1.649668785	5.138215093	1.408396784
1.772308782	5.436140856	1.157645276	0.246539273	0.726013296	2.878473764	5.187336992	2.501508969	4.190822687	3.180577432	1.89065918	2.782991584	1.587825847	4.807505474	2.80265209
1.471135113	4.876757588	0.407179562	0.049014789	0.241705691	2.251756462	4.745908112	1.033295359	2.533271362	1.224662058	0	1.997299976	0.845679395	4.510226832	3.686598502
0	3.510325795	0.269095896	0.035392061	0.604004413	1.728038985	4.288729592	0.242861749	2.246040748	3.456746359	0.293438355	1.659023056	0.32264305	2.654214293	0
1.084989947	5.625907959	3.763939265	0.285275024	0.931885879	2.092703938	5.064570934	1.963384037	2.97791176	2.61198596	2.96500135	2.241511438	0.589107328	4.073372245	0.515359044
3.261716658	6.054403844	0.480430209	0.123981815	0.509568694	2.679200811	6.115562879	1.850978572	2.766201488	4.456092642	0.520724562	2.908692104	0.78614411	6.202481474	4.175041842
1.728114494	4.372828022	0.301135488	1.822602526	1.009093115	2.243836532	5.663941936	1.678005249	4.811754843	3.556261125	0.595169746	2.677846869	0.648363499	5.436157932	1.427963633
3.515514061	3.854519853	0.91546441	0.025579491	0.453111621	2.592470597	4.693439342	2.017150133	1.741947858	3.387507318	0.405917503	3.287349265	0	6.303266965	3.168570224
0.409433844	5.31797979	0.626101982	0.015738655	0.229251688	1.749641096	5.467803518	2.799696173	3.887837937	3.403801466	0.84556707	2.198965514	0.914964945	5.288254567	1.936248894
1.928379821	4.432907099	3.612311903	0.167502434	0.922366948	1.58630474	3.925422725	2.187674006	2.565161869	3.475790942	0.889010036	1.322602856	0.559032149	5.476525716	2.078413108
3.300932195	4.349882669	3.803564235	0.153944602	0.388513171	2.044484416	4.39337235	2.704390331	1.749206857	3.433279198	0	2.429385627	0.603572966	5.905759531	1.869292873
3.818200148	4.520744496	1.664523628	0.020913774	1.220739398	2.473184413	5.525111851	1.519532038	3.081477687	2.965619186	0.339403291	2.376455175	0	4.756067334	1.002583709
4.397349195	4.776544913	1.520459576	0.275219767	0.74583083	2.551732403	5.70639904	2.035046552	4.149125458	3.708597692	0.666615503	2.554069799	0.98470449	5.865443763	1.807430667
3.086594428	4.364791381	2.837152844	0.151785226	0.881101609	2.747472268	4.847700263	2.336156361	2.883627601	3.253072683	0.333454219	2.664978346	0.900532917	4.970469023	0.988328055
3.86082518	5.340758921	2.179478603	0.035152582	0.388254087	2.534581271	5.034586286	2.710393394	3.557930367	2.911492992	0.675696152	2.833795724	2.110599653	4.750288688	0.646233865
5.244659635	5.051183221	3.368508647	0.299149227	0.548186436	2.945814032	5.667778183	2.911306994	1.898436256	2.722702137	0.622977423	3.06733444	1.765629505	5.039018517	1.929685304
3.905929985	5.055449372	1.764010142	0.225657198	0.302550298	2.960932301	6.025853104	3.08732919	2.875979185	3.906498498	0	3.795036726	0	5.682815261	1.412713096
4.422820041	4.854911975	2.078866888	0.124502743	0.566552217	0.619050422	5.668669623	1.670116343	3.464996097	3.394326434	0.628285891	0.469529296	1.145825795	5.448630691	0
2.796341499	3.921306145	1.157603026	2.361777052	1.020662142	2.552435517	4.570205475	1.855683625	1.480942351	2.436064881	0.745861691	2.51326432	1.002109699	5.739307421	0.714032097
0.30732446	2.227532816	1.208132624	0.072559414	0.730865078	2.545839246	2.868830357	1.965546974	2.042856199	2.780808995	0.365451193	2.381430997	0	5.897863661	1.669757997
1.22312295	4.782336738	1.073734255	0.152528348	1.931775831	1.97731265	4.831261479	2.149840309	1.461597045	3.327336155	0.856182049	2.348042834	1.887838913	3.636773849	2.670185804
0.266700929	1.778515212	0.534360643	0.05758545	0.053266548	2.886990351	2.379100364	0.984053235	2.008942453	2.865172196	0	3.077537601	0	5.948036564	0
1.088715855	2.795809746	0	0.062042156	0.065411709	2.280509178	2.790922978	1.073667417	1.403670142	3.132464977	1.713925617	2.089846863	0	6.138607842	0
0.650280548	2.79911204	1.529030455	0.204488302	0.65470995	2.427844636	2.31339615	1.032414876	0.584022571	3.165250085	0	2.049696219	0.469019622	5.553967961	2.094446908
0.35539371	6.272277409	0.693063498	0.020065021	0.208171994	3.011629478	6.330607107	2.200112768	2.674711824	3.288045588	0.747072986	3.636528908	1.32626144	4.119702093	0
0.639646108	5.77955549	0.864905696	0	0.059500585	0.200702026	6.273674465	0.052016168	3.219042089	3.392236117	0.746318794	0.0947058	1.172898766	3.014029953	0.279326675
0.552878181	5.681934753	1.139406084	0.294362048	0.00921489	2.860130026	7.838994745	0.837268375	4.048840711	2.583018377	0	3.132809387	0.879802087	3.584555936	0.441079577
1.684468554	4.485846214	1.995462559	1.116179128	0.926982582	2.234591573	4.64131882	1.797765623	4.046348307	3.064537053	0.675804968	2.131898012	1.218936618	5.550926004	5.44863462
0.697477378	4.730896865	0.29719357	0.096618394	0.248114653	2.18868254	4.711005084	1.79522243	3.078105421	2.095348921	0.588088832	2.043589633	0	5.249095594	3.911069112
0	1.519984424	0	0.043987494	0.371963665	0.564987507	1.381576325	0.725078075	0.300883974	1.48061292	0	0.391661897	0	6.4288513	1.778967869
1.729972675	5.188515392	0	0.045823357	1.089961794	0.191167786	5.334661068	2.570485321	3.927313074	3.305754576	2.827104257	0.162485994	1.357946522	3.915259921	4.262136421
3.590638911	5.492933491	0	0.556604017	1.087775941	1.94000699	4.742088407	2.45740998	3.279852749	3.143667175	0	2.210114657	0	4.840109807	4.53646215

0.560913343	4.956451949	1.957974061	0.10493718	1.770118724	3.147569458	5.113360767	1.962941626	3.538361731	3.299422374	2.489608751	3.390664417	0	5.356601455	3.817642343
0.319461844	4.602271725	0	0.640835007	0.292144286	2.88522097	4.674512235	3.009966978	2.58391118	1.734594173	1.768853898	3.694074673	0	4.426970278	1.832844652
1.506871463	3.885823051	0.660785305	0.031378409	0.703283331	0.835406043	4.426577097	1.619409748	3.478394575	4.154652591	1.544540642	0.816032506	0.774031236	6.91001627	4.139437073
0.781903807	6.102903772	0.674104234	0.021520549	0.112944424	0.787267343	6.41285325	0.522734862	3.983168191	2.598135861	0.522836718	0.958864814	0.789071985	2.690423718	0.868952142
3.857985656	4.961222915	1.434646853	0.050264288	0.46990178	0.303779588	5.247960974	1.962409116	3.541879576	3.589490363	1.118308177	0.497036089	0.832375576	5.627933951	1.871177482
0.497705387	4.580634529	1.984038804	0.922235975	1.277642589	3.147184498	4.459681314	2.731992189	2.940828561	3.164542773	1.170316837	3.067200688	0.637681827	5.633262142	3.177036273
2.901882912	6.029761733	1.405569767	0.070303005	0.93136017	2.820376487	5.465587113	1.760778106	4.304867336	2.738646705	0.682317474	2.412847232	1.422106523	4.812702406	3.383837489
2.490256934	6.746141439	0	0.046954911	1.845498134	2.384364926	6.071818282	2.264016481	3.090272749	4.249681607	2.072060447	2.924427196	0	4.239201998	5.441046647
0.997648325	5.047730349	0.938787751	0.20848684	1.144261928	0.064482539	5.565524892	0.437398984	2.537019685	4.349082093	1.686585127	0.153004497	0	5.479944487	1.540115718
0.781176489	5.881816571	1.295235987	0.53437749	0.910653547	2.921536542	6.400293367	1.016919474	4.065316404	3.837796362	2.019260626	2.795947189	0.71922584	4.749521394	1.548828443
1.408630779	6.969767842	0.334532863	0.10442841	0.061831101	1.985396323	5.453237651	1.500459836	2.721010993	2.004340486	0.896231433	2.957312457	1.375629376	3.706408236	4.018521746
1.203042764	4.559148684	1.544350615	0.517678811	1.209941908	2.239564993	3.290640285	1.737679105	2.524757931	2.45275272	0.337482793	2.118942064	0	5.03531436	3.550909982
1.485239256	6.094243747	2.799052784	0.163892823	0.935137893	0.634052615	5.11830684	1.710867627	4.65800488	4.377327806	1.671416695	0.55919968	1.777400903	5.352040833	3.799054556
1.872623259	5.796632125	1.977663683	0.212740215	0.949624682	2.756890655	5.3450849	2.87345531	4.272624494	4.425648236	0.547718061	3.179032424	0.597488007	5.744752638	3.147730021
0.391670479	3.820438407	0.426874414	1.481926913	0.640153911	2.81628112	3.475489304	2.443413287	3.474177616	2.591151735	2.010779164	3.311494536	1.634205411	3.163713882	5.070526255
3.431022847	5.856462964	1.680112068	0.163695072	0.829294317	2.831417193	6.048557575	1.995717717	5.658914589	3.866444976	1.145671349	2.946380427	1.776096099	4.564470284	0.297942796
0.651379969	4.346115568	0.274991242	0.045150979	0.476641644	2.724475828	2.182566851	2.020256974	3.481314571	2.305455056	0.299813672	2.285529612	0.329583423	3.607878062	3.148281723
0.667816427	4.145897633	0.217176981	0.010605972	0.034540891	2.401140133	3.269113773	1.959966539	1.839640992	1.674205436	0.237199478	3.134310387	0	2.919908995	1.51574809
3.644010825	6.754312935	1.3900085	0.096970438	1.596758312	0.794979114	6.833995515	2.948465916	5.202057907	3.964300686	2.666449259	1.02639258	2.104884851	5.28282484	2.900133896
2.984527977	7.005455107	0.429704569	0.095298489	0.860014548	2.370933864	6.97427707	1.610523144	2.927911834	3.977461702	1.718052925	3.07793856	0	5.75466427	3.425947532
1.892597912	5.530467797	0.484726733	0.156869768	1.165628333	3.187770623	4.51124268	2.308115529	3.349451843	3.764093351	2.174413752	3.059039264	0.315020547	4.221248021	3.312295392
2.92942975	6.589279269	0.503315945	0.450566854	0.391032887	3.065352063	5.429559017	3.577755787	3.747086387	3.934741124	1.616576732	3.783115156	1.186244453	5.536194431	4.111841409
1.503581139	6.138230963	1.426780692	0.007357024	0.331285873	2.977113496	5.984929858	1.682634161	5.097613252	4.219753423	0	3.106751738	0	5.091045376	1.463053432
1.966628178	5.909824034	2.739957507	0.119024888	0.52350681	2.617121735	6.046090204	2.484796055	4.789911711	3.724927963	2.685610105	3.127847447	0.380577269	6.045776903	0.329974539
1.868298548	5.773047445	2.485092822	2.58424068	1.447030301	2.412657712	5.612191031	2.570833003	3.963665488	4.178117092	0.756857242	2.264437936	1.605905937	5.91051955	2.091731588
2.627289484	4.188374349	2.101455969	0.059303763	0.827935434	1.782262595	4.825090813	1.739331092	3.209590647	3.069348112	1.099858208	1.69077087	1.041694849	5.664012913	1.486743803
1.905218514	5.947019279	2.011108887	0.118566446	1.085858892	3.315660595	5.851424505	2.238190596	3.917921139	3.012009516	2.445024318	2.860267255	0.991212648	4.18598237	1.177291317
2.313566403	6.577122548	0.791284351	0.25305455	2.17887426	1.714335128	5.794384213	1.904434706	3.56390851	3.408150228	1.980687655	2.196786297	0.375909917	4.699005522	4.188398516
0	2.469291982	0.883172312	0.085051117	0.592377461	2.395672676	1.822143283	1.191249805	0.878056267	2.764456976	0.55034375	2.143549414	0	6.15801818	1.212874365
2.556949847	6.629981002	2.102719192	0.125260906	1.332928239	2.20638962	6.262224638	2.61630836	3.606020647	3.481530707	1.212925822	2.923548541	1.301309755	4.670244179	3.939084907
0.216125281	1.626113684	1.169156294	3.071400342	0.414255229	2.94064928	1.184661548	1.669964389	0.359783508	1.088445694	0	2.11546547	0	3.193252709	0
1.418453166	6.276778102	0.692071401	0.182869503	0.736115031	2.522047646	5.65731876	2.979226047	3.429410733	3.822751097	1.89089282	2.631225912	1.324697543	5.451583053	5.716029113
2.511629875	6.503759682	1.449116829	0.066476169	1.146033251	2.226688574	5.920037917	1.779106811	3.679554427	3.687496263	2.016324805	2.325317315	0.508924259	4.833710889	5.643141541
0.848808838	7.064971991	1.074021944	0.120372256	0.141479762	1.885708618	6.358461557	1.019897474	4.705624082	2.519983797	1.43410951	2.478788335	1.531709035	4.636991124	0.75707629
1.519259529	5.559049315	0.754275993	0.403079069	0.168355116	2.456516165	4.153977315	1.425784227	2.154714457	2.19118217	0	2.390245812	0.641178366	3.979640264	1.948043857
1.202432391	6.099403507	1.135573523	0.279591691	1.454601075	3.197008003	4.070659245	2.987540964	3.610329504	2.762691777	0.840007018	2.903359313	1.11799921	3.638544512	2.696124811
2.593434255	5.63513731	0	0.107928799	0.442257793	2.508271438	3.786839086	1.142776913	1.755277398	1.414380171	1.74487935	2.526266394	0	4.91702705	5.576388251
0	2.930205899	0	0.579627662	0.594717242	2.431447169	2.649663346	2.070053201	0.553005297	1.770957512	0	1.93916393	0	4.792369773	2.560383339
4.019592305	6.024803924	1.137500639	1.265098191	0.555648112	2.464089921	4.711449186	1.670562671	3.428126539	2.924659952	0.731181138	2.177384652	1.302559625	3.871194544	3.138163315
0.596955899	5.076566339	0.886476919	0.614534253	0.480844231	2.512642071	4.592826734	1.725394345	3.147233036	2.767845383	0.698083186	2.152050196	0.758301142	4.148147635	4.780931263
3.167369889	6.253898907	2.566873775	0.114254057	0.535522153	2.793619765	6.152977848	2.826924271	6.053903879	4.224273166	0.891758668	3.201254415	2.263617447	5.679896853	3.773723745
1.726407403	5.208036025	1.044081288	0.023012652	0.949083871	1.804396514	5.03614288	1.987865289	3.288056955	2.793036335	2.313848798	2.378774225	1.200419327	4.464645909	0.733844911
2.470461475	6.006697824	0	0.012827243	1.267688763	0.265647567	5.481924281	2.543802936	4.267722701	3.412014114	1.709778152	0.283848617	0.444129414	4.101616696	4.74309014
2.437661836	6.724562757	0.722653057	0.204868967	0.582101138	0.989816721	6.257511135	1.631662654	5.194335093	3.974776035	2.403652722	1.361913805	2.314270041	5.44220227	4.242542264
1.057926063	6.518142841	2.01871116	0.174511861	0.802997812	3.065372459	6.123523476	1.817586153	4.827271742	3.80889861	2.346904611	3.095331731	0.572002146	4.673445749	2.98262847
2.489500522	6.607518178	1.227060412	0.139473536	0.545963696	2.101702715	5.449081916	1.800791363	3.514922125	2.414751748	2.273504125	1.742080532	0.987919814	4.385150065	4.543749187
0.368639809	6.506633414	0.5676176	0.256556895	0.761334877	2.400282006	4.938797408	2.229451805	3.885221572	2.572398969	2.484337509	2.534983203	0.986959027	5.455385103	2.81135018
1.873540231	6.436956866	2.365179124	0.033614419	0.735057563	1.714551467	5.352364489	3.288002037	3.250172477	3.259423597	2.525490215	2.158861037	0.708724671	5.357480738	1.887646439
0.301896428	3.259779224	0	0.169261108	0.3537199	2.788321928	2.413514984	0.83648934	1.014242076	2.821346894	0	2.979028949	0	4.558314326	0.617982705
3.184066838	7.400817369	0.532518195	0.066693639	0.596368553	1.716294032	6.493056202	2.631624286	3.666210618	3.10452957	1.980603552	2.161197934	0.348041584	5.48547469	5.096574188
1.06750379	5.173761909	0.264967473	1.237725261	0.764803761	3.208771145	4.251267778	3.006711725	3.914902087	3.301784735	0.916155137	3.490560458	0.98960684	5.331665274	1.303620652
0	3.836805664	3.378805357	0.253816434	0.692335108	2.926592186	2.966451451	2.735923831	1.270242143	2.28872026	0.355605423	3.594365045	0	3.277658871	2.977232387
1.518879288	4.424263118	1.905361561	0.230478477	0.490889011	1.769731173	3.899711589	1.042531976	3.719265957	3.278322826	1.465731304	1.810327797	1.262096364	5.721473912	1.415103858
2.914172153	6.265442287	1.702826113	0.206211841	0.20336164	2.075104613	5.369497396	2.158470485	4.78121446	3.22786522	1.315305971	2.673085286	1.550705265	5.065080634	3.128373992
1.538011815	4.150360677	0	0.150041181	0.149061372	2.667939052	3.461684476	2.408599031	0.946273937	3.590319974	0	1.550586748	0	5.652253702	5.179285368
1.65097363	6.377577693	2.51744705	0.065957381	0.560144003	1.975798794	5.341838821	1.734278335	5.052012521	3.043920349	2.20125341	2.167693593	1.687720736	4.243166732	2.999574628
0.511250871	4.753022603	2.099731667	0.589418079	0.940391575	3.306477941	4.421461165	3.499956752	2.874883048	4.126589302	1.350075176	3.631958793	1.444345541	4.952557884	2.925011556
1.773626026	5.45219948	2.409749217	0.035454729	0.832924229	1.290703711	5.048917581	1.111546014	3.689720713	3.443542933	0.928918218	1.47815006	0.587017086	4.306247409	0.714789241

0.531400375	2.923532238	1.30731133	1.251329048	1.063975469	2.567286762	4.15509132	2.059729896	1.11817145	3.748607045	0	2.78629245	0	4.131637774	1.016538206
2.266920007	5.626322995	1.634383095	0.079488805	0.417592941	2.475316878	5.076630778	1.520615453	3.196524771	3.018711795	1.020690637	2.594750988	0	5.029505825	1.14834382
1.883368101	5.96626719	1.837108233	0.186290379	0.877845058	1.961319874	5.516240889	2.273951915	3.24548661	3.289273835	0.521472091	2.066886737	1.964507483	5.984268676	2.296571488
1.095401601	6.152820559	2.430792159	0.07996545	0.723482539	2.645326242	6.300793629	2.165269041	3.242884063	3.73156404	2.295980724	2.786866164	1.187808543	6.013373143	2.912360764
0.251005738	3.527718977	3.131240048	1.44434255	0.459926239	3.318514002	2.072521782	2.869068874	1.321201318	3.294540291	0	3.966053075	0.329293961	5.827272337	1.20771205
0.739892413	6.631391653	0	0.042451076	0.089979704	2.968411292	6.297287741	1.05058268	3.204800689	3.72153763	0.346203784	2.971644852	0	2.008524317	0.329469424
0.817886992	4.777796343	0.881332964	0.036323834	1.2279425	2.866405618	4.366948295	2.425081668	3.77616684	3.984491923	0	3.589162175	1.02091433	5.98569081	1.067135062
0.477282787	4.641832538	0	0.904264616	0.774149918	3.255586663	3.455440969	1.302643446	1.767768132	1.930929038	0.308095677	2.779251195	0	4.244840181	2.518352828
0.211764846	3.897176442	1.362202643	0.141498841	0.233536757	2.069523481	3.300127641	1.122076896	1.485984178	1.395914291	0.468809454	2.798781026	0	3.725386591	1.824761186
0.669018334	3.973844079	2.708397339	0.059225187	0.665286912	1.751653593	4.079399757	1.121865502	3.799611036	3.153410727	0	2.354524128	0.483374273	4.081862029	0
0.230099355	3.743210564	1.101169961	0.037043042	0.060331851	2.093244745	2.671710191	1.891493425	1.710552461	1.951717485	0	3.177157769	0	4.308269647	0.261373642
2.492060512	3.926190053	2.993364499	0.176347972	0.536109289	2.194522491	3.584635345	1.681725488	3.523858416	3.115322338	0	2.375544053	0.888358449	5.172089017	0
0.779913117	4.520209105	0.560631125	0.100170481	0.302364839	2.18000732	3.353191162	1.370305741	2.215452971	2.008863114	0	2.821419568	0.471933674	4.034894511	1.106386972
0.28410543	4.167239426	0.310652484	0.076646013	1.209464357	2.199541423	2.527152448	1.620681854	1.223484492	2.11427621	0.338333537	1.929643537	0	4.815322877	1.700476158
0.253787374	4.272874328	0.886950557	0.182697116	0.226770525	2.539990416	3.458847141	1.724457436	1.674013784	1.65343856	1.116189646	3.415015215	0.33284778	4.199458783	1.468523644
0	4.44409687	1.159844479	0.340612633	0.229707845	3.185153845	3.745698695	2.752495783	2.619205518	2.819755201	0	2.66317238	0	5.788756883	0.401732562
0.406685737	3.902095228	0.781518141	0.163149465	0.541746819	2.709358673	3.705504818	1.720195459	1.891747277	2.520665799	0.480695606	2.352631924	0	5.828300078	1.697887112
0.616553747	2.41587114	0.667761907	0.095343474	0.328905955	2.905377612	2.151840819	1.787614834	2.196042698	1.973867545	0	2.569920289	0	5.18760349	0.689271456
0	3.739205364	0	0.061652881	0.109137084	2.124047152	3.072952752	1.929245868	2.336351729	1.880954793	0.315010403	3.326848045	0	4.036716643	0.943599778
0	4.304902616	2.551768137	0.072880948	0.74129284	2.913187287	3.210216657	2.471179777	2.147881497	1.740373877	0.481595706	3.060094941	0.287069809	4.338525487	0.459291287
0.232353961	4.35963379	1.109317834	0.049695216	0.510347493	2.004762525	2.754012704	1.229787607	1.966984123	2.725880738	0.510347493	1.613351181	0	4.673489179	1.140359325
0.296866621	3.574792395	0.324477561	0.111279499	0.013504386	2.028716096	3.278453718	1.647659734	1.486394062	1.368578427	0	3.133739682	0.38764682	3.118935688	1.36435614
0.37761339	2.972589255	0.993367771	0.111036453	0.510840425	3.517058246	2.829644469	2.549602534	2.0517656	1.94505248	0	3.56036589	0	5.587790525	0
0	1.675735191	1.77272522	0	0.536674517	1.495650725	1.958891052	2.994458162	0	1.03768288	0	1.460008316	0	7.119622683	4.96253464
0.791991031	4.053710686	1.884218176	0.222890471	0.657257576	3.040680272	3.363497377	1.908057571	2.455966368	2.935256099	0	2.921297834	0.729382324	5.489749373	1.696223272
0	3.70976058	0.833027411	0.09893461	0.107956279	2.49485908	4.241515711	1.794365102	1.711248803	2.301623336	0	3.181352883	0	5.296864752	1.53467451
0.591768461	4.375179277	1.610000441	0.020235049	0.106394716	2.448156666	4.1788876	2.130941176	2.187412691	1.530906262	1.018819075	3.146492809	0	2.91770957	0.662179707
0.745688624	4.411813399	0	0.053392306	0.128106588	2.337828964	4.736177327	2.282539607	2.471633556	2.504975854	0.349288848	2.594340305	0.383352561	3.895679517	1.736443871
0.41296409	4.201844565	0.631129752	0.054878922	0.188818299	2.058352703	4.165542308	1.669848879	2.49740594	1.838468629	0.26449843	3.18374054	0	3.523680887	1.537751746
0.223811837	3.041588215	1.415355026	0.391011501	0.881846541	2.545189893	2.553986756	2.654648898	2.179290229	2.163087268	0	2.340297971	0.294449791	4.054161452	0.658354701
0.85942747	4.861275804	0.925181136	0.126791803	0.654449687	1.350369746	3.379095343	1.328266846	2.400938609	2.844531443	0.800422575	1.614636075	0.35012471	4.404493228	1.521418104
0.465539927	3.859563185	0.506298488	0.118922508	0.756803832	0.760624082	2.748621972	1.527653419	2.808790676	3.101689525	0	0.55078649	0	5.51990736	0.285369367
0.928659734	3.8680669	1.84526197	0.164527807	0.290592043	2.515849289	3.531767815	1.497101621	1.455129707	1.835161985	0.729678273	2.530256607	0.791947629	4.651218229	1.715096545
1.598750491	4.558074351	0.267355368	0.073684581	0.660633035	2.747059113	3.831900943	1.664437027	2.734808609	1.948271801	0.741469772	2.087575537	1.166213428	4.976060624	2.062744267
2.542165718	9.244004162	0	0.053729128	0.166921089	0.217461998	5.820802635	0.116408967	4.577711323	3.551599133	1.024212352	0.271504019	0.820333258	3.03877789	0.407068073
3.25994158	9.089282879	0.706099491	0	0.939743542	2.413569249	4.855083499	0.987155197	3.861213136	3.52810076	1.256438539	2.665575238	0	5.524576042	2.099268734
1.526669596	3.639666313	0.299524569	0.059243829	0.941563514	2.674077985	4.114112198	1.630427278	2.778469409	3.408254514	0.32632178	3.02993979	0	5.311779183	0.565778551
0.365603052	6.841499125	3.309584037	0.788982352	0.513382066	2.139429487	6.319830793	2.879609467	2.920525868	3.195166171	0.765915541	3.931015588	1.11597516	4.78497081	5.064656421
4.062316504	5.9792036	2.495635396	0.031652627	1.357060409	0.681324801	5.619433626	1.642583659	4.416437695	3.949803959	0.852681384	0.87817268	1.480672026	5.482976719	2.720313375
3.546350441	5.750137677	1.223459303	0.066484584	0.871605533	2.614863228	5.421931029	2.310058892	4.603796566	3.274427084	1.153029981	3.13567858	1.895134149	4.974531932	2.234038144
2.363665171	3.394571351	0.36300246	0.192746303	0.616739895	1.011642816	3.329831811	2.006644348	2.347093264	2.609452459	0	0.818569692	0	4.637063287	0.375955024
2.563217982	7.090022421	0	0.091733886	0.420666024	1.088073206	3.853859714	0.34090549	2.67321498	3.470437893	0	0.520088916	0	4.135970172	0
1.603871648	4.498486975	2.337046104	0.090534325	0.572337734	2.297007842	4.350013343	2.083040186	1.808246755	2.896868345	0.319228537	2.465073306	0.868326436	5.165261314	1.523164463
2.901210234	11.72194634	2.192634604	0.127884901	1.393188095	2.162215474	9.42531847	2.007139709	6.343242806	3.330243462	2.72512878	2.493185653	0	4.119407034	5.19255588
1.18682002	2.7237501	0.304341225	0.160061756	0.243551033	1.303555258	2.732114889	2.474215127	2.350916431	3.150815396	0	1.250429041	0.364062002	4.345749413	0.574103007
1.407249028	4.324801905	0.266233879	0.143159146	0.240457565	2.198753359	3.853971025	1.813560866	1.62826546	2.23450556	0	3.383103186	0.319271887	3.152497476	1.535019187
0.441894638	2.701448614	2.618963876	0.042571219	0.498818939	3.135920748	1.778149808	2.088915382	0.394020754	2.227171307	0	3.215218164	0	3.736741281	0.866569097
1.519500106	4.519701643	0.330684928	0.055197272	0.054394563	2.19864854	3.788111695	2.488309409	3.028094693	1.976846911	0.359938649	3.209664489	0.39490593	2.970267415	1.879917262
0.195442914	3.805660456	1.084771227	0.041376168	0.530956621	2.555002933	4.333567122	1.046369246	2.748625807	1.757137672	0	2.213995324	0.666493898	4.360585816	0.584888295
1.322190102	4.427983484	1.305857913	0.119521234	0.21995685	0.629150539	4.952634697	1.035498821	3.053913462	4.207875284	0	0.86663356	0.535925131	4.975072075	0
0	4.27330749	0	3.098926009	0.523601084	3.297205541	2.698513785	2.072917336	0.615664814	1.61765486	0	1.240990669	0	3.022111108	0.43172848
0.993572185	2.930743823	1.828979401	0.007943668	0.521211621	0.932839023	3.118708098	1.446217605	2.456079988	3.234916191	0.263733437	0.882563111	0	5.032311117	1.219199701
0	3.063191094	1.638555152	0.378833539	0.371861697	2.694694404	3.154580028	2.552561354	2.92257302	2.310216058	0.77850425	3.304151535	0	4.110850249	0.592170348
0	4.1660809	0	0.154082568	0.511959898	0.65129755	3.462469879	2.124464407	2.700959161	3.374806832	0.358841005	0.449390924	0	4.177383862	1.649406342
0.290741473	4.620163743	0	0.200699694	0.908942064	2.808411838	4.449784391	1.923807315	3.007792103	3.298978218	0.625012571	2.961933808	0.379882311	4.575064727	0.329361783
1.997839852	5.88586879	0.55792913	0.015386731	1.579880649	0.492628094	5.997722446	1.819137712	4.621324854	3.364163724	1.027677797	0.84170921	1.449552567	3.985531195	1.888354656
1.295940374	3.206794209	0	0.090665334	0.280557436	2.830349023	4.475429894	1.453172818	3.148039061	2.917279308	0	3.205172215	0	5.989581703	2.716411985
2.297844633	4.497454462	1.390435966	0.085934656	0.636235745	1.960175809	3.928583247	1.919806475	3.628838419	2.749185889	0.375500425	2.101278022	0	4.94696126	2.303657058

1.591803708	2.46538702	1.145896795	0.056283892	0.680767324	2.327168643	2.80955428	1.762521517	2.578768811	3.915075137	0.530776612	3.156807404	0	6.83841743	3.09963735
0.681167214	3.500047007	0	0.043001293	0.276353392	1.834582018	3.867200539	0.99612346	2.525833603	1.562090637	0	3.113370646	0.492704534	3.553286593	3.049270638
0.877885627	5.947561897	1.510670082	0.039839606	0.175432517	2.362905584	6.878839574	1.08144942	4.189837558	2.510105537	0.593202177	3.300182069	0.646256349	3.438727399	0.566669093
1.364257257	6.18213743	1.203834852	0.148661524	0.400522976	2.43532466	5.291120434	1.910073388	3.547496533	4.155581515	1.655715484	2.65070609	0.339741864	5.163251819	3.00141363
0.393654726	4.005258476	1.254137281	0.311710688	0.431525669	1.971046245	3.552402741	1.054874625	1.54811484	1.544427098	0	2.321008181	0.509208577	5.047358195	3.728130345
1.973780958	5.354502767	0.320343883	0.599970763	0.811945026	2.688946551	4.680771114	2.50290933	4.799169504	3.103421054	1.243336858	3.000533247	0.934808224	4.960143438	3.928741838
0	3.206924098	0	0.223922261	0.318595596	2.323686824	2.365014997	0.74604373	1.323702295	1.379957392	0.375787649	2.971208518	0.412086295	4.847546193	3.582206731
0.396713928	3.161810408	2.720329502	0.24505339	0.580210386	1.619165754	2.731845225	1.676312216	1.915905124	2.234710654	0.656718574	1.622154043	0	2.941560731	0
0	2.748234103	1.056937486	0.100274454	0.900134281	2.181785886	3.255579496	1.08976123	2.147599422	2.575755853	0.307675988	1.899282933	0	5.931215585	1.36403492
0.735310859	3.322024611	1.539995739	0.286849661	0.901032639	1.992188912	2.144852588	1.160993068	1.190008063	1.813652998	0.21591559	1.750770212	0	3.91181056	0.68782842
0.210223576	3.630974044	1.027856263	0.044687666	1.284920088	1.760581239	3.924435029	1.580666504	2.39541828	1.359263742	0.465675512	1.832678799	0	4.294357022	0
0.403383765	3.471806656	1.379930129	0.11937633	0.487173199	1.899012758	2.923776799	1.203076067	1.881905359	3.121391821	0	1.88489796	0	5.298648579	0.24522875
0	2.602705393	2.696218587	0.097446478	0.255343574	2.815571724	3.658078485	2.990683212	2.148909601	1.790843679	0	3.307808098	0.435713468	4.066696374	0.67837681
0.242032328	3.404202681	0	0.013152091	0.010806459	2.383380796	4.070216232	2.137542509	1.465017223	1.959548505	0	3.01319975	0.578103345	2.933658852	0
0.295319119	2.86063336	2.090799819	0.452328733	1.308792791	1.904940309	2.824075203	1.740137878	2.132223994	2.686794304	0.351439228	2.052760881	0.385685972	3.034561412	0
0.627912974	3.989863855	1.38144629	0.060090481	0.260966313	3.108250292	2.919788585	1.444855027	1.760997208	1.715053697	0	3.045920985	0	2.65611119	1.299582852
1.360993757	3.521834269	1.449743102	0.188801891	0.918082682	2.715238996	3.406516444	1.844260638	2.976880822	2.611596966	0	2.699873776	0.770164511	4.398889627	1.379396116
1.279542592	5.494732224	1.632853936	0.088988822	1.705940178	2.09908847	6.116561135	2.531416047	2.682804997	3.727334098	0	2.272798039	0.715499207	3.55361428	4.767513908
0.330036365	4.388169489	3.450391283	2.457722265	0.482095017	3.531311314	5.193959142	1.897147938	0.293071049	1.632246862	0.391906423	3.551645406	0	3.481025532	0
2.248197487	5.598441618	1.35203637	0.295417416	0.430166026	2.548093374	6.394043717	2.427000809	4.054205277	4.448841775	1.096708978	2.749188242	0.707162879	6.459119332	0
0	5.008964014	2.780575498	0.146220259	2.269123557	2.28453558	5.559883208	2.034641	3.66126335	3.761283916	1.386588914	2.671997069	1.054083271	4.45587622	0
3.864525081	5.395934474	2.133328632	0.079538907	0.941633021	2.612114639	5.700978765	2.173114816	4.037879285	3.107118494	0.477378935	2.639214844	0.904258463	4.932535046	0.455240536
2.405119814	5.967960653	1.987142526	0.727066356	0.926689873	2.679534581	5.345387837	2.740458397	2.021213892	2.981460435	0.724381506	2.500109896	0.786310282	4.75121152	1.511374742
2.310968627	5.418646531	2.119250453	0.092502167	0.09377011	1.180508336	6.039929098	2.022934456	2.748157052	5.173351025	0	1.64715947	0.394467215	5.259119348	0.618640111
3.209016299	5.461505633	0.982169584	0.161102676	0.341107709	1.954976414	5.745118985	1.355138954	2.358866611	3.38568987	1.47938347	1.688589477	1.372727048	5.934563652	3.292263556
0.795679678	4.879371148	0	0.198599924	0.381738285	3.261342778	3.227146132	1.455848782	1.83972815	3.053084536	0	2.269148632	0	5.493739588	2.533550174
0.569646829	5.973955414	1.767689344	0.051597583	1.201868809	0.334439574	5.779942525	1.642338645	3.686992888	2.615361447	1.744865165	0.259428903	0.985389698	5.280000803	0.637962556
2.049332886	5.72654393	0.616472107	0.122717394	0.732035045	2.709821696	5.165626478	2.486606446	4.442066367	3.379912964	1.742323152	3.476440532	1.203630971	5.403091235	3.4205977
0.528128607	4.621005879	0	0.198818592	0.274118243	2.757541266	2.898320206	1.042805014	2.683287415	2.501272556	1.052115367	2.565256012	0.376527482	5.026669879	3.341962381
0.780444518	4.673624954	1.369854066	1.196195278	0.919254627	2.258458621	3.298675644	1.567752918	1.174888743	2.055252177	0.367909394	1.775428118	0	3.671983564	5.954221965
1.382661418	5.575300861	1.704188628	0.137679353	0.813871558	2.22832858	5.702703633	1.789838847	5.09991657	3.46255676	2.850150266	2.467658218	0.479575806	4.713379502	3.124572781
0.727709448	4.4824411	0	0.124922776	0.672706215	3.08847298	3.63536972	1.790942997	1.953921104	2.232754309	1.995368112	2.861606461	0.528616253	5.282027083	0.248996973
0.796464682	5.741751874	1.694583858	0.013263361	1.641225295	2.409407256	5.478687648	1.961431926	2.236549259	2.920285411	1.227803319	2.666810604	0.803732669	4.863727973	1.182409477
0.709039233	4.658247836	0.951746466	0.117511051	0.481555937	3.091580248	3.386240121	1.926897771	3.280021017	1.922724328	0.598190809	2.604591172	0.362277856	5.001949178	1.297560811
0	2.163976128	0.254483896	0.10950127	0.060959465	1.164897704	2.781533729	0.588461036	1.58050274	4.161773646	0	0.982496022	0	5.639126105	1.381746521
2.900248571	6.031791939	0.532148338	0.080527563	0.899131883	3.330170701	6.027530623	3.074238483	4.452346709	3.419907774	1.305717969	3.883148607	1.240635012	4.799880153	0.76234712
1.823260059	7.119931911	1.737892578	0.025282691	0.538880596	0.384736844	6.782075304	2.674292388	4.368188969	3.716111516	2.424207216	0.388160701	0.651585914	3.735432835	3.961710699
3.99190243	4.935969428	1.488792468	0.055898042	0.869525412	2.473262297	4.920417228	2.102680973	3.694933378	3.505045794	1.21710399	2.777756616	1.154883071	5.321309436	2.941792628
2.673272571	5.232179384	0.730949097	0.088491293	0.79743118	3.711022783	5.041012402	3.838687214	3.088551988	4.140413797	0	4.507563343	1.228908364	5.45762142	2.593153093
0.879327366	6.685263392	1.390554931	1.15553317	0.491635381	1.794430834	5.999959298	2.504188367	4.584322963	3.970965712	2.020854954	2.125316972	1.092669054	5.323758521	4.392966129
0.191828459	4.647064226	1.447230519	0.731810637	0.624304058	1.461698801	1.783158257	1.97376911	1.456618701	1.522579619	0.229814415	0.40842809	0	5.139982985	3.366103868
3.807998722	6.934585949	0.742446101	0.048185499	0.550174561	2.520806697	6.488786948	2.119578456	4.163318456	3.375373761	1.793980535	2.499150253	2.569346946	5.410757077	5.338064522
2.518418594	5.696702828	1.083225666	1.380416704	0.867031623	1.946529923	5.27264774	1.895610533	5.13107959	3.495059719	0.864279196	2.479970408	1.713243571	4.688975914	3.366390507
0.289200308	3.314016973	0	0.451273545	0.717317294	2.287856498	1.734926302	0.694028455	1.650552208	2.369937817	0	2.007531285	0	5.036134729	2.608606843
0.805574594	6.194296533	1.514735405	0.161867332	0.785254734	1.713979525	4.194999656	1.308146508	3.659013864	2.306706548	2.409647423	1.658238933	0.589280693	4.758466489	4.754084467
1.436400528	6.387308614	2.253227495	0.060432916	2.207087368	1.807070755	5.828997107	2.121568441	4.515344036	3.466007728	2.362955207	2.528743231	1.591945699	4.916247538	3.465394403
2.380005487	6.530136598	0.695080478	0.162829558	0.595173418	2.54772247	5.038139855	1.24303682	3.500594653	2.960222023	1.239852858	2.647835429	1.09409215	4.062336242	3.43590081
2.119116462	6.384068018	0.864244144	0.112008829	0.490904988	2.252774146	6.053348017	1.827388117	4.82536984	3.408153122	2.132277724	2.676043759	0.809019415	5.095744868	2.210385555
2.548351687	6.794792335	0.980658945	0.309718879	0.846786547	2.903031294	6.512957026	2.571248956	3.413919655	4.000073897	2.163429606	3.119217009	1.756741683	5.291506195	2.716014215
1.566345101	6.261675856	0.837856883	0.305020372	0.150691676	2.302599902	5.118579328	3.411616639	4.292417863	4.957379085	1.931656583	3.186117418	1.548307618	5.783349774	4.556499583
0.490761986	4.799530123	0.740648089	0.229807458	0.951580363	2.080160449	4.013888752	0.98535289	2.740057653	3.762992636	0.988430364	2.372566264	0	5.294161785	1.394492557
0.349298162	4.872616238	4.879009647	0.726560321	1.350752661	1.483604759	5.021173367	2.111112504	2.651023094	3.957116308	2.412605454	0.66378916	1.509955675	3.274352166	1.814465616
4.412112623	7.141038259	0.296960069	0.213091189	0.38133007	1.516508077	6.71230981	2.420251814	5.429329644	4.112759124	2.91391672	1.888173828	2.211464826	5.423718478	5.139382878
0.683458333	3.562136604	0.919855534	0.483725796	0.518391385	1.99713119	3.415640924	1.529081946	2.657680474	2.835757061	0	1.515628684	0.347702949	3.625015257	4.027985797
0.2533252	2.321186599	1.735884449	0.032038694	0.400533742	2.902808133	1.719831914	1.215613399	1.816833228	3.022202374	0.302270648	2.539921583	0	6.839185475	2.178795011
0.500267112	3.49458776	1.246477055	0.29292956	0.432686874	2.694693362	2.866593641	1.835541971	0.446997362	2.992730071	1.004740959	2.320560122	0	4.7218563	2.265644852
1.390482991	5.75488976	1.950184078	0.105525198	0.549838346	3.128532399	4.730757592	3.145549808	4.207804382	2.296344082	1.364757917	3.492210089	0	4.116006229	1.693884815
1.950927065	6.053455093	1.315537301	1.374085933	0.587990488	2.917014704	6.035374559	2.915177173	3.992960505	3.459999325	1.399359849	3.678592976	0.295697739	5.543182567	0.47230916

1.732510981	4.244037903	1.51854921	0.575889865	0.137462418	2.365918222	4.066182853	1.180986816	2.492242652	2.948533026	1.19042947	2.012314003	1.582336222	5.159163906	3.866360095
0.202057774	3.334067594	1.73266204	1.645292509	0.226726454	2.887803729	2.196187898	1.82145642	0.610660188	2.646906742	0.630088294	1.375570007	0.266441067	5.879016241	3.104028248
1.30482827	5.850092517	3.340735114	0.073443371	1.930131962	2.269896901	5.528804875	2.587610474	4.21392671	3.635429269	1.478018961	2.701308816	0.802420574	4.357150983	3.940887277
4.036421478	6.234271474	0.863747855	0.099446407	0.434587154	2.033784122	5.338657054	1.635627031	4.136994641	2.910029493	1.366273071	2.28417469	2.089588723	5.305093612	5.404976806
0.954863319	5.484086777	1.51833622	0.770392933	1.465382634	1.603983603	4.692844211	1.384919665	1.79365341	2.919582922	0.296188569	2.002618406	0.815291689	4.196036864	2.081906035
0.483624794	4.013205284	3.15596398	1.639948834	0.312428298	3.523753857	3.181450774	2.650528231	1.854945312	2.723746021	0	2.995247496	0	5.974280676	2.888368988
0.889200191	2.881344766	1.121582418	0.312789266	0.222325654	1.659516823	2.837098886	0.489247006	1.084127418	3.326939811	0	1.301397486	0	5.676864263	3.431382051
2.706617843	5.083259735	0	0.240274149	0.510405481	2.213135188	4.455953912	2.415603137	2.350156625	2.467912132	1.349945812	1.670691177	0.654172598	4.989837383	3.057705344
1.87859426	3.746384089	2.415341979	0.087651778	0.552382322	1.337124898	3.774960892	1.161319748	1.44380717	3.089599968	0.519487462	0.972047901	0	6.026459015	1.157075533
2.115603442	4.681487906	1.036643433	0.045215138	1.293160617	1.530658361	3.617081035	2.129271928	3.158535413	2.947566328	0	1.103893156	0.973668901	4.881876924	3.673704745
0	3.146214074	0.815526278	0.084611615	0.628057211	2.757356823	2.965861048	1.603307076	1.705841982	2.037083711	0	2.473607406	0	3.705711751	0.991633784
0	4.943276738	0.592879119	0.275392543	0.628955068	2.846705201	2.754012265	1.37323389	1.985026819	1.700708623	1.082810299	2.677253661	0	3.351524096	1.269206461
0.711244556	4.56772753	0	0.838397474	0.404351404	2.258300312	2.746923509	0.970974839	2.181560127	2.526962082	0.826812548	1.903749584	0	5.583321889	2.43071959
2.328715028	4.761866552	2.676579958	0.370714055	0.978236985	2.554968528	3.856633717	1.994257759	3.501515719	2.634516433	0	2.429577327	0	5.332127055	3.986901327
0.320465332	3.555313346	2.102627246	0.029678791	0.397683447	3.166553133	2.861989355	1.807014505	1.063372233	2.190021778	0.681733931	3.039887297	0.417479883	4.889535324	0
0.199372763	5.321749694	1.56713468	0.126498454	0.453188072	2.292095837	2.730048499	1.148596119	2.212950281	2.110002476	0.443513266	2.264619282	0	4.767645479	2.610430545
2.432928347	6.478158823	1.114822408	0.156670855	0.636166216	2.447630465	5.834746217	2.621901653	4.010227308	2.532913402	1.547636262	2.684310176	1.277820693	4.794391402	4.513044517
1.993663707	5.190372891	1.360521511	0.183064274	0.340267288	1.758631382	4.095531445	1.038519726	2.901404973	2.977834691	0.42751131	1.410310709	0.468046354	5.116010815	1.976880224
0	5.259847353	0.615008883	0.351973101	0.655181068	2.104805129	2.800894133	0.665549792	1.700654898	1.750494653	0.256899613	1.410117014	0.28281282	3.497447911	0.244122881
1.926914895	6.312623014	0.867858897	0.22479054	0.937448323	0.638680102	5.288106976	2.357026611	4.498911614	3.387120479	0.2949729	0.745294725	1.708681361	4.118926086	2.148474056
2.837209101	6.029274848	0.41248126	0.248419366	0.804592655	2.77764852	5.34657708	1.86379424	4.100806408	3.417159709	1.185388245	2.989366769	0.855228303	6.379875724	0.600827473
1.552903668	4.479086371	1.451381633	0.253286508	0.599300142	2.55877668	3.722860293	1.742257615	2.793233148	2.67621826	0.526415887	2.735103363	0	5.087985773	0
1.479977322	4.806879401	0.21985101	0.210397973	0.524942086	3.508542261	5.123738087	3.004756517	4.308654178	2.41405145	1.061048848	3.891685016	1.004300183	5.009716965	1.136852682
2.976110021	7.186391925	1.115365125	0.021077324	0.498983218	2.824098549	6.076765653	2.491551218	4.759254945	3.12830949	1.918445144	3.035773771	1.853524781	5.602403244	3.670533522
0.324262053	4.902819131	0.87548478	0.082658229	0.672564447	2.581859339	3.55326731	2.194393216	2.249256007	2.034551872	0.688903596	2.724288364	0	3.630606235	0
1.987769187	5.190110731	1.630230192	0.017063287	0.685304834	0.983085324	4.431157799	1.666071172	3.401266573	2.619325739	0.51825556	0.713055714	1.189450776	5.46218476	1.398041767
1.916018818	6.567495726	1.153672919	0.158198996	0.835891071	2.870367463	6.08505927	2.635205368	3.113792502	3.784223897	2.181610773	3.507907319	1.320180008	5.550783625	1.691662316
4.063561941	6.247446944	0.519894146	0.486114262	0.899557767	2.271120219	6.211408806	1.826700404	5.637769426	3.627008339	2.568379667	2.093635343	3.043852352	4.915608092	2.50204159
0.803830947	6.856832285	2.156227766	0.093966428	0.744428531	2.60208913	4.836622056	2.812228831	5.04528082	3.378519588	1.665971215	2.80686108	1.590860162	5.309390172	0.228130946
0	5.400001554	0.803998171	1.023838008	1.953020055	1.639210107	4.634735921	0.710990267	4.878840728	4.039907476	1.284467324	0.36644838	1.713361329	5.06988746	0.97835444
1.910251497	6.11246962	1.599434969	0.096475746	0.771100882	1.900762465	5.67106295	2.343472166	3.664981133	3.487295719	0.639989372	2.031410364	1.799672519	5.602079796	0.611775159
2.799171942	6.222475712	1.089459974	0.1029922	0.875126201	2.786643969	5.217943895	2.883702369	4.619312453	3.551094066	0.696322415	3.898802963	1.504940906	5.023496403	0.257749108
1.566122155	7.667097322	1.577452256	0.023450248	1.948195325	2.775192062	6.37675344	3.327098277	4.375901504	3.394490582	2.476167526	3.041261648	1.466722105	5.379683505	3.85952562
3.208164117	8.260628986	1.569072942	0.976660969	1.462491632	2.126272942	6.567200641	2.244125105	4.721104137	4.470660038	2.259044733	3.226098607	0.927218193	3.296176483	4.951203042
0.698396563	4.830520204	0	0.106103465	0.649082002	2.583190022	4.916917528	1.488993035	2.37390798	2.905295311	1.005889612	2.546059578	0	5.077263671	0
0.729998556	6.215624568	0.661433882	0.173366797	0.500596139	2.703843917	6.122754753	1.773265098	4.475043343	3.032865822	0.713536774	2.485993271	0.917346054	5.275835278	0
1.311903179	6.41269866	0.408545337	0.119991198	0.928469009	3.166040445	6.295795917	2.583312249	4.052342504	3.151399445	2.459698687	3.329064407	2.26329282	5.211728995	1.961715766
2.2558039	5.568442851	0	0.243160773	0.746944231	2.120736053	4.557287644	1.782166539	1.890351939	2.121703727	0.874728608	2.100860888	0	3.552816727	3.46658185
4.127857429	7.120981948	0.540900313	0.12825445	1.089463288	0.752765554	6.777333299	2.726987504	3.750351363	3.093362507	1.460107471	0.813448952	1.415701285	4.832147074	1.409594234
1.481363658	5.9750785	1.498833757	0.036106788	0.347522416	2.229237278	5.708847042	1.049250777	3.998139666	3.063410744	0.587066284	2.171487908	1.202869867	5.816047751	0.212068576
2.168997696	5.746033298	2.800359766	0.034088285	0.350283714	0.692412965	4.598701316	1.111700557	2.942257493	2.700508769	0.470973521	0.536583991	1.441352321	5.651578006	0.241948261
1.491611658	6.038999315	1.502636698	0.091685635	0.886299631	1.7068292	5.623529037	1.90162941	2.953899982	3.016892131	2.222557996	1.819566872	0.493101015	5.662629646	1.146747291
1.432082077	6.59020405	1.523467321	0.23005998	0.791487944	2.96623984	4.61966146	2.797487595	2.956945693	3.0650496	0.643803952	3.059220777	1.297594468	5.125499011	1.376358867
0.288719452	5.230749074	0	0.574114959	0.910773268	1.255131257	4.446041361	0.823531811	6.220747112	3.0977736	0.85367248	1.108637492	0.676130415	4.146414587	3.152049273
0.289665227	3.338039699	0.576200111	0.118321613	0.231310952	2.327013128	2.149543264	2.019684276	2.108499586	4.05610855	0.856000325	2.051320599	0	6.823391234	0.820672267
0.238531822	3.414589173	1.371708498	0.080099125	0.570210613	2.839774359	2.897372018	1.577212205	2.349444191	3.001888194	0.52269488	2.423363239	0.313333776	6.116941712	1.023316296
0.641202369	4.52295457	0	0.803315994	0.299075267	1.953965345	3.40161238	1.647117649	1.917970535	1.883279417	1.238235132	1.94007241	0.588199637	5.192417074	1.440514916
0.645107977	5.638444747	1.166636789	0.08790157	0.451525067	1.941132396	4.261426289	2.235774666	3.127784173	2.118760264	0.542461248	2.054517234	0.816197077	5.124042171	4.87192803
0	3.05541369	0	0.367992286	0.416885819	0.851624685	2.863503643	2.032738498	0.501303362	1.083737235	0.468672201	0.668919853	0.278867119	5.409552604	0.627262329
0	3.500064542	0	0.036238418	0.606042518	1.909592408	4.300594534	2.158697485	1.753202511	2.829451271	0.4964128	2.167612791	0	4.282022781	0.473528698
0	2.804729254	0	0.107644723	0.322417875	2.612352398	2.091823047	1.969593734	1.428916676	3.175288989	0.303955433	3.059782901	0.33409082	5.260118365	0.289064199
1.078393976	4.446994371	1.442575778	0.10840012	0.781084734	3.020028991	4.518283378	2.517742206	4.15019286	2.956511791	0	2.818043384	0.925949823	5.564993724	0.595287612
0.786122608	3.463607019	1.765152128	0.034517548	0.488063085	0.791507874	3.694987666	1.11626721	1.429125625	2.790423754	0	0.376468437	0	5.55399386	2.514840602
1.71919863	5.641852084	0.396532186	0.00689295	0.249423092	1.74235004	4.755369429	0.908277993	2.863518591	2.280557738	0.430792683	1.657715102	0	4.458401856	5.495313244
1.326618547	4.544069537	0.699828491	0.040339066	0.520597396	3.096201042	4.591693091	2.542642093	2.652203777	2.814030317	0.543821076	3.496224381	1.012494886	4.631316963	0.282784396
0	3.676373853	1.968845662	0.186362227	0.580959359	2.880533149	3.47360738	1.708025512	1.451650329	2.868608149	0.849199169	2.585229377	0.531347159	6.019617781	0.463714669
0.962551367	4.687494892	1.802150481	0.167209686	0.359670442	1.475682399	4.213622819	1.415119294	2.955122284	2.414600336	0.194096103	1.429723281	0.214134723	4.93916418	0
0.753646903	5.125364094	0.324744325	1.406564494	0.988171941	1.982638993	3.778531254	1.612808797	2.850432712	2.558406902	0	2.09952045	0	3.398501947	3.899355531

0.511287682	3.844377002	1.809027107	0.259013862	0.511982439	0.837295875	3.19640449	1.575995226	3.220908417	3.341636982	0	0.802378379	0	5.831391571	0.983379004
0.739307	3.745173821	1.842462708	0.27804642	0.381927945	2.685571874	2.871636235	1.781513606	1.539201038	1.880190849	0.492012615	2.553300258	0	3.946034475	1.106557973
0.490256272	4.171428921	1.358166619	0.121579294	0.84766814	1.417005017	3.896769577	2.512302873	1.87173256	2.945600725	0.576681369	0.664358015	0.34824161	5.803137186	2.717697208
2.36682499	6.015326316	0.794443604	0.312468374	1.661472671	2.659947843	4.91172015	2.51631997	5.600305187	3.789076648	1.145203829	3.274496883	1.884727085	5.789545821	2.942343624
0	4.293987341	0.199762907	0.124242136	0.520754323	1.900376212	3.797728661	0.91405333	1.642471944	1.505328748	0.407865127	1.466257875	0	3.650537357	1.51316883
0.684459246	4.130314526	0	0.043258446	0.232571088	2.26938909	3.660838111	1.34989888	2.112688458	2.223957386	0	2.148233889	0.268764483	5.770902171	1.707687354
2.887536988	5.162471207	0	0.105554734	0.638421219	2.941906508	5.568080015	2.842815672	4.111211767	2.976861364	1.587628439	3.503529544	1.560636263	4.396062919	1.128103954
1.532296302	6.018745687	1.480594987	1.412899923	1.01309465	3.168412114	6.235973799	3.661068705	4.36898999	3.556795139	1.209849172	4.207858422	1.672903919	5.09467001	2.500312208
1.727336803	5.613265368	1.828339404	0.647549723	0.983860588	3.421635288	4.152794281	3.508625711	2.822879072	3.381322384	1.521522773	3.482225512	1.02815231	3.590556679	1.307334938
0.375732187	5.769268038	1.983918721	1.261300317	0.477015785	2.540022992	5.462512808	3.109106206	4.079150693	3.354773139	2.205540878	2.463749921	0.486854425	3.965852342	3.657468344
0.702970685	5.445837581	0.429259659	0.137088684	1.10002894	2.134338245	5.398430606	1.75949623	3.711932697	3.980396086	0.817535902	2.277901788	0.509495015	6.189830951	0.444233894
0.524873721	5.170019753	1.294661398	0.188196121	1.640530537	1.750389927	4.796659981	1.746772321	3.795090755	3.322921611	2.158157967	1.866899105	1.473073125	4.767224162	3.097701295
2.73950597	5.56186813	0.702070714	0.155595028	0.989566206	2.734478467	5.986588152	2.01085247	3.748442872	2.8849312	1.250381891	2.66527283	0.595271625	4.608924949	2.531860578
0.281195918	2.800568436	1.129482292	0.16180618	0.465610922	0.639322325	2.051269267	1.064711707	1.3269004	1.473834762	0	0.670679679	0	4.139361721	1.574116094
0.983604567	4.589360638	2.67688547	0.292315308	0.918486937	1.883372691	4.458390499	1.959489111	2.279227455	2.920168326	1.31240566	1.56612922	0.730624293	5.621242577	3.278109547
0.573614068	2.985067509	0.621968909	0.331585167	1.250971989	1.413793868	2.229796869	1.353279363	1.998165776	1.997960078	0.671629849	0.970180404	0	5.242102058	1.207012075
0.722620733	2.845218969	1.419376113	0.292270879	0.135048875	2.169437391	3.438070283	1.732499838	1.535626141	3.767605815	0	2.040494818	0	6.272845125	1.166527863
0.455422613	3.732130962	1.017474827	0.20800014	0.311085407	0.59924351	2.607115295	1.551246284	1.300821385	2.087743997	0.536773001	0.475715342	0	4.841275002	1.046771248
0.462906592	5.167925727	1.417102233	0.777751062	0.892568147	2.657382398	3.773933807	2.076789061	2.93173578	3.468916961	0.940179691	2.599003386	0.820190681	5.883930416	3.176197185
1.581820862	6.001629074	0.703755932	1.245323165	1.142343565	2.358092276	4.907582077	3.056459007	2.805133617	3.458881188	0.428471271	2.700040564	1.724815436	5.49084127	5.919693781
3.209836934	6.692426831	0	0.045696313	0.346817019	1.675823262	7.067725078	1.394529011	5.206199059	4.499541595	3.810144556	1.857816015	1.079584343	5.132456985	4.864015597
0.891889652	4.570508763	2.737036487	0.30838683	1.036108353	2.75731149	4.417480574	1.755228361	4.228376071	3.685867904	1.449736512	2.79295551	0	4.804287677	1.719479446
1.518811877	6.714537715	0.247582104	0.861001588	1.089104496	2.593577578	5.126299232	2.113603653	3.265983429	3.080847758	1.795454252	2.870008864	1.61323599	4.894503006	3.476367245
1.602352706	4.924732203	0.292540625	0.197037955	1.086250037	2.751452403	4.202683691	1.753467155	2.449203033	2.625422574	0.800599198	2.616269627	0.63180235	5.811988834	3.044598103
1.965804243	5.680490687	1.502478336	0.230781552	1.355840489	2.70838824	4.855897403	2.441986056	4.008868354	2.590263067	1.708715438	2.860447317	0.356240727	3.370244793	3.637572705
1.737383524	5.239140527	1.194346713	1.794481227	1.840188163	2.788020356	4.926983626	1.725624527	1.859951291	2.914399201	0.49049767	2.700981533	0.535974888	4.687846184	2.272146717
0.880232413	4.846980353	1.079714107	0.275896797	1.352598199	1.8349531	4.243202673	1.06254815	2.874870139	2.556248057	1.698571739	1.434705266	0.748928178	4.911175293	1.551527367
1.303265458	5.303371322	1.009156429	0.320062501	0.50888003	1.715678706	4.78486376	1.682297931	2.862073943	3.210849839	1.3559241	2.66903439	0.992934205	5.563679491	3.554424885
2.268707712	5.263097802	1.002691039	0.230493113	1.174764127	2.892524117	4.448418606	2.620050314	3.764378168	3.189639912	0.733323512	3.204955789	0.986541165	5.740039229	2.372312462
1.768074526	6.64750101	0.845421122	0.389146881	0.960358406	1.827322953	6.08024501	2.313390811	4.681876861	3.878742587	1.60648589	1.991862997	0.405576152	5.487324127	3.777444442
0.839101986	6.076538336	0.28418945	0.432324484	1.730252333	0.804228694	5.14823331	2.299985259	4.573743192	3.663577484	0.781146093	0.676048832	0.340402129	5.181654603	2.344245497
0.386844034	5.449968452	0.226188607	0.065236554	0.699051401	0.56167197	5.137060432	2.292889876	4.711321511	3.253892633	1.853598458	0.371188055	0.271976241	5.794835075	0.613405158
0	3.199972393	0.425691977	0.256836843	0.501751977	1.9666831	3.018639083	1.525090083	2.422018947	3.178931493	0.462092402	2.110970879	0.27470117	4.926115599	0.618892839
2.976695271	6.370799364	0.345873362	0.052297076	0.510241372	2.936824297	4.63308278	2.978073449	4.23649303	3.254301137	1.316180905	3.320136537	1.867499879	5.869762804	2.462171181
2.290038576	6.252664434	0.778898159	0.0423675	1.677494617	0.505931303	5.643010431	1.758754185	4.423323696	3.095946004	1.12505783	0.476362397	0.727849264	5.974634989	4.088654925
1.754956129	3.758126893	0.682628019	0.142501832	0.552153643	1.934938184	3.029094922	2.110378468	2.015047324	2.834255194	0.529782285	2.418933534	0.989883882	5.011172412	1.421644856
0.690506032	2.877951826	2.057365325	0.503042139	0.96476028	2.556830484	1.768239625	2.086594719	2.139718053	3.116505519	0	2.345548182	0	3.982111854	0.769854136
0.401915388	3.8722606	0.437908086	0.071771357	0.037660022	2.200000295	3.687155579	2.106741754	2.034458814	1.644574987	0	3.04014179	0.28300151	3.797119754	0.453139217
0.366182417	3.55040056	1.692739452	0.133007971	0.910813477	2.177795921	2.902621509	1.626447123	2.538702544	2.03540802	0	2.266291054	0	4.558532771	1.218812851
0.9855408	3.929278877	1.057886459	0.920420948	0.593887676	2.076953356	2.447780438	1.171633444	2.516888296	2.023702798	0.375753241	1.653145178	0	5.01873028	3.195601158
0	3.35399494	0.62159105	1.176165907	0.502542356	2.613065515	2.949357058	2.962454177	2.072600086	2.50202999	0.374303996	2.863405856	0	5.311134022	2.214943069
0.942339878	4.862959641	0.267211553	0.131587552	0.184689934	2.420448501	3.919257852	1.645515282	2.586710368	1.675443947	0.533717699	3.202230245	0.320423621	3.853844565	2.500299937
0.318030802	4.222172313	0.347383867	0.351808245	0.962873711	3.316622828	3.527323459	1.755222164	2.817885228	3.943789342	0	3.276888608	0	5.729029621	2.312096407
0.311120377	4.075410351	0.339907651	2.652881434	0.919598821	3.109705396	2.384666277	2.691241366	1.038777167	1.724154992	0	2.856076912	0.721966837	3.418732451	2.363645752
0.256994601	3.622555907	2.004836214	0.130275919	0.148416388	2.930921263	2.240111213	2.840418541	2.574119733	2.39456811	0	3.615983776	0.839340478	4.747883622	0.922871449
0	3.943412325	1.086002954	0.019487872	0.384761343	2.059114849	3.741199064	1.740363489	2.819888652	2.299374844	1.688513635	2.620427281	0	4.13129916	1.520746476
0.262709724	4.964188812	1.950936695	4.389880207	0.146495298	2.888004414	2.594037019	2.235737124	2.47263041	1.742381371	0.313279811	2.536884493	0	3.693069656	0.297977341
0.309884835	4.812091133	1.215688404	0.338333281	0.241794246	2.052463538	3.966231712	1.210949177	2.256828197	3.293674744	0.368437006	3.004769728	0	4.070693549	2.639069801
0.331973473	3.556594947	2.864639948	1.247967021	0.610868616	3.221794028	2.196535822	1.739494533	0.539482822	2.733427041	0	2.978755712	0	5.127174581	2.666643185
0.573124292	4.627756695	2.714495301	0.123885324	0.790311451	2.670038001	3.662376785	1.88213641	2.047638415	2.605199705	1.252134328	2.679510141	0.729500138	5.224706947	0.951393967
0.593581554	3.570042075	0.35719734	0.200207455	0.255558231	2.120864901	3.060192125	1.618125581	2.765167236	1.756424912	0.946420737	2.638693375	0	4.493861844	0.908407871
0.48309224	4.183965313	0	0.25832835	0.630827038	2.879931692	2.789007004	1.910626383	0.606724352	1.716439018	0	2.985501924	0	5.121831392	1.245238809
0	4.021369441	0	1.201476823	1.471624339	2.258400189	3.272311149	1.882643955	1.305012181	3.080283166	0.747942781	3.094509934	0	3.381861007	1.050411434
0.196945153	3.420384195	1.55463018	0.092210219	0.171764804	2.18323141	3.02698572	1.337651828	1.34373883	1.645429941	0.235864131	3.176468033	0	4.485378125	1.882908994
0.428863609	3.577495552	2.942501188	0.241524144	0.918827156	2.628366179	3.049785494	2.070889111	0.814237234	2.563633745	0.880258718	2.49905785	0.302791947	5.138825308	1.373243762
0.434615913	2.85956181	1.648196925	0.054069832	0.717159947	2.346782942	2.201080554	1.354980015	1.855396172	2.302793669	0.279104743	2.115850052	0	6.113410969	1.271025135
1.539159819	3.188314133	0.233101089	0.089113891	0.546941644	2.327008715	3.544448693	1.055684557	3.042384691	1.613486517	0.254466518	1.957611651	0	3.84180269	1.29850732
1.33990395	2.992996456	1.210881855	0.106720834	0.402909337	2.882639144	3.001910476	1.794381735	3.247730034	2.230413946	0	2.502056821	0	5.281165837	1.562874373

1.117080591	3.786240733	0.452832891	0.031860129	0.444575146	0.823527367	3.826629539	1.766955772	2.648140937	3.218380009	0.266395448	0.904365072	0.536713614	5.418861583	0.253186689
0	2.648684431	1.292557596	0.166425238	0.268591888	2.325771475	1.661845217	1.645848187	1.649416678	1.828428448	0.296072438	2.255368359	0	4.504896219	0
0.278995094	3.66405281	0.556827865	0.04556949	0.26541838	2.610526284	2.924956785	1.601293639	1.611886509	1.672277112	0.829607273	3.108937752	0	4.574156393	2.435933557
0.571535037	3.478968753	1.051870796	0.057443575	0.197530222	2.597633293	4.194472472	1.653622006	2.577638969	3.065946693	0	2.527391802	0.906484831	4.824458482	0.802799979
0	4.136106666	0.254533272	0.125064694	0.095384356	2.150150981	2.865302962	1.568853875	1.652751776	1.473034635	0	3.273078426	0.305477346	4.397001526	1.140537995
0	2.846928941	0.259578739	0.079572204	0.981086461	0.474039973	2.48601542	1.38652453	1.60135945	2.241863091	0.283141863	0.4915018	0.311428205	4.939680755	0.691857911
0.697847961	1.913864161	1.991233105	0.546872971	0.468943022	1.157993669	1.143566466	1.555923216	1.669252846	2.410683804	0	0.661656721	0.355887866	6.380454527	0.308226967
0.240320186	3.436960413	0.67830577	0.068282362	0.113792981	2.122331992	3.163630269	1.699086639	0.843306629	2.11597463	0	3.157785326	0	4.367865344	1.2971248
0.698506651	4.389192266	1.022403097	0	0.045507324	2.224600272	3.290653767	2.101599056	2.095803694	1.838275647	0	3.100405301	0.506052592	2.689795371	0.921632539
0	2.647708876	2.794637385	0.398701847	1.343746964	2.261211209	1.894720075	2.857622817	1.719558083	2.28766098	0	3.003936695	0.667670967	2.714681272	0
1.539212323	4.484946446	2.331554313	0.03406787	0.60394158	2.780725777	4.535214336	2.181847713	3.743050431	2.564271908	0.658751444	2.807459225	0.716347856	5.132469769	0.629877717
0.623218613	4.42045987	0.674861589	0.003253183	0.058940555	2.025907968	3.936373032	2.19817382	2.953616131	2.01818519	0	3.078679342	0.241531062	3.98052451	0
1.594363561	4.176058679	2.152346451	0.026319418	0.554403031	2.775588089	4.122084355	1.36341355	2.182542163	2.450644281	0.531956341	2.617479839	0.580562029	5.249579546	0
0.650153176	5.91022807	1.747097022	0.086576878	0.519591365	1.752497133	5.512131992	1.742463331	4.084476971	2.913715677	0.898532584	1.906118781	1.448022285	4.320720114	2.392530866
1.164885785	5.706659449	0.476925161	1.88934686	0.763460246	3.025182867	4.698916017	2.941096943	4.865183363	3.440254341	1.752848703	3.841014606	0.969137776	5.881084778	3.094515323
0.553124475	5.106808377	1.488166617	1.70519725	0.742584976	2.606249849	4.604515626	3.308222074	3.262815911	3.2854375	1.094083429	2.926385228	1.176871746	5.677492332	1.653917564
0.299727464	3.83555135	3.451699802	0.221496472	0.946148837	1.580537625	3.256723946	2.05142147	4.749545918	4.090641146	0	0.906722582	0	3.580001841	1.217964596
0	1.931148262	0.213670689	0.058088901	0.244311268	2.544614564	2.608655427	1.574457521	2.144520785	2.162049588	0	2.718053397	0.257142631	4.35462789	0.735025117
0	2.821141389	0	0.378134888	0.217609417	2.224037414	2.957248839	1.52122156	1.254812747	6.114798179	0	0.920570407	0	6.516485319	0.48937348
1.204959686	5.229421886	2.818008706	0.19955256	1.612510975	2.842295942	5.43338755	2.402605099	2.975072704	3.063727132	1.537057774	3.34342005	0	3.490232909	2.458912616
1.698392254	5.665309624	2.147963105	0.100527967	1.324055013	2.672275002	4.401821948	1.686880949	3.899623133	2.888792146	0.274690233	2.823356302	0.950208972	4.936167985	2.622539277
0.2044027	4.800646195	0	0.071592535	0.133710405	1.660093006	3.648316405	1.181381716	2.569255495	1.774641026	0	3.016205059	0.49643502	3.85779004	1.547540638
0.583099357	3.148327243	1.677731065	0.047253134	0.693859821	3.110119663	2.811122008	2.292276044	2.019750109	1.872975548	0.682386429	3.063284928	0.224037572	5.112233034	0.362947102
0	3.611765336	0.219885748	0.137115467	0.098420156	1.968537675	3.229721994	1.49240616	1.360834048	1.58893168	0	3.331338146	0.264510391	4.369745171	0.425063933
1.945881263	2.453540406	0.336199401	0.11031064	0.55552068	1.542654011	2.989738531	1.129123066	1.02969298	2.53977804	0	1.549893595	0	5.893976611	0
0.242383584	3.383851605	0	0.01753705	0.094606313	2.236479092	3.575662138	1.976732892	1.897365068	1.909709071	0	2.877851329	0	4.482186826	0
0.819479127	4.059853525	0.508035706	0.115137148	1.368404687	1.539542033	4.16190296	2.154879471	1.668125487	2.679977315	0.550234672	1.882834395	0.826902815	5.270234354	2.648627273
0.202496132	3.490685724	2.056765355	0.01085876	0.074125045	2.617742353	3.288302676	1.931278569	1.214409463	2.285078246	0.792364708	3.45005802	0.492229728	3.746674181	0
0	4.844314156	1.144171095	0.035894808	0.567819605	2.648338525	3.181885525	1.336664454	2.412240141	4.43862366	0.736046382	2.734821082	0.2447535	6.79866617	0.837566311
0.818928938	3.873546062	1.17965348	0.036383723	1.330519703	0.509351092	3.484868141	1.255592567	2.590186135	2.472652966	0	0.455926294	0.599757178	4.739914645	0
0.384580161	2.91648071	0	0.011006096	0.026957731	2.692322694	2.930769501	1.757836348	1.225547972	1.808409636	0.245459024	3.237403207	0	4.089314862	0
0.833963593	3.872903548	0	0	0.045740107	2.378214975	4.278785911	2.267730149	1.612252198	1.692189138	0	2.915384871	0	4.321828191	1.32087442
0	2.864532702	0.500043709	0.840145885	0.865954212	2.668220355	2.399255653	2.31984153	2.486404959	2.066580089	0.29612378	2.816532082	0.32556682	5.041917973	0
0.686068257	3.43314662	2.634406712	1.434006795	0.518377433	3.246209299	3.136419674	1.485384346	1.976221557	3.353111773	0.63647607	3.409853517	0	5.087479904	0.765028832
0.21480051	5.047098838	1.163861197	0	0.078843112	2.623172956	3.076348348	1.74206747	1.426634372	1.610105563	0	3.111162539	0.282862276	3.002446144	1.074812267
1.704709661	4.619918499	1.447257859	0.328416835	1.200745502	2.483204912	4.764941344	1.708403183	4.222534448	3.17636057	0.707696119	2.605833618	0	3.897947255	1.763780787
0	2.955389827	0.371395274	0.0991209	0.117490764	2.458029472	3.13391008	1.884935355	1.518299631	1.464963345	0	2.921339739	0	4.401874036	0.205083512
0.162788759	1.868777984	0.611112868	0.008614369	0.048818586	2.554655325	1.554684342	1.582455891	0.504556425	1.438886828	0	3.054966341	0	3.233466039	0.631169089
0.215524945	3.570645378	3.566799379	0.322428076	0.699052249	2.932011496	3.460739178	1.729221839	1.880752261	2.172423784	0.257799238	2.904658807	0	3.836821484	0.799344453
0	3.925193826	0.735215172	0.014444523	0.164553414	2.466827766	3.660645748	1.890196434	1.440802051	2.056977851	0.242178796	3.056610296	1.011088371	3.859766051	0
0.708380124	4.330381561	0.765394966	0.067196089	0.989216877	0.806230723	3.005028325	1.642102221	2.96446465	2.855462863	0.469791614	0.682461656	0	5.044043404	1.586197519
1.165618209	4.434976296	0	0.092872465	0.386325713	2.040224822	3.919040888	1.747830964	3.490717379	3.647019729	1.238017551	2.378700557	0.588073186	5.646497231	1.192381275
0	4.850272649	1.262950844	0.15906509	0.30743067	2.3438705	1.925263401	1.250526549	0.530562158	1.152203127	0	3.209818282	0	5.082065348	1.603718656
0.230562115	4.33292078	0.654333905	0.041189008	0.309127689	2.794193679	3.757753986	1.582222092	3.271376046	2.953318382	0.506756307	2.278413642	0.30311636	5.888480291	0.844953238
0.632881274	3.419270588	0.266184896	0.008824286	0.058738971	2.222142879	3.464692436	1.631290592	1.193306742	1.853115341	0.290289569	2.907958689	0	3.288747536	0.507592362
0	2.922117426	0	0.010717179	0.056291485	2.546506685	4.134187854	1.526939122	1.797443114	1.984871535	0	3.061623766	0	4.273620279	0
0.466854048	4.388268181	0	1.360475603	0.323824713	2.455687814	3.963723239	3.29474862	3.093141368	3.230743725	0	2.64723395	0.330857499	6.392454753	2.17348539
0.192827421	2.541463235	0	0.006879425	0.125964595	2.998924718	2.541015086	1.882580749	0.17032336	1.980001312	0	3.405297064	0	4.397021891	0.21941153
0.960393306	4.278402891	0.231454818	1.169145367	0.927475806	2.173062839	3.71749893	2.303466625	1.911792711	3.225188397	0.820224098	2.192172193	0	5.395577768	2.558015765
0	3.537265171	1.021108815	0.099894484	0.038255131	2.005442579	2.524747428	1.189841723	1.566238007	1.380712072	0	3.304067577	0	3.147126004	0
0.584740405	3.285641918	0.633843301	0.312920681	0.693451972	2.062847664	3.002266816	2.068016621	3.15103983	2.718826672	0	1.821207894	0	4.760244595	1.226182491
0	3.847073609	0.710043071	0.094081874	0.017002402	1.800344592	3.644708032	1.099442676	1.594069327	1.419560006	0	2.784874251	0	3.161389543	0
0.377608439	4.33712831	1.406729048	0.130935025	0.618906069	2.515973021	4.562374485	1.969940294	2.223555733	2.660333134	0.787930176	2.561482719	0.853858666	5.870515418	0.426168187
0.789692626	4.13304049	0.851531855	0.074195277	0.220297036	2.142149465	3.394358205	1.81446931	2.799059205	2.000488073	0	3.317636115	1.399786253	4.335634228	1.081364438
0.748723635	4.542553772	1.524799247	0.220156253	0.43299806	0.218831828	3.749871593	1.961247176	2.194359564	2.14584927	1.162937028	0.252061735	0.544916905	4.184949083	1.90111301
0.379452185	2.739445749	0.734837013	0.028727174	0.144386193	2.173525225	2.317634016	1.121588112	0.610944785	1.761166791	0.449234049	2.336956698	0	4.190741316	0
0.591578815	4.09945482	0.355895446	0.083111943	0.080491953	2.079071903	3.77714494	1.392857993	2.114832416	1.800549443	0.387095186	3.215023461	0	3.948038892	1.603052806
0	3.766931897	1.852904553	0.028249939	0.512129508	2.143532722	3.608341945	1.263862964	2.039307903	2.698033676	0.442743661	2.31259956	0	5.002899625	0.748068253

0	5.522699353	2.689111489	0.360825151	1.262882206	4.320321537	4.053075182	2.714800027	3.078897298	2.421349184	0.414681417	4.866230064	0.454180906	3.826829034	0.959779173
0.25607269	4.202591533	1.441068695	0.004675355	0.147864117	3.341139152	3.858889249	2.162658606	1.961003635	1.988001245	0.305495063	3.339681409	0.607952613	4.226529677	0
1.315884081	4.036495012	0.769520304	0.045452417	0.404994317	2.873495332	3.34043279	1.596765027	1.994030903	2.801435467	0	2.297848506	0.36412546	4.590064841	2.06264384
0.34788177	3.666668255	2.612912991	0.089230956	0.337741934	2.668698075	3.217771682	2.463685412	0.77977839	2.179471925	0	2.847662805	0	4.864089623	2.510553327
0.855254578	4.182654346	0.920779583	0.212731903	0.7292205	2.696453525	4.023293684	1.718900865	2.441260761	2.330029705	0.576498824	2.088279087	0.348122801	4.141710521	3.488361955
1.546696418	4.334803845	0.277521534	0.0590043	0.311771692	2.556231852	3.928154436	1.287666474	2.011058497	2.831507522	1.736915828	2.197146766	0	5.827271535	0.28772032
0.625234595	3.124180219	1.26138015	0.176252251	0.934904886	1.367192034	2.940472723	1.553165551	2.113524078	2.283029456	0	1.457081596	0	3.984147149	0.501208336
0.726220502	3.733628288	2.859919678	0.020882647	0.765741049	2.606669094	3.774749384	1.944891407	2.431816515	2.92018202	1.640937515	2.490788608	0	5.17577045	1.323727809
0.894199717	4.233051054	2.671466024	0.358618672	0.613640208	2.35460825	4.938803268	2.385642172	3.003971308	3.470430685	1.625326375	0.224848393	0	5.020851138	2.982480035
4.021373792	6.110725635	1.633550963	0.089050373	0.23440051	2.706021727	6.933021827	1.519027316	3.124335858	4.366305237	1.108702852	3.778425691	2.074692521	5.753212334	5.079807611
4.189671775	5.164486227	2.511978631	0.099250697	0.317768253	1.676330218	5.508614937	1.553212843	3.082061368	4.525635085	0.798426241	2.334270972	2.004166009	4.745051299	0.552046472
3.357590338	5.519639638	1.551823626	0.45173789	0.393823884	0.395382222	5.565281639	2.264529625	3.350681368	3.942116961	0.382479529	0.583149674	1.008583378	5.14881718	1.284427122
0.695077356	2.445455997	0.423997575	0.220965527	0.446854201	2.013373032	3.011371377	0.682404511	2.908576551	3.659960826	0	1.775286961	0	5.45849074	1.942579028
0.966784178	3.387732749	1.409107991	0.032507492	0.415706906	2.17747667	3.144753191	1.386794575	2.920618829	2.177578079	0.581803129	2.337985717	0	5.141490625	0.955861904
0.366806926	3.39346931	0	0.145926002	0.215206258	0.293597204	3.591261901	1.728756011	3.747431096	2.078370189	0	0.155404517	0	4.813831051	0
2.484236953	3.942468994	1.181551939	0.006908418	0.675386258	0.203476521	4.000322056	2.203457323	2.007253523	3.057483548	0	0.143754813	0	5.685621307	5.444857568
2.142950786	4.242449026	0.323590721	0.053899864	0.608850592	0.855620454	5.10772876	2.193627794	2.376166403	3.836289381	0.352290195	0.831722139	0.691207623	4.922463767	1.033186741
1.241189762	5.892934771	1.325179977	0.292898013	0.31686831	3.132856658	7.536871571	3.224932428	2.639000476	2.974541937	1.164885492	3.693959674	0.941192128	3.542703634	0.4768174

The test set

id	age	grade	stage	M	T	MIR324	XIST	MIR210	MIR25	GABPB1.AS1	LINC00894	SNHG7	FGD5.AS1	MIR22HG	MIR126
TCGA-CJ-6033-11A-01R-1858-07	0	4	1	2	2	0.423758576	1.774511129	0.605435533	0.541240885	0.578250392	0.210684135	2.766387983	5.10113994	2.869272594	0.925799049
TCGA-CZ-5984-11A-01R-1672-07	0	3	1	1	0	0.184250443	0.001103974	0.187391932	0.932194695	0.548654848	0.213201265	2.348907139	5.203332882	3.741524511	0.621520036
TCGA-CZ-4865-11A-01R-1503-07	1	2	1	1	0	0	2.328843557	0.547993771	1.493237877	0.591588621	0.260960168	2.585594648	6.118883206	3.535878892	0.676220939
TCGA-CJ-5678-11A-01R-1541-07	0	3	1	2	1	0	0.003427006	0	0.645512292	0.294260866	0.035607773	2.332400781	5.261142852	3.362677958	0
TCGA-CZ-5458-11A-01R-1503-07	0	3	1	1	2	0.123968392	0	0.630552675	0.163031905	0.505320481	0.084603019	2.417820495	5.861753631	4.651786329	0.161217145
TCGA-CZ-5454-11A-01R-1503-07	0	2	1	2	1	0.24105013	0	0.45451078	0.97810819	0.750956274	0.247111086	2.083761756	5.751243723	3.57660018	0
TCGA-CW-6090-11A-01R-1672-07	1	3	1	1	0	0	0.002200799	0	1.061220927	0.703789363	0.265571536	2.11665476	5.436677986	3.273014934	0.237545779
TCGA-A3-3387-11A-01R-1541-07	0	2	1	1	0	0.40325	0	0.219306143	1.053073685	0.498897888	0.129943891	2.192274703	4.988400416	3.94430098	0.510880692
TCGA-CJ-5679-11A-01R-1541-07	1	4	1	1	2	0	0.002166821	0.347484005	0.919598345	0.566013457	0.191733525	2.25232309	5.446084945	4.155398913	0.234157759
TCGA-CW-5580-11A-02R-1672-07	1	3	1	2	2	0	2.062755009	0.159639041	1.250366488	0.709588861	0.274604674	2.45621765	5.798934457	3.72323509	0.540290102
TCGA-B0-5701-11A-01R-1541-07	0	4	1	1	2	0	0.001137543	0.514771648	0.458719273	0.591676841	0.133523426	2.420296189	5.390304842	3.077164498	0.244836818
TCGA-A3-3358-11A-01R-1541-07	0	2	1	1	0	0.617148764	2.619590617	0.446112932	1.6486326	0.535032156	0.280167165	2.605699634	5.867546222	3.768518254	0.768428925
TCGA-CZ-5465-11A-01R-1503-07	1	2	1	1	2	0	1.71248891	0	0.667027567	0.546544127	0.08742266	2.343893448	6.062220341	3.566486167	0.52171633
TCGA-CW-5581-11A-01R-1541-07	0	3	1	1	0	0	0	0	1.13344063	0.280737195	0.126953999	2.468011887	5.162362558	3.041573084	0.557686815
TCGA-CW-5591-11A-01R-1541-07	0	2	1	2	2	0.356504127	0	0.651529711	0.247173854	0.358800679	0.10696826	1.754311687	3.946357119	1.768833503	0.453504367
TCGA-CZ-5467-11A-01R-1503-07	1	4	1	1	2	0	0.91865786	0.466111847	0.414624977	0.24065099	0.059383701	1.540283685	5.309006702	4.653360075	0
TCGA-CZ-4863-11A-01R-1503-07	0	3	1	1	2	0	1.469485604	0.61773031	0.765438914	0.450146277	0.138050512	2.448819075	5.899575892	2.984530673	0
TCGA-CW-5585-11A-01R-1541-07	0	2	1	2	2	0.166912829	0	0	0.575907052	0.252314587	0.055343358	2.445829264	5.235789152	3.824903846	0.40406197
TCGA-B0-5697-11A-01R-1541-07	0	2	1	1	0	0	0.001510918	0.464372586	0.320339512	0.341932448	0.144951991	1.648054333	5.379947805	3.887466888	0.31695593
TCGA-CJ-5676-11A-01R-1541-07	0	3	1	1	2	0	0	0	0.718323148	0.400810218	0.066464774	2.231318537	5.252813564	4.17967765	0.213402414
TCGA-B0-5711-11A-01R-1672-07	0	3	1	1	2	0	0.000983712	0	0.720162788	0.804260014	0.12756973	2.450175869	5.73739922	3.388798765	0.214047635
TCGA-BP-4163-01A-02R-1325-07	0	3	1	1	2	0.272726949	2.040929114	1.640956868	2.01862511	1.411722627	1.022938632	1.338396533	4.299806039	2.354249151	0.630756013
TCGA-A3-3320-01A-02R-1325-07	0	0	1	1	0	0.933075831	5.156265877	2.369170925	1.688331196	1.506302809	1.258989153	2.571875303	4.427215191	3.773057894	3.167991903
TCGA-B0-4703-01A-01R-1277-07	0	4	1	2	2	0.672809855	0.006008735	2.008949369	1.755613718	0.75414063	1.196547269	2.535482013	4.617255319	2.648641346	1.74361757
TCGA-CW-6097-01A-11R-1672-07	0	4	1	1	2	0	0.004021632	1.124444102	1.217036168	0.854860515	0.12942451	1.873182166	4.499160698	2.654861391	0.726439739
TCGA-MM-A564-01A-11R-A266-07	1	2	1	3	1	1.058869154	0.012243211	3.29630378	3.229203939	1.149919031	0.602258795	5.481691476	4.067585269	1.945764409	4.011717959
TCGA-BP-5192-01A-01R-1426-07	0	2	1	1	0	0	0.001044814	2.341785786	0.894907459	0.542413651	0.10175281	3.932624126	4.095907131	2.27631581	0.887040873
TCGA-CJ-4920-01A-01R-1426-07	0	2	1	1	0	1.193708723	3.881392462	1.39972301	2.07673017	1.504410628	0.490332421	2.950143258	4.267720346	3.694358016	2.268063817
TCGA-B2-5641-01A-01R-1541-07	1	3	1	1	0	0.190502781	0.002286993	0.193743883	1.527146045	0.734261965	0.084077544	1.502786509	3.979546049	1.975733501	0.802402783
TCGA-CZ-5459-01A-01R-1503-07	0	3	1	1	2	0.23768756	0	1.186819468	0.967320952	0.638747371	0.196521715	4.331374831	3.710171403	1.921392484	1.846358521
TCGA-A3-3323-01A-02R-1325-07	0	0	1	1	0	0.662914344	0.015676745	1.755114512	3.294837527	2.408991321	0.723248165	2.624576036	4.32224568	2.603038567	3.733014442
TCGA-BP-4161-01A-02R-1325-07	1	3	1	1	0	0.916059426	0.003590823	2.423548561	2.305100494	1.962033232	0.827869509	2.284346644	4.481253905	2.488821332	1.651960099
TCGA-B8-4143-01A-01R-1188-07	1	3	1	2	2	1.21249948	4.165786343	4.837604519	4.544291224	3.604429472	1.849408169	4.649642183	4.757716997	3.650259039	1.798999734
TCGA-CZ-4866-01A-01R-1503-07	1	3	1	1	0	1.11195523	1.544592065	2.770096937	1.706291533	1.430546515	1.143383027	2.0403374	4.391367971	3.194201099	0.436316162
TCGA-B8-A54J-01A-11R-A33J-07	0	2	1	1	1	1.436364766	0	2.480377157	2.360836301	1.268324615	0.558379256	3.469957864	4.257956734	3.382110947	4.449486696
TCGA-B2-3923-01B-10R-A277-07	0	2	1	1	1	0	0.41290208	0	0	3.444476789	3.01015859	2.544793738	6.184994672	3.185586281	0
TCGA-CW-5580-01A-01R-1672-07	1	3	1	2	2	0.822706371	3.988939231	0.740611277	1.608772472	0.413818238	0.261122709	2.610379222	4.687847665	2.851133733	0.194970048
TCGA-AK-3460-01A-02R-1277-07	0	2	1	1	0	0.552959005	0.137164071	3.257088509	1.520229434	0.910790853	0.486736863	4.019050271	4.476588074	3.180766873	0.943503489
TCGA-BP-5177-01A-01R-1426-07	0	3	1	1	0	0.560384087	3.85755208	1.384587678	1.179998043	0.497312606	0.314782773	3.593211966	4.269564724	2.504992065	0.502796775
TCGA-B8-5549-01A-01R-1541-07	0	3	1	1	0	0.204826956	0.088731557	1.864124271	0.85884822	0.210562193	0.06106227	2.420331412	4.339122449	2.621845476	0.487495714
TCGA-B2-3923-01A-02R-1325-07	0	2	1	1	1	0.456180805	0.010514297	0	1.31476661	1.267547891	1.03844722	3.1676088	5.533900654	4.05696761	1.153869862
TCGA-CZ-5982-01A-11R-1672-07	0	2	1	1	0	1.018843031	3.525357146	1.133716224	1.464333681	1.226578577	0.218607138	1.815877469	4.372073961	3.597941036	0.825979739
TCGA-BP-4982-01A-01R-1334-07	0	3	1	1	0	0.528269473	0.004474216	2.09228421	1.98111767	1.385315008	0.810564826	2.815815321	4.673657725	3.033148678	2.25921154
TCGA-A3-3358-01A-01R-1541-07	0	2	1	1	0	1.698426199	3.862673901	0.972802975	2.477605536	0.621599718	0.65213548	3.02244565	4.585153714	2.693791487	2.290739969
TCGA-CZ-5461-01A-01R-1503-07	0	4	1	2	0	0.411042563	0	0.834435981	1.384936326	0.273179689	0.105554461	2.775875414	4.701756389	2.178875006	0.785700531
TCGA-6D-AA2E-01A-11R-A37O-07	1	2	1	3	0	0.511810328	1.731167988	1.663528531	1.829861529	0.718514977	0.903969374	2.649458624	4.794021197	4.41771234	1.92809458
TCGA-AK-3461-01A-02R-1277-07	1	2	1	1	0	1.680807472	0.095918725	2.913483639	2.073420641	1.7762912	0.838857617	3.092023539	4.435968858	3.433181986	2.45742822
TCGA-B0-5095-01A-01R-1420-07	1	3	1	1	2	0.220065499	0.001335316	2.066216352	1.947216828	0.98769993	0.636531713	2.780366091	4.455359959	2.511883868	1.665076198
TCGA-B0-4821-01A-01R-1503-07	1	3	1	1	2	2.035045434	1.651470185	2.839789693	2.119109671	1.064718889	0.944967701	4.005091109	3.791807438	2.791062943	1.35346636
TCGA-B0-5092-01A-01R-1420-07	0	3	1	2	0	0.632245508	3.586473321	3.833167944	3.100547408	1.299675714	1.423352529	3.051631539	4.145583717	3.430017446	1.552113052
TCGA-CJ-4908-01A-01R-1426-07	0	2	1	1	0	0.452316615	0.00099476	2.832660928	1.398680042	1.027664994	0.76006718	2.701618271	4.598213085	3.140376942	1.706768167
TCGA-CJ-4907-01A-01R-1426-07	0	3	1	1	2	0.313447316	0	2.540566054	1.296334715	0.521932354	0.702729728	2.755268175	4.557640846	2.379969509	1.083487871
TCGA-B2-5635-01A-01R-A277-07	1	2	1	1	0	0	0.028037144	1.521759206	2.065521397	0.991748208	0.688908263	2.46337974	5.07251543	2.196885234	1.668864302
TCGA-BP-5182-01A-01R-1426-07	0	3	1	1	0	0	0.002915758	1.151785301	0.214135977	0.332246848	0.128993428	2.529344188	4.37165686	2.935149893	0.211792875
TCGA-DV-5576-01A-01R-1541-07	0	2	1	1	0	0.191779535	3.057082851	3.669626322	1.711383492	1.068636834	0.249594272	3.325178096	4.873254782	3.360847648	1.522719244
TCGA-BP-4776-01A-01R-1289-07	0	2	1	1	0	2.221334608	0.011830624	2.453075246	2.909281129	1.513441117	0.615489174	4.081222001	4.022962159	4.121500115	4.410940447

TCGA-B0-4817-01A-01R-1277-07	1	3	1	1	2	1.156776115	0.060197827	3.405750898	0.628990834	0.592354292	1.045149546	3.794793508	3.592975639	2.559830443	0
TCGA-B0-5691-01A-11R-1541-07	1	3	1	1	0	0	3.062984332	1.528468384	1.346312615	0.660658354	0.288451212	3.074822307	4.808230859	2.742764568	0.686869226
TCGA-BP-4760-01A-02R-1420-07	1	2	1	1	0	0	0.00588699	1.222535254	0.661851209	0.554537904	0.040338753	3.024115857	4.921743925	3.379207547	0.253086894
TCGA-B2-3924-01B-03R-A277-07	1	2	1	1	0	1.097213651	0.015313894	2.033396024	2.184227652	3.823327066	2.79081453	3.072296226	4.511198259	3.693542127	3.955402839
TCGA-CW-6090-01A-11R-1672-07	1	3	1	1	0	0.168283241	0	0.324165337	1.652825431	0.495445675	0.070944209	3.126049389	4.105988676	2.305816289	0.217875847
TCGA-B0-5106-01A-01R-1420-07	0	2	1	1	0	1.194474761	0.015585828	3.660425916	1.74951743	1.779557553	1.504591441	3.504751716	4.513801753	2.192403498	2.204178876
TCGA-CW-5591-01A-01R-1541-07	0	2	1	2	2	0	0	0	0.884364853	0.392099127	0.032067906	2.134041237	5.219441694	3.04480513	0.503857221
TCGA-BP-5190-01A-01R-1426-07	0	3	1	1	0	0	0.003541026	1.47564669	1.969182875	0.899056922	0.490969565	2.063121368	3.658786395	2.700555056	0.253478099
TCGA-CJ-5675-01A-11R-1541-07	1	3	1	1	1	0	0	0.893478033	0.642984286	0.363431422	0.063173038	2.337147437	4.191249777	1.801340786	0
TCGA-CJ-4640-01A-02R-1325-07	0	4	1	1	2	0	0.018330484	1.109978636	0.649725057	0.46094429	0.141803605	2.671416576	4.706583405	2.615016021	0.459205386
TCGA-B0-4690-01A-01R-1277-07	0	3	1	2	3	0.365625446	0.012804219	0.911540759	1.807278267	1.532902412	0.548318233	2.001355464	4.749818674	2.648192309	0.250999292
TCGA-BP-4765-01A-01R-1289-07	0	2	1	1	0	1.263292048	0.007206836	3.09057134	2.294025257	1.311744508	0.700136823	2.914011683	4.47524501	4.156459067	2.670853963
TCGA-CZ-4861-01A-01R-1305-07	0	2	1	1	1	0.18156754	0.02265413	2.270083419	1.383710193	0.974117005	0.388275838	3.46412585	4.262608165	1.984737502	0.436624234
TCGA-CZ-5468-01A-01R-1503-07	1	4	1	2	2	0.625284222	0.002197426	2.509751062	1.961034886	0.305787164	0.285247041	2.628696003	4.493134653	2.532831913	0.921096256
TCGA-B0-4848-01A-01R-1277-07	0	3	1	1	2	0	0.073960556	1.372123032	0	0.463505958	0.101034098	1.694651527	4.390187144	2.480025901	0.598298851
TCGA-CZ-5458-01A-01R-1503-07	0	3	1	1	2	0.327750176	0.001033501	1.846762064	0.594652956	0.474157905	0.180498532	2.718467794	4.457792939	1.959450688	0.588908157
TCGA-B0-5080-01A-01R-1503-07	0	3	1	2	2	0	0.003680425	0.69340529	0.854475947	0.35006731	0.324562023	2.519459576	4.8743529	3.070315748	1.136120598
TCGA-CZ-5985-01A-11R-1672-07	0	2	1	1	1	0.33669118	0	0.739062021	1.036628265	0.398235237	0.263285153	2.036757893	3.875924427	1.782486306	0
TCGA-BP-4758-01A-01R-1289-07	0	2	1	1	0	0.462534235	0.044255057	2.865046386	2.511848753	0.764682245	0.372476825	3.418712799	3.890982574	3.003994059	1.659047133
TCGA-B2-4099-01A-02R-1188-07	1	3	1	1	0	0.868000586	0	2.067814097	3.121219248	0.784928829	0.503865243	2.615918938	4.745853828	3.691722725	2.686200618
TCGA-BP-4981-01A-01R-1334-07	1	3	1	1	2	1.416512174	0.84452771	2.608629334	2.499289149	1.212833494	1.268203691	3.399047115	3.554553492	3.055666983	2.137337245
TCGA-B0-5705-01A-11R-1541-07	0	2	1	1	0	0.445895804	2.393621444	1.156492346	0.975086512	0.35013321	0.195859165	2.442085657	4.386715511	2.35500436	0.966723703
TCGA-A3-3317-01A-02R-1325-07	1	2	1	1	1	1.640225841	0.003673285	1.585763232	2.495070482	2.056542943	0.983831665	2.207087238	4.538444684	3.696920623	2.80277735
TCGA-BP-4971-01A-01R-1334-07	0	3	1	1	2	2.097661534	0.0068779	2.964079774	2.510328897	2.734654826	2.166153715	2.773404452	4.516199142	3.139941046	2.227054814
TCGA-CJ-4895-01A-01R-1305-07	0	4	1	2	2	0.991849889	0.009321497	1.421075484	2.027175101	1.311233693	0.289053284	1.353135052	4.393023033	2.7820093	1.45263174
TCGA-BP-4330-01A-01R-1289-07	0	2	1	1	2	0.948895612	4.316219755	3.820385136	2.393205136	1.709439634	1.015619649	3.500282175	4.29042405	2.621728591	1.36524321
TCGA-DV-5573-01A-01R-1541-07	0	2	1	1	0	0.223219143	0.076612038	1.830120624	2.507259657	1.022933168	0.622418353	2.555427057	4.557507492	3.010965717	1.4667117
TCGA-CJ-4874-01A-01R-1305-07	1	3	1	1	0	0	1.24902199	0.921540306	0.475393816	0.28059879	0.06222236	2.826386899	4.596019199	1.923834264	0.47062114
TCGA-CJ-4881-01A-01R-1305-07	0	3	1	1	2	1.093173017	0.101368341	3.985281373	3.022261414	1.223660105	0.611385633	2.56172394	4.320197108	2.880647518	2.141961325
TCGA-A3-3319-01A-02R-1325-07	1	2	1	1	0	0.974497516	0.000977539	0.576522796	1.550858016	1.001341697	0.348774216	1.306700897	4.482213286	2.40299052	0.843718763
TCGA-B0-4694-01A-01R-1277-07	1	4	1	1	2	0.188850764	0.023615792	1.648913011	1.083276594	0.338477806	0.165283477	1.717730574	4.336641985	2.310496842	0.796697417
TCGA-BP-4985-01A-01R-1334-07	1	4	1	1	2	0.160443754	0.005710527	0.969496276	0.210190763	0.199138483	0.13204505	0.75297463	4.255263309	2.649042763	0.948582679
TCGA-B0-4696-01A-01R-1277-07	0	3	1	1	2	1.14954938	0.364651538	0.696260721	1.23500939	1.754400894	0.431801109	2.697297349	4.676164408	3.499960743	0.34207712
TCGA-AK-3447-01A-01R-1766-07	1	2	1	1	1	0	0.00313833	0	0.600944524	0.074584236	0.029407916	4.388786231	4.314522524	3.847459769	0
TCGA-A3-3376-01A-02R-1420-07	0	2	1	1	0	0.923606159	0.015268437	1.498921875	2.111588714	0.817804429	0.480663541	3.123281006	5.218212342	2.652799216	2.674336161
TCGA-B8-5550-01A-01R-1541-07	1	3	1	1	2	0	0.004787694	1.002756061	1.036767425	0.325673768	0.116130523	2.641596271	4.306497426	2.898520747	0
TCGA-MM-A84U-01A-11R-A37O-07	0	2	1	3	0	0.487097311	0.59997913	2.875450832	2.326600583	0.998277942	0.623550466	3.163672833	3.898042345	2.870245227	2.234002208
TCGA-EU-5904-01A-11R-1672-07	0	0	1	1	0	0.193897005	2.84783604	1.803186019	0.468424999	0.394158282	0.24920671	3.256707417	4.330726326	2.735508225	0.961781181
TCGA-CJ-4899-01A-01R-1334-07	0	2	1	1	0	0.153845163	0.004554271	2.111941066	1.632534992	0.813823821	0.674449284	3.255242971	4.665149795	3.399413068	2.140890703
TCGA-B0-5102-01A-01R-1420-07	1	3	1	1	0	0.736980449	3.327357583	2.773516618	2.130227377	1.366616069	0.876629198	2.600774505	4.037361957	2.447872557	1.930189577
TCGA-BP-4158-01A-02R-1289-07	1	2	1	1	0	1.23017343	0.02969941	2.770681265	3.500670079	1.070507555	0.636267901	4.159825055	4.324928568	2.375793075	2.608521259
TCGA-CJ-4893-01A-01R-1305-07	1	3	1	1	0	0.454979252	2.463142712	1.591371095	1.10667339	0.397761235	0.230130058	2.090501398	4.29321846	2.171364883	0.573893044
TCGA-CJ-4904-01A-02R-1426-07	0	3	1	2	2	0	2.163520944	0.811388504	0.770713695	0.370484621	0.042669443	2.81876429	4.580475687	3.303226661	0
TCGA-BP-4759-01A-01R-1289-07	0	2	1	1	0	0	0.032024534	1.758772724	1.071690962	0.321513333	0.095334387	3.79022332	3.851620168	1.523743956	1.062765564
TCGA-BP-5178-01A-01R-1426-07	1	4	1	2	2	0.45285216	0.000996131	0.701102424	0.987914644	0.167882245	0.085372381	1.897151379	3.996905937	2.66209925	0.216557859
TCGA-B0-4839-01A-01R-1305-07	1	2	1	1	0	0.204097449	0.290284781	1.731585962	0.490817783	0.494856541	0.185588538	3.311393329	4.070625323	1.703628226	0
TCGA-B0-5707-01A-11R-1541-07	0	3	1	1	0	0	2.386758209	0.463068468	2.077757577	0.539211128	0.144499162	1.375915388	5.293813185	2.780250469	0.316021424
TCGA-CJ-6028-01A-11R-1672-07	0	4	1	2	2	0.24444596	0.010440219	0.248528516	0.577602676	0.645761027	0.139319881	2.406662697	4.370439501	2.535889893	0
TCGA-AK-3429-01A-02R-1325-07	0	2	1	1	1	0	1.37052591	1.411818109	0.346922263	0.411046965	0.081043062	2.987673808	4.004610054	2.204623129	0
TCGA-B8-5545-01A-01R-1672-07	0	2	1	1	0	0	0	0.808684665	0.669529033	0.347551452	0.048231668	2.622143457	5.109047476	2.393123889	1.115840229
TCGA-CZ-4864-01A-01R-1503-07	1	3	1	1	1	0.661971017	0.003771289	3.390291389	2.786609019	1.247408608	0.877699121	2.754500707	4.699120213	2.72807753	2.273800125
TCGA-BP-4999-01A-01R-1334-07	0	2	1	1	0	0.494765274	0.002485255	2.368850759	1.844350576	1.427398503	0.520826485	3.169249137	4.772724534	4.173119978	2.445479084
TCGA-B0-5119-01A-02R-1420-07	0	2	1	1	0	0.375571978	3.12401375	2.509134294	0.842330016	0.453446911	0.181815044	2.690085549	4.56451069	2.425479954	0.258101203
TCGA-CJ-4637-01A-02R-1325-07	0	4	1	2	1	0.604940773	4.186130684	1.936867221	2.084491928	1.633828691	0.860014016	2.712490675	4.554273714	3.487840314	1.100286792
TCGA-CZ-5463-01A-01R-1503-07	1	2	1	1	1	0.444213183	0.001460849	1.006909287	1.42366895	1.884033433	0.398072068	3.563560548	4.306346515	2.275289576	0.672545826
TCGA-B0-5120-01A-01R-1420-07	1	2	1	1	0	0.378126887	3.655756605	2.018283809	2.534672683	0.621263102	0.258685768	3.051640465	4.325549648	4.168553756	1.127311112
TCGA-B4-5836-01A-11R-1672-07	0	2	1	1	0	0	2.797372416	0.360282701	0.245789118	0.281250048	0.106339945	2.110346031	4.361009913	2.559725013	0.451131384
TCGA-B0-5098-01A-01R-1420-07	0	3	1	1	0	2.011575631	0.240060145	0.47084332	2.334272143	1.669927758	0.709793207	3.206791113	4.434309525	2.089372448	0

TCGA-A3-3313-01A-02R-1325-07	0	3	1	1	0	0.987779147	0.007948359	0	1.99863069	1.318135834	0.874520062	3.010695444	4.889012013	3.390607882	1.198756996
TCGA-B0-5081-01A-01R-1334-07	1	2	1	1	2	1.04987197	4.005470142	1.612835707	1.687050435	1.217283824	0.893265366	2.550633332	4.254249835	2.590441138	1.602720761
TCGA-B0-4713-01A-01R-1277-07	1	2	1	1	2	0.502127999	2.675524335	2.389209401	1.255603638	1.047291173	1.190977496	3.568643516	3.461854276	2.626742446	1.245705431
TCGA-B0-4836-01A-01R-1305-07	0	3	1	2	2	0	0.008864138	1.922855771	0.98177187	1.06708596	0.240396651	2.128832306	4.165551808	2.241774467	0
TCGA-BP-4994-01A-01R-1334-07	0	3	1	1	0	0.13477323	0.006336825	0.997838605	1.031735634	0.880811313	0.890143229	2.413119725	5.78789405	4.237612391	0.82712455
TCGA-BP-5168-01A-01R-1420-07	1	2	1	1	0	0	0.011355485	1.654287811	0.768170568	0.376244431	0.048583798	3.602430183	4.094591834	2.222116015	0.300548177
TCGA-B8-5553-01A-01R-1541-07	1	2	1	1	0	0	2.116423967	1.723562085	1.141482266	0.452683535	0.132425601	2.730904226	4.501866417	3.021358902	0.482637163
TCGA-B4-5844-01A-11R-1672-07	0	0	1	1	1	0	2.898008565	0.847098802	0.702827096	0.571394559	0.144861451	2.590683851	4.51153514	2.872134848	0.499310449
TCGA-B0-4688-01A-01R-1277-07	0	4	1	2	3	0.925948109	0.037210968	0.818506227	1.847918673	2.008471766	0.802313012	1.873351619	5.40474421	3.126893745	0.840385786
TCGA-BP-4803-01A-01R-1305-07	1	3	1	1	2	0	0.004551218	0.363170445	1.209126784	1.105664292	0.107269123	2.728390016	4.411341133	2.018913316	0.945683443
TCGA-BP-5007-01A-01R-1334-07	0	2	1	1	1	0.32075337	0.011059347	3.126318945	2.410259218	0.875913141	0.94737362	3.200965534	4.483824715	2.977851994	2.717475752
TCGA-G6-A8L6-01A-11R-A37O-07	0	3	1	2	1	1.481941	0.016519834	2.960088764	3.253876081	0.776662809	0.507927776	5.200227897	3.914595981	2.918922822	4.498461631
TCGA-CJ-4902-01A-01R-1426-07	0	3	1	1	2	1.12369286	0.003471657	1.398905554	2.652559225	1.039275831	0.90415806	2.465414823	4.394683715	3.104079456	2.134083945
TCGA-CJ-4889-01A-01R-1305-07	0	4	1	1	0	0.367327	2.323154565	1.616653295	1.812700232	1.252112635	0.241634837	2.616138408	4.93331862	3.555363974	1.338146501
TCGA-B0-5693-01A-11R-1541-07	0	2	1	1	0	0.875919544	4.526547416	1.757456372	0.531496703	0.812102015	0.3030369	2.516841961	4.258457687	2.851020849	2.029033495
TCGA-DV-5567-01A-01R-1541-07	0	2	1	1	0	0	2.357826876	2.401260811	1.264270276	0.51101124	0.084791955	3.638307082	5.10685079	2.602452718	0.899930638
TCGA-B0-5812-01A-11R-1672-07	0	3	1	1	0	0.342964449	0.002173973	0.184749638	0.921842888	0.717080322	0.137271825	3.083684383	4.523128077	2.754362589	0.913806534
TCGA-CJ-4876-01A-01R-1305-07	0	3	1	1	1	1.400301001	0.022421378	2.4169423	1.941825686	1.292468898	0.663336091	2.563384187	4.180752145	3.591092378	1.056711821
TCGA-BP-5010-01A-02R-1420-07	0	4	1	1	2	1.772986453	0.004515929	3.034803637	2.797227249	0.522538536	0.428550653	2.325479763	4.047822572	3.744408925	1.304659999
TCGA-B0-5121-01A-02R-1420-07	0	2	1	1	0	0.171959627	0.004099188	1.194649992	1.332881137	0.92316851	0.393873789	2.847944458	3.93096702	2.104312684	1.223041463
TCGA-B8-4621-01A-01R-1503-07	0	3	1	1	0	0.768161899	0	1.351106988	1.988644053	0.860713713	0.356387693	1.576044831	4.834563061	2.976350289	0.693876131
TCGA-B8-A8YJ-01A-13R-A39I-07	0	2	1	1	0	1.104362402	3.225563671	1.900031074	2.418079109	1.872517748	0.43263663	2.383874548	5.042666321	4.843367544	3.370809504
TCGA-CW-5589-01A-01R-1541-07	0	2	1	1	0	0.2040582	0.077920395	1.662548108	0.856240963	0.405883121	0.057094467	3.755836224	4.291943127	2.341685254	1.000705075
TCGA-B2-5633-01B-04R-A277-07	0	2	1	1	0	1.54508067	0.178243446	2.507767423	2.935417009	4.126210098	2.822761049	2.758152896	4.488937427	3.457657352	3.65839112
TCGA-B0-4822-01A-01R-1277-07	1	4	1	1	1	3.076824132	0.014592708	2.612763415	3.133000326	1.413089117	0.757668574	3.055940642	3.85021919	3.28370832	4.766690434
TCGA-CJ-5683-01A-11R-1541-07	1	3	1	1	0	0.586661473	0.282346453	0.226874517	1.477191449	0.97215391	0.206713603	3.510820929	4.241447935	2.521744646	1.346355065
TCGA-CZ-5453-01A-01R-1503-07	1	2	1	1	1	0	0.001003817	1.3296304	0.580582273	0.949415387	0.206118243	3.244894969	4.131787891	2.822158906	0.574948064
TCGA-B0-5088-01A-01R-1334-07	0	3	1	1	0	1.195602736	0.010423646	2.761501125	1.532363856	0.960223261	0.994215896	2.846687649	3.824289249	3.384468947	2.245086839
TCGA-A3-3357-01A-02R-1420-07	0	3	1	1	1	1.413480914	0.004809281	1.062831042	1.45576471	1.535189799	0.60002327	2.410418088	3.74022403	2.592781291	1.171678753
TCGA-CZ-4856-01A-02R-1426-07	0	2	1	1	0	0.152129637	1.3080524	0.930430335	0.374684743	0.231442459	0.016951377	2.697476959	4.099521683	2.330394926	0.910120088
TCGA-BP-4176-01A-02R-1289-07	0	2	1	1	0	0.195488223	0.038308994	1.617533623	1.349284425	0.971740344	0.467786903	2.56073048	4.297642229	2.986114054	0.467187416
TCGA-BP-4801-01A-02R-1420-07	0	2	1	1	0	0	0.01046695	1.119522927	0.970736573	0.298626589	0.06122944	2.617381476	4.386714279	2.598053298	0
TCGA-CJ-4892-01A-01R-1305-07	0	2	1	1	0	0.911737782	3.040697332	2.864203675	1.977353501	1.607584936	0.813213694	2.91433517	4.374393255	2.32822633	1.553570095
TCGA-B0-5700-01A-11R-1541-07	1	2	1	1	0	0.854956787	0.135982935	2.031317948	1.055385018	0.233038857	0.055741044	3.372192064	3.616156164	3.549375543	1.377612885
TCGA-BP-4775-01A-01R-1289-07	0	2	1	1	0	1.721440439	4.304943953	3.489929284	3.015147131	1.506690608	1.080060423	2.815792832	4.080172981	4.427012653	1.329840675
TCGA-CW-5581-01A-02R-1541-07	0	3	1	1	0	0.741028813	0.001088153	1.791133214	0.620205243	0.254097753	0.109009083	2.094496772	4.037608884	1.933713199	0.614262327
TCGA-BP-4164-01A-02R-1325-07	0	2	1	1	2	0.548110951	4.081125468	2.87828104	1.158629834	1.669035501	1.126275396	1.674476999	4.450059426	2.701313693	0.936187664
TCGA-CJ-4642-01B-01R-1305-07	0	2	1	1	1	0	2.64850482	2.380352438	0	2.963713167	0.664898446	3.982136609	3.046098555	2.717824047	0
TCGA-BP-4976-01A-01R-1334-07	1	3	1	1	0	0	0.011339441	1.505004428	1.139875215	0.221687842	0.099047597	3.35583597	4.73469191	2.100345296	0.48180159
TCGA-CJ-4897-01A-03R-1426-07	1	3	1	1	2	0.258546795	1.737410289	1.377808802	1.206947858	0.333152135	0.187849182	3.165253004	4.310812906	2.174821858	0.175440727
TCGA-BP-4160-01A-02R-1289-07	1	2	1	1	2	0.32539169	0.012254549	1.194101761	0	0.546172391	0.111921992	2.787031526	4.733207814	3.244031925	0.415077978
TCGA-B8-5159-01A-01R-1420-07	0	3	1	1	0	0.298049584	2.301930715	1.398117499	0.205376766	0.365136571	0.147590456	3.138357441	4.203536776	1.610170893	0.381145051
TCGA-CJ-4885-01A-01R-1305-07	0	3	1	2	2	0	0.038138888	2.203417147	0.240748128	0.468440668	0.116951346	2.918071791	4.136175303	1.796038364	0
TCGA-BP-5201-01A-01R-1426-07	0	4	1	2	2	0.937977022	0.018967039	1.301788894	1.151287273	1.485762986	0.445225798	2.345985867	3.801150408	3.768882782	1.534224679
TCGA-BP-4991-01A-01R-1334-07	0	2	1	1	0	0.804677975	0.00259224	2.090369402	2.316927102	1.712413489	0.707546081	2.768328463	4.62361426	3.586377194	2.081685116
TCGA-BP-4968-01A-01R-1334-07	0	3	1	1	0	0.862207701	0.002838677	2.694199428	1.867173874	1.698763554	0.634631576	1.979023774	4.182919473	3.14184344	1.509597731
TCGA-BP-4975-01A-01R-1334-07	0	3	1	1	0	0.245965541	0.001506204	0.888847773	1.080873911	1.303824172	0.230034106	3.701814801	4.277904972	2.059668436	1.230896544
TCGA-BP-4340-01A-01R-1289-07	1	2	1	1	0	2.482020319	1.472482245	2.79335538	2.263535089	1.252203499	1.547727531	3.109493976	3.969599208	2.931803184	0.861341025
TCGA-B0-5084-01A-01R-1334-07	0	3	1	2	2	0.177638624	1.201841781	1.137976602	2.720423182	1.300629013	2.210287279	4.288813721	4.512418261	1.941381898	1.025678471
TCGA-CW-5590-01A-01R-1541-07	0	3	1	2	2	0.974035364	0.004460448	2.079331441	2.166698276	0.835180967	0.312174314	2.101585332	4.635640266	2.668658978	1.397082236
TCGA-BP-5198-01A-01R-1426-07	1	3	1	1	2	0.181895677	0.003264425	1.157916486	0.779603004	0.259559819	0.053921452	1.222513375	4.697449465	3.09253789	0
TCGA-A3-3308-01A-02R-1325-07	1	2	1	1	2	0.93247729	3.766980855	0.636953271	2.285840653	0.95476395	0.536660807	2.718789971	4.445188058	2.730431727	2.533213243
TCGA-B0-5706-01A-11R-1541-07	0	2	1	1	1	0	0.003603007	1.491749017	0.672028396	0.231514256	0.140734929	2.184058897	3.987422638	2.760210747	0
TCGA-CZ-5466-01A-01R-1503-07	1	2	1	1	2	0.451446413	0.007427396	1.85544554	0.316076603	0.597038272	0.064270171	2.558657276	3.973837158	2.632807072	0.569605191
TCGA-AK-3434-01A-02R-1277-07	1	2	1	1	0	0.517865997	0.176100462	1.67736684	1.286557374	0.986348153	0.372386542	3.466907837	4.511728837	2.220523438	0
TCGA-BP-4335-01A-01R-1289-07	0	3	1	2	2	0.594702026	4.012269323	2.041459496	1.895634858	1.279096794	1.449162986	3.069880142	4.121271707	2.668546406	3.010461654
TCGA-B0-5699-01A-11R-1541-07	0	2	1	1	0	0.364597072	0.002329077	1.300854882	1.722542929	0.800226575	0.373385081	3.229937808	4.251239774	1.735283043	1.095288151
TCGA-CJ-5684-01A-11R-1541-07	0	2	1	1	2	0.206575191	0	1.172459827	1.401677541	0.397427443	0.156432754	3.538407809	4.164795467	2.562696928	0.685749866

TCGA-BP-4344-01A-01R-1289-07	1	2	1	1	0	0.713101778	3.402369845	2.743257149	2.886208428	0.776315443	0.428029996	3.294625219	4.796162281	3.735494339	4.256384
TCGA-G6-A8L7-01A-11R-A37O-07	1	3	1	3	0	0.393687716	1.006100032	3.680292125	3.350957124	0.586066351	0.328425433	2.84373514	2.780135881	1.908331249	3.50273828
TCGA-BP-4782-01A-02R-1420-07	0	2	1	1	0	0	2.563890811	1.029880959	0.474887646	0.596242968	0.083456362	2.338610011	4.17431464	2.901079953	0.254124746
TCGA-G6-A8L8-01A-21R-A37O-07	0	3	1	3	0	1.102199924	3.837963124	5.387790745	3.484645787	1.897351458	2.09440775	3.926396063	3.894563479	3.230814663	4.453094111
TCGA-BP-4756-01A-01R-1289-07	0	2	1	1	0	0.357379008	3.022040668	1.100862536	2.55393479	1.000445249	0.845313076	1.814714028	4.536201825	2.561378034	1.199342752
TCGA-AK-3436-01A-02R-1325-07	0	2	1	2	1	0.568514898	0.024588573	0.988089051	0.894262814	0.303425306	0.279630717	3.533882675	4.302150045	1.730704882	0
TCGA-B0-5712-01A-11R-1672-07	1	3	1	2	1	0.36282406	2.540329815	0.368644963	0.465978851	0.32707265	0.348804076	2.912014752	4.41830843	2.02980125	0.249001606
TCGA-CJ-4875-01A-01R-1305-07	1	3	1	2	2	1.160195672	0.061593962	3.643346622	3.961930142	2.532199699	1.207447107	3.02811952	4.321332492	2.399037591	2.717811324

MALAT1	MIR4645	HOXA13	TRG.AS1	HOXA10	NEAT1	HOXA3	MIR570	SNHG1	MIR4668	HOXA9	MIR4701	SNHG5	MIR4768
4.086426021	1.16447674	0.068835685	0.210886751	2.457412918	3.629246175	2.329766159	2.567467221	1.825858919	0	3.179601717	0	3.917460738	2.035779462
4.178425832	0	0.132242554	0.102840251	2.247780943	3.588971738	1.457771513	1.814213679	1.686130223	0.90446447	3.2483253	0.787884826	4.025442636	1.796667351
4.739927055	1.464770091	0.042615937	0.108277987	3.570118999	4.132151495	1.88100518	2.376235954	2.221141003	0	3.312139236	0.620902879	4.748120426	0.754316954
3.84509945	0.496100407	0.112062236	0.116585646	2.003302004	2.684315539	1.019644923	0.983557371	2.693469179	0	2.927722907	0	3.049294121	1.546784388
3.29034579	0.33459985	0.005690083	0.048328708	2.293898827	3.473791533	1.491118558	1.465328586	2.247477606	0	2.790547609	0.213523805	3.92807592	0.747623278
3.745247653	0	0.011508861	0.089648313	2.337659094	4.541666492	1.929652988	2.517129126	1.811754334	0.788610926	2.875954323	0.570404934	3.568237767	0.350764701
4.394545444	0.260123503	0.095991791	0.092622753	2.155625976	3.845935898	1.633303623	2.153981084	1.699568831	0	3.40234883	0.3120705	3.693901473	0.693117855
3.34782931	0.554900469	0.02034642	0.155346657	1.661292949	3.403867301	1.092452943	1.516903635	1.486984193	0.600245017	2.880004021	0.363622155	4.567595861	2.22052387
4.272535786	0.256441179	0.168248519	0.355313662	2.416747272	3.916261066	1.57665776	1.347209064	1.830363683	1.049565468	3.296749876	0	4.354769537	2.335795972
4.526248779	0.416091199	0.050228591	0.128943693	2.234091366	5.007769223	2.218968754	2.354271886	2.148476828	0.451792178	2.56677317	1.015422765	3.870040634	1.626281718
3.995749154	0	0.039587882	0.223413773	2.1938307	3.605684572	1.927524481	1.845283635	1.763080453	0.535242273	2.519435166	0.321405742	4.418226787	1.18568618
3.37715603	0.601852253	0.055454057	0.145566538	2.036377673	4.035307204	1.96251298	2.029264726	2.425540458	0.361448462	3.302014937	0.396543256	5.349090989	2.170318079
3.452638275	0	0.010466241	0.063271079	2.974436727	3.325699588	2.287304973	1.718348496	1.803681701	0	4.000438496	0	4.327451933	0
3.731545467	0.604960045	0.082907995	0.101438744	2.211113601	3.072166901	1.593011649	1.163872914	1.346636196	0	3.262208904	0	3.880179503	0.858445389
2.332304559	0.688499681	1.274879989	0.60836498	2.188122014	1.693314439	1.470800292	2.992562684	1.551340008	0.291905006	1.821408414	0.320973392	4.91669732	1.043112943
3.198173297	0	0.003961255	0.033795354	1.677739362	3.182218391	0.908306413	1.52313182	1.253986718	0	2.055828699	0	3.585684933	0
4.2624701	0.38449777	0.013279909	0.072100268	2.674156584	3.466336412	1.074441114	1.826017042	1.993983165	0.224006799	2.919927079	0.457618569	4.491339674	1.183420621
3.138763504	0.777047956	0.072200058	0.070242282	2.338507783	2.330036106	1.357957772	1.354918692	1.189091205	0	3.089216001	0	4.37060955	0.455518684
4.294828658	1.89229997	0.05235517	0.02889028	2.041062048	3.506954689	1.546280494	2.15213702	1.55335097	0.675091876	3.107485218	0	3.959151992	0
3.316815856	0.233866407	0.034185734	0.243349197	2.138550993	2.446314279	1.203404913	1.75482535	1.967699701	0	2.852563525	0	4.309419246	1.301549112
3.014488882	0.23456862	0.041807431	0.14877293	2.603887957	3.111563775	2.409439543	2.418110659	1.993653552	0.256057004	3.359468199	0	3.991722385	0.451461736
4.955727203	1.14450368	0.231592718	0.559756181	1.013281748	5.097685284	1.947767457	3.43975402	2.681891502	3.413504404	0.977814644	1.310191906	4.053705409	2.928628287
6.163650285	0.732347283	0.04736352	0.685521531	0.525652963	7.061506343	2.055739821	4.181848748	3.259661329	2.053940884	0.631512195	1.529093026	4.264523511	3.060095345
4.97951314	1.329092026	0.418918462	0.728237085	2.358657888	3.734137837	1.861960207	4.182011971	3.116323587	1.536729044	2.216313525	1.638130207	4.712121774	1.886696419
2.796189524	2.128270056	0.041483036	1.183201245	1.725226172	3.256095208	1.943687392	2.432268056	2.65150589	0.339065875	1.788767729	0	5.938044442	0.3226393
5.321728557	0.705121906	0.060656167	0.837797054	4.015563119	5.023347623	2.743590791	0.585277242	4.875871933	0.429380646	3.857865892	0.470065743	5.988818755	2.787121
3.719006435	0.643926054	0.132942555	0.236716551	2.169243259	4.073532681	1.888610617	3.312028148	2.915895029	0	2.186330665	0	5.578518113	0.257164655
4.882448571	3.085820244	0.168682928	0.752884452	2.633954318	5.106655058	3.196337968	4.631320907	3.2241627	1.193708723	3.24477208	1.441766806	5.612445936	2.612306662
3.018660551	1.95403184	0.099627249	0.696129959	1.767831347	2.263844044	1.824064376	2.247556798	1.971311067	0.293792046	1.864374855	0	4.143244383	0.513287796
3.793615212	2.216346806	0.03929032	0.680113464	2.790742402	3.584664778	1.671211146	1.291356554	3.561863395	1.587813117	2.592886477	0	6.240616693	0
5.204992041	1.271429626	0.373423712	0.954953334	2.501852818	5.521143911	2.185884413	4.632042935	3.511803491	0.951024006	2.606409916	1.773794848	5.711915661	2.179891541
5.862565997	1.65543979	0.205391653	1.255232473	0.65640428	5.105199052	2.529936787	4.228916893	3.247720943	1.274486368	0.512325099	0.609379117	3.743797051	1.966641103
6.241292439	2.427575779	0.011908803	1.348467839	1.521408589	6.929076902	1.837866915	3.64581198	5.478202171	2.004023859	3.08300073	2.226607612	6.123639622	4.464550948
6.323006994	1.995931551	2.213979906	0.454885179	1.071251412	5.842295314	1.450731183	4.103908995	3.084576961	1.000415979	1.376015463	1.280597294	5.003482005	3.029681324
5.59394842	1.089623942	0.067397479	0.982565505	2.416839541	5.324906444	1.829467664	4.035958467	2.971107956	0.994799306	2.393976754	1.072527577	5.066766407	2.018705129
12.32690828	0	0.043588239	0.30945359	0.913631857	9.425610076	0.83591934	8.362384011	3.196718868	2.94224795	1.547376726	0	2.349000601	1.636090177
3.716750488	1.082797551	0.956594419	0.304825864	1.975645692	3.732483069	1.932205517	2.508827853	3.037513821	0.767934828	1.911194041	0.2572891	5.3321548	0.872670846
4.686162105	0	0.086397379	0.510294474	2.295459968	5.353676185	1.297425463	2.901468946	3.488423525	0.458038955	2.188354598	0.500998391	6.186165581	3.536580506
4.539445189	1.723073734	0.218791082	0.471099518	2.689205909	4.152283764	2.166657127	1.830915517	3.787074895	0	2.930282142	0.643913516	6.38034004	0.309722186
3.035462971	0.289112217	0.042971606	0.271240249	1.907484718	3.159016554	1.484095639	0.769392639	2.222295438	0	1.929419993	0	4.321442933	0.299694344
5.704915752	0	0.023439358	0.203243105	0.18248504	6.935587299	0.219644614	4.462143954	3.125000234	0.375761411	0.237282111	1.215572766	2.448250319	0
3.711424357	1.188739265	0.107669871	0.304021881	2.098408365	4.157430858	1.213783922	2.719494816	2.574751923	1.524390025	2.363784276	0	3.488797913	0
5.687111558	1.700968302	0.178185121	0.860322039	1.637336214	5.35716213	1.988205308	4.455080929	2.927162729	1.469257151	1.828105768	1.363036678	5.198752574	0.41644686
4.879622974	0.754979145	0.108458523	1.360206913	2.692228679	4.213508612	2.132386489	4.228014929	3.954999421	0.960111241	3.177119363	0.880026257	6.10934538	2.249835655
2.792979579	0.565095634	1.020334645	0.533043069	2.906314534	1.916141801	1.945735637	1.599311631	2.785456233	0.233656645	2.779671029	0.257428248	5.461127893	0.221948903
5.615079876	1.663528531	0.60449872	0.465806444	2.830113633	6.337944187	2.23248661	2.564840353	2.881287385	1.240425708	3.07452637	0.813151778	3.805480026	1.552640614
5.78198557	0.906995674	0.074501144	0.558921045	1.683312807	5.474101853	1.252383712	2.811614679	3.557829115	1.54858481	1.794683392	0.776587677	5.095006285	3.576205381
5.410583602	2.473552822	0.964025932	0.9144264	2.499551376	5.163765617	3.040264472	3.932644057	3.371682381	1.636574407	2.670850679	1.118901135	4.402389388	3.206395666
6.193407064	0.515217447	0.969722527	0.799256925	2.72415771	5.435798427	2.294736627	5.207314746	4.54151038	1.124544014	2.951864613	1.035045922	5.97930218	4.688563694
6.525903114	2.662911123	0.308227809	2.103035963	2.490410877	6.044471842	2.912236296	2.835632944	3.821876011	2.176272937	3.028253819	1.563386998	5.284057679	2.937186944
5.756322383	1.807164576	0.11248665	0.979641559	0.946452721	5.565997639	1.262055139	4.027370892	3.352788949	2.117252508	0.945515685	1.669995752	5.376386624	2.632673453
5.474861869	0.613031336	0.082407857	0.359500933	2.569098136	4.613134166	1.736509959	2.868787091	2.940253232	0.829082568	2.482777614	1.198028124	4.816072625	2.892890084
7.45097725	1.224485633	0.068422922	0.55926322	2.003531851	4.549117739	0.614147141	2.204068476	3.325776674	0.371925326	2.059009472	0	4.699145644	1.257561414
3.391244904	1.362328086	0.922863179	0.787211493	1.987642992	3.098633425	1.589850965	2.286823626	2.031474417	0.468866415	1.839195503	0	5.18189845	0.240780579
3.684628534	1.408364878	0.166202607	0.086359939	2.181151723	4.577782671	2.17291086	1.858582665	3.37892977	0.295693299	3.032270372	0.590242089	4.663388508	2.079866756
5.648887311	0.438531197	0.097493908	0.674509123	2.48840856	5.647913131	1.51660614	2.957462253	2.978516593	1.901194705	2.306309832	0.520208516	6.106246013	5.659060542

5.463888198	1.479483747	2.245234102	0.879059041	3.16786696	4.752994527	3.468445186	3.681569802	2.46519471	1.208866085	3.021987776	0.448184075	3.584923879	3.180197915
3.879144316	1.423767435	0.014628209	0.434457218	2.343634506	3.525674595	1.682039067	2.990325963	3.054321407	0.454415367	2.448277465	0.497089918	4.889616392	0
2.244929883	1.429436565	0.041021903	0.181651677	1.780668919	2.839896816	0.648724687	0.879831855	2.419044721	0	1.730844727	0.331953026	4.802432699	1.465748323
11.89457248	1.682468462	0.212769984	1.330021813	2.329910766	8.659886382	2.162638395	6.658015065	3.648526462	3.422633145	2.771481343	2.129623464	4.461096881	4.400492095
3.91984101	1.887274279	0.127807922	0.5977316	2.565398091	3.378131276	2.468363847	1.836467766	4.334079612	0	3.233225634	0	6.886558522	2.726303607
7.165540737	2.317012168	0.103651174	1.034472611	0.654676326	6.141705751	2.029833921	3.750717917	3.927641132	1.194474761	0.757784184	1.838842846	4.949324726	3.920287307
2.529196608	0.299495677	0.078455047	0.186052198	2.664164555	1.858878113	1.760750239	1.405065341	1.190150575	1.010881586	3.277649618	0	4.943125674	0.782578135
5.087385529	0.886069878	0.789682311	0.347970298	2.483788253	4.081084519	1.828526362	3.28575729	2.130095959	0.765289547	3.023449341	0	4.702287896	2.436319771
2.721239909	1.015077079	0.017731183	0.916146477	2.740653056	2.040074702	2.487551708	1.199618364	2.139996633	0.535301291	2.682441555	0	3.716994111	0
3.635228075	1.024537839	0.162220888	0.846669996	2.329447047	3.264997188	2.296795347	1.858917769	2.066626555	0.750729131	2.537636942	0	5.528830526	3.01848771
5.013782232	1.725750164	1.557607549	1.347589422	2.920457703	5.102044193	3.094219418	4.840094082	3.540413501	1.386956839	3.209396267	1.725750164	3.683858036	0.726689069
5.810995928	1.4150154	0.024066248	0.558752839	0.917269358	5.931875174	1.18999133	3.722566878	3.266816192	1.01327898	0.925335404	1.893743829	6.006118424	3.410666529
5.43006827	2.234643552	0.130283061	0.569223682	1.809070365	4.115521953	2.28660632	3.076052624	3.930272891	2.076822897	2.255147082	0	5.508252192	3.220702778
4.749303832	1.251535182	1.983260686	0.451864219	0.8829477	4.804880681	1.972504917	1.444997726	3.364838878	0.28333613	0.517315329	0	2.475466984	1.704001697
3.153572038	3.16068793	0.536974846	0.637198562	3.591391318	1.970975401	3.164922181	0.744614961	1.913819693	0.391799776	3.617999386	0.759921188	4.564553531	1.307902675
3.045538181	2.446749261	0.043892359	0.667140558	2.25345415	2.806848366	1.518112871	0.527815139	2.738481548	0	2.601481452	0.748586355	5.062003694	1.347163648
4.110877067	2.2469109	0.864582644	0.887963034	2.427878348	3.129855997	1.526007878	2.052797709	2.063769195	1.431362949	1.661104802	0.621744387	5.136566906	1.102074219
3.909512152	2.710148757	0.234559233	0.83808826	2.269024056	3.67239588	1.848584047	2.790427012	2.843267851	0.705629917	3.052980513	0	4.459428517	0.261733882
5.390110607	1.502093466	0.051117282	0.909245848	2.222066501	4.163346952	1.780212795	3.282165982	3.005213091	1.385502964	2.612353289	0.741513818	5.146811513	3.592109267
3.295767779	2.201814114	0.086484092	0.713666403	3.054747099	3.702293902	2.465754915	3.2302059	4.300805547	0.338044572	2.882953543	1.120033812	6.07574606	2.522463188
6.545197746	1.578560367	0.038065392	0.660199235	2.352863071	6.126525805	1.882906366	3.338002575	3.215063136	2.055428905	2.291142014	1.056031382	5.817076766	1.994956573
3.420156629	2.93969998	0.071089369	0.582663374	2.680915626	3.745438783	1.496294274	2.62231018	2.069210633	0	2.639576526	0.280496866	4.870893542	0.63052991
5.295699453	0.286837684	0.193073629	0.724266553	3.241407392	5.521410327	2.89357381	4.047912618	3.531185515	0.976611663	3.324719101	2.252117551	4.571732496	3.663676436
7.599832655	2.235995775	0.049322451	1.162697399	2.334823679	7.008661772	3.349388384	4.874565367	3.646571142	2.964079774	2.808576289	2.772176773	4.688076364	3.296585222
4.742622867	2.703308438	1.905752641	0.656708559	2.583452069	5.00367496	2.544690194	2.900961161	2.7142365	1.368474161	2.659782832	0.370675462	4.853123566	2.456644288
5.883257963	1.454153937	0.03339178	1.450872059	1.940775986	5.521188801	2.024997776	4.312988555	3.745579937	1.91249674	2.48163933	1.939409997	4.965882886	0
4.827848248	0.980820711	0.112457952	1.438714313	1.260254278	4.214083188	1.868145504	3.026595504	3.150371579	0.34225508	1.523449183	0.375718042	3.718221688	3.060854566
3.37580658	0.888740192	0.120382099	0.771044603	2.247894102	2.482123782	1.217106912	1.333969708	2.415163894	0.554202985	2.009069716	0.333650945	5.379321775	3.235385736
5.848986484	0.556831341	1.355120097	0.797169355	2.617452493	4.494520722	3.032216321	3.835313111	3.249774886	1.352992772	3.125867425	2.24552898	3.469035194	3.259617251
5.345135292	0	1.506267412	0.566418524	2.123134354	3.690378176	1.968861401	3.407322142	2.662292965	1.109660121	2.823546424	1.322530291	4.232520076	3.41625158
3.359283099	1.012303971	0.544869486	0.995436878	2.638514252	2.9155185	2.013445495	2.586394071	2.19607907	0.740975653	2.495346224	0	4.72776383	0
4.592246707	0.227863563	0.981108559	0.460919432	1.712864327	2.826954133	1.395484596	1.665910757	1.984382092	0.248789067	1.739863041	0	5.603204156	0.617399733
4.463420139	1.749768779	0.209787912	0.240905705	1.549200502	3.730288337	0.820396351	3.238969067	3.710030175	1.20143039	1.062663359	1.488054804	5.174636389	0
1.046755589	0	0.048425544	0.015082656	3.000308278	1.566430956	0.196804616	0.291285006	2.925688115	0	2.411506618	0	3.341177986	0.371020598
4.654148578	2.20902887	0.083042151	0.563958976	2.387665533	4.434626325	2.071630063	3.783629269	3.084428283	0.213428327	2.493546623	1.560085273	4.194437103	1.039666388
2.286746111	2.151106606	2.361224204	0.317273301	2.769855102	2.226571517	2.081024001	0.541359222	1.866260327	0	1.982880249	0	4.768582191	0
5.215978393	0.663733932	1.242712711	0.521711861	2.730391896	4.609724078	2.637642185	2.662861174	3.099093083	0.715976968	2.91628505	0.440417873	4.395318248	4.35413685
4.132870803	0.27409507	0.088623032	0.475188485	1.72207489	4.936678857	1.354161904	2.803100068	2.960861628	0.75743012	1.607767614	0.59594682	5.38551879	0.725220796
5.690853045	1.101493596	0.126561145	0.923867222	2.231338508	5.325576251	1.615180197	4.129399317	3.258023624	1.485679993	2.559062732	2.070689372	5.030734889	2.194292539
5.928943678	0	0.041750993	0.53803056	3.168735173	5.55050211	2.854436316	4.918940119	2.805659074	1.047324882	2.999282134	1.127775689	5.051854851	3.244262038
6.209691985	1.439904833	0.166410848	0.49166518	2.631105206	5.462664852	2.504273366	3.593698278	4.221050644	1.65696447	3.190743071	0.923948775	5.89741462	2.107793011
5.202462756	1.65863061	0.156144502	0.788503788	2.041896144	4.60314459	1.339207214	3.531037114	2.019729432	1.253494496	1.620433403	0	3.278638368	2.765237933
3.153934037	0.823998531	0.156123162	0.43647759	2.116925858	2.827801395	0.72260798	1.372867142	2.027231489	0	1.982929275	0	4.921656095	0.401208556
5.14402623	0	0.137626197	0.34786116	2.585461727	2.425032723	1.46416681	2.169540965	3.944834501	0.526828241	3.188742407	0	5.892766206	0.273177744
3.862111274	1.281121346	0.243739262	0.411419978	1.867033767	3.177518686	1.361624295	1.094489658	1.779756102	0.259017192	2.001500421	0.285123647	5.078739422	2.294749643
4.652537807	0.288111237	0.066055476	0.406609223	3.061905716	2.827868693	1.873454614	1.155915536	2.348204197	0.313994259	2.090533149	0	5.614241552	4.469896541
3.334791629	0.856793211	0.011761198	0.228191443	3.235316593	3.718209491	3.273164357	2.403795152	1.57994782	0.375589688	3.897477851	0.411871803	4.349994454	1.57001104
4.055462996	2.078188964	0.946384169	1.491893806	2.591251191	3.090524519	1.430430462	3.315273335	2.652022163	1.12544055	2.341695322	0	4.400833325	0.355525713
3.503217738	0.918136469	0.129988565	0.980472167	0.56004595	2.180310046	1.35563476	0.556624053	2.282919692	0.407312903	0.371851051	0	5.236029974	0.693462805
2.133045766	2.97671134	0.009350555	0.550344563	2.214082005	2.387391932	1.807317767	1.759823672	1.949189458	0.305937213	2.101587904	0.336247148	3.495688165	0.740157509
5.751266652	1.429641287	0.096581951	1.462475109	2.626527917	5.653523762	2.172299086	3.879168521	3.443461552	0.456948469	3.099224414	1.292273875	4.837315408	1.46595605
6.086859522	2.633807542	0.156863741	0.334235474	2.176159635	5.534367154	1.778783709	3.479694976	2.804570443	0.72643572	1.868261471	0.932886759	5.636611016	1.258894145
3.887041984	2.353009586	0.060134063	0.467834562	2.753873496	3.441052126	1.931782297	2.35615403	2.164993667	0.307875017	2.885193859	0.338355376	4.592053177	0.926073839
5.453668253	2.471494111	0.131712617	1.89231193	2.241847805	5.500555606	2.282743225	4.173260249	3.506628513	2.205294425	2.848428546	0.693660727	5.113204194	2.379863247
4.462705759	2.672606151	0.112883506	0.698984361	2.995955839	3.692812137	1.840542526	1.671626139	3.627531942	0.657104912	3.186311737	0	5.883518497	0
4.534921331	1.9385065	0.41493242	0.580818684	3.1492567	4.169707243	2.289607469	3.16314523	3.525681491	1.658784664	3.320684192	1.377485434	5.882333037	1.239146418
2.924814076	0.856393673	0.098361354	0.678674136	2.719218963	2.777922946	1.361163019	2.182265273	2.048318707	0.290293927	3.002811863	0	4.672937744	0.707104169
4.267091287	1.072192773	0.406558299	0.167800672	3.448012837	5.919063604	4.31913907	1.605173047	5.474828527	1.495354472	4.459059036	1.795226951	6.588398254	3.747321321

6.665642539	1.280960505	0.336728548	0.165917772	1.993881235	6.239962882	2.403907345	2.511672138	3.461746459	1.23161204	2.39462118	1.699175786	2.69302534	2.924029009
6.207669598	2.264739101	0.095036057	1.532647149	1.541794225	4.621397851	2.30748028	4.204720422	2.906957598	1.796468506	0.90696113	1.638891211	4.872062753	2.021468518
5.043182803	0	0.507620462	1.157404533	0.944457195	4.265351998	1.635086626	3.620108057	3.246551924	0.736459187	0.621767067	0.799162613	5.278215937	4.424755047
5.280772192	1.375886631	0.117071701	0.810215917	2.383869799	3.999431271	1.710450006	2.262749924	2.212752969	0.664422444	2.262582338	0	3.051500051	3.490100131
6.511445466	0.513276725	0.245427371	0.354817373	3.01005683	6.50726871	1.853255744	4.661546858	3.18675151	0.834599411	2.603170564	1.781202558	4.752193576	0.199378461
3.49494569	0	0.314826827	0.405061172	2.024897038	3.542777599	1.316419773	1.86333191	2.690728519	0.35754469	2.249967835	0	5.309957287	1.517168875
3.097317133	2.025032606	0.115030226	0.417107883	2.255809521	2.039040995	1.005594441	1.627080694	2.072203278	0.567964237	1.761900583	0.342573874	4.239417262	0.752345629
3.414939738	0.935841023	0.058617973	0.366673393	2.459255611	3.05091286	1.270368967	2.264528613	2.065774294	0	2.775847306	0.354986827	3.93942353	0.963488358
4.21833259	0.726392074	1.14331179	0.375097118	1.970252649	5.665562511	0.526910238	2.414019629	3.7940911	1.545897143	1.859488202	0.616633684	2.900988154	1.240045544
4.954962395	1.949532825	1.460508602	0.40492848	3.242498757	2.88495563	3.045724829	2.291412938	2.950792985	1.087158216	3.334212651	0.321813022	5.808991775	2.138829977
5.81614155	0.625957533	0.08075383	0.571578828	0.54113177	5.588540212	1.791153429	4.288564253	3.724711836	0.997061393	0.800008684	1.350058727	5.80577516	2.513612394
5.251639689	2.122207862	0.197456541	0.675778131	2.76494067	5.143755991	2.076676062	3.542076587	4.674477492	0.495576546	3.035451552	0.541442233	6.782522909	1.351013537
5.796820495	2.095596759	0.053480766	1.067525329	1.149025785	5.583974952	1.573681677	3.616147153	3.269872544	1.346798589	1.255287913	1.440935017	5.663765266	1.559110008
4.139282347	1.636359827	0.156959898	0.955810424	2.973644068	4.445185967	2.760673353	1.741401862	2.341443483	0.762055728	3.257185793	1.022169652	3.64601528	1.861223197
4.060054442	0.789839498	0.046986818	0.6924714	2.397659404	4.387434366	1.167941463	3.877102808	3.354659	0	2.443805686	0.375125054	6.082578969	0.325168165
3.233043185	2.270167496	0.089063832	0.151210806	2.435132299	3.431426788	1.089649112	2.433307234	3.411689303	0.275374854	2.648114499	0	4.167212767	1.480505266
4.021722544	2.043602157	0.118611581	0.35224711	1.37924884	2.890647408	1.5915648	2.035128153	3.074172897	0.280585933	1.147664933	0.778735968	6.02847651	1.392134294
5.682623553	0.778584713	0.051170602	0.570117746	0.506966886	5.596683069	2.526945268	3.279218	2.768298713	0	0.334207976	0.662622039	5.641488154	5.159511497
4.902224137	0.345398042	0.080431142	0.61784786	2.057742107	4.732588533	2.683113608	4.947067065	2.457277392	0.920411337	2.62712319	0.412090932	4.225932056	3.648485864
5.143202277	0.452337265	0.243648065	0.678752688	1.906849535	4.225495929	1.545756141	3.69881585	3.207923881	1.147464264	1.93273316	0	5.328198351	2.625969286
6.228000013	1.759698622	0.033122921	0.44937896	2.233681631	4.232990239	2.492314627	2.086546153	1.872883654	1.209422535	2.421949761	0.874444427	1.898859676	2.660094751
4.859055962	2.373497234	0.04200849	0.913527321	1.144259963	5.424344937	1.189832618	3.566126879	3.171111108	0.690062842	1.126693011	1.240624776	3.565750552	0
2.435663989	0.528061304	0.028674749	0.441973677	2.689032357	2.956558468	1.816097308	1.713950029	3.264913518	0	2.791036183	0	5.812588711	0.545878113
12.15577746	0.639716476	0.266346208	1.623287939	1.006057652	9.41018149	2.260585905	5.723206301	3.601973795	2.563305048	1.015321513	0.750201755	4.014648515	4.822237516
5.616979651	0.664273938	0.4135883	1.037094954	1.951061851	5.004325684	2.403962889	3.854173114	3.445750825	1.383009642	2.450764137	1.051079194	5.447918346	3.615514268
4.208468546	0.790641632	0.243862805	0.246062416	2.917270316	4.189431898	1.624719859	1.811701914	2.806292711	0.850261758	2.230236103	0	5.456459291	2.33637006
4.0293531	1.486274756	0.069148296	0.288883077	3.243752613	3.679139465	2.501263776	2.441891221	3.309763787	0	3.557854566	0	4.918379838	0.247889348
6.211587143	0	0.087484412	0.890527971	1.523703396	6.10368474	1.755447846	3.417473038	3.006096094	2.358760844	1.732775527	1.503472232	5.248333698	0.832864236
6.518638814	2.457961043	0.408311377	0.789405242	0.090353039	4.779407125	1.347980806	3.310237173	2.927052891	0.964019829	0.09802705	1.040103561	5.080959046	3.396854491
2.285075029	1.906846266	0.075706364	0.514245762	2.208527749	1.649587805	1.43030656	1.191599147	1.680424389	0.236258365	1.757394328	0	4.987088936	0
5.117424795	0.276283967	0.115145083	0.834396358	2.643388679	4.968019459	1.85274766	3.036453536	2.921656607	0.762591825	2.750712709	0	4.543468539	2.242962971
2.993494144	0.703330668	0.062657032	0.816694198	2.364535613	2.734565852	1.627432577	0.735489227	2.488236425	0.299082647	2.574420221	0	5.657757029	1.063191714
6.062644153	2.211655955	0.054938862	1.452452523	1.127571876	5.524371012	2.202493826	3.742086348	3.398939942	0.956104441	0.876884898	1.205170278	5.100247474	4.790641695
3.499767323	1.466990574	0.019141812	0.239924081	2.231298439	2.847248854	1.409512256	0.765807551	2.289391749	0.404338389	2.351664794	0	6.325172472	2.768487171
6.311615877	1.299915166	0.307048849	0.51830416	3.069213332	6.324895058	2.395560174	4.484751	3.406057451	1.617246488	3.226976887	1.186801589	4.739532918	1.935646274
2.540147153	1.562214823	0.316632343	0.455664201	2.352052447	2.566820837	1.26542355	1.980285121	1.564575413	0.280763389	1.914719169	0	4.916215287	1.011583999
6.91767565	2.009028937	0.071608589	0.47356463	2.966104072	6.348228211	2.079864829	4.132809053	3.047835183	0.453897767	3.25597174	0.865234206	4.761903377	0.765110684
4.946528131	0	0	1.815955665	0.998996049	4.916785181	4.409834921	0	3.01504504	0	0.938549392	0	5.194062582	2.858600192
2.850066919	1.672832324	0.050104973	0.134775033	2.494812211	2.039156359	1.521331179	2.004004096	3.164261788	0.214724911	2.060384815	0	5.420995305	0.54143915
3.550142683	0.892557712	0.186893048	0.315963091	3.049474107	3.168071672	1.483878947	2.312064182	3.087493882	0.394005873	2.287677818	0	5.045522238	2.666101227
3.709183134	1.305694302	0.023914504	1.146930178	0.514063768	3.169404569	0.81832844	2.074991399	2.205935239	0	0.224523517	0	4.927509667	0
3.082584698	2.001483955	0.105412395	0.421334072	0.272620639	1.86771895	1.368347258	1.81442532	2.106051675	0.243163194	0.172844707	0.267815285	5.632881434	1.147716802
4.544593918	0.481536215	0.079959264	0.217257149	3.409434744	4.002012564	2.600757945	1.363263008	1.574310107	1.206366738	3.010930274	0	5.692403503	1.022000169
4.741929991	0.816573166	0.045012971	1.25965389	2.740773274	5.154016408	2.228196659	3.995988382	2.664695941	0.504538713	2.161397366	0.301697985	5.302080523	3.12478441
6.263658053	2.880092252	0.10457122	0.730839215	2.402379804	5.400009049	2.135378895	4.328571865	3.768927813	1.242142976	3.307849305	1.711859329	5.161405025	2.080389734
6.717270106	0	0.086439188	0.982463917	2.130288134	5.870713611	1.978767297	3.439295934	3.138105493	1.31929334	1.894231785	0.501209395	6.026661332	4.167939742
4.540830487	0.182933716	0.169732647	0.397892534	0.686484969	3.953851566	1.524041958	2.936133832	3.374003738	1.029249728	0.536253578	0.220617419	6.2543909	0.882857205
6.391990551	0.927199315	0.102601289	0.396172365	2.880392145	7.186299446	1.994873475	4.902738915	3.159121462	3.644337652	3.368495076	1.07171614	5.039283537	2.808187618
4.823204668	0.251677552	0.06511382	0.266088504	2.713307851	5.247892798	3.804053886	3.496714037	3.781688862	1.30307846	3.599229716	1.517806654	6.293280427	3.549562959
3.700996908	0.486209188	0.29575256	0.735512582	0.902959313	3.66033483	1.447367585	2.880209866	3.013979513	0.526905488	0.915580508	0.316039745	5.373899916	2.72891892
3.640689429	2.971006455	0.972012627	0.397288484	2.37679455	2.564654085	1.552269263	3.078496611	2.298284367	0.280953377	2.842169402	0.309043604	4.776625925	0.687175487
5.01004128	2.457645438	1.105963869	0.687991102	2.677326985	4.091704871	2.316018414	3.263363891	3.315656568	1.601769201	2.825239726	2.285840653	4.679196321	2.97737456
3.962546708	2.287913666	0.09571332	0.775660677	2.260418365	3.251262124	1.580506851	1.345615923	2.300887711	0	2.278032071	0	4.412724265	1.594771324
2.697650821	0.341652937	1.045404201	0.869464788	2.890671386	3.098916288	2.436979844	1.54053298	2.68696068	0.371757791	2.834666373	0	6.449224651	0.875088932
4.747505617	1.67736684	0.112141264	0.577105956	2.538005851	4.669801158	1.403545209	4.205666685	3.360555535	1.252097321	2.421999243	0.821879968	4.679436174	4.537422424
7.825968793	0.455255851	0.146298727	1.002182379	2.882349342	7.389249003	2.89317178	3.675932124	3.903523887	2.912660643	3.31754503	0.931268725	5.385370439	4.474677014
3.494348732	0.876902024	0.262367078	0.458661184	1.923281993	3.515384596	2.134715168	3.242693332	3.187550969	0.757068297	2.277556661	0.595647066	4.701661507	0
3.372192385	0.29151026	0.196321357	0.719922274	1.950514591	2.171972736	1.671868694	2.444611414	3.511803976	0.317666354	1.993765948	0.629810814	5.637913575	0.950091267

5.720780786	1.259959759	0.167600487	0.438741326	2.927151114	5.385286667	2.259197153	2.667182317	2.961910366	1.188179169	2.879520636	2.044599828	5.056234406	2.865834568
4.972118341	0	0.068139318	0.666374444	2.828764795	5.527115776	1.931841368	3.811628658	3.494731127	0	3.317251872	0	3.383030922	5.015283629
2.981920458	1.73935555	0.098714375	0.848607675	2.515217273	2.3305305	1.929708524	2.429850598	1.574798816	0	2.418376926	0.333278678	4.328892643	0
6.522619858	0.353906459	0.573617628	0.45754366	3.243618193	7.790816572	3.490096984	4.351983639	4.344513145	0.384953996	3.451121163	0.748114156	5.066820457	2.633936554
6.507996621	2.511461883	0.017751165	0.520556305	2.330510086	5.351858613	3.399487678	4.160062922	2.425247994	1.972105262	3.726688376	1.584437039	3.310959017	1.739821047
2.643366346	0.303552792	0.145823154	0.399141462	2.449043061	2.233726401	1.264746638	2.347580555	3.616205944	0	2.17766883	0	6.558233077	1.879867014
4.01177385	0.501733308	0.1467139	0.435971689	1.8464197	2.835935479	0.690268456	1.575772011	1.992766578	0.937351986	1.822729767	0.326732302	4.98647784	0.721733611
5.845050576	0.597598668	0.08171026	1.421799063	2.956740072	5.416383616	3.551871524	3.25625991	3.531427641	1.429506761	3.862767024	0	5.271573578	1.977098779

The validation set

https://www.ncbi.nlm.nih.gov/geo/query/acc.cgi?acc=GSE151419

	age	grade	stage	M	T	MIR324	XIST	MIR210	MIR25	GABPB1.AS1	LINC00894	SNHG7	FGD5.AS1	MIR22HG	MIR126	MALAT1
001C	-	-	-	-	-	20.03255	0	10.993069	14.395685	2.023725	2.988192	4.974711	7.011268	9.213046	1.778291	92.194867
004C	-	-	-	-	-	22.21272431	0.03694517	23.46471422	21.94828711	0.75124238	2.27431879	3.56426039	15.19911397	6.23607729	4.33801439	88.31860374
005C	-	-	-	-	-	13.345747	6.836745	7.55248	20.439647	2.350286	1.716816	3.836787	5.710112	8.23539	3.909519	77.032704
006C	-	-	-	-	-	14.8269868	0.01896991	8.60587346	12.39655581	0.47941213	2.69486469	4.61884854	8.61593684	18.0389022	10.02331144	80.60818422
009C	-	-	-	-	-	30.80111507	0	18.26066108	8.69555289	2.52985467	1.41510737	6.38807936	5.33833397	20.30321325	15.03819147	96.7992325
011C	-	-	-	-	-	49.63599324	16.63643284	32.9129024	34.18294051	4.10592902	4.71884365	9.51040654	5.62044671	4.93347661	30.84332842	223.1328725
012C	-	-	-	-	-	11.05212447	0.01378678	8.33933028	10.92055156	0.58203953	0.75440407	4.56024137	7.77698215	7.8338317	4.31682979	38.19137039
016C	-	-	-	-	-	1.12476713	0.49107487	4.24343961	5.55688521	0.29345167	0.47246267	10.50654162	22.28818896	6.75312292	2.19660403	16.34657274
019C	-	-	-	-	-	35.20949023	0	14.68185203	22.88837476	2.14433121	0.63246194	5.00899834	11.73583461	19.93450199	6.33334793	75.73665171
024C	-	-	-	-	-	39.58230937	0	20.18017001	7.39939567	0.59956684	0.91278327	3.86233127	11.90594634	18.22838398	5.22310283	67.15997459
028C	-	-	-	-	-	4.1274048	0	9.3429436	1.0195673	0.7129077	0.7720525	6.3271833	13.9039485	8.8052915	11.0832958	36.5465076
030C	-	-	-	-	-	5.8094445	0	5.0097028	0	0.6696275	0.42487	4.8511679	23.1580369	14.9485772	0.8103931	27.5848368
034C	-	-	-	-	-	0	0	8.78528018	5.75226678	0.39377529	0.76417904	19.90588799	8.94622664	13.99731093	5.68459305	180.002915
035C	-	-	-	-	-	8.818724654	0.007333842	6.654128603	12.19923577	3.740938008	1.273366378	15.16130568	11.29814235	5.49088174	3.4444901	85.04211966
045C	-	-	-	-	-	8.87340631	0.01229886	13.39077679	14.61295086	0.50016969	0.15530449	6.21512847	7.94242786	6.90616084	5.77641352	58.7736055
050C	-	-	-	-	-	2.9083383	7.9633628	12.069604	4.3105728	0.5269342	0.4199451	5.5278508	23.0524496	8.9733827	0	27.4064793
058C	-	-	-	-	-	10.4264813	18.7833105	11.8008811	0	1.2593835	0.5474604	9.5602075	11.9327431	10.5783741	0	42.5779745
059C	-	-	-	-	-	0.72539233	6.58147903	3.83139038	2.15027011	0.18224537	0.05713196	5.08542593	24.71197194	3.205962	1.41664855	6.80816534
062C	-	-	-	-	-	10.6706717	2.6067263	4.0257534	0	0.7411051	0.1925969	4.000274	17.5415674	10.2329098	0	35.8616231
065C	-	-	-	-	-	0.8875509	1.40609298	1.33939499	1.75396963	0.20583286	0.04660239	8.22286797	22.91406979	6.19234689	6.06667142	5.56181665
067C	-	-	-	-	-	6.18486499	8.06881496	1.75003566	6.11123565	0.51920068	0.25370858	7.01491088	23.87383803	6.68326859	0.75491734	9.93852349
068C	-	-	-	-	-	4.15497476	4.36412854	9.40535195	3.28440862	0.36937409	0.17453128	6.03212446	18.98056604	6.32903121	3.24576852	18.25350887
069C	-	-	-	-	-	4.38922121	6.61776082	8.61085398	3.46957486	0.69132981	0.2419868	7.83109797	37.47424857	13.8353074	5.1431345	31.24995156
073C	-	-	-	-	-	1.5187613	10.3474125	0.5729872	0.7503404	0.6824215	0.2990444	2.1468888	24.3835037	6.5012749	0.7415129	13.3832273
074C	-	-	-	-	-	0	0.008671391	4.720626369	0	0.231740006	0	8.285993963	23.00665174	6.666211606	9.163568833	7.018483141
075C	-	-	-	-	-	15.3697269	29.836368	16.5673679	8.6781451	2.7899992	0.4323289	7.5496867	21.843353	3.0515915	0	25.0204156
081C	-	-	-	-	-	3.55153046	10.89852448	4.8236241	0.70185007	0.72754794	0.32633864	1.75034461	19.11942996	11.15532627	0.69359301	17.82466693
082C	-	-	-	-	-	7.5910692	5.4038403	11.4556135	1.5001399	1.0782774	0.2790075	10.64357	19.3524182	10.5080125	0	24.9432735
083C	-	-	-	-	-	0.9179852	0	0.6926615	2.7211703	0.4923102	0.120501	2.8583248	20.046821	5.9453835	1.792771	8.8246109
084C	-	-	-	-	-	0	2.3381555	6.8990276	2.2586102	0.3092294	0.2400421	7.4230044	20.8174226	4.2570189	4.4640767	2.0586762
090C	-	-	-	-	-	1.015823	8.4055649	2.2994538	2.0074597	0.5398722	0.173347	4.4436703	16.4157072	5.8731306	1.9838425	10.9881763
091C	-	-	-	-	-	19.0309044	17.4179561	11.1686418	2.0893718	0.7662287	0.5551391	2.9082901	16.3159127	7.4058643	2.0647909	31.8539106
091N	-	-	-	-	-	2.950061825	0.391385953	0.127052448	1.687225468	0.405705865	0.195456583	5.121516065	25.79447826	12.42183865	0.262500615	26.47945337
096C	-	-	-	-	-	6.47879321	0.03591937	0	8.53555296	1.41903133	0.65201203	6.47680509	13.48454342	7.80377079	0	21.78394007
104C	-	-	-	-	-	7.33474131	20.68486137	6.14932857	4.02634609	1.90969602	0.24605095	4.85719118	30.40028622	14.88318741	5.57056824	27.50516356
106C	-	-	-	-	-	0	0.3805244	17.2628368	15.0707306	0.847449	0.8008486	17.7727096	14.618403	9.5390759	11.1700709	27.0756734
106N	-	-	-	-	-	4.75179216	0.8516447	0	1.87808928	0.85863631	1.06037937	4.12099038	19.75452709	13.94792629	5.56798234	47.10260787
107C	-	-	-	-	-	1.21265479	4.15741753	1.37250473	1.19821842	0.24021563	0.05571346	3.27779612	32.22929652	3.89320666	0.59206087	10.73182171
117C	-	-	-	-	-	13.61170968	0.005957786	7.027289354	12.74178839	1.713343686	0.639474013	4.666612794	20.29183399	16.54810964	12.591885	42.91021004
118C	-	-	-	-	-	9.64469499	5.39895878	1.81934019	5.95617325	0.27376415	0.15825354	1.03633302	15.65994145	9.34955635	0	9.82921657
119C	-	-	-	-	-	3.30722188	0.02062768	0	0	0.64714048	0.13023853	7.09149815	16.39720737	3.92995647	0	20.47379996
119N	-	-	-	-	-	3.385710432	0.009385434	1.703114944	2.23026957	0.256275227	0.029628759	5.454279506	21.56028673	11.85792354	1.102015552	9.85394489
123C	-	-	-	-	-	0	1.11748235	0	0	0.34355864	0.09608702	4.33470751	47.49905111	8.19689081	2.04221243	5.07579011
123N	-	-	-	-	-	0	1.57327764	0	1.3644506	0.4003045	0.07250599	5.38117963	26.35828173	12.82016948	4.04519472	7.48819217
124C	-	-	-	-	-	3.36429523	5.92671704	6.7693698	1.10808137	0.41178133	0.3091342	7.7976285	31.31047918	16.42751034	0	20.3274394
125C	-	-	-	-	-	3.7657034	3.4878646	1.4206972	0.9302184	0.3138451	0.1482936	1.02506	16.7363166	3.2186927	0	12.2004702
126C	-	-	-	-	-	3.60312413	0	16.8560698	4.27227576	2.27005839	0.80405	7.99785754	21.51874774	13.50073478	10.55503422	42.35677164
126N	-	-	-	-	-	3.9067147	0	5.8955876	5.7903092	2.3688395	1.589748	4.8507999	38.8021814	9.3389582	1.907396	75.147698
132C	-	-	-	-	-	8.22143495	0	8.75780664	4.77856513	0.77106831	0.49516304	10.56849241	14.82463453	3.18592347	0.94446934	14.27249062
132N	-	-	-	-	-	8.144506414	0.009675926	0	3.448949145	1.641458071	0.565097472	6.956637584	29.6995457	10.28447528	6.816746544	57.5783446
143C	-	-	-	-	-	14.19561765	0.00621336	7.32874232	5.16770292	0.71473672	0.56883145	4.05328689	11.92872215	6.04340877	1.45911612	24.2737424
143N	-	-	-	-	-	0	0	0	3.309232	0.63106374	0.63745763	3.96650869	21.53483851	19.92188694	1.63514993	50.26101625
147C	-	-	-	-	-	5.6583669	0.0156854	19.9243101	7.4546738	3.9367135	0.8170313	6.7014859	19.8316699	8.1262436	27.6261443	38.211696
147N	-	-	-	-	-	8.328579081	0.008657783	2.356609308	3.086035999	1.242390417	0.601296276	2.754349276	22.81898378	20.43026109	7.11603595	69.96119394
149C	-	-	-	-	-	4.25888697	0	8.35516189	13.46619499	0.40330388	0.54786947	2.94505219	20.25440651	11.00949381	1.66347115	28.90079238
152N	-	-	-	-	-	2.68825932	0	0	2.65625623	0.56628866	0.43757022	7.60013291	24.5694642	9.23587231	1.05000246	39.03868384

153C	-	-	-	-	-	10.3400987	0	4.255677	1.8576368	0.7720779	0.3948543	3.5015064	16.0116633	3.0832042	0	17.3508155
153N	-	-	-	-	-	2.7948925	0	0	2.76162	0.6211595	0.1834383	4.9651957	33.4408443	13.0256717	0	21.3384429
156N	-	-	-	-	-	2.94944024	0	0	0	0.14250162	0.01935818	3.95798589	21.98269948	15.98683533	0	15.57459415
158C	-	-	-	-	-	12.5784657	12.5944715	17.0838434	0	1.0574434	0.8255675	7.7850425	14.6499534	8.8108234	0	40.9734719
158N	-	-	-	-	-	3.36765524	1.56554381	0.50820979	1.33102564	0.29287368	0.14145963	3.96274976	23.18490507	11.43897524	0	29.96609258
162C	-	-	-	-	-	3.43721762	0	1.29676847	1.69814918	0.61860513	0.21431647	6.38535358	18.29275025	7.18955189	4.19542739	22.68789374
164C	-	-	-	-	-	11.98662294	0.34889174	13.15556633	11.84392505	0.65283895	0.67229043	11.71927824	12.95589965	6.5728267	1.06405316	31.2397743
171N	-	-	-	-	-	2.328844016	0.011303992	0.851557472	2.68617854	0.203420414	0.044033855	3.966329893	25.43007281	14.09628476	1.482544311	13.31080125
175C	-	-	-	-	-	8.3739947	0.7080062	12.6371192	3.6774686	0.512478	0.1465637	14.6455503	20.4543511	6.5950583	8.1769595	32.6196292
178C	-	-	-	-	-	6.5509892	0	8.6502834	0	0.5697176	0.3224728	15.86146	22.2801938	5.8270018	6.3968482	25.587393
179C	-	-	-	-	-	4.95127167	0.00823517	8.21911098	6.84925915	0.88989734	0.36396506	21.60235843	20.58381915	4.7068586	1.93390847	23.56364198
179N	-	-	-	-	-	11.0694538	0	2.08810151	1.36720932	0.57492987	0.94448368	4.41408801	23.85994771	15.73069107	4.05337353	54.12368662
180C	-	-	-	-	-	15.216511	0	9.3287587	2.8191304	0.5054381	0.574259	12.7350727	15.2456109	7.0846465	4.6432736	24.893356
180N	-	-	-	-	-	5.2240648	0	0	7.7428104	0.5552793	0.7886082	7.0852506	26.0699263	22.5075666	5.1011456	60.4956253
182C	-	-	-	-	-	12.6870418	0	13.0540224	10.2567319	1.5435748	1.4685689	5.3758337	21.2317615	16.5426727	2.2524588	37.7778885
182N	-	-	-	-	-	5.39648436	0	0	0	0.6518252	0.37780227	3.0582148	20.36872222	14.75027009	7.026011	59.90868121
183C	-	-	-	-	-	6.165981793	0.005697516	3.618621638	4.061718165	0.261497194	0.125904912	2.682884035	30.24400784	7.769770314	2.006966623	16.53636577
183N	-	-	-	-	-	1.2719776	0.01322255	0	3.14208751	0.1505656	0.05843895	2.47838028	29.29985889	16.33464597	1.86307307	16.7676576
185C	-	-	-	-	-	8.23556903	1.80353777	2.07137039	8.13752654	0.90190643	0.14414091	13.52945725	15.63115603	5.34144408	2.68059698	21.60092711

MIR4645	HOXA13	TRG.AS1	HOXA10	NEAT1	HOXA3	MIR570	SNHG1	MIR4668	HOXA9	MIR4701	SNHG5	MIR4768	y
7.852192	0	1.35417	0	50.464568	5.441007	42.073987	11.710867	0	0.19961	0	12.713441	20.42631	tumor
0	0.14499851	0.89281168	4.32689728	64.31377382	4.32441402	20.90744051	15.2165441	2.63379445	9.92129241	2.92643828	25.27273776	0	tumor
0	0	1.394679	2.898479	32.087063	3.562843	18.842269	10.21877	2.373636	5.924296	1.758249	5.292683	14.968878	tumor
0	0.03722556	0.38966061	0.89378508	41.56727221	1.86866969	5.85554276	7.66188521	1.35235154	0.71881346	0	45.70599722	23.02652628	tumor
0	0.03590378	2.12230443	0.09851989	154.5182218	1.5266693	30.12067806	19.05798494	2.60866587	0.48228884	0	30.3255073	7.40297071	tumor
0	0.07363864	0.84638674	0.26941903	209.7754302	9.13266227	48.90719234	28.11937404	0	1.77227068	0	12.24292053	26.99287429	tumor
0	0.27054453	0.35538066	1.163052	16.4068732	3.28073049	6.61988074	5.5684346	0	2.66506164	0	54.50113493	19.83408284	tumor
0	0.03671084	4.52085576	3.24868671	16.20510448	1.62602772	7.69943682	14.01826377	0	4.2840668	0	59.34132876	0	tumor
2.99629633	0.06048363	0.52138942	3.46456025	35.34310092	3.96490862	11.89251637	10.89281886	1.09864199	4.72245582	2.44142664	40.65447215	14.54958309	tumor
1.15315257	0.06983307	1.33296619	1.77250158	38.18162267	4.45407189	9.15389155	11.7728404	2.53693566	2.6969075	0	24.05730504	0	tumor
2.2245104	0	0.8087415	5.1289156	47.2776116	3.659645	16.7755602	8.9383148	0	5.1459703	1.359423	23.3573589	3.4720398	tumor
0.8945898	0.2708746	3.1022554	1.133501	18.722298	0.7358655	6.3912445	4.4565147	0.9840488	1.6828566	0	25.2950543	0	tumor
0	0.09500401	0.35098577	5.01829802	27.50537053	2.24426572	2.49067221	8.29759889	0	4.62612145	0	45.30990957	0	tumor
0	0	0.708914427	0.552865284	51.4075056	8.941195563	21.1285733	14.88852417	4.182595122	0.193319022	0	54.22852838	0	tumor
6.37656038	2.07557918	0.35665507	3.97352427	22.16993952	4.96012285	7.59270849	24.31445626	3.50710821	12.3194557	0	22.05436335	13.27013916	tumor
1.567481	0.5695448	1.1689689	5.4047862	14.3242719	3.5597764	3.7328672	8.6119645	0	6.3754924	1.9158102	32.225929	0	tumor
5.6194672	0.2552294	0.785773	4.7273559	20.3644289	9.3453332	8.9216284	12.1538184	3.090707	11.4281671	0	24.6238858	5.8472834	tumor
3.90958202	0.0473516	0.6122803	4.011672	7.45751869	2.72653851	4.96557222	3.17388113	0	4.88975257	0	14.91182309	0	tumor
0	0.2176725	0.8309818	2.6878176	11.3445898	2.4681814	3.4239655	5.6592061	0	5.2996672	0	11.0153489	0	tumor
0	0.11587373	0.24971771	3.81548721	2.89593351	2.30956985	3.03780307	3.35383649	0	3.64807153	0	34.01219558	0	tumor
1.66670063	0.08831613	0.63701863	2.40608266	2.76491337	3.50196794	1.32305102	5.43275182	0	2.24556133	2.03707855	32.95670835	0	tumor
1.79149561	0.05424505	0.36740842	3.31187467	14.89907117	3.31568144	11.37692057	4.38883037	0	3.09682107	0	26.36460862	0.93206191	tumor
1.89249538	0.02865162	0.5821829	4.02927429	14.21570178	2.82577776	8.26259581	5.43160282	1.04087246	4.54629998	0	27.2511884	0	tumor
0.8185532	0	2.6249196	0.9521449	11.657812	0.5415834	5.1982345	4.0441669	0	0.5202096	0	6.9480339	0	tumor
2.247917318	0.15314655	0.230506776	3.035021899	2.980240538	1.386807328	9.814360663	2.465467357	0	2.342910193	0	39.6792807	0	tumor
2.3667668	0.0716638	0.3971355	4.0803903	28.4964259	3.0895486	18.7877368	6.8413173	0	5.8360454	0	18.9154988	0	tumor
0	0.06955022	0.5995465	2.59232024	22.51062248	2.23170718	8.50902763	4.36357902	0	2.04368359	0.93580009	26.01718513	0	tumor
0	0.0991048	0.9763622	5.9487607	14.6064481	4.7408043	24.6827136	8.0854184	0	12.6885385	0	39.7338405	1.7028615	tumor
0	0.4194647	0.7194959	4.3779498	9.5680207	2.1764307	2.3564774	3.1036991	0	3.4713114	1.209409	7.82653	0	tumor
0	0.7460608	0.2296892	2.2007284	6.1999431	2.9079738	0	10.7085546	0	2.1296107	0	69.8051222	0	tumor
0	0	0.5920305	6.8233051	14.428104	2.2517488	3.4768374	3.5467259	0	8.183596	1.3383065	27.3701394	0	tumor
0	0.2760633	0.7649225	5.9654467	28.9467055	3.5460123	12.6654701	6.9623475	0	9.3287104	0	36.6844029	0	tumor
0.144886703	0.037815478	0.104875648	2.565561678	16.64023356	2.003103748	4.323002243	2.813306963	0	6.571072478	0.705768033	15.80036012	1.131525938	normal
4.65575616	0.21145884	0.69441787	4.40017311	32.01985205	11.57235443	31.41435471	9.68771626	0	1.53859753	2.84518432	35.09230381	0	tumor
0.87847551	0.09309837	1.26113349	3.26626058	15.82695157	3.11036638	5.57877231	5.54683845	1.93264612	3.61772978	1.07369229	13.33968521	0	tumor
0	0	0.3065233	0.6830005	22.6309191	1.5434724	6.525471	14.4086777	0	1.0448509	0	53.4649539	0	tumor
0	0.03101838	0.11459528	1.34055145	42.70531014	1.86849384	10.57151286	3.94818769	0	5.70309185	1.25205952	12.76078987	2.13188513	normal
0.65357368	0.18800202	0.87733959	6.92361439	6.49207339	3.44773019	2.07526489	2.6797217	0	9.81914084	0	21.30804594	0.68006991	tumor
4.633377596	0.303972406	1.454147601	1.090745666	21.72452441	2.306107854	17.16419604	7.994714582	0	2.061233513	1.887672354	22.32473208	0.803536205	tumor
0	0.01967434	0.31497052	1.61959068	7.65190495	1.13867204	11.34743109	10.09695056	0	1.65176148	0	58.19739788	0	tumor
0	0.08095736	0.61479893	0.81453833	11.47515348	2.05587695	1.41494544	4.12918451	0	0.67967832	0	24.19308561	0	tumor
0	0	0.18144566	2.147845256	10.08426275	2.240628463	0	2.34427696	0	9.030051505	0	16.72941648	1.265828675	normal
0	0.03413057	0.18913953	8.78008109	18.71826233	0.82641783	4.47391898	7.60256531	0	12.5506056	0	7.67291517	0	tumor
0	0.09014066	0	2.84447646	7.86343543	2.60848986	0	3.11251671	0	6.62179724	0	17.92636101	0	normal
0	0.03660198	0.18029798	8.63747481	17.70243573	4.77715338	8.63618054	3.96501958	0	8.02032877	0	12.7258822	0	tumor
2.0295674	1.3827076	1.2297802	1.6230517	7.9727401	0.9799049	4.8332997	3.2037553	1.1162621	2.2443169	0	5.4534636	0	tumor
1.55355482	0.04704039	1.18754784	2.96881099	27.40621644	2.6391671	6.1661712	10.5896931	3.41782061	5.1933045	1.89878923	49.34371463	0	tumor
0	0.1275098	0.353307	3.498865	58.280008	3.5769212	21.7285831	5.2229946	0	10.4374673	0	20.4836852	0	normal
1.04259603	0.09470691	0.38876462	0.24904742	8.22827484	2.74994993	7.86246387	6.90372892	1.14685563	0.50357147	0	36.9505884	0	tumor
0	0.037975059	0	3.178206171	48.21954182	5.068497489	15.92916718	6.427753997	0	7.874853841	6.131465146	16.86701543	0	normal
3.22142519	0.73156628	0.72072564	3.12822372	43.25916864	0.9072883	3.83582072	7.13242064	0.88589193	4.68829568	0	55.13202881	0	tumor
0	0	0.06730641	2.88698578	61.9269853	1.83982847	5.73145336	4.36649462	0	7.0663957	2.20615467	19.1021094	0	normal
0	0	1.7057305	3.2095116	31.8638438	2.2540607	0	11.5035382	0	4.6514802	2.4848913	57.3748413	0	tumor
1.122194909	0.016989581	0.115072529	2.109526631	64.27825181	2.217416484	7.126516121	4.290543127	0	5.505757193	2.057357333	18.1539326	2.335378594	normal
3.67259863	0.05560167	1.74604054	4.80598219	14.84315734	5.91061508	0.72884045	10.69121231	0	8.21575713	0	28.92171423	0.95537194	tumor
0.57954681	0.03509643	0.11885621	2.61225997	22.1684866	2.10378455	4.60052626	2.54253792	0	6.93906382	0.708335	18.09600703	3.01520978	normal

1.0132564	0	0.3211508	3.7463427	14.3862207	2.3917181	11.2607261	7.0002638	0	6.5425121	2.476849	31.6383335	0	tumor
0	0	0.1872285	3.4209223	18.1298707	3.070747	7.9716865	3.952734	0	6.6118415	1.2273867	15.858064	0	normal
0	0.07219924	0.05927446	1.46934955	3.05372712	0.83856299	1.26187392	1.31982739	0	4.88960521	0	19.82347921	0	normal
0	0	0.5055751	0.4506124	31.9068829	3.4428657	8.6104136	21.5139987	2.9828933	0.5514758	3.3143259	20.4019652	0	tumor
0	0.04396624	0.05414342	2.75971489	23.20884983	1.99932045	3.45792229	3.43812854	0	7.84377938	0.88735043	9.42389022	1.51089397	normal
1.85252638	0.05609301	0.69077255	2.27030681	12.05151121	2.2857577	13.97034068	4.74722215	1.01888951	3.57906384	0	24.35269913	0	tumor
4.69841655	0.03556607	0.41608858	3.83053397	21.96061398	0.92418923	8.39175945	22.70744967	0	8.39052813	0	42.40598399	0	tumor
0	0.010378895	0.212145875	2.704383793	8.588396755	1.972311767	1.3604915	2.704031625	0	8.558142388	0	17.55615765	0.632914338	normal
1.002946	0	0.8040567	3.2915675	12.9644214	1.9369481	1.592306	8.7795156	0	5.2011473	0	50.5816452	0	tumor
1.765364	0.267269	0.1974814	0.6600471	18.9626779	2.1466432	15.4150853	10.640125	0	0.9872983	2.1576671	44.1758006	0	tumor
2.13483402	0.06464106	0.61693085	4.83346694	13.33931351	4.90550748	9.32064132	16.49949328	0	4.66717428	0	40.03654274	0	tumor
0	0.04516146	0.11123059	2.26159363	54.70638863	2.66710597	11.83975085	3.69976453	0	6.82476229	0	9.08406367	0	normal
4.1005533	0	0.6498334	0.1703494	20.2097732	2.2547805	8.1376959	6.5149232	0	0.2345397	0	55.1758503	0	tumor
0	0	0	1.1111883	59.4489489	2.0139219	4.4700761	4.0981648	0	6.5132654	0	19.0882168	0	normal
13.6756425	0	0.7649088	5.6554518	29.4626692	4.7798054	10.8559224	15.1393295	0	14.632802	0	33.5087504	0	tumor
1.93899654	0.05871126	0	1.6513116	26.05436648	1.24823465	4.61761032	4.29304985	2.1328962	5.76705839	0	13.30579124	6.05281353	normal
0.738494212	0.279512654	1.748604091	3.144626688	10.7149292	2.443067823	4.103591548	7.539483239	0	2.853527981	0	24.91109609	0.768433166	tumor
0	0.02075779	0.24284609	3.26092233	7.09253076	1.70399507	2.720983	3.06378629	0	8.08623327	0	18.38289882	0	normal
0	0.08959919	0.49652704	5.90063969	18.57473446	3.28071205	9.39590694	8.79615489	3.25501062	6.16829348	0	38.41005728	0	tumor
