# Supplementary material for: The Diagnostic and Prognostic Values of HOXA Gene Family in Kidney Clear Cell Renal Cell Carcinoma
Source: J Oncol. 2022 Mar 16;2022:1762637. doi: 10.1155/2022/1762637 (PMC8942704; doi:10.1155/2022/1762637)
Supplement: Supplementary Materials — Supplementary Figure 1. Flow chart of data collection and subsequent model establishment in our study; Supplementary Figure 2. The TNM stage and clinical grade measured by the time-dependent receiver-operating characteristic curves at 1, 3, and 5 years. Supplementary Figure 3. The Kaplan–Meier survival curves of evaluating single prognostic roles of the prognosis-related HOXA genes. Supplementary Figure 4. A more detailed machine learning model construction process for early screening classification. Supplementary Figure 5. Custom scripts for prognostic model and machine learning model construction in this study; Table S1: differentially expressed genes between KIRC samples and paired normal samples, including the differential expression HOXA genes; Table S2: the training set, the test set, and the external validation set for machine learning model; Table S3: small molecule drugs by querying the CMAP and DGIdb databases; and Table S4: a detailed summary of the different roles of HOXA family members in KIRC. [file 1762637.f1.zip › 1762637.f1/Supplementary Table S3 (2).pdf]

###DGIdb

match_term	gene	drug	interaction_types	sources
UMOD	UMOD	CYCLOSPORINE		PharmGKB
SLC12A1	SLC12A1	ETHACRYNATE SODIUM	inhibitor	ChemblInteractions
SLC12A1	SLC12A1	TORSEMIDE	inhibitor	TdgClinicalTrial|ChemblInteractions|TEND|TTD
SLC12A1	SLC12A1	HYDROFLUMETHIAZIDE	inhibitor	TdgClinicalTrial|TEND|TTD
SLC12A1	SLC12A1	BUMETANIDE	inhibitor	TdgClinicalTrial|ChemblInteractions|TEND|GuideToPharmacology|TTD
SLC12A1	SLC12A1	CHLORTHALIDONE	inhibitor	TdgClinicalTrial|TEND|TTD
SLC12A1	SLC12A1	FUROSEMIDE	inhibitor	TdgClinicalTrial|ChemblInteractions|TEND|GuideToPharmacology|TTD
SLC12A1	SLC12A1	METHYCLOTHIAZIDE	inhibitor	TdgClinicalTrial|TEND|TTD
SLC12A1	SLC12A1	TRICHLORMETHIAZIDE	inhibitor	TdgClinicalTrial|TEND|TTD
SLC12A1	SLC12A1	ALDOSTERONE		TTD
SLC12A1	SLC12A1	METOLAZONE		TdgClinicalTrial|TEND
SLC12A1	SLC12A1	BENDROFLUMETHIAZIDE		TTD
SLC12A1	SLC12A1	CHLORMERODRIN	inducer	TdgClinicalTrial|TEND
SLC12A1	SLC12A1	PIRETANIDE	inhibitor	GuideToPharmacology
SLC12A1	SLC12A1	ETHACRYNIC ACID	inhibitor	ChemblInteractions
KCNJ10	KCNJ10	CHLORPROPAMIDE		TTD
KCNJ10	KCNJ10	NORTRIPTYLINE	blocker	GuideToPharmacology
KCNJ10	KCNJ10	TOLAZAMIDE		TTD
KCNJ10	KCNJ10	MITIGLINIDE		TTD
KCNJ10	KCNJ10	GLISOXEPIDE		TTD
KCNJ10	KCNJ10	GLIQUIDONE		TTD
KCNJ10	KCNJ10	GLYMIDINE		TTD
KCNJ10	KCNJ10	GLIPIZIDE		TTD
KNG1	KNG1	AVORALSTAT		TTD
PVT1	PVT1	GLIVEC		NCI
ESRRB	ESRRB	DAIDZEIN	agonist	GuideToPharmacology
ESRRB	ESRRB	GENISTEIN	agonist	GuideToPharmacology
ESRRB	ESRRB	DIETHYLSTILBESTROL	antagonist	DTC|GuideToPharmacology
CA9	CA9	GIRENTUXIMAB		ChemblInteractions|TTD
CA9	CA9	ETHOXZOLAMIDE	inhibitor	ChemblInteractions
CA9	CA9	CG250 177LU		ChemblInteractions
CA9	CA9	CURCUMIN		TTD
CA9	CA9	PHENOL		TTD
CA9	CA9	INDISULAM		TTD
CA9	CA9	CELECOXIB		PharmGKB
CA9	CA9	SUNITINIB		CGI
CA9	CA9	PARABEN		TTD
CA9	CA9	CG250 111IN		ChemblInteractions
CA9	CA9	SULFAMIDE		TTD
CA9	CA9	IODINE I 124 GIRENTUXIMAB		ChemblInteractions
KCNJ1	KCNJ1	GLICLAZIDE		TdgClinicalTrial|TEND
KCNJ1	KCNJ1	GLYMIDINE	other/unknown	TdgClinicalTrial|TEND
KCNJ1	KCNJ1	NATEGLINIDE		TEND
KCNJ1	KCNJ1	GLIMEPIRIDE	inhibitor	TdgClinicalTrial|TEND
KCNJ1	KCNJ1	GLYBURIDE		TdgClinicalTrial|TEND
KCNJ1	KCNJ1	ACETOHEXAMIDE	inhibitor	TdgClinicalTrial|TEND
KCNJ1	KCNJ1	CHLORPROPAMIDE		TdgClinicalTrial|TEND
KCNJ1	KCNJ1	MINOXIDIL	inducer	TdgClinicalTrial|TEND
KCNJ1	KCNJ1	REPAGLINIDE		TEND
KCNJ1	KCNJ1	TOLAZAMIDE		TdgClinicalTrial|TEND
KCNJ1	KCNJ1	GLIPIZIDE		TdgClinicalTrial|TEND
KCNJ1	KCNJ1	TOLBUTAMIDE	inhibitor	TdgClinicalTrial|TEND
KCNJ1	KCNJ1	HYDROCHLOROTHIAZIDE		PharmGKB
KCNJ1	KCNJ1	MK-7145	blocker	GuideToPharmacology
SERPINA5	SERPINA5	GEMCITABINE		PharmGKB
SERPINA5	SERPINA5	THROMBIN		NCI
SERPINA5	SERPINA5	HEPARIN		NCI
SERPINA5	SERPINA5	CARBOPLATIN		PharmGKB
EGF	EGF	CETUXIMAB		CIViC|PharmGKB

EGF	EGF	NIMOTUZUMAB		TdgClinicalTrial
EGF	EGF	PANITUMUMAB		PharmGKB
CASR	CASR	CINACALCET HYDROCHLORIDE	positive modulator	ChemblInteractions
CASR	CASR	TECALCET HYDROCHLORIDE	modulator	ChemblInteractions
CASR	CASR	VELCALCETIDE	agonist	ChemblInteractions|GuideToPharmacology|TTD
CASR	CASR	CINACALCET	allosteric modulator|agonist	TdgClinicalTrial|TEND|GuideToPharmacology|TTD
CASR	CASR	RONACALERET	allosteric modulator|antagonist	TdgClinicalTrial|ChemblInteractions|GuideToPharmacology
CASR	CASR	TECALCET	allosteric modulator	GuideToPharmacology
CASR	CASR	ASP7991	positive modulator	ChemblInteractions|TTD
CASR	CASR	CALINDOL	allosteric modulator	GuideToPharmacology
CASR	CASR	ENCALERET	allosteric modulator	GuideToPharmacology
CASR	CASR	CHEMBL1084513	allosteric modulator	GuideToPharmacology
CASR	CASR	ENCALERET SULFATE		TTD
CASR	CASR	PAMIDRONIC ACID		NCI
CASR	CASR	EVOCALCET	agonist	GuideToPharmacology
CASR	CASR	TRYPTOPHAN	allosteric modulator	GuideToPharmacology
CASR	CASR	CHEMBL180672		TdgClinicalTrial
CASR	CASR	SPERMINE	agonist	GuideToPharmacology
SCNN1G	SCNN1G	TRIAMTERENE	inhibitor|blocker	TdgClinicalTrial|ChemblInteractions|TEND|PharmGKB
SCNN1G	SCNN1G	AMILORIDE	blocker|inhibitor	TdgClinicalTrial|TEND|PharmGKB
SCNN1G	SCNN1G	AMILORIDE HYDROCHLORIDE	blocker	ChemblInteractions
SCNN1G	SCNN1G	P-1037	blocker	ChemblInteractions
PTGER1	PTGER1	CLOPROSTENOL	agonist	GuideToPharmacology
PTGER1	PTGER1	DINOPROSTONE	agonist	DTC|TdgClinicalTrial|ChemblInteractions|TEND|GuideToPharmacology
PTGER1	PTGER1	EPOPROSTENOL	agonist	GuideToPharmacology
PTGER1	PTGER1	CICAPROST	agonist	GuideToPharmacology
PTGER1	PTGER1	ECRAPROST		TTD
PTGER1	PTGER1	ALPROSTADIL	agonist	TdgClinicalTrial|ChemblInteractions|TEND|GuideToPharmacology
PTGER1	PTGER1	CAFFEINE		NCI
PTGER1	PTGER1	ILOPROST	agonist	TdgClinicalTrial|TEND|GuideToPharmacology
PTGER1	PTGER1	CHEMBL234940	antagonist	GuideToPharmacology
PTGER1	PTGER1	IBUPROFEN		NCI
PTGER1	PTGER1	SULPROSTONE	agonist	GuideToPharmacology
PTGER1	PTGER1	CARBACYCLIN	agonist	GuideToPharmacology
PTGER1	PTGER1	INTERLEUKIN-10		NCI
PTGER1	PTGER1	BIMATOPROST	agonist	TdgClinicalTrial
PTGER1	PTGER1	CHEMBL112816	antagonist	GuideToPharmacology
PTGER1	PTGER1	TREPROSTINIL	agonist	GuideToPharmacology
PTGER1	PTGER1	BUTAPROST		TTD
PTGER1	PTGER1	ENPROSTIL	agonist	GuideToPharmacology
GABRD	GABRD	CLORAZEPIC ACID		TdgClinicalTrial|TEND
GABRD	GABRD	DIAZEPAM	potentiator|positive modulator	TdgClinicalTrial|ChemblInteractions|TEND
GABRD	GABRD	TRIAZOLAM	positive modulator|potentiator	TdgClinicalTrial|ChemblInteractions|TEND
GABRD	GABRD	ENFLURANE		ChemblInteractions
GABRD	GABRD	HALAZEPAM	positive modulator|potentiator	TdgClinicalTrial|ChemblInteractions|TEND
GABRD	GABRD	BROMAZEPAM	potentiator	TdgClinicalTrial|TEND
GABRD	GABRD	QUAZEPAM	positive modulator|potentiator	TdgClinicalTrial|ChemblInteractions|TEND
GABRD	GABRD	PRAZEPAM	positive modulator|potentiator	TdgClinicalTrial|ChemblInteractions|TEND
GABRD	GABRD	CLOTIAZEPAM	potentiator	TdgClinicalTrial|TEND
GABRD	GABRD	OXAZEPAM	positive modulator|potentiator	TdgClinicalTrial|ChemblInteractions|TEND
GABRD	GABRD	DESFLURANE	positive modulator	ChemblInteractions
GABRD	GABRD	ESZOPICLONE	positive modulator	ChemblInteractions
GABRD	GABRD	PENTOBARBITAL	positive modulator	ChemblInteractions
GABRD	GABRD	FLUDIAZEPAM	potentiator	TdgClinicalTrial|TEND
GABRD	GABRD	ALPRAZOLAM	positive modulator	TdgClinicalTrial|ChemblInteractions|TEND
GABRD	GABRD	MIDAZOLAM HYDROCHLORIDE	positive modulator	ChemblInteractions
GABRD	GABRD	CLONAZEPAM	positive modulator	TdgClinicalTrial|ChemblInteractions|TEND
GABRD	GABRD	CHLORDIAZEPOXIDE	positive modulator	TdgClinicalTrial|ChemblInteractions|TEND
GABRD	GABRD	FLURAZEPAM	potentiator	TdgClinicalTrial|TEND
GABRD	GABRD	THIAMYLAL SODIUM	positive modulator	ChemblInteractions
GABRD	GABRD	TEMAZEPAM	potentiator|positive modulator	TdgClinicalTrial|ChemblInteractions|TEND

GABRD	GABRD	CLOBAZAM	positive modulator	TdgClinicalTrial|ChemblInteractions|TEND
GABRD	GABRD	NITRAZEPAM	potentiator	TdgClinicalTrial|TEND
GABRD	GABRD	ESTAZOLAM	potentiator|positive modulator	TdgClinicalTrial|ChemblInteractions|TEND
GABRD	GABRD	TALBUTAL	positive modulator	ChemblInteractions
GABRD	GABRD	TOPIRAMATE		ChemblInteractions
GABRD	GABRD	ADIPIPLON		TdgClinicalTrial
GABRD	GABRD	GLUTETHIMIDE		ChemblInteractions
GABRD	GABRD	BUTABARBITAL SODIUM	positive modulator	ChemblInteractions
GABRD	GABRD	ISOFLURANE	positive modulator	ChemblInteractions
GABRD	GABRD	METHARBITAL	positive modulator	ChemblInteractions
GABRD	GABRD	ADINAZOLAM	potentiator	TdgClinicalTrial|TEND
GABRD	GABRD	CINOLAZEPAM	potentiator	TdgClinicalTrial|TEND
GABRD	GABRD	GANAXOLONE		TTD
GABRD	GABRD	BUTALBITAL	positive modulator	ChemblInteractions
GABRD	GABRD	FLUMAZENIL		ChemblInteractions
GABRD	GABRD	THIOPENTAL SODIUM	positive modulator	ChemblInteractions
GABRD	GABRD	LORAZEPAM	positive modulator	TdgClinicalTrial|ChemblInteractions|TEND
GABRD	GABRD	CLORAZEPATE DIPOTASSIUM	positive modulator	ChemblInteractions
GABRD	GABRD	ETHCHLORVYNOL		ChemblInteractions
GABRD	GABRD	PICROTOXIN	blocker	GuideToPharmacology
GABRD	GABRD	PF-06372865	agonist	ChemblInteractions
GABRD	GABRD	GABOXADOL	agonist	GuideToPharmacology|TTD
GABRD	GABRD	BREXANOLONE		TTD
GABRD	GABRD	AZIETOMIDATE		DTC
GABRD	GABRD	OCINAPLON		TdgClinicalTrial
GABRD	GABRD	SEVOFLURANE	positive modulator	ChemblInteractions
GABRD	GABRD	CHLORDIAZEPOXIDE HYDROCHLORIDE	positive modulator	ChemblInteractions
GABRD	GABRD	TRICLOFOS SODIUM		ChemblInteractions
GABRD	GABRD	ETOMIDATE		DTC|ChemblInteractions
GABRD	GABRD	METHYPRYLON	positive modulator	ChemblInteractions
GABRD	GABRD	HALOTHANE	positive modulator	ChemblInteractions
GABRD	GABRD	PRIMIDONE	positive modulator	ChemblInteractions
GABRD	GABRD	MIDAZOLAM	potentiator	TdgClinicalTrial|TEND
GABRD	GABRD	REMIMAZOLAM		TdgClinicalTrial
GABRD	GABRD	METHOXYFLURANE		ChemblInteractions
GABRD	GABRD	SECOBARBITAL SODIUM	positive modulator	ChemblInteractions
GABRD	GABRD	ACAMPROSATE CALCIUM		ChemblInteractions
GABRD	GABRD	METHOHEXITAL SODIUM	positive modulator	ChemblInteractions
GABRD	GABRD	PROPOFOL	positive modulator	ChemblInteractions
GABRD	GABRD	MEPROBAMATE	agonist	ChemblInteractions
GABRD	GABRD	PENTOBARBITAL SODIUM	positive modulator	ChemblInteractions
SCNN1B	SCNN1B	TRIAMTERENE	blocker|inhibitor	TdgClinicalTrial|ChemblInteractions|TEND|PharmGKB
SCNN1B	SCNN1B	AMILORIDE	blocker|inhibitor	TdgClinicalTrial|TEND|PharmGKB
SCNN1B	SCNN1B	AMILORIDE HYDROCHLORIDE	blocker	ChemblInteractions
SCNN1B	SCNN1B	FARGLITAZAR		PharmGKB
SCNN1B	SCNN1B	P-1037	blocker	ChemblInteractions
SCNN1B	SCNN1B	GLYBURIDE		PharmGKB
CA10	CA10	MONTELUKAST		PharmGKB
ATP1A1	ATP1A1	ISTAROXIME		TdgClinicalTrial
ATP1A1	ATP1A1	DIGOXIN	inhibitor	TdgClinicalTrial|ChemblInteractions|TEND|GuideToPharmacology
ATP1A1	ATP1A1	ACETYLDIGITOXIN	inhibitor	TdgClinicalTrial|ChemblInteractions|TEND|GuideToPharmacology|TTD
ATP1A1	ATP1A1	ARTEMETHER		TTD
ATP1A1	ATP1A1	ALMITRINE	binder	TdgClinicalTrial|TEND|TTD
ATP1A1	ATP1A1	DESLANOSIDE	inhibitor	TdgClinicalTrial|ChemblInteractions|TEND|GuideToPharmacology|TTD
ATP1A1	ATP1A1	BEPRIDIL	inhibitor	TdgClinicalTrial|TEND
ATP1A1	ATP1A1	LUMEFANTRINE		TTD
ATP1A1	ATP1A1	DIGITOXIN	inhibitor	TdgClinicalTrial|ChemblInteractions|TEND|GuideToPharmacology
ATP1A1	ATP1A1	OUABAIN	inhibitor	TdgClinicalTrial|TEND|TTD
ATP1A1	ATP1A1	EPLERENONE		PharmGKB
ATP1A1	ATP1A1	CHLOROPROCAINE		TTD
ADH1C	ADH1C	FOMEPIZOLE	inhibitor	TdgClinicalTrial|ChemblInteractions|TEND

ADH1C	ADH1C	CYCLOPHOSPHAMIDE		PharmGKB
ADH1C	ADH1C	CISPLATIN		PharmGKB
ADH1C	ADH1C	ABACAVIR		PharmGKB
ADH1C	ADH1C	ALCOHOL		PharmGKB
GPC3	GPC3	ERY-974		TTD
GPC3	GPC3	CODRITUZUMAB	antibody	GuideToPharmacology|TTD
TYRP1	TYRP1	FLANVOTUMAB		ChemblInteractions|TTD
CDKN2A	CDKN2A	RIGOSERTIB		JAX-CKB
CDKN2A	CDKN2A	ABEMACICLIB		ClearityFoundationBiomarkers|JAX-CKB|OncoKB
CDKN2A	CDKN2A	ALVOCIDIB		JAX-CKB
CDKN2A	CDKN2A	PALBOCICLIB		JAX-CKB|CIViC|OncoKB
CDKN2A	CDKN2A	SAPANISERTIB		JAX-CKB
CDKN2A	CDKN2A	MILCICLIB MALEATE		JAX-CKB
CDKN2A	CDKN2A	HMN-214		JAX-CKB
CDKN2A	CDKN2A	ALECTINIB		JAX-CKB
CDKN2A	CDKN2A	TRAMETINIB		JAX-CKB
CDKN2A	CDKN2A	CARBOPLATIN		JAX-CKB
CDKN2A	CDKN2A	CRIZOTINIB		JAX-CKB
CDKN2A	CDKN2A	LETROZOLE		CIViC
CDKN2A	CDKN2A	RIBOCICLIB		ClearityFoundationBiomarkers|OncoKB
CDKN2A	CDKN2A	PACLITAXEL		JAX-CKB
CDKN2A	CDKN2A	PF-00477736		JAX-CKB
CDKN2A	CDKN2A	IMATINIB		JAX-CKB
CDKN2A	CDKN2A	PANITUMUMAB		CIViC
CDKN2A	CDKN2A	GSK-461364		JAX-CKB
CDKN2A	CDKN2A	ILORASERTIB		CGI
CDKN2A	CDKN2A	GEMCITABINE		JAX-CKB
CDKN2A	CDKN2A	DABRAFENIB		CIViC
CDKN2A	CDKN2A	VEMURAFENIB		CIViC
NDUFA4L2	NDUFA4L2	NV-128	inhibitor	ChemblInteractions
NDUFA4L2	NDUFA4L2	METFORMIN HYDROCHLORIDE	inhibitor	ChemblInteractions
NDUFA4L2	NDUFA4L2	ME-344	inhibitor	ChemblInteractions
PIK3C2G	PIK3C2G	APITOLISIB	inhibitor	MyCancerGenome
PIK3C2G	PIK3C2G	INFIGRATINIB	inhibitor	MyCancerGenome
PIK3C2G	PIK3C2G	PILARALISIB	inhibitor	MyCancerGenome
PIK3C2G	PIK3C2G	PF-04691502	inhibitor	MyCancerGenome
PIK3C2G	PIK3C2G	DACTOLISIB	inhibitor	MyCancerGenome
PIK3C2G	PIK3C2G	SONOLISIB	inhibitor	MyCancerGenome
PIK3C2G	PIK3C2G	PI-103	inhibitor	MyCancerGenome
PIK3C2G	PIK3C2G	GSK-2636771	inhibitor	MyCancerGenome
PIK3C2G	PIK3C2G	BUPARLISIB	inhibitor	MyCancerGenome
PIK3C2G	PIK3C2G	QUERCETIN	inhibitor	MyCancerGenome
PIK3C2G	PIK3C2G	COPANLISIB	inhibitor	MyCancerGenome
PIK3C2G	PIK3C2G	VOXTALISIB	inhibitor	MyCancerGenome
PIK3C2G	PIK3C2G	SAR-260301	inhibitor	GuideToPharmacology
PIK3C2G	PIK3C2G	ALPELISIB	inhibitor	MyCancerGenome
PIK3C2G	PIK3C2G	GEDATOLISIB	inhibitor	MyCancerGenome
PIK3C2G	PIK3C2G	PICTILISIB	inhibitor	MyCancerGenome
TMPRSS2	TMPRSS2	NAFAMOSTAT	inhibitor	GuideToPharmacology
TMPRSS2	TMPRSS2	CAMOSTAT	inhibitor	GuideToPharmacology
SLC4A1	SLC4A1	METOPROLOL		PharmGKB
SLC4A1	SLC4A1	ATENOLOL		PharmGKB
GATA3	GATA3	MERCAPTOPURINE		PharmGKB
GATA3	GATA3	PEGASPARGASE		PharmGKB
GATA3	GATA3	LEUCOVORIN		PharmGKB
GATA3	GATA3	VINCRISTINE		PharmGKB
GATA3	GATA3	THIOGUANINE		PharmGKB
GATA3	GATA3	ASPARAGINASE		PharmGKB
GATA3	GATA3	PREDNISONE		PharmGKB
GATA3	GATA3	METHOTREXATE		PharmGKB
GATA3	GATA3	DEXAMETHASONE		PharmGKB

GATA3	GATA3	DOXORUBICIN		PharmGKB
GATA3	GATA3	CYCLOPHOSPHAMIDE		PharmGKB
GATA3	GATA3	DAUNORUBICIN		PharmGKB
GATA3	GATA3	CYTARABINE		PharmGKB
SOST	SOST	BLOSOZUMAB	inhibitor	ChemblInteractions
SOST	SOST	SETRUSUMAB		TTD
SOST	SOST	ROMOSOZUMAB	inhibitor	ChemblInteractions|TTD
TRPV6	TRPV6	SOR-C13		TTD
CXCR4	CXCR4	BKT140	antagonist	TdgClinicalTrial|ChemblInteractions|GuideToPharmacology|TTD
CXCR4	CXCR4	PLERIXAFOR	partial agonist|antagonist	DTC|TdgClinicalTrial|ChemblInteractions|NCI|TEND|GuideToPharmacology|TTD
CXCR4	CXCR4	BURIXAFOR	antagonist	TdgClinicalTrial|ChemblInteractions|TTD
CXCR4	CXCR4	CHEMBL2347630		DTC
CXCR4	CXCR4	MSX-122	antagonist	TdgClinicalTrial|ChemblInteractions|TTD
CXCR4	CXCR4	BEVACIZUMAB		PharmGKB
CXCR4	CXCR4	MAVORIXAFOR	antagonist	DTC|TdgClinicalTrial|GuideToPharmacology|TTD
CXCR4	CXCR4	POL6326	antagonist	ChemblInteractions|TTD
CXCR4	CXCR4	ULOCUPLUMAB	antibody	GuideToPharmacology|TTD
CXCR4	CXCR4	CTCE-9908	antagonist	ChemblInteractions|TTD
CXCR4	CXCR4	CHEMBL2347632		DTC
CXCR4	CXCR4	CHEMBL2347631		DTC
CXCR4	CXCR4	CHEMBL1242210		DTC
CXCR4	CXCR4	CHEMBL2347633		DTC
CXCR4	CXCR4	CHEMBL1221944		DTC
CXCR4	CXCR4	USL-311		TTD
CXCR4	CXCR4	PF-06747143		TTD
CXCR4	CXCR4	BALIXAFORTIDE		TTD
CXCR4	CXCR4	CHEMBL2062277		DTC
CXCR4	CXCR4	CISPLATIN		CIViC
TNNC1	TNNC1	LEVOSIMENDAN	potentiator	TdgClinicalTrial|TEND|TTD
TNNC1	TNNC1	BEPRIDIL	other/unknown	TdgClinicalTrial|TEND
TNNC1	TNNC1	PURPUROGALLIN		DTC
TNNC1	TNNC1	PYROGALLOL RED		DTC
TNNC1	TNNC1	CHEMBL1601846		DTC
CD70	CD70	MDX-1411		TdgClinicalTrial|TTD
CD70	CD70	VORSETUZUMAB MAFODOTIN		TdgClinicalTrial
GSTM3	GSTM3	OLANZAPINE		PharmGKB
F11	F11	THROMBIN	activator	NCI
F11	F11	SULFATED PENTAGALLOYLGLUCOSIDE		DTC
SCARB1	SCARB1	RIBAVIRIN		PharmGKB
SCARB1	SCARB1	ATORVASTATIN		PharmGKB
SCARB1	SCARB1	FENOFIBRATE		PharmGKB
SLC16A3	SLC16A3	STREPTOZOCIN		NCI
SLC15A2	SLC15A2	SORAFENIB		PharmGKB
TYMP	TYMP	URIDINE		TTD
TYMP	TYMP	FLUOROURACIL		NCI|PharmGKB
TYMP	TYMP	CAPECITABINE		PharmGKB
TYMP	TYMP	TIPIRACIL HYDROCHLORIDE	inhibitor	ChemblInteractions
TYMP	TYMP	PROGESTERONE		NCI
TYMP	TYMP	SULFASALAZINE		NCI
FGF1	FGF1	MUPARFOSTAT	inhibitor	ChemblInteractions
SCNN1A	SCNN1A	TRIAMTERENE	inhibitor|blocker	TdgClinicalTrial|ChemblInteractions|TEND|PharmGKB
SCNN1A	SCNN1A	AMILORIDE	inhibitor|blocker	TdgClinicalTrial|TEND|PharmGKB
SCNN1A	SCNN1A	AMILORIDE HYDROCHLORIDE	blocker	ChemblInteractions
SCNN1A	SCNN1A	P-1037	blocker	ChemblInteractions
SDS	SDS	DEXAMETHASONE		NCI
COL4A6	COL4A6	OCRIPLASMIN		ChemblInteractions
COL4A6	COL4A6	COLLAGENASE CLOSTRIDIUM HISTOLYTICUM		ChemblInteractions
COL5A3	COL5A3	OCRIPLASMIN		ChemblInteractions
COL5A3	COL5A3	COLLAGENASE CLOSTRIDIUM HISTOLYTICUM		ChemblInteractions
ESRRG	ESRRG	DAIDZEIN	agonist	GuideToPharmacology
ESRRG	ESRRG	DIETHYLSTILBESTROL	antagonist|agonist	NCI|GuideToPharmacology

ESRRG	ESRRG	GENISTEIN	agonist	GuideToPharmacology
ESRRG	ESRRG	AFIMOXIFENE	antagonist	ChemblInteractions
ESRRG	ESRRG	AFIMOXIFENE	agonist	GuideToPharmacology
ESRRG	ESRRG	CHEMBL193836	agonist	GuideToPharmacology
ESRRG	ESRRG	CHEMBL201013	agonist	GuideToPharmacology
PIK3R5	PIK3R5	QUERCETIN	inhibitor	MyCancerGenome
PIK3R5	PIK3R5	BGT-226	inhibitor	ChemblInteractions
PIK3R5	PIK3R5	WX-037	inhibitor	ChemblInteractions
PIK3R5	PIK3R5	OMIPALISIB	inhibitor	ChemblInteractions|MyCancerGenomeClinicalTrial
PIK3R5	PIK3R5	VS-5584	inhibitor	ChemblInteractions
PIK3R5	PIK3R5	PUQUITINIB	inhibitor	ChemblInteractions
PIK3R5	PIK3R5	PILARALISIB	inhibitor	MyCancerGenome|ChemblInteractions
PIK3R5	PIK3R5	RG-7666	inhibitor	ChemblInteractions
PIK3R5	PIK3R5	PI-103	inhibitor	MyCancerGenome
PIK3R5	PIK3R5	GSK-2636771	inhibitor	MyCancerGenome
PIK3R5	PIK3R5	VOXTALISIB	inhibitor	MyCancerGenome|ChemblInteractions
PIK3R5	PIK3R5	RECILISIB	inhibitor	ChemblInteractions
PIK3R5	PIK3R5	TASELISIB	inhibitor	ChemblInteractions
PIK3R5	PIK3R5	AZD-6482	inhibitor	ChemblInteractions
PIK3R5	PIK3R5	BUPARLISIB	inhibitor	MyCancerGenome|ChemblInteractions
PIK3R5	PIK3R5	GEDATOLISIB	inhibitor	MyCancerGenome|ChemblInteractions|MyCancerGenomeClinicalTrial
PIK3R5	PIK3R5	DACTOLISIB	inhibitor	MyCancerGenome|ChemblInteractions|MyCancerGenomeClinicalTrial
PIK3R5	PIK3R5	INFIGRATINIB	inhibitor	MyCancerGenome
PIK3R5	PIK3R5	PWT-33587	inhibitor	ChemblInteractions
PIK3R5	PIK3R5	APITOLISIB	inhibitor	MyCancerGenome|ChemblInteractions
PIK3R5	PIK3R5	SF-1126	inhibitor	ChemblInteractions
PIK3R5	PIK3R5	SONOLISIB	inhibitor	MyCancerGenome|ChemblInteractions|MyCancerGenomeClinicalTrial
PIK3R5	PIK3R5	ALPELISIB	inhibitor	MyCancerGenome|MyCancerGenomeClinicalTrial
PIK3R5	PIK3R5	PICTILISIB	inhibitor	MyCancerGenome|ChemblInteractions
PIK3R5	PIK3R5	SAMOTOLISIB	inhibitor	ChemblInteractions
PIK3R5	PIK3R5	PANULISIB	inhibitor	ChemblInteractions
PIK3R5	PIK3R5	COPANLISIB	inhibitor	MyCancerGenome|ChemblInteractions|MyCancerGenomeClinicalTrial
PIK3R5	PIK3R5	ZSTK-474	inhibitor	ChemblInteractions
PIK3R5	PIK3R5	PF-04691502	inhibitor	MyCancerGenome|ChemblInteractions
PIK3R5	PIK3R5	GSK-1059615	inhibitor	ChemblInteractions
PIK3R5	PIK3R5	DS-7423	inhibitor	ChemblInteractions
PIK3R5	PIK3R5	PA-799	inhibitor	ChemblInteractions
CEL	CEL	GENISTEIN		NCI
CEL	CEL	RESVERATROL		NCI
CEL	CEL	SIMVASTATIN		NCI
CEL	CEL	GELDANAMYCIN		NCI
EGLN3	EGLN3	DAPRODUSTAT	inhibitor	ChemblInteractions
EGLN3	EGLN3	ROXADUSTAT	inhibitor	TdgClinicalTrial|ChemblInteractions
NNMT	NNMT	NIACIN	binder	TdgClinicalTrial
PDE1A	PDE1A	CRISABOROLE	inhibitor	GuideToPharmacology
PDE1A	PDE1A	PENTOXIFYLLINE	inhibitor	ChemblInteractions
PDE1A	PDE1A	DIPYRIDAMOLE	inhibitor	ChemblInteractions
PDE1A	PDE1A	VINPOCETINE	inhibitor	GuideToPharmacology
FCGR3A	FCGR3A	CETUXIMAB		NCI|PharmGKB
FCGR3A	FCGR3A	CYCLOSPORINE		NCI
FCGR3A	FCGR3A	INDOMETHACIN		NCI
FCGR3A	FCGR3A	ETANERCEPT	ligand	PharmGKB
FCGR3A	FCGR3A	THALIDOMIDE		NCI
FCGR3A	FCGR3A	TRASTUZUMAB		NCI|PharmGKB
FCGR3A	FCGR3A	ALDESLEUKIN		NCI
FCGR3A	FCGR3A	SODIUM CHLORIDE		NCI
FCGR3A	FCGR3A	RITUXIMAB		PharmGKB
FCGR3A	FCGR3A	EPOETIN ALFA		NCI
FCGR3A	FCGR3A	GELDANAMYCIN		NCI
FCGR3A	FCGR3A	PUROMYCIN		NCI
FCGR3A	FCGR3A	TESMILIFENE HYDROCHLORIDE		NCI

FCGR3A	FCGR3A	DOXORUBICIN		NCI
FCGR3A	FCGR3A	MAFOSFAMIDE		NCI
FCGR3A	FCGR3A	TOCILIZUMAB		PharmGKB
FCGR3A	FCGR3A	PENICILLIN G POTASSIUM		NCI
FCGR3A	FCGR3A	INFLIXIMAB		PharmGKB
FCGR3A	FCGR3A	CIMETIDINE		NCI
FCGR3A	FCGR3A	FENTANYL		NCI
FCGR3A	FCGR3A	LACTULOSE		NCI
FCGR3A	FCGR3A	ADALIMUMAB		PharmGKB
FCGR3A	FCGR3A	PREDNISOLONE		NCI
FCGR3A	FCGR3A	HEPARIN		NCI
HLA-B	HLA-B	PAZOPANIB		PharmGKB|FDA
HLA-B	HLA-B	MINOCYCLINE		PharmGKB
HLA-B	HLA-B	DAPSONE		PharmGKB
HLA-B	HLA-B	BCG VACCINE		NCI
HLA-B	HLA-B	TICLOPIDINE		PharmGKB
HLA-B	HLA-B	CLOZAPINE		PharmGKB
HLA-B	HLA-B	RIBAVIRIN		PharmGKB
HLA-B	HLA-B	CARBAMAZEPINE		NCI|FDA
HLA-B	HLA-B	PHENYTOIN		FDA
HLA-B	HLA-B	FOSPHENYTOIN		PharmGKB|FDA
HLA-B	HLA-B	STAVUDINE		PharmGKB
HLA-B	HLA-B	ABACAVIR		FDA
HLA-B	HLA-B	METHIMAZOLE		PharmGKB
HLA-B	HLA-B	OXCARBAZEPINE		FDA
HLA-B	HLA-B	CARBIMAZOLE		PharmGKB
HLA-B	HLA-B	CLAVULANIC ACID		PharmGKB
HLA-B	HLA-B	ACETAZOLAMIDE		PharmGKB
HLA-B	HLA-B	THALIDOMIDE		NCI
HLA-B	HLA-B	AMOXICILLIN		PharmGKB
HLA-B	HLA-B	FLOXACILLIN		PharmGKB
HLA-B	HLA-B	SULFASALAZINE		PharmGKB
HLA-B	HLA-B	METHAZOLAMIDE		PharmGKB
HLA-B	HLA-B	LAMIVUDINE		PharmGKB
HLA-B	HLA-B	CLINDAMYCIN		PharmGKB
HLA-B	HLA-B	TRICHLOROETHYLENE		PharmGKB
SCD	SCD	ARAMCHOL		TTD
SCD	SCD	COLCHICINE		NCI
SCD	SCD	CLOFIBRATE		NCI
SCD	SCD	ROSIGLITAZONE		NCI
SCD	SCD	MK-8245		TTD
NPHS1	NPHS1	LOSARTAN		PharmGKB
SLC12A3	SLC12A3	HYDROCHLOROTHIAZIDE	inhibitor	TdgClinicalTrial|ChemblInteractions|TEND|GuideToPharmacology
SLC12A3	SLC12A3	CHLOROTHIAZIDE	inhibitor	TdgClinicalTrial|ChemblInteractions|TEND|GuideToPharmacology
SLC12A3	SLC12A3	TRICHLORMETHIAZIDE	inhibitor	ChemblInteractions
SLC12A3	SLC12A3	BENZTHIAZIDE	inhibitor	TdgClinicalTrial|ChemblInteractions|TEND
SLC12A3	SLC12A3	METOLAZONE	inhibitor	TdgClinicalTrial|ChemblInteractions|TEND|GuideToPharmacology|TTD
SLC12A3	SLC12A3	QUINETHAZONE	inhibitor	TdgClinicalTrial|ChemblInteractions|TEND
SLC12A3	SLC12A3	DIAZOXIDE		TdgClinicalTrial|TEND
SLC12A3	SLC12A3	BUMETANIDE		PharmGKB
SLC12A3	SLC12A3	BENDROFLUMETHIAZIDE	inhibitor	TdgClinicalTrial|ChemblInteractions|TEND|TTD
SLC12A3	SLC12A3	HYDROFLUMETHIAZIDE	inhibitor	ChemblInteractions
SLC12A3	SLC12A3	POLYTHIAZIDE	inhibitor	TdgClinicalTrial|ChemblInteractions|TEND|TTD
SLC12A3	SLC12A3	FUROSEMIDE		PharmGKB
SLC12A3	SLC12A3	CYCLOTHIAZIDE	inhibitor	ChemblInteractions|GuideToPharmacology
SLC12A3	SLC12A3	TORSEMIDE		PharmGKB
SLC12A3	SLC12A3	CHLOROTHIAZIDE SODIUM	inhibitor	ChemblInteractions
SLC12A3	SLC12A3	CHLORTHALIDONE	inhibitor	ChemblInteractions
SLC12A3	SLC12A3	METHYCLOTHIAZIDE	inhibitor	ChemblInteractions
SLC12A3	SLC12A3	INDAPAMIDE	inhibitor	ChemblInteractions
NPTX2	NPTX2	PRM-151		TTD

VIM	VIM	PRITUMUMAB		ChemblInteractions
VIM	VIM	GELDANAMYCIN		NCI
VIM	VIM	ALDESLEUKIN		NCI
SLC16A5	SLC16A5	CISPLATIN		PharmGKB
ADM	ADM	CHEMBL35482		NCI
ADM	ADM	CHEMBL472004		DTC
ADM	ADM	INDOMETHACIN		NCI
ADM	ADM	CHEMBL2063510		DTC
ADM	ADM	PAROXETINE		PharmGKB
ADM	ADM	INSULIN		NCI
ADM	ADM	ALTEPLASE		NCI
PSMB8	PSMB8	CARFILZOMIB	inhibitor	DTC|MyCancerGenome|ChemblInteractions
PSMB8	PSMB8	OPROZOMIB	inhibitor	ChemblInteractions
PSMB8	PSMB8	BORTEZOMIB	inhibitor	DTC|MyCancerGenome|ChemblInteractions
PSMB8	PSMB8	KZR-616	inhibitor	GuideToPharmacology
PSMB8	PSMB8	BELACTOSIN A		DTC
PSMB8	PSMB8	MARIZOMIB	inhibitor	ChemblInteractions
PSMB8	PSMB8	IXAZOMIB CITRATE	inhibitor	ChemblInteractions
PSMB8	PSMB8	LACTACYSTIN		DTC
FOLR3	FOLR3	PEMETREXED		PharmGKB
TMSB10	TMSB10	MELATONIN		NCI
P2RX7	P2RX7	GSK1482160	allosteric modulator|antagonist	ChemblInteractions|GuideToPharmacology|TTD
P2RX7	P2RX7	CYCLIC ADENOSINE MONOPHOSPHATE	allosteric modulator	GuideToPharmacology
P2RX7	P2RX7	AZD9056	antagonist	ChemblInteractions|TTD
P2RX7	P2RX7	JNJ-54175446		TTD
P2RX7	P2RX7	CHELERYTHRINE	allosteric modulator	GuideToPharmacology
P2RX7	P2RX7	FENTANYL		PharmGKB
P2RX7	P2RX7	ADENOSINE TRIPHOSPHATE	agonist	GuideToPharmacology
P2RX7	P2RX7	EVT401	antagonist	TdgClinicalTrial|ChemblInteractions
P2RX7	P2RX7	IVERMECTIN	allosteric modulator	GuideToPharmacology
P2RX7	P2RX7	CE-224535	antagonist	ChemblInteractions|TTD
P2RX7	P2RX7	CLEMASTINE	allosteric modulator	GuideToPharmacology
KLK7	KLK7	CASTANIN		DTC
SLC6A3	SLC6A3	MODAFINIL	inhibitor	TdgClinicalTrial|ChemblInteractions|TTD
SLC6A3	SLC6A3	ACETYLCYSTEINE		NCI
SLC6A3	SLC6A3	ALTROPANE		TdgClinicalTrial|TTD
SLC6A3	SLC6A3	BENZTROPINE		TdgClinicalTrial|TEND
SLC6A3	SLC6A3	FENCAMFAMIN	inhibitor	TdgClinicalTrial|TEND
SLC6A3	SLC6A3	AMPHETAMINE	negative modulator	TdgClinicalTrial|ChemblInteractions|NCI|TEND
SLC6A3	SLC6A3	DEXMETHYLPHENIDATE	inhibitor	TdgClinicalTrial|GuideToPharmacology
SLC6A3	SLC6A3	PHENDIMETRAZINE TARTRATE	inhibitor	ChemblInteractions
SLC6A3	SLC6A3	SIBUTRAMINE HYDROCHLORIDE	inhibitor	ChemblInteractions
SLC6A3	SLC6A3	VANOXERINE	inhibitor	GuideToPharmacology
SLC6A3	SLC6A3	LISDEXAMFETAMINE	inhibitor	TdgClinicalTrial|TEND
SLC6A3	SLC6A3	DIETHYLPROPION HYDROCHLORIDE	inhibitor	ChemblInteractions
SLC6A3	SLC6A3	LEVOMILNACIPRAN	inhibitor	GuideToPharmacology
SLC6A3	SLC6A3	METHYLPHENIDATE	inhibitor	TdgClinicalTrial|ChemblInteractions|NCI|TEND|GuideToPharmacology|TTD
SLC6A3	SLC6A3	AMPHETAMINE ADIPATE		ChemblInteractions
SLC6A3	SLC6A3	ATOMOXETINE	binder|inhibitor	GuideToPharmacology
SLC6A3	SLC6A3	PSEUDOEPHEDRINE	inhibitor	TdgClinicalTrial
SLC6A3	SLC6A3	AMITIFADINE		TTD
SLC6A3	SLC6A3	DEXTROAMPHETAMINE SULFATE		ChemblInteractions
SLC6A3	SLC6A3	DEXTROAMPHETAMINE	negative modulator|inhibitor	TdgClinicalTrial|ChemblInteractions|TEND|GuideToPharmacology
SLC6A3	SLC6A3	SIBUTRAMINE	inhibitor	TdgClinicalTrial|GuideToPharmacology
SLC6A3	SLC6A3	METHAMPHETAMINE	negative modulator	TdgClinicalTrial
SLC6A3	SLC6A3	DASOTRALINE	inhibitor	ChemblInteractions|TTD
SLC6A3	SLC6A3	BUPROPION	inhibitor	TdgClinicalTrial|TEND|GuideToPharmacology
SLC6A3	SLC6A3	DISULFIRAM		PharmGKB
SLC6A3	SLC6A3	PHENMETRAZINE	inhibitor	TdgClinicalTrial|TEND|TTD
SLC6A3	SLC6A3	TRIMIPRAMINE	inhibitor	GuideToPharmacology
SLC6A3	SLC6A3	ALTROPANE I-123		TTD

SLC6A3	SLC6A3	DIETHYLPROPION	inhibitor	TdgClinicalTrial|TEND
SLC6A3	SLC6A3	HALOPERIDOL		PharmGKB
SLC6A3	SLC6A3	NICOTINE		NCI
SLC6A3	SLC6A3	SERTRALINE	binder|inhibitor	TdgClinicalTrial
SLC6A3	SLC6A3	COCAINE	inhibitor	TdgClinicalTrial|TEND|PharmGKB|TTD
SLC6A3	SLC6A3	AMPHETAMINE SULFATE		ChemblInteractions
SLC6A3	SLC6A3	PHENTERMINE	inhibitor	TdgClinicalTrial|TEND
SLC6A3	SLC6A3	CLOZAPINE		PharmGKB
SLC6A3	SLC6A3	MAZINDOL	inhibitor	ChemblInteractions|GuideToPharmacology
SLC6A3	SLC6A3	PHENELZINE	inhibitor	GuideToPharmacology
SLC6A3	SLC6A3	PHENMETRAZINE HYDROCHLORIDE	inhibitor	ChemblInteractions
SLC6A3	SLC6A3	TEDATIOXETINE	inhibitor	TdgClinicalTrial|ChemblInteractions
SLC6A3	SLC6A3	METHAMPHETAMINE HYDROCHLORIDE	inhibitor	ChemblInteractions
SLC6A3	SLC6A3	NOMIFENSINE	inhibitor	GuideToPharmacology
SLC6A3	SLC6A3	DEXMETHYLPHENIDATE HYDROCHLORIDE	inhibitor	ChemblInteractions
SLC6A3	SLC6A3	DEXTROAMPHETAMINE ADIPATE		ChemblInteractions
SLC6A3	SLC6A3	SPIROGLUMIDE		TTD
SLC6A3	SLC6A3	CLOMIPRAMINE	inhibitor	GuideToPharmacology
SLC6A3	SLC6A3	RTI-336		TTD
SLC6A3	SLC6A3	ARMODAFINIL	antagonist|inhibitor	TdgClinicalTrial|ChemblInteractions
SLC6A3	SLC6A3	BUPROPION HYDROCHLORIDE	inhibitor	ChemblInteractions
SLC6A3	SLC6A3	AMPHETAMINE ASPARTATE		ChemblInteractions
SLC6A3	SLC6A3	BUPROPION HYDROBROMIDE	inhibitor	ChemblInteractions
SLC6A3	SLC6A3	METHYLPHENIDATE HYDROCHLORIDE	inhibitor	ChemblInteractions
SLC6A3	SLC6A3	METYROSINE		NCI
SLC6A3	SLC6A3	HINOKININ		DTC
SLC6A3	SLC6A3	SOLRIAMFETOL	inhibitor	GuideToPharmacology
SLC6A3	SLC6A3	LISDEXAMFETAMINE DIMESYLATE		TdgClinicalTrial|ChemblInteractions
SLC6A3	SLC6A3	LIAFENSINE	inhibitor	ChemblInteractions
SLC6A3	SLC6A3	MIDOMAFETAMINE	negative modulator	TTD
SLC6A3	SLC6A3	LEVODOPA		PharmGKB
SLC6A3	SLC6A3	DESVENLAFAXINE	inhibitor	GuideToPharmacology
HSD11B2	HSD11B2	HYDROCORTISONE		NCI
ERBB4	ERBB4	PALIPERIDONE		PharmGKB
ERBB4	ERBB4	PELITINIB	inhibitor	TALC|MyCancerGenome
ERBB4	ERBB4	ERLOTINIB		DTC|JAX-CKB|PharmGKB
ERBB4	ERBB4	AC-480	inhibitor	TALC|MyCancerGenome|TdgClinicalTrial|ChemblInteractions|GuideToPharmacology
ERBB4	ERBB4	AFATINIB	inhibitor	JAX-CKB|CGI
ERBB4	ERBB4	LAPATINIB		DTC|JAX-CKB|CGI|DoCM|CIViC|PharmGKB
ERBB4	ERBB4	ILORASERTIB		DTC
ERBB4	ERBB4	CETUXIMAB		JAX-CKB
ERBB4	ERBB4	JNJ-26483327	inhibitor	ChemblInteractions
ERBB4	ERBB4	AZD-1152-HQPA		DTC
ERBB4	ERBB4	IBRUTINIB	inhibitor	GuideToPharmacology
ERBB4	ERBB4	DACOMITINIB	inhibitor	TALC|MyCancerGenome|TdgClinicalTrial|ChemblInteractions|GuideToPharmacology|MyCancerGenomeClinicalTrial
ERBB4	ERBB4	CURCUMIN		DTC
ERBB4	ERBB4	LAUROGUADINE		DTC
ERBB4	ERBB4	SP-600125		DTC
ERBB4	ERBB4	TUCATINIB	inhibitor	GuideToPharmacology
ERBB4	ERBB4	ALLITINIB	inhibitor	GuideToPharmacology
ERBB4	ERBB4	GEFITINIB	inhibitor	DTC|MyCancerGenomeClinicalTrial|PharmGKB
ERBB4	ERBB4	METHYLCURCUMIN		DTC
ERBB4	ERBB4	R-406		DTC
ERBB4	ERBB4	DIMETHYLCURCUMIN		DTC
ERBB4	ERBB4	AEE-788	inhibitor	GuideToPharmacology
ERBB4	ERBB4	POZIOTINIB	inhibitor	ChemblInteractions|GuideToPharmacology|MyCancerGenomeClinicalTrial
ERBB4	ERBB4	SB-220025		DTC
ERBB4	ERBB4	BMS-690514	inhibitor	TALC|MyCancerGenome|ChemblInteractions
ERBB4	ERBB4	TAK-715		DTC
ERBB4	ERBB4	OSIMERTINIB MESYLATE	inhibitor	ChemblInteractions
ERBB4	ERBB4	AFATINIB DIMALEATE	inhibitor	ChemblInteractions

ERBB4	ERBB4	TAE-684		DTC
ERBB4	ERBB4	CENISERTIB		DTC
ERBB4	ERBB4	VANDETANIB	inhibitor	ChemblInteractions
ERBB4	ERBB4	DASATINIB		DTC
ERBB4	ERBB4	CHEMBL578061		DTC
ERBB4	ERBB4	CANERTINIB DIHYDROCHLORIDE	inhibitor	ChemblInteractions
ERBB4	ERBB4	RG-1530		DTC
ERBB4	ERBB4	ACALABRUTINIB	inhibitor	GuideToPharmacology
ERBB4	ERBB4	ALISERTIB		DTC
ERBB4	ERBB4	CEDIRANIB		DTC
KCNK13	KCNK13	HALOTHANE	inhibitor	GuideToPharmacology
TRPM2	TRPM2	ECONAZOLE	blocker	GuideToPharmacology
TRPM2	TRPM2	CLOTRIMAZOLE	blocker	GuideToPharmacology
TRPM2	TRPM2	NADIDE	activator	GuideToPharmacology
TRPM2	TRPM2	MICONAZOLE	blocker	GuideToPharmacology
IL2RB	IL2RB	BASILIXIMAB	inhibitor|antibody	ChemblInteractions
IL2RB	IL2RB	ALDESLEUKIN	agonist	TdgClinicalTrial
IL2RB	IL2RB	DENILEUKIN DIFTITOX	agonist	ChemblInteractions
IL2RB	IL2RB	ALDESLEUKIN	modulator|agonist	TdgClinicalTrial|ChemblInteractions|TEND
IL2RB	IL2RB	DACLIZUMAB	inhibitor|antibody	ChemblInteractions
IL2RB	IL2RB	BEMPEGALDESLEUKIN	agonist	GuideToPharmacology
TNFRSF4	TNFRSF4	TAVOLIMAB		TTD
TNFRSF4	TNFRSF4	VONLEROLIZUMAB	antibody	GuideToPharmacology
TNFRSF4	TNFRSF4	IVUXOLIMAB		TTD
COL26A1	COL26A1	ASPIRIN		PharmGKB
COL26A1	COL26A1	CITALOPRAM		PharmGKB
FCER1G	FCER1G	ASPIRIN		PharmGKB
ENO2	ENO2	INDOMETHACIN		NCI
ENO2	ENO2	STREPTOZOCIN		NCI
ENO2	ENO2	INSULIN		NCI
ENO2	ENO2	FLOXURIDINE		NCI
ENO2	ENO2	GABAPENTIN		NCI
ENO2	ENO2	FENRETINIDE		NCI
ENO2	ENO2	LIOTHYRONINE SODIUM		NCI
ENO2	ENO2	PROPOFOL		NCI
PROX1	PROX1	ATENOLOL		PharmGKB
GRIK3	GRIK3	TEZAMPANEL	antagonist	TdgClinicalTrial|ChemblInteractions
GRIK3	GRIK3	SELURAMPANEL	antagonist	ChemblInteractions
GRIK3	GRIK3	TOPIRAMATE	antagonist	ChemblInteractions
GRIK3	GRIK3	DYSIHERBAINE	agonist	GuideToPharmacology
VEGFA	VEGFA	SQUALAMINE		TTD
VEGFA	VEGFA	FENOFIBRATE		NCI
VEGFA	VEGFA	AFLIBERCEPT	antibody|binder|inhibitor	MyCancerGenome|TdgClinicalTrial|ChemblInteractions|PharmGKB|TTD
VEGFA	VEGFA	BEVACIZUMAB	inhibitor|antibody	TALC|MyCancerGenome|TdgClinicalTrial|ClearityFoundationClinicalTrial|ChemblInteractions|TEND|CIViC|PharmGKB|TTD
VEGFA	VEGFA	ZALTRAP	antibody	TALC
VEGFA	VEGFA	SORAFENIB		CGI|PharmGKB
VEGFA	VEGFA	CARBOPLATIN		PharmGKB
VEGFA	VEGFA	ELMIRON		NCI
VEGFA	VEGFA	SUNITINIB		PharmGKB
VEGFA	VEGFA	MUPARFOSTAT	inhibitor	ChemblInteractions
VEGFA	VEGFA	OSI-632		TTD
VEGFA	VEGFA	RANIBIZUMAB	inhibitor	TdgClinicalTrial|ChemblInteractions|TEND|PharmGKB|TTD
VEGFA	VEGFA	BEVACIZUMAB 111IN		ChemblInteractions
VEGFA	VEGFA	IRINOTECAN		PharmGKB
VEGFA	VEGFA	REGORAFENIB		PharmGKB
VEGFA	VEGFA	ABICIPAR PEGOL		TTD
VEGFA	VEGFA	OXALIPLATIN		PharmGKB
VEGFA	VEGFA	CONBERCEPT	inhibitor	ChemblInteractions
VEGFA	VEGFA	BROLUCIZUMAB	inhibitor	ChemblInteractions
VEGFA	VEGFA	LENALIDOMIDE		ClearityFoundationClinicalTrial
VEGFA	VEGFA	NAVICIXIZUMAB		TTD

VEGFA	VEGFA	LEUCOVORIN		PharmGKB
VEGFA	VEGFA	DOMATINOSTAT		TdgClinicalTrial
VEGFA	VEGFA	PEGAPTANIB SODIUM	antagonist	TdgClinicalTrial|ChemblInteractions|PharmGKB
VEGFA	VEGFA	DOCETAXEL		PharmGKB
VEGFA	VEGFA	CAPECITABINE		PharmGKB
VEGFA	VEGFA	PHENYTOIN		NCI
VEGFA	VEGFA	SILDENAFIL		PharmGKB
VEGFA	VEGFA	GENTAMICIN		NCI
VEGFA	VEGFA	BEVASIRANIB		TdgClinicalTrial
VEGFA	VEGFA	CDC-801		NCI
VEGFA	VEGFA	CILOSTAZOL		NCI
VEGFA	VEGFA	FLUOROURACIL		PharmGKB
VEGFA	VEGFA	CELECOXIB		PharmGKB
VEGFA	VEGFA	CISPLATIN		PharmGKB
VEGFA	VEGFA	MP-0250		TTD
VEGFA	VEGFA	RISUTEGANIB		TTD
VEGFA	VEGFA	ENALAPRIL		PharmGKB
GABRA2	GABRA2	HALAZEPAM	positive modulator|potentiator	TdgClinicalTrial|ChemblInteractions|TEND
GABRA2	GABRA2	CLONAZEPAM	allosteric modulator|positive mo	TdgClinicalTrial|ChemblInteractions|TEND|GuideToPharmacology
GABRA2	GABRA2	FLUMAZENIL	allosteric modulator	TdgClinicalTrial|ChemblInteractions|TEND|GuideToPharmacology|TTD
GABRA2	GABRA2	BROMAZEPAM	potentiator	TdgClinicalTrial|TEND
GABRA2	GABRA2	CINOLAZEPAM	potentiator	TdgClinicalTrial|TEND
GABRA2	GABRA2	BUTETHAL	potentiator	TdgClinicalTrial|TEND
GABRA2	GABRA2	PHENOBARBITAL		TdgClinicalTrial|TEND
GABRA2	GABRA2	ISOGUVACINE	agonist	GuideToPharmacology
GABRA2	GABRA2	FLUNITRAZEPAM	allosteric modulator|potentiator	TdgClinicalTrial|TEND|GuideToPharmacology
GABRA2	GABRA2	GANAXOLONE		TdgClinicalTrial
GABRA2	GABRA2	PAGOCLONE		TdgClinicalTrial
GABRA2	GABRA2	TALBUTAL	potentiator|positive modulator	TdgClinicalTrial|ChemblInteractions|TEND
GABRA2	GABRA2	PRIMIDONE	positive modulator|potentiator	TdgClinicalTrial|ChemblInteractions|TEND
GABRA2	GABRA2	DIAZEPAM	allosteric modulator|positive mo	TdgClinicalTrial|ChemblInteractions|TEND|GuideToPharmacology
GABRA2	GABRA2	ENFLURANE		ChemblInteractions
GABRA2	GABRA2	ADIPIPLON	partial agonist	TdgClinicalTrial|ChemblInteractions
GABRA2	GABRA2	MEPHOBARBITAL	potentiator	TdgClinicalTrial|TEND
GABRA2	GABRA2	PICROTOXIN	blocker	GuideToPharmacology
GABRA2	GABRA2	AZD7325	positive modulator|allosteric mo	ChemblInteractions|GuideToPharmacology
GABRA2	GABRA2	ADINAZOLAM	potentiator	TdgClinicalTrial|TEND
GABRA2	GABRA2	HEXOBARBITAL	potentiator	TdgClinicalTrial|TEND
GABRA2	GABRA2	TEMAZEPAM	positive modulator|potentiator	TdgClinicalTrial|ChemblInteractions|TEND
GABRA2	GABRA2	TRIAZOLAM	allosteric modulator|potentiator|	TdgClinicalTrial|ChemblInteractions|TEND|GuideToPharmacology
GABRA2	GABRA2	ALPRAZOLAM	allosteric modulator|positive mo	TdgClinicalTrial|ChemblInteractions|TEND|GuideToPharmacology
GABRA2	GABRA2	QUAZEPAM	potentiator|positive modulator	TdgClinicalTrial|ChemblInteractions|TEND
GABRA2	GABRA2	THIOPENTAL SODIUM	positive modulator	ChemblInteractions
GABRA2	GABRA2	MIDAZOLAM	potentiator	TdgClinicalTrial|TEND
GABRA2	GABRA2	TRICLOFOS SODIUM		ChemblInteractions
GABRA2	GABRA2	NITRAZEPAM	potentiator	TdgClinicalTrial|TEND
GABRA2	GABRA2	DESFLURANE	positive modulator	ChemblInteractions
GABRA2	GABRA2	MEPROBAMATE	agonist	TdgClinicalTrial|ChemblInteractions|TEND
GABRA2	GABRA2	PRAZEPAM	potentiator|positive modulator	TdgClinicalTrial|ChemblInteractions|TEND
GABRA2	GABRA2	CHLORDIAZEPOXIDE	positive modulator	TdgClinicalTrial|ChemblInteractions|TEND
GABRA2	GABRA2	CLOBAZAM	positive modulator	TdgClinicalTrial|ChemblInteractions|TEND
GABRA2	GABRA2	OXAZEPAM	positive modulator|potentiator	TdgClinicalTrial|ChemblInteractions|TEND
GABRA2	GABRA2	PROPOFOL	positive modulator	ChemblInteractions
GABRA2	GABRA2	ETOMIDATE		DTC|ChemblInteractions
GABRA2	GABRA2	MUSCIMOL	agonist	GuideToPharmacology
GABRA2	GABRA2	PF-06372865	allosteric modulator|agonist	ChemblInteractions|GuideToPharmacology
GABRA2	GABRA2	MIDAZOLAM HYDROCHLORIDE	positive modulator	ChemblInteractions
GABRA2	GABRA2	BUTALBITAL	positive modulator|potentiator	TdgClinicalTrial|ChemblInteractions|TEND
GABRA2	GABRA2	ESTAZOLAM	potentiator|positive modulator	TdgClinicalTrial|ChemblInteractions|TEND
GABRA2	GABRA2	TOPIRAMATE		ChemblInteractions
GABRA2	GABRA2	METHARBITAL	potentiator|positive modulator	TdgClinicalTrial|ChemblInteractions|TEND

GABRA2	GABRA2	PENTOBARBITAL SODIUM	positive modulator	ChemblInteractions
GABRA2	GABRA2	ZOPICLONE	potentiator	TdgClinicalTrial|TEND
GABRA2	GABRA2	FLUDIAZEPAM	potentiator	TdgClinicalTrial|TEND
GABRA2	GABRA2	ZOLPIDEM	agonist|allosteric modulator	TdgClinicalTrial|TEND|GuideToPharmacology
GABRA2	GABRA2	GABOXADOL	agonist	TdgClinicalTrial|ChemblInteractions|GuideToPharmacology
GABRA2	GABRA2	METHYPRYLON	positive modulator	ChemblInteractions
GABRA2	GABRA2	COCAINE		PharmGKB
GABRA2	GABRA2	CLORAZEPIC ACID		TdgClinicalTrial|TEND
GABRA2	GABRA2	METHOHEXITAL SODIUM	positive modulator	ChemblInteractions
GABRA2	GABRA2	RESEQUINIL	inverse agonist	ChemblInteractions
GABRA2	GABRA2	OCINAPLON	allosteric modulator	TdgClinicalTrial|GuideToPharmacology
GABRA2	GABRA2	ISOFLURANE	positive modulator	ChemblInteractions
GABRA2	GABRA2	LORAZEPAM	positive modulator	TdgClinicalTrial|ChemblInteractions|TEND
GABRA2	GABRA2	AZD6280	positive modulator	ChemblInteractions
GABRA2	GABRA2	METHOXYFLURANE		ChemblInteractions
GABRA2	GABRA2	SECOBARBITAL	potentiator	TdgClinicalTrial|TEND
GABRA2	GABRA2	GLUTETHIMIDE		ChemblInteractions
GABRA2	GABRA2	CLOTIAZEPAM	potentiator	TdgClinicalTrial|TEND
GABRA2	GABRA2	THIOPENTAL	potentiator	TdgClinicalTrial|TEND
GABRA2	GABRA2	BUTABARBITAL		TdgClinicalTrial|TEND
GABRA2	GABRA2	GABAZINE	antagonist	GuideToPharmacology
GABRA2	GABRA2	FLURAZEPAM	potentiator	TdgClinicalTrial|TEND
GABRA2	GABRA2	ETAZOLATE		TdgClinicalTrial
GABRA2	GABRA2	ESZOPICLONE	positive modulator|agonist	TdgClinicalTrial|ChemblInteractions
GABRA2	GABRA2	THIAMYLAL SODIUM	positive modulator	ChemblInteractions
GABRA2	GABRA2	BRETAZENIL	allosteric modulator	GuideToPharmacology
GABRA2	GABRA2	SECOBARBITAL SODIUM	positive modulator	ChemblInteractions
GABRA2	GABRA2	BUTABARBITAL SODIUM	positive modulator	ChemblInteractions
GABRA2	GABRA2	PENTOBARBITAL	potentiator|positive modulator	TdgClinicalTrial|ChemblInteractions|TEND
GABRA2	GABRA2	AZIETOMIDATE		DTC
GABRA2	GABRA2	ETHCHLORVYNOL		ChemblInteractions
GABRA2	GABRA2	SEVOFLURANE	positive modulator	ChemblInteractions
GABRA2	GABRA2	CHLORDIAZEPOXIDE HYDROCHLORIDE	positive modulator	ChemblInteractions
GABRA2	GABRA2	BICUCULLINE	antagonist	GuideToPharmacology
GABRA2	GABRA2	MK-0777	allosteric modulator	GuideToPharmacology
GABRA2	GABRA2	REMIMAZOLAM		TdgClinicalTrial
GABRA2	GABRA2	HALOTHANE	positive modulator	ChemblInteractions
GABRA2	GABRA2	ACAMPROSATE CALCIUM		ChemblInteractions
GABRA2	GABRA2	LOREDIPLON	positive modulator	ChemblInteractions
GABRA2	GABRA2	CLORAZEPATE DIPOTASSIUM	positive modulator	ChemblInteractions
AVPR2	AVPR2	TOLVAPTAN	antagonist	TdgClinicalTrial|ChemblInteractions|TEND|GuideToPharmacology|TTD
AVPR2	AVPR2	DESMOPRESSIN	agonist	TdgClinicalTrial|GuideToPharmacology|TTD
AVPR2	AVPR2	VASOPRESSIN	agonist	TdgClinicalTrial|ChemblInteractions|TEND|GuideToPharmacology|TTD
AVPR2	AVPR2	VASOPRESSIN TANNATE	agonist	ChemblInteractions
AVPR2	AVPR2	LIXIVAPTAN	antagonist	TdgClinicalTrial|ChemblInteractions|GuideToPharmacology|TTD
AVPR2	AVPR2	CONIVAPTAN HYDROCHLORIDE	antagonist	ChemblInteractions
AVPR2	AVPR2	TERLIPRESSIN	agonist	TdgClinicalTrial|TEND
AVPR2	AVPR2	MOZAVAPTAN	antagonist	GuideToPharmacology|TTD
AVPR2	AVPR2	FEDOVAPAGON		TTD
AVPR2	AVPR2	CONIVAPTAN	antagonist	TdgClinicalTrial|TEND|GuideToPharmacology|TTD
AVPR2	AVPR2	OXYTOCIN	agonist	GuideToPharmacology|TTD
AVPR2	AVPR2	SATAVAPTAN	antagonist	ChemblInteractions|GuideToPharmacology|TTD
AVPR2	AVPR2	CHEMBL296908	antagonist	GuideToPharmacology
AVPR2	AVPR2	CHEMBL435323	agonist	GuideToPharmacology
AVPR2	AVPR2	LYPRESSIN	agonist	ChemblInteractions
AVPR2	AVPR2	RELCOVAPTAN	antagonist	GuideToPharmacology
AVPR2	AVPR2	QUERCETIN		DTC
AVPR2	AVPR2	ITRACONAZOLE		DTC
AVPR2	AVPR2	CLOTRIMAZOLE		DTC
AVPR2	AVPR2	DESMOPRESSIN ACETATE	agonist	ChemblInteractions
AVPR2	AVPR2	RO-5028442	antagonist	GuideToPharmacology

AVPR2	AVPR2	NELIVAPTAN	antagonist	GuideToPharmacology
AVPR2	AVPR2	TETRA-IODOPHENOLPHTHALEIN		DTC
AVPR2	AVPR2	BALOVAPTAN	antagonist	GuideToPharmacology
AVPR2	AVPR2	ATOSIBAN	antagonist	GuideToPharmacology|TTD
CD247	CD247	MUROMONAB-CD3		TdgClinicalTrial
CD247	CD247	AZACITIDINE		NCI
CD247	CD247	BLINATUMOMAB		TdgClinicalTrial
CRHBP	CRHBP	ALCOHOL		PharmGKB
C3	C3	COMPSTATIN		DTC
C3	C3	PEGCETACOPLAN		TTD
C3	C3	CLOZAPINE		PharmGKB
FCGR1A	FCGR1A	MDX-447		ChemblInteractions|NCI
FCGR1A	FCGR1A	MDX-210		ChemblInteractions
FCGR1A	FCGR1A	CEP-11981		DTC
RHBDF2	RHBDF2	FENTANYL		PharmGKB
RHBDF2	RHBDF2	MORPHINE		PharmGKB
RHBDF2	RHBDF2	OXYCODONE		PharmGKB
HLA-A	HLA-A	TERBINAFINE		PharmGKB
HLA-A	HLA-A	ZONISAMIDE		PharmGKB
HLA-A	HLA-A	TICLOPIDINE		PharmGKB
HLA-A	HLA-A	CARBAMAZEPINE		FDA
HLA-A	HLA-A	RIBAVIRIN		PharmGKB
HLA-A	HLA-A	VANCOMYCIN		PharmGKB
HLA-A	HLA-A	LEVETIRACETAM		PharmGKB
HLA-A	HLA-A	SERTRALINE		PharmGKB
HLA-A	HLA-A	FENOFIBRATE		PharmGKB
HLA-A	HLA-A	CLAVULANIC ACID		PharmGKB
HLA-A	HLA-A	DAPSONE		PharmGKB
HLA-A	HLA-A	IPILIMUMAB		PharmGKB|FDA
HLA-A	HLA-A	AMPICILLIN		PharmGKB
HLA-A	HLA-A	BACAMPICILLIN		PharmGKB
HLA-A	HLA-A	AMOXICILLIN		PharmGKB
CD40	CD40	RAVAGALIMAB		TTD
CD40	CD40	ISCALIMAB	antibody	GuideToPharmacology|TTD
CD40	CD40	LUCATUMUMAB		TdgClinicalTrial
CD40	CD40	TENELIXIMAB	partial agonist	ChemblInteractions
CD40	CD40	HYDROQUINONE		NCI
CD40	CD40	APX-005M		TTD
CD40	CD40	BLESELUMAB		TTD
CD40	CD40	STREPTOZOCIN		NCI
CD40	CD40	DACETUZUMAB	inhibitor	TdgClinicalTrial|ChemblInteractions
CD40	CD40	PG-102	inhibitor	ChemblInteractions
CD40	CD40	FLUDARABINE		NCI
PRKAR2B	PRKAR2B	THEOPHYLLINE		NCI
PSMB9	PSMB9	CARFILZOMIB	inhibitor	DTC|MyCancerGenome|ChemblInteractions
PSMB9	PSMB9	KZR-616	inhibitor	GuideToPharmacology
PSMB9	PSMB9	OPROZOMIB	inhibitor	ChemblInteractions
PSMB9	PSMB9	BORTEZOMIB	inhibitor	DTC|MyCancerGenome|ChemblInteractions
PSMB9	PSMB9	MARIZOMIB	inhibitor	ChemblInteractions
PSMB9	PSMB9	IXAZOMIB CITRATE	inhibitor	ChemblInteractions
GCGR	GCGR	SAR425899	agonist	ChemblInteractions|TTD
GCGR	GCGR	REDIOCIDE A		DTC
GCGR	GCGR	LGD-6972		TTD
GCGR	GCGR	GLUCAGON HYDROCHLORIDE RECOMBINANT	agonist	ChemblInteractions
GCGR	GCGR	GLUCAGON HYDROCHLORIDE	agonist	ChemblInteractions
GCGR	GCGR	ADOMEGLIVANT	antagonist	GuideToPharmacology|TTD
GCGR	GCGR	GLUCAGON	agonist	DTC|ChemblInteractions|GuideToPharmacology
GCGR	GCGR	PF-06291874	antagonist	ChemblInteractions|TTD
GCGR	GCGR	MK-0893	antagonist|allosteric modulator	DTC|ChemblInteractions|GuideToPharmacology
GCGR	GCGR	PEGAPAMODUTIDE		TTD
GCGR	GCGR	CROTEDUMAB	antibody	GuideToPharmacology

GCGR	GCGR	VOLAGIDEMAB		TTD
TRPV2	TRPV2	AMILORIDE	blocker	GuideToPharmacology
TRPV2	TRPV2	CANNABIDIOL	activator	GuideToPharmacology
CD1D	CD1D	RGI-7000		DTC
MAPK4	MAPK4	SORAFENIB		PharmGKB
LAMA4	LAMA4	OCRIPLASMIN		ChemblInteractions
EPCAM	EPCAM	OPORTUZUMAB MONATOX		TdgClinicalTrial|ChemblInteractions|TTD
EPCAM	EPCAM	TUCOTUZUMAB CELMOLEUKIN		ChemblInteractions
EPCAM	EPCAM	ING-1		TdgClinicalTrial|ChemblInteractions|TTD
EPCAM	EPCAM	ADECATUMUMAB	inhibitor	TdgClinicalTrial|ChemblInteractions
EPCAM	EPCAM	TRASTUZUMAB		NCI
EPCAM	EPCAM	SOLITOMAB	antibody	TALC|TdgClinicalTrial
EPCAM	EPCAM	CITATUZUMAB BOGATOX		TdgClinicalTrial|ChemblInteractions
EPCAM	EPCAM	CATUMAXOMAB	ligand|antibody	TALC|TdgClinicalTrial
LST1	LST1	ABACAVIR		PharmGKB
IDO1	IDO1	PHENYLHYDRAZINE		DTC
IDO1	IDO1	LINRODOSTAT	inhibitor	GuideToPharmacology|TTD
IDO1	IDO1	2-MERCAPTOBENZOTHIAZOLE		DTC
IDO1	IDO1	PF-06840003	inhibitor	GuideToPharmacology|TTD
IDO1	IDO1	CHEMBL14145		DTC
IDO1	IDO1	EPACADOSTAT	inhibitor	ChemblInteractions|GuideToPharmacology|TTD
IDO1	IDO1	NECROSTATIN	inhibitor	GuideToPharmacology
IDO1	IDO1	NORHARMANE	inhibitor	GuideToPharmacology
IDO1	IDO1	CHEMBL1668301		DTC
IDO1	IDO1	TRYPTOPHAN		TTD
IDO1	IDO1	TRYPTANTHRIN	inhibitor	GuideToPharmacology
IDO1	IDO1	CHEMBL1224312		DTC
IDO1	IDO1	NAVOXIMOD	inhibitor	GuideToPharmacology
IDO1	IDO1	CHEMBL1933308		DTC
DIO1	DIO1	PROPYLTHIOURACIL	inhibitor	ChemblInteractions|TTD
XPNPEP2	XPNPEP2	TOSEDOSTAT	inhibitor	ChemblInteractions
LGALS9	LGALS9	LACTOSE, ANHYDROUS		DTC
CSF3R	CSF3R	FILGRASTIM	stimulator|agonist	TdgClinicalTrial|ChemblInteractions|TEND|GuideToPharmacology|TTD
CSF3R	CSF3R	PEGFILGRASTIM	agonist	TdgClinicalTrial|ChemblInteractions|TEND|GuideToPharmacology|TTD
CSF3R	CSF3R	PEXIDARTINIB		TTD
CSF3R	CSF3R	RUXOLITINIB		JAX-CKB|DoCM|CIViC
CSF3R	CSF3R	BALUGRASTIM	agonist	ChemblInteractions
CSF3R	CSF3R	LIPEGFILGRASTIM	agonist	ChemblInteractions
CSF3R	CSF3R	DASATINIB		CGI
CSF3R	CSF3R	PLX-5622		TTD
E2F1	E2F1	DIETHYLSTILBESTROL		NCI
E2F1	E2F1	CARMUSTINE		NCI
E2F1	E2F1	QUERCETIN		NCI
E2F1	E2F1	LARGAZOLE		DTC
E2F1	E2F1	VASOPRESSIN		NCI
E2F1	E2F1	ADRIAMYCIN		NCI
E2F1	E2F1	ACETYLCYSTEINE		NCI
E2F1	E2F1	IRINOTECAN		NCI
E2F1	E2F1	ETOPOSIDE		NCI
E2F1	E2F1	FLUOROURACIL		NCI
E2F1	E2F1	CISPLATIN		NCI
E2F1	E2F1	PRIMAQUINE		NCI
E2F1	E2F1	NICOTINE		NCI
E2F1	E2F1	METHOTREXATE		NCI
E2F1	E2F1	PACLITAXEL		NCI
E2F1	E2F1	BORTEZOMIB		NCI
MT1H	MT1H	CISPLATIN		NCI
MT1H	MT1H	STREPTOZOCIN		NCI
CYP2B6	CYP2B6	ARTEMISININ		PharmGKB
CYP2B6	CYP2B6	TAMOXIFEN		DTC
CYP2B6	CYP2B6	MITOTANE		PharmGKB

CYP2B6	CYP2B6	METHADONE		DTC|PharmGKB
CYP2B6	CYP2B6	VORICONAZOLE		DTC
CYP2B6	CYP2B6	ZIDOVUDINE		PharmGKB
CYP2B6	CYP2B6	SORAFENIB		PharmGKB
CYP2B6	CYP2B6	SIBUTRAMINE	inhibitor	GuideToPharmacology
CYP2B6	CYP2B6	MIDAZOLAM		PharmGKB
CYP2B6	CYP2B6	PRASUGREL		PharmGKB|FDA
CYP2B6	CYP2B6	THIOTEPA	inhibitor	GuideToPharmacology|PharmGKB
CYP2B6	CYP2B6	PHENOBARBITAL		NCI|PharmGKB
CYP2B6	CYP2B6	TRAMADOL		PharmGKB
CYP2B6	CYP2B6	RIFAMPIN		PharmGKB
CYP2B6	CYP2B6	ISONIAZID		PharmGKB
CYP2B6	CYP2B6	NICOTINE		PharmGKB
CYP2B6	CYP2B6	BICALUTAMIDE		NCI
CYP2B6	CYP2B6	CYCLOPHOSPHAMIDE		NCI|PharmGKB
CYP2B6	CYP2B6	TROFOSFAMIDE		NCI
CYP2B6	CYP2B6	COLCHICINE		NCI
CYP2B6	CYP2B6	CLOPIDOGREL		DTC
CYP2B6	CYP2B6	ARTEMETHER		PharmGKB
CYP2B6	CYP2B6	STAVUDINE		PharmGKB
CYP2B6	CYP2B6	OSPEMIFENE		PharmGKB|FDA
CYP2B6	CYP2B6	SELEGILINE		PharmGKB
CYP2B6	CYP2B6	IFOSFAMIDE		PharmGKB
CYP2B6	CYP2B6	NEVIRAPINE		NCI|PharmGKB
CYP2B6	CYP2B6	LAMIVUDINE		PharmGKB
CYP2B6	CYP2B6	EFAVIRENZ		NCI|FDA
CYP2B6	CYP2B6	PYRAZINAMIDE		PharmGKB
CYP2B6	CYP2B6	CARBAMAZEPINE		NCI|PharmGKB
CYP2B6	CYP2B6	6-O-BENZYLGUANINE		NCI
CYP2B6	CYP2B6	PHENYTOIN		PharmGKB
CYP2B6	CYP2B6	IMATINIB		PharmGKB
CYP2B6	CYP2B6	MIRTAZAPINE		PharmGKB
CYP2B6	CYP2B6	BUSULFAN		PharmGKB
CYP2B6	CYP2B6	TESTOSTERONE		NCI
CYP2B6	CYP2B6	TICLOPIDINE	inhibitor	GuideToPharmacology|PharmGKB
CYP2B6	CYP2B6	KETAMINE		PharmGKB
CYP2B6	CYP2B6	CHEMBL530733		DTC
CYP2B6	CYP2B6	DOXORUBICIN		PharmGKB
CYP2B6	CYP2B6	RITONAVIR		NCI
CYP2B6	CYP2B6	FLIBANSERIN		PharmGKB
CYP2B6	CYP2B6	COTININE		PharmGKB
CYP2B6	CYP2B6	SERTRALINE		PharmGKB
CYP2B6	CYP2B6	MEPERIDINE		PharmGKB
CYP2B6	CYP2B6	DIAZEPAM		PharmGKB
CYP2B6	CYP2B6	ETHAMBUTOL		PharmGKB
CYP2B6	CYP2B6	MEPHENYTOIN		PharmGKB
TUBB2B	TUBB2B	BRENTUXIMAB VEDOTIN	inhibitor	ChemblInteractions
TUBB2B	TUBB2B	PODOFILOX		DTC
TUBB2B	TUBB2B	CYCLOSTREPTIN		DTC
TUBB2B	TUBB2B	CHEMBL1935538		DTC
TUBB2B	TUBB2B	CHEMBL1795737		DTC
TUBB2B	TUBB2B	SAGOPILONE		ChemblInteractions
TUBB2B	TUBB2B	VERUBULIN		ChemblInteractions
TUBB2B	TUBB2B	FOSBRETABULIN DISODIUM	inhibitor	ChemblInteractions
TUBB2B	TUBB2B	ZAMPANOLIDE		DTC
TUBB2B	TUBB2B	ABT-751		DTC
TUBB2B	TUBB2B	COMBRETASTATIN A4		DTC
TUBB2B	TUBB2B	PACLITAXEL	inhibitor	DTC|ChemblInteractions
TUBB2B	TUBB2B	VINCRISTINE		DTC
TUBB2B	TUBB2B	VINORELBINE TARTRATE	inhibitor	ChemblInteractions
TUBB2B	TUBB2B	DOCETAXEL	inhibitor	ChemblInteractions

TUBB2B	TUBB2B	IXABEPILONE	inhibitor	ChemblInteractions
TUBB2B	TUBB2B	VINCRISTINE SULFATE	inhibitor	ChemblInteractions
TUBB2B	TUBB2B	INDIBULIN	inhibitor	ChemblInteractions
TUBB2B	TUBB2B	CROLIBULIN	inhibitor	ChemblInteractions
TUBB2B	TUBB2B	VINBLASTINE		DTC
TUBB2B	TUBB2B	MAYTANSINE		DTC
TUBB2B	TUBB2B	CHEMBL2036124		DTC
TUBB2B	TUBB2B	CURCUMIN		DTC
TUBB2B	TUBB2B	CHEMBL2036119		DTC
TUBB2B	TUBB2B	PLINABULIN	inhibitor	ChemblInteractions
TUBB2B	TUBB2B	PACLITAXEL POLIGLUMEX		ChemblInteractions
TUBB2B	TUBB2B	NOCODAZOLE		DTC
TUBB2B	TUBB2B	CHEMBL453818		DTC
TUBB2B	TUBB2B	MAYTANSINOL		DTC
TUBB2B	TUBB2B	CABAZITAXEL	inhibitor	ChemblInteractions
TUBB2B	TUBB2B	VINBLASTINE SULFATE	inhibitor	ChemblInteractions
TUBB2B	TUBB2B	VORINOSTAT		DTC
TUBB2B	TUBB2B	FOSBRETABULIN TROMETHAMINE		ChemblInteractions
TUBB2B	TUBB2B	VINORELBINE		DTC
TUBB2B	TUBB2B	ERIBULIN MESYLATE	inhibitor	ChemblInteractions
TUBB2B	TUBB2B	LARGAZOLE		DTC
TUBB2B	TUBB2B	COLCHICINE	inhibitor	DTC|ChemblInteractions
TUBB2B	TUBB2B	VINFLUNINE	inhibitor	ChemblInteractions
TUBB2B	TUBB2B	LEXIBULIN	inhibitor	ChemblInteractions
TUBB2B	TUBB2B	TRASTUZUMAB EMTANSINE	inhibitor	ChemblInteractions
TUBB2B	TUBB2B	DAVUNETIDE		ChemblInteractions
DNER	DNER	BIRABRESIB		TTD
DNER	DNER	MOLIBRESIB		TTD
DNER	DNER	BMS-986158		TTD
DNER	DNER	MIVEBRESIB		TTD
DNER	DNER	ALOBRESIB		TTD
IGFBP3	IGFBP3	CELECOXIB		PharmGKB
IGFBP3	IGFBP3	FLUOROURACIL		PharmGKB
SLC22A8	SLC22A8	PROBENECID	inhibitor	DTC|TdgClinicalTrial|ChemblInteractions|TEND|TTD
SLC22A8	SLC22A8	DICLOXACILLIN		DTC
SLC22A8	SLC22A8	BENZYLPENICILLIN	inhibitor|substrate	DTC
SLC22A8	SLC22A8	PRAVASTATIN		PharmGKB
SLC22A8	SLC22A8	CEFOTAXIME		PharmGKB
SLC22A8	SLC22A8	CEPHALOTHIN	inhibitor	GuideToPharmacology
SLC22A8	SLC22A8	FLUORESCEIN		TTD
SLC22A8	SLC22A8	OXACILLIN		DTC
SLC22A8	SLC22A8	ZIDOVUDINE		PharmGKB
SLC22A8	SLC22A8	RUCAPARIB		PharmGKB
SLC22A8	SLC22A8	PRATOSARTAN	inhibitor	ChemblInteractions
SLC22A8	SLC22A8	CEFACLOR		DTC
SLC22A8	SLC22A8	METHOTREXATE		PharmGKB
TNNT2	TNNT2	PYROGALLOL RED		DTC
TNNT2	TNNT2	PURPUROGALLIN		DTC
TNNT2	TNNT2	CHEMBL1601846		DTC
SELPLG	SELPLG	INCLACUMAB		TTD
SELPLG	SELPLG	NEIHULIZUMAB		TTD
RUNX3	RUNX3	LEUCOVORIN		NCI
RUNX3	RUNX3	IRINOTECAN		NCI
CD37	CD37	OTLERTUZUMAB	antibody	TdgClinicalTrial|GuideToPharmacology|TTD
CD37	CD37	NARATUXIMAB EMTANSINE		TTD
PLA2G7	PLA2G7	DARAPLADIB	inhibitor	TdgClinicalTrial|ChemblInteractions|GuideToPharmacology
PLA2G7	PLA2G7	RILAPLADIB	inhibitor	ChemblInteractions|GuideToPharmacology|TTD
PLA2G7	PLA2G7	EPOETIN BETA		NCI
SLC16A7	SLC16A7	METHOTREXATE		PharmGKB
BTK	BTK	CHEMBL202721		DTC
BTK	BTK	TIRABRUTINIB	inhibitor	ChemblInteractions|GuideToPharmacology|TTD

BTK	BTK	ZANUBRUTINIB	inhibitor	GuideToPharmacology|TTD
BTK	BTK	ACALABRUTINIB	inhibitor	GuideToPharmacology|TTD
BTK	BTK	MSC-2364447	inhibitor	ChemblInteractions
BTK	BTK	SPEBRUTINIB BESYLATE	inhibitor	TdgClinicalTrial|ChemblInteractions|TTD
BTK	BTK	CENISERTIB	inhibitor	TALC|DTC|MyCancerGenome
BTK	BTK	HM-71224	inhibitor	ChemblInteractions
BTK	BTK	ABIVERTINIB	inhibitor	GuideToPharmacology
BTK	BTK	IBRUTINIB	inhibitor	DTC|MyCancerGenome|TdgClinicalTrial|ChemblInteractions|CGI|DoCM|COSMIC
BTK	BTK	ALISERTIB		DTC
BTK	BTK	ILORASERTIB		DTC
BTK	BTK	BRANEBRUTINIB	inhibitor	GuideToPharmacology
BTK	BTK	TAE-684		DTC
BTK	BTK	MLN-8054		DTC
BTK	BTK	EVOBRUTINIB	inhibitor	GuideToPharmacology
BTK	BTK	R-406		DTC
BTK	BTK	CHEMBL1997335		DTC
BTK	BTK	CHEMBL406845		DTC
BTK	BTK	FENEBRUTINIB	inhibitor	GuideToPharmacology|TTD
BTK	BTK	BMS-986142	inhibitor	GuideToPharmacology|TTD
BTK	BTK	CHEMBL228043	inhibitor	GuideToPharmacology
BTK	BTK	SPEBRUTINIB	inhibitor	GuideToPharmacology
BTK	BTK	HESPERADIN	inhibitor	DTC
BTK	BTK	VECABRUTINIB	inhibitor	GuideToPharmacology|TTD
BTK	BTK	RG-1530		DTC
BTK	BTK	VANDETANIB		DTC
BTK	BTK	PD-0166285		DTC
BTK	BTK	TOZASERTIB		DTC
BTK	BTK	AZD-1152-HQPA		DTC
BTK	BTK	POSELTINIB	inhibitor	GuideToPharmacology
BTK	BTK	PF-00562271		DTC
BTK	BTK	ENTRECTINIB		DTC
BTK	BTK	DOVITINIB		DTC
BTK	BTK	DASATINIB		DTC
HPD	HPD	NITISINONE	inhibitor	TdgClinicalTrial|ChemblInteractions|TEND|TTD
SLC9A3	SLC9A3	TENAPANOR HYDROCHLORIDE	inhibitor	ChemblInteractions|TTD
SLC9A3	SLC9A3	ALBUMIN HUMAN		NCI
TAPBP	TAPBP	ASPIRIN		PharmGKB
ITGB2	ITGB2	ALCOHOL		NCI
ITGB2	ITGB2	SODIUM CHLORIDE		NCI
ITGB2	ITGB2	MLNM-2201	inhibitor	ChemblInteractions
ITGB2	ITGB2	INDOMETHACIN		NCI
ITGB2	ITGB2	ROVELIZUMAB	antagonist|antibody	ChemblInteractions|GuideToPharmacology
ITGB2	ITGB2	MYCOPHENOLATE MOFETIL		NCI
ITGB2	ITGB2	EFALIZUMAB	inhibitor	ChemblInteractions
ITGB2	ITGB2	CYCLOPHOSPHAMIDE		NCI
ITGB2	ITGB2	LIFITEGRAST	antagonist	ChemblInteractions
ITGB2	ITGB2	ERLIZUMAB	negative modulator	ChemblInteractions
ITGB2	ITGB2	ANTIBIOTIC		NCI
ITGB2	ITGB2	PREDNISONE		NCI
ITGB2	ITGB2	CYCLOSPORINE		NCI
ITGB2	ITGB2	METHYLPREDNISOLONE		NCI
ITGB2	ITGB2	COLCHICINE		NCI
ITGB2	ITGB2	PENTOXIFYLLINE		NCI
ITGB2	ITGB2	AME-133V	inhibitor	ChemblInteractions
ITGB2	ITGB2	BUTEIN		NCI
ITGB2	ITGB2	THALIDOMIDE		NCI
PRF1	PRF1	ALDESLEUKIN		NCI
PRF1	PRF1	EMAPALUMAB		PharmGKB|FDA
RPS6KA6	RPS6KA6	CHEMBL573107	inhibitor	GuideToPharmacology|TTD
RPS6KA6	RPS6KA6	CEP-1347		DTC
RPS6KA6	RPS6KA6	AT-9283	inhibitor	GuideToPharmacology

CTH	CTH	AMINOETHOXYVINYLGLYCINE	inhibitor	GuideToPharmacology
CTH	CTH	BUSULFAN		PharmGKB
CTH	CTH	CYCLOPHOSPHAMIDE		PharmGKB
SLC47A2	SLC47A2	METFORMIN		PharmGKB
SLC47A2	SLC47A2	CIMETIDINE	inhibitor	GuideToPharmacology
SLC47A2	SLC47A2	RUCAPARIB		PharmGKB
CAV1	CAV1	ALCOHOL		NCI
CAV1	CAV1	TESTOSTERONE		NCI
KISS1R	KISS1R	TAK-448	agonist	GuideToPharmacology
KISS1R	KISS1R	KISSPEPTIN-10	agonist	GuideToPharmacology
KISS1R	KISS1R	BENZETHONIUM CHLORIDE		DTC
FMO5	FMO5	METFORMIN		PharmGKB
GZMA	GZMA	CYCLOSPORINE		NCI
ANGPT2	ANGPT2	NESVACUMAB	inhibitor	TdgClinicalTrial|ChemblInteractions|TTD
ANGPT2	ANGPT2	MEDI-3617		TTD
ANGPT2	ANGPT2	TREBANANIB	inhibitor	ChemblInteractions
ANGPT2	ANGPT2	RIBAVIRIN		NCI
COL4A5	COL4A5	COLLAGENASE CLOSTRIDIUM HISTOLYTICUM		ChemblInteractions
COL4A5	COL4A5	OCRIPLASMIN		ChemblInteractions
VAV1	VAV1	EPOETIN ALFA		NCI
KCNQ1	KCNQ1	INDAPAMIDE	inhibitor	TdgClinicalTrial|TEND|TTD
KCNQ1	KCNQ1	CHEMBL298475	blocker	GuideToPharmacology
KCNQ1	KCNQ1	BEPRIDIL	inhibitor	TdgClinicalTrial|TEND
KCNQ1	KCNQ1	INSULIN		NCI
KCNQ1	KCNQ1	NERISPIRDINE	blocker	ChemblInteractions
KCNQ1	KCNQ1	EZOGABINE	blocker	TdgClinicalTrial|ChemblInteractions
KCNQ1	KCNQ1	DOLASETRON		NCI
KCNQ1	KCNQ1	INDOMETHACIN		TdgClinicalTrial
KCNQ1	KCNQ1	TEDISAMIL	blocker	ChemblInteractions
KCNQ1	KCNQ1	DALFAMPRIDINE	blocker	ChemblInteractions
KCNQ1	KCNQ1	CELECOXIB		PharmGKB
KCNQ1	KCNQ1	REPAGLINIDE		PharmGKB
KCNQ1	KCNQ1	GUANIDINE HYDROCHLORIDE	blocker	ChemblInteractions
KCNQ1	KCNQ1	TACROLIMUS		PharmGKB
ALB	ALB	RALTITREXED		NCI
ALB	ALB	EBSELEN		TTD
ALB	ALB	EPIGALOCATECHIN GALLATE		DTC
ALB	ALB	OXYPHENBUTAZONE		TTD
ALB	ALB	PHENYLBUTAZONE		DTC
ALB	ALB	EPIGALLOCATECHIN		DTC
ALB	ALB	[19F]-HALOPERIDOL		DTC
ALB	ALB	DIAZEPAM		DTC
ALB	ALB	IODIPAMIDE		TTD
ALB	ALB	(-)-EPICATECHIN		DTC
ALB	ALB	PYROGALLOL		DTC
ALB	ALB	AMODIAQUINE HYDROCHLORIDE		DTC
ALB	ALB	DICLOFENAC		DTC
ALB	ALB	WARFARIN		DTC
ALB	ALB	NAPROXEN		NCI
ALB	ALB	OLMESARTAN MEDOXOMIL		DTC
ALB	ALB	GADOFOSVESET		TTD
ALB	ALB	CHEMBL1945287		DTC
ALB	ALB	FLUCONAZOLE		DTC
ALB	ALB	EPICATECHIN GALLATE		DTC
ALB	ALB	COCHINCHINENIN C		DTC
BMP7	BMP7	PEGASPARGASE		PharmGKB
BMP7	BMP7	METHOTREXATE		PharmGKB
BMP7	BMP7	CYCLOPHOSPHAMIDE		PharmGKB
BMP7	BMP7	THIOGUANINE		PharmGKB
BMP7	BMP7	PREDNISONE		PharmGKB
BMP7	BMP7	DEXAMETHASONE		PharmGKB

BMP7	BMP7	CYTARABINE		PharmGKB
BMP7	BMP7	VINCRISTINE		PharmGKB
BMP7	BMP7	DAUNORUBICIN		PharmGKB
BMP7	BMP7	DOXORUBICIN		PharmGKB
ST6GAL1	ST6GAL1	FLOXACILLIN		PharmGKB
ABAT	ABAT	VIGABATRIN	inhibitor	TdgClinicalTrial|ChemblInteractions|TEND|GuideToPharmacology|TTD
ABAT	ABAT	TIAGABINE		TdgClinicalTrial|TEND
ABAT	ABAT	CHEMBL378577	inhibitor	GuideToPharmacology
ABAT	ABAT	CPP-115	inhibitor	GuideToPharmacology|TTD
ABAT	ABAT	4-HYDROXYBENZALDEHYDE		TTD
ABAT	ABAT	L-ALANINE	inhibitor	TTD
ABAT	ABAT	VALPROIC ACID		DTC
TBXAS1	TBXAS1	ASPIRIN		PharmGKB
TBXAS1	TBXAS1	PYRIDINE		TTD
TBXAS1	TBXAS1	OZAGREL	inhibitor	GuideToPharmacology
GRIK5	GRIK5	DYSIHERBAINE	agonist	GuideToPharmacology
GRIK5	GRIK5	TEZAMPANEL		TdgClinicalTrial
LTB4R	LTB4R	AMELUBANT	antagonist	ChemblInteractions|TTD
LTB4R	LTB4R	SC-41390	antagonist	GuideToPharmacology
LTB4R	LTB4R	CP-195543	antagonist	GuideToPharmacology|TTD
LTB4R	LTB4R	ETALOCIB	antagonist	ChemblInteractions|GuideToPharmacology
LTB4R	LTB4R	CHEMBL422420	antagonist	GuideToPharmacology
COL4A4	COL4A4	OCRIPLASMIN		ChemblInteractions
COL4A4	COL4A4	COLLAGENASE CLOSTRIDIUM HISTOLYTICUM		ChemblInteractions
SCN1B	SCN1B	ZONISAMIDE	inhibitor	TdgClinicalTrial
HMOX1	HMOX1	SORAFENIB		CIViC
HMOX1	HMOX1	SUNITINIB		CIViC
HMOX1	HMOX1	STANNSOPORFIN		TdgClinicalTrial|TTD
HMOX1	HMOX1	ASPIRIN		NCI
TYMS	TYMS	OSI-7904		TdgClinicalTrial
TYMS	TYMS	CAPECITABINE	inhibitor	ClearityFoundationBiomarkers|ClearityFoundationClinicalTrial|ChemblInteractions|GuideToPharmacology
TYMS	TYMS	METHOTREXATE	inhibitor	CIViC
TYMS	TYMS	IRINOTECAN		CIViC|PharmGKB
TYMS	TYMS	HYDROCORTISONE		NCI
TYMS	TYMS	TEGAFUR	inhibitor	TdgClinicalTrial
TYMS	TYMS	FLUOROURACIL	other/unknown|inhibitor	DTC|ClearityFoundationClinicalTrial|ChemblInteractions|CIViC
TYMS	TYMS	PEMETREXED	inhibitor	ClearityFoundationBiomarkers|TdgClinicalTrial|TEND|CIViC|GuideToPharmacology
TYMS	TYMS	RALTITREXED	inhibitor	TdgClinicalTrial|TEND|GuideToPharmacology|PharmGKB
TYMS	TYMS	INDOMETHACIN		NCI
TYMS	TYMS	TAMOXIFEN		NCI
TYMS	TYMS	DEXAMETHASONE		NCI|PharmGKB
TYMS	TYMS	HYDROXYCHLOROQUINE		PharmGKB
TYMS	TYMS	ASPARAGINASE		PharmGKB
TYMS	TYMS	DAUNORUBICIN		NCI|PharmGKB
TYMS	TYMS	CISPLATIN		NCI
TYMS	TYMS	DEOXYURIDINE MONOPHOSPHATE		DTC
TYMS	TYMS	AZATHIOPRINE		PharmGKB
TYMS	TYMS	FLOXURIDINE	inhibitor	TdgClinicalTrial|ClearityFoundationClinicalTrial|ChemblInteractions|TEND
TYMS	TYMS	ARFOLITIXORIN		TdgClinicalTrial
TYMS	TYMS	9-AMINOCAMPTOTHECIN		NCI
TYMS	TYMS	PLATINUM		PharmGKB
TYMS	TYMS	LEUCOVORIN		TdgClinicalTrial|TEND
TYMS	TYMS	DOXIFLURIDINE		NCI
TYMS	TYMS	RESERPINE		NCI
TYMS	TYMS	TOPOTECAN		NCI
TYMS	TYMS	PREDNISONE		PharmGKB
TYMS	TYMS	AZACITIDINE		PharmGKB
TYMS	TYMS	ENILURACIL		NCI
TYMS	TYMS	CARBOGEN		NCI
TYMS	TYMS	PHENTOLAMINE		NCI
TYMS	TYMS	SULFASALAZINE		PharmGKB

TYMS	TYMS	ETOPOSIDE	PharmGKB
TYMS	TYMS	CYTARABINE	PharmGKB
TYMS	TYMS	VINCRISTINE	NCI
TYMS	TYMS	THYMIDINE MONOPHOSPHATE	DTC
TYMS	TYMS	VERAPAMIL	NCI
APOBEC3G	APOBEC3G	CHEMBL1388703	DTC
APOBEC3G	APOBEC3G	SCOULERINE	DTC
APOBEC3G	APOBEC3G	CHEMBL122355	DTC
APOBEC3G	APOBEC3G	CHEMBL601550	DTC
APOBEC3G	APOBEC3G	CHEMBL410484	DTC
APOBEC3G	APOBEC3G	CHEMBL1520346	DTC
APOBEC3G	APOBEC3G	CHEMBL1451921	DTC
APOBEC3G	APOBEC3G	CHEMBL584635	DTC
APOBEC3G	APOBEC3G	CEFOTETAN	DTC
APOBEC3G	APOBEC3G	CHEMBL124006	DTC
APOBEC3G	APOBEC3G	CHEMBL609606	DTC
APOBEC3G	APOBEC3G	CHEMBL605003	DTC
APOBEC3G	APOBEC3G	CHEMBL582287	DTC
APOBEC3G	APOBEC3G	CHEMBL589421	DTC
APOBEC3G	APOBEC3G	CHEMBL576409	DTC
APOBEC3G	APOBEC3G	CHEMBL103583	DTC
APOBEC3G	APOBEC3G	2,3,4,4'-TETRAMETHOXYBIPHENYL	DTC
APOBEC3G	APOBEC3G	CHEMBL584442	DTC
APOBEC3G	APOBEC3G	PYRONIN Y	DTC
APOBEC3G	APOBEC3G	MYRICETIN	DTC
APOBEC3G	APOBEC3G	CHEMBL72365	DTC
APOBEC3G	APOBEC3G	CHEMBL116548	DTC
APOBEC3G	APOBEC3G	CHEMBL582675	DTC
APOBEC3G	APOBEC3G	MICHLER'S KETONE	DTC
APOBEC3G	APOBEC3G	SB-216763	DTC
APOBEC3G	APOBEC3G	CHEMBL585240	DTC
APOBEC3G	APOBEC3G	SEMAXANIB	DTC
APOBEC3G	APOBEC3G	CHEMBL118678	DTC
APOBEC3G	APOBEC3G	GLAUCINE METHIODIDE	DTC
APOBEC3G	APOBEC3G	CHEMBL590706	DTC
APOBEC3G	APOBEC3G	CHEMBL277148	DTC
APOBEC3G	APOBEC3G	BENZOQUINONE	DTC
APOBEC3G	APOBEC3G	CHEMBL584514	DTC
APOBEC3G	APOBEC3G	3-O-METHYLQUERCETIN	DTC
APOBEC3G	APOBEC3G	CHEMBL1287981	DTC
APOBEC3G	APOBEC3G	CHEMBL586670	DTC
APOBEC3G	APOBEC3G	CHEMBL589711	DTC
APOBEC3G	APOBEC3G	CHEMBL1390716	DTC
APOBEC3G	APOBEC3G	CHEMBL600360	DTC
APOBEC3G	APOBEC3G	CHEMBL600306	DTC
APOBEC3G	APOBEC3G	CGS-15943	DTC
APOBEC3G	APOBEC3G	CHEMBL600692	DTC
APOBEC3G	APOBEC3G	DIMETHYLPHENYLPIPERAZINIUM IODIDE	DTC
APOBEC3G	APOBEC3G	STILONIUM IODIDE	DTC
APOBEC3G	APOBEC3G	CHEMBL88147	DTC
APOBEC3G	APOBEC3G	CHEMBL578504	DTC
APOBEC3G	APOBEC3G	APOMORPHINE HYDROCHLORIDE HEMIHYDRATE	DTC
APOBEC3G	APOBEC3G	ELLIPTECINE	DTC
APOBEC3G	APOBEC3G	CHEMBL429095	DTC
APOBEC3G	APOBEC3G	CHEMBL192626	DTC
APOBEC3G	APOBEC3G	CHEMBL601744	DTC
APOBEC3G	APOBEC3G	CHEMBL592314	DTC
APOBEC3G	APOBEC3G	CHEMBL428064	DTC
APOBEC3G	APOBEC3G	PROTOAPIGENONE	DTC
APOBEC3G	APOBEC3G	CHEMBL1255944	DTC
APOBEC3G	APOBEC3G	CHEMBL609438	DTC

APOBEC3G	APOBEC3G	CHEMBL584849		DTC
APOBEC3G	APOBEC3G	CHEMBL313737		DTC
CD52	CD52	ALEMTUZUMAB	antibody|inhibitor	MyCancerGenome|TdgClinicalTrial|ChemblInteractions|NCI|TEND|GuideToPharmacology|TTD
GZMB	GZMB	HEXACHLOROPHENE		DTC
BMPR1B	BMPR1B	GALUNISERTIB	inhibitor	GuideToPharmacology
HCK	HCK	XL-228	inhibitor	ChemblInteractions
HCK	HCK	CHEMBL406845		DTC
HCK	HCK	DASATINIB	inhibitor	DTC|ChemblInteractions
HCK	HCK	MASITINIB		TdgClinicalTrial
HCK	HCK	BOSUTINIB	inhibitor	ChemblInteractions
HCK	HCK	ENMD-981693	inhibitor	ChemblInteractions
HCK	HCK	HESPERADIN	inhibitor	DTC
HCK	HCK	NINTEDANIB	inhibitor	TALC
HCK	HCK	MLN-8054		DTC
HCK	HCK	TG100-801	inhibitor	ChemblInteractions
HCK	HCK	GEFITINIB		DTC
HCK	HCK	AST-487		DTC
HCK	HCK	ILORASERTIB	inhibitor	ChemblInteractions
HCK	HCK	PEXMETINIB	inhibitor	GuideToPharmacology
HCK	HCK	R-406		DTC
HCK	HCK	IMATINIB MESYLATE		NCI
HCK	HCK	DORAMAPIMOD		TTD
HCK	HCK	IBRUTINIB	inhibitor	GuideToPharmacology
HCK	HCK	DOVITINIB		DTC
HCK	HCK	PF-00562271		DTC
THRB	THRB	MB07811	agonist	TdgClinicalTrial|ChemblInteractions|TTD
THRB	THRB	DEXTROTHYROXINE	agonist	ChemblInteractions|GuideToPharmacology
THRB	THRB	LEVOTHYROXINE	agonist	DTC|TdgClinicalTrial|TEND
THRB	THRB	CHEMBL297304		DTC
THRB	THRB	CHEMBL1397308		DTC
THRB	THRB	ERYTHROSINE		DTC
THRB	THRB	ROSE BENGAL		DTC
THRB	THRB	PROTOPORPHYRIN		DTC
THRB	THRB	CHEMBL581044		DTC
THRB	THRB	3-METHYLCHOLANTHRENE		DTC
THRB	THRB	WEDELOLACTONE		DTC
THRB	THRB	SHIKONIN		DTC
THRB	THRB	TIRATRICOL	agonist	GuideToPharmacology|TTD
THRB	THRB	AMMONIA SOLUTION, STRONG		DTC
THRB	THRB	HYPERICIN		DTC
THRB	THRB	DOXORUBICIN HYDROCHLORIDE		DTC
THRB	THRB	SOBETIROME	agonist	TdgClinicalTrial|GuideToPharmacology
THRB	THRB	PRIMAQUINE PHOSPHATE		DTC
THRB	THRB	TOLONIUM CHLORIDE		DTC
THRB	THRB	METHYLTHIONINIUM CHLORIDE		DTC
THRB	THRB	LIOTHYRONINE	agonist	TdgClinicalTrial|ChemblInteractions|TEND
THRB	THRB	DEXTROTHYROXINE SODIUM	agonist	ChemblInteractions
THRB	THRB	CHEMBL602969		DTC
THRB	THRB	ORTHO-PHTHALALDEHYDE		DTC
THRB	THRB	ZIRAM		DTC
THRB	THRB	DAUNORUBICIN		DTC
THRB	THRB	ISOTRETINOIN		DTC
THRB	THRB	RHAMNETIN		DTC
THRB	THRB	OXYTETRACYCLINE		DTC
THRB	THRB	LIOTHYRONINE SODIUM	agonist	ChemblInteractions|GuideToPharmacology
THRB	THRB	EPROTIROME	agonist	TdgClinicalTrial|ChemblInteractions
THRB	THRB	CHEMBL601952		DTC
THRB	THRB	CHEMBL426404		DTC
THRB	THRB	SLAZINIC ACID		DTC
THRB	THRB	CHEMBL1493528		DTC
THRB	THRB	DITHIAZANINE IODIDE		DTC

THRB	THRB	ROSMARINIC ACID		DTC
THRB	THRB	QUINALDINE BLUE		DTC
THRB	THRB	CHEMBL1341830		DTC
THRB	THRB	CIRAZOLINE		DTC
THRB	THRB	PYRACLOSTROBIN		DTC
THRB	THRB	METHYL PARATHIONE		DTC
THRB	THRB	QUININE		DTC
THRB	THRB	BENZO(K)FLUORANTHENE		DTC
THRB	THRB	DIENOCHLOR		DTC
THRB	THRB	RESMETIROM	agonist	TdgClinicalTrial|ChemblInteractions|TTD
THRB	THRB	DIPYRIDAMOLE		DTC
THRB	THRB	GOSSYPOL		DTC
THRB	THRB	CHEMBL584508		DTC
THRB	THRB	METHYLENE BLUE		DTC
THRB	THRB	WORTMANNIN		DTC
THRB	THRB	CHEMBL1465049		DTC
THRB	THRB	TRIAMTERENE		DTC
THRB	THRB	AZORUBINE		DTC
THRB	THRB	CHEMBL578741		DTC
THRB	THRB	QUINALIZARIN		DTC
THRB	THRB	LEVOTHYROXINE SODIUM	agonist	ChemblInteractions|GuideToPharmacology
THRB	THRB	3R14S-OCHRATOXIN A		DTC
THRB	THRB	CLOSANTEL		DTC
THRB	THRB	CHEMBL429095		DTC
THRB	THRB	DEPHOSTATIN		DTC
THRB	THRB	FUMARPROTOCETRARIC ACID		DTC
THRB	THRB	CHEMBL592124		DTC
THRB	THRB	BENZO[B]FLUORANTHENE		DTC
THRB	THRB	CHEMBL211670		DTC
THRB	THRB	QUINPIROLE		DTC
THRB	THRB	4-METHOXYCINNAMIC ACID		DTC
THRB	THRB	CHEMBL87568		DTC
THRB	THRB	AXITIROME		TTD
THRB	THRB	CHLOROPHENYLPIPERAZINE		DTC
THRB	THRB	CHEMBL577415		DTC
THRB	THRB	OXYCARBOXIN		DTC
THRB	THRB	SPIRONOLACTONE		DTC
THRB	THRB	TETRAIDOFLUORESCEIN		DTC
THRB	THRB	HOMIDIUM BROMIDE		DTC
THRB	THRB	INAMRINONE		DTC
THRB	THRB	METHYLENE BLUE		DTC
THRB	THRB	CHEMBL1451216		DTC
THRB	THRB	CHEMBL533293		DTC
THRB	THRB	ANAZOLENE		DTC
THRB	THRB	CARUBICIN		DTC
THRB	THRB	PICEATANNOL		DTC
THRB	THRB	DIBENZ[A,H]ANTHRACENE		DTC
THRB	THRB	IDARUBICIN		DTC
THRB	THRB	HOMIDIUM		DTC
THRB	THRB	ACID BLUE 129		DTC
THRB	THRB	DYCLONINE HYDROCHLORIDE		DTC
THRB	THRB	BAICALEIN		DTC
THRB	THRB	CHEMBL603024		DTC
THRB	THRB	JUGLONE		DTC
THRB	THRB	METHACYCLINE HYDROCHLORIDE		DTC
THRB	THRB	DACTINOMYCIN		DTC
THRB	THRB	DAUNORUBICIN HYDROCHLORIDE		DTC
THRB	THRB	GENTIAN VIOLET		DTC
THRB	THRB	HEXACHLOROPHENE		DTC
THRB	THRB	LOMOFUNGIN		DTC
THRB	THRB	OXIDOPAMINE		DTC

THRB	THRB	CHLOROTHALONIL		DTC
PLK2	PLK2	ONVANSERTIB	inhibitor	GuideToPharmacology
PLK2	PLK2	BI-2536	inhibitor	GuideToPharmacology
PLK2	PLK2	WORTMANNIN		DTC
P2RY1	P2RY1	ASPIRIN		PharmGKB
P2RY1	P2RY1	ADENOSINE DIPHOSPHATE	agonist	GuideToPharmacology
P2RY1	P2RY1	SURAMIN	antagonist	GuideToPharmacology
P2RY1	P2RY1	ADENOSINE TRIPHOSPHATE	agonist|antagonist	GuideToPharmacology
P2RY1	P2RY1	ISO-IANTHERAN A		DTC
PPP1R18	PPP1R18	CARBOPLATIN		PharmGKB
PPP1R18	PPP1R18	GEMCITABINE		PharmGKB
HOMER1	HOMER1	LEVODOPA		PharmGKB
HOMER1	HOMER1	HALOPERIDOL		PharmGKB
JAK3	JAK3	AT-9283	inhibitor	TALC|ChemblInteractions|GuideToPharmacology
JAK3	JAK3	CERDULATINIB	inhibitor	ChemblInteractions|GuideToPharmacology|TTD
JAK3	JAK3	PACRITINIB	inhibitor	GuideToPharmacology
JAK3	JAK3	R-406		DTC
JAK3	JAK3	CHEMBL1997335		DTC
JAK3	JAK3	NS-018	inhibitor	GuideToPharmacology
JAK3	JAK3	SOLCITINIB	inhibitor	GuideToPharmacology
JAK3	JAK3	LAUROGUADINE		DTC
JAK3	JAK3	PF-00562271		DTC
JAK3	JAK3	GO-6976		DTC
JAK3	JAK3	RG-1530		DTC
JAK3	JAK3	PEFICITINIB	inhibitor	TdgClinicalTrial|ChemblInteractions|GuideToPharmacology|TTD
JAK3	JAK3	ACALABRUTINIB	inhibitor	GuideToPharmacology
JAK3	JAK3	OCLACITINIB	inhibitor	GuideToPharmacology
JAK3	JAK3	RUXOLITINIB	inhibitor	DTC|JAX-CKB|GuideToPharmacology
JAK3	JAK3	BREPOCITINIB	inhibitor	GuideToPharmacology
JAK3	JAK3	TOFACITINIB	inhibitor	DTC|JAX-CKB|CIViC|GuideToPharmacology|TTD
JAK3	JAK3	IBRUTINIB	inhibitor	GuideToPharmacology
JAK3	JAK3	UPADACITINIB	inhibitor	GuideToPharmacology
JAK3	JAK3	CYC-116		DTC
JAK3	JAK3	R-333	inhibitor	ChemblInteractions
JAK3	JAK3	CENISERTIB		DTC
JAK3	JAK3	AZD-1480	inhibitor	GuideToPharmacology
JAK3	JAK3	MOMELOTINIB	inhibitor	GuideToPharmacology
JAK3	JAK3	TOFACITINIB CITRATE	inhibitor	ChemblInteractions
JAK3	JAK3	DECERNOTINIB	inhibitor	TdgClinicalTrial|ChemblInteractions|GuideToPharmacology|TTD
JAK3	JAK3	CHEMBL379975		DTC
JAK3	JAK3	POSELTINIB	inhibitor	GuideToPharmacology
JAK3	JAK3	JNJ-7706621		DTC
JAK3	JAK3	GW843682X		DTC
JAK3	JAK3	GANDOTINIB	inhibitor	GuideToPharmacology
JAK3	JAK3	TRICETAMIDE	inhibitor	ChemblInteractions
JAK3	JAK3	FEDRATINIB	inhibitor	GuideToPharmacology
JAK3	JAK3	BMS-911543	inhibitor	GuideToPharmacology
JAK3	JAK3	WHI-P131	inhibitor	ChemblInteractions
JAK3	JAK3	ENTRECTINIB		DTC
JAK3	JAK3	R-348	inhibitor	ChemblInteractions
JAK3	JAK3	XELJANZ	inhibitor	MyCancerGenome
JAK3	JAK3	PF-06651600	inhibitor	TTD
JAK3	JAK3	ABIVERTINIB	inhibitor	GuideToPharmacology
JAK3	JAK3	DELGOCITINIB	inhibitor	GuideToPharmacology
JAK3	JAK3	XL-019	inhibitor	GuideToPharmacology
JAK3	JAK3	TAE-684		DTC
JAK3	JAK3	ADAVOSERTIB		DTC
JAK3	JAK3	SOTRASTAURIN		DTC
JAK3	JAK3	PD-0166285		DTC
JAK3	JAK3	CHEMBL225519		DTC
JAK3	JAK3	TASOCITINIB		TdgClinicalTrial

JAK3	JAK3	BARICITINIB	inhibitor	GuideToPharmacology
JAK3	JAK3	CHEMBL2062804	inhibitor	GuideToPharmacology
JAK3	JAK3	FILGOTINIB	inhibitor	GuideToPharmacology
JAK3	JAK3	ATEZOLIZUMAB		CIViC
PTP4A3	PTP4A3	CETUXIMAB		CIViC
PTP4A3	PTP4A3	EMODIN		DTC
CD2	CD2	ALEFACEPT	inhibitor	TdgClinicalTrial|ChemblInteractions|TEND|GuideToPharmacology
CD2	CD2	SIPLIZUMAB	inhibitor	TdgClinicalTrial|ChemblInteractions|TTD
CD2	CD2	ALDESLEUKIN		NCI
LDHA	LDHA	CHEMBL2058994		DTC
SLC36A2	SLC36A2	OXITRIPTAN	inhibitor	GuideToPharmacology
PROZ	PROZ	MENADIONE	activator	TEND
PPP1R1A	PPP1R1A	INSULIN		NCI
SLC5A2	SLC5A2	CANAGLIFLOZIN	inhibitor	TdgClinicalTrial|GuideToPharmacology
SLC5A2	SLC5A2	EMPAGLIFLOZIN	antagonist|inhibitor	TdgClinicalTrial|ChemblInteractions|GuideToPharmacology
SLC5A2	SLC5A2	ERTUGLIFLOZIN	antagonist|inhibitor	ChemblInteractions|GuideToPharmacology
SLC5A2	SLC5A2	IPRAGLIFLOZIN	inhibitor	TdgClinicalTrial|GuideToPharmacology
SLC5A2	SLC5A2	SOTAGLIFLOZIN	inhibitor	ChemblInteractions|GuideToPharmacology
SLC5A2	SLC5A2	TOFOGLIFLOZIN	inhibitor	ChemblInteractions|GuideToPharmacology
SLC5A2	SLC5A2	DAPAGLIFLOZIN	antagonist|inhibitor	TdgClinicalTrial|GuideToPharmacology
SLC5A2	SLC5A2	REMOGLIFLOZIN ETABONATE	inhibitor	ChemblInteractions
SLC5A2	SLC5A2	LICOGLIFLOZIN	inhibitor	GuideToPharmacology
SLC5A2	SLC5A2	REMOGLIFLOZIN	inhibitor	GuideToPharmacology
SLC5A2	SLC5A2	BEXAGLIFLOZIN	inhibitor	ChemblInteractions
SLC5A2	SLC5A2	CANAGLIFLOZIN	inhibitor	ChemblInteractions
SLC5A2	SLC5A2	DAPAGLIFLOZIN PROPYLENE GLYCOL	inhibitor	ChemblInteractions
SLC5A2	SLC5A2	GLUFOSFAMIDE		TdgClinicalTrial
SLC5A2	SLC5A2	SERGLIFLOZIN ETABONATE	inhibitor	GuideToPharmacology
CXCR3	CXCR3	DIOSCIN	antagonist	GuideToPharmacology
ITGAL	ITGAL	CASEARINONE B		DTC
ITGAL	ITGAL	BUSULFAN		NCI
ITGAL	ITGAL	LIFITEGRAST	antagonist	ChemblInteractions|GuideToPharmacology|TTD
ITGAL	ITGAL	EFALIZUMAB	inhibitor|antibody	TdgClinicalTrial|ChemblInteractions|TEND|GuideToPharmacology|TTD
ITGAL	ITGAL	FLUOROURACIL		NCI
ITGAL	ITGAL	CASEARINOL B		DTC
ITGAL	ITGAL	EPOETIN ALFA		NCI
ITGAL	ITGAL	ETOPOSIDE		NCI
ITGAL	ITGAL	CASEARINONE A		DTC
ITGAL	ITGAL	ROVELIZUMAB	antibody|antagonist	ChemblInteractions|GuideToPharmacology
ITGAL	ITGAL	ODULIMOMAB	inhibitor	ChemblInteractions
ITGAL	ITGAL	CYCLOSPORINE		NCI
ITGAL	ITGAL	THROMBIN		NCI
ITGAL	ITGAL	CYCLOPHOSPHAMIDE		NCI
ITGAL	ITGAL	STAUROSPORINE		NCI
ITGAL	ITGAL	MYCOPHENOLATE MOFETIL		NCI
ITGAL	ITGAL	SIROLIMUS		NCI
CBLC	CBLC	OLAPARIB		JAX-CKB
CYP4F2	CYP4F2	CLOFIBRATE		PharmGKB
CYP4F2	CYP4F2	KETOCONAZOLE		PharmGKB
CYP4F2	CYP4F2	CLOPIDOGREL		PharmGKB
CYP4F2	CYP4F2	PHENPROCOUMON		PharmGKB
CYP4F2	CYP4F2	ACENOCOUMAROL		PharmGKB
CYP4F2	CYP4F2	LOVASTATIN		PharmGKB
CYP4F2	CYP4F2	ASPIRIN		PharmGKB
CYP4F2	CYP4F2	PAFURAMIDINE MALEATE		PharmGKB
ADH1B	ADH1B	ACETALDEHYDE		PharmGKB
ADH1B	ADH1B	FOMEPIZOLE	inhibitor	TdgClinicalTrial|ChemblInteractions|TEND
ADH1B	ADH1B	ALCOHOL		PharmGKB
ADH1B	ADH1B	ABACAVIR		PharmGKB
NR3C2	NR3C2	PROGESTERONE	agonist|antagonist	TdgClinicalTrial|GuideToPharmacology
NR3C2	NR3C2	DESOXYCORTICOSTERONE PIVALATE	agonist	ChemblInteractions

NR3C2	NR3C2	FELODIPINE	antagonist	ChemblInteractions
NR3C2	NR3C2	FINERENONE	antagonist	ChemblInteractions|GuideToPharmacology|TTD
NR3C2	NR3C2	ONAPRISTONE	antagonist	GuideToPharmacology
NR3C2	NR3C2	FLUDROCORTISONE	agonist	TdgClinicalTrial|TEND|GuideToPharmacology|TTD
NR3C2	NR3C2	XL550	antagonist	ChemblInteractions|TTD
NR3C2	NR3C2	HYDROCORTISONE	agonist	GuideToPharmacology
NR3C2	NR3C2	BREMELANOTIDE		TTD
NR3C2	NR3C2	FLUDROCORTISONE ACETATE	agonist	ChemblInteractions
NR3C2	NR3C2	DROSPIRENONE	antagonist	TdgClinicalTrial|ChemblInteractions|GuideToPharmacology|TTD
NR3C2	NR3C2	EPLERENONE	antagonist	TdgClinicalTrial|ChemblInteractions|TEND|GuideToPharmacology|TTD
NR3C2	NR3C2	PF-03882845	antagonist	ChemblInteractions|GuideToPharmacology
NR3C2	NR3C2	SPIRONOLACTONE	antagonist	TdgClinicalTrial|ChemblInteractions|TEND|GuideToPharmacology|TTD
NR3C2	NR3C2	PREDNISOLONE	agonist	GuideToPharmacology
NR3C2	NR3C2	NIMODIPINE	antagonist	ChemblInteractions|GuideToPharmacology
NR3C2	NR3C2	LY2623091	antagonist	ChemblInteractions|TTD
NR3C2	NR3C2	ENALAPRIL		PharmGKB
NR3C2	NR3C2	ALDOSTERONE	agonist	GuideToPharmacology|TTD
NR3C2	NR3C2	MT-3995	antagonist	ChemblInteractions|TTD
NR3C2	NR3C2	DEXAMETHASONE	agonist	GuideToPharmacology
NR3C2	NR3C2	DESOXYCORTICOSTERONE ACETATE	agonist	ChemblInteractions
NR3C2	NR3C2	BUDESONIDE	agonist	GuideToPharmacology
NCF4	NCF4	DOXORUBICIN		PharmGKB
NCF4	NCF4	IDARUBICIN		PharmGKB
NTNG1	NTNG1	ALLOPURINOL		PharmGKB
FABP1	FABP1	FENOFIBRATE		PharmGKB
FABP1	FABP1	DEXAMETHASONE		NCI
FABP1	FABP1	CLOFIBRATE		NCI
LOXL2	LOXL2	SIMTUZUMAB	inhibitor	ChemblInteractions|GuideToPharmacology|TTD
DPEP1	DPEP1	CILASTATIN	inhibitor	TdgClinicalTrial|TEND|TTD
DPEP1	DPEP1	CILASTATIN SODIUM	inhibitor	ChemblInteractions
DPEP1	DPEP1	DEXAMETHASONE		NCI
VWF	VWF	EGAPTIVON PEGOL		TdgClinicalTrial
VWF	VWF	MITOMYCIN		NCI
VWF	VWF	THALIDOMIDE		NCI
VWF	VWF	HEPARIN		NCI
VWF	VWF	O-CHLOROACETYLCARBAMOYLFUMAGILLOL		NCI
VWF	VWF	VINCRISTINE		NCI
VWF	VWF	ACETYLCYSTEINE		NCI
VWF	VWF	PENTOXIFYLLINE		NCI
VWF	VWF	CAPLACIZUMAB	inhibitor	TdgClinicalTrial|ChemblInteractions|TTD
VWF	VWF	PHENYLEPHRINE		NCI
VWF	VWF	STREPTOZOCIN		NCI
VWF	VWF	WARFARIN		NCI
VWF	VWF	PREDNISONE		NCI
VWF	VWF	RIBAVIRIN		NCI
GLDC	GLDC	ESCITALOPRAM		PharmGKB
GLDC	GLDC	CITALOPRAM		PharmGKB
POU5F1	POU5F1	PHENYTOIN		PharmGKB
POU5F1	POU5F1	ALCOHOL		NCI
POU5F1	POU5F1	ALLOPURINOL		PharmGKB
POU5F1	POU5F1	CARBAMAZEPINE		PharmGKB
CASP1	CASP1	BELNACASAN		TdgClinicalTrial|TTD
CASP1	CASP1	NIVOCASAN	inhibitor	ChemblInteractions|TTD
CASP1	CASP1	BERKELEYDIONE		DTC
CASP1	CASP1	PRALNACASAN	inhibitor	GuideToPharmacology|TTD
CASP1	CASP1	DIACEREIN		TTD
CASP1	CASP1	VERMISTATIN		DTC
CASP1	CASP1	BERKELEYACETAL C		DTC
CASP1	CASP1	CHEMBL415893		DTC
CASP1	CASP1	4-CHLOROMERCURIBENZOIC ACID		DTC
CASP1	CASP1	BERKELEYTRIONE		DTC

CASP1	CASP1	ISOBOLDINE		DTC
CASP1	CASP1	JUGLONE		DTC
CASP1	CASP1	CHEMBL429095		DTC
CASP1	CASP1	MESALAMINE		DTC
CASP1	CASP1	EMRICASAN	inhibitor	ChemblInteractions
CASP1	CASP1	BERKELEYAMIDE C		DTC
CASP1	CASP1	AMMONIUM TRICHLOROTELLURATE	inhibitor	GuideToPharmacology
CASP1	CASP1	CHEMBL580421		DTC
CASP1	CASP1	GOSSYPOL		DTC
CASP1	CASP1	BERKELEYAMIDE B		DTC
CASP1	CASP1	CHEMBL337173		DTC
CASP1	CASP1	BERKELEYACETAL B		DTC
CASP1	CASP1	BERKELEYACETAL A		DTC
CASP1	CASP1	CHEMBL578512		DTC
PADI2	PADI2	CHEMBL1910970		DTC
PADI2	PADI2	STREPTONIGRIN		DTC
PADI2	PADI2	CHEMBL1910971		DTC
CSPG4	CSPG4	ALTEPLASE		NCI
TRIB3	TRIB3	METHYLPHENIDATE		PharmGKB
GJC1	GJC1	CARBENOXOLONE	inhibitor	GuideToPharmacology
GJC1	GJC1	OCTANOL	inhibitor	GuideToPharmacology
DNASE1L3	DNASE1L3	IMG-7289		TTD
SLC29A2	SLC29A2	GEMCITABINE		PharmGKB
SLC29A2	SLC29A2	TECADENOSON		DTC
SLC29A2	SLC29A2	MERCAPTOPURINE		NCI
CD86	CD86	PAMIDRONIC ACID		NCI
CD86	CD86	ABATACEPT	antagonist|inhibitor	TdgClinicalTrial|ChemblInteractions|TEND|GuideToPharmacology|TTD
CD86	CD86	INDOMETHACIN		NCI
CD86	CD86	BELATACEPT	antagonist|inhibitor	TdgClinicalTrial|ChemblInteractions|GuideToPharmacology
CD86	CD86	TERFENADINE		NCI
CD86	CD86	ROXITHROMYCIN		NCI
CD86	CD86	DEXAMETHASONE		NCI
ITGA5	ITGA5	VOLOCIXIMAB	antibody|antagonist	TALC|ChemblInteractions|GuideToPharmacology
ITGA5	ITGA5	PF-04605412	antibody	TALC
ITGA5	ITGA5	GLPG-0187		TdgClinicalTrial
ITGA5	ITGA5	ETARACIZUMAB	antibody	TALC
ITGA5	ITGA5	CILMOSTIM		NCI
ITGA5	ITGA5	DIMETHYL SULFOXIDE		NCI
ITGA5	ITGA5	CILENGITIDE		TdgClinicalTrial
CALCA	CALCA	MINOXIDIL		TTD
CALCA	CALCA	DALFAMPRIDINE		TTD
CALCA	CALCA	PINACIDIL		TTD
CALCA	CALCA	AZD7009		TTD
CALCA	CALCA	DOFETILIDE		TTD
CALCA	CALCA	CHEMBL1563246		TTD
CALCA	CALCA	BIMAKALIM		TTD
CALCA	CALCA	IBUTILIDE		TTD
CALCA	CALCA	NATEGLINIDE		TTD
CALCA	CALCA	DIAZOXIDE		TTD
CALCA	CALCA	EPTINEZUMAB	antibody|inhibitor	ChemblInteractions
CALCA	CALCA	AMIODARONE		TTD
CALCA	CALCA	BUDIODARONE		TTD
CALCA	CALCA	CHEMBL42661		TTD
CALCA	CALCA	RANITIDINE		NCI
CALCA	CALCA	ANDOLAST		TTD
CALCA	CALCA	PREDNISONE		NCI
CALCA	CALCA	AMIFAMPRIDINE		TTD
CALCA	CALCA	AZIMILIDE		TTD
CALCA	CALCA	DRONEDARONE		TTD
CALCA	CALCA	CIZOLIRTINE		TTD
CD84	CD84	ETANERCEPT		PharmGKB

DEPTOR	DEPTOR	OSI-027	inhibitor	MyCancerGenome
DEPTOR	DEPTOR	AZD-8055	inhibitor	MyCancerGenome
DEPTOR	DEPTOR	SAPANISERTIB	inhibitor	MyCancerGenome
CD3D	CD3D	FORALUMAB	inhibitor	TdgClinicalTrial|ChemblInteractions
CD3D	CD3D	MUROMONAB-CD3	inhibitor	TdgClinicalTrial|ChemblInteractions
CD3D	CD3D	BLINATUMOMAB	activator	TdgClinicalTrial|ChemblInteractions
CD3D	CD3D	CATUMAXOMAB		TdgClinicalTrial
CD3D	CD3D	SOLITOMAB		TdgClinicalTrial
CD3D	CD3D	OTELIXIZUMAB		TdgClinicalTrial
CD3D	CD3D	VISILIZUMAB		TdgClinicalTrial
PGF	PGF	TB-403		TdgClinicalTrial|TTD
PGF	PGF	AFLIBERCEPT	binder|inhibitor	ChemblInteractions|TTD
PGF	PGF	CONBERCEPT	inhibitor	ChemblInteractions
PTH1R	PTH1R	9-AMINOCAMPTOTHECIN		DTC
PTH1R	PTH1R	TERIPARATIDE	binder|agonist	TdgClinicalTrial|ChemblInteractions|TEND|GuideToPharmacology
PTH1R	PTH1R	DEXAMETHASONE		NCI
PTH1R	PTH1R	CHEMBL586870		DTC
PTH1R	PTH1R	CHEMBL599896		DTC
PTH1R	PTH1R	PARATHYROID HORMONE	agonist	TdgClinicalTrial|ChemblInteractions
PTH1R	PTH1R	TERIPARATIDE ACETATE	agonist	ChemblInteractions
PTH1R	PTH1R	PCO-371		TTD
PTH1R	PTH1R	ABALOPARATIDE	ligand|agonist	GuideToPharmacology|TTD
TNFSF13B	TNFSF13B	BLISIBIMOD	inhibitor	TdgClinicalTrial|ChemblInteractions|TTD
TNFSF13B	TNFSF13B	TABALUMAB	inhibitor	ChemblInteractions|TTD
TNFSF13B	TNFSF13B	BELIMUMAB	antibody|inhibitor|modulator	MyCancerGenome|TdgClinicalTrial|ChemblInteractions|PharmGKB|TTD
TNFSF13B	TNFSF13B	ATACICEPT	inhibitor	TdgClinicalTrial|ChemblInteractions
TNFSF13B	TNFSF13B	ETOPOSIDE		NCI
PYGL	PYGL	DEXAMETHASONE		PharmGKB
PYGL	PYGL	METHOTREXATE		PharmGKB
PYGL	PYGL	ASPARAGINASE		PharmGKB
CYP2J2	CYP2J2	TERFENADINE	inhibitor	GuideToPharmacology|PharmGKB
CYP2J2	CYP2J2	NABUMETONE		PharmGKB
CYP2J2	CYP2J2	THIORIDAZINE		PharmGKB
CYP2J2	CYP2J2	DICLOFENAC		PharmGKB
CYP2J2	CYP2J2	ASTEMIZOLE		PharmGKB
CYP2J2	CYP2J2	ALBENDAZOLE		PharmGKB
CYP2J2	CYP2J2	DANAZOL		PharmGKB
CYP2J2	CYP2J2	MESORIDAZINE		PharmGKB
CYP2J2	CYP2J2	TACROLIMUS		PharmGKB
CYP2J2	CYP2J2	AMIODARONE		PharmGKB
SLC14A2	SLC14A2	NIFEDIPINE		PharmGKB
RAPGEF3	RAPGEF3	CRYPTOTANSHINONE		DTC
RAPGEF3	RAPGEF3	CHEMBL1256796		DTC
RAPGEF3	RAPGEF3	HEXACHLOROPHENE		DTC
RAPGEF3	RAPGEF3	LUTEOLIN		DTC
RAPGEF3	RAPGEF3	CHEMBL1210769		DTC
RAPGEF3	RAPGEF3	IDARUBICIN HYDROCHLORIDE		DTC
RAPGEF3	RAPGEF3	CHEMBL234338		DTC
RAPGEF3	RAPGEF3	2,4-DIHYDROXYBENZOPHENONE		DTC
RAPGEF3	RAPGEF3	9,10-PHENANTHRENEQUINONE		DTC
RAPGEF3	RAPGEF3	RAFOXANIDE		DTC
RAPGEF3	RAPGEF3	TANSHINONE I		DTC
RAPGEF3	RAPGEF3	BITHIONOLOXIDE		DTC
RAPGEF3	RAPGEF3	DAPHNORETIN		DTC
RAPGEF3	RAPGEF3	ACRIFLAVINE		DTC
RAPGEF3	RAPGEF3	CHEMBL601757		DTC
RAPGEF3	RAPGEF3	PENTABROMOPHENOL		DTC
RAPGEF3	RAPGEF3	SENNOSIDE B		DTC
RAPGEF3	RAPGEF3	CHEMBL1576310		DTC
RAPGEF3	RAPGEF3	PHYSODIC ACID		DTC
RAPGEF3	RAPGEF3	CHEMBL585591		DTC

RAPGEF3	RAPGEF3	PYROGALLOL RED		DTC
RAPGEF3	RAPGEF3	LOBARIC ACID		DTC
RAPGEF3	RAPGEF3	GYROPHORIC ACID		DTC
RAPGEF3	RAPGEF3	ACID BLUE 129		DTC
RAPGEF3	RAPGEF3	CHEMBL515252		DTC
RAPGEF3	RAPGEF3	CHEMBL601385		DTC
RAPGEF3	RAPGEF3	CHEMBL505670		DTC
RAPGEF3	RAPGEF3	PURPUROGALLIN		DTC
RAPGEF3	RAPGEF3	1,4-DIMETHOXYANTHRAQUINONE		DTC
RAPGEF3	RAPGEF3	DIPYRIDAMOLE		DTC
RAPGEF3	RAPGEF3	CHEMBL1306556		DTC
RAPGEF3	RAPGEF3	MYRICETIN		DTC
RAPGEF3	RAPGEF3	ESTROPIPATE		DTC
ADA	ADA	PENTOSTATIN	inhibitor	TdgClinicalTrial|ChemblInteractions|TEND|GuideToPharmacology|TTD
ADA	ADA	DIPYRIDAMOLE	inhibitor	TdgClinicalTrial|TEND
ADA	ADA	FLUDARABINE		TTD
ADA	ADA	HYDROCORTISONE		NCI
ADA	ADA	CLADRIBINE		TTD
ADA	ADA	CORDYCEPIN		DTC
ADA	ADA	GARLIC		NCI
ADA	ADA	ELAPEGADEMASE		TTD
ADA	ADA	EHNA	inhibitor	GuideToPharmacology
ADA	ADA	METHOTREXATE		NCI
ADA	ADA	AMITRIPTYLINE		NCI
ADA	ADA	LEVODOPA		NCI
RAC2	RAC2	IDARUBICIN		PharmGKB
RAC2	RAC2	DOXORUBICIN		PharmGKB
ITGB6	ITGB6	STX-100	inhibitor	ChemblInteractions
ITGB6	ITGB6	INTETUMUMAB	antagonist	ChemblInteractions
ITGB6	ITGB6	GLPG-0187		TdgClinicalTrial
ITGB6	ITGB6	ABITUZUMAB	inhibitor	ChemblInteractions
FCGR2A	FCGR2A	INFLIXIMAB		PharmGKB
FCGR2A	FCGR2A	CETUXIMAB		PharmGKB
FCGR2A	FCGR2A	ADALIMUMAB		PharmGKB
FCGR2A	FCGR2A	RITUXIMAB		PharmGKB
FCGR2A	FCGR2A	VALZIFLOCEPT		TTD
FCGR2A	FCGR2A	ETANERCEPT	ligand	PharmGKB
FCGR2A	FCGR2A	TRASTUZUMAB		PharmGKB
DCN	DCN	SIROLIMUS		NCI
IL10RA	IL10RA	INTERLEUKIN-10	agonist	GuideToPharmacology
PVALB	PVALB	SODIUM CHLORIDE		NCI
COL4A3	COL4A3	COLLAGENASE CLOSTRIDIUM HISTOLYTICUM		ChemblInteractions
COL4A3	COL4A3	OCRIPLASMIN		ChemblInteractions
CCL4	CCL4	EPOETIN ALFA		NCI
CCL4	CCL4	CLODRONIC ACID		NCI
CYP27B1	CYP27B1	CHEMBL2036072		DTC
CYP27B1	CYP27B1	RIBAVIRIN		PharmGKB
CYP27B1	CYP27B1	DEFERASIROX		PharmGKB
CYP27B1	CYP27B1	CHEMBL255088	inhibitor	GuideToPharmacology
APLN	APLN	STAUROSPORINE		NCI
HLA-DQB1	HLA-DQB1	TICLOPIDINE		PharmGKB
HLA-DQB1	HLA-DQB1	CARBAMAZEPINE		PharmGKB
HLA-DQB1	HLA-DQB1	AMOXICILLIN		PharmGKB
HLA-DQB1	HLA-DQB1	FLOXACILLIN		PharmGKB
HLA-DQB1	HLA-DQB1	ACETAMINOPHEN		PharmGKB
HLA-DQB1	HLA-DQB1	CLAVULANIC ACID		PharmGKB
HLA-DQB1	HLA-DQB1	BUCILLAMINE		PharmGKB
HLA-DQB1	HLA-DQB1	FLUPIRTINE		PharmGKB
HLA-DQB1	HLA-DQB1	NEVIRAPINE		PharmGKB
HLA-DQB1	HLA-DQB1	LAMOTRIGINE		PharmGKB
HLA-DQB1	HLA-DQB1	LUMIRACOXIB		PharmGKB

HLA-DQB1	HLA-DQB1	ASPIRIN		PharmGKB
SLAMF7	SLAMF7	ELOTUZUMAB	antibody|inhibitor|modulator	MyCancerGenome|TdgClinicalTrial|ChemblInteractions|GuideToPharmacology
CCR5	CCR5	MARAVIROC	antagonist	DTC|TdgClinicalTrial|ChemblInteractions|TEND|GuideToPharmacology|PharmGKB|TTD
CCR5	CCR5	ANCRIVIROC	antagonist	GuideToPharmacology
CCR5	CCR5	LERONLIMAB	antibody|antagonist	ChemblInteractions|GuideToPharmacology|TTD
CCR5	CCR5	CHEMBL207004	antagonist	DTC|GuideToPharmacology|TTD
CCR5	CCR5	INCB-9471	antagonist	ChemblInteractions|TTD
CCR5	CCR5	CENICRIVIROC	antagonist	TdgClinicalTrial|ChemblInteractions|GuideToPharmacology|TTD
CCR5	CCR5	APLAVIROC HYDROCHLORIDE	antagonist	ChemblInteractions
CCR5	CCR5	CHEMBL41275	antagonist	GuideToPharmacology
CCR5	CCR5	VICRIVIROC MALEATE	antagonist	ChemblInteractions
CCR5	CCR5	PF-04634817	antagonist	ChemblInteractions
CCR5	CCR5	VICRIVIROC	antagonist	TdgClinicalTrial|GuideToPharmacology|TTD
CCR5	CCR5	AZD5672	antagonist	ChemblInteractions|GuideToPharmacology
CCR5	CCR5	CHEMBL2018969	antagonist	GuideToPharmacology
CCR5	CCR5	PF-232798		TTD
CCR5	CCR5	APLAVIROC	antagonist	GuideToPharmacology
CD4	CD4	PRILIXIMAB	negative modulator	ChemblInteractions
CD4	CD4	CLENOLIXIMAB	inhibitor	ChemblInteractions
CD4	CD4	TRX-1	inhibitor	ChemblInteractions
CD4	CD4	ZANOLIMUMAB		TdgClinicalTrial|ChemblInteractions|TTD
CD4	CD4	CEDELIZUMAB	inhibitor	ChemblInteractions
CD4	CD4	IBALIZUMAB	antagonist|inhibitor|antibody	ChemblInteractions|GuideToPharmacology|TTD
CD4	CD4	KELIXIMAB	negative modulator	ChemblInteractions
CD4	CD4	TREGALIZUMAB	agonist|antibody	TdgClinicalTrial|ChemblInteractions|GuideToPharmacology|TTD
CD4	CD4	HERBIMYCIN		NCI
HLA-DPB1	HLA-DPB1	CLOZAPINE		PharmGKB
HLA-DPB1	HLA-DPB1	ASPIRIN		PharmGKB
ABCC3	ABCC3	EMTRICITABINE		DTC
ABCC3	ABCC3	PACLITAXEL		CIViC
ABCC3	ABCC3	METHOTREXATE		PharmGKB
ABCC3	ABCC3	CEFOPERAZONE		DTC
ABCC3	ABCC3	DOXORUBICIN		DTC|PharmGKB
ABCC3	ABCC3	CEFACLOR		DTC
ABCC3	ABCC3	ABACAVIR		DTC
ABCC3	ABCC3	DELAVIRDINE		DTC
ABCC3	ABCC3	TENOFOVIR		DTC
ABCC3	ABCC3	CISPLATIN		PharmGKB
ABCC3	ABCC3	LAMIVUDINE		DTC|PharmGKB
ABCC3	ABCC3	VINCRISTINE		PharmGKB
ABCC3	ABCC3	CEPHALEXIN		DTC
ABCC3	ABCC3	CEFUROXIME		DTC
ABCC3	ABCC3	MORPHINE		PharmGKB
ABCC3	ABCC3	CEPHRADINE		DTC
ABCC3	ABCC3	CYCLOPHOSPHAMIDE		PharmGKB
ABCC3	ABCC3	CEFOXITIN		DTC
ABCC3	ABCC3	RIMONABANT		DTC
ABCC3	ABCC3	CEFMETAZOLE		DTC
ABCC3	ABCC3	CEFTRIAXONE		DTC
ABCC3	ABCC3	ETRAVIRINE		DTC
ABCC3	ABCC3	NEVIRAPINE		DTC
ABCC3	ABCC3	EFAVIRENZ		DTC
ABCC3	ABCC3	CEFAMANDOLE		DTC
PDK1	PDK1	CELECOXIB		PharmGKB
PDK1	PDK1	CHEMBL445304		DTC
PDK1	PDK1	AR-12	inhibitor	TdgClinicalTrial|ChemblInteractions
PDK1	PDK1	DICHLOROACETIC ACID	inhibitor	DTC
PDK1	PDK1	UCN-01	inhibitor	ChemblInteractions
PDK1	PDK1	CHEMBL257158		DTC
AQP3	AQP3	AUPHEN	inhibitor	GuideToPharmacology
BTC	BTC	PANITUMUMAB		PharmGKB

BTC	BTC	CETUXIMAB		PharmGKB
CD3E	CD3E	ERTUMAXOMAB		ChemblInteractions|TTD
CD3E	CD3E	OTELIXIZUMAB	antibody	TdgClinicalTrial|ChemblInteractions|GuideToPharmacology
CD3E	CD3E	BLINATUMOMAB	antibody	TdgClinicalTrial|ChemblInteractions|GuideToPharmacology
CD3E	CD3E	MUROMONAB-CD3	antibody|inhibitor|binder	TdgClinicalTrial|ChemblInteractions|TEND|GuideToPharmacology|TTD
CD3E	CD3E	TEPLIZUMAB		TdgClinicalTrial|ChemblInteractions
CD3E	CD3E	CATUMAXOMAB	agonist|antibody	TdgClinicalTrial|GuideToPharmacology
CD3E	CD3E	FORALUMAB	inhibitor	TdgClinicalTrial|ChemblInteractions
CD3E	CD3E	SOLITOMAB		TdgClinicalTrial
CD3E	CD3E	VISILIZUMAB	inhibitor	TdgClinicalTrial|ChemblInteractions
CD3E	CD3E	RESIMMUNE		TTD
CDH3	CDH3	PF-06671008		TTD
RRM2	RRM2	HYDROXYUREA	inhibitor	ChemblInteractions|GuideToPharmacology|TTD
RRM2	RRM2	FLUDARABINE PHOSPHATE	inhibitor	ChemblInteractions
RRM2	RRM2	CYTARABINE		PharmGKB
RRM2	RRM2	GALLIUM NITRATE	inhibitor	ChemblInteractions
RRM2	RRM2	GEMCITABINE	inhibitor	ClearityFoundationClinicalTrial|GuideToPharmacology|TTD
RRM2	RRM2	FLUDARABINE	inhibitor	GuideToPharmacology
RRM2	RRM2	LY-2334737		TTD
RRM2	RRM2	CLOFARABINE	inhibitor	ChemblInteractions|GuideToPharmacology
RRM2	RRM2	TRIAPINE		TdgClinicalTrial|TTD
RRM2	RRM2	GEMCITABINE HYDROCHLORIDE	inhibitor	ChemblInteractions
RRM2	RRM2	TEZACITABINE	inhibitor	ChemblInteractions
RRM2	RRM2	CLADRIBINE	inhibitor	PharmGKB
RRM2	RRM2	GEMCITABINE ELAIDATE		TTD
TOP2A	TOP2A	AMRUBICIN	inhibitor	ChemblInteractions
TOP2A	TOP2A	EPIRUBICIN	inhibitor	TdgClinicalTrial|TEND|PharmGKB
TOP2A	TOP2A	CHEMBL1773343		DTC
TOP2A	TOP2A	DOXORUBICIN	inhibitor	DTC|TdgClinicalTrial|ClearityFoundationClinicalTrial|TEND|GuideToPharmacology
TOP2A	TOP2A	BECATECARIN	inhibitor	TdgClinicalTrial|ChemblInteractions
TOP2A	TOP2A	VOSAROXIN	inhibitor	TdgClinicalTrial|ChemblInteractions
TOP2A	TOP2A	ELLIPTECINE		DTC
TOP2A	TOP2A	TENIPOSIDE	inhibitor	TdgClinicalTrial|ChemblInteractions|NCI|TEND
TOP2A	TOP2A	MAKALUVAMINE C TFA SALT		DTC
TOP2A	TOP2A	DAUNORUBICIN	inhibitor	DTC|TdgClinicalTrial|NCI
TOP2A	TOP2A	ADRIAMYCIN		NCI
TOP2A	TOP2A	C-1311	inhibitor	TdgClinicalTrial|ChemblInteractions
TOP2A	TOP2A	CARINATIN G		DTC
TOP2A	TOP2A	4'-O-ACETYLPATENTIFLORIN B		DTC
TOP2A	TOP2A	MITOXANTRONE	inhibitor	TdgClinicalTrial|NCI|TEND|GuideToPharmacology
TOP2A	TOP2A	DEXRAZOXANE	inhibitor	NCI
TOP2A	TOP2A	DAUNORUBICIN CITRATE	inhibitor	ChemblInteractions
TOP2A	TOP2A	ETOPOSIDE	inhibitor	DTC|TdgClinicalTrial|ChemblInteractions|NCI|TEND|GuideToPharmacology
TOP2A	TOP2A	BETULIN		DTC
TOP2A	TOP2A	CHEMBL2332127		DTC
TOP2A	TOP2A	IDARUBICIN	inhibitor	TdgClinicalTrial|TEND
TOP2A	TOP2A	KAEMPFERITRIN		DTC
TOP2A	TOP2A	CHEMBL244268		DTC
TOP2A	TOP2A	GANCOTAMAB	inhibitor	ChemblInteractions
TOP2A	TOP2A	PODOFILOX	inhibitor	TdgClinicalTrial|TEND
TOP2A	TOP2A	CHEMBL2171781		DTC
TOP2A	TOP2A	CHEMBL2171794		DTC
TOP2A	TOP2A	AMONAFIDE		DTC|TdgClinicalTrial
TOP2A	TOP2A	AMSACRINE	inhibitor	DTC|NCI
TOP2A	TOP2A	BERUBICIN HYDROCHLORIDE	inhibitor	ChemblInteractions
TOP2A	TOP2A	CHEMBL1080077		DTC
TOP2A	TOP2A	CAMPTOTHECIN		NCI
TOP2A	TOP2A	ELINAFIDE		DTC
TOP2A	TOP2A	CHEMBL2332126		DTC
TOP2A	TOP2A	VALRUBICIN	inhibitor	TdgClinicalTrial|ChemblInteractions|TEND
TOP2A	TOP2A	CHEMBL2332128		DTC

TOP2A	TOP2A	ETOPOSIDE PHOSPHATE	inhibitor	ChemblInteractions
TOP2A	TOP2A	CHEMBL2171812		DTC
TOP2A	TOP2A	MAKALUVAMINE A		DTC
TOP2A	TOP2A	MYRICETIN		DTC
TOP2A	TOP2A	DEMETHYLZEYLASTERONE		DTC
TOP2A	TOP2A	CHEMBL594695		DTC
TOP2A	TOP2A	IDRONOXIL		ClearityFoundationClinicalTrial
TOP2A	TOP2A	CHEMBL594379		DTC
TOP2A	TOP2A	CHEMBL593570		DTC
TOP2A	TOP2A	MAKALUVAMINE F		DTC
TOP2A	TOP2A	ALDOXORUBICIN	inhibitor	TdgClinicalTrial|ChemblInteractions
TOP2A	TOP2A	FRANGULIN B		DTC
TOP2A	TOP2A	CHEMBL2023733		DTC
TOP2A	TOP2A	IDARUBICIN HYDROCHLORIDE	inhibitor	ChemblInteractions
TOP2A	TOP2A	MAKALUVAMINE E		DTC
TOP2A	TOP2A	CHEMBL594153		DTC
TOP2A	TOP2A	PACLITAXEL		TdgClinicalTrial
TOP2A	TOP2A	CHEMBL594257		DTC
TOP2A	TOP2A	GENISTEIN		DTC
TOP2A	TOP2A	13-DEOXYDOXORUBICIN		TdgClinicalTrial
TOP2A	TOP2A	DIPHYLLIN		DTC
TOP2A	TOP2A	FLUOROURACIL		PharmGKB
TOP2A	TOP2A	LYCOBETAINE		DTC
TOP2A	TOP2A	VINCRISTINE		NCI
TOP2A	TOP2A	SECAUBRYOLIDE		DTC
TOP2A	TOP2A	MITOXANTRONE HYDROCHLORIDE	inhibitor	ChemblInteractions
TOP2A	TOP2A	TRICITRINOL B		DTC
TOP2A	TOP2A	AMRUBICIN HYDROCHLORIDE	inhibitor	ChemblInteractions
TOP2A	TOP2A	OLEANDEROLIDE		DTC
TOP2A	TOP2A	QUERCETIN		DTC
TOP2A	TOP2A	CHEMBL596082		DTC
TOP2A	TOP2A	HURATOXIN		DTC
TOP2A	TOP2A	HYDROQUINONE		NCI
TOP2A	TOP2A	SIMOCYCLINONE D8		DTC
TOP2A	TOP2A	DIAZIRINE		DTC
TOP2A	TOP2A	LUPEOL		DTC
TOP2A	TOP2A	CHEMBL507986		DTC
TOP2A	TOP2A	DOXORUBICIN HYDROCHLORIDE	inhibitor	ChemblInteractions
TOP2A	TOP2A	UNGEREMINE		DTC
TOP2A	TOP2A	FISETIN		DTC
TOP2A	TOP2A	DAUNORUBICIN HYDROCHLORIDE	inhibitor	ChemblInteractions
TOP2A	TOP2A	CHEMBL594259		DTC
TOP2A	TOP2A	CHEMBL607534		DTC
TOP2A	TOP2A	DIGITOXIN		DTC
CYP17A1	CYP17A1	ORTERONEL	inhibitor	TdgClinicalTrial|ChemblInteractions|CancerCommons|TTD
CYP17A1	CYP17A1	GALETERONE	inhibitor	TdgClinicalTrial|ChemblInteractions|GuideToPharmacology
CYP17A1	CYP17A1	ABIRATERONE	inhibitor	TdgClinicalTrial|CGI|GuideToPharmacology|PharmGKB|TTD
CYP17A1	CYP17A1	ABIRATERONE ACETATE	inhibitor	ChemblInteractions
CYP17A1	CYP17A1	CFG-920		TTD
CYP17A1	CYP17A1	ENZALUTAMIDE		CGI
CYP17A1	CYP17A1	SEVITERONEL		TTD
CYP17A1	CYP17A1	ZYTIGA	inhibitor	CancerCommons
BCL2A1	BCL2A1	OBATOCLAX MESYLATE	inhibitor	ChemblInteractions
EIF4EBP1	EIF4EBP1	PACLITAXEL		NCI
CD27	CD27	VARLILUMAB		TTD
SGK2	SGK2	ILORASERTIB		DTC
SGK2	SGK2	CHEMBL225519		DTC
SGK2	SGK2	PF-00562271		DTC
SGK2	SGK2	RG-1530		DTC
SGK2	SGK2	CENISERTIB		DTC
SGK2	SGK2	SB-220025		DTC

SGK2	SGK2	CYC-116		DTC
SGK2	SGK2	SOTRASTAURIN		DTC
SGK2	SGK2	GSK-269962A		DTC
SGK2	SGK2	SP-600125		DTC
SGK2	SGK2	DOVITINIB		DTC
SGK2	SGK2	AZD-1080		DTC
SGK2	SGK2	GW441756X		DTC
SGK2	SGK2	FASUDIL		DTC
DLL4	DLL4	NAVICIXIZUMAB		TTD
DLL4	DLL4	DEMCIZUMAB	inhibitor	TdgClinicalTrial|ChemblInteractions|TTD
DLL4	DLL4	ENOTICUMAB		TTD
ITGAM	ITGAM	DIMETHYL SULFOXIDE		NCI
ITGAM	ITGAM	PHENYLEPHRINE		NCI
ITGAM	ITGAM	LIAROZOLE		NCI
ITGAM	ITGAM	ROVELIZUMAB	antagonist	ChemblInteractions
ITGAM	ITGAM	CLARITHROMYCIN		NCI
ITGAM	ITGAM	HYDROCORTISONE		NCI
ITGAM	ITGAM	MORPHINE		NCI
ITGAM	ITGAM	ATORVASTATIN		NCI
ITGAM	ITGAM	FENTANYL		NCI
ITGAM	ITGAM	THEOPHYLLINE		NCI
ACKR3	ACKR3	PLERIXAFOR	agonist	GuideToPharmacology
HPGD	HPGD	CHEMBL601618		DTC
HPGD	HPGD	CHEMBL592106		DTC
HPGD	HPGD	LY-255283		DTC
HPGD	HPGD	4-CHLOROMERCURIBENZOIC ACID		DTC
HPGD	HPGD	HAEMATOXYLIN		DTC
HPGD	HPGD	TYRPHOSTIN 47		DTC
HPGD	HPGD	ALLITRIDIN		DTC
HPGD	HPGD	CHEMBL472656		DTC
HPGD	HPGD	LUTEOLIN		DTC
HPGD	HPGD	REV-5901		DTC
HPGD	HPGD	DICHLOROPHEN		DTC
HPGD	HPGD	BRIMONIDINE		DTC
HPGD	HPGD	THIMEROSAL		DTC
HPGD	HPGD	ZARDAVERINE		DTC
HPGD	HPGD	HYPERICIN		DTC
HPGD	HPGD	CHEMBL239439		DTC
HPGD	HPGD	MERCURIC CHLORIDE		DTC
HPGD	HPGD	CHEMBL220845		DTC
HPGD	HPGD	ZAPA		DTC
HPGD	HPGD	OBTUSAQUINONE		DTC
HPGD	HPGD	METHYL 7-DESHYDROXYPYROGALLIN-4-CARBOXYLATE		DTC
HPGD	HPGD	CHEMBL1481974		DTC
HPGD	HPGD	PHENAMIPHOS		DTC
HPGD	HPGD	CHEMBL581929		DTC
HPGD	HPGD	CHEMBL38508		DTC
HPGD	HPGD	ARACHIDONIC ACID		DTC
HPGD	HPGD	SAPPANONE A DIMETHYL ETHER		DTC
HPGD	HPGD	DEMETHYLENCECALIN		DTC
HPGD	HPGD	7-NITROINDAZOLE		DTC
HPGD	HPGD	CHEMBL502257		DTC
HPGD	HPGD	FPL-55712		DTC
HPGD	HPGD	TOLMETIN SODIUM		DTC
HPGD	HPGD	GOSSYPETIN		DTC
HPGD	HPGD	CHLORANIL		DTC
HPGD	HPGD	CHEMBL592124		DTC
HPGD	HPGD	CHEMBL242171		DTC
HPGD	HPGD	CHEMBL120806		DTC
HPGD	HPGD	GUTHION		DTC
HPGD	HPGD	NIFEDIPINE		DTC

HPGD	HPGD	CARNOSIC ACID	DTC
HPGD	HPGD	CHEMBL515505	DTC
HPGD	HPGD	CISPLATIN	DTC
HPGD	HPGD	SULFADIAZINE, SILVER	DTC
HPGD	HPGD	LEOIDIN	DTC
HPGD	HPGD	(S)-THIORPHAN	DTC
HPGD	HPGD	OXYBENZONE	DTC
HPGD	HPGD	MANGOSTIN	DTC
HPGD	HPGD	THIOTEPA	DTC
HPGD	HPGD	TANSHINONE IIA	DTC
HPGD	HPGD	PURPUROGALLIN	DTC
HPGD	HPGD	CHEMBL602807	DTC
HPGD	HPGD	QUINALIZARIN	DTC
HPGD	HPGD	BAY-11-7085	DTC
HPGD	HPGD	CHEMBL277148	DTC
HPGD	HPGD	HEXACHLOROPHENE	DTC
HPGD	HPGD	SULFASALAZINE	DTC
HPGD	HPGD	2',4'-DIHYDROXYCHALCONE	DTC
HPGD	HPGD	LECANORIC ACID	DTC
HPGD	HPGD	NBQX	DTC
HPGD	HPGD	CHEMBL523200	DTC
HPGD	HPGD	IRIGINOL HEXAACEATATE	DTC
HPGD	HPGD	ELLAGIC ACID	DTC
HPGD	HPGD	DIOXYBENZONE	DTC
HPGD	HPGD	APIGENIN	DTC
HPGD	HPGD	2-ARACHIDONOYLGLYCEROL	DTC
HPGD	HPGD	CHEMBL598663	DTC
HPGD	HPGD	LIOTHYRONINE SODIUM	DTC
HPGD	HPGD	BERGAPTOL	DTC
HPGD	HPGD	SOFALCONE	DTC
HPGD	HPGD	HOMIDIUM BROMIDE	DTC
HPGD	HPGD	TYRPHOSTIN 23	DTC
HPGD	HPGD	PROSTAGLANDIN A2	DTC
HPGD	HPGD	CARMINE	DTC
HPGD	HPGD	IODOPHTHALEIN	DTC
HPGD	HPGD	CURCUMIN	DTC
HPGD	HPGD	ATHRAQUINONES A	DTC
HPGD	HPGD	PURPURIN	DTC
HPGD	HPGD	CHEMBL73711	DTC
HPGD	HPGD	THIOGUANINE	DTC
HPGD	HPGD	DIETHYLTOLUAMIDE	DTC
HPGD	HPGD	CHEMBL531709	DTC
HPGD	HPGD	CHEMBL471005	DTC
HPGD	HPGD	CHEMBL244707	DTC
HPGD	HPGD	AURIN	DTC
HPGD	HPGD	CHEMBL516616	DTC
HPGD	HPGD	(4-CHLOROPHENYL)(MORPHOLINO)METHANETHIONE	DTC
HPGD	HPGD	KAEMPFEROL	DTC
HPGD	HPGD	STICTIC ACID	DTC
HPGD	HPGD	PENTABROMOPHENOL	DTC
HPGD	HPGD	IBUPROFEN	NCI
HPGD	HPGD	CHEMBL585269	DTC
HPGD	HPGD	JUGLONE	DTC
HPGD	HPGD	CEAROIN	DTC
HPGD	HPGD	BENZOTHIAZYL DISULFIDE	DTC
HPGD	HPGD	LORECLEZOLE	DTC
HPGD	HPGD	CHEMBL53898	DTC
HPGD	HPGD	PYROGALLOL	DTC
HPGD	HPGD	CHEMBL80155	DTC
HPGD	HPGD	CHEMBL67535	DTC
HPGD	HPGD	CHEMBL261123	DTC

HPGD	HPGD	CHEMBL1091971	DTC
HPGD	HPGD	DISULFIRAM	DTC
HPGD	HPGD	CHEMBL1465049	DTC
HPGD	HPGD	FLAVANONE	DTC
HPGD	HPGD	PLUMBAGIN	DTC
HPGD	HPGD	CHEMBL25230	DTC
HPGD	HPGD	DERRUBONE	DTC
HPGD	HPGD	ALPRAZOLAM	DTC
HPGD	HPGD	CHEMBL1459280	DTC
HPGD	HPGD	TPCK	DTC
HPGD	HPGD	CADMIUM DICHLORIDE	DTC
HPGD	HPGD	CHEMBL39878	DTC
HPGD	HPGD	CHEMBL607309	DTC
HPGD	HPGD	CHEMBL35482	DTC
HPGD	HPGD	CHEMBL585628	DTC
HPGD	HPGD	PYRITHIONE ZINC	DTC
HPGD	HPGD	PIPERINE	DTC
HPGD	HPGD	CHEMBL166486	DTC
HPGD	HPGD	CHEMBL395915	DTC
HPGD	HPGD	PAEONOL	DTC
HPGD	HPGD	NORSTICTIC ACID	DTC
HPGD	HPGD	EBSELEN	DTC
HPGD	HPGD	1,2-NAPHTHOQUINONE	DTC
HPGD	HPGD	PHOSMET	DTC
HPGD	HPGD	CHEMBL1368535	DTC
HPGD	HPGD	U-0126	DTC
HPGD	HPGD	PEUCENIN	DTC
HPGD	HPGD	7,8-DIHYDROXYFLAVONE	DTC
HPGD	HPGD	CHEMBL1255936	DTC
HPGD	HPGD	CHEMBL595227	DTC
HPGD	HPGD	FENTICLOR	DTC
HPGD	HPGD	MESALAMINE	DTC
HPGD	HPGD	EMBELIN	DTC
HPGD	HPGD	5,7-DIHYDROXYISOFLAVONE	DTC
HPGD	HPGD	CHLORPROPHAM	DTC
HPGD	HPGD	DIHYDREXIDINE HYDROCHLORIDE	DTC
HPGD	HPGD	CHEMBL222808	DTC
HPGD	HPGD	KETANSERIN	DTC
HPGD	HPGD	CHEMBL429095	DTC
HPGD	HPGD	FUMARPROTOCETRARIC ACID	DTC
HPGD	HPGD	CHEMBL1332182	DTC
HPGD	HPGD	EMODIC ACID	DTC
HPGD	HPGD	QUERCETIN	DTC
HPGD	HPGD	PHENYLMERCURIC ACETATE	DTC
HPGD	HPGD	HYDROQUINONE	DTC
HPGD	HPGD	FLUFENAMIC ACID	DTC
HPGD	HPGD	OXYTETRACYCLINE HYDROCHLORIDE	DTC
HPGD	HPGD	SODIUM CHLORIDE	NCI
HPGD	HPGD	CATECHOL	DTC
HPGD	HPGD	OXOGLAUCINE	DTC
HPGD	HPGD	GALLIC ACID	DTC
HPGD	HPGD	DANTHRON	DTC
HPGD	HPGD	TOLFENAMIC ACID	DTC
HPGD	HPGD	CHEMBL72365	DTC
HPGD	HPGD	AMENTOFLAVONE	DTC
HPGD	HPGD	LINOLEIC ACID	DTC
HPGD	HPGD	TRICLOSAN	DTC
HPGD	HPGD	ARACHIDONOYL GLYCINE	DTC
HPGD	HPGD	CHEMBL469309	DTC
HPGD	HPGD	THIRAM	DTC
HPGD	HPGD	TRANILAST	DTC

HPGD	HPGD	3,4-DIDESMETHYL-5-DESHYDROXY-3'-ETHOXYSCLEROIN	DTC
HPGD	HPGD	NORDIHYDROGUAIARETIC ACID	DTC
HPGD	HPGD	KHELLIN	DTC
HPGD	HPGD	E927A	DTC
HPGD	HPGD	2',BETA-DIHYDROXYCHALCONE	DTC
HPGD	HPGD	PHLORETIN	DTC
HPGD	HPGD	GOSSYPOL	DTC
HPGD	HPGD	PIRINIXIC ACID	DTC
HPGD	HPGD	CHEMBL1388908	DTC
HPGD	HPGD	SUPROFEN METHYL ESTER	DTC
HPGD	HPGD	DEPHOSTATIN	DTC
HPGD	HPGD	ZAPRINAST	DTC
HPGD	HPGD	KETANSERIN TARTRATE	DTC
HPGD	HPGD	CHEMBL56393	DTC
HPGD	HPGD	CAPTAN	DTC
HPGD	HPGD	CONGO RED	DTC
HPGD	HPGD	ALTANSERIN	DTC
HPGD	HPGD	BROMOENOL LACTONE	DTC
HPGD	HPGD	CHEMBL529138	DTC
HPGD	HPGD	DNQX	DTC
HPGD	HPGD	CHEMBL1214274	DTC
HPGD	HPGD	CHEMBL452954	DTC
HPGD	HPGD	FLAVONE	DTC
HPGD	HPGD	ACETYLSEROTONIN	DTC
HPGD	HPGD	2-MERCAPTOBENZOTHIAZOLE	DTC
HPGD	HPGD	BENZBROMARONE	DTC
HPGD	HPGD	SULFINPYRAZONE	DTC
HPGD	HPGD	CHEMBL222556	DTC
HPGD	HPGD	CHEMBL271023	DTC
HPGD	HPGD	PAPAVERINE HYDROCHLORIDE	DTC
HPGD	HPGD	2-BENZOYL-5-METHOXYBENZOQUINONE	DTC
HPGD	HPGD	CHEMBL421215	DTC
HPGD	HPGD	CARBIDOPA	DTC
HPGD	HPGD	IRIGENOL	DTC
HPGD	HPGD	MYRICETIN	DTC
HPGD	HPGD	CHEMBL50267	DTC
HPGD	HPGD	CHEMBL77387	DTC
HPGD	HPGD	DITHIOLETHIONE	DTC
HPGD	HPGD	FOSFONET	DTC
HPGD	HPGD	CAPSAZEPINE	DTC
HPGD	HPGD	CHEMBL1256291	DTC
HPGD	HPGD	MUNDULONE	DTC
HPGD	HPGD	BAICALEIN	DTC
HPGD	HPGD	HEMATOXYLIN	DTC
HPGD	HPGD	CHEMBL484663	DTC
HPGD	HPGD	NITRAZEPAM	DTC
HPGD	HPGD	METHYLDOPA (RACEMIC)	DTC
HPGD	HPGD	CHEMBL284609	DTC
HPGD	HPGD	SULFANITRAN	DTC
HPGD	HPGD	PENTACHLOROPHENOL	DTC
HPGD	HPGD	GW282974X	DTC
HPGD	HPGD	LUZINDOLE	DTC
HPGD	HPGD	TARENFLURBIL	DTC
HPGD	HPGD	ONONETIN	DTC
HPGD	HPGD	DIALLYLDISULFIDE	DTC
HPGD	HPGD	CHEMBL225513	DTC
HPGD	HPGD	AMBUNOL	DTC
HPGD	HPGD	CHEMBL1485420	DTC
HPGD	HPGD	MECYSTEINE HYDROCHLORIDE	DTC
HPGD	HPGD	CHEMBL68534	DTC
HPGD	HPGD	ZEARALENONE	DTC

HPGD	HPGD	BITHIONOL		DTC
HPGD	HPGD	3-HYDROXYCOUMARIN		DTC
HPGD	HPGD	KAINIC ACID		DTC
HPGD	HPGD	GENISTEIN		DTC
HPGD	HPGD	CLORGYLINE HYDROCHLORIDE		DTC
HPGD	HPGD	CHLOROTHALONIL		DTC
HPGD	HPGD	EPHEDRINE SULFATE		DTC
HPGD	HPGD	TRICLOFENOL		DTC
HPGD	HPGD	CHEMBL1214407		DTC
HPGD	HPGD	7-CHLOROKYNURENIC ACID		DTC
HPGD	HPGD	KOPARIN		DTC
HPGD	HPGD	DIHYDROXIDINE		DTC
HPGD	HPGD	CHEMBL242172		DTC
HPGD	HPGD	OMEPRAZOLE		DTC
HPGD	HPGD	3,4-DIMETHOXYDALBERGIONE		DTC
HPGD	HPGD	PYRROLIDINE DITHIOCARBAMIC ACID, AMMONIUM SALT		DTC
HPGD	HPGD	CLOROPHENE		DTC
HPGD	HPGD	PYRROLIDINE DITHIOCARBAMATE		DTC
HPGD	HPGD	3-METHOXYCATECHOL		DTC
ALDH1A2	ALDH1A2	RETINOL		NCI
ALDH1A2	ALDH1A2	HYDROCHLOROTHIAZIDE		PharmGKB
ALDH1A2	ALDH1A2	ATENOLOL		PharmGKB
CTSS	CTSS	ODANACATIB	inhibitor	GuideToPharmacology
CTSS	CTSS	PETESICATIB	inhibitor	GuideToPharmacology|TTD
CDK18	CDK18	AZD-5438	inhibitor	ChemblInteractions
CDK18	CDK18	PHA-793887	inhibitor	ChemblInteractions
CDK18	CDK18	RONICICLIB	inhibitor	ChemblInteractions
CDK18	CDK18	AT-7519	inhibitor	ChemblInteractions
CXCL10	CXCL10	ELDELUMAB		TdgClinicalTrial|TTD
CXCL10	CXCL10	RITONAVIR		NCI
CXCL10	CXCL10	NI-0801	inhibitor	ChemblInteractions|TTD
CXCL10	CXCL10	ATROPINE		NCI
CXCL10	CXCL10	ANTIBIOTIC		NCI
CXCL10	CXCL10	ATORVASTATIN		NCI
CXCL10	CXCL10	REGRAMOSTIM		NCI
CXCL10	CXCL10	METHYLPREDNISOLONE		NCI
CXCL10	CXCL10	OXALIPLATIN		NCI
CXCL10	CXCL10	TESTOSTERONE		NCI
CXCL10	CXCL10	STAVUDINE		NCI
CXCL10	CXCL10	ZIDOVUDINE		NCI
PDGFRA	PDGFRA	IMATINIB	inhibitor|antagonist	DTC|MyCancerGenome|TdgClinicalTrial|JAX-CKB|CGI|TEND|DoCM|COSMIC|CIViC|FDA|OncoKB
PDGFRA	PDGFRA	ILORASERTIB	inhibitor	DTC|ChemblInteractions|GuideToPharmacology
PDGFRA	PDGFRA	ENMD-2076	inhibitor	TdgClinicalTrial|GuideToPharmacology
PDGFRA	PDGFRA	FAMITINIB	inhibitor	ChemblInteractions|TTD
PDGFRA	PDGFRA	RIPRETINIB	inhibitor	JAX-CKB|TTD
PDGFRA	PDGFRA	DASATINIB		DTC|JAX-CKB|CGI|DoCM|CIViC|OncoKB
PDGFRA	PDGFRA	VANDETANIB		DTC
PDGFRA	PDGFRA	LINIFANIB	inhibitor	DTC|ChemblInteractions
PDGFRA	PDGFRA	OLARATUMAB	antagonist|antibody	MyCancerGenome|TdgClinicalTrial|ChemblInteractions|GuideToPharmacology|PharmGKB|TTD|FDA
PDGFRA	PDGFRA	CYC-116		DTC
PDGFRA	PDGFRA	AVAPRITINIB		JAX-CKB|CIViC|TTD
PDGFRA	PDGFRA	AMUVATINIB	inhibitor	MyCancerGenome|TdgClinicalTrial|ChemblInteractions
PDGFRA	PDGFRA	PAZOPANIB	inhibitor	TALC|DTC|MyCancerGenome|MyCancerGenomeClinicalTrial
PDGFRA	PDGFRA	NINTEDANIB	inhibitor	TALC|MyCancerGenome|TdgClinicalTrial|ClearityFoundationClinicalTrial|GuideToPharmacology
PDGFRA	PDGFRA	ENMD-981693	inhibitor	ChemblInteractions
PDGFRA	PDGFRA	PD-0166285		DTC
PDGFRA	PDGFRA	PEGPLERANIB SODIUM		TdgClinicalTrial
PDGFRA	PDGFRA	REGORAFENIB	inhibitor	JAX-CKB|ChemblInteractions|CGI|MyCancerGenomeClinicalTrial
PDGFRA	PDGFRA	TELATINIB		TdgClinicalTrial
PDGFRA	PDGFRA	SORAFENIB	inhibitor	DTC|CIViC|MyCancerGenomeClinicalTrial
PDGFRA	PDGFRA	SU-014813	inhibitor	ChemblInteractions

PDGFRA	PDGFRA	TANDUTINIB	inhibitor	DTC|MyCancerGenome
PDGFRA	PDGFRA	ROMIPLOSTIM		TTD
PDGFRA	PDGFRA	FORETINIB	inhibitor	ChemblInteractions
PDGFRA	PDGFRA	MASITINIB	inhibitor	TdgClinicalTrial|ChemblInteractions|GuideToPharmacology|MyCancerGenomeClinicalTrial
PDGFRA	PDGFRA	R-406		DTC
PDGFRA	PDGFRA	CARBOPLATIN		JAX-CKB
PDGFRA	PDGFRA	SUNITINIB	inhibitor	TdgClinicalTrial|JAX-CKB|CGI|TEND|DoCM|COSMIC|CIViC|MyCancerGenomeClinicalTrial
PDGFRA	PDGFRA	DOVITINIB	inhibitor	DTC|ClearityFoundationClinicalTrial|ChemblInteractions|GuideToPharmacology
PDGFRA	PDGFRA	X-82	inhibitor	MyCancerGenome|ChemblInteractions
PDGFRA	PDGFRA	PONATINIB	inhibitor	DTC|JAX-CKB|CIViC
PDGFRA	PDGFRA	CRENOLANIB	inhibitor	MyCancerGenome|JAX-CKB|ChemblInteractions|CGI|DoCM|CIViC|GuideToPharmacology|TTD
PDGFRA	PDGFRA	QUIZARTINIB	inhibitor	ChemblInteractions|GuideToPharmacology
PDGFRA	PDGFRA	MOTESANIB	inhibitor	MyCancerGenome|TdgClinicalTrial|ChemblInteractions
PDGFRA	PDGFRA	BECAPLERMIN	agonist	TdgClinicalTrial|ChemblInteractions|TEND
PDGFRA	PDGFRA	ORANTINIB	inhibitor	ChemblInteractions
PDGFRA	PDGFRA	CHEMBL406845		DTC
PDGFRA	PDGFRA	LUCITANIB	inhibitor	GuideToPharmacology
PDGFRA	PDGFRA	SITRAVATINIB	inhibitor	GuideToPharmacology
PDGFRA	PDGFRA	CEDIRANIB	inhibitor	DTC|ChemblInteractions|GuideToPharmacology
PDGFRA	PDGFRA	AZD-1152-HQPA		DTC
PDGFRA	PDGFRA	MIDOSTAURIN	antagonist|inhibitor	MyCancerGenome|ChemblInteractions
PDGFRA	PDGFRA	AST-487		DTC
PDGFRA	PDGFRA	TOVETUMAB	antibody	TALC|MyCancerGenome|TTD
PDGFRA	PDGFRA	GEFITINIB		JAX-CKB
PDGFRA	PDGFRA	NILOTINIB	inhibitor	JAX-CKB|MyCancerGenomeClinicalTrial
PDGFRA	PDGFRA	RABEPRAZOLE SODIUM		TTD
PDGFRA	PDGFRA	RAMUCIRUMAB	antibody	TALC|MyCancerGenome
PDGFRA	PDGFRA	PACLITAXEL		JAX-CKB
PDGFRA	PDGFRA	CENISERTIB		DTC
PDGFRA	PDGFRA	VISTUSERTIB		JAX-CKB
PDGFRA	PDGFRA	VATALANIB	inhibitor	DTC|TdgClinicalTrial|ChemblInteractions
PDGFRA	PDGFRA	XL-820	inhibitor	ChemblInteractions|TTD
PDGFRA	PDGFRA	SUNITINIB MALATE	inhibitor	ChemblInteractions
PDGFRA	PDGFRA	TAE-684		DTC
PDGFRA	PDGFRA	LENVATINIB	inhibitor	TdgClinicalTrial
PDGFRA	PDGFRA	CHEMBL202721		DTC
PDGFRA	PDGFRA	TAK-593	inhibitor	ChemblInteractions
PDGFRA	PDGFRA	BARASERTIB		DTC
PDGFRA	PDGFRA	RG-1530		DTC
PDGFRA	PDGFRA	CHEMBL1997335		DTC
PDGFRA	PDGFRA	XL-999	inhibitor	ChemblInteractions
PDGFRA	PDGFRA	NINTEDANIB ESYLATE	inhibitor	ChemblInteractions
PDGFRA	PDGFRA	PAZOPANIB HYDROCHLORIDE	inhibitor	ChemblInteractions
PDGFRA	PDGFRA	OSI-632		DTC
PDGFRA	PDGFRA	AXITINIB		TdgClinicalTrial
P4HA1	P4HA1	PYRIDINE-2,5-DICARBOXYLIC ACID		DTC
P4HA1	P4HA1	LUFIRONIL		TTD
P4HA1	P4HA1	SAFIRONIL		TTD
BIRC7	BIRC7	GDC-0152	antagonist	GuideToPharmacology
BIRC7	BIRC7	AT-406	antagonist	TALC
BIRC7	BIRC7	CISPLATIN		CIViC
HMGCS2	HMGCS2	HYDROCHLOROTHIAZIDE		PharmGKB
HMGCS2	HMGCS2	CHLOROTHIAZIDE		PharmGKB
PLAT	PLAT	MELPHALAN		NCI
PLAT	PLAT	BORTEZOMIB		DTC
PLAT	PLAT	NAPROXEN		NCI
PLAT	PLAT	UROKINASE		TdgClinicalTrial
PLAT	PLAT	EPOETIN BETA		NCI
PLAT	PLAT	ATORVASTATIN		NCI
PLAT	PLAT	RALOXIFENE		NCI
PLAT	PLAT	CHEMBL290376	inhibitor	GuideToPharmacology

PLAT	PLAT	AMINOCAPROIC ACID	inhibitor	ChemblInteractions
MYH14	MYH14	MAVACAMTEN		TTD
C3AR1	C3AR1	CHEMBL389348		DTC
CD74	CD74	MILATUZUMAB	antagonist|antibody	MyCancerGenome|TdgClinicalTrial|ChemblInteractions|GuideToPharmacology|TTD
PTHLH	PTHLH	VINBLASTINE		NCI
PTHLH	PTHLH	CAL	inhibitor	ChemblInteractions
CCND1	CCND1	NIFEDIPINE		NCI
CCND1	CCND1	BRICICLIB	inhibitor	ChemblInteractions|TTD
CCND1	CCND1	FLUOROURACIL		PharmGKB
CCND1	CCND1	METHOTREXATE		NCI|PharmGKB
CCND1	CCND1	TAMOXIFEN		NCI|CIViC
CCND1	CCND1	LAPATINIB		PharmGKB
CCND1	CCND1	ACETAMINOPHEN		NCI
CCND1	CCND1	PALBOCICLIB	inhibitor	DTC|ChemblInteractions
CCND1	CCND1	TROGLITAZONE		NCI
CCND1	CCND1	PROGESTERONE		NCI
CCND1	CCND1	GOSSYPOL		NCI
CCND1	CCND1	RIBOCICLIB		ClearityFoundationBiomarkers|CIViC
CCND1	CCND1	URACIL		NCI
CCND1	CCND1	ALVOCIDIB		DTC
CCND1	CCND1	VITAMIN E		NCI
CCND1	CCND1	ABEMACICLIB		ClearityFoundationBiomarkers
CCND1	CCND1	SELICICLIB		DTC
CCND1	CCND1	NAVITOCLAX		TTD
CCND1	CCND1	CETUXIMAB		PharmGKB
CCND1	CCND1	BORTEZOMIB		CIViC
PTGER3	PTGER3	ENPROSTIL	agonist	GuideToPharmacology
PTGER3	PTGER3	DINOPROSTONE	agonist	DTC|TdgClinicalTrial|ChemblInteractions|TEND|GuideToPharmacology
PTGER3	PTGER3	TREPROSTINIL	agonist	GuideToPharmacology
PTGER3	PTGER3	BUTAPROST		TTD
PTGER3	PTGER3	SEPETAPROST		TTD
PTGER3	PTGER3	CICAPROST	agonist	GuideToPharmacology
PTGER3	PTGER3	ILOPROST	agonist	GuideToPharmacology
PTGER3	PTGER3	SULPROSTONE	agonist	GuideToPharmacology
PTGER3	PTGER3	CLOPROSTENOL	agonist	GuideToPharmacology
PTGER3	PTGER3	CHEMBL565591	antagonist	GuideToPharmacology
PTGER3	PTGER3	CARBACYCLIN	agonist	GuideToPharmacology
PTGER3	PTGER3	BIMATOPROST	agonist	TdgClinicalTrial
PTGER3	PTGER3	FLUPROSTENOL	agonist	GuideToPharmacology
PTGER3	PTGER3	BERAPROST	agonist	GuideToPharmacology
PTGER3	PTGER3	MISOPROSTOL	agonist	ChemblInteractions|NCI
PTGER3	PTGER3	ASPIRIN		PharmGKB
PLAU	PLAU	RETINOL		NCI
PLAU	PLAU	RAZOXANE		NCI
PLAU	PLAU	ALENDRONIC ACID		NCI
PLAU	PLAU	AMEDIPLASE		TTD
PLAU	PLAU	CARMUSTINE		NCI
PLAU	PLAU	HYDROCORTISONE		NCI
PLAU	PLAU	FILGRASTIM		NCI
PLAU	PLAU	CALCITRIOL		NCI
PLAU	PLAU	UROKINASE		TdgClinicalTrial|TTD
PLAU	PLAU	IONOMYCIN		NCI
PLAU	PLAU	VERAPAMIL		NCI
PLAU	PLAU	UPAMOSTAT	inhibitor	TdgClinicalTrial|GuideToPharmacology|TTD
PLAU	PLAU	CISPLATIN		NCI
PLAU	PLAU	DACARBAZINE		NCI
PLAU	PLAU	SIROLIMUS		NCI
PLAU	PLAU	BLEOMYCIN		NCI
PLAU	PLAU	CLODRONIC ACID		NCI
PLAU	PLAU	ESTRADIOL		NCI
PLAU	PLAU	ANTIBIOTIC		NCI

PLAU	PLAU	AZACITIDINE		NCI
PLAU	PLAU	IRESSA		NCI
PLAU	PLAU	PAPAVERINE		NCI
PLAU	PLAU	ZOLEDRONIC ACID		NCI
PLAU	PLAU	GELDANAMYCIN		NCI
PLAU	PLAU	CELECOXIB		NCI
PLAU	PLAU	SARUPLASE		TTD
HLA-DQA1	HLA-DQA1	MERCAPTOPURINE		PharmGKB
HLA-DQA1	HLA-DQA1	LAPATINIB		PharmGKB|FDA
HLA-DQA1	HLA-DQA1	AZATHIOPRINE		PharmGKB
HLA-DQA1	HLA-DQA1	LUMIRACOXIB		PharmGKB
KCNJ13	KCNJ13	DALFAMPRIDINE	blocker	GuideToPharmacology
TGFB1	TGFB1	VERAPAMIL		NCI
TGFB1	TGFB1	TOREMIFENE		NCI
TGFB1	TGFB1	TAMOXIFEN		NCI
TGFB1	TGFB1	PIRFENIDONE		NCI|TTD
TGFB1	TGFB1	TRIAMCINOLONE		NCI
TGFB1	TGFB1	HYDROCORTISONE		NCI
TGFB1	TGFB1	M-7824		TTD
TGFB1	TGFB1	INOSITOL		NCI
TGFB1	TGFB1	SILYBIN A		NCI
TGFB1	TGFB1	VACTOSERTIB		TTD
TGFB1	TGFB1	IRINOTECAN		PharmGKB
TGFB1	TGFB1	SOTATERCEPT		TTD
TGFB1	TGFB1	GENISTEIN		NCI
TGFB1	TGFB1	FRESOLIMUMAB	antibody|inhibitor	TALC|ChemblInteractions
TGFB1	TGFB1	NIS-793		TTD
TGFB1	TGFB1	RITUXIMAB		PharmGKB
TGFB1	TGFB1	SANDOSTATIN		NCI
TGFB1	TGFB1	RAMIPRIL		NCI
TGFB1	TGFB1	STREPTOZOCIN		NCI
TGFB1	TGFB1	ASPIRIN		PharmGKB
TGFB1	TGFB1	TESTOSTERONE		NCI
TGFB1	TGFB1	METELIMUMAB	inhibitor	ChemblInteractions
TGFB1	TGFB1	ADRIAMYCIN		NCI
TGFB1	TGFB1	MELATONIN		NCI
TGFB1	TGFB1	DOXORUBICIN		NCI
TGFB1	TGFB1	CLADRIBINE		NCI
TGFB1	TGFB1	ATENOLOL		NCI
TGFB1	TGFB1	AMIFOSTINE		NCI
TGFB1	TGFB1	ETOPOSIDE		NCI
TGFB1	TGFB1	LY-2382770	inhibitor	ChemblInteractions
TGFB1	TGFB1	NICOTINE		NCI
TGFB1	TGFB1	EMODIN		NCI
TGFB1	TGFB1	DISITERTIDE		TTD
TGFB1	TGFB1	VITAMIN E		NCI
TGFB1	TGFB1	FENRETINIDE		NCI
TGFB1	TGFB1	PIOGLITAZONE		NCI
TGFB1	TGFB1	GOSSYPOL		NCI
GRB14	GRB14	INSULIN		NCI
LAG3	LAG3	RELATLIMAB	antibody	GuideToPharmacology|TTD
LAG3	LAG3	GSK2831781		TTD
CXCL13	CXCL13	RITUXIMAB		PharmGKB
HLA-G	HLA-G	FLUOROURACIL		PharmGKB
HLA-G	HLA-G	METHOTREXATE		PharmGKB
HLA-G	HLA-G	CAPECITABINE		PharmGKB
SLC44A4	SLC44A4	ASG-5ME		TTD
FBP1	FBP1	MB-07803		TdgClinicalTrial|TTD
SLC2A3	SLC2A3	RESVERATROL		DTC
SLC2A3	SLC2A3	INSULIN		NCI
SLC2A3	SLC2A3	GLUFOSFAMIDE		TdgClinicalTrial

COL6A2	COL6A2	COLLAGENASE CLOSTRIDIUM HISTOLYTICUM		ChemblInteractions
COL6A2	COL6A2	OCRIPLASMIN		ChemblInteractions
BIRC3	BIRC3	AT-406	antagonist	TALC|GuideToPharmacology|TTD
BIRC3	BIRC3	GDC-0152	antagonist	GuideToPharmacology
BIRC3	BIRC3	CHEMBL504559	antagonist	GuideToPharmacology
BIRC3	BIRC3	BESTATIN METHYL ESTER		DTC
BIRC3	BIRC3	BIRINAPANT	inhibitor|antagonist	TALC|ChemblInteractions
BIRC3	BIRC3	LCL-161	antagonist	TALC
BIRC3	BIRC3	CHEMBL481422	inhibitor	GuideToPharmacology
SAMHD1	SAMHD1	STAVUDINE		PharmGKB
ASS1	ASS1	CHLOROQUINE		CIViC
ASS1	ASS1	PEGARGIMINASE		CIViC
NRP2	NRP2	DAUNORUBICIN		PharmGKB
ALOX5	ALOX5	TEPOXALIN		TTD
ALOX5	ALOX5	MESALAMINE	inhibitor	TdgClinicalTrial|ChemblInteractions
ALOX5	ALOX5	TENIDAP		TTD
ALOX5	ALOX5	CHEMBL416038		DTC
ALOX5	ALOX5	PF-4191834	inhibitor	GuideToPharmacology|TTD
ALOX5	ALOX5	RILOPIROX		TTD
ALOX5	ALOX5	ZILEUTON	inhibitor	DTC|TdgClinicalTrial|ChemblInteractions|NCI|TEND|GuideToPharmacology|PharmGKB|TTD
ALOX5	ALOX5	SULFASALAZINE	inhibitor	ChemblInteractions
ALOX5	ALOX5	SILYBIN A		TTD
ALOX5	ALOX5	DIETHYLCARBAMAZINE	inhibitor	TTD
ALOX5	ALOX5	MONTELUKAST	other/unknown	PharmGKB
ALOX5	ALOX5	TIPELUKAST		TTD
ALOX5	ALOX5	ATRELEUTON		TdgClinicalTrial|TTD
ALOX5	ALOX5	LICOFELONE		TdgClinicalTrial
ALOX5	ALOX5	5-OXO-ETE		DTC
ALOX5	ALOX5	ACACETIN		DTC
ALOX5	ALOX5	DARBUFELONE		TTD
ALOX5	ALOX5	MELATONIN		NCI
ALOX5	ALOX5	MECLOFENAMATE SODIUM	inhibitor	ChemblInteractions
ALOX5	ALOX5	CHEMBL502496		DTC
ALOX5	ALOX5	BALSALAZIDE DISODIUM	inhibitor	ChemblInteractions
ALOX5	ALOX5	BALSALAZIDE	inhibitor	TdgClinicalTrial
ALOX5	ALOX5	METHOTREXATE		NCI
ALOX5	ALOX5	6BIO		DTC
ALOX5	ALOX5	OLSALAZINE SODIUM	inhibitor	ChemblInteractions
CA2	CA2	DICHLORPHENAMIDE	inhibitor	ChemblInteractions|TTD
CA2	CA2	DORZOLAMIDE	inhibitor	NCI|TTD
CA2	CA2	ACETAZOLAMIDE	inhibitor	TdgClinicalTrial|ChemblInteractions|TEND
CA2	CA2	SULFAMIDE		TTD
CA2	CA2	ETHINAMATE	inhibitor	TTD
CA2	CA2	BRINZOLAMIDE	inhibitor	TdgClinicalTrial|ChemblInteractions|TEND
CA2	CA2	CYCLOTHIAZIDE	inhibitor	TTD
CA2	CA2	ETHOXZOLAMIDE	inhibitor	ChemblInteractions|TTD
CA2	CA2	TRICHLORMETHIAZIDE	inhibitor	TdgClinicalTrial|TEND
CA2	CA2	BENZTHIAZIDE	inhibitor	TTD
CA2	CA2	CHLOROTHIAZIDE	inhibitor	TdgClinicalTrial|TEND|TTD
CA2	CA2	SULTHIAME		TTD
CA2	CA2	POLMACOXIB	inhibitor	ChemblInteractions|TTD
CA2	CA2	CURCUMIN		TTD
CA2	CA2	PARABEN		TTD
CA2	CA2	TOPIRAMATE	inhibitor	TdgClinicalTrial|ChemblInteractions|TEND
CA2	CA2	DORZOLAMIDE HYDROCHLORIDE	inhibitor	ChemblInteractions
CA2	CA2	METHAZOLAMIDE	inhibitor	ChemblInteractions
CA2	CA2	SULFAMYLON		TTD
CA2	CA2	ACETAZOLAMIDE SODIUM	inhibitor	ChemblInteractions
CA2	CA2	PHENOL		TTD
CA2	CA2	EPOETIN ALFA		NCI
CA2	CA2	GUAIACOL		TTD

SLC2A1	SLC2A1	DIAZEPAM		NCI
SLC2A1	SLC2A1	PIOGLITAZONE		NCI
SLC2A1	SLC2A1	GENISTEIN		NCI
SLC2A1	SLC2A1	TRIAMCINOLONE		NCI
SLC2A1	SLC2A1	THIOCTIC ACID		DTC
SLC2A1	SLC2A1	PROGESTERONE		NCI
SLC2A1	SLC2A1	GLUFOSFAMIDE		TdgClinicalTrial
SLC2A1	SLC2A1	THYMIDINE		NCI
SLC2A1	SLC2A1	PHENYTOIN		NCI
SLC2A1	SLC2A1	ROSIGLITAZONE		NCI
SLC2A1	SLC2A1	GENTAMICIN		NCI
CD200	CD200	SAMALIZUMAB	inhibitor	ChemblInteractions|TTD
SLFN11	SLFN11	NIRAPARIB		CIViC
SLFN11	SLFN11	TEMOZOLOMIDE		CIViC
SLFN11	SLFN11	TALAZOPARIB		CIViC
MCHR1	MCHR1	CHEMBL178707	antagonist	GuideToPharmacology|TTD
MCHR1	MCHR1	SNAP-7941	antagonist	GuideToPharmacology|TTD
MCHR1	MCHR1	CHEMBL2110360	antagonist	DTC|GuideToPharmacology
MCHR1	MCHR1	BMS-830216		TTD
MCHR1	MCHR1	CHEMBL214957	antagonist	GuideToPharmacology
PSORS1C3	PSORS1C3	ALLOPURINOL		PharmGKB
AXL	AXL	DASATINIB		DTC
AXL	AXL	PF-00562271		DTC
AXL	AXL	GILTERITINIB	inhibitor	ChemblInteractions|GuideToPharmacology|TTD
AXL	AXL	HESPERADIN	inhibitor	DTC
AXL	AXL	BMS-754807		DTC
AXL	AXL	DUBERMATINIB	inhibitor	GuideToPharmacology|TTD
AXL	AXL	GLESATINIB		TTD
AXL	AXL	CEDIRANIB		DTC
AXL	AXL	GW843682X		DTC
AXL	AXL	LINIFANIB		DTC
AXL	AXL	R428	inhibitor	ChemblInteractions
AXL	AXL	BEMCENTINIB	inhibitor	GuideToPharmacology|TTD
AXL	AXL	BPI-9016	inhibitor	ChemblInteractions
AXL	AXL	SORAFENIB		DTC
AXL	AXL	OSI-632		DTC
AXL	AXL	ILORASERTIB		DTC
AXL	AXL	GONIOTHALAMIN		DTC
AXL	AXL	BI-505		TTD
AXL	AXL	SITRAVATINIB	inhibitor	GuideToPharmacology
AXL	AXL	MERESTINIB	inhibitor	GuideToPharmacology
AXL	AXL	DOVITINIB		DTC
AXL	AXL	BMS-777607	inhibitor	GuideToPharmacology
AXL	AXL	VANDETANIB		DTC
AXL	AXL	GEFITINIB		DTC
AXL	AXL	TAE-684		DTC
AXL	AXL	CENISERTIB		DTC
AXL	AXL	ENTRECTINIB		DTC
AXL	AXL	TOZASERTIB		DTC
TFAP2A	TFAP2A	NELFINAVIR		NCI
TFAP2A	TFAP2A	INDINAVIR		NCI
TFAP2A	TFAP2A	AMPRENAVIR		NCI
COL4A1	COL4A1	OCRIPLASMIN		ChemblInteractions
COL4A1	COL4A1	COLLAGENASE CLOSTRIDIUM HISTOLYTICUM		ChemblInteractions
NTRK2	NTRK2	LITHIUM		NCI|PharmGKB
NTRK2	NTRK2	AST-487		DTC
NTRK2	NTRK2	PILOCARPINE		NCI
NTRK2	NTRK2	AZD-7451	inhibitor	ChemblInteractions
NTRK2	NTRK2	CHEMBL546797		DTC
NTRK2	NTRK2	DORAMAPIMOD		DTC
NTRK2	NTRK2	ENTRECTINIB	inhibitor	DTC|CIViC|PharmGKB|OncoKB

NTRK2	NTRK2	LAROTRECTINIB	inhibitor	ChemblInteractions|CIViC|TTD|OncoKB
NTRK2	NTRK2	HESPERADIN	inhibitor	DTC
NTRK2	NTRK2	SORAFENIB		DTC
NTRK2	NTRK2	ALTIRATINIB	inhibitor	ChemblInteractions
NTRK2	NTRK2	CISPLATIN		NCI
NTRK2	NTRK2	MK-2461	inhibitor	GuideToPharmacology|TTD
NTRK2	NTRK2	TOZASERTIB		DTC
NTRK2	NTRK2	DOVITINIB		DTC
NTRK2	NTRK2	CEP-2563	inhibitor	ChemblInteractions
NTRK2	NTRK2	SNS-314		DTC
NTRK2	NTRK2	CORTICOTROPIN		NCI
NTRK2	NTRK2	DITIOCARB		NCI
NTRK2	NTRK2	GW632580X		DTC
NTRK2	NTRK2	PF-00562271		DTC
NTRK2	NTRK2	METHADONE		PharmGKB
NTRK2	NTRK2	PLX-7486	inhibitor	ChemblInteractions
NTRK2	NTRK2	LINIFANIB		DTC
NTRK2	NTRK2	CHEMBL202721		DTC
NTRK2	NTRK2	SB-202190		DTC
NTRK2	NTRK2	AZD-6918	inhibitor	ChemblInteractions
NTRK2	NTRK2	SP-600125		DTC
NTRK2	NTRK2	LAUROGUADINE		DTC
NTRK2	NTRK2	JNJ-7706621		DTC
NTRK2	NTRK2	CLOZAPINE		PharmGKB
NTRK2	NTRK2	R-406		DTC
NTRK2	NTRK2	LESTAURTINIB	inhibitor	TdgClinicalTrial|ChemblInteractions
NTRK2	NTRK2	ILORASERTIB		DTC
NTRK2	NTRK2	SELITRECTINIB	inhibitor	GuideToPharmacology
NTRK2	NTRK2	CEP-11981	inhibitor	GuideToPharmacology
NTRK2	NTRK2	RG-1530		DTC
NTRK2	NTRK2	GW441756X		DTC
NTRK2	NTRK2	ALISERTIB		DTC
NTRK2	NTRK2	CENISERTIB		DTC
NTRK2	NTRK2	PD-0166285		DTC
NTRK2	NTRK2	GAMBOGIC AMIDE		DTC
NTRK2	NTRK2	DOXORUBICIN		NCI
NTRK2	NTRK2	BMS-754807		DTC
NTRK2	NTRK2	REPOTRECTINIB	inhibitor	GuideToPharmacology
USP2	USP2	QUINALIZARIN		DTC
USP2	USP2	THONZONIUM BROMIDE		DTC
USP2	USP2	DEPHOSTATIN		DTC
USP2	USP2	SURAMIN		DTC
USP2	USP2	CALMIDAZOLIUM CHLORIDE		DTC
USP2	USP2	ZAPRINAST		DTC
USP2	USP2	CHEMBL461579		DTC
USP2	USP2	JUGLONE		DTC
USP2	USP2	SENNOSIDE B		DTC
USP2	USP2	ELLAGIC ACID		DTC
USP2	USP2	CADMIUM ACETATE		DTC
USP2	USP2	CHEMBL56393		DTC
USP2	USP2	HEXACHLOROPHENE		DTC
USP2	USP2	CURCUMIN		DTC
USP2	USP2	MANGOSTIN		DTC
USP2	USP2	AURINTRICARBOXYLIC ACID		DTC
USP2	USP2	GOSSYPOL		DTC
USP2	USP2	CHEMBL578741		DTC
USP2	USP2	TANNIC ACID		DTC
USP2	USP2	ALEXIDINE HYDROCHLORIDE		DTC
USP2	USP2	ROBUSTIC ACID		DTC
USP2	USP2	GENTIAN VIOLET		DTC
USP2	USP2	CLOFOCTOL		DTC

USP2	USP2	CHAULMOSULFONE		DTC
USP2	USP2	ALLOXAN		DTC
USP2	USP2	IODOACETAMIDE		DTC
CD14	CD14	IC14	inhibitor	TdgClinicalTrial|ChemblInteractions|TTD
CD14	CD14	LOVASTATIN		NCI
CSF1R	CSF1R	SUNITINIB MALATE	inhibitor	ChemblInteractions
CSF1R	CSF1R	ZOTIRACICLIB	inhibitor	GuideToPharmacology
CSF1R	CSF1R	AST-487		DTC
CSF1R	CSF1R	CERDULATINIB	inhibitor	GuideToPharmacology
CSF1R	CSF1R	DASATINIB	inhibitor	TALC
CSF1R	CSF1R	GW843682X		DTC
CSF1R	CSF1R	SURUFATINIB		TTD
CSF1R	CSF1R	GW632580X		DTC
CSF1R	CSF1R	IMATINIB	antagonist	DTC|TdgClinicalTrial|JAX-CKB|CGI|TEND|DoCM|CIViC
CSF1R	CSF1R	CYC-116		DTC
CSF1R	CSF1R	REGRAMOSTIM		GuideToPharmacology
CSF1R	CSF1R	CC-223	inhibitor	GuideToPharmacology
CSF1R	CSF1R	ARRY-382	inhibitor	ChemblInteractions|GuideToPharmacology|TTD
CSF1R	CSF1R	EMACTUZUMAB	antibody	GuideToPharmacology
CSF1R	CSF1R	CRENOLANIB	inhibitor	GuideToPharmacology
CSF1R	CSF1R	TANDUTINIB	inhibitor	DTC|GuideToPharmacology
CSF1R	CSF1R	PLX-5622	inhibitor	ChemblInteractions
CSF1R	CSF1R	QUIZARTINIB	inhibitor	ChemblInteractions|GuideToPharmacology
CSF1R	CSF1R	HESPERADIN	inhibitor	DTC
CSF1R	CSF1R	EDICOTINIB	inhibitor	ChemblInteractions|GuideToPharmacology|TTD
CSF1R	CSF1R	PEXIDARTINIB	inhibitor	MyCancerGenome|JAX-CKB|ChemblInteractions|CIViC|GuideToPharmacology|CancerCommons
CSF1R	CSF1R	DOVITINIB	inhibitor	DTC|ClearityFoundationClinicalTrial|CIViC|GuideToPharmacology
CSF1R	CSF1R	PLX-7486	inhibitor	ChemblInteractions
CSF1R	CSF1R	CENISERTIB		DTC
CSF1R	CSF1R	SUNITINIB	inhibitor	TEND
CSF1R	CSF1R	CEDIRANIB	inhibitor	DTC|GuideToPharmacology
CSF1R	CSF1R	KENPAULLONE		DTC
CSF1R	CSF1R	LINIFANIB	inhibitor	DTC|TdgClinicalTrial|ChemblInteractions|GuideToPharmacology
CSF1R	CSF1R	OSI-930	inhibitor	GuideToPharmacology
CSF1R	CSF1R	CHEMBL365617		DTC
CSF1R	CSF1R	PROGESTERONE		NCI
CSF1R	CSF1R	PAZOPANIB	inhibitor	DTC|GuideToPharmacology
CSF1R	CSF1R	MCS-110		TTD
CSF1R	CSF1R	VEMURAFENIB		JAX-CKB
CSF1R	CSF1R	SORAFENIB		DTC|JAX-CKB
CSF1R	CSF1R	CHEMBL1908396	inhibitor	GuideToPharmacology
CSF1R	CSF1R	CHEMBL406375	inhibitor	GuideToPharmacology
CSF1R	CSF1R	CILMOSTIM		GuideToPharmacology
CSF1R	CSF1R	CT-1578	inhibitor	ChemblInteractions
CSF1R	CSF1R	SP-600125		DTC
CSF1R	CSF1R	CHEMBL1908395	inhibitor	GuideToPharmacology
CSF1R	CSF1R	CHEMBL546797		DTC
CSF1R	CSF1R	DACTOLISIB		DTC
CSF1R	CSF1R	PAZOPANIB HYDROCHLORIDE	inhibitor	ChemblInteractions
CSF1R	CSF1R	ILORASERTIB	inhibitor	DTC|GuideToPharmacology
CSF1R	CSF1R	PD-0166285		DTC
CSF1R	CSF1R	JNJ-7706621		DTC
CSF1R	CSF1R	ENMD-2076	inhibitor	TdgClinicalTrial|ChemblInteractions
CSF1R	CSF1R	VATALANIB	inhibitor	DTC|ChemblInteractions|CIViC
CSF1R	CSF1R	GO-6976		DTC
CSF1R	CSF1R	RG-1530		DTC
CSF1R	CSF1R	TAE-684		DTC
CSF1R	CSF1R	CABIRALIZUMAB	antibody	GuideToPharmacology|TTD
CSF1R	CSF1R	IMC-CS4		TTD
CSF1R	CSF1R	MASITINIB		TdgClinicalTrial
CSF1R	CSF1R	ENTRECTINIB		DTC

CSF1R	CSF1R	ALSTERPAULLONE		DTC
CSF1R	CSF1R	FILGRASTIM		GuideToPharmacology
CSF1R	CSF1R	PD-360324		TTD
CSF1R	CSF1R	CHEMBL541400		DTC
SLC5A3	SLC5A3	PHLORIZIN	inhibitor	GuideToPharmacology
SLC22A7	SLC22A7	CAPECITABINE		PharmGKB
SLC22A7	SLC22A7	ZIDOVUDINE		PharmGKB
SLC22A7	SLC22A7	GS-9256		DTC
PTPRC	PTPRC	HYDROGEN PEROXIDE		DTC
PTPRC	PTPRC	PREDNISONE		NCI
PTPRC	PTPRC	EPOETIN BETA		NCI
PTPRC	PTPRC	ETANERCEPT		PharmGKB
PTPRC	PTPRC	ADALIMUMAB		PharmGKB
PTPRC	PTPRC	CHEMBL204543		DTC
PTPRC	PTPRC	APAMISTAMAB IODINE I-131		TTD
PTPRC	PTPRC	ALENDRONIC ACID		DTC
PTPRC	PTPRC	FLUOROURACIL		NCI
PTPRC	PTPRC	HYDROCORTISONE		NCI
PTPRC	PTPRC	ESTRADIOL		NCI
PTPRC	PTPRC	INFLIXIMAB		PharmGKB
COL5A2	COL5A2	OCRIPLASMIN		ChemblInteractions
COL5A2	COL5A2	COLLAGENASE CLOSTRIDIUM HISTOLYTICUM		ChemblInteractions
HLA-DRB1	HLA-DRB1	FLUPIRTINE		PharmGKB
HLA-DRB1	HLA-DRB1	FLUVASTATIN		PharmGKB
HLA-DRB1	HLA-DRB1	METHIMAZOLE		PharmGKB
HLA-DRB1	HLA-DRB1	ASPIRIN		PharmGKB
HLA-DRB1	HLA-DRB1	LYM-1		ChemblInteractions|TTD
HLA-DRB1	HLA-DRB1	EFAVIRENZ		PharmGKB
HLA-DRB1	HLA-DRB1	INFLIXIMAB		PharmGKB
HLA-DRB1	HLA-DRB1	ADALIMUMAB		PharmGKB
HLA-DRB1	HLA-DRB1	PROPYLTHIOURACIL		PharmGKB
HLA-DRB1	HLA-DRB1	LAPATINIB		PharmGKB|FDA
HLA-DRB1	HLA-DRB1	PITAVASTATIN		PharmGKB
HLA-DRB1	HLA-DRB1	SIMVASTATIN		PharmGKB
HLA-DRB1	HLA-DRB1	DABRAFENIB		PharmGKB
HLA-DRB1	HLA-DRB1	PRAVASTATIN		PharmGKB
HLA-DRB1	HLA-DRB1	BUCILLAMINE		PharmGKB
HLA-DRB1	HLA-DRB1	ATORVASTATIN		PharmGKB
HLA-DRB1	HLA-DRB1	ASPARAGINASE		PharmGKB
HLA-DRB1	HLA-DRB1	CARBIMAZOLE		PharmGKB
HLA-DRB1	HLA-DRB1	DAPSONE		PharmGKB
HLA-DRB1	HLA-DRB1	LUMIRACOXIB		PharmGKB
HLA-DRB1	HLA-DRB1	CARBAMAZEPINE		PharmGKB
HLA-DRB1	HLA-DRB1	CLAVULANIC ACID		PharmGKB
HLA-DRB1	HLA-DRB1	ETANERCEPT		PharmGKB
HLA-DRB1	HLA-DRB1	OXCARBAZEPINE		PharmGKB
HLA-DRB1	HLA-DRB1	LAMOTRIGINE		PharmGKB
HLA-DRB1	HLA-DRB1	FLOXACILLIN		PharmGKB
HLA-DRB1	HLA-DRB1	ROSUVASTATIN		PharmGKB
HLA-DRB1	HLA-DRB1	AMOXICILLIN		PharmGKB
HLA-DRB1	HLA-DRB1	MERCAPTOPURINE		PharmGKB
HLA-DRB1	HLA-DRB1	TICLOPIDINE		PharmGKB
HLA-DRB1	HLA-DRB1	AZATHIOPRINE		PharmGKB
MUC1	MUC1	HUHMFG1		TdgClinicalTrial|TTD
MUC1	MUC1	SAR-566658		TTD
MUC1	MUC1	EMEPEPIMUT-S		TTD
MUC1	MUC1	GO-203-2C		TdgClinicalTrial|TTD
HLA-DRA	HLA-DRA	AMOXICILLIN		PharmGKB
HLA-DRA	HLA-DRA	CLAVULANIC ACID		PharmGKB
HLA-DRA	HLA-DRA	PEMBROLIZUMAB		CIViC
HLA-DRA	HLA-DRA	NIVOLUMAB		CIViC

HLA-DRA	HLA-DRA	FLOXACILLIN		PharmGKB
HLA-DRA	HLA-DRA	ATEZOLIZUMAB		CIViC
SLC29A4	SLC29A4	FLUOXETINE	inhibitor	GuideToPharmacology
SLC29A4	SLC29A4	CIMETIDINE	inhibitor	GuideToPharmacology
SLC29A4	SLC29A4	METFORMIN		PharmGKB
SLC29A4	SLC29A4	HISTAMINE		DTC
SLC29A4	SLC29A4	QUININE	inhibitor	GuideToPharmacology
SLC29A4	SLC29A4	QUINIDINE	inhibitor	GuideToPharmacology
SLC29A4	SLC29A4	DIPYRIDAMOLE	inhibitor	GuideToPharmacology
SLC29A4	SLC29A4	RHODAMINE123	inhibitor	GuideToPharmacology
SLC29A4	SLC29A4	DESIPRAMINE	inhibitor	GuideToPharmacology
SLC29A4	SLC29A4	VERAPAMIL	inhibitor	GuideToPharmacology
KCNMA1	KCNMA1	CHLORZOXAZONE		TdgClinicalTrial|TEND
KCNMA1	KCNMA1	ANDOLAST	activator	ChemblInteractions
KCNMA1	KCNMA1	FLINDOKALNER	activator	ChemblInteractions|GuideToPharmacology
KCNMA1	KCNMA1	TRICHLORMETHIAZIDE		TdgClinicalTrial|TEND
KCNMA1	KCNMA1	ENFLURANE		TdgClinicalTrial|TEND
KCNMA1	KCNMA1	CHEMBL1290115	activator	GuideToPharmacology
KCNMA1	KCNMA1	BMS-223131	activator	ChemblInteractions
KCNMA1	KCNMA1	CROMOLYN		TdgClinicalTrial|TEND
KCNMA1	KCNMA1	CHLOROTHIAZIDE		TEND
KLK1	KLK1	ECALLANTIDE		TdgClinicalTrial|TEND
ABCB1	ABCB1	DAPTOMYCIN		PharmGKB
ABCB1	ABCB1	PACLITAXEL		DTC|NCI|CIViC
ABCB1	ABCB1	AGOMELATINE		PharmGKB
ABCB1	ABCB1	NICARDIPINE		PharmGKB
ABCB1	ABCB1	DEXTROMETHORPHAN		NCI
ABCB1	ABCB1	VINBLASTINE		DTC
ABCB1	ABCB1	2'-HYDROXYCHALCONE		DTC
ABCB1	ABCB1	RANOLAZINE		PharmGKB
ABCB1	ABCB1	SPIRONOLACTONE		NCI|PharmGKB
ABCB1	ABCB1	TESMILIFENE	inducer	TdgClinicalTrial
ABCB1	ABCB1	PROPRANOLOL		NCI|PharmGKB
ABCB1	ABCB1	HETEROCODEINE		DTC
ABCB1	ABCB1	RISPERIDONE		PharmGKB
ABCB1	ABCB1	DENDROAMIDE A		DTC
ABCB1	ABCB1	GABAPENTIN		NCI
ABCB1	ABCB1	REMIFENTANIL		PharmGKB
ABCB1	ABCB1	QUININE		PharmGKB
ABCB1	ABCB1	ERLOTINIB		PharmGKB
ABCB1	ABCB1	BIRICODAR		PharmGKB|TTD
ABCB1	ABCB1	NORTRIPTYLINE		PharmGKB
ABCB1	ABCB1	CHEMBL270688		DTC
ABCB1	ABCB1	METHYLPREDNISOLONE		PharmGKB
ABCB1	ABCB1	GRANISETRON		PharmGKB
ABCB1	ABCB1	VORICONAZOLE		PharmGKB
ABCB1	ABCB1	RITONAVIR		DTC|PharmGKB
ABCB1	ABCB1	MIDAZOLAM		PharmGKB
ABCB1	ABCB1	VALINOMYCIN		PharmGKB
ABCB1	ABCB1	PREDNISOLONE		PharmGKB
ABCB1	ABCB1	RESERPINE		DTC|PharmGKB
ABCB1	ABCB1	MORPHINE		DTC|PharmGKB
ABCB1	ABCB1	DELAVIRDINE		DTC
ABCB1	ABCB1	ALISKIREN		PharmGKB
ABCB1	ABCB1	AMIODARONE		PharmGKB
ABCB1	ABCB1	CELIPROLOL		PharmGKB
ABCB1	ABCB1	OXYCODONE		PharmGKB
ABCB1	ABCB1	PENTAZOCINE		PharmGKB
ABCB1	ABCB1	ITRACONAZOLE		NCI|PharmGKB
ABCB1	ABCB1	FLUVASTATIN		PharmGKB
ABCB1	ABCB1	7-ETHYL-10-HYDROXYCAMPTOTHECIN		PharmGKB

ABCB1	ABCB1	NIFEDIPINE		NCI
ABCB1	ABCB1	BLEOMYCIN		PharmGKB
ABCB1	ABCB1	MODAFINIL		PharmGKB
ABCB1	ABCB1	DIGOXIN		NCI
ABCB1	ABCB1	MURUCOIDIN V		DTC
ABCB1	ABCB1	ONDANSETRON		PharmGKB
ABCB1	ABCB1	PREDNISONE		PharmGKB
ABCB1	ABCB1	PURGIN II		DTC
ABCB1	ABCB1	RITUXIMAB		NCI|PharmGKB
ABCB1	ABCB1	CHEMBL217425		DTC
ABCB1	ABCB1	IDARUBICIN		PharmGKB
ABCB1	ABCB1	TENIPOSIDE		PharmGKB
ABCB1	ABCB1	PRAVASTATIN		NCI|PharmGKB
ABCB1	ABCB1	CYCLOSPORINE		DTC|NCI|PharmGKB
ABCB1	ABCB1	NEFAZODONE		PharmGKB
ABCB1	ABCB1	NELFINAVIR		DTC|PharmGKB
ABCB1	ABCB1	TARIQUIDAR	inhibitor	DTC|TdgClinicalTrial|ChemblInteractions|PharmGKB
ABCB1	ABCB1	COLCHICINE		TEND|PharmGKB
ABCB1	ABCB1	PHENOBARBITAL		NCI|PharmGKB
ABCB1	ABCB1	LOPINAVIR		DTC|PharmGKB
ABCB1	ABCB1	IRINOTECAN		PharmGKB
ABCB1	ABCB1	TANGERETIN		DTC
ABCB1	ABCB1	TAMOXIFEN		PharmGKB
ABCB1	ABCB1	METHYLPHENIDATE		PharmGKB
ABCB1	ABCB1	ADRIAMYCIN		NCI
ABCB1	ABCB1	PROPAFENONE		PharmGKB
ABCB1	ABCB1	TETRACYCLINE		PharmGKB
ABCB1	ABCB1	DOXYCYCLINE		NCI|PharmGKB
ABCB1	ABCB1	PROPOFOL		PharmGKB
ABCB1	ABCB1	CARBOPLATIN		NCI
ABCB1	ABCB1	EPIRUBICIN		NCI
ABCB1	ABCB1	LAMIVUDINE		PharmGKB
ABCB1	ABCB1	CURCUMIN		NCI|PharmGKB
ABCB1	ABCB1	FEXOFENADINE		PharmGKB
ABCB1	ABCB1	PROGESTERONE		PharmGKB
ABCB1	ABCB1	DEFEROXAMINE		NCI
ABCB1	ABCB1	PAROXETINE		PharmGKB
ABCB1	ABCB1	FLAVOKAWAIN A		DTC
ABCB1	ABCB1	CYTARABINE		PharmGKB
ABCB1	ABCB1	BISANTRENE		NCI
ABCB1	ABCB1	BROMPERIDOL		PharmGKB
ABCB1	ABCB1	CHEMBL595816		DTC
ABCB1	ABCB1	VALGANCICLOVIR		PharmGKB
ABCB1	ABCB1	MAPROTILINE		PharmGKB
ABCB1	ABCB1	QUERCETIN-3'-O-PHOSPHATE		TdgClinicalTrial
ABCB1	ABCB1	RIFAMPIN		PharmGKB
ABCB1	ABCB1	TAXOL C		DTC
ABCB1	ABCB1	LANSOPRAZOLE		PharmGKB
ABCB1	ABCB1	AMITRIPTYLINE		PharmGKB
ABCB1	ABCB1	TERFENADINE		NCI|PharmGKB
ABCB1	ABCB1	AMISULPRIDE		PharmGKB
ABCB1	ABCB1	FLUOROURACIL		NCI
ABCB1	ABCB1	CLOPIDOGREL		PharmGKB
ABCB1	ABCB1	SIMEPREVIR		PharmGKB
ABCB1	ABCB1	CAMPTOTHECIN		NCI
ABCB1	ABCB1	CARVEDILOL		PharmGKB
ABCB1	ABCB1	CIMETIDINE		PharmGKB
ABCB1	ABCB1	FENOFIBRATE		NCI
ABCB1	ABCB1	LOPERAMIDE		PharmGKB
ABCB1	ABCB1	ETOPOSIDE		PharmGKB
ABCB1	ABCB1	RANITIDINE		NCI|PharmGKB

ABCB1	ABCB1	FLUPHENAZINE		PharmGKB
ABCB1	ABCB1	QUINIDINE		NCI|PharmGKB
ABCB1	ABCB1	LEVETIRACETAM		PharmGKB
ABCB1	ABCB1	DEXRAZOXANE		PharmGKB
ABCB1	ABCB1	BEPRIDIL		PharmGKB
ABCB1	ABCB1	FELODIPINE		PharmGKB
ABCB1	ABCB1	METHADONE		NCI
ABCB1	ABCB1	PERVILLEINE B		DTC
ABCB1	ABCB1	TETRABENAZINE		PharmGKB
ABCB1	ABCB1	VOACAMINE	inhibitor	DTC
ABCB1	ABCB1	ALDOSTERONE		PharmGKB
ABCB1	ABCB1	KETOCONAZOLE		PharmGKB
ABCB1	ABCB1	CLOMIPRAMINE		PharmGKB
ABCB1	ABCB1	NEVIRAPINE		DTC|PharmGKB
ABCB1	ABCB1	MITOMYCIN		PharmGKB
ABCB1	ABCB1	LITHIUM		PharmGKB
ABCB1	ABCB1	BROMOCRIPTINE		NCI
ABCB1	ABCB1	GEFITINIB		NCI|PharmGKB
ABCB1	ABCB1	E-FLUPENTIXOL		PharmGKB
ABCB1	ABCB1	CHEMBL410907		DTC
ABCB1	ABCB1	NITRENDIPINE		PharmGKB
ABCB1	ABCB1	DOCETAXEL		PharmGKB
ABCB1	ABCB1	SIROLIMUS		NCI|PharmGKB
ABCB1	ABCB1	RUCAPARIB		PharmGKB
ABCB1	ABCB1	ELACRIDAR		PharmGKB
ABCB1	ABCB1	MEFLOQUINE		PharmGKB
ABCB1	ABCB1	CLARITHROMYCIN		NCI|PharmGKB
ABCB1	ABCB1	ZIDOVUDINE		NCI|PharmGKB
ABCB1	ABCB1	DOMPERIDONE		NCI|PharmGKB
ABCB1	ABCB1	ATAZANAVIR		NCI
ABCB1	ABCB1	HYDROCORTISONE		NCI|PharmGKB
ABCB1	ABCB1	IMATINIB		NCI
ABCB1	ABCB1	LOVASTATIN		NCI|PharmGKB
ABCB1	ABCB1	TEMOZOLOMIDE		PharmGKB
ABCB1	ABCB1	DOXORUBICIN		NCI|PharmGKB
ABCB1	ABCB1	AUSTOCYSTIN D		DTC
ABCB1	ABCB1	PERVILLEINE C		DTC
ABCB1	ABCB1	TRIMETREXATE		NCI
ABCB1	ABCB1	SORAFENIB		PharmGKB
ABCB1	ABCB1	IBUPROFEN		NCI
ABCB1	ABCB1	BOCEPREVIR		PharmGKB
ABCB1	ABCB1	ERYTHROMYCIN		PharmGKB
ABCB1	ABCB1	SUFENTANIL		PharmGKB
ABCB1	ABCB1	SILIBININ		PharmGKB
ABCB1	ABCB1	VINCRISTINE		PharmGKB
ABCB1	ABCB1	IVERMECTIN		PharmGKB
ABCB1	ABCB1	CHEMBL444075		DTC
ABCB1	ABCB1	PANTOPRAZOLE		PharmGKB
ABCB1	ABCB1	CHEMBL408422		DTC
ABCB1	ABCB1	TIPIFARNIB		PharmGKB
ABCB1	ABCB1	ZOSUQUIDAR	inhibitor	TdgClinicalTrial|PharmGKB
ABCB1	ABCB1	VERAPAMIL		DTC|NCI
ABCB1	ABCB1	PERVILLEINE F		DTC
ABCB1	ABCB1	SPARFLOXACIN		PharmGKB
ABCB1	ABCB1	PHENOTHIAZINE		NCI
ABCB1	ABCB1	INDINAVIR		PharmGKB
ABCB1	ABCB1	FENTANYL		PharmGKB
ABCB1	ABCB1	CITALOPRAM		PharmGKB
ABCB1	ABCB1	RIVAROXABAN		PharmGKB
ABCB1	ABCB1	NICOTINE		NCI
ABCB1	ABCB1	DABIGATRAN		PharmGKB

ABCB1	ABCB1	MILTEFOSINE	NCI
ABCB1	ABCB1	FILGRASTIM	NCI
ABCB1	ABCB1	ANASTROZOLE	PharmGKB
ABCB1	ABCB1	CHEMBL203365	DTC
ABCB1	ABCB1	EFAVIRENZ	DTC|PharmGKB
ABCB1	ABCB1	LAMOTRIGINE	PharmGKB
ABCB1	ABCB1	AMPRENAVIR	PharmGKB
ABCB1	ABCB1	WARFARIN	PharmGKB
ABCB1	ABCB1	DAUNORUBICIN	NCI|PharmGKB
ABCB1	ABCB1	LENALIDOMIDE	PharmGKB
ABCB1	ABCB1	VECURONIUM	PharmGKB
ABCB1	ABCB1	CODEINE	DTC|PharmGKB
ABCB1	ABCB1	ASTEMIZOLE	PharmGKB
ABCB1	ABCB1	CYANOCOBALAMIN	PharmGKB
ABCB1	ABCB1	ARIPIPRAZOLE	PharmGKB
ABCB1	ABCB1	SIMVASTATIN	NCI|PharmGKB
ABCB1	ABCB1	OMEPRAZOLE	PharmGKB
ABCB1	ABCB1	DISULFIRAM	PharmGKB
ABCB1	ABCB1	VALSPODAR	PharmGKB
ABCB1	ABCB1	MILTIRONE	DTC
ABCB1	ABCB1	HALOPERIDOL	PharmGKB
ABCB1	ABCB1	SEVOFLURANE	PharmGKB
ABCB1	ABCB1	DRONABINOL	NCI
ABCB1	ABCB1	DESIPRAMINE	PharmGKB
ABCB1	ABCB1	CHEMBL306786	DTC
ABCB1	ABCB1	SCUTELLARIN	DTC
ABCB1	ABCB1	LEVOFLOXACIN	PharmGKB
ABCB1	ABCB1	ETRAVIRINE	DTC
ABCB1	ABCB1	ATORVASTATIN	NCI|PharmGKB
ABCB1	ABCB1	VENLAFAXINE	PharmGKB
ABCB1	ABCB1	MITOXANTRONE	PharmGKB
ABCB1	ABCB1	ACENOCOUMAROL	PharmGKB
ABCB1	ABCB1	TALINOLOL	PharmGKB
ABCB1	ABCB1	ATENOLOL	PharmGKB
ABCB1	ABCB1	CAPECITABINE	PharmGKB
ABCB1	ABCB1	LONAFARNIB	NCI
ABCB1	ABCB1	FLUVOXAMINE	PharmGKB
ABCB1	ABCB1	ISONIAZID	PharmGKB
ABCB1	ABCB1	DILTIAZEM	PharmGKB
ABCB1	ABCB1	DIPYRIDAMOLE	PharmGKB
ABCB1	ABCB1	OXCARBAZEPINE	PharmGKB
ABCB1	ABCB1	TEMSIROLIMUS	PharmGKB
ABCB1	ABCB1	GEMTUZUMAB OZOGAMICIN	NCI
ABCB1	ABCB1	BRECANAVIR	DTC
ABCB1	ABCB1	CHLORPROMAZINE	NCI|PharmGKB
ABCB1	ABCB1	EPOTHILONE D	DTC
ABCB1	ABCB1	VEMURAFENIB	PharmGKB
ABCB1	ABCB1	MELPHALAN	PharmGKB
ABCB1	ABCB1	NIMODIPINE	NCI
ABCB1	ABCB1	2',4'-DIHYDROXYCHALCONE	DTC
ABCB1	ABCB1	SUNITINIB	PharmGKB
ABCB1	ABCB1	FLUOXETINE	PharmGKB
ABCB1	ABCB1	EVEROLIMUS	PharmGKB
ABCB1	ABCB1	METOCHALCONE	DTC
ABCB1	ABCB1	TRABECTEDIN	PharmGKB
ABCB1	ABCB1	MICONAZOLE	NCI
ABCB1	ABCB1	3,4-DIHYDROXYXANTHONE	DTC
ABCB1	ABCB1	PROBENECID	NCI
ABCB1	ABCB1	SAQUINAVIR	PharmGKB
ABCB1	ABCB1	MIFEPRISTONE	PharmGKB
ABCB1	ABCB1	VINDESINE	PharmGKB

ABCB1	ABCB1	CLOZAPINE	PharmGKB
ABCB1	ABCB1	ROCURONIUM	PharmGKB
ABCB1	ABCB1	SERTRALINE	PharmGKB
ABCB1	ABCB1	LOSARTAN	PharmGKB
ABCB1	ABCB1	OLANZAPINE	PharmGKB
ABCB1	ABCB1	TOPOTECAN	PharmGKB
ABCB1	ABCB1	HYDROXOCOBALAMIN	PharmGKB
ABCB1	ABCB1	CHEMBL533293	DTC
ABCB1	ABCB1	GARLIC	NCI
ABCB1	ABCB1	DEXAMETHASONE	PharmGKB
ABCB1	ABCB1	CISPLATIN	PharmGKB
ABCB1	ABCB1	CAPSAICIN	NCI
ABCB1	ABCB1	PERVILLEINE D	DTC
ABCB1	ABCB1	DICLOXACILLIN	PharmGKB
ABCB1	ABCB1	BAFETINIB	CIViC
ABCB1	ABCB1	CERITINIB	CIViC
ABCB1	ABCB1	CRIZOTINIB	CIViC
CP	CP	DEXRAZOXANE	NCI
CP	CP	AMPICILLIN	NCI
CP	CP	PENICILLAMINE	NCI
CP	CP	PROGESTERONE	NCI
CP	CP	VINCRISTINE	NCI
CP	CP	HYDROXYUREA	NCI
CP	CP	NICOTINE	NCI
CP	CP	ESTRADIOL	NCI
CP	CP	ISOFLAVONE	NCI
CP	CP	ISOPROTERENOL	NCI
CP	CP	DIETHYLSTILBESTROL	NCI
CP	CP	DANAZOL	NCI
CP	CP	ANAKINRA	NCI
CP	CP	PROTOPORPHYRIN	NCI
CYP3A5	CYP3A5	HALOPERIDOL	PharmGKB
CYP3A5	CYP3A5	TESMILIFENE	TdgClinicalTrial
CYP3A5	CYP3A5	DIAZEPAM	PharmGKB
CYP3A5	CYP3A5	ROSUVASTATIN	PharmGKB
CYP3A5	CYP3A5	TRAMADOL	PharmGKB
CYP3A5	CYP3A5	BUPRENORPHINE	PharmGKB
CYP3A5	CYP3A5	CILOSTAZOL	PharmGKB
CYP3A5	CYP3A5	APIXABAN	PharmGKB
CYP3A5	CYP3A5	MEPERIDINE	PharmGKB
CYP3A5	CYP3A5	CLARITHROMYCIN	PharmGKB
CYP3A5	CYP3A5	AMLODIPINE	PharmGKB
CYP3A5	CYP3A5	GEFITINIB	NCI
CYP3A5	CYP3A5	MODAFINIL	PharmGKB
CYP3A5	CYP3A5	ZIPRASIDONE	PharmGKB
CYP3A5	CYP3A5	HYDROXYZINE	PharmGKB
CYP3A5	CYP3A5	CITALOPRAM	PharmGKB
CYP3A5	CYP3A5	LAPATINIB	DTC
CYP3A5	CYP3A5	QUETIAPINE	PharmGKB
CYP3A5	CYP3A5	DOCETAXEL	PharmGKB
CYP3A5	CYP3A5	LORATADINE	PharmGKB
CYP3A5	CYP3A5	SIMVASTATIN	NCI|PharmGKB
CYP3A5	CYP3A5	SUFENTANIL	PharmGKB
CYP3A5	CYP3A5	CHLORPHENIRAMINE	PharmGKB
CYP3A5	CYP3A5	CYCLOSPORINE	NCI
CYP3A5	CYP3A5	OLANZAPINE	PharmGKB
CYP3A5	CYP3A5	ATROPINE	NCI
CYP3A5	CYP3A5	SIBUTRAMINE	PharmGKB
CYP3A5	CYP3A5	PREDNISOLONE	NCI
CYP3A5	CYP3A5	TRETINOIN	PharmGKB
CYP3A5	CYP3A5	TRAZODONE	PharmGKB

CYP3A5	CYP3A5	ISOPROPYL ALCOHOL		NCI
CYP3A5	CYP3A5	ESZOPICLONE		PharmGKB
CYP3A5	CYP3A5	VINCRISTINE		PharmGKB
CYP3A5	CYP3A5	ALPRAZOLAM		PharmGKB
CYP3A5	CYP3A5	HYDROCODONE		PharmGKB
CYP3A5	CYP3A5	BUSPIRONE		PharmGKB
CYP3A5	CYP3A5	MONTELUKAST		PharmGKB
CYP3A5	CYP3A5	OMEPRAZOLE		PharmGKB
CYP3A5	CYP3A5	ESOMEPRAZOLE		PharmGKB
CYP3A5	CYP3A5	PIOGLITAZONE		PharmGKB
CYP3A5	CYP3A5	ONDANSETRON		PharmGKB
CYP3A5	CYP3A5	RITONAVIR	inhibitor	GuideToPharmacology|PharmGKB
CYP3A5	CYP3A5	PROPOXYPHENE		PharmGKB
CYP3A5	CYP3A5	MEDROXYPROGESTERONE ACETATE		PharmGKB
CYP3A5	CYP3A5	BOCEPREVIR		PharmGKB
CYP3A5	CYP3A5	NIFEDIPINE		PharmGKB
CYP3A5	CYP3A5	MIRTAZAPINE		PharmGKB
CYP3A5	CYP3A5	MELOXICAM		PharmGKB
CYP3A5	CYP3A5	AMIODARONE		PharmGKB
CYP3A5	CYP3A5	PAROXETINE		PharmGKB
CYP3A5	CYP3A5	PRASUGREL		PharmGKB|FDA
CYP3A5	CYP3A5	SERTRALINE		PharmGKB
CYP3A5	CYP3A5	TERAZOSIN		PharmGKB
CYP3A5	CYP3A5	NEVIRAPINE		PharmGKB
CYP3A5	CYP3A5	ISOTRETINOIN		PharmGKB
CYP3A5	CYP3A5	PANTOPRAZOLE		PharmGKB
CYP3A5	CYP3A5	PACLITAXEL		NCI|PharmGKB
CYP3A5	CYP3A5	ZOLPIDEM		PharmGKB
CYP3A5	CYP3A5	ATORVASTATIN		PharmGKB
CYP3A5	CYP3A5	CARBAMAZEPINE		NCI|PharmGKB
CYP3A5	CYP3A5	PHENYTOIN		NCI
CYP3A5	CYP3A5	ARTEMETHER		PharmGKB
CYP3A5	CYP3A5	RIFAMPIN		DTC|PharmGKB
CYP3A5	CYP3A5	GRANISETRON		PharmGKB
CYP3A5	CYP3A5	LOVASTATIN		PharmGKB
CYP3A5	CYP3A5	MIDAZOLAM		PharmGKB
CYP3A5	CYP3A5	FLUOXETINE		PharmGKB
CYP3A5	CYP3A5	DOLUTEGRAVIR		PharmGKB
CYP3A5	CYP3A5	CLOPIDOGREL		DTC|PharmGKB
CYP3A5	CYP3A5	CLINDAMYCIN		PharmGKB
CYP3A5	CYP3A5	AMPRENAVIR		NCI
CYP3A5	CYP3A5	DABIGATRAN		PharmGKB
CYP3A5	CYP3A5	EFAVIRENZ		PharmGKB
CYP3A5	CYP3A5	DONEPEZIL		PharmGKB
CYP3A5	CYP3A5	CARBOPLATIN		PharmGKB
CYP3A5	CYP3A5	TAMSULOSIN		PharmGKB
CYP3A5	CYP3A5	CELECOXIB		PharmGKB
CYP3A5	CYP3A5	TADALAFIL		PharmGKB
CYP3A5	CYP3A5	LOSARTAN		PharmGKB
CYP3A5	CYP3A5	AXITINIB		PharmGKB
CYP3A5	CYP3A5	NIMODIPINE		PharmGKB
CYP3A5	CYP3A5	SIROLIMUS		PharmGKB
CYP3A5	CYP3A5	FENTANYL		PharmGKB
CYP3A5	CYP3A5	CYCLOBENZAPRINE		PharmGKB
CYP3A5	CYP3A5	FENOFIBRATE		PharmGKB
CYP3A5	CYP3A5	EVEROLIMUS		PharmGKB
CYP3A5	CYP3A5	HYDROMORPHONE		PharmGKB
CYP3A5	CYP3A5	LUMEFANTRINE		PharmGKB
CYP3A5	CYP3A5	PROGESTERONE		PharmGKB
CYP3A5	CYP3A5	OXYCODONE		PharmGKB
CYP3A5	CYP3A5	VARDENAFIL		PharmGKB

CYP3A5	CYP3A5	LANSOPRAZOLE		PharmGKB
CYP3A5	CYP3A5	SUNITINIB		PharmGKB
CYP3A5	CYP3A5	LOPINAVIR		PharmGKB
CYP3A5	CYP3A5	COBICISTAT	inhibitor	ChemblInteractions
CYP3A5	CYP3A5	ERYTHROMYCIN		PharmGKB
CYP3A5	CYP3A5	VENLAFAXINE		PharmGKB
CYP3A5	CYP3A5	FLUVASTATIN		PharmGKB
CYP3A5	CYP3A5	SALMETEROL		PharmGKB
CYP3A5	CYP3A5	TRIAZOLAM		PharmGKB
CYP3A5	CYP3A5	IMATINIB		PharmGKB
CYP3A5	CYP3A5	WARFARIN		PharmGKB
CYP3A5	CYP3A5	SILDENAFIL		PharmGKB
MYC	MYC	GLUTAMINE		NCI
MYC	MYC	AN-9		NCI
MYC	MYC	PROTOPORPHYRIN		NCI
MYC	MYC	HYPOXANTHINE		NCI
MYC	MYC	ADOZELESIN		NCI
MYC	MYC	CISPLATIN		CIViC
MYC	MYC	MITOGUAZONE		NCI
MYC	MYC	ALISERTIB		ClearityFoundationBiomarkers
MYC	MYC	OLAPARIB		CIViC
MYC	MYC	BROMOCRIPTINE		NCI
MYC	MYC	CHEMBL35482		NCI
MYC	MYC	ANTIBIOTIC		NCI
MYC	MYC	CETUXIMAB		NCI
MYC	MYC	THIOGUANINE		NCI
MYC	MYC	INDOMETHACIN		NCI
MYC	MYC	DIETHYLSTILBESTROL		NCI
MYC	MYC	AZACITIDINE		NCI
MYC	MYC	IMATINIB		NCI
MYC	MYC	VORINOSTAT		NCI
MYC	MYC	DINACICLIB		ClearityFoundationBiomarkers
MYC	MYC	TEMOZOLOMIDE		CGI
MYC	MYC	QUINAPRIL		NCI
MYC	MYC	PREXASERTIB		CIViC
MYC	MYC	ACIVICIN		NCI
MYC	MYC	TROGLITAZONE		NCI
MYC	MYC	LITHIUM		NCI
MYC	MYC	AMINOPTERIN		NCI
MYC	MYC	AMG-900		ClearityFoundationBiomarkers
MYC	MYC	MELATONIN		NCI
MYC	MYC	THROMBIN		NCI
MYC	MYC	AMIFOSTINE		NCI
MYC	MYC	ESTRONE		NCI
MYC	MYC	METHYLPREDNISOLONE		NCI
MYC	MYC	CALCITRIOL		NCI
MYC	MYC	NOVOBIOCIN		NCI
MYC	MYC	VERAPAMIL		NCI
MYC	MYC	THYROTROPIN		NCI
MYC	MYC	LOBAPLATIN		NCI
MYC	MYC	SULINDAC		NCI
MYC	MYC	BIZELESIN		NCI
MYC	MYC	IBRUTINIB		CIViC
MT1F	MT1F	DEXAMETHASONE		NCI
MT1F	MT1F	CISPLATIN		NCI
DAO	DAO	CHEMBL492591		DTC
DAO	DAO	3-HYDROXYCOUMARIN		DTC
DAO	DAO	BENZOIC ACID		DTC
DAO	DAO	CHEMBL164585		DTC
GUCA2B	GUCA2B	VOLANESORSEN SODIUM		NCI
TPSAB1	TPSAB1	GABEXATE	inhibitor	GuideToPharmacology

TPSAB1	TPSAB1	PENTAMIDINE		TTD
TPSAB1	TPSAB1	CHEMBL256892	inhibitor	GuideToPharmacology
TPSAB1	TPSAB1	THROMBIN		NCI
TPSAB1	TPSAB1	NAFAMOSTAT	inhibitor	GuideToPharmacology
TPSAB1	TPSAB1	REGRAMOSTIM		NCI
COL1A1	COL1A1	COLLAGENASE CLOSTRIDIUM HISTOLYTICUM		ChemblInteractions|TEND
COL1A1	COL1A1	OCRIPLASMIN		ChemblInteractions
CDH1	CDH1	VOLASERTIB		JAX-CKB
CDH1	CDH1	ERLOTINIB		JAX-CKB
CDH1	CDH1	SELUMETINIB		JAX-CKB
CDH1	CDH1	BICALUTAMIDE		CGI
CDH1	CDH1	CAPECITABINE		JAX-CKB
CDH1	CDH1	LAPATINIB		JAX-CKB
ANGPTL3	ANGPTL3	VUPANORSEN		TTD
ANGPTL3	ANGPTL3	EVINACUMAB		TTD
GPR183	GPR183	CHEMBL169046	agonist	GuideToPharmacology
ST14	ST14	SURAMIN		NCI
KIT	KIT	OSI-930	inhibitor	ChemblInteractions|GuideToPharmacology|TTD
KIT	KIT	SORAFENIB	antagonist|inhibitor	TALC|MyCancerGenome|TdgClinicalTrial|JAX-CKB|CGI|TEND|DoCM|CIViC|GuideToPharmacology|PharmGKB|TTD|OncoKB
KIT	KIT	XL-820	inhibitor	ChemblInteractions|TTD
KIT	KIT	TANDUTINIB	inhibitor	DTC|MyCancerGenome|ChemblInteractions|GuideToPharmacology
KIT	KIT	SITRAVATINIB	inhibitor	GuideToPharmacology
KIT	KIT	PEXIDARTINIB	inhibitor	MyCancerGenome|JAX-CKB|ChemblInteractions|GuideToPharmacology|CancerCommons|TTD
KIT	KIT	MIDOSTAURIN	antagonist|inhibitor	MyCancerGenome|JAX-CKB|ChemblInteractions|CIViC|PharmGKB|FDA
KIT	KIT	CHEMBL406375	inhibitor	GuideToPharmacology
KIT	KIT	SU-014813	inhibitor	ChemblInteractions|GuideToPharmacology
KIT	KIT	MOTESANIB	inhibitor	TALC|MyCancerGenome|TdgClinicalTrial|JAX-CKB|ChemblInteractions
KIT	KIT	ENTRECTINIB		DTC
KIT	KIT	DASATINIB	antagonist|inhibitor	TALC|TdgClinicalTrial|JAX-CKB|ChemblInteractions|CGI|TEND|DoCM|CIViC|CancerCommons|MyCancerGenomeClinicalTrial
KIT	KIT	REGORAFENIB	inhibitor	TALC|MyCancerGenome|JAX-CKB|ChemblInteractions|CGI|CIViC|MyCancerGenomeClinicalTrial|TTD|OncoKB
KIT	KIT	IMATINIB	inhibitor|antagonist|multitarget	TALC|MyCancerGenome|TdgClinicalTrial|JAX-CKB|CGI|TEND|DoCM|COSMIC|CIViC|PharmGKB|TTD|FDA|OncoKB
KIT	KIT	ENMD-2076	inhibitor	TdgClinicalTrial|ChemblInteractions
KIT	KIT	BARASERTIB		DTC
KIT	KIT	QUIZARTINIB	inhibitor	JAX-CKB|ChemblInteractions|DoCM|GuideToPharmacology
KIT	KIT	AKN-028	inhibitor	GuideToPharmacology
KIT	KIT	SUNITINIB	inhibitor	TALC|MyCancerGenome|TdgClinicalTrial|JAX-CKB|CGI|TEND|DoCM|COSMIC|CIViC|GuideToPharmacology|MyCancerGenom
KIT	KIT	CENISERTIB	inhibitor	TALC|DTC|MyCancerGenome
KIT	KIT	SUNITINIB MALATE	inhibitor	ChemblInteractions
KIT	KIT	AXITINIB	inhibitor	TALC|MyCancerGenome|TdgClinicalTrial|CancerCommons
KIT	KIT	CHEMBL578061		DTC
KIT	KIT	SEMAXANIB	inhibitor	ChemblInteractions|GuideToPharmacology
KIT	KIT	PAZOPANIB HYDROCHLORIDE	inhibitor	ChemblInteractions
KIT	KIT	CEDIRANIB	inhibitor	TALC|ChemblInteractions|GuideToPharmacology
KIT	KIT	GW843682X		DTC
KIT	KIT	VATALANIB	inhibitor	TALC|DTC|TdgClinicalTrial|ChemblInteractions
KIT	KIT	AST-487	inhibitor	DTC|GuideToPharmacology
KIT	KIT	MASITINIB	inhibitor	MyCancerGenome|TdgClinicalTrial|ChemblInteractions|GuideToPharmacology|CancerCommons|MyCancerGenomeClinicalTı
KIT	KIT	PAZOPANIB	inhibitor	TALC|MyCancerGenome|GuideToPharmacology|MyCancerGenomeClinicalTrial
KIT	KIT	ILORASERTIB	inhibitor	DTC|GuideToPharmacology
KIT	KIT	LINIFANIB	inhibitor	TdgClinicalTrial|GuideToPharmacology
KIT	KIT	CHEMBL1908396	inhibitor	GuideToPharmacology
KIT	KIT	CABOZANTINIB	inhibitor	MyCancerGenome|JAX-CKB
KIT	KIT	PD-0325901		JAX-CKB
KIT	KIT	CHEMBL541400		DTC
KIT	KIT	TELATINIB	inhibitor	TALC|MyCancerGenome|ChemblInteractions
KIT	KIT	FLUMATINIB	inhibitor	GuideToPharmacology
KIT	KIT	CHEMBL1908395	inhibitor	GuideToPharmacology
KIT	KIT	JNJ-7706621		DTC
KIT	KIT	NILOTINIB	antagonist|inhibitor	JAX-CKB|CGI|COSMIC|CIViC|CancerCommons|MyCancerGenomeClinicalTrial
KIT	KIT	PONATINIB	inhibitor	DTC|JAX-CKB|CGI|CIViC|TTD
KIT	KIT	CYC-116		DTC

KIT	KIT	DOVITINIB	inhibitor	DTC|ClearityFoundationClinicalTrial|ChemblInteractions|GuideToPharmacology
KIT	KIT	MK-2206		JAX-CKB
KIT	KIT	BEVACIZUMAB		JAX-CKB
KIT	KIT	LENVATINIB	inhibitor	TdgClinicalTrial
KIT	KIT	RIVOCERANIB	inhibitor	GuideToPharmacology
KIT	KIT	AVAPRITINIB	inhibitor	JAX-CKB|GuideToPharmacology|TTD|OncoKB
KIT	KIT	FAMITINIB	inhibitor	ChemblInteractions|GuideToPharmacology|TTD
KIT	KIT	TRAMETINIB		JAX-CKB
KIT	KIT	HENATINIB	inhibitor	GuideToPharmacology
KIT	KIT	FORETINIB	inhibitor	ChemblInteractions
KIT	KIT	PEMBROLIZUMAB		JAX-CKB
KIT	KIT	AMG-191		TTD
KIT	KIT	IMATINIB MESYLATE	inhibitor	ChemblInteractions|TTD
KIT	KIT	ANLOTINIB	inhibitor	ChemblInteractions
KIT	KIT	XL-999	inhibitor	ChemblInteractions
KIT	KIT	CYTARABINE		JAX-CKB
KIT	KIT	LUCITANIB	inhibitor	GuideToPharmacology
KIT	KIT	INFIGRATINIB		JAX-CKB
KIT	KIT	CM-082	inhibitor	ChemblInteractions
KIT	KIT	ROMIPLOSTIM		TTD
KIT	KIT	AMUVATINIB	inhibitor	TALC|MyCancerGenome|TdgClinicalTrial|ChemblInteractions
KIT	KIT	SP-600125		DTC
KIT	KIT	CRENOLANIB	inhibitor	GuideToPharmacology
KIT	KIT	RIPRETINIB	inhibitor	JAX-CKB|GuideToPharmacology|TTD
KIT	KIT	SNS-314		DTC
KIT	KIT	EDICOTINIB	inhibitor	GuideToPharmacology
KIT	KIT	SORAFENIB TOSYLATE	inhibitor	ChemblInteractions
KIT	KIT	RG-1530	inhibitor	DTC|GuideToPharmacology
NECTIN4	NECTIN4	ENFORTUMAB VEDOTIN	antibody	TALC|TdgClinicalTrial|TTD
HRH2	HRH2	CONESSINE	antagonist	GuideToPharmacology
HRH2	HRH2	RANITIDINE	antagonist	TdgClinicalTrial|TEND|GuideToPharmacology|TTD
HRH2	HRH2	NIZATIDINE	antagonist	TdgClinicalTrial|ChemblInteractions|TEND|TTD
HRH2	HRH2	HISTAMINE	agonist	TdgClinicalTrial
HRH2	HRH2	ARPROMIDINE	agonist	GuideToPharmacology
HRH2	HRH2	ASENAPINE	antagonist	TdgClinicalTrial
HRH2	HRH2	MK-0249	antagonist	GuideToPharmacology
HRH2	HRH2	DOXEPIN	antagonist	TdgClinicalTrial
HRH2	HRH2	FAMOTIDINE	antagonist	TdgClinicalTrial|ChemblInteractions|TEND|GuideToPharmacology|TTD
HRH2	HRH2	CIMETIDINE	antagonist	TdgClinicalTrial|ChemblInteractions|TEND|GuideToPharmacology|TTD
HRH2	HRH2	CHEMBL472273	agonist	GuideToPharmacology
HRH2	HRH2	AMTHAMINE	agonist	GuideToPharmacology
HRH2	HRH2	TOLAZOLINE HYDROCHLORIDE	agonist	ChemblInteractions
HRH2	HRH2	IMPROMIDINE	agonist	GuideToPharmacology
HRH2	HRH2	CIPROXIFAN	antagonist	GuideToPharmacology
HRH2	HRH2	BURIMAMIDE	agonist	GuideToPharmacology
HRH2	HRH2	METHANTHELINE BROMIDE	antagonist	ChemblInteractions
HRH2	HRH2	BETAZOLE HYDROCHLORIDE	agonist	ChemblInteractions
HRH2	HRH2	BETAZOLE	agonist	TdgClinicalTrial|TEND|TTD
HRH2	HRH2	EPINASTINE	antagonist	TdgClinicalTrial|TEND
HRH2	HRH2	RANITIDINE HYDROCHLORIDE	antagonist	ChemblInteractions
HRH2	HRH2	PITOLISANT	antagonist	GuideToPharmacology
HRH2	HRH2	CHEMBL351231	antagonist	GuideToPharmacology
HRH2	HRH2	CLOBENPROPIT	antagonist	GuideToPharmacology
HRH2	HRH2	CIMETIDINE HYDROCHLORIDE	antagonist	ChemblInteractions
HRH2	HRH2	RANITIDINE BISMUTH CITRATE	antagonist	ChemblInteractions
IL2RG	IL2RG	DENILEUKIN DIFTITOX		ChemblInteractions
IL2RG	IL2RG	ALDESLEUKIN	agonist	TdgClinicalTrial|ChemblInteractions|TEND
IL2RG	IL2RG	DACLIZUMAB	inhibitor	ChemblInteractions
IL2RG	IL2RG	ALDESLEUKIN	agonist	TdgClinicalTrial
IL2RG	IL2RG	BASILIXIMAB	inhibitor	ChemblInteractions
SLC28A1	SLC28A1	GEMCITABINE		PharmGKB

SLC28A1	SLC28A1	ADENOSINE		DTC
SLC28A1	SLC28A1	ZIDOVUDINE		PharmGKB
SLC28A1	SLC28A1	THYMIDINE		NCI
SLC28A1	SLC28A1	URIDINE		DTC|NCI
HAVCR2	HAVCR2	LY-3321367		TTD
MMP9	MMP9	CELECOXIB		PharmGKB
MMP9	MMP9	MARIMASTAT	inhibitor	TdgClinicalTrial|TEND|GuideToPharmacology
MMP9	MMP9	ANDECALIXIMAB	antibody|inhibitor	ChemblInteractions|GuideToPharmacology|TTD
MMP9	MMP9	ILOMASTAT	inhibitor	GuideToPharmacology
MMP9	MMP9	DEMETHYLWEDELOLACTONE		DTC
MMP9	MMP9	CURCUMIN		TTD
MMP9	MMP9	PRINOMASTAT	vaccine	TALC
MMP9	MMP9	CGS-27023A	inhibitor	GuideToPharmacology
MMP9	MMP9	TOZULERISTIDE		TTD
MMP9	MMP9	AZD-1236	inhibitor	GuideToPharmacology
MMP9	MMP9	BEVACIZUMAB		CIViC
MMP9	MMP9	S-3304	vaccine	TALC
MMP9	MMP9	INCYCLINIDE		TdgClinicalTrial
MMP9	MMP9	CURCUMIN PYRAZOLE		DTC
MMP9	MMP9	CARBOXYLATED GLUCOSAMINE		DTC
SLCO2B1	SLCO2B1	ROSUVASTATIN		PharmGKB
SLCO2B1	SLCO2B1	VERLUKAST	inhibitor	GuideToPharmacology
SLCO2B1	SLCO2B1	ISOSILYBIN B		DTC
SLCO2B1	SLCO2B1	ELTROMBOPAG		DTC
SLCO2B1	SLCO2B1	SILDENAFIL	inhibitor	GuideToPharmacology
SLCO2B1	SLCO2B1	SILIBININ		DTC
SLCO2B1	SLCO2B1	FLUVASTATIN		PharmGKB
SLCO2B1	SLCO2B1	SULFOBROMOPHTHALEIN		DTC
SLCO2B1	SLCO2B1	GLYBURIDE	inhibitor	GuideToPharmacology
SLCO2B1	SLCO2B1	ISOSILYBIN A		DTC
SLCO2B1	SLCO2B1	ERLOTINIB	inhibitor	GuideToPharmacology
SLCO2B1	SLCO2B1	SCUTELLARIN		DTC
SLCO2B1	SLCO2B1	PRAVASTATIN		PharmGKB
SLCO2B1	SLCO2B1	ROSIGLITAZONE		NCI
SLCO2B1	SLCO2B1	ATORVASTATIN		PharmGKB
SLCO2B1	SLCO2B1	SILICRISTIN		DTC
SLCO2B1	SLCO2B1	FEXOFENADINE		PharmGKB
SLCO2B1	SLCO2B1	SIMVASTATIN		PharmGKB
SLCO2B1	SLCO2B1	SILYBIN B		DTC
SLCO2B1	SLCO2B1	MONTELUKAST		PharmGKB
SLCO2B1	SLCO2B1	LOVASTATIN		PharmGKB
SLCO2B1	SLCO2B1	GEMFIBROZIL	inhibitor	GuideToPharmacology
SLCO2B1	SLCO2B1	SILYDIANIN		DTC
CD36	CD36	ABT-510		TdgClinicalTrial
GJA1	GJA1	BLEOMYCIN		NCI
GJA1	GJA1	CARVEDILOL	other/unknown	NCI
GJA1	GJA1	CARBENOXOLONE	inhibitor	GuideToPharmacology
GJA1	GJA1	OCTANOL	inhibitor	GuideToPharmacology
GJA1	GJA1	EPIGALOCATECHIN GALLATE		NCI
GJA1	GJA1	PROPYLTHIOURACIL		NCI
GJA1	GJA1	LABETALOL		NCI
GJA1	GJA1	ATENOLOL		NCI
RGS5	RGS5	IRINOTECAN		PharmGKB
RGS5	RGS5	BEVACIZUMAB		PharmGKB
RGS5	RGS5	FLUOROURACIL		PharmGKB
RGS5	RGS5	LEUCOVORIN		PharmGKB
CD248	CD248	ONTUXIZUMAB	inhibitor	TdgClinicalTrial|ChemblInteractions|TTD
WT1	WT1	CYTARABINE		CIViC
WT1	WT1	DIMETHYL SULFOXIDE		NCI
WT1	WT1	HALOFUGINONE		NCI
WT1	WT1	SIROLIMUS		NCI

WT1	WT1	CURCUMIN		NCI
WT1	WT1	DEFEROXAMINE		NCI
WT1	WT1	DAUNORUBICIN		CIViC
SLC39A14	SLC39A14	NORTRIPTYLINE		PharmGKB
ANO1	ANO1	CROFELEMER	blocker|antagonist|inhibitor	ChemblInteractions|GuideToPharmacology
ANO1	ANO1	MIBEFRADIL	blocker	GuideToPharmacology
ANO1	ANO1	FLUOXETINE	blocker	GuideToPharmacology
PROC	PROC	AVATROMBOPAG		PharmGKB|FDA
PROC	PROC	PROGESTERONE		FDA
PROC	PROC	ANCROD		TTD
PROC	PROC	WARFARIN		FDA
PROC	PROC	LUSUTROMBOPAG		PharmGKB|FDA
PROC	PROC	MENADIONE	activator	TEND
PROC	PROC	ESTRADIOL		FDA
PROC	PROC	PHENPROCOUMON		PharmGKB
BDKRB2	BDKRB2	ICATIBANT	antagonist	TdgClinicalTrial|TEND|GuideToPharmacology|TTD
BDKRB2	BDKRB2	LISINOPRIL		PharmGKB
BDKRB2	BDKRB2	ANATIBANT	antagonist	TdgClinicalTrial|ChemblInteractions|GuideToPharmacology|TTD
BDKRB2	BDKRB2	BRADYKININ	agonist	GuideToPharmacology|TTD
BDKRB2	BDKRB2	ATORVASTATIN		PharmGKB
BDKRB2	BDKRB2	DELTIBANT		TTD
BDKRB2	BDKRB2	ICATIBANT ACETATE	antagonist	ChemblInteractions
BDKRB2	BDKRB2	LABRADIMIL	agonist	ChemblInteractions|GuideToPharmacology
BDKRB2	BDKRB2	ENALAPRIL		PharmGKB
BDKRB2	BDKRB2	VOLANESORSEN SODIUM		NCI
BDKRB2	BDKRB2	CHEMBL446325	antagonist	GuideToPharmacology
BDKRB2	BDKRB2	FASITIBANT CHLORIDE	antagonist	ChemblInteractions
BDKRB2	BDKRB2	IMIDAPRIL		PharmGKB
NGF	NGF	MEDI-578		TTD
NGF	NGF	TANEZUMAB	inhibitor	TdgClinicalTrial|ChemblInteractions|TTD
NGF	NGF	FASINUMAB	inhibitor	ChemblInteractions|TTD
NGF	NGF	METHADONE		PharmGKB
NGF	NGF	FULRANUMAB	inhibitor	ChemblInteractions
PLG	PLG	UROKINASE	activator	TdgClinicalTrial|ChemblInteractions|TEND
PLG	PLG	ANISTREPLASE	activator	TdgClinicalTrial|TEND|TTD
PLG	PLG	ALTEPLASE	activator	TdgClinicalTrial|ChemblInteractions|NCI|TEND|TTD
PLG	PLG	DESMOTEPLASE	activator	TdgClinicalTrial|ChemblInteractions|TTD
PLG	PLG	NYSTATIN		NCI
PLG	PLG	APROTININ	inhibitor	ChemblInteractions|GuideToPharmacology
PLG	PLG	RETEPLASE	activator	TdgClinicalTrial|ChemblInteractions|TEND|TTD
PLG	PLG	BORTEZOMIB		DTC
PLG	PLG	DANAZOL		NCI
PLG	PLG	CHEMBL35482		NCI
PLG	PLG	AMEDIPLASE		TdgClinicalTrial
PLG	PLG	STREPTOKINASE	activator	TdgClinicalTrial|ChemblInteractions|TEND|TTD
PLG	PLG	PREDNISOLONE		NCI
PLG	PLG	TENECTEPLASE	activator	TdgClinicalTrial|ChemblInteractions|TEND|TTD
PLG	PLG	NORETHINDRONE		NCI
PLG	PLG	RADIOSUMIN B		DTC
PLG	PLG	RANOLAZINE		TTD
PLG	PLG	AMINOCAPROIC ACID	inhibitor	ChemblInteractions
PLG	PLG	LEPIRUDIN		NCI
PLG	PLG	COUMARIN		NCI
PLG	PLG	INOSITOL		NCI
PLG	PLG	TRANEXAMIC ACID	inhibitor	ChemblInteractions
PLG	PLG	GARLIC		NCI
PLG	PLG	GENISTEIN		NCI
PLG	PLG	HUMAN GROWTH HORMONE		NCI
PLG	PLG	DEFIBROTIDE SODIUM	activator	ChemblInteractions
PLG	PLG	FIBRINOLYSIN, HUMAN		TdgClinicalTrial
PLG	PLG	OXYMETHOLONE		NCI

PLG	PLG	MELAGATRAN		TTD
FLT1	FLT1	DOVITINIB	inhibitor	DTC|TdgClinicalTrial|ClearityFoundationClinicalTrial|ChemblInteractions|GuideToPharmacology|CancerCommons
FLT1	FLT1	VANDETANIB	inhibitor	DTC|TdgClinicalTrial|ClearityFoundationClinicalTrial|ChemblInteractions|MyCancerGenomeClinicalTrial
FLT1	FLT1	NINTEDANIB	inhibitor	TALC|MyCancerGenome|TdgClinicalTrial|ClearityFoundationClinicalTrial|GuideToPharmacology
FLT1	FLT1	AXITINIB	inhibitor	TALC|MyCancerGenome|TdgClinicalTrial|ChemblInteractions|CancerCommons|MyCancerGenomeClinicalTrial|PharmGKB
FLT1	FLT1	LINIFANIB	inhibitor	DTC|MyCancerGenome|TdgClinicalTrial|ChemblInteractions|GuideToPharmacology
FLT1	FLT1	L-21649	inhibitor	ChemblInteractions
FLT1	FLT1	REGORAFENIB	inhibitor	TALC|MyCancerGenome|ChemblInteractions|MyCancerGenomeClinicalTrial
FLT1	FLT1	SUNITINIB	inhibitor	TALC|DTC|TdgClinicalTrial|CGI|TEND|MyCancerGenomeClinicalTrial|PharmGKB
FLT1	FLT1	SORAFENIB	inhibitor	DTC|MyCancerGenome|MyCancerGenomeClinicalTrial|PharmGKB
FLT1	FLT1	PAZOPANIB	inhibitor	TALC|DTC|MyCancerGenome|TdgClinicalTrial|GuideToPharmacology|MyCancerGenomeClinicalTrial
FLT1	FLT1	TESEVATINIB	inhibitor	MyCancerGenome|TdgClinicalTrial
FLT1	FLT1	CEDIRANIB	inhibitor	TALC|DTC|TdgClinicalTrial|ClearityFoundationClinicalTrial|ChemblInteractions|GuideToPharmacology|CancerCommons
FLT1	FLT1	TELATINIB		TdgClinicalTrial
FLT1	FLT1	SUNITINIB MALATE	inhibitor	ChemblInteractions
FLT1	FLT1	SEMAXANIB	inhibitor	ChemblInteractions|GuideToPharmacology
FLT1	FLT1	TIVOZANIB	inhibitor	TALC|MyCancerGenome|TdgClinicalTrial|ClearityFoundationClinicalTrial|ChemblInteractions|GuideToPharmacology|TTD
FLT1	FLT1	MOTESANIB	inhibitor	TALC|MyCancerGenome|TdgClinicalTrial|ChemblInteractions|GuideToPharmacology|PharmGKB
FLT1	FLT1	AG-13958	inhibitor	ChemblInteractions
FLT1	FLT1	PEGPLERANIB SODIUM		TdgClinicalTrial
FLT1	FLT1	TG100-801	inhibitor	ChemblInteractions
FLT1	FLT1	ENMD-2076	inhibitor	MyCancerGenome
FLT1	FLT1	PF-00562271		DTC
FLT1	FLT1	CHEMBL1997335		DTC
FLT1	FLT1	FAMITINIB	inhibitor	ChemblInteractions|TTD
FLT1	FLT1	LUCITANIB	inhibitor	ChemblInteractions|GuideToPharmacology|MyCancerGenomeClinicalTrial
FLT1	FLT1	FRUQUINTINIB	inhibitor	TdgClinicalTrial|ChemblInteractions|GuideToPharmacology|TTD
FLT1	FLT1	SURUFATINIB	inhibitor	ChemblInteractions|TTD
FLT1	FLT1	JNJ-7706621		DTC
FLT1	FLT1	RG-1530	inhibitor	DTC|ChemblInteractions|GuideToPharmacology
FLT1	FLT1	PEXIDARTINIB	inhibitor	GuideToPharmacology
FLT1	FLT1	AZD-1152-HQPA		DTC
FLT1	FLT1	ICRUCUMAB	antagonist	ChemblInteractions
FLT1	FLT1	CEP-5214	inhibitor	DTC|ChemblInteractions
FLT1	FLT1	BRIVANIB	inhibitor	ChemblInteractions|GuideToPharmacology
FLT1	FLT1	ILORASERTIB	inhibitor	DTC|ChemblInteractions|GuideToPharmacology
FLT1	FLT1	GLESATINIB	inhibitor	TALC|MyCancerGenome|TdgClinicalTrial
FLT1	FLT1	CHIAURANIB	inhibitor	ChemblInteractions
FLT1	FLT1	GO-6976		DTC
FLT1	FLT1	BMS-690514	inhibitor	ChemblInteractions
FLT1	FLT1	VATALANIB	inhibitor	TALC|DTC|MyCancerGenome|TdgClinicalTrial|ChemblInteractions|GuideToPharmacology
FLT1	FLT1	CEP-11981	inhibitor	ChemblInteractions|GuideToPharmacology|TTD
FLT1	FLT1	XL-999	inhibitor	ChemblInteractions
FLT1	FLT1	R-406		DTC
FLT1	FLT1	XL-820	inhibitor	ChemblInteractions
FLT1	FLT1	DORSOMORPHIN	inhibitor	GuideToPharmacology
FLT1	FLT1	X-82	inhibitor	MyCancerGenome|ChemblInteractions|TTD
FLT1	FLT1	ENTRECTINIB		DTC
FLT1	FLT1	SP-600125		DTC
FLT1	FLT1	TAK-593	inhibitor	ChemblInteractions
FLT1	FLT1	SITRAVATINIB	inhibitor	GuideToPharmacology
FLT1	FLT1	TOZASERTIB		DTC
FLT1	FLT1	MK-2461	inhibitor	GuideToPharmacology
FLT1	FLT1	PEXMETINIB	inhibitor	GuideToPharmacology
FLT1	FLT1	OSI-930	inhibitor	ChemblInteractions|GuideToPharmacology
FLT1	FLT1	CYC-116		DTC
FLT1	FLT1	RABEPRAZOLE SODIUM	inhibitor	MyCancerGenome|TTD
FLT1	FLT1	CENISERTIB		DTC
FLT1	FLT1	ABICIPAR PEGOL		TdgClinicalTrial
FLT1	FLT1	SU-014813	inhibitor	ChemblInteractions|GuideToPharmacology
FLT1	FLT1	SORAFENIB TOSYLATE	inhibitor	ChemblInteractions

FLT1	FLT1	LENVATINIB	inhibitor	TALC|TdgClinicalTrial
FLT1	FLT1	TAE-684		DTC
FLT1	FLT1	TELBERMIN	agonist	ChemblInteractions
FLT1	FLT1	DORAMAPIMOD		DTC
FLT1	FLT1	MGCD-265	inhibitor	ChemblInteractions
FLT1	FLT1	PAZOPANIB HYDROCHLORIDE	inhibitor	ChemblInteractions
FLT1	FLT1	FORETINIB	inhibitor	MyCancerGenome|ChemblInteractions|GuideToPharmacology
FLT1	FLT1	CP-459632	inhibitor	ChemblInteractions
FLT1	FLT1	GW843682X		DTC
FLT1	FLT1	VEGFRECINE		DTC
FLT1	FLT1	KRN-633	inhibitor	ChemblInteractions
FLT1	FLT1	LENVATINIB MESYLATE	inhibitor	ChemblInteractions
FLT1	FLT1	BRIVANIB ALANINATE	inhibitor	ChemblInteractions
FLT1	FLT1	ORANTINIB	inhibitor	MyCancerGenome
FLT1	FLT1	4SC-203	inhibitor	ChemblInteractions
FLT1	FLT1	OSI-632		DTC
FLT1	FLT1	KENPAULLONE		DTC
FLT1	FLT1	AEE-788	inhibitor	ChemblInteractions
FLT1	FLT1	ALISERTIB		DTC
FLT1	FLT1	AST-487		DTC
FLT1	FLT1	MUPARFOSTAT		TdgClinicalTrial
FLT1	FLT1	CEP-7055	inhibitor	ChemblInteractions
FLT1	FLT1	ERLOTINIB		DTC
FLT1	FLT1	NINTEDANIB ESYLATE	inhibitor	ChemblInteractions
FLT1	FLT1	PD-0166285		DTC
TPSB2	TPSB2	VOLANESORSEN SODIUM		NCI
TPSB2	TPSB2	HEPARIN		NCI
DSP	DSP	ENALAPRIL		NCI
LAMC2	LAMC2	OCRIPLASMIN		ChemblInteractions
PAH	PAH	SAPROPTERIN		TdgClinicalTrial|TEND
PAH	PAH	SAPROPTERIN DIHYDROCHLORIDE	activator	ChemblInteractions
PAH	PAH	CARBOCYSTEINE		PharmGKB
PAH	PAH	SAPROPTERIN	activator|cofactor	TdgClinicalTrial|TEND|GuideToPharmacology
PAH	PAH	FENCLONINE	inhibitor	GuideToPharmacology
PAH	PAH	PEGVALIASE		TTD
CYBB	CYBB	APIGENIN		DTC
CYBB	CYBB	CHRYSIN		DTC
CYBB	CYBB	LUTEOLIN		DTC
F2RL3	F2RL3	RUSALATIDE		TdgClinicalTrial
F2RL3	F2RL3	VORAPAXAR		TdgClinicalTrial
TEK	TEK	CE-245677	inhibitor	ChemblInteractions
TEK	TEK	CETIRIZINE		DTC
TEK	TEK	CEP-11981	inhibitor	ChemblInteractions|GuideToPharmacology|TTD
TEK	TEK	REGORAFENIB	inhibitor	TALC|TdgClinicalTrial|ChemblInteractions|MyCancerGenomeClinicalTrial
TEK	TEK	GLESATINIB	inhibitor	TALC|TdgClinicalTrial
TEK	TEK	LOPERAMIDE		DTC
TEK	TEK	FORETINIB	inhibitor	ChemblInteractions
TEK	TEK	LINIFANIB	inhibitor	TdgClinicalTrial|GuideToPharmacology
TEK	TEK	ALTIRATINIB	inhibitor	ChemblInteractions|GuideToPharmacology|TTD
TEK	TEK	REBASTINIB		TTD
TEK	TEK	PEXMETINIB	inhibitor	TdgClinicalTrial|ChemblInteractions|GuideToPharmacology
TEK	TEK	MGCD-265	inhibitor	ChemblInteractions
TEK	TEK	RAZUPROTAFIB		TTD
TEK	TEK	CABOZANTINIB		ClearityFoundationClinicalTrial
TEK	TEK	GW559768X		DTC
TEK	TEK	AMPICILLIN		DTC
TEK	TEK	MERESTINIB	inhibitor	GuideToPharmacology
TEK	TEK	VANDETANIB	inhibitor	ChemblInteractions
SLPI	SLPI	HEPARIN		NCI
SLC2A5	SLC2A5	STREPTOZOCIN		NCI
SLC2A5	SLC2A5	GLUFOSFAMIDE		TdgClinicalTrial

F3	F3	SIMVASTATIN		PharmGKB
TUBA3D	TUBA3D	ZAMPANOLIDE		DTC
TUBA3D	TUBA3D	VINORELBINE TARTRATE	inhibitor	ChemblInteractions
TUBA3D	TUBA3D	VORINOSTAT		DTC
TUBA3D	TUBA3D	CABAZITAXEL	inhibitor	ChemblInteractions
TUBA3D	TUBA3D	DOCETAXEL	inhibitor	ChemblInteractions
TUBA3D	TUBA3D	CHEMBL1935538		DTC
TUBA3D	TUBA3D	ERIBULIN MESYLATE	inhibitor	ChemblInteractions
TUBA3D	TUBA3D	INDIBULIN	inhibitor	ChemblInteractions
TUBA3D	TUBA3D	FOSBRETABULIN TROMETHAMINE		ChemblInteractions
TUBA3D	TUBA3D	SAGOPILONE		ChemblInteractions
TUBA3D	TUBA3D	VINFLUNINE	inhibitor	ChemblInteractions
TUBA3D	TUBA3D	VINBLASTINE		DTC
TUBA3D	TUBA3D	PLINABULIN	inhibitor	ChemblInteractions
TUBA3D	TUBA3D	LEXIBULIN	inhibitor	ChemblInteractions
TUBA3D	TUBA3D	COLCHICINE	inhibitor	DTC|ChemblInteractions
TUBA3D	TUBA3D	PACLITAXEL	inhibitor	DTC|ChemblInteractions
TUBA3D	TUBA3D	VINORELBINE		DTC
TUBA3D	TUBA3D	CHEMBL1795737		DTC
TUBA3D	TUBA3D	TRASTUZUMAB EMTANSINE	inhibitor	ChemblInteractions
TUBA3D	TUBA3D	NOCODAZOLE		DTC
TUBA3D	TUBA3D	PACLITAXEL POLIGLUMEX		ChemblInteractions
TUBA3D	TUBA3D	PODOFILOX		DTC
TUBA3D	TUBA3D	CHEMBL2036119		DTC
TUBA3D	TUBA3D	CYCLOSTREPTIN		DTC
TUBA3D	TUBA3D	VERUBULIN		ChemblInteractions
TUBA3D	TUBA3D	MAYTANSINOL		DTC
TUBA3D	TUBA3D	VINBLASTINE SULFATE	inhibitor	ChemblInteractions
TUBA3D	TUBA3D	CHEMBL2036124		DTC
TUBA3D	TUBA3D	CHEMBL453818		DTC
TUBA3D	TUBA3D	LARGAZOLE		DTC
TUBA3D	TUBA3D	VINCRISTINE		DTC
TUBA3D	TUBA3D	IXABEPILONE	inhibitor	ChemblInteractions
TUBA3D	TUBA3D	VINCRISTINE SULFATE	inhibitor	ChemblInteractions
TUBA3D	TUBA3D	MAYTANSINE		DTC
TUBA3D	TUBA3D	CROLIBULIN	inhibitor	ChemblInteractions
TUBA3D	TUBA3D	FOSBRETABULIN DISODIUM	inhibitor	ChemblInteractions
TUBA3D	TUBA3D	CURCUMIN		DTC
TUBA3D	TUBA3D	BRENTUXIMAB VEDOTIN	inhibitor	ChemblInteractions
TUBA3D	TUBA3D	COMBRETASTATIN A4		DTC
TUBA3D	TUBA3D	DAVUNETIDE		ChemblInteractions
TUBA3D	TUBA3D	ABT-751		DTC
FN1	FN1	L19IL2		ChemblInteractions
FN1	FN1	OCRIPLASMIN	cleavage	ChemblInteractions|TTD
FN1	FN1	L19SIP 131I		ChemblInteractions
FN1	FN1	L19TNFA		ChemblInteractions
CYP1B1	CYP1B1	AMINOGLUTETHIMIDE		NCI
CYP1B1	CYP1B1	ANASTROZOLE		NCI
CYP1B1	CYP1B1	FORMESTANE		NCI
CYP1B1	CYP1B1	PACLITAXEL		PharmGKB
CYP1B1	CYP1B1	FLUOROURACIL		PharmGKB
CYP1B1	CYP1B1	FADROZOLE		NCI
CYP1B1	CYP1B1	DOCETAXEL		PharmGKB
CYP1B1	CYP1B1	ACACETIN		DTC
CYP1B1	CYP1B1	TOREMIFENE		NCI
CYP1B1	CYP1B1	EPIRUBICIN		PharmGKB
CYP1B1	CYP1B1	CYCLOPHOSPHAMIDE		PharmGKB
CYP1B1	CYP1B1	CHRYSIN	inhibitor	DTC|GuideToPharmacology
CYP1B1	CYP1B1	ISOPROPYL ALCOHOL		NCI
CYP1B1	CYP1B1	2'H,4H-[2,8'-BICHROMEN]-4-ONE		DTC
CYP1B1	CYP1B1	PINOCEMBRIN		TTD

CYP1B1	CYP1B1	SPIRONOLACTONE		NCI
CYP1B1	CYP1B1	RISPERIDONE		PharmGKB
CYP1B1	CYP1B1	AMODIAQUINE		PharmGKB
CYP1B1	CYP1B1	DOXORUBICIN		PharmGKB
CYP1B1	CYP1B1	ALPHA-NAPHTHOFLAVONE		DTC
CYP1B1	CYP1B1	RIFAMPIN		NCI
CYP1B1	CYP1B1	CHEMBL2347759		DTC
CYP1B1	CYP1B1	NARINGENIN		TTD
CYP1B1	CYP1B1	EXEMESTANE		NCI
CYP1B1	CYP1B1	MEDROXYPROGESTERONE ACETATE		NCI
CYP1B1	CYP1B1	2'H,4H-[2,5'-BICHROMEN]-4-ONE		DTC
CYP1B1	CYP1B1	LETROZOLE		NCI
CYP1B1	CYP1B1	BETA-NAPHTHOFLAVONE		DTC
PDGFD	PDGFD	SUNITINIB	inhibitor	MyCancerGenomeClinicalTrial
CA4	CA4	SULFAMYLON		TTD
CA4	CA4	METHAZOLAMIDE	inhibitor	ChemblInteractions|GuideToPharmacology
CA4	CA4	ETHOXZOLAMIDE	inhibitor	ChemblInteractions
CA4	CA4	PARABEN		TTD
CA4	CA4	PHENOL		TTD
CA4	CA4	TOPIRAMATE	inhibitor	TdgClinicalTrial|ChemblInteractions|TEND|GuideToPharmacology
CA4	CA4	ACETAZOLAMIDE	inhibitor	ChemblInteractions|GuideToPharmacology
CA4	CA4	CHLOROTHIAZIDE	inhibitor	TdgClinicalTrial|TEND
CA4	CA4	TRICHLORMETHIAZIDE	inhibitor	TdgClinicalTrial|TEND
CA4	CA4	DICHLORPHENAMIDE	inhibitor	ChemblInteractions|GuideToPharmacology
CA4	CA4	ACETAZOLAMIDE SODIUM	inhibitor	ChemblInteractions
CA4	CA4	CHLORTHALIDONE	inhibitor	GuideToPharmacology
CA4	CA4	BRINZOLAMIDE	inhibitor	TTD
CA4	CA4	SULFAMIDE		TTD
CA4	CA4	CURCUMIN		TTD
GJB1	GJB1	CARBENOXOLONE	inhibitor	GuideToPharmacology
GJB1	GJB1	OCTANOL	inhibitor	GuideToPharmacology
VCAN	VCAN	CYCLOSPORINE		NCI
KCNK3	KCNK3	HALOTHANE	binder|activator	ChemblInteractions|GuideToPharmacology
KCNK3	KCNK3	DOXAPRAM HYDROCHLORIDE	blocker	ChemblInteractions
KCNK3	KCNK3	ENFLURANE		ChemblInteractions
KCNK3	KCNK3	SEVOFLURANE		ChemblInteractions
KCNK3	KCNK3	ISOFLURANE		ChemblInteractions
KCNK3	KCNK3	ANANDAMIDE	blocker	GuideToPharmacology
KCNK3	KCNK3	DESFLURANE		ChemblInteractions
KCNK3	KCNK3	CANDESARTAN		PharmGKB
SORCS2	SORCS2	ATAZANAVIR		PharmGKB
NGFR	NGFR	VOCLOSPORIN		TTD
NGFR	NGFR	FULRANUMAB		TTD
NGFR	NGFR	CENEGERMIN		TTD
CCL20	CCL20	GSK-3050002		TTD
SERPINE1	SERPINE1	DEFIBROTIDE		NCI
SERPINE1	SERPINE1	LEVOTHYROXINE		NCI
SERPINE1	SERPINE1	CITALOPRAM		PharmGKB
SERPINE1	SERPINE1	CETRORELIX		NCI
SERPINE1	SERPINE1	UROKINASE	inducer|substrate	TdgClinicalTrial
SERPINE1	SERPINE1	ALEPLASININ	inhibitor	ChemblInteractions
SERPINE1	SERPINE1	REGRAMOSTIM		NCI
SERPINE1	SERPINE1	FLUOXETINE		PharmGKB
SERPINE1	SERPINE1	DEXAMETHASONE		PharmGKB
SERPINE1	SERPINE1	VASOPRESSIN		NCI
SERPINE1	SERPINE1	TIPLASININ		DTC
SERPINE1	SERPINE1	NIMODIPINE		NCI
SERPINE1	SERPINE1	DALTEPARIN		NCI
SERPINE1	SERPINE1	PREMARIN		NCI
SERPINE1	SERPINE1	ORLISTAT		NCI
SERPINE1	SERPINE1	CAPTOPRIL		NCI

SERPINE1	SERPINE1	URIDINE		NCI
SERPINE1	SERPINE1	DIAPLASININ		TTD
SERPINE1	SERPINE1	HYDROCHLOROTHIAZIDE		NCI
SERPINE1	SERPINE1	EPIRUBICIN		NCI
COL5A1	COL5A1	OCRIPLASMIN		ChemblInteractions
COL5A1	COL5A1	COLLAGENASE CLOSTRIDIUM HISTOLYTICUM		ChemblInteractions
VCAM1	VCAM1	TROGLITAZONE		NCI
VCAM1	VCAM1	MERCAPTOPURINE		NCI
VCAM1	VCAM1	DEXAMETHASONE		NCI
VCAM1	VCAM1	CYCLOSPORINE		NCI
CYP24A1	CYP24A1	DEFERASIROX		PharmGKB
CYP24A1	CYP24A1	CHEMBL255088	inhibitor	GuideToPharmacology
CYP24A1	CYP24A1	LUNACALCIPOL		TdgClinicalTrial|TTD
CYP24A1	CYP24A1	CALCITRIOL		DTC
CYP24A1	CYP24A1	TELAPREVIR		PharmGKB
COL1A2	COL1A2	OCRIPLASMIN		ChemblInteractions
COL1A2	COL1A2	COLLAGENASE CLOSTRIDIUM HISTOLYTICUM		ChemblInteractions|TEND
SLC22A6	SLC22A6	TENOFOVIR		PharmGKB
SLC22A6	SLC22A6	PRATOSARTAN	inhibitor	ChemblInteractions
SLC22A6	SLC22A6	RUCAPARIB		PharmGKB
SLC22A6	SLC22A6	CIDOFOVIR		PharmGKB
SLC22A6	SLC22A6	GS-9256		DTC
SLC22A6	SLC22A6	PROBENECID	inhibitor	ChemblInteractions|GuideToPharmacology
SLC22A6	SLC22A6	PRAVASTATIN		PharmGKB
SLC22A6	SLC22A6	ZIDOVUDINE		PharmGKB
IGF2	IGF2	XENTUZUMAB		TTD
IGF2	IGF2	CABAZITAXEL		CIViC
IGF2	IGF2	ANASTROZOLE		NCI
IGF2	IGF2	GEFITINIB		NCI
IGF2	IGF2	ETOPOSIDE		NCI
IGF2	IGF2	DOCETAXEL		CIViC
IGF2	IGF2	HUMAN GROWTH HORMONE		NCI
IGF2	IGF2	LINSITINIB		CIViC
IGF2	IGF2	GLUTAMINE		NCI
IGF2	IGF2	BETAMETHASONE		NCI
IGF2	IGF2	DUSIGITUMAB	inhibitor	ChemblInteractions|TTD
IGF2	IGF2	FLUOXETINE		NCI
IGF2	IGF2	GANITUMAB		CIViC
IGF2	IGF2	GEMCITABINE		CIViC
SLC6A19	SLC6A19	CINROMIDE	inhibitor	GuideToPharmacology
SLC6A19	SLC6A19	BENZATROPINE	inhibitor	GuideToPharmacology
LTF	LTF	BACITRACIN		NCI
LTF	LTF	PARECOXIB		TTD
LTF	LTF	RESERPINE		NCI
LTF	LTF	TALACTOFERRIN ALFA		TTD
SLC22A12	SLC22A12	LESINURAD	inhibitor	ChemblInteractions|GuideToPharmacology|TTD
SLC22A12	SLC22A12	SULFINPYRAZONE	inhibitor	ChemblInteractions
SLC22A12	SLC22A12	PRATOSARTAN	inhibitor	ChemblInteractions
SLC22A12	SLC22A12	VERINURAD	inhibitor	GuideToPharmacology
SLC22A12	SLC22A12	ARHALOFENATE	inhibitor	ChemblInteractions
SLC22A12	SLC22A12	FLUDARABINE		PharmGKB
SLC22A12	SLC22A12	IDARUBICIN		PharmGKB
SLC22A12	SLC22A12	CYTARABINE		PharmGKB
REG1A	REG1A	ROQUINIMEX		NCI
REG1A	REG1A	INSULIN		NCI
MMP7	MMP7	PRINOMASTAT		TTD
MMP7	MMP7	MARIMASTAT	inhibitor|antagonist	GuideToPharmacology|TTD
MMP7	MMP7	DOXYCYCLINE HYCLATE	inhibitor	ChemblInteractions
MMP7	MMP7	DOXYCYCLINE	inhibitor	ChemblInteractions
MMP7	MMP7	DOXYCYCLINE	inhibitor	GuideToPharmacology
MMP7	MMP7	DOXYCYCLINE CALCIUM	inhibitor	ChemblInteractions

DES	DES	ALTEPLASE		NCI
DES	DES	THROMBIN		NCI
FGG	FGG	ALFIMEPRASE		TdgClinicalTrial
FGG	FGG	EPTIFIBATIDE		PharmGKB
FGG	FGG	FIBRINOLYSIN, HUMAN	inhibitor	ChemblInteractions
FGG	FGG	TIROFIBAN		PharmGKB
FGG	FGG	ABCIXIMAB		PharmGKB
RBP4	RBP4	FENRETINIDE		DTC|NCI
RBP4	RBP4	ROSIGLITAZONE		NCI
CYP4A11	CYP4A11	AMILORIDE		PharmGKB
CYP4A11	CYP4A11	SPIRONOLACTONE		PharmGKB

CMAP

drug	cellline	pval	FDR
TRICHOSTATIN A	HL60	1.30E-12	4.65E-09
TANESPIMYCIN	HL60	3.03E-08	5.44E-05
VALPROIC ACID	HL60	4.88E-08	5.84E-05
LY-294002	HL60	1.18E-06	0.00106222
LY-294002	PC3	1.66E-05	0.009953761
TRICHOSTATIN A	PC3	1.66E-05	0.009953761
TRETINOIN	MCF7	0.00170032	0.087129237
VORINOSTAT	HL60	0.002878702	1
15-DELTA PROSTAGLANDIN J2	HL60	0.003788203	1
ALPHA-ESTRADIOL	HL60	0.006282167	1
TANESPIMYCIN	PC3	0.013305691	1
ALVESPIMYCIN	HL60	0.018594206	1
SIROLIMUS	HL60	0.020864777	1
CHLORPROMAZINE	HL60	0.029648688	1
TERGURIDE	HL60	0.031330123	1
LYCORINE	MCF7	0.032774027	1
SIROLIMUS	MCF7	0.034302261	1
DIPHEMANIL METILSULFATE	MCF7	0.0421254	1
LITHOCHOLIC ACID	PC3	0.047452449	1
RALOXIFENE	HL60	0.048218234	1
AG-028671	PC3	0.05309223	1
TINIDAZOLE	MCF7	0.054409598	1
SIROLIMUS	PC3	0.054628188	1
OFLOXACIN	PC3	0.055833851	1
CEFADROXIL	MCF7	0.056899583	1
C-75	PC3	0.056962793	1
MEFLOQUINE	PC3	0.057725839	1
IOPANOIC ACID	MCF7	0.060330425	1
0297417-0002B	MCF7	0.061466252	1
SCOPOLAMINE N-OXIDE	MCF7	0.061736041	1
SULFADIMIDINE	MCF7	0.064758632	1
PF-00562151-00	PC3	0.065243736	1
DROPROPIZINE	MCF7	0.068876342	1
WORTMANNIN	PC3	0.069543942	1
SITOSTEROL	MCF7	0.070517524	1
DILAZEP	PC3	0.070668283	1
NEOMYCIN	MCF7	0.072961199	1
DEMECLOCYCLINE	PC3	0.073426928	1
FLUFENAMIC ACID	MCF7	0.0742278	1
CLOPERASTINE	PC3	0.074996742	1
CETIRIZINE	MCF7	0.076513043	1
CEPHAELINE	MCF7	0.076959928	1
ETOPOSIDE	MCF7	0.078710161	1
SCRIPTAID	PC3	0.079453697	1
METARAMINOL	PC3	0.080537634	1
DILTIAZEM	PC3	0.081041895	1
PROSCILLARIDIN	MCF7	0.08334244	1
DICLOFENAC	PC3	0.083774137	1
SULFADIAZINE	PC3	0.084468564	1
TORASEMIDE	MCF7	0.085518806	1
FUROSEMIDE	MCF7	0.086224728	1
NIZATIDINE	MCF7	0.087113645	1
URAPIDIL	MCF7	0.088822648	1
ETANIDAZOLE	MCF7	0.090742384	1
GELDANAMYCIN	HL60	0.091666037	1
NORDIHYDROGUAIARETIC ACID	HL60	0.091741948	1
ERYTHROMYCIN	PC3	0.09525367	1
ROSIGLITAZONE	HL60	0.095645748	1
URSOLIC ACID	PC3	0.096602206	1

METICRANE	PC3	0.096990246	1
TREMORINE	MCF7	0.097477017	1
PRESTWICK-1100	MCF7	0.09934426	1
OZAGREL	MCF7	0.099542431	1
ACENOCOUMAROL	PC3	0.104083629	1
COLFORSIN	MCF7	0.104392022	1
FLUDROXYCORTIDE	PC3	0.111791651	1
TROLOX C	MCF7	0.112008871	1
FURSULTIAMINE	MCF7	0.112117602	1
APIGENIN	MCF7	0.113099857	1
ORLISTAT	PC3	0.113209404	1
IMIDUREA	MCF7	0.114973301	1
TIRATRICOL	MCF7	0.116533978	1
MONASTROL	MCF7	0.116596619	1
FULVESTRANT	MCF7	0.117127668	1
PYRIDOXINE	MCF7	0.121082644	1
ACACETIN	PC3	0.121198073	1
DILAZEP	MCF7	0.123641517	1
PRESTWICK-920	MCF7	0.124228777	1
STREPTOMYCIN	MCF7	0.125765574	1
PHA-00745360	MCF7	0.125832224	1
PNU-0293363	PC3	0.129003418	1
CICLACILLIN	MCF7	0.129124537	1
CITALOPRAM	MCF7	0.13095165	1
HELVETICOSIDE	PC3	0.13168793	1
HALCINONIDE	MCF7	0.131810945	1
PHA-00851261E	PC3	0.131819148	1
AMANTADINE	MCF7	0.131934048	1
DOXYLAMINE	MCF7	0.132303875	1
PROCAINE	PC3	0.133915479	1
DORZOLAMIDE	MCF7	0.135039816	1
SOLANINE	HL60	0.135379663	1
SECURININE	HL60	0.135468458	1
FURSULTIAMINE	HL60	0.135557297	1
MEVALOLACTONE	HL60	0.13564618	1
APIGENIN	HL60	0.135735106	1
LEVCYCLOSERINE	MCF7	0.135793299	1
PIPERLONGUMINE	HL60	0.135824076	1
TORASEMIDE	HL60	0.13591309	1
SANGUINARINE	HL60	0.136002148	1
THIOSTREPTON	HL60	0.136091249	1
ETAMSYLATE	HL60	0.136180394	1
PRESTWICK-967	HL60	0.136269583	1
TODRALAZINE	HL60	0.136448091	1
FLUVOXAMINE	HL60	0.136626775	1
PRESTWICK-1080	MCF7	0.13667634	1
CINOXACIN	HL60	0.136716183	1
NARINGIN	HL60	0.136805634	1
SULFAPHENAZOLE	HL60	0.136895129	1
THAPSIGARGIN	MCF7	0.136929425	1
APRAMYCIN	HL60	0.136984668	1
MECLOFENOXATE	HL60	0.137074251	1
OXAMNIQUINE	HL60	0.137343263	1
METICRANE	HL60	0.137433021	1
PROSCILLARIDIN	HL60	0.137522823	1
DEQUALINIUM CHLORIDE	HL60	0.137702559	1
LYMECYCLINE	HL60	0.137792493	1
NAFTIFINE	HL60	0.13788247	1
SOTALOL	HL60	0.137972492	1
TRACAZOLATE	HL60	0.138062557	1
FELODIPINE	HL60	0.138152667	1

CHLORAMPHENICOL	HL60	0.13824282	1
8-AZAGUANINE	HL60	0.138423259	1
PENTOLONIUM	MCF7	0.138455309	1
SULFACHLORPYRIDAZINE	HL60	0.138513544	1
ANDROSTERONE	HL60	0.138694247	1
ROXARSONE	HL60	0.138875126	1
TIABENDAZOLE	HL60	0.138965631	1
BICUCULLINE	MCF7	0.13909483	1
PINACIDIL	HL60	0.139146774	1
PIPEMIDIC ACID	HL60	0.139237411	1
ESTROPIPATE	HL60	0.139328093	1
F0447-0125	MCF7	0.139351255	1
LEVONORGESTREL	HL60	0.139418818	1
NATAMYCIN	HL60	0.139509588	1
DICLOXACILLIN	HL60	0.139600402	1
CEFALOTIN	HL60	0.13969126	1
PIMETHIXENE	HL60	0.139782162	1
NALBUPHINE	HL60	0.139873108	1
HYDROFLUMETHIAZIDE	HL60	0.139964098	1
DECAMETHONIUM BROMIDE	HL60	0.140055132	1
BENZONATATE	HL60	0.140146211	1
MORANTEL	HL60	0.1403285	1
IPRATROPIUM BROMIDE	HL60	0.140419711	1
ACENOCOUMAROL	HL60	0.140510966	1
NAFTOPIDIL	HL60	0.140602266	1
ZUCLOPENTHIXOL	HL60	0.140693609	1
IOXAGLIC ACID	HL60	0.140784997	1
FLUPENTIXOL	HL60	0.140876429	1
PRESTWICK-1103	MCF7	0.140897218	1
ALLANTOIN	HL60	0.140967905	1
GIBBERELLIC ACID	HL60	0.141059425	1
ZOMEPIRAC	HL60	0.14115099	1
BRINZOLAMIDE	MCF7	0.141156115	1
GINKGOLIDE A	HL60	0.141242599	1
RISPERIDONE	HL60	0.141334252	1
SKIMMIANINE	HL60	0.14142595	1
CANTHARIDIN	HL60	0.141517691	1
TACRINE	HL60	0.141609477	1
ETOMIDATE	HL60	0.141701308	1
NAPELLINE	HL60	0.141793182	1
LAUDANOSINE	HL60	0.141885101	1
N-ACETYL-L-LEUCINE	HL60	0.141977064	1
TALAMPICILLIN	HL60	0.142069072	1
ETOFENAMATE	HL60	0.142161124	1
PRESTWICK-1083	HL60	0.14225322	1
LUTEOLIN	HL60	0.142529775	1
IMIPRAMINE	HL60	0.142622049	1
TUBOCURARINE CHLORIDE	HL60	0.142806729	1
ZARDAVERINE	HL60	0.142899136	1
AMINOHIPPURIC ACID	HL60	0.143084083	1
ISOXSUPRINE	PC3	0.143109148	1
CYANOCOBALAMIN	HL60	0.143176624	1
MOLINDONE	HL60	0.143269208	1
KINETIN	HL60	0.143454511	1
HYDROCOTARNINE	HL60	0.143547229	1
TROPICAMIDE	HL60	0.143639992	1
EPIRIZOLE	HL60	0.143732799	1
HESPERETIN	PC3	0.1437646	1
LETROZOLE	HL60	0.14382565	1
URAPIDIL	HL60	0.144011487	1
PF-01378883-00	PC3	0.144158939	1

PRONETALOL	HL60	0.144290576	1
CHRYSIN	HL60	0.144476858	1
BEPRIDIL	HL60	0.144570066	1
REPAGLINIDE	HL60	0.144663318	1
BECLOMETASONE	HL60	0.144756615	1
NIFLUMIC ACID	HL60	0.144849957	1
METHAZOLAMIDE	HL60	0.144943343	1
PRESTWICK-664	HL60	0.145223769	1
METITEPINE	HL60	0.145317334	1
PYRVINIUM	HL60	0.145410943	1
CIPROFLOXACIN	PC3	0.145479197	1
PRESTWICK-665	HL60	0.145504597	1
BISOPROLOL	HL60	0.145598296	1
OUABAIN	HL60	0.145692039	1
METHYLDOPATE	HL60	0.14587966	1
HYMECROMONE	MCF7	0.146009801	1
VERTEPORFIN	HL60	0.14606746	1
MOXONIDINE	HL60	0.146161427	1
FLUNIXIN	HL60	0.146255438	1
HOMATROPINE	PC3	0.146275639	1
TRIAMTERENE	HL60	0.146443596	1
EPIVINCAMINE	HL60	0.146537742	1
PROMETHAZINE	HL60	0.146726167	1
PHTHALYLSULFATHIAZOLE	HL60	0.146820448	1
METHYLDOPA	HL60	0.146914773	1
SCOPOLETIN	HL60	0.147009142	1
OXOLINIC ACID	HL60	0.147103557	1
SULCONAZOLE	HL60	0.147198016	1
CHLORPROTHIXENE	HL60	0.14729252	1
SULFADIMIDINE	HL60	0.147387069	1
CEFALONIUM	HL60	0.147481663	1
SPAGLUMIC ACID	HL60	0.147576302	1
6-BENZYLAMINOPURINE	HL60	0.147670985	1
NITRENDIPINE	HL60	0.147765713	1
PROMAZINE	HL60	0.147860487	1
DOXYCYCLINE	HL60	0.147955305	1
PROTRIPTYLINE	HL60	0.148145076	1
CEFUROXIME	HL60	0.148240028	1
VANOXERINE	HL60	0.148430069	1
AMINOGLUTETHIMIDE	HL60	0.148525156	1
MESALAZINE	HL60	0.148620289	1
PRESTWICK-1084	MCF7	0.148684314	1
BETHANECHOL	HL60	0.148715466	1
FAMPROFAZONE	HL60	0.148810689	1
THIOGUANOSINE	HL60	0.148905956	1
DIPHENYLPYRALINE	HL60	0.149096625	1
NETILMICIN	HL60	0.149192028	1
CICLOSPORIN	HL60	0.149287475	1
FLAVOXATE	HL60	0.149382967	1
PHENTOLAMINE	HL60	0.149574087	1
ISRADIPINE	HL60	0.149765387	1
ETYNODIOL	HL60	0.149861104	1
DIOXYBENZONE	HL60	0.149956866	1
CICLOPIROX	HL60	0.150148526	1
CONESSINE	HL60	0.150244424	1
CEFUROXIME	MCF7	0.150306273	1
TRIOXYSALEN	HL60	0.150340367	1
BUFEXAMAC	HL60	0.150436355	1
SITOSTEROL	HL60	0.150532388	1
LIOTHYRONINE	HL60	0.150628466	1
DIGOXIGENIN	HL60	0.150724589	1

BUSPIRONE	HL60	0.150820757	1
TRIPROLIDINE	HL60	0.151013229	1
LATAMOXEF	HL60	0.151109533	1
YOHIMBIC ACID	HL60	0.151205882	1
0175029-0000	MCF7	0.151258411	1
CINCHONIDINE	HL60	0.151302276	1
BROMOPRIDE	HL60	0.151398715	1
HYOSCYAMINE	HL60	0.151495199	1
16,16-DIMETHYLPROSTAGLANDIN E2	PC3	0.151531264	1
DIGITOXIGENIN	HL60	0.151591729	1
REMOXIPRIDE	HL60	0.151688304	1
RIMEXOLONE	HL60	0.151784924	1
CEFOTAXIME	MCF7	0.151804478	1
LEVOCABASTINE	HL60	0.151978299	1
ANTIMYCIN A	HL60	0.152075055	1
ESTRIOL	HL60	0.152171856	1
CP-690334-01	PC3	0.15222458	1
CISAPRIDE	HL60	0.152268702	1
DIFLORASONE	HL60	0.152365593	1
PICROTOXININ	HL60	0.152559512	1
BUTIROSIN	HL60	0.152656539	1
MEROPENEM	HL60	0.152753612	1
HELIOTRINE	HL60	0.152850729	1
TREMORINE	HL60	0.152947893	1
ACETAZOLAMIDE	HL60	0.153045101	1
PENTETRAZOL	HL60	0.153142355	1
CYCLOPENTOLATE	HL60	0.153239654	1
RONIDAZOLE	HL60	0.153336998	1
PHENAZOPYRIDINE	HL60	0.153434388	1
METAMIZOLE SODIUM	HL60	0.153531823	1
IDAZOXAN	HL60	0.153629303	1
DICOUMAROL	HL60	0.153726829	1
PANCURONIUM BROMIDE	HL60	0.1538244	1
FLUSPIRILENE	HL60	0.153922017	1
NIRIDAZOLE	HL60	0.154117386	1
METRIZAMIDE	MCF7	0.154141421	1
PARACETAMOL	HL60	0.154215138	1
TINIDAZOLE	HL60	0.15441078	1
DEXPROPRANOLOL	HL60	0.154508669	1
NAFTIDROFURYL	HL60	0.154606604	1
OXAPROZIN	PC3	0.154695101	1
PRESTWICK-674	HL60	0.154704583	1
RETRORSINE	HL60	0.154900679	1
DROPROPIZINE	HL60	0.154998796	1
TOBRAMYCIN	HL60	0.155195164	1
PIRIBEDIL	HL60	0.155293417	1
CETIRIZINE	HL60	0.155391715	1
RIBOSTAMYCIN	HL60	0.155490059	1
LORGLUMIDE	HL60	0.155588448	1
OXPRENOLOL	HL60	0.155785363	1
DELSOLINE	HL60	0.155883889	1
ETHOTOIN	HL60	0.156179739	1
KETOTIFEN	HL60	0.156377201	1
PYRANTEL	HL60	0.156476	1
(-)-ATENOLOL	HL60	0.156673735	1
CYCLOSERINE	HL60	0.156772671	1
DESOXYCORTONE	HL60	0.156871652	1
SULFANILAMIDE	HL60	0.156970679	1
MEDRYSONE	HL60	0.15716887	1
IOPANOIC ACID	HL60	0.157268034	1
PARTHENOLIDE	HL60	0.157367244	1

BEZAFIBRATE	HL60	0.157466499	1
PENTAMIDINE	MCF7	0.157643169	1
CANAVANINE	HL60	0.157764539	1
PRESTWICK-860	HL60	0.157863977	1
TRIFLUSAL	HL60	0.157963461	1
STAUROSPORINE	MCF7	0.158045164	1
FLUDROXYCORTIDE	HL60	0.158062991	1
MECLOZINE	HL60	0.158162566	1
AZLOCILLIN	HL60	0.158262186	1
RIBOFLAVIN	HL60	0.158361853	1
AMIODARONE	HL60	0.158461565	1
CARBAMAZEPINE	HL60	0.158661127	1
SELEGILINE	HL60	0.158760976	1
PAPAVERINE	HL60	0.158860872	1
EQUILIN	HL60	0.158960813	1
MEPHENESIN	HL60	0.1590608	1
DACARBAZINE	HL60	0.159160832	1
ALTIZIDE	HL60	0.15926091	1
LISINOPRIL	HL60	0.159361035	1
GUAIFENESIN	HL60	0.159461205	1
ETOFYLLINE	HL60	0.15956142	1
PIMOZIDE	HL60	0.159661682	1
HALCINONIDE	HL60	0.159761989	1
DIMENHYDRINATE	HL60	0.159962742	1
PIRENZEPINE	HL60	0.160063187	1
METACYCLINE	HL60	0.160264214	1
MYOSMINE	HL60	0.160364796	1
ESTRONE	HL60	0.160465425	1
TERAZOSIN	MCF7	0.160739307	1
BERBERINE	HL60	0.160767585	1
ELLIPTICINE	HL60	0.160969255	1
MINOXIDIL	MCF7	0.161024552	1
FLUTICASONE	HL60	0.161070159	1
BRINZOLAMIDE	HL60	0.161171108	1
FLUVASTATIN	HL60	0.161272104	1
ATOVAQUONE	HL60	0.161373146	1
CITIOLONE	HL60	0.161474233	1
TELENZEPINE	HL60	0.161575367	1
ACECLOFENAC	PC3	0.161596144	1
SUPROFEN	HL60	0.161676546	1
BENZYLPENICILLIN	HL60	0.161777771	1
METACYCLINE	MCF7	0.161882491	1
BENZAMIL	HL60	0.16198036	1
NIPECOTIC ACID	HL60	0.162081723	1
ORPHENADRINE	HL60	0.162183133	1
ACACETIN	HL60	0.162284588	1
ARTICAINE	HL60	0.162386089	1
MEMANTINE	HL60	0.162487637	1
TERCONAZOLE	HL60	0.16258923	1
TRICHLORMETHIAZIDE	MCF7	0.16259997	1
CEFACLOR	HL60	0.162690869	1
ALFAXALONE	HL60	0.162792555	1
ETHIONAMIDE	HL60	0.162894286	1
TESTOSTERONE	HL60	0.162996064	1
MAFENIDE	MCF7	0.163175611	1
PYRITHYLDIONE	HL60	0.163199757	1
NORETYNODREL	HL60	0.163301672	1
DIGOXIN	HL60	0.163403634	1
PHENYLPROPANOLAMINE	HL60	0.163505642	1
OXANTEL	HL60	0.163607696	1
CHLORMEZANONE	HL60	0.163709796	1

CINCHONINE	HL60	0.163914134	1
DANTROLENE	HL60	0.164118657	1
METOPROLOL	HL60	0.164425788	1
SULFACETAMIDE	HL60	0.164528257	1
PRAZOSIN	HL60	0.164630772	1
TRAZODONE	HL60	0.164733333	1
TIAPROFENIC ACID	HL60	0.164835941	1
CEFOTAXIME	HL60	0.164938594	1
TICARCILLIN	HL60	0.165041294	1
DYCLONINE	HL60	0.16514404	1
THIOPROPERAZINE	HL60	0.165246833	1
TRIDIHEXETHYL	HL60	0.165349671	1
COLECALCIFEROL	HL60	0.165452556	1
CLOMIPRAMINE	MCF7	0.165930058	1
DIFENIDOL	HL60	0.165967673	1
MINOCYCLINE	HL60	0.166070835	1
BUMETANIDE	HL60	0.166174043	1
METYRAPONE	HL60	0.166380599	1
BICUCULLINE	HL60	0.166483946	1
XYLAZINE	HL60	0.16658734	1
15(S)-15-METHYLPROSTAGLANDIN E2	PC3	0.166660458	1
LY-294002	MCF7	0.166813463	1
CORALYNE	HL60	0.166897798	1
METHANTHELINIUM BROMIDE	MCF7	0.166953267	1
CARBIMAZOLE	HL60	0.167001376	1
TICLOPIDINE	HL60	0.167105001	1
CLEBOPRIDE	HL60	0.167208673	1
TETRAMISOLE	HL60	0.16731239	1
PHENACETIN	MCF7	0.167393174	1
VELNACRINE	HL60	0.167416154	1
MEPHENTERMINE	PC3	0.167686908	1
DOXAZOSIN	HL60	0.167727724	1
OXYTETRACYCLINE	MCF7	0.167831674	1
SULFAMETHOXAZOLE	MCF7	0.167833915	1
ETAMIVAN	HL60	0.167935669	1
FOLIOSIDINE	HL60	0.168039711	1
BERGENIN	PC3	0.1681438	1
CLOFAZIMINE	HL60	0.168247935	1
DENATONIUM BENZOATE	MCF7	0.168275491	1
ACEPROMAZINE	HL60	0.168352116	1
OLIGOMYCIN	MCF7	0.168560618	1
SULFAMETOXYDIAZINE	HL60	0.168664938	1
3-ACETYLCOUMARIN	HL60	0.168769305	1
TERGURIDE	MCF7	0.168934854	1
SULFAMETHOXYPYRIDAZINE	HL60	0.168978178	1
(+)-ISOPRENALINE	MCF7	0.169161149	1
PYRAZINAMIDE	HL60	0.169187237	1
TRACAZOLATE	PC3	0.169291836	1
PRESTWICK-675	HL60	0.169396482	1
FENOPROFEN	HL60	0.169501174	1
GALLAMINE TRIETHIODIDE	HL60	0.169605912	1
SULINDAC	HL60	0.169710697	1
NEOSTIGMINE BROMIDE	HL60	0.169815529	1
CEFTAZIDIME	HL60	0.170130302	1
IMIPENEM	HL60	0.170655854	1
AMRINONE	HL60	0.170761104	1
APOMORPHINE	HL60	0.1708664	1
THIAMPHENICOL	MCF7	0.171091555	1
PRESTWICK-1103	HL60	0.171182569	1
CORTISONE	HL60	0.171288052	1
BETAXOLOL	PC3	0.171710449	1

RAMIPRIL	HL60	0.172133591	1
LIDOCAINE	HL60	0.172451438	1
PROTOVERATRINE A	HL60	0.17255748	1
MEROPENEM	MCF7	0.172587191	1
FLURBIPROFEN	HL60	0.172769704	1
PHENSUXIMIDE	HL60	0.172875886	1
TYLOXAPOL	HL60	0.17308839	1
SULMAZOLE	PC3	0.173194712	1
KETOCONAZOLE	HL60	0.173407496	1
MECLOFENAMIC ACID	HL60	0.173513958	1
6-AZATHYMINE	HL60	0.173620466	1
ADRENOSTERONE	HL60	0.173727022	1
BISACODYL	HL60	0.173940273	1
DROFENINE	HL60	0.174046968	1
PYRAZINAMIDE	MCF7	0.174092157	1
IFENPRODIL	HL60	0.17415371	1
ARCAINE	HL60	0.174260499	1
RESVERATROL	HL60	0.174367335	1
ETANIDAZOLE	HL60	0.174688123	1
ZALCITABINE	HL60	0.174795146	1
SUXIBUZONE	HL60	0.174902215	1
ENALAPRIL	HL60	0.175009331	1
ESCULIN	MCF7	0.175151193	1
BUTOCONAZOLE	HL60	0.175223704	1
PYRIMETHAMINE	HL60	0.175330961	1
ESCULETIN	HL60	0.175438264	1
MOXISYLYTE	HL60	0.175545614	1
PIVAMPICILLIN	HL60	0.175760455	1
SERTACONAZOLE	HL60	0.175867946	1
CLOPERASTINE	HL60	0.175975483	1
PEMPIDINE	HL60	0.176190698	1
HEXESTROL	HL60	0.176298376	1
R-ATENOLOL	HL60	0.176406101	1
CLOMIFENE	HL60	0.176513873	1
NIMESULIDE	MCF7	0.17651955	1
AMPROLIUM	HL60	0.176621691	1
TRICHLORMETHIAZIDE	HL60	0.176729556	1
IOVERSOL	MCF7	0.17682466	1
CEFOTETAN	HL60	0.176837469	1
GOSSYPOL	HL60	0.176945428	1
PRESTWICK-664	PC3	0.177130145	1
PF-00562151-00	MCF7	0.177226827	1
WORTMANNIN	SKMEL5	0.177269586	1
ANABASINE	HL60	0.177377733	1
MECLOFENOXATE	PC3	0.177589075	1
DEXPANTHENOL	HL60	0.177594166	1
PIPENZOLATE BROMIDE	HL60	0.177702454	1
LABETALOL	HL60	0.177919169	1
DOXEPIN	HL60	0.178136072	1
HELVETICOSIDE	HL60	0.178244594	1
NIFURTIMOX	PC3	0.178353162	1
COLFORSIN	HL60	0.178679151	1
CHLORPHENESIN	HL60	0.178787907	1
GRAMINE	HL60	0.178896711	1
NIFUROXAZIDE	HL60	0.179005561	1
LANATOSIDE C	HL60	0.179114459	1
INDOMETACIN	HL60	0.179332395	1
GLICLAZIDE	HL60	0.179441433	1
FLUVASTATIN	PC3	0.179550519	1
TOLBUTAMIDE	HL60	0.17976883	1
FLUNISOLIDE	HL60	0.179878057	1

FLUOROCURARINE	HL60	0.17998733	1
OXYBUPROCAINE	HL60	0.180315433	1
DILTIAZEM	HL60	0.180424894	1
ENOXACIN	MCF7	0.180515262	1
NICOTINIC ACID	PC3	0.180534403	1
ECONAZOLE	HL60	0.180643959	1
DICLOFENAMIDE	HL60	0.180753561	1
NIFEDIPINE	HL60	0.180863211	1
PRESTWICK-689	HL60	0.180972908	1
DL-ALPHA TOCOPHEROL	PC3	0.181082652	1
CHLORTALIDONE	HL60	0.181302281	1
PIPERACETAZINE	HL60	0.181412166	1
OMEPRAZOLE	HL60	0.181742104	1
EPITIOSTANOL	HL60	0.181852177	1
DEMECLOCYCLINE	HL60	0.181962298	1
CARBACHOL	HL60	0.182072466	1
DEQUALINIUM CHLORIDE	PC3	0.18218268	1
ROLIPRAM	HL60	0.182292942	1
OXYPHENBUTAZONE	HL60	0.182513607	1
EBSELEN	HL60	0.182734461	1
HARPAGOSIDE	HL60	0.182844959	1
METRIFONATE	HL60	0.182955503	1
1,4-CHRYSENEQUINONE	HL60	0.183066095	1
SOTALOL	MCF7	0.18316222	1
PHENACETIN	HL60	0.18328742	1
ZIMELDINE	HL60	0.183619762	1
VITEXIN	MCF7	0.183632158	1
SC-560	PC3	0.183730637	1
ETHOXYQUIN	MCF7	0.183788993	1
BENSERAZIDE	PC3	0.183841559	1
FLUVASTATIN	MCF7	0.183945923	1
RIFABUTIN	PC3	0.184285719	1
(-)-ISOPRENALINE	MCF7	0.184417277	1
CEFAPIRIN	HL60	0.184508082	1
SULFAFURAZOLE	MCF7	0.184637626	1
ISOETARINE	PC3	0.184953376	1
TERGURIDE	PC3	0.185047071	1
AH-23848	PC3	0.185176306	1
MIDECAMYCIN	HL60	0.185287841	1
ANISOMYCIN	HL60	0.185622732	1
PEPSTATIN	HL60	0.185846229	1
PREDNICARBATE	PC3	0.185958049	1
CLIDINIUM BROMIDE	HL60	0.186069916	1
CAPSAICIN	HL60	0.186293791	1
BUFLOMEDIL	HL60	0.186405799	1
PHENFORMIN	HL60	0.186517855	1
RIBAVIRIN	HL60	0.186742109	1
CLOXACILLIN	HL60	0.187191183	1
LINCOMYCIN	HL60	0.18730357	1
HEMICHOLINIUM	HL60	0.187416005	1
FIPEXIDE	MCF7	0.187641015	1
LANSOPRAZOLE	PC3	0.187753592	1
TIOGUANINE	MCF7	0.187866215	1
PRESTWICK-1100	PC3	0.187899868	1
NICLOSAMIDE	HL60	0.188091605	1
BETAHISTINE	MCF7	0.188218738	1
BETA-ESCIN	HL60	0.188317183	1
CLINDAMYCIN	HL60	0.188655907	1
CEFAMANDOLE	MCF7	0.188857615	1
BIOTIN	PC3	0.188881959	1
NORCYCLOBENZAPRINE	HL60	0.188995056	1

SIMVASTATIN	MCF7	0.189177623	1
ASCORBIC ACID	HL60	0.189334633	1
(-)-MK-801	HL60	0.189447919	1
BETAHISTINE	HL60	0.189561254	1
QUINIDINE	MCF7	0.189658345	1
BUTACAINE	HL60	0.189901541	1
BOLDINE	PC3	0.190015065	1
FOSFOSAL	HL60	0.190242255	1
FLORFENICOL	HL60	0.190355921	1
GINKGOLIDE A	PC3	0.190469635	1
ALIMEMAZINE	HL60	0.190583396	1
DIETHYLCARBAMAZINE	HL60	0.191152913	1
SANTONIN	MCF7	0.191266918	1
HARMINE	HL60	0.191266959	1
PENTOXYVERINE	HL60	0.191381052	1
CANADINE	PC3	0.191495193	1
AMRINONE	PC3	0.191723617	1
RACECADOTRIL	HL60	0.1918379	1
PIROMIDIC ACID	MCF7	0.191913005	1
AMIPRILOSE	HL60	0.191952231	1
CEFALEXIN	HL60	0.192295507	1
FENSPIRIDE	HL60	0.192410028	1
FLORFENICOL	PC3	0.192524596	1
ORCIPRENALINE	HL60	0.193098149	1
LYMECYCLINE	PC3	0.193213003	1
HARMALINE	HL60	0.193442852	1
BROMPERIDOL	HL60	0.193557848	1
LYCORINE	HL60	0.193672891	1
LATAMOXEF	PC3	0.194018306	1
BENPERIDOL	HL60	0.194248821	1
CHLOROPYRAZINE	HL60	0.194364149	1
METHYLBENZETHONIUM CHLORIDE	HL60	0.194479526	1
AMYLOCAINE	MCF7	0.194512559	1
EDROPHONIUM CHLORIDE	PC3	0.194675839	1
PARGYLINE	HL60	0.194710421	1
ROXITHROMYCIN	HL60	0.19482594	1
MEFLOQUINE	HL60	0.19505712	1
BENZATHINE BENZYLPENICILLIN	HL60	0.195172782	1
DIPERODON	PC3	0.195288491	1
CHLORPHENESIN	PC3	0.195404248	1
CEFORANIDE	PC3	0.195635905	1
ROLITETRACYCLINE	HL60	0.195751804	1
CAFFEIC ACID	HL60	0.195867751	1
MESTRANOL	HL60	0.195983746	1
SCOPOLAMINE N-OXIDE	HL60	0.196215879	1
SIMVASTATIN	HL60	0.196332016	1
PENTOXYVERINE	PC3	0.196448202	1
ELDELINE	HL60	0.196564435	1
MS-275	PC3	0.19664263	1
RIMEXOLONE	PC3	0.196680715	1
TRIFLUOPERAZINE	HL60	0.19675601	1
ACEBUTOLOL	MCF7	0.196807148	1
PERHEXILINE	HL60	0.197029843	1
QUINETHAZONE	MCF7	0.197796259	1
NISOXETINE	HL60	0.197962945	1
3-ACETAMIDOCOUMARIN	HL60	0.198079798	1
DL-ALPHA TOCOPHEROL	MCF7	0.198292101	1
CICLOHEXIMIDE	HL60	0.198430641	1
NORETYNODREL	PC3	0.198547684	1
SULFAMETHOXYPYRIDAZINE	MCF7	0.198886497	1
ROFECOXIB	PC3	0.199016332	1

MEPRYLCAINE	PC3	0.199133614	1
METOCLOPRAMIDE	HL60	0.199368319	1
PREDNISONE	MCF7	0.199452405	1
NITROFURANTOIN	HL60	0.199485744	1
MERCAPTOPURINE	MCF7	0.199603216	1
THIOPERAMIDE	HL60	0.200191293	1
DEXTROMETHORPHAN	HL60	0.200309051	1
NORFLOXACIN	PC3	0.200450671	1
FLUNARIZINE	HL60	0.200544711	1
NARINGIN	PC3	0.200780562	1
FLUOROMETHOLONE	HL60	0.200898559	1
SODIUM PHENYLBUTYRATE	SKMEL5	0.201016604	1
PALMATINE	HL60	0.201134696	1
CHENODEOXYCHOLIC ACID	HL60	0.201252837	1
NORTRIPTYLINE	HL60	0.20148926	1
IOPAMIDOL	HL60	0.201607544	1
IOVERSOL	HL60	0.202081156	1
DIAZOXIDE	HL60	0.202199678	1
AG-013608	MCF7	0.202396426	1
RESERPINE	HL60	0.203268528	1
PRESTWICK-642	HL60	0.203625671	1
HEXETIDINE	PC3	0.203864006	1
SULFAMERAZINE	HL60	0.203983245	1
MICONAZOLE	HL60	0.204221866	1
BETHANECHOL	MCF7	0.20430921	1
RIFAMPICIN	HL60	0.204580157	1
TRIMETAZIDINE	HL60	0.204699682	1
FLURBIPROFEN	PC3	0.204819256	1
DIHYDROERGOCRISTINE	MCF7	0.204985422	1
CARISOPRODOL	PC3	0.205058547	1
AMINOGLUTETHIMIDE	MCF7	0.205154714	1
GRISEOFULVIN	PC3	0.205493585	1
ESCULIN	HL60	0.205777566	1
PROADIFEN	HL60	0.20589757	1
CYPROHEPTADINE	HL60	0.206017622	1
QUINISOCAINE	HL60	0.206378065	1
SUCCINYLSULFATHIAZOLE	HL60	0.206618599	1
MEXILETINE	HL60	0.206859324	1
METIXENE	HL60	0.206979759	1
ETHAVERINE	HL60	0.207220772	1
URSODEOXYCHOLIC ACID	HL60	0.207461976	1
EPIANDROSTERONE	HL60	0.207703371	1
MEPHENYTOIN	PC3	0.207824141	1
MONORDEN	HL60	0.207905136	1
ETILEFRINE	HL60	0.208549763	1
FUSARIC ACID	HL60	0.208792021	1
SODIUM PHENYLBUTYRATE	MCF7	0.20879742	1
CARMUSTINE	PC3	0.208913221	1
DIGOXIGENIN	PC3	0.209155765	1
DOPAMINE	MCF7	0.20927711	1
PROGESTERONE	HL60	0.209641429	1
INDAPAMIDE	HL60	0.209762965	1
N6-METHYLADENOSINE	HL60	0.209884548	1
TETRACYCLINE	PC3	0.210105817	1
PIVMECILLINAM	HL60	0.210127859	1
AH-23848	MCF7	0.210277981	1
PHENELZINE	MCF7	0.210692537	1
NORETHISTERONE	HL60	0.210736974	1
AZACYCLONOL	HL60	0.211225128	1
GUANADREL	MCF7	0.211312978	1
METHYLPREDNISOLONE	HL60	0.211714049	1

DEBRISOQUINE	HL60	0.211958797	1
METFORMIN	PC3	0.212004892	1
MORACIZINE	HL60	0.212326278	1
INDAPAMIDE	PC3	0.212698341	1
HARMINE	PC3	0.212816924	1
ZIDOVUDINE	PC3	0.212939705	1
BIOTIN	HL60	0.213062534	1
CIPROFIBRATE	PC3	0.21343131	1
AG-012559	PC3	0.213567308	1
DIMETHADIONE	PC3	0.213923681	1
DIENESTROL	PC3	0.214170154	1
SELEGILINE	MCF7	0.214613233	1
0179445-0000	PC3	0.214748172	1
LOBELINE	HL60	0.214787177	1
0198306-0000	MCF7	0.215137491	1
TACRINE	PC3	0.215157965	1
DEPTROPINE	HL60	0.215281657	1
TROGLITAZONE	MCF7	0.215636615	1
PROFENAMINE	HL60	0.215653021	1
TIABENDAZOLE	MCF7	0.215837843	1
FLUCYTOSINE	PC3	0.216148845	1
ISOSORBIDE	MCF7	0.216188595	1
CEFOXITIN	HL60	0.216272921	1
ACONITINE	HL60	0.216397044	1
PYRAZINAMIDE	PC3	0.216521216	1
BISACODYL	MCF7	0.216539731	1
KANAMYCIN	PC3	0.216645436	1
EMETINE	MCF7	0.216715442	1
BEPHENIUM HYDROXYNAPHTHOATE	HL60	0.216894019	1
BENZATHINE BENZYLPENICILLIN	PC3	0.217267253	1
TRIFLURIDINE	HL60	0.217516316	1
7-AMINOCEPHALOSPORANIC ACID	MCF7	0.21759544	1
GELSEMINE	MCF7	0.21794811	1
MILRINONE	PC3	0.218139812	1
TROGLITAZONE	HL60	0.218226087	1
CINNARIZINE	HL60	0.218264656	1
SULFAMONOMETHOXINE	HL60	0.218389547	1
TRICHOSTATIN A	ssMCF7	0.218514486	1
S-PROPRANOLOL	HL60	0.218639473	1
VIDARABINE	PC3	0.218764508	1
TANESPIMYCIN	SKMEL5	0.219014722	1
EXISULIND	MCF7	0.21918548	1
PHTHALYLSULFATHIAZOLE	PC3	0.219265128	1
LEVCYCLOSERINE	PC3	0.220072194	1
KHELLIN	HL60	0.220394329	1
TOLAZAMIDE	HL60	0.220520036	1
EMETINE	PC3	0.220771593	1
PHENIRAMINE	PC3	0.220783292	1
DENATONIUM BENZOATE	HL60	0.221653555	1
PREDNISOLONE	HL60	0.221779741	1
PROXYPHYLLINE	HL60	0.222032258	1
MEPTAZINOL	PC3	0.222284967	1
DOMPERIDONE	HL60	0.222411393	1
FENOFIBRATE	HL60	0.22279096	1
DEXPANTHENOL	PC3	0.222917578	1
PROXYMETACAINE	HL60	0.223297721	1
AMPROLIUM	MCF7	0.223463588	1
HECOGENIN	HL60	0.223551389	1
SPIRONOLACTONE	HL60	0.223678296	1
PICOTAMIDE	HL60	0.22380525	1
NS-398	MCF7	0.224361819	1

PHENAZONE	HL60	0.22444074	1
SULFINPYRAZONE	HL60	0.224567982	1
FLUMETASONE	PC3	0.224721783	1
PROGLUMIDE	HL60	0.22482261	1
PRESTWICK-1084	PC3	0.22507743	1
TIAPRIDE	MCF7	0.225623373	1
BUTYL HYDROXYBENZOATE	HL60	0.225715319	1
SISOMICIN	PC3	0.225843041	1
FLUMETASONE	HL60	0.225970811	1
BUCLADESINE	HL60	0.226482369	1
INDOMETACIN	MCF7	0.226572911	1
ALVESPIMYCIN	PC3	0.226708448	1
THEOPHYLLINE	HL60	0.226738437	1
DL-PPMP	MCF7	0.22750779	1
METHYLPREDNISOLONE	MCF7	0.228342538	1
FENDILINE	HL60	0.228793883	1
MESORIDAZINE	PC3	0.228922756	1
SULFADIAZINE	HL60	0.229051677	1
SULFAGUANIDINE	MCF7	0.229253724	1
MESALAZINE	PC3	0.229309663	1
ETIFENIN	HL60	0.229438728	1
RISPERIDONE	MCF7	0.229984399	1
IOCETAMIC ACID	PC3	0.230343525	1
LINCOMYCIN	MCF7	0.230533413	1
XAMOTEROL	HL60	0.23060247	1
TETRANDRINE	PC3	0.230861607	1
NEOSTIGMINE BROMIDE	PC3	0.231120936	1
TOCAINIDE	HL60	0.231640169	1
PRESTWICK-1082	MCF7	0.232369694	1
CALCIUM PANTOTHENATE	HL60	0.232420457	1
AZATHIOPRINE	PC3	0.23255385	1
ERGOCALCIFEROL	HL60	0.232941607	1
DORZOLAMIDE	HL60	0.233463525	1
HARMOL	HL60	0.233594124	1
HALOFANTRINE	MCF7	0.233660797	1
CEFTAZIDIME	PC3	0.23398621	1
GRAMINE	PC3	0.234247839	1
TOLNAFTATE	HL60	0.234378726	1
TICLOPIDINE	MCF7	0.234956598	1
YOHIMBINE	HL60	0.235033879	1
ANTAZOLINE	MCF7	0.235142095	1
PNU-0293363	MCF7	0.235427545	1
MEPACRINE	HL60	0.235558863	1
ATRACURIUM BESILATE	PC3	0.235821643	1
RICININE	PC3	0.235953105	1
SULFAGUANIDINE	HL60	0.236216172	1
AMIPRILOSE	MCF7	0.236257094	1
BERGENIN	MCF7	0.236815887	1
MONOCROTALINE	MCF7	0.237002343	1
STAUROSPORINE	SKMEL5	0.237138416	1
SULFADIAZINE	MCF7	0.237188895	1
ETODOLAC	HL60	0.237270356	1
ACEBUTOLOL	HL60	0.237402345	1
PRESTWICK-920	PC3	0.237534381	1
NICLOSAMIDE	PC3	0.237749125	1
IOPROMIDE	HL60	0.237798598	1
NIFURTIMOX	HL60	0.238063006	1
KETANSERIN	MCF7	0.238123091	1
ACETYLSALICYLIC ACID	HL60	0.238218756	1
PENTAMIDINE	HL60	0.238459977	1
BUPROPION	MCF7	0.238684757	1

DIFLORASONE	PC3	0.238989942	1
PRENYLAMINE	PC3	0.239122553	1
SOLASODINE	HL60	0.239255212	1
LOXAPINE	HL60	0.239520673	1
PIROXICAM	PC3	0.239653475	1
ISOCONAZOLE	HL60	0.239919223	1
MEBHYDROLIN	MCF7	0.239998663	1
DIGITOXIGENIN	MCF7	0.240374926	1
ROLITETRACYCLINE	MCF7	0.240563201	1
CEFALONIUM	PC3	0.240850849	1
FUSARIC ACID	MCF7	0.240940039	1
AMINOCAPROIC ACID	HL60	0.24098413	1
CEFORANIDE	HL60	0.241117458	1
CLOMIFENE	MCF7	0.241506012	1
OXYBUPROCAINE	PC3	0.241651251	1
6-AZATHYMINE	PC3	0.241918434	1
METHYLPREDNISOLONE	PC3	0.242052097	1
PIROMIDIC ACID	HL60	0.242587229	1
SISOMICIN	HL60	0.242855081	1
PRESTWICK-682	PC3	0.242989079	1
NIZATIDINE	PC3	0.243123125	1
DEXTROMETHORPHAN	MCF7	0.243398807	1
AZAPERONE	HL60	0.243525549	1
PRESTWICK-1080	PC3	0.243588613	1
ERASTIN	PC3	0.243778514	1
LANATOSIDE C	PC3	0.24434879	1
NADIDE	PC3	0.244600782	1
EUCATROPINE	HL60	0.245274355	1
TONZONIUM BROMIDE	HL60	0.245814073	1
CYCLIZINE	HL60	0.245949122	1
ACETAZOLAMIDE	PC3	0.246354555	1
PRESTWICK-674	MCF7	0.246563045	1
ORPHENADRINE	PC3	0.247021448	1
AMIKACIN	HL60	0.24743781	1
SULFAPYRIDINE	HL60	0.247573431	1
SALSOLIDIN	MCF7	0.247596595	1
SIROLIMUS	ssMCF7	0.247844817	1
CHLOROQUINE	MCF7	0.2481726	1
GABEXATE	MCF7	0.248557081	1
METANEPHRINE	MCF7	0.248749464	1
MIMOSINE	PC3	0.248796171	1
PREGNENOLONE	PC3	0.24893227	1
PROPRANOLOL	HL60	0.249068416	1
PROPYLTHIOURACIL	HL60	0.249204609	1
N6-METHYLADENOSINE	PC3	0.24947714	1
VALINOMYCIN	PC3	0.250098818	1
BAS-012416453	PC3	0.2501593	1
URAPIDIL	PC3	0.250295875	1
IDOXURIDINE	MCF7	0.250678542	1
PARACETAMOL	PC3	0.250979464	1
RICININE	HL60	0.251664244	1
CP-320650-01	MCF7	0.251767625	1
AZLOCILLIN	PC3	0.252212925	1
PRESTWICK-1084	HL60	0.252350214	1
OXANTEL	PC3	0.252762367	1
7-AMINOCEPHALOSPORANIC ACID	HL60	0.253037374	1
MENADIONE	PC3	0.253174948	1
OXOLAMINE	HL60	0.25331257	1
ALPHA-YOHIMBINE	MCF7	0.253395255	1
0175029-0000	PC3	0.253416788	1
SEROTONIN	HL60	0.253725722	1

ZARDAVERINE	PC3	0.254139302	1
CARBINOXAMINE	HL60	0.254691408	1
ISONIAZID	HL60	0.254829553	1
PERGOLIDE	HL60	0.254967745	1
BUCLADESINE	PC3	0.255244273	1
QUERCETIN	HL60	0.255797899	1
GRISEOFULVIN	MCF7	0.255934613	1
PRESTWICK-920	HL60	0.256213617	1
PRIDINOL	HL60	0.256352284	1
0316684-0000	MCF7	0.256522899	1
STROPHANTHIDIN	HL60	0.256629762	1
H-89	PC3	0.256719184	1
PF-00539758-00	MCF7	0.256907429	1
PNU-0251126	MCF7	0.25711204	1
FENBENDAZOLE	HL60	0.257324287	1
CANAVANINE	PC3	0.257463334	1
TRIMETHADIONE	HL60	0.258019997	1
DANTROLENE	PC3	0.258095839	1
DEHYDROCHOLIC ACID	HL60	0.258437993	1
NALIDIXIC ACID	PC3	0.258687257	1
FURAZOLIDONE	PC3	0.258716894	1
ISOCONAZOLE	PC3	0.258884586	1
PIROXICAM	MCF7	0.259477142	1
THIOSTREPTON	PC3	0.259694539	1
ROXITHROMYCIN	MCF7	0.259872653	1
CARBENOXOLONE	PC3	0.260254236	1
MEPACRINE	MCF7	0.260394278	1
MEGLUMINE	MCF7	0.260664811	1
RETRORSINE	PC3	0.261375902	1
AZTREONAM	HL60	0.261656791	1
WORTMANNIN	MCF7	0.261690506	1
HOMOCHLORCYCLIZINE	PC3	0.261797307	1
8-AZAGUANINE	PC3	0.26193787	1
BUTYL HYDROXYBENZOATE	PC3	0.262359843	1
PRESTWICK-864	MCF7	0.262851057	1
SULOCTIDIL	PC3	0.263064077	1
0173570-0000	PC3	0.263455402	1
CLOBETASOL	PC3	0.263487185	1
BAMBUTEROL	PC3	0.263910717	1
NALOXONE	PC3	0.264048377	1
ETHOXYQUIN	HL60	0.264051989	1
BISACODYL	PC3	0.264193308	1
LEVOTHYROXINE SODIUM	MCF7	0.26444824	1
TIAPRIDE	HL60	0.264900612	1
CO-DERGOCRINE MESILATE	HL60	0.265183864	1
LOXAPINE	MCF7	0.265449548	1
METHOXAMINE	HL60	0.266176732	1
ETIFENIN	PC3	0.266318759	1
FAMOTIDINE	MCF7	0.266654232	1
LYNESTRENOL	HL60	0.266745122	1
CROMOGLICIC ACID	PC3	0.267599121	1
METRIFONATE	PC3	0.267660727	1
SITOSTEROL	PC3	0.267741619	1
BUTOCONAZOLE	MCF7	0.267862308	1
MELATONIN	PC3	0.268026756	1
BENZYLPENICILLIN	MCF7	0.268063984	1
BETAXOLOL	HL60	0.268169395	1
PRESTWICK-864	HL60	0.269026218	1
RIFAMPICIN	MCF7	0.26947835	1
KAEMPFEROL	HL60	0.269598374	1
FULVESTRANT	HL60	0.270102649	1

MONOBENZONE	HL60	0.270601457	1
R-ATENOLOL	MCF7	0.270897324	1
SPIRONOLACTONE	PC3	0.27110041	1
TICARCILLIN	MCF7	0.27117568	1
HALCINONIDE	PC3	0.27130359	1
TIRATRICOL	PC3	0.272038423	1
IDOXURIDINE	HL60	0.272182378	1
TRIAMCINOLONE	MCF7	0.272320899	1
DEXPROPRANOLOL	MCF7	0.27232638	1
NIFURTIMOX	MCF7	0.27293241	1
MOMETASONE	HL60	0.273191374	1
ISOMETHEPTENE	PC3	0.273335703	1
GUANETHIDINE	MCF7	0.274058055	1
BENZONATATE	PC3	0.27436255	1
TRIDIHEXETHYL	PC3	0.274492027	1
ATROPINE OXIDE	PC3	0.274772004	1
CYCLIC ADENOSINE MONOPHOSPHATE	PC3	0.274926421	1
NITROFURANTOIN	PC3	0.274976871	1
ETHOSUXIMIDE	HL60	0.275361236	1
HALOPERIDOL	HL60	0.275608834	1
GABEXATE	HL60	0.275796471	1
PRASTERONE	HL60	0.276086862	1
PRESTWICK-981	HL60	0.276232127	1
SALSOLIDIN	PC3	0.276813656	1
HYDROQUININE	PC3	0.277395932	1
DESOXYCORTONE	MCF7	0.277648656	1
CLOTRIMAZOLE	HL60	0.27768735	1
VELNACRINE	PC3	0.278124827	1
OXETACAINE	HL60	0.278270746	1
ORCIPRENALINE	PC3	0.278416711	1
AMPYRONE	HL60	0.279147238	1
ABAMECTIN	HL60	0.279293483	1
GLY-HIS-LYS	MCF7	0.279439775	1
ATROPINE	PC3	0.28046512	1
TERBUTALINE	HL60	0.280758494	1
SALSOLIDIN	HL60	0.280905251	1
FLUSPIRILENE	MCF7	0.281166317	1
CHLORHEXIDINE	HL60	0.281492744	1
SPECTINOMYCIN	PC3	0.282080981	1
CHLORCYCLIZINE	HL60	0.282375378	1
PUROMYCIN	HL60	0.282817321	1
OFLOXACIN	HL60	0.282964728	1
NALBUPHINE	PC3	0.283039494	1
PRESTWICK-1082	PC3	0.283997878	1
COLCHICINE	SKMEL5	0.28429348	1
TERBUTALINE	PC3	0.284885242	1
FLUPHENAZINE	PC3	0.284939005	1
ETHOSUXIMIDE	MCF7	0.285033298	1
PILOCARPINE	HL60	0.2851814	1
AMOXICILLIN	PC3	0.285774272	1
HARMINE	MCF7	0.286388132	1
TINIDAZOLE	PC3	0.286598209	1
FLUMEQUINE	HL60	0.286813578	1
ROFECOXIB	HL60	0.287557325	1
STOCK1N-28457	PC3	0.287855148	1
PRESTWICK-665	MCF7	0.287860608	1
PAROXETINE	PC3	0.288493054	1
METOLAZONE	MCF7	0.288915146	1
LEVONORGESTREL	PC3	0.28912633	1
AMOXAPINE	HL60	0.289795486	1
CHLOROPYRAZINE	PC3	0.290394079	1

ALPRENOLOL	HL60	0.290543842	1
MEFENAMIC ACID	PC3	0.290819118	1
CARCININE	PC3	0.291143354	1
DINOPROST	MCF7	0.291243235	1
PRALIDOXIME	HL60	0.291293347	1
GLYCOCHOLIC ACID	PC3	0.291443386	1
CARBARSONE	PC3	0.291743602	1
PRACTOLOL	PC3	0.292044002	1
BRETYLIUM TOSILATE	HL60	0.29219427	1
LM-1685	MCF7	0.292348107	1
DOXYLAMINE	PC3	0.29251779	1
BACLOFEN	HL60	0.292645351	1
COLECALCIFEROL	PC3	0.292946301	1
METHOXSALEN	MCF7	0.293369329	1
CALCIUM FOLINATE	MCF7	0.293582443	1
ISOPROPAMIDE IODIDE	HL60	0.294604802	1
SULFAMETHOXAZOLE	HL60	0.294906942	1
BUPIVACAINE	HL60	0.295058081	1
STOCK1N-35215	MCF7	0.295965874	1
ORLISTAT	MCF7	0.296095151	1
VIGABATRIN	MCF7	0.296268837	1
KHELLIN	MCF7	0.296361253	1
CAPTOPRIL	HL60	0.296420387	1
AMIKACIN	PC3	0.296571983	1
SPIRONOLACTONE	MCF7	0.296575648	1
DOXORUBICIN	PC3	0.296875311	1
FLAVOXATE	PC3	0.297330646	1
INDOPROFEN	PC3	0.297482515	1
SULFINPYRAZONE	PC3	0.29763443	1
MERCAPTOPURINE	PC3	0.29778639	1
GEMFIBROZIL	HL60	0.297938396	1
THIOGUANOSINE	PC3	0.298242545	1
CEFOTAXIME	PC3	0.298724608	1
GABAPENTIN	HL60	0.299156083	1
THEOBROMINE	HL60	0.299308498	1
NICOTINIC ACID	HL60	0.299460959	1
DANAZOL	HL60	0.299613466	1
ENOXACIN	PC3	0.299766018	1
PAROMOMYCIN	MCF7	0.300234295	1
GLYCOPYRRONIUM BROMIDE	PC3	0.300450329	1
PACLITAXEL	PC3	0.300666453	1
METOLAZONE	HL60	0.300682282	1
TETRAHYDROALSTONINE	PC3	0.300835152	1
CINCHONINE	PC3	0.300988067	1
TICARCILLIN	PC3	0.301141027	1
PROCHLORPERAZINE	HL60	0.301422323	1
SULFAQUINOXALINE	HL60	0.30160018	1
CLOFIBRATE	MCF7	0.301965101	1
GLAFENINE	HL60	0.302366342	1
PROCAINE	HL60	0.302673123	1
N-ACETYL-L-LEUCINE	MCF7	0.3030498	1
DIPIVEFRINE	PC3	0.303594553	1
EPITIOSTANOL	PC3	0.303748282	1
PIRINIXIC ACID	PC3	0.304136759	1
TRIMETHOPRIM	HL60	0.304209743	1
ETAMSYLATE	MCF7	0.304572176	1
OUABAIN	PC3	0.304671609	1
TIAPRIDE	PC3	0.304790019	1
NORFLOXACIN	HL60	0.304825655	1
ISOXSUPRINE	HL60	0.304979746	1
PROPAFENONE	HL60	0.305133882	1

PRALIDOXIME	PC3	0.305442289	1
FLUNIXIN	PC3	0.305444091	1
ARCAINE	MCF7	0.305662295	1
CAMPTOTHECIN	PC3	0.306059644	1
THIAMAZOLE	MCF7	0.306165019	1
CEFOTETAN	PC3	0.306214095	1
IMATINIB	PC3	0.306523132	1
NIFEDIPINE	PC3	0.306973411	1
BETAHISTINE	PC3	0.306987026	1
12,13-EODE	MCF7	0.307296513	1
AZATHIOPRINE	HL60	0.30760618	1
SULINDAC SULFIDE	MCF7	0.307761081	1
GELSEMINE	HL60	0.308071017	1
GLYCOPYRRONIUM BROMIDE	HL60	0.308381133	1
HYDROXYZINE	PC3	0.3087266	1
4,5-DIANILINOPHTHALIMIDE	PC3	0.309467951	1
MEGLUMINE	HL60	0.309778873	1
PEPSTATIN	PC3	0.310556962	1
PRESTWICK-1085	PC3	0.310712714	1
METHYLERGOMETRINE	MCF7	0.310926155	1
ALFUZOSIN	PC3	0.311024353	1
TIRATRICOL	HL60	0.311648165	1
PROPAFENONE	PC3	0.311960338	1
TERBUTALINE	MCF7	0.312029285	1
CORBADRINE	PC3	0.312428934	1
SERTACONAZOLE	PC3	0.313054351	1
CLENBUTEROL	PC3	0.313367327	1
ISOPROPAMIDE IODIDE	PC3	0.313680481	1
BEMEGRIDE	HL60	0.31462101	1
MEPENZOLATE BROMIDE	HL60	0.315406005	1
TRIMETAZIDINE	PC3	0.316192109	1
COLISTIN	PC3	0.316349463	1
FURAZOLIDONE	MCF7	0.31757831	1
CLIOQUINOL	PC3	0.317925433	1
AMIODARONE	PC3	0.318399086	1
GIBBERELLIC ACID	MCF7	0.318471294	1
PRESTWICK-857	HL60	0.318715075	1
AMOXICILLIN	HL60	0.318873136	1
CARISOPRODOL	MCF7	0.320037412	1
ACICLOVIR	MCF7	0.320717936	1
BECLOMETASONE	PC3	0.320931933	1
PRESTWICK-675	PC3	0.321158708	1
LOMEFLOXACIN	PC3	0.321607842	1
PHENELZINE	HL60	0.321725756	1
MELATONIN	HL60	0.322361605	1
LETROZOLE	PC3	0.322520676	1
CHLORAMPHENICOL	PC3	0.322957348	1
FISETIN	PC3	0.323316691	1
PRESTWICK-864	PC3	0.323476025	1
NETILMICIN	MCF7	0.323633284	1
LYSERGOL	PC3	0.324113799	1
MEPHENYTOIN	MCF7	0.324535755	1
DIHYDROERGOCRISTINE	HL60	0.325231585	1
AMBROXOL	PC3	0.325551345	1
ETILEFRINE	MCF7	0.326118439	1
SULFAPHENAZOLE	PC3	0.326191387	1
NARINGENIN	PC3	0.326671876	1
6-BROMOINDIRUBIN-3'-OXIME	MCF7	0.326794029	1
ROFECOXIB	MCF7	0.326812587	1
ISOETARINE	HL60	0.326832125	1
ENALAPRIL	PC3	0.326992419	1

SR-95531	PC3	0.327313135	1
PROGUANIL	MCF7	0.327478421	1
LASALOCID	HL60	0.328276327	1
CEFALONIUM	MCF7	0.328437011	1
NOMEGESTROL	PC3	0.328597738	1
PRESTWICK-967	PC3	0.328758508	1
ABAMECTIN	PC3	0.328919322	1
LACTOBIONIC ACID	PC3	0.329563009	1
PIOGLITAZONE	MCF7	0.329689149	1
DIMETHADIONE	HL60	0.329885112	1
ZAPRINAST	PC3	0.330207388	1
CANAVANINE	MCF7	0.330368591	1
PREGNENOLONE	MCF7	0.330663851	1
METANEPHRINE	HL60	0.330691126	1
ALPRENOLOL	PC3	0.331175252	1
NADOLOL	PC3	0.331336713	1
AMYLOCAINE	HL60	0.331659765	1
TRIBENOSIDE	PC3	0.332629954	1
SULFASALAZINE	PC3	0.332791803	1
CORALYNE	MCF7	0.332949519	1
THEOBROMINE	MCF7	0.334095576	1
GANCICLOVIR	MCF7	0.334784239	1
PIPERINE	MCF7	0.335243776	1
PRESTWICK-860	PC3	0.335387214	1
ETOFYLLINE	PC3	0.335473673	1
CEPHAELINE	PC3	0.335875073	1
FLUNARIZINE	PC3	0.336037778	1
IOPAMIDOL	MCF7	0.336163875	1
DEBRISOQUINE	PC3	0.336689025	1
SPECTINOMYCIN	HL60	0.337177907	1
CO-DERGOCRINE MESILATE	MCF7	0.337546583	1
LYMECYCLINE	MCF7	0.338008166	1
MORACIZINE	MCF7	0.338239086	1
PROPIDIUM IODIDE	PC3	0.338320122	1
BENZAMIL	MCF7	0.33856848	1
AMILORIDE	HL60	0.338646852	1
ATOVAQUONE	PC3	0.338810281	1
SULPIRIDE	MCF7	0.339163612	1
CHLORPROTHIXENE	PC3	0.339464419	1
IOBENGUANE	PC3	0.340119235	1
METHOCARBAMOL	PC3	0.340283045	1
16,16-DIMETHYLPROSTAGLANDIN E2	MCF7	0.340446897	1
DIPYRIDAMOLE	HL60	0.341595044	1
PROBENECID	PC3	0.341923466	1
ACEBUTOLOL	PC3	0.34217768	1
YOHIMBIC ACID	MCF7	0.342416415	1
ISOCARBOXAZID	PC3	0.343340734	1
ZUCLOPENTHIXOL	PC3	0.343403449	1
PSEUDOPELLETIERINE	PC3	0.343897533	1
FINASTERIDE	HL60	0.344062312	1
NOVOBIOCIN	SKMEL5	0.3445569	1
PHENYL BIGUANIDE	MCF7	0.344721846	1
FLUCYTOSINE	MCF7	0.34473917	1
BENZTHIAZIDE	MCF7	0.345205987	1
IOCETAMIC ACID	MCF7	0.345906839	1
SPIRAMYCIN	HL60	0.346373611	1
PRESTWICK-691	PC3	0.347035486	1
PROBENECID	HL60	0.347366673	1
PERPHENAZINE	HL60	0.347698028	1
DIGOXIN	MCF7	0.348248436	1
DIZOCILPINE	HL60	0.348361235	1

JOSAMYCIN	HL60	0.348693088	1
CINCHOCAINE	MCF7	0.348952538	1
HYDROCHLOROTHIAZIDE	HL60	0.349191179	1
OZAGREL	PC3	0.349357292	1
DESIPRAMINE	PC3	0.349689642	1
TOLBUTAMIDE	MCF7	0.350340452	1
ANISOMYCIN	PC3	0.350687688	1
LOVASTATIN	HL60	0.3510207	1
TAMOXIFEN	ssMCF7	0.351187268	1
CALYCANTHINE	HL60	0.351520529	1
ATRACTYLOSIDE	MCF7	0.351540625	1
COLISTIN	HL60	0.352020728	1
BUTACAINE	PC3	0.352187543	1
FENOTEROL	PC3	0.353523548	1
BUTAMBEN	MCF7	0.353665576	1
TRIMETHYLCOLCHICINIC ACID	HL60	0.353690734	1
DINOPROST	PC3	0.354359885	1
PIRINIXIC ACID	MCF7	0.354612134	1
FULVESTRANT	ssMCF7	0.355197246	1
H-7	PC3	0.356034426	1
TOBRAMYCIN	PC3	0.356035629	1
RALOXIFENE	MCF7	0.356358698	1
SYROSINGOPINE	HL60	0.356371267	1
0225151-0000	PC3	0.356509177	1
GELSEMINE	PC3	0.356875031	1
MIDODRINE	MCF7	0.357935383	1
METHAPYRILENE	PC3	0.358051903	1
ESTRIOL	PC3	0.358725295	1
LISINOPRIL	MCF7	0.359602984	1
PROFENAMINE	MCF7	0.359841536	1
FOSFOSAL	PC3	0.360074021	1
METYRAPONE	PC3	0.360242794	1
CP-645525-01	MCF7	0.360557678	1
AZTREONAM	MCF7	0.360796554	1
BENZTHIAZIDE	PC3	0.361087262	1
NALTREXONE	HL60	0.361256277	1
GLIBENCLAMIDE	MCF7	0.361274547	1
EQUILIN	PC3	0.361425331	1
PIVAMPICILLIN	PC3	0.361763562	1
CEFIXIME	PC3	0.362440504	1
3-NITROPROPIONIC ACID	PC3	0.362710455	1
IFENPRODIL	PC3	0.362779217	1
DICLOFENAMIDE	MCF7	0.362950054	1
IODIXANOL	MCF7	0.363429493	1
TOLAZAMIDE	MCF7	0.364475181	1
AMRINONE	MCF7	0.36462949	1
MECLOZINE	PC3	0.364644997	1
LORACARBEF	MCF7	0.36486973	1
FLUOROMETHOLONE	PC3	0.365664732	1
SULFADIMETHOXINE	MCF7	0.365831483	1
OMEPRAZOLE	PC3	0.365834827	1
PEPSTATIN	MCF7	0.366312837	1
CYPROTERONE	HL60	0.367708494	1
ALBENDAZOLE	HL60	0.368220326	1
CEFOPERAZONE	HL60	0.368391016	1
CINCHONINE	MCF7	0.368724367	1
DORZOLAMIDE	PC3	0.369245057	1
LIDOFLAZINE	MCF7	0.369691193	1
TODRALAZINE	MCF7	0.369933096	1
STREPTOMYCIN	HL60	0.370442369	1
ISOSORBIDE	HL60	0.370613571	1

CITIOLONE	MCF7	0.370752193	1
SCOPOLAMINE	HL60	0.370784812	1
KETOROLAC	HL60	0.370956093	1
RILMENIDINE	MCF7	0.371386166	1
PIRACETAM	PC3	0.372327746	1
MOROXYDINE	PC3	0.373084978	1
(+/-)-CATECHIN	MCF7	0.373571056	1
PIVAMPICILLIN	MCF7	0.373814212	1
KAWAIN	HL60	0.37456201	1
TAMOXIFEN	HL60	0.374734149	1
FOLIC ACID	MCF7	0.374787612	1
AG-013608	PC3	0.375414465	1
7-AMINOCEPHALOSPORANIC ACID	PC3	0.375595424	1
PARGYLINE	PC3	0.376285142	1
LIOTHYRONINE	PC3	0.376630232	1
ETHIONAMIDE	PC3	0.377493633	1
BENZETHONIUM CHLORIDE	HL60	0.377839263	1
PICOTAMIDE	MCF7	0.37795972	1
15-DELTA PROSTAGLANDIN J2	PC3	0.378505075	1
THIOPROPERAZINE	PC3	0.378693595	1
MICONAZOLE	PC3	0.378938374	1
DOBUTAMINE	PC3	0.380089601	1
MESORIDAZINE	HL60	0.380436381	1
PIRIBEDIL	PC3	0.380609828	1
PHYSOSTIGMINE	HL60	0.380783313	1
LOBELANIDINE	PC3	0.381651311	1
MECLOCYCLINE	PC3	0.381825025	1
TOLFENAMIC ACID	HL60	0.382694163	1
IOPROMIDE	MCF7	0.383111292	1
DIPHENHYDRAMINE	MCF7	0.383849969	1
PROXYPHYLLINE	PC3	0.385307251	1
BAMBUTEROL	MCF7	0.385576189	1
KETOPROFEN	MCF7	0.386638517	1
NAFTIDROFURYL	PC3	0.387054014	1
PRESTWICK-642	MCF7	0.387058737	1
LEFLUNOMIDE	PC3	0.387228896	1
METHANTHELINIUM BROMIDE	PC3	0.387578772	1
CIMETIDINE	PC3	0.388048599	1
PARAROSANILINE	MCF7	0.388103867	1
CHLOROPYRAMINE	HL60	0.388629297	1
PYRIMETHAMINE	PC3	0.389783725	1
METHYLBENZETHONIUM CHLORIDE	PC3	0.390528463	1
NS-398	PC3	0.390558733	1
BETULINIC ACID	MCF7	0.391771172	1
LEVAMISOLE	HL60	0.392140691	1
DEMECARIUM BROMIDE	PC3	0.392316648	1
SULMAZOLE	HL60	0.392492642	1
PROPRANOLOL	MCF7	0.392766666	1
IC-86621	MCF7	0.393015723	1
ASCORBIC ACID	PC3	0.393196985	1
DEMECLOCYCLINE	MCF7	0.39332641	1
ATROPINE METHONITRATE	PC3	0.394254599	1
QUINISOCAINE	PC3	0.394607429	1
NARINGIN	MCF7	0.39526053	1
CLOTRIMAZOLE	MCF7	0.396077768	1
NAFTOPIDIL	MCF7	0.396196969	1
TYLOXAPOL	PC3	0.396550599	1
DEXAMETHASONE	HL60	0.397258295	1
OXAPROZIN	MCF7	0.397431934	1
HESPERIDIN	PC3	0.39761236	1
FASUDIL	MCF7	0.397789447	1

PROBUCOL	MCF7	0.398140524	1
ETHAMBUTOL	PC3	0.398513427	1
GABAPENTIN	PC3	0.398675421	1
CAFFEIC ACID	MCF7	0.399030062	1
VALPROIC ACID	ssMCF7	0.399384847	1
ILOPROST	SKMEL5	0.399739776	1
HYDRALAZINE	MCF7	0.399759954	1
FLUTAMIDE	HL60	0.400094848	1
PARACETAMOL	MCF7	0.400270036	1
MONOCROTALINE	PC3	0.400272438	1
DEXAMETHASONE	ssMCF7	0.400450063	1
BETULINIC ACID	PC3	0.401160923	1
PENTETIC ACID	MCF7	0.401606867	1
BUTIROSIN	MCF7	0.401778492	1
LOMEFLOXACIN	MCF7	0.401915061	1
TRIAMCINOLONE	HL60	0.402584352	1
ALPHA-ERGOCRYPTINE	PC3	0.402785554	1
SUPROFEN	MCF7	0.403037497	1
NAPHAZOLINE	HL60	0.403475149	1
ESTRADIOL	ssMCF7	0.403541595	1
PERHEXILINE	MCF7	0.40379375	1
CALYCANTHINE	MCF7	0.404045976	1
LY-294002	ssMCF7	0.404188423	1
HEXYLCAINE	MCF7	0.405308164	1
1,5-ISOQUINOLINEDIOL	HL60	0.406152834	1
PRESTWICK-689	PC3	0.406689319	1
AJMALINE	MCF7	0.406825107	1
CONVOLAMINE	PC3	0.406868217	1
TROLOX C	PC3	0.40704715	1
OXOLAMINE	MCF7	0.407078176	1
ALVERINE	MCF7	0.407226118	1
VALDECOXIB	PC3	0.407837801	1
STOCK1N-35696	MCF7	0.408121482	1
SUCCINYLSULFATHIAZOLE	MCF7	0.408851609	1
BUMETANIDE	MCF7	0.409612694	1
AMBROXOL	MCF7	0.409866527	1
OXYMETAZOLINE	PC3	0.410628441	1
TETRYZOLINE	HL60	0.410633114	1
ANISOMYCIN	MCF7	0.41088255	1
PERGOLIDE	PC3	0.410992472	1
IMIPRAMINE	MCF7	0.411390975	1
ANTAZOLINE	PC3	0.411711599	1
TRIFLUPROMAZINE	PC3	0.411891466	1
NAFCILLIN	PC3	0.412071368	1
TRIAMCINOLONE	PC3	0.412154128	1
BEMEGRIDE	PC3	0.412971391	1
FLUCYTOSINE	HL60	0.413331639	1
FLUOROCURARINE	PC3	0.413511814	1
METERGOLINE	HL60	0.414052544	1
ALPROSTADIL	PC3	0.414954094	1
FLUPHENAZINE	SKMEL5	0.41495444	1
NIMODIPINE	MCF7	0.415212884	1
METHYLERGOMETRINE	PC3	0.415315435	1
PIPERIDOLATE	HL60	0.415676566	1
TETROQUINONE	PC3	0.415857182	1
LY-294002	SKMEL5	0.416760769	1
ALPHA-ERGOCRYPTINE	MCF7	0.416936079	1
DOXYLAMINE	HL60	0.416941587	1
BETHANECHOL	PC3	0.417122439	1
BEPHENIUM HYDROXYNAPHTHOATE	PC3	0.417484243	1
ISOXICAM	MCF7	0.418025308	1

DROPERIDOL	HL60	0.418208254	1
AMINOHIPPURIC ACID	PC3	0.418389341	1
NICARDIPINE	MCF7	0.418793742	1
IVERMECTIN	HL60	0.4189328	1
DOXYCYCLINE	PC3	0.41947656	1
WORTMANNIN	ssMCF7	0.420746495	1
CLOMIPRAMINE	HL60	0.42129125	1
SULFAMETOXYDIAZINE	PC3	0.421654585	1
DIPIVEFRINE	MCF7	0.421873464	1
XYLOMETAZOLINE	HL60	0.422199835	1
BAS-012416453	MCF7	0.422387677	1
PENTOXIFYLLINE	HL60	0.422563499	1
AZACITIDINE	MCF7	0.422902152	1
PILOCARPINE	PC3	0.423473234	1
PARGYLINE	MCF7	0.423674358	1
AZAPERONE	PC3	0.423837357	1
GLIBENCLAMIDE	PC3	0.424383786	1
ISONIAZID	PC3	0.424447151	1
RAMIPRIL	PC3	0.425295154	1
LANSOPRAZOLE	HL60	0.425659928	1
SULFAMONOMETHOXINE	MCF7	0.425736443	1
FLUDROCORTISONE	PC3	0.425994496	1
BENZTHIAZIDE	HL60	0.426207333	1
MINOXIDIL	PC3	0.427285731	1
BROMPHENIRAMINE	HL60	0.427303015	1
BENSERAZIDE	MCF7	0.427961211	1
NORETHISTERONE	PC3	0.429131723	1
DICYCLOVERINE	MCF7	0.429355052	1
ETICLOPRIDE	PC3	0.431146988	1
BENZYDAMINE	MCF7	0.431169058	1
ACETOHEXAMIDE	HL60	0.431330385	1
BUFEXAMAC	PC3	0.431697272	1
FURALTADONE	PC3	0.433505905	1
COLECALCIFEROL	MCF7	0.433765868	1
THIOPERAMIDE	MCF7	0.434208412	1
DEXTROMETHORPHAN	PC3	0.434269008	1
L-METHIONINE SULFOXIMINE	MCF7	0.434806348	1
ATROPINE OXIDE	MCF7	0.435066624	1
BENFOTIAMINE	PC3	0.43518897	1
FAMPROFAZONE	MCF7	0.435483496	1
TROLOX C	HL60	0.4359255	1
GELDANAMYCIN	PC3	0.436108348	1
BISOPROLOL	PC3	0.43610971	1
LEVOCABASTINE	PC3	0.436662525	1
FIPEXIDE	HL60	0.437031223	1
CYANOCOBALAMIN	MCF7	0.437672789	1
PERHEXILINE	PC3	0.437768987	1
SPARTEINE	HL60	0.437953505	1
MG-262	PC3	0.438194761	1
16-PHENYLTETRANORPROSTAGLANDIN E2	MCF7	0.438194761	1
CHLORTALIDONE	MCF7	0.43845584	1
TRIMETHYLCOLCHICINIC ACID	PC3	0.438691884	1
PROPOFOL	PC3	0.439800367	1
BETONICINE	HL60	0.44035502	1
SC-560	MCF7	0.441331722	1
TOLAZAMIDE	PC3	0.441650269	1
TETRAETHYLENEPENTAMINE	MCF7	0.442950887	1
MORACIZINE	PC3	0.443132361	1
CHLORCYCLIZINE	PC3	0.444214908	1
BUSPIRONE	PC3	0.444245187	1
STACHYDRINE	MCF7	0.445002486	1

SULFAMETHIZOLE	HL60	0.445916432	1
PF-00875133-00	PC3	0.446102274	1
LEVODOPA	MCF7	0.446579239	1
METHOTREXATE	PC3	0.446703036	1
PSEUDOPELLETIERINE	MCF7	0.447105295	1
PROXYMETACAINE	PC3	0.447217942	1
FLUPHENAZINE	HL60	0.447226936	1
CLOMIFENE	PC3	0.447962307	1
MEBENDAZOLE	PC3	0.448158109	1
PRESTWICK-674	PC3	0.448421459	1
NEFOPAM	MCF7	0.4486374	1
ERGOCALCIFEROL	MCF7	0.448684867	1
3-ACETYLCOUMARIN	PC3	0.44907973	1
MENADIONE	HL60	0.449266069	1
OLEANDOMYCIN	PC3	0.449475439	1
CONVOLAMINE	HL60	0.449638834	1
0198306-0000	PC3	0.449739079	1
HYDRASTININE	MCF7	0.450530347	1
HARMOL	PC3	0.450757822	1
ETYNODIOL	MCF7	0.452114435	1
PHTHALYLSULFATHIAZOLE	MCF7	0.452515524	1
SULFAGUANIDINE	PC3	0.452907251	1
GALANTAMINE	HL60	0.453185841	1
FENSPIRIDE	PC3	0.45343608	1
LYCORINE	PC3	0.45355981	1
DROPERIDOL	MCF7	0.45370058	1
ETACRYNIC ACID	HL60	0.453746837	1
F0447-0125	PC3	0.453965136	1
PIPERACETAZINE	MCF7	0.454759144	1
FENOTEROL	MCF7	0.454869593	1
(-)-ATENOLOL	MCF7	0.45581861	1
ETHISTERONE	HL60	0.456180755	1
NAFCILLIN	MCF7	0.456613797	1
METRONIDAZOLE	HL60	0.456930605	1
CO-DERGOCRINE MESILATE	PC3	0.457118138	1
BEMEGRIDE	MCF7	0.457144201	1
PROBENECID	MCF7	0.457409486	1
TROPICAMIDE	PC3	0.457940224	1
ENILCONAZOLE	MCF7	0.458205675	1
HYDROXYACHILLIN	PC3	0.458243914	1
CLOZAPINE	MCF7	0.458801545	1
NIMODIPINE	PC3	0.458807176	1
10-METHOXYHARMALAN	HL60	0.458994985	1
ZAPRINAST	MCF7	0.459002362	1
AMPICILLIN	MCF7	0.459533762	1
ROSIGLITAZONE	MCF7	0.459618393	1
NORFLOXACIN	MCF7	0.459799544	1
THIOPROPERAZINE	MCF7	0.460065381	1
LABETALOL	PC3	0.460122416	1
SALSOLINOL	PC3	0.460310417	1
ALTIZIDE	PC3	0.460686501	1
ALTIZIDE	MCF7	0.460863219	1
RESERPINE	PC3	0.461062693	1
ISRADIPINE	PC3	0.462003651	1
GRISEOFULVIN	HL60	0.462380223	1
LEVOPROPOXYPHENE	PC3	0.46256855	1
MESORIDAZINE	MCF7	0.462726744	1
BROMOPRIDE	PC3	0.462726744	1
PF-00875133-00	MCF7	0.463259666	1
N-ACETYLMURAMIC ACID	PC3	0.464076131	1
BENZYDAMINE	HL60	0.464830564	1

LYSERGOL	MCF7	0.465660462	1
DIGITOXIGENIN	PC3	0.46596301	1
DYDROGESTERONE	HL60	0.466151844	1
STREPTOZOCIN	MCF7	0.466194556	1
ACETYLSALICYLIC ACID	MCF7	0.466378714	1
INDOMETACIN	PC3	0.466461683	1
MOROXYDINE	MCF7	0.466728862	1
BACAMPICILLIN	PC3	0.467096407	1
BENZBROMARONE	MCF7	0.467285399	1
STOCK1N-28457	MCF7	0.467530715	1
0297417-0002B	PC3	0.46766346	1
BEPHENIUM HYDROXYNAPHTHOATE	MCF7	0.468148605	1
TIABENDAZOLE	PC3	0.468419897	1
SC-58125	SKMEL5	0.468798272	1
ATROPINE	MCF7	0.469135832	1
VIDARABINE	HL60	0.470312807	1
MECLOFENAMIC ACID	MCF7	0.470474855	1
SODIUM PHENYLBUTYRATE	PC3	0.471260229	1
PALMATINE	MCF7	0.471547	1
PIMOZIDE	PC3	0.471639377	1
MOMETASONE	MCF7	0.471815164	1
NOSCAPINE	PC3	0.472018626	1
10-METHOXYHARMALAN	MCF7	0.472888329	1
NICLOSAMIDE	MCF7	0.473156747	1
HARMALINE	PC3	0.473916398	1
FENOPROFEN	MCF7	0.474387442	1
MINOXIDIL	HL60	0.474866228	1
ZOMEPIRAC	PC3	0.475816683	1
LEFLUNOMIDE	MCF7	0.476650748	1
BERBERINE	PC3	0.477148363	1
3-NITROPROPIONIC ACID	MCF7	0.477458249	1
TRIMETHOPRIM	PC3	0.477727514	1
PROPANTHELINE BROMIDE	MCF7	0.477996829	1
PHENINDIONE	PC3	0.478481248	1
SERTACONAZOLE	MCF7	0.478805065	1
VALPROIC ACID	SKMEL5	0.479434042	1
TYLOXAPOL	MCF7	0.479883395	1
CP-863187	MCF7	0.480153099	1
NICERGOLINE	HL60	0.480768971	1
NIRIDAZOLE	PC3	0.480959771	1
PICROTOXININ	PC3	0.481150596	1
SCOPOLAMINE	MCF7	0.481232395	1
MYRICETIN	HL60	0.481341444	1
NICARDIPINE	PC3	0.481723212	1
THIAMAZOLE	PC3	0.482312458	1
LUTEOLIN	MCF7	0.482852775	1
TOLAZOLINE	HL60	0.48286909	1
MEROPENEM	PC3	0.483060152	1
FLUNISOLIDE	PC3	0.483123004	1
TYRPHOSTIN AG-1478	MCF7	0.483633482	1
SULPIRIDE	PC3	0.483933976	1
DIRITHROMYCIN	MCF7	0.484204394	1
METRIZAMIDE	PC3	0.484207023	1
VALINOMYCIN	MCF7	0.484474859	1
THAPSIGARGIN	PC3	0.485163394	1
HEMICHOLINIUM	PC3	0.485354738	1
HEXAMETHONIUM BROMIDE	PC3	0.485557186	1
NAFCILLIN	HL60	0.485737496	1
CARBINOXAMINE	MCF7	0.48691114	1
RALOXIFENE	PC3	0.487844318	1
PRESTWICK-664	MCF7	0.487941254	1

TRIOXYSALEN	MCF7	0.487995132	1
GUANFACINE	PC3	0.488419387	1
CP-690334-01	MCF7	0.489206215	1
L-METHIONINE SULFOXIMINE	PC3	0.48976201	1
PRESTWICK-691	MCF7	0.489893871	1
6-BENZYLAMINOPURINE	MCF7	0.48993467	1
REPAGLINIDE	PC3	0.49014582	1
PRESTWICK-984	MCF7	0.490979857	1
PROTOVERATRINE A	PC3	0.492258371	1
TRIAMTERENE	PC3	0.492338343	1
MECLOCYCLINE	MCF7	0.493153965	1
THALIDOMIDE	HL60	0.493219508	1
VALPROIC ACID	MCF7	0.493773517	1
CINNARIZINE	PC3	0.493988813	1
AJMALINE	HL60	0.494181194	1
DIPHENYLPYRALINE	PC3	0.494242076	1
CYCLIZINE	MCF7	0.494514213	1
RAMIFENAZONE	PC3	0.494566021	1
MEFENAMIC ACID	MCF7	0.495875546	1
DIMENHYDRINATE	MCF7	0.496147942	1
MOLINDONE	PC3	0.496298812	1
GUANETHIDINE	PC3	0.496684113	1
N-ACETYL-L-ASPARTIC ACID	MCF7	0.496692862	1
MEPHENESIN	MCF7	0.497783214	1
RAUBASINE	HL60	0.498033337	1
CANRENOIC ACID	MCF7	0.498328644	1
BUFLOMEDIL	PC3	0.49841902	1
FLUFENAMIC ACID	PC3	0.498601422	1
STOCK1N-35874	PC3	0.499420009	1
CLONIDINE	HL60	0.499769572	1
MEBEVERINE	HL60	0.500348692	1
FLORFENICOL	MCF7	0.500512042	1
TAMOXIFEN	PC3	0.501121143	1
CALCIUM PANTOTHENATE	PC3	0.501314307	1
JOSAMYCIN	MCF7	0.501604737	1
GABEXATE	PC3	0.501700697	1
PIPEMIDIC ACID	MCF7	0.502087169	1
DIPHEMANIL METILSULFATE	PC3	0.502424689	1
BENZAMIL	PC3	0.502424689	1
BACITRACIN	HL60	0.502473722	1
EPITIOSTANOL	MCF7	0.502971527	1
NORCYCLOBENZAPRINE	PC3	0.503053705	1
CHLORAMBUCIL	MCF7	0.504339329	1
NIMODIPINE	HL60	0.504601212	1
NIALAMIDE	PC3	0.50461301	1
CEFEPIME	HL60	0.504794741	1
PIPEMIDIC ACID	PC3	0.505181858	1
CEFMETAZOLE	MCF7	0.505434292	1
STOCK1N-35215	PC3	0.506255931	1
ISOFLUPREDONE	PC3	0.506343685	1
FUSIDIC ACID	PC3	0.507312415	1
KHELLIN	PC3	0.507352	1
ETHOXYQUIN	PC3	0.507626115	1
BENDROFLUMETHIAZIDE	HL60	0.508281632	1
CEFOXITIN	PC3	0.508475534	1
PRONETALOL	MCF7	0.509271617	1
PRESTWICK-692	PC3	0.509639346	1
NIFEDIPINE	MCF7	0.509664384	1
SANTONIN	PC3	0.510369385	1
PRIMIDONE	MCF7	0.510643922	1
NABUMETONE	PC3	0.510803846	1

CARBACHOL	PC3	0.511192163	1
SULCONAZOLE	PC3	0.51138635	1
MYCOPHENOLIC ACID	PC3	0.511969024	1
MOXISYLYTE	PC3	0.512017172	1
NIFENAZONE	MCF7	0.513391355	1
AZAPROPAZONE	HL60	0.513523639	1
LORACARBEF	PC3	0.514690373	1
PROXYMETACAINE	MCF7	0.514766459	1
PYRVINIUM	PC3	0.51546856	1
PRILOCAINE	HL60	0.515663152	1
SIMVASTATIN	PC3	0.515857762	1
KETOCONAZOLE	MCF7	0.515867196	1
3-HYDROXY-DL-KYNURENINE	MCF7	0.515884409	1
ARACHIDONIC ACID	MCF7	0.516056651	1
GW-8510	MCF7	0.51641778	1
PHENSUXIMIDE	MCF7	0.517243925	1
DIZOCILPINE	MCF7	0.517519377	1
VERTEPORFIN	MCF7	0.517519377	1
CEFALEXIN	MCF7	0.517743204	1
HELIOTRINE	PC3	0.517794865	1
PF-00539745-00	PC3	0.518070388	1
0317956-0000	MCF7	0.518611522	1
METIXENE	MCF7	0.518621539	1
FLAVOXATE	MCF7	0.51917283	1
BETAMETHASONE	MCF7	0.519753684	1
PROCYCLIDINE	MCF7	0.520000027	1
CLORSULON	HL60	0.521118902	1
STOCK1N-35696	PC3	0.522094568	1
ERYTHROMYCIN	MCF7	0.522483466	1
AMODIAQUINE	HL60	0.523070656	1
SB-202190	MCF7	0.5232617	1
METHOXSALEN	PC3	0.523265923	1
PRESTWICK-972	MCF7	0.523461207	1
FIPEXIDE	PC3	0.523656508	1
SULFAFURAZOLE	HL60	0.524047159	1
MORANTEL	PC3	0.52441691	1
DISULFIRAM	PC3	0.52552246	1
ZIDOVUDINE	MCF7	0.525798929	1
SCOPOLETIN	MCF7	0.5258059	1
MEGLUMINE	PC3	0.526001397	1
TRANYLCYPROMINE	MCF7	0.526351963	1
OXYMETAZOLINE	MCF7	0.526392439	1
SODIUM PHENYLBUTYRATE	HL60	0.526628528	1
CLIDINIUM BROMIDE	MCF7	0.526905125	1
GUAIFENESIN	PC3	0.526905125	1
URSODEOXYCHOLIC ACID	PC3	0.527370325	1
PNU-0251126	PC3	0.527987021	1
CAPTOPRIL	MCF7	0.528288588	1
TROPINE	PC3	0.52854431	1
KETOTIFEN	MCF7	0.528565375	1
DOXEPIN	MCF7	0.528935764	1
NILUTAMIDE	MCF7	0.529395924	1
MEPYRAMINE	PC3	0.529523062	1
RIFABUTIN	MCF7	0.529672835	1
PENTOLONIUM	HL60	0.530893962	1
CARCININE	MCF7	0.531057854	1
IC-86621	PC3	0.531612075	1
FLUTICASONE	PC3	0.531873634	1
PROTOVERATRINE A	MCF7	0.532166416	1
METOCLOPRAMIDE	PC3	0.532166416	1
EBSELEN	PC3	0.532265608	1

FLUNIXIN	MCF7	0.532720878	1
DIGOXIN	PC3	0.532853681	1
RONIDAZOLE	PC3	0.533441888	1
DEXIBUPROFEN	MCF7	0.533830156	1
KARAKOLINE	PC3	0.534107549	1
PAPAVERINE	PC3	0.534618698	1
VIDARABINE	MCF7	0.534662423	1
CELECOXIB	PC3	0.535599772	1
SR-95639A	PC3	0.536188589	1
SB-203580	PC3	0.536327737	1
ALPRENOLOL	MCF7	0.536605389	1
(+)-ISOPRENALINE	PC3	0.536973877	1
GEMFIBROZIL	PC3	0.537994073	1
GLYCOPYRRONIUM BROMIDE	MCF7	0.537994073	1
BETULINIC ACID	HL60	0.53815223	1
ISOTRETINOIN	MCF7	0.538271893	1
MECLOCYCLINE	HL60	0.538348671	1
PHA-00851261E	MCF7	0.538577631	1
PRAZIQUANTEL	MCF7	0.538827616	1
CHLORTETRACYCLINE	MCF7	0.539105519	1
PRASTERONE	PC3	0.539134572	1
METHYLBENZETHONIUM CHLORIDE	MCF7	0.539439757	1
SOLANINE	PC3	0.540117255	1
OXOLINIC ACID	PC3	0.540773507	1
NADIDE	MCF7	0.541051599	1
AZACITIDINE	PC3	0.542083626	1
AZACYCLONOL	PC3	0.542164232	1
CLEMIZOLE	HL60	0.542477059	1
TRIHEXYPHENIDYL	PC3	0.544051308	1
NEOMYCIN	PC3	0.544112343	1
CIPROFIBRATE	MCF7	0.544390747	1
LOBELINE	MCF7	0.54522611	1
PROCYCLIDINE	PC3	0.545232529	1
BEPRIDIL	PC3	0.54562637	1
PIRETANIDE	HL60	0.545823309	1
HYDRASTINE HYDROCHLORIDE	MCF7	0.546061699	1
ILOPROST	MCF7	0.546340278	1
SULFATHIAZOLE	HL60	0.5464142	1
OXYPHENBUTAZONE	MCF7	0.546618881	1
KETANSERIN	PC3	0.546808189	1
MEPENZOLATE BROMIDE	PC3	0.547176162	1
1,4-CHRYSENEQUINONE	PC3	0.547202227	1
TRIFLUSAL	MCF7	0.547454838	1
GALANTAMINE	PC3	0.547596313	1
CLOFILIUM TOSYLATE	MCF7	0.548291012	1
SPIPERONE	PC3	0.549370292	1
CIPROFLOXACIN	MCF7	0.549685111	1
GLIPIZIDE	MCF7	0.549964	1
PRESTWICK-1085	MCF7	0.550242913	1
MYRICETIN	MCF7	0.55052185	1
HYDROCORTISONE	PC3	0.550947959	1
KETOROLAC	MCF7	0.55107979	1
TRICHLORMETHIAZIDE	PC3	0.551145218	1
FENOPROFEN	PC3	0.551358795	1
PHENFORMIN	MCF7	0.552365064	1
ETIDRONIC ACID	MCF7	0.552475038	1
TACROLIMUS	MCF7	0.552518296	1
FLECAINIDE	MCF7	0.552860178	1
DICOUMAROL	MCF7	0.553202093	1
PRESTWICK-1083	PC3	0.553513212	1
ACONITINE	PC3	0.554302894	1

PHENINDIONE	MCF7	0.554429314	1
ATROPINE METHONITRATE	MCF7	0.554895269	1
CHLOROQUINE	PC3	0.555290239	1
HYDROXYZINE	HL60	0.55548774	1
NOSCAPINE	MCF7	0.555546517	1
LABETALOL	MCF7	0.556105245	1
PIPERIDOLATE	MCF7	0.55638464	1
NIMESULIDE	PC3	0.5564754	1
SCOPOLAMINE	PC3	0.556870537	1
PRESTWICK-967	MCF7	0.556943491	1
ROXARSONE	PC3	0.55706812	1
IVERMECTIN	PC3	0.557502425	1
PRILOCAINE	PC3	0.557502425	1
MINAPRINE	PC3	0.557781922	1
BUDESONIDE	MCF7	0.558620534	1
BCB000040	PC3	0.558620534	1
NITROFURAL	PC3	0.558846827	1
NICERGOLINE	PC3	0.558900111	1
PRESTWICK-689	MCF7	0.559179708	1
BENZBROMARONE	PC3	0.559242204	1
IOPANOIC ACID	PC3	0.560033075	1
MEBEVERINE	PC3	0.560230817	1
AMINOPHENAZONE	HL60	0.56062633	1
PROPIDIUM IODIDE	MCF7	0.561417175	1
PRESTWICK-559	HL60	0.561417469	1
DAPSONE	MCF7	0.561696943	1
FLUVOXAMINE	PC3	0.561813094	1
PROADIFEN	PC3	0.562208757	1
TIMOLOL	MCF7	0.562331308	1
DOXAZOSIN	PC3	0.562406602	1
IFOSFAMIDE	MCF7	0.56253636	1
ROLITETRACYCLINE	PC3	0.562604456	1
SUXIBUZONE	PC3	0.562802319	1
DEPTROPINE	MCF7	0.562816202	1
PF-00539745-00	MCF7	0.563000192	1
ETHAMBUTOL	MCF7	0.563375941	1
CYCLIC ADENOSINE MONOPHOSPHATE	MCF7	0.563655837	1
SB-202190	PC3	0.563655837	1
CHLORPHENESIN	MCF7	0.563935751	1
VANCOMYCIN	MCF7	0.564215682	1
DESOXYCORTONE	PC3	0.564583492	1
ESTROPIPATE	MCF7	0.564775598	1
HEXESTROL	MCF7	0.564775598	1
PUROMYCIN	MCF7	0.565615601	1
BUDESONIDE	PC3	0.566167348	1
HEPTAMINOL	MCF7	0.566455757	1
CROTAMITON	MCF7	0.566735842	1
OXOLINIC ACID	MCF7	0.567296063	1
MEMANTINE	PC3	0.567553664	1
MERBROMIN	PC3	0.568696898	1
ETANIDAZOLE	PC3	0.569336651	1
ALVESPIMYCIN	MCF7	0.569792487	1
FENBUFEN	HL60	0.570327473	1
LOVASTATIN	MCF7	0.570378418	1
DEXIBUPROFEN	PC3	0.57052566	1
CORYNANTHINE	MCF7	0.570939047	1
TERCONAZOLE	MCF7	0.571499735	1
ROLIPRAM	MCF7	0.571780101	1
AMIPRILOSE	PC3	0.571913178	1
DICLOXACILLIN	PC3	0.572111424	1
4-HYDROXYPHENAZONE	PC3	0.572340876	1

TRANYLCYPROMINE	PC3	0.572621285	1
ALCURONIUM CHLORIDE	MCF7	0.573182146	1
TROGLITAZONE	PC3	0.573739144	1
CAPSAICIN	MCF7	0.574304034	1
ISOFLUPREDONE	MCF7	0.574490926	1
PROFENAMINE	PC3	0.574689262	1
ELLIPTICINE	PC3	0.575284311	1
IOXAGLIC ACID	MCF7	0.575426138	1
ARTEMISININ	MCF7	0.575482674	1
TOCAINIDE	PC3	0.576077801	1
MINOCYCLINE	MCF7	0.576404186	1
HYOSCYAMINE	MCF7	0.57654845	1
BOLDINE	MCF7	0.57682906	1
CEFMETAZOLE	PC3	0.576871393	1
IDAZOXAN	MCF7	0.577109682	1
DEPTROPINE	PC3	0.577268226	1
CHICAGO SKY BLUE 6B	MCF7	0.577951622	1
GANCICLOVIR	HL60	0.578260417	1
FLUFENAMIC ACID	HL60	0.578458873	1
MOLSIDOMINE	MCF7	0.57879367	1
ALTRETAMINE	MCF7	0.579635824	1
FLECAINIDE	PC3	0.579916564	1
ARTICAINE	MCF7	0.580245238	1
LEVOBUNOLOL	HL60	0.580443751	1
ETODOLAC	PC3	0.580478079	1
ALEXIDINE	MCF7	0.581237858	1
HARMAN	PC3	0.581833494	1
GLIBENCLAMIDE	HL60	0.582230611	1
AMINOPHYLLINE	PC3	0.583422084	1
ASIATICOSIDE	MCF7	0.583567167	1
METHAZOLAMIDE	PC3	0.584415112	1
PANCURONIUM BROMIDE	PC3	0.584613732	1
THIETHYLPERAZINE	MCF7	0.584971695	1
ETHOTOIN	PC3	0.585252628	1
3-ACETAMIDOCOUMARIN	MCF7	0.585252628	1
SALSOLINOL	MCF7	0.585533571	1
THIORIDAZINE	HL60	0.586048797	1
HARPAGOSIDE	PC3	0.586202853	1
MEPTAZINOL	HL60	0.586401513	1
DICLOFENAC	MCF7	0.5867224	1
TRIFLURIDINE	PC3	0.587394876	1
AZAPERONE	MCF7	0.587500409	1
HEXAMETHONIUM BROMIDE	MCF7	0.587781419	1
PERPHENAZINE	PC3	0.587781419	1
NALIDIXIC ACID	MCF7	0.588062437	1
NABUMETONE	HL60	0.588587045	1
N-ACETYLMURAMIC ACID	MCF7	0.588905538	1
BETAZOLE	HL60	0.589183181	1
CORTICOSTERONE	MCF7	0.589186586	1
LACTOBIONIC ACID	MCF7	0.589186586	1
AMODIAQUINE	PC3	0.589580623	1
ETIDRONIC ACID	HL60	0.58997808	1
BUTOCONAZOLE	PC3	0.590574292	1
PROADIFEN	MCF7	0.590591937	1
IDAZOXAN	PC3	0.591170534	1
ETOFENAMATE	PC3	0.591369288	1
SULFAMETHIZOLE	MCF7	0.59143523	1
ZALCITABINE	PC3	0.591568045	1
(-)-MK-801	MCF7	0.59171634	1
DAPSONE	PC3	0.592559709	1
REMOXIPRIDE	PC3	0.592760653	1

CHLORTETRACYCLINE	HL60	0.593158213	1
AR-A014418	MCF7	0.59435096	1
TANESPIMYCIN	MCF7	0.59436306	1
CHRYSIN	MCF7	0.594527772	1
CLOXACILLIN	PC3	0.59454976	1
DIPROPHYLLINE	PC3	0.595090124	1
TETROQUINONE	MCF7	0.595090124	1
PRESTWICK-1085	HL60	0.595344987	1
RETRORSINE	MCF7	0.595371307	1
LISURIDE	PC3	0.595652495	1
OXAMNIQUINE	PC3	0.595941432	1
L-METHIONINE SULFOXIMINE	HL60	0.596140252	1
MECLOFENAMIC ACID	PC3	0.596214884	1
TRIMETHADIONE	PC3	0.596339074	1
PRIDINOL	PC3	0.596935552	1
PROPAFENONE	MCF7	0.597058501	1
ALPHA-YOHIMBINE	PC3	0.597333215	1
CALCIUM FOLINATE	PC3	0.597339715	1
ALPROSTADIL	MCF7	0.597396363	1
LEVOTHYROXINE SODIUM	HL60	0.597929724	1
CYCLOPENTOLATE	MCF7	0.598464609	1
ROSIGLITAZONE	PC3	0.599118666	1
PILOCARPINE	MCF7	0.599308316	1
CLEBOPRIDE	PC3	0.599321639	1
NIFENAZONE	PC3	0.599870803	1
SISOMICIN	MCF7	0.599870803	1
ACETOHEXAMIDE	PC3	0.600117054	1
SOTALOL	PC3	0.600315912	1
HYDROXYZINE	MCF7	0.600714554	1
PRIMAQUINE	PC3	0.601707948	1
SPARTEINE	MCF7	0.602402121	1
ALPHA-ESTRADIOL	ssMCF7	0.603497781	1
HARMALOL	MCF7	0.603527203	1
TUBOCURARINE CHLORIDE	MCF7	0.603808477	1
BENFLUOREX	MCF7	0.604652305	1
NAFTIFINE	PC3	0.604691034	1
VANOXERINE	MCF7	0.605214861	1
ADIPIODONE	MCF7	0.60662126	1
PINACIDIL	PC3	0.606679817	1
METACYCLINE	PC3	0.607077574	1
OXYBUTYNIN	MCF7	0.6074651	1
BENFLUOREX	HL60	0.607674209	1
HYDRALAZINE	PC3	0.607746379	1
PRAMOCAINE	HL60	0.608469719	1
METOPROLOL	MCF7	0.609152765	1
ARACHIDONYLTRIFLUOROMETHANE	MCF7	0.60943404	1
NAPROXEN	MCF7	0.609514519	1
OXPRENOLOL	MCF7	0.609715313	1
HESPERIDIN	HL60	0.609861844	1
BETAZOLE	PC3	0.609996584	1
ALEXIDINE	PC3	0.611121655	1
XYLOMETAZOLINE	PC3	0.611651665	1
CLIDINIUM BROMIDE	PC3	0.612049392	1
NAFTIFINE	MCF7	0.612246694	1
MEDRYSONE	PC3	0.612527948	1
ETAMSYLATE	PC3	0.613242543	1
(-)-CATECHIN	MCF7	0.613640249	1
CHLORPHENAMINE	MCF7	0.614496645	1
ETACRYNIC ACID	PC3	0.614634485	1
DIPYRIDAMOLE	PC3	0.614777875	1
ROLIPRAM	PC3	0.615032168	1

IPRONIAZID	MCF7	0.616183968	1
FLECAINIDE	HL60	0.616622822	1
(+)-CHELIDONINE	HL60	0.617020464	1
QUINOSTATIN	MCF7	0.617308767	1
INDAPAMIDE	MCF7	0.617721357	1
IMIPRAMINE	PC3	0.617815722	1
BETAZOLE	MCF7	0.618152318	1
FURALTADONE	HL60	0.618412139	1
CYANOCOBALAMIN	PC3	0.618809738	1
ALSTERPAULLONE	MCF7	0.619207326	1
NOMIFENSINE	PC3	0.619558133	1
SUPROFEN	PC3	0.619803687	1
LANSOPRAZOLE	MCF7	0.620120421	1
MONOBENZONE	PC3	0.621195094	1
GLIQUIDONE	HL60	0.621393854	1
ESTRADIOL	HL60	0.621516231	1
CHENODEOXYCHOLIC ACID	MCF7	0.621807137	1
KETOROLAC	PC3	0.621990114	1
LEVOTHYROXINE SODIUM	PC3	0.622387605	1
METHACHOLINE CHLORIDE	MCF7	0.622650407	1
ISRADIPINE	MCF7	0.622931483	1
BETULIN	MCF7	0.622931483	1
PENTAMIDINE	PC3	0.623182543	1
TRIMETHOPRIM	MCF7	0.623212552	1
SULFAMETOXYDIAZINE	MCF7	0.623493614	1
DIETHYLSTILBESTROL	MCF7	0.623575031	1
DIFLORASONE	MCF7	0.623774668	1
DENATONIUM BENZOATE	PC3	0.623778709	1
BACAMPICILLIN	MCF7	0.624336754	1
QUINETHAZONE	PC3	0.624336754	1
PRESTWICK-972	PC3	0.624573544	1
PAROXETINE	MCF7	0.624617786	1
DICLOFENAMIDE	PC3	0.625169629	1
GENTAMICIN	PC3	0.625460832	1
COLISTIN	MCF7	0.625741831	1
CLOPAMIDE	MCF7	0.626022822	1
HARPAGOSIDE	MCF7	0.626303804	1
PROPANTHELINE BROMIDE	PC3	0.627354952	1
0179445-0000	MCF7	0.627409714	1
THALIDOMIDE	PC3	0.627708583	1
KINETIN	MCF7	0.627989511	1
BENZBROMARONE	HL60	0.628546718	1
GEMFIBROZIL	MCF7	0.628832239	1
THIAMPHENICOL	PC3	0.62939401	1
OXPRENOLOL	PC3	0.629539724	1
CEFORANIDE	MCF7	0.62967488	1
PIRLINDOLE	HL60	0.630135468	1
FOLIC ACID	HL60	0.630334039	1
MILRINONE	MCF7	0.630798258	1
APRAMYCIN	PC3	0.63112827	1
AMPYRONE	PC3	0.631359884	1
ALVERINE	PC3	0.63164068	1
COTININE	PC3	0.631921466	1
FENOTEROL	HL60	0.632319453	1
PYRVINIUM	MCF7	0.632573959	1
METERGOLINE	PC3	0.633311951	1
CLORGILINE	PC3	0.633510433	1
MOLINDONE	MCF7	0.633605941	1
PAROMOMYCIN	PC3	0.633708909	1
DINOPROSTONE	MCF7	0.633886646	1
PROCAINAMIDE	PC3	0.634105844	1

CLORSULON	PC3	0.634304302	1
GALANTAMINE	MCF7	0.635289987	1
APRAMYCIN	MCF7	0.635570618	1
SANGUINARINE	MCF7	0.635891743	1
HNMPA-(AM)3	PC3	0.636090145	1
ISOCARBOXAZID	MCF7	0.636693012	1
CONVOLAMINE	MCF7	0.636973578	1
TROGLITAZONE	SKMEL5	0.637082054	1
FLUTAMIDE	MCF7	0.63725413	1
MITOXANTRONE	PC3	0.637478771	1
LORGLUMIDE	PC3	0.637677119	1
VINPOCETINE	MCF7	0.637815193	1
HEMICHOLINIUM	MCF7	0.637815193	1
CORBADRINE	MCF7	0.638095705	1
QUIPAZINE	PC3	0.638470443	1
SULFAMERAZINE	MCF7	0.639051503	1
NOVOBIOCIN	HL60	0.639065362	1
BETONICINE	PC3	0.639217609	1
BUFLOMEDIL	MCF7	0.640619663	1
CROMOGLICIC ACID	MCF7	0.640651493	1
MECAMYLAMINE	MCF7	0.641180381	1
TROPINE	MCF7	0.641741037	1
CHLORPROPAMIDE	PC3	0.642237142	1
CP-944629	MCF7	0.64230163	1
PHENOXYBENZAMINE	PC3	0.642435313	1
CLONIDINE	MCF7	0.643702835	1
DELSOLINE	PC3	0.643822285	1
FENBENDAZOLE	PC3	0.644263203	1
FELBINAC	HL60	0.644812733	1
CYPROHEPTADINE	PC3	0.644823504	1
CROTAMITON	HL60	0.645604938	1
PREDNISONE	HL60	0.646000989	1
ALFADOLONE	PC3	0.646199001	1
ESTRIOL	MCF7	0.646223956	1
VINCAMINE	PC3	0.646223956	1
ALVERINE	HL60	0.646397005	1
PIZOTIFEN	MCF7	0.646784014	1
EMETINE	HL60	0.646990962	1
VINBLASTINE	MCF7	0.647064017	1
N-ACETYL-L-ASPARTIC ACID	PC3	0.647386889	1
ALCLOMETASONE	MCF7	0.647903916	1
TETROQUINONE	HL60	0.648178633	1
LITHOCHOLIC ACID	MCF7	0.648432079	1
SULFATHIAZOLE	MCF7	0.648463757	1
TOLMETIN	PC3	0.64857445	1
PHENANTHRIDINONE	MCF7	0.64897023	1
R-ATENOLOL	PC3	0.649168105	1
SCOULERINE	MCF7	0.64986303	1
LUMICOLCHICINE	PC3	0.650552966	1
CLOPAMIDE	PC3	0.651146332	1
FOLIOSIDINE	PC3	0.651261822	1
0173570-0000	MCF7	0.651541521	1
ISOCORYDINE	MCF7	0.6518212	1
FLUNISOLIDE	MCF7	0.651859033	1
DISOPYRAMIDE	HL60	0.652925887	1
RIBAVIRIN	MCF7	0.652939712	1
PHENACETIN	PC3	0.653321231	1
SPIRADOLINE	PC3	0.653498845	1
TENOXICAM	PC3	0.653518888	1
ADIPHENINE	MCF7	0.65377838	1
MOXONIDINE	PC3	0.654111794	1

AMINOCAPROIC ACID	MCF7	0.654309408	1
PHA-00745360	PC3	0.654370496	1
MIFEPRISTONE	MCF7	0.654616857	1
NAPHAZOLINE	PC3	0.655175735	1
MAFENIDE	PC3	0.656013888	1
CEFSULODIN	PC3	0.65608745	1
PRESTWICK-692	MCF7	0.656572547	1
QUINIDINE	PC3	0.657272315	1
METHOXAMINE	MCF7	0.657410366	1
OXEDRINE	MCF7	0.657689594	1
PREDNICARBATE	MCF7	0.657968799	1
TERFENADINE	MCF7	0.658247981	1
ALTRETAMINE	PC3	0.658259391	1
XYLAZINE	PC3	0.659838107	1
METHOCARBAMOL	MCF7	0.660201598	1
LUTEOLIN	PC3	0.660232667	1
IDOXURIDINE	PC3	0.660759559	1
PREGNENOLONE	HL60	0.660824417	1
TRIMIPRAMINE	PC3	0.661021643	1
FLUOROCURARINE	MCF7	0.661038504	1
TILETAMINE	HL60	0.661416058	1
CHLORPROMAZINE	PC3	0.661873219	1
MIDECAMYCIN	MCF7	0.662990421	1
FINASTERIDE	MCF7	0.663145176	1
PROXYPHYLLINE	MCF7	0.663269166	1
S-PROPRANOLOL	PC3	0.663387385	1
PHENINDIONE	HL60	0.663584448	1
DIZOCILPINE	PC3	0.664105247	1
DIPHENYLPYRALINE	MCF7	0.664738039	1
MOROXYDINE	HL60	0.664963529	1
EDROPHONIUM CHLORIDE	HL60	0.665357434	1
NAFTIDROFURYL	MCF7	0.665498196	1
SULOCTIDIL	HL60	0.665948193	1
GUANADREL	PC3	0.666055191	1
ANTIMYCIN A	MCF7	0.666333648	1
CYCLOSERINE	PC3	0.666735683	1
ZARDAVERINE	MCF7	0.667168857	1
ETIOCHOLANOLONE	HL60	0.667326159	1
APIGENIN	PC3	0.667719741	1
DILTIAZEM	MCF7	0.667725526	1
ALCLOMETASONE	PC3	0.667916511	1
TICLOPIDINE	PC3	0.66800382	1
PHENYLPROPANOLAMINE	PC3	0.668506739	1
IOBENGUANE	MCF7	0.668560323	1
DIHYDROERGOTAMINE	MCF7	0.669116715	1
CALYCANTHINE	PC3	0.669293513	1
PRESTWICK-685	MCF7	0.669394868	1
NAPELLINE	MCF7	0.669951089	1
XAMOTEROL	MCF7	0.670785206	1
GALLAMINE TRIETHIODIDE	PC3	0.671063187	1
OXYBUTYNIN	PC3	0.67125945	1
CARBIMAZOLE	MCF7	0.671619062	1
N-PHENYLANTHRANILIC ACID	MCF7	0.671848948	1
HYDRASTININE	PC3	0.672174818	1
BROMOPRIDE	MCF7	0.67223531	1
MIANSERIN	HL60	0.672634737	1
NORTRIPTYLINE	MCF7	0.672730456	1
CLENBUTEROL	MCF7	0.672915848	1
Y-27632	MCF7	0.673285974	1
TOBRAMYCIN	MCF7	0.673563688	1
PANCURONIUM BROMIDE	MCF7	0.673841371	1

DECITABINE	MCF7	0.674009292	1
GLICLAZIDE	PC3	0.674598158	1
PHYSOSTIGMINE	MCF7	0.675229327	1
PRILOCAINE	MCF7	0.675296297	1
METAMPICILLIN	MCF7	0.675506825	1
ETHOSUXIMIDE	PC3	0.676616504	1
OXYTETRACYCLINE	PC3	0.677148292	1
ETOFYLLINE	MCF7	0.678280065	1
BECLOMETASONE	MCF7	0.678520325	1
PRESTWICK-642	PC3	0.678716264	1
FLUMETASONE	MCF7	0.678806078	1
VALPROIC ACID	PC3	0.678992078	1
MEXILETINE	MCF7	0.679032338	1
CLEMIZOLE	PC3	0.679111407	1
MIANSERIN	MCF7	0.679388455	1
ADENOSINE PHOSPHATE	PC3	0.679499858	1
RALOXIFENE	ssMCF7	0.68008738	1
DIHYDROERGOTAMINE	HL60	0.680478977	1
ZIMELDINE	PC3	0.680496315	1
BUPROPION	HL60	0.680870507	1
ACEMETACIN	HL60	0.681066246	1
TRANEXAMIC ACID	PC3	0.681326856	1
HARMOL	MCF7	0.681603635	1
TERCONAZOLE	PC3	0.681849034	1
RIBOFLAVIN	MCF7	0.68188038	1
QUINISOCAINE	MCF7	0.682157091	1
ACECLOFENAC	MCF7	0.682987016	1
AMPHOTERICIN B	PC3	0.683413784	1
TONZONIUM BROMIDE	PC3	0.684195739	1
PROPYLTHIOURACIL	MCF7	0.684369524	1
ZIDOVUDINE	HL60	0.68478202	1
BCB000039	PC3	0.685198603	1
PRESTWICK-857	PC3	0.685368139	1
ADRENOSTERONE	PC3	0.685563477	1
MEPRYLCAINE	HL60	0.68614938	1
CLEMASTINE	HL60	0.686539891	1
3-ACETAMIDOCOUMARIN	PC3	0.687125521	1
PROBUCOL	PC3	0.687320694	1
FLUMEQUINE	MCF7	0.68740789	1
TRIMETHOBENZAMIDE	MCF7	0.687959845	1
HYDROCORTISONE	MCF7	0.688235766	1
HYDROFLUMETHIAZIDE	MCF7	0.68851165	1
ENILCONAZOLE	HL60	0.6892714	1
ABAMECTIN	MCF7	0.689890504	1
SPECTINOMYCIN	MCF7	0.690441779	1
PF-00539758-00	PC3	0.691268402	1
PRESTWICK-981	MCF7	0.691543866	1
HALOPERIDOL	MCF7	0.691569378	1
CINCHOCAINE	HL60	0.691609742	1
AR-A014418	PC3	0.69181929	1
ALIMEMAZINE	MCF7	0.692094676	1
COLFORSIN	PC3	0.692370022	1
IKARUGAMYCIN	MCF7	0.692565063	1
PENTETRAZOL	PC3	0.69316708	1
MEFLOQUINE	MCF7	0.693195823	1
DISULFIRAM	HL60	0.693361658	1
PENTETIC ACID	PC3	0.694139771	1
CEFALOTIN	PC3	0.694334248	1
ERASTIN	MCF7	0.69457136	1
VIGABATRIN	PC3	0.694723144	1
NATAMYCIN	PC3	0.695306334	1

CHLORHEXIDINE	PC3	0.695671062	1
CINCHONIDINE	PC3	0.695889341	1
DINOPROSTONE	PC3	0.695945885	1
LOBELINE	PC3	0.69627791	1
BENZONATATE	MCF7	0.696495408	1
SULFACETAMIDE	PC3	0.696770107	1
PINDOLOL	MCF7	0.696770107	1
PROGLUMIDE	MCF7	0.697044765	1
PIRIBEDIL	MCF7	0.697593955	1
PRESTWICK-665	PC3	0.698142978	1
VITEXIN	PC3	0.698413549	1
S-PROPRANOLOL	MCF7	0.698417425	1
OLEANDOMYCIN	MCF7	0.698966194	1
METHOXAMINE	PC3	0.699189513	1
LIOTHYRONINE	MCF7	0.699240514	1
CYTOCHALASIN B	MCF7	0.699577367	1
OCTOPAMINE	HL60	0.700158987	1
LANATOSIDE C	MCF7	0.700304437	1
SULFABENZAMIDE	PC3	0.700352817	1
TRIFLURIDINE	MCF7	0.701159551	1
HYDRASTININE	HL60	0.701321642	1
JOSAMYCIN	PC3	0.701707456	1
PRESTWICK-559	MCF7	0.702528983	1
OXYBUTYNIN	HL60	0.702677075	1
HEPTAMINOL	PC3	0.702802736	1
BROMOCRIPTINE	HL60	0.70287062	1
CLIOQUINOL	HL60	0.703064142	1
LEVOPROPOXYPHENE	MCF7	0.703076445	1
MAPROTILINE	MCF7	0.703623729	1
FAMOTIDINE	HL60	0.703838008	1
MELATONIN	MCF7	0.703897304	1
GLIPIZIDE	PC3	0.704444318	1
DOSULEPIN	PC3	0.704611514	1
NITRENDIPINE	PC3	0.704998131	1
DIETHYLSTILBESTROL	HL60	0.705191406	1
SULFAPYRIDINE	PC3	0.705771091	1
MYCOPHENOLIC ACID	MCF7	0.706357432	1
PHENAZOPYRIDINE	MCF7	0.70663055	1
PROGESTERONE	PC3	0.70692984	1
METARAMINOL	MCF7	0.707722555	1
GLIQUIDONE	PC3	0.707894824	1
4-HYDROXYPHENAZONE	MCF7	0.70799544	1
CARMUSTINE	MCF7	0.70881381	1
SPIRADOLINE	MCF7	0.709086505	1
NOMIFENSINE	HL60	0.709630309	1
RONIDAZOLE	MCF7	0.710401015	1
METOCLOPRAMIDE	MCF7	0.71134796	1
CHLORZOXAZONE	PC3	0.711748819	1
BW-B70C	MCF7	0.712133686	1
PIOGLITAZONE	PC3	0.712339898	1
GLIMEPIRIDE	MCF7	0.712355091	1
ASCORBIC ACID	MCF7	0.712899172	1
DROPROPIZINE	PC3	0.712903126	1
TALAMPICILLIN	PC3	0.713287697	1
NICOTINIC ACID	MCF7	0.713443054	1
ISOCARBOXAZID	HL60	0.71405654	1
SULFANILAMIDE	PC3	0.71463291	1
ARTEMISININ	PC3	0.71501703	1
METHYLDOPATE	MCF7	0.715616581	1
SULFAMETHOXYPYRIDAZINE	PC3	0.715784967	1
BACAMPICILLIN	HL60	0.715976888	1

SULFADOXINE	PC3	0.716168783	1
METRONIDAZOLE	PC3	0.716702127	1
CEFAZOLIN	PC3	0.716973385	1
KINETIN	PC3	0.717319615	1
SR-95531	MCF7	0.717515747	1
IPRATROPIUM BROMIDE	PC3	0.717703019	1
PIVMECILLINAM	MCF7	0.71778685	1
ONDANSETRON	PC3	0.717894682	1
OXYBUPROCAINE	MCF7	0.718057901	1
EUCATROPINE	MCF7	0.718224095	1
SULOCTIDIL	MCF7	0.719412371	1
TOLNAFTATE	MCF7	0.719683107	1
BCB000038	MCF7	0.719683107	1
NOVOBIOCIN	MCF7	0.720650342	1
TRAPIDIL	MCF7	0.720765521	1
APOMORPHINE	PC3	0.721306408	1
CHLORPHENAMINE	PC3	0.722295653	1
ETHOTOIN	MCF7	0.722311793	1
ARECOLINE	HL60	0.722677676	1
ACICLOVIR	PC3	0.723197809	1
TORASEMIDE	PC3	0.724395428	1
DOXEPIN	PC3	0.724776847	1
NIPECOTIC ACID	PC3	0.725348768	1
TRIHEXYPHENIDYL	HL60	0.72572991	1
ACETAZOLAMIDE	MCF7	0.725895149	1
SALBUTAMOL	PC3	0.725895149	1
RESCINNAMINE	MCF7	0.726164576	1
DEHYDROCHOLIC ACID	PC3	0.726433948	1
FUSARIC ACID	PC3	0.727443654	1
NEOMYCIN	HL60	0.728965049	1
MEPYRAMINE	MCF7	0.729124539	1
ETIOCHOLANOLONE	MCF7	0.729133445	1
PINDOLOL	HL60	0.729345111	1
DIFLUNISAL	MCF7	0.730199167	1
SULFAPHENAZOLE	MCF7	0.730199167	1
SECURININE	MCF7	0.731004526	1
FUROSEMIDE	PC3	0.731053951	1
MESALAZINE	MCF7	0.731107093	1
TRIMETHADIONE	MCF7	0.731541139	1
PRONETALOL	PC3	0.731623038	1
TETRACYCLINE	MCF7	0.731809357	1
METHYLDOPATE	PC3	0.732002282	1
ARTEMISININ	HL60	0.732191859	1
DIRITHROMYCIN	HL60	0.732381407	1
CARBENOXOLONE	MCF7	0.732613657	1
CYCLOPENTHIAZIDE	PC3	0.733139301	1
HYDROCOTARNINE	PC3	0.733707408	1
CONESSINE	MCF7	0.734220644	1
HOMATROPINE	MCF7	0.734488265	1
HECOGENIN	PC3	0.734653652	1
PENTOXIFYLLINE	MCF7	0.735023325	1
GLYCOCHOLIC ACID	HL60	0.735221035	1
GENISTEIN	HL60	0.735372374	1
6-BROMOINDIRUBIN-3'-OXIME	PC3	0.735487985	1
EPIRIZOLE	PC3	0.73582546	1
SULFAMONOMETHOXINE	PC3	0.73597712	1
FUSIDIC ACID	MCF7	0.736092716	1
GENTAMICIN	MCF7	0.73635991	1
CICLOSPORIN	MCF7	0.736621948	1
AZLOCILLIN	MCF7	0.736627044	1
NALTREXONE	MCF7	0.737428074	1

BENZOCAINE	PC3	0.738430997	1
CEFSULODIN	HL60	0.738619539	1
MK-886	MCF7	0.738761882	1
TETRYZOLINE	MCF7	0.739561416	1
ACETYLSALICYLSALICYLIC ACID	MCF7	0.739827801	1
PEMPIDINE	MCF7	0.740165911	1
MORANTEL	MCF7	0.740360382	1
PHYSOSTIGMINE	PC3	0.741067725	1
METHAPYRILENE	MCF7	0.741158777	1
DILOXANIDE	HL60	0.741255825	1
TROPICAMIDE	MCF7	0.74136113	1
TACRINE	MCF7	0.741424781	1
CLOBETASOL	MCF7	0.741690721	1
PIRETANIDE	MCF7	0.741956596	1
WITHAFERIN A	PC3	0.742222408	1
SULFANILAMIDE	MCF7	0.742488155	1
KETOPROFEN	PC3	0.742488155	1
TOLNAFTATE	PC3	0.743285008	1
VINBURNINE	MCF7	0.744081275	1
AMILORIDE	MCF7	0.744346567	1
VANCOMYCIN	HL60	0.744823593	1
SYROSINGOPINE	MCF7	0.745407078	1
DEPUDECIN	MCF7	0.745936938	1
CEFEPIME	PC3	0.746135057	1
ALPROSTADIL	HL60	0.746509463	1
EPIRIZOLE	MCF7	0.746995859	1
(-)-MK-801	PC3	0.747257875	1
TRACAZOLATE	MCF7	0.747260422	1
LEVOBUNOLOL	MCF7	0.747260422	1
PIRENZEPINE	PC3	0.747524918	1
DAUNORUBICIN	MCF7	0.748181225	1
SULFATHIAZOLE	PC3	0.748846388	1
SAQUINAVIR	PC3	0.748939834	1
HOMOSALATE	MCF7	0.749110479	1
PINDOLOL	PC3	0.749638457	1
MONENSIN	MCF7	0.749690815	1
GALLAMINE TRIETHIODIDE	MCF7	0.750166161	1
DL-THIORPHAN	MCF7	0.751220746	1
PROTRIPTYLINE	PC3	0.751736969	1
SOLASODINE	MCF7	0.751951006	1
CLINDAMYCIN	MCF7	0.752274224	1
PIRINIXIC ACID	SKMEL5	0.752295464	1
NITROFURANTOIN	MCF7	0.753063602	1
BCB000040	MCF7	0.753063602	1
TETRAMISOLE	MCF7	0.754377828	1
NALBUPHINE	MCF7	0.754903024	1
COLCHICINE	HL60	0.755083211	1
CHENODEOXYCHOLIC ACID	PC3	0.755825266	1
EXEMESTANE	MCF7	0.756381431	1
OFLOXACIN	MCF7	0.756476902	1
PROCHLORPERAZINE	PC3	0.756562977	1
16-PHENYLTETRANORPROSTAGLANDIN E2	PC3	0.756738964	1
THIOCOLCHICOSIDE	MCF7	0.757262872	1
NAFTOPIDIL	PC3	0.757492791	1
THIETHYLPERAZINE	PC3	0.757677891	1
DELSOLINE	MCF7	0.757786491	1
HYDROXYACHILLIN	MCF7	0.758048192	1
IOPAMIDOL	PC3	0.758232974	1
0317956-0000	PC3	0.758684661	1
ZIMELDINE	MCF7	0.759094266	1
ALFUZOSIN	MCF7	0.759127508	1

VINCAMINE	HL60	0.759157384	1
MONORDEN	PC3	0.759636531	1
CHLORZOXAZONE	MCF7	0.760139168	1
PROPRANOLOL	PC3	0.760265466	1
NITRENDIPINE	MCF7	0.760300575	1
BEZAFIBRATE	MCF7	0.76118289	1
PRESTWICK-984	PC3	0.761740839	1
LAUDANOSINE	PC3	0.761925093	1
TENOXICAM	MCF7	0.7619649	1
ORNIDAZOLE	PC3	0.762225421	1
MEPHENTERMINE	MCF7	0.762746238	1
LOBELANIDINE	HL60	0.762845802	1
VERAPAMIL	PC3	0.763006534	1
ZOXAZOLAMINE	MCF7	0.763266754	1
NORTRIPTYLINE	PC3	0.763581693	1
LORGLUMIDE	MCF7	0.763704613	1
AMINOCAPROIC ACID	PC3	0.765418766	1
CEFAPIRIN	MCF7	0.767160927	1
PAPAVERINE	MCF7	0.767678842	1
BENPERIDOL	PC3	0.76816718	1
NORDIHYDROGUAIARETIC ACID	ssMCF7	0.768196446	1
PHA-00767505E	MCF7	0.768713738	1
KAWAIN	PC3	0.769489088	1
BENZYDAMINE	PC3	0.769994589	1
NALTREXONE	PC3	0.770005594	1
TROPINE	HL60	0.770359598	1
BROMOCRIPTINE	PC3	0.770521784	1
PRESTWICK-685	PC3	0.770521784	1
FLUVOXAMINE	MCF7	0.77077976	1
W-13	MCF7	0.771295473	1
THEOPHYLLINE	MCF7	0.771810868	1
GUANFACINE	HL60	0.772364306	1
HYDRASTINE HYDROCHLORIDE	PC3	0.772546312	1
ESTRADIOL	PC3	0.773673874	1
MONORDEN	MCF7	0.77375477	1
SEROTONIN	PC3	0.773819228	1
GIBBERELLIC ACID	PC3	0.774000911	1
GOSSYPOL	MCF7	0.774152679	1
TANESPIMYCIN	ssMCF7	0.774182554	1
CLOFILIUM TOSYLATE	PC3	0.774364156	1
SULFACHLORPYRIDAZINE	PC3	0.77438302	1
TRIFLUOPERAZINE	PC3	0.774572094	1
QUINPIROLE	MCF7	0.775305547	1
SKIMMIANINE	PC3	0.77563423	1
SCRIPTAID	MCF7	0.775815505	1
CARBARSONE	MCF7	0.775922414	1
FELODIPINE	PC3	0.775996739	1
CITIOLONE	PC3	0.776178693	1
PYRIMETHAMINE	MCF7	0.776434889	1
MONENSIN	PC3	0.776691003	1
CEFOXITIN	MCF7	0.777202983	1
INDOMETACIN	SKMEL5	0.777264226	1
MAPROTILINE	PC3	0.77744513	1
PHENIRAMINE	MCF7	0.777714631	1
HELVETICOSIDE	MCF7	0.778128287	1
BACLOFEN	PC3	0.778225947	1
PHENYLPROPANOLAMINE	MCF7	0.778225947	1
CLIOQUINOL	MCF7	0.778232639	1
OXYPHENBUTAZONE	PC3	0.77925188	1
FENDILINE	MCF7	0.779502773	1
FLUPENTIXOL	PC3	0.779793088	1

XYLAZINE	MCF7	0.78179575	1
ALSTERPAULLONE	PC3	0.7820501	1
SULFAFURAZOLE	PC3	0.782313693	1
METFORMIN	MCF7	0.78280216	1
IOVERSOL	PC3	0.78303233	1
LYNESTRENOL	PC3	0.783320566	1
GLIQUIDONE	MCF7	0.783574401	1
SKIMMIANINE	MCF7	0.78382815	1
TOPIRAMATE	MCF7	0.784288288	1
SULFADIMIDINE	PC3	0.784335388	1
ALIMEMAZINE	PC3	0.784646743	1
PHENAZOPYRIDINE	PC3	0.785184101	1
PROMETHAZINE	MCF7	0.785601963	1
TOLAZOLINE	PC3	0.785855016	1
RANITIDINE	MCF7	0.78661365	1
TETRAETHYLENEPENTAMINE	HL60	0.786972452	1
FLUOROMETHOLONE	MCF7	0.787118967	1
CINCHOCAINE	PC3	0.787876279	1
CLOTRIMAZOLE	PC3	0.78804335	1
CICLOHEXIMIDE	PC3	0.788221678	1
NAPROXEN	PC3	0.79013803	1
DANAZOL	MCF7	0.790143415	1
TETRAHYDROALSTONINE	HL60	0.790535884	1
SC-19220	MCF7	0.790646238	1
QUIPAZINE	MCF7	0.790897514	1
SULFABENZAMIDE	MCF7	0.7911487	1
OMEPRAZOLE	MCF7	0.791901715	1
ISOXICAM	PC3	0.792653912	1
CARTEOLOL	PC3	0.793196656	1
LOXAPINE	PC3	0.794434836	1
HYCANTHONE	MCF7	0.794655747	1
CLORSULON	MCF7	0.794905562	1
DESIPRAMINE	MCF7	0.795155285	1
KAWAIN	MCF7	0.795404915	1
SPARTEINE	PC3	0.795494336	1
PUROMYCIN	PC3	0.795670757	1
AMOXAPINE	PC3	0.796153248	1
SUCCINYLSULFATHIAZOLE	PC3	0.796375977	1
OXETACAINE	PC3	0.796402506	1
CIMETIDINE	MCF7	0.797149721	1
WORTMANNIN	HL60	0.79734358	1
GLIMEPIRIDE	PC3	0.797432407	1
PROMAZINE	PC3	0.797896092	1
NATAMYCIN	MCF7	0.798144694	1
C-75	MCF7	0.798641616	1
GLYCOCHOLIC ACID	MCF7	0.798889935	1
URSODEOXYCHOLIC ACID	MCF7	0.799013881	1
GENISTEIN	MCF7	0.799876952	1
CELASTROL	MCF7	0.800766574	1
CLOPERASTINE	MCF7	0.800936542	1
PRENYLAMINE	HL60	0.800941581	1
CIPROFIBRATE	HL60	0.801291452	1
DIGOXIGENIN	MCF7	0.801542518	1
DICLOXACILLIN	MCF7	0.801615169	1
2,6-DIMETHYLPIPERIDINE	MCF7	0.801643457	1
IOHEXOL	HL60	0.801815897	1
BUTAMBEN	HL60	0.801990616	1
PRIDINOL	MCF7	0.802109421	1
ALLANTOIN	MCF7	0.802356403	1
TOLBUTAMIDE	PC3	0.802603288	1
VINBURNINE	HL60	0.802863487	1

CYCLOPENTHIAZIDE	MCF7	0.803096767	1
2-AMINOBENZENESULFONAMIDE	PC3	0.80338663	1
2-AMINOBENZENESULFONAMIDE	MCF7	0.803589859	1
CEFAMANDOLE	PC3	0.804082562	1
DESIPRAMINE	HL60	0.804083473	1
METHYLDOPA	MCF7	0.804563704	1
HECOGENIN	MCF7	0.804820885	1
BENFOTIAMINE	HL60	0.805127274	1
SCOPOLAMINE N-OXIDE	PC3	0.805312611	1
PIPERACETAZINE	PC3	0.805822161	1
SURAMIN SODIUM	MCF7	0.806049462	1
CEFSULODIN	MCF7	0.806294883	1
PRESTWICK-692	HL60	0.80651626	1
CEFOTIAM	MCF7	0.806785427	1
ZOXAZOLAMINE	HL60	0.807209569	1
ACENOCOUMAROL	MCF7	0.807275575	1
THIOCOLCHICOSIDE	HL60	0.807555926	1
SPLITOMICIN	PC3	0.80772903	1
RAMIPRIL	MCF7	0.808254679	1
TRIFLUPROMAZINE	MCF7	0.808499206	1
BUSPIRONE	MCF7	0.809232186	1
BROMOCRIPTINE	MCF7	0.809476312	1
CLOFAZIMINE	MCF7	0.809566293	1
DANAZOL	PC3	0.809629879	1
HEPTAMINOL	HL60	0.810147239	1
DACARBAZINE	MCF7	0.810208087	1
BEZAFIBRATE	PC3	0.810491894	1
LOMUSTINE	MCF7	0.810695432	1
DICYCLOVERINE	PC3	0.811182373	1
GELDANAMYCIN	MCF7	0.811429331	1
PRIMIDONE	PC3	0.812040333	1
SPIRAMYCIN	MCF7	0.812051947	1
FELODIPINE	MCF7	0.812358353	1
PAROMOMYCIN	HL60	0.812383871	1
CEFOTIAM	PC3	0.813584646	1
CHLOROPYRAZINE	MCF7	0.813853266	1
CLOZAPINE	HL60	0.814358538	1
DIFLUNISAL	PC3	0.815063202	1
PROPOFOL	HL60	0.815295666	1
HYDROQUININE	MCF7	0.815304879	1
BENDROFLUMETHIAZIDE	MCF7	0.815316726	1
HEXETIDINE	MCF7	0.815546452	1
DEXVERAPAMIL	MCF7	0.816490297	1
LIDOCAINE	PC3	0.816511705	1
SAQUINAVIR	MCF7	0.816752757	1
0225151-0000	MCF7	0.8170015	1
DIHYDROERGOTAMINE	PC3	0.817234548	1
TOLFENAMIC ACID	PC3	0.817234548	1
EBSELEN	MCF7	0.81734204	1
EPIVINCAMINE	MCF7	0.817475287	1
SR-95639A	MCF7	0.81771592	1
DIETHYLSTILBESTROL	PC3	0.81771592	1
MEFEXAMIDE	HL60	0.818362402	1
TOMATIDINE	MCF7	0.81843719	1
AZAPROPAZONE	MCF7	0.818532278	1
KARAKOLINE	MCF7	0.818856814	1
GUANABENZ	MCF7	0.818917509	1
CARBAMAZEPINE	PC3	0.819637194	1
FELBINAC	MCF7	0.821312738	1
AMOXAPINE	MCF7	0.821551674	1
NORETHISTERONE	MCF7	0.821790503	1

PHENSUXIMIDE	PC3	0.821918655	1
ONDANSETRON	MCF7	0.822267838	1
SENECIPHYLLINE	MCF7	0.822744743	1
ACETYLSALICYLSALICYLIC ACID	PC3	0.822761908	1
ISOCONAZOLE	MCF7	0.822983034	1
TYRPHOSTIN AG-825	MCF7	0.823435539	1
ISOSORBIDE	PC3	0.823603812	1
BACLOFEN	MCF7	0.823935115	1
TALAMPICILLIN	MCF7	0.823935115	1
TRAPIDIL	HL60	0.82427636	1
PROGLUMIDE	PC3	0.824410505	1
RITODRINE	PC3	0.824444361	1
THIORIDAZINE	PC3	0.824516425	1
SULINDAC	MCF7	0.824534054	1
DIETHYLCARBAMAZINE	PC3	0.825115821	1
LEVOMEPROMAZINE	MCF7	0.826070933	1
PROPOXYCAINE	MCF7	0.826307699	1
ETOPOSIDE	HL60	0.826623407	1
GRAMINE	MCF7	0.826780902	1
ROTTLERIN	MCF7	0.827027465	1
NAPHAZOLINE	MCF7	0.827253663	1
DINOPROST	HL60	0.827459032	1
ZOMEPIRAC	MCF7	0.827725983	1
TRIOXYSALEN	PC3	0.828126536	1
ELLIPTICINE	MCF7	0.828197861	1
PRENYLAMINE	MCF7	0.828669294	1
PIMOZIDE	MCF7	0.828904844	1
METOLAZONE	PC3	0.829845928	1
LOVASTATIN	PC3	0.829957591	1
ATRACURIUM BESILATE	MCF7	0.83008092	1
PROTRIPTYLINE	MCF7	0.830785222	1
HARMAN	HL60	0.831119285	1
6-AZATHYMINE	MCF7	0.831254195	1
ETHAVERINE	PC3	0.83161631	1
METITEPINE	MCF7	0.831722718	1
MYOSMINE	PC3	0.832892044	1
BENSERAZIDE	SKMEL5	0.832939224	1
QUIPAZINE	HL60	0.833104333	1
PRACTOLOL	MCF7	0.833592278	1
MEVALOLACTONE	MCF7	0.833599319	1
TOLFENAMIC ACID	MCF7	0.833764201	1
HYDROFLUMETHIAZIDE	PC3	0.833825461	1
CYCLOBENZAPRINE	MCF7	0.83405853	1
ARCAINE	PC3	0.834258502	1
ADIPHENINE	PC3	0.834291485	1
MECLOFENOXATE	MCF7	0.834783498	1
DOCOSAHEXAENOIC ACID ETHYL ESTER	PC3	0.835081194	1
NEOSTIGMINE BROMIDE	MCF7	0.835222159	1
TONZONIUM BROMIDE	MCF7	0.835222159	1
ISOTRETINOIN	HL60	0.835409869	1
IMIDUREA	PC3	0.836558418	1
CHLORTETRACYCLINE	PC3	0.83661472	1
CHLOROGENIC ACID	PC3	0.836722265	1
DOBUTAMINE	MCF7	0.836846409	1
DOXAZOSIN	MCF7	0.837077982	1
(+)-CHELIDONINE	PC3	0.837540633	1
RANITIDINE	PC3	0.837540781	1
MEFEXAMIDE	MCF7	0.837772005	1
CHLOROGENIC ACID	MCF7	0.83869574	1
LOBELANIDINE	MCF7	0.839617603	1
IRINOTECAN	PC3	0.839661574	1

HARMALINE	MCF7	0.839847776	1
OCTOPAMINE	PC3	0.839986995	1
TILETAMINE	PC3	0.84031218	1
AMINOPHENAZONE	PC3	0.840537588	1
OLEANDOMYCIN	HL60	0.840961843	1
CLINDAMYCIN	PC3	0.841226338	1
FLUNARIZINE	MCF7	0.841226338	1
H-7	MCF7	0.842143014	1
CP-645525-01	PC3	0.842420123	1
SPIPERONE	HL60	0.842743531	1
AMINOPHYLLINE	HL60	0.842905146	1
11-DEOXY-16,16-DIMETHYLPROSTAGLANDIN E2	MCF7	0.843057787	1
(+)-ISOPRENALINE	HL60	0.843873582	1
HARMALOL	PC3	0.844195915	1
SULFACHLORPYRIDAZINE	MCF7	0.844198567	1
SULFAMETHIZOLE	PC3	0.844518008	1
ORNIDAZOLE	HL60	0.844839861	1
PREDNISOLONE	PC3	0.845322188	1
DROFENINE	PC3	0.845482843	1
MEBHYDROLIN	PC3	0.845643438	1
FAMOTIDINE	PC3	0.845790612	1
BUDESONIDE	HL60	0.845803972	1
LATAMOXEF	MCF7	0.846445504	1
TETRAMISOLE	PC3	0.846605735	1
ZOXAZOLAMINE	PC3	0.847405981	1
ESTRONE	MCF7	0.847828812	1
HC TOXIN	MCF7	0.84804508	1
FENSPIRIDE	MCF7	0.848956876	1
NALIDIXIC ACID	HL60	0.84916115	1
DIAZOXIDE	MCF7	0.849182121	1
SULFADOXINE	MCF7	0.849407243	1
EPIANDROSTERONE	PC3	0.849479475	1
ETHAMBUTOL	HL60	0.849638545	1
PROPANTHELINE BROMIDE	HL60	0.849797553	1
TRANEXAMIC ACID	MCF7	0.85008187	1
PERPHENAZINE	MCF7	0.850306499	1
CINNARIZINE	MCF7	0.850979645	1
IOBENGUANE	HL60	0.851067399	1
CEFALEXIN	PC3	0.851384242	1
CEFAZOLIN	MCF7	0.851427789	1
PRAZIQUANTEL	PC3	0.85154257	1
BENFOTIAMINE	MCF7	0.851745882	1
ANTAZOLINE	HL60	0.85185904	1
IPRONIAZID	PC3	0.851875436	1
BRETYLIUM TOSILATE	MCF7	0.852099073	1
ETIFENIN	MCF7	0.853215386	1
NYSTATIN	MCF7	0.853280069	1
(-)-ISOPRENALINE	HL60	0.853437648	1
PYRITHYLDIONE	PC3	0.853595165	1
FLUOXETINE	MCF7	0.853883672	1
SULFAPYRIDINE	MCF7	0.854772961	1
FLUMEQUINE	PC3	0.854853039	1
FLURBIPROFEN	MCF7	0.854930779	1
ARTICAINE	PC3	0.855637161	1
DISULFIRAM	MCF7	0.855660232	1
ALFAXALONE	MCF7	0.855660232	1
SOLASODINE	PC3	0.856103108	1
CICLOSPORIN	PC3	0.856106875	1
DEXAMETHASONE	MCF7	0.856354378	1
LYNESTRENOL	MCF7	0.856545476	1
ROXARSONE	MCF7	0.85676647	1

MEPTAZINOL	MCF7	0.857649169	1
SB-203580	MCF7	0.858359471	1
SEROTONIN	MCF7	0.858449285	1
RESCINNAMINE	HL60	0.858602344	1
TRIAMTERENE	MCF7	0.859188968	1
OXEDRINE	HL60	0.859223651	1
TETRANDRINE	MCF7	0.859408426	1
MEPHENESIN	PC3	0.859846954	1
ANTIMYCIN A	PC3	0.860284967	1
TOMATIDINE	PC3	0.86030846	1
PALMATINE	PC3	0.860617824	1
METHOTREXATE	MCF7	0.860687665	1
HEXAMETHONIUM BROMIDE	HL60	0.861852688	1
DICOUMAROL	PC3	0.862031832	1
TELENZEPINE	PC3	0.862314688	1
ALTRETAMINE	HL60	0.862468558	1
HYDROCHLOROTHIAZIDE	MCF7	0.862684759	1
CEFAPIRIN	PC3	0.863083384	1
ETIDRONIC ACID	PC3	0.863543816	1
LISURIDE	MCF7	0.863553498	1
CHLORZOXAZONE	HL60	0.864003657	1
TRETINOIN	PC3	0.864400948	1
CLORGILINE	MCF7	0.864420137	1
PROCAINAMIDE	HL60	0.864768745	1
PROPOXYCAINE	PC3	0.864921565	1
PHA-00846566E	MCF7	0.865227006	1
PNU-0230031	MCF7	0.865623227	1
CEFIXIME	MCF7	0.866147084	1
OXYBENZONE	MCF7	0.866147084	1
TOMATIDINE	HL60	0.866293967	1
ALPHA-ESTRADIOL	MCF7	0.866435264	1
NARINGENIN	MCF7	0.866577496	1
IFOSFAMIDE	PC3	0.866598216	1
DOXORUBICIN	MCF7	0.867007376	1
CYTISINE	PC3	0.867205916	1
PERGOLIDE	MCF7	0.867436723	1
PIVMECILLINAM	PC3	0.867509366	1
DECAMETHONIUM BROMIDE	PC3	0.867660991	1
MESTRANOL	PC3	0.867964041	1
CLOPAMIDE	HL60	0.868115465	1
SULFINPYRAZONE	MCF7	0.868293814	1
MEXILETINE	PC3	0.868507752	1
ATOVAQUONE	MCF7	0.868720493	1
TODRALAZINE	PC3	0.868721556	1
VORINOSTAT	PC3	0.868935226	1
DOCOSAHEXAENOIC ACID ETHYL ESTER	MCF7	0.869022604	1
FLUOCINONIDE	PC3	0.869148761	1
ALCLOMETASONE	HL60	0.869324447	1
ADENOSINE PHOSPHATE	MCF7	0.869362162	1
RACECADOTRIL	PC3	0.869475267	1
3-ACETYLCOUMARIN	MCF7	0.869504856	1
NORCYCLOBENZAPRINE	MCF7	0.869575428	1
BETAXOLOL	MCF7	0.869788559	1
GENISTEIN	PC3	0.870113749	1
SENECIPHYLLINE	PC3	0.870829615	1
METRIZAMIDE	HL60	0.870979761	1
DIPHENHYDRAMINE	PC3	0.871276696	1
CAY-10397	MCF7	0.871579663	1
PIPERINE	PC3	0.871729468	1
BUCLADESINE	MCF7	0.871970114	1
METITEPINE	PC3	0.872028876	1

TRIMETHOBENZAMIDE	PC3	0.872124077	1
FULVESTRANT	PC3	0.872189594	1
ROTENONE	MCF7	0.872969277	1
ALFADOLONE	HL60	0.873372835	1
SULFAQUINOXALINE	MCF7	0.873391057	1
MAFENIDE	HL60	0.873521822	1
CP-319743	MCF7	0.87381229	1
AMBROXOL	HL60	0.873968372	1
METRIFONATE	MCF7	0.8740227	1
TRIMETHYLCOLCHICINIC ACID	MCF7	0.8740227	1
BENFLUOREX	PC3	0.87456281	1
VINCAMINE	MCF7	0.875287281	1
CHLOROPYRAMINE	MCF7	0.87549172	1
PRAMOCAINE	PC3	0.875600425	1
ISOMETHEPTENE	MCF7	0.875701029	1
HESPERETIN	MCF7	0.875910198	1
NIALAMIDE	MCF7	0.876328123	1
VINPOCETINE	HL60	0.876634654	1
MINAPRINE	HL60	0.876929523	1
PIRACETAM	MCF7	0.876953968	1
TRIHEXYPHENIDYL	MCF7	0.877076854	1
SULFAMETHOXAZOLE	PC3	0.877162305	1
RAMIFENAZONE	MCF7	0.877786481	1
PYRANTEL	PC3	0.877786481	1
MEFEXAMIDE	PC3	0.87825299	1
NU-1025	MCF7	0.878616761	1
CINCHONIDINE	MCF7	0.879031062	1
VINBLASTINE	PC3	0.87927844	1
ALPHA-ESTRADIOL	PC3	0.879282091	1
3-AMINOBENZAMIDE	MCF7	0.879716866	1
NORDIHYDROGUAIARETIC ACID	PC3	0.879857977	1
STROPHANTHIDIN	MCF7	0.880064355	1
LIDOFLAZINE	PC3	0.880154659	1
PROCYCLIDINE	HL60	0.880446169	1
TETRAETHYLENEPENTAMINE	PC3	0.880737396	1
(+/-)-CATECHIN	PC3	0.881464228	1
METRONIDAZOLE	MCF7	0.881505043	1
ECONAZOLE	PC3	0.881754465	1
CHLORPROTHIXENE	MCF7	0.882529856	1
CARTEOLOL	HL60	0.882623473	1
ASTEMIZOLE	HL60	0.882768058	1
MESTRANOL	MCF7	0.883143037	1
BETAMETHASONE	PC3	0.883489917	1
BETAMETHASONE	HL60	0.884209989	1
DEQUALINIUM CHLORIDE	MCF7	0.88456879	1
CORALYNE	PC3	0.884641172	1
PRASTERONE	MCF7	0.884771896	1
ANABASINE	MCF7	0.884974858	1
CICLACILLIN	PC3	0.885785266	1
GUANETHIDINE	HL60	0.885930842	1
CLOMIPRAMINE	PC3	0.886073778	1
BUPIVACAINE	MCF7	0.886189605	1
SELEGILINE	PC3	0.8866448	1
TTNPB	MCF7	0.887072307	1
PROGESTERONE	MCF7	0.88739915	1
THIOPERAMIDE	PC3	0.887783367	1
NIMESULIDE	HL60	0.887925362	1
(+)-CHELIDONINE	MCF7	0.888001963	1
FLUOXETINE	HL60	0.888350907	1
PROBUCOL	HL60	0.88849261	1
DILOXANIDE	MCF7	0.888803674	1

ESCULETIN	MCF7	0.889003737	1
PENTOXIFYLLINE	PC3	0.889603048	1
FENBUFEN	MCF7	0.889734051	1
IFENPRODIL	MCF7	0.889802526	1
BROMPHENIRAMINE	PC3	0.890187341	1
EDROPHONIUM CHLORIDE	MCF7	0.89020104	1
CELECOXIB	MCF7	0.890277196	1
ETACRYNIC ACID	MCF7	0.890328093	1
VIOMYCIN	PC3	0.89046877	1
OXYMETAZOLINE	HL60	0.890749905	1
ENALAPRIL	MCF7	0.89079771	1
GLIMEPIRIDE	HL60	0.890890362	1
NOVOBIOCIN	PC3	0.890996306	1
PENTETRAZOL	MCF7	0.890996306	1
LASALOCID	PC3	0.891171056	1
PARBENDAZOLE	PC3	0.891194754	1
EPIVINCAMINE	PC3	0.892151157	1
AMPROLIUM	PC3	0.892382341	1
PHENAZONE	MCF7	0.893127631	1
RIBAVIRIN	PC3	0.893405955	1
ADRENOSTERONE	MCF7	0.893564593	1
CP-320650-01	PC3	0.893634547	1
MIFEPRISTONE	PC3	0.894100463	1
NICERGOLINE	MCF7	0.894153709	1
LEVOBUNOLOL	PC3	0.894516273	1
THEOPHYLLINE	PC3	0.894793106	1
CAFFEIC ACID	PC3	0.89493141	1
TROLEANDOMYCIN	HL60	0.895069639	1
CHLORMEZANONE	PC3	0.895207794	1
CARTEOLOL	MCF7	0.895523085	1
SYROSINGOPINE	PC3	0.895621807	1
AMIODARONE	MCF7	0.895772258	1
SULFASALAZINE	MCF7	0.896169834	1
KETOCONAZOLE	PC3	0.896310328	1
VERAPAMIL	MCF7	0.896408078	1
ACEMETACIN	MCF7	0.896496711	1
MEGESTROL	MCF7	0.897272899	1
PHENIRAMINE	HL60	0.897408046	1
ESTRADIOL	MCF7	0.897444015	1
NOMIFENSINE	MCF7	0.89804667	1
TIAPROFENIC ACID	PC3	0.898091664	1
HOMOCHLORCYCLIZINE	MCF7	0.898239734	1
PYRANTEL	MCF7	0.898625406	1
NITROFURAL	MCF7	0.898818015	1
BUTACAINE	MCF7	0.898818015	1
CEFUROXIME	PC3	0.898909504	1
METENEPROST	MCF7	0.899010471	1
TIAPROFENIC ACID	MCF7	0.899970468	1
AZAPROPAZONE	PC3	0.900131127	1
BERBERINE	MCF7	0.900162009	1
TUBOCURARINE CHLORIDE	PC3	0.900536961	1
MEPYRAMINE	HL60	0.900807134	1
NEFOPAM	HL60	0.900942106	1
MECLOZINE	MCF7	0.901203072	1
PARBENDAZOLE	MCF7	0.901308044	1
LORACARBEF	HL60	0.901481226	1
CEFIXIME	HL60	0.901615814	1
NEFOPAM	PC3	0.901750325	1
ISOETARINE	MCF7	0.902068998	1
LEVODOPA	PC3	0.902258852	1
OXEDRINE	PC3	0.9022876	1

ECONAZOLE	MCF7	0.902448551	1
YOHIMBINE	PC3	0.902555776	1
SALBUTAMOL	HL60	0.902689748	1
0316684-0000	PC3	0.902827488	1
URSOLIC ACID	MCF7	0.903016724	1
FURSULTIAMINE	PC3	0.90335845	1
GINKGOLIDE A	MCF7	0.903583506	1
ALLANTOIN	PC3	0.903960585	1
ETHAVERINE	MCF7	0.903960585	1
ORNIDAZOLE	MCF7	0.904337045	1
TERAZOSIN	PC3	0.904424347	1
FASUDIL	PC3	0.904557234	1
GUANADREL	HL60	0.904822777	1
FLUTAMIDE	PC3	0.905088098	1
VIOMYCIN	MCF7	0.905275472	1
HEXYLCAINE	PC3	0.905617535	1
CORBADRINE	HL60	0.906013861	1
SPAGLUMIC ACID	MCF7	0.906409483	1
PODOPHYLLOTOXIN	MCF7	0.906582717	1
PIROXICAM	HL60	0.90667284	1
FURAZOLIDONE	HL60	0.906935883	1
CEFEPIME	MCF7	0.906954807	1
PHA-00767505E	PC3	0.907882282	1
MG-262	MCF7	0.908377002	1
COPPER SULFATE	MCF7	0.908621428	1
CLONIDINE	PC3	0.90876838	1
ISOXSUPRINE	MCF7	0.909174129	1
TERAZOSIN	HL60	0.909419096	1
AZTREONAM	PC3	0.909725406	1
RILUZOLE	PC3	0.91009213	1
VERAPAMIL	HL60	0.910585391	1
PIRENPERONE	HL60	0.910714582	1
ASIATICOSIDE	PC3	0.910843694	1
TRIPROLIDINE	MCF7	0.911188488	1
DYDROGESTERONE	MCF7	0.911370657	1
AMPHOTERICIN B	MCF7	0.911370657	1
MEFENAMIC ACID	HL60	0.911616688	1
GUANFACINE	MCF7	0.911696723	1
CORTISONE	MCF7	0.911916205	1
ALFADOLONE	MCF7	0.912898619	1
CORTICOSTERONE	PC3	0.913154043	1
ESTROPIPATE	PC3	0.913663928	1
KANAMYCIN	MCF7	0.913724284	1
10-METHOXYHARMALAN	PC3	0.914299471	1
KETOTIFEN	PC3	0.914553124	1
RAUBASINE	MCF7	0.915337789	1
RIFAMPICIN	PC3	0.91569056	1
CINOXACIN	MCF7	0.915694566	1
METIXENE	PC3	0.915942433	1
CHICAGO SKY BLUE 6B	PC3	0.916068247	1
IPRATROPIUM BROMIDE	MCF7	0.91619398	1
GLICLAZIDE	MCF7	0.91693817	1
LYSERGOL	HL60	0.916946672	1
SURAMIN SODIUM	PC3	0.917292019	1
AMINOPHYLLINE	MCF7	0.917645216	1
11-DEOXY-16,16-DIMETHYLPROSTAGLANDIN E2	PC3	0.91799776	1
CYPROHEPTADINE	MCF7	0.918349648	1
2-DEOXY-D-GLUCOSE	MCF7	0.918443245	1
TRICHOSTATIN A	MCF7	0.918651342	1
CLEMIZOLE	MCF7	0.918700882	1
ESTRONE	PC3	0.918939484	1

CARBINOXAMINE	PC3	0.919063339	1
METHACHOLINE CHLORIDE	PC3	0.919310801	1
PHENETICILLIN	MCF7	0.919401378	1
KAEMPFEROL	MCF7	0.919576091	1
CYCLOPENTOLATE	PC3	0.919928019	1
PHENETICILLIN	PC3	0.920051215	1
SULMAZOLE	MCF7	0.920174329	1
DIPYRIDAMOLE	MCF7	0.920409973	1
LEVAMISOLE	MCF7	0.920447186	1
METHAZOLAMIDE	MCF7	0.920967862	1
ETICLOPRIDE	MCF7	0.920967862	1
LOPERAMIDE	PC3	0.92114109	1
THIOCOLCHICOSIDE	PC3	0.92127864	1
MEBEVERINE	MCF7	0.921314152	1
TESTOSTERONE	MCF7	0.92147152	1
IONOMYCIN	MCF7	0.921683084	1
VELNACRINE	MCF7	0.921832345	1
OUABAIN	MCF7	0.922004744	1
CICLOHEXIMIDE	MCF7	0.922176977	1
H-89	MCF7	0.922254619	1
LEVONORGESTREL	MCF7	0.922316282	1
HYDRASTINE HYDROCHLORIDE	HL60	0.922376243	1
OXANTEL	MCF7	0.922692678	1
COLCHICINE	PC3	0.923949772	1
AMILORIDE	PC3	0.92423078	1
IMATINIB	MCF7	0.924310891	1
DEXPROPRANOLOL	PC3	0.924911079	1
ETILEFRINE	PC3	0.925150567	1
METAMIZOLE SODIUM	MCF7	0.925241437	1
INDOPROFEN	MCF7	0.925586597	1
CEFALOTIN	MCF7	0.925586597	1
ETHIONAMIDE	MCF7	0.925747818	1
ELDELINE	MCF7	0.925861845	1
(-)-ATENOLOL	PC3	0.925986129	1
BETONICINE	MCF7	0.926068142	1
NOCODAZOLE	HL60	0.926224103	1
DECAMETHONIUM BROMIDE	MCF7	0.92626047	1
PRAZOSIN	PC3	0.926580431	1
TRIMIPRAMINE	MCF7	0.926596395	1
KETOPROFEN	HL60	0.926699039	1
NORETYNODREL	MCF7	0.926764104	1
NIFLUMIC ACID	MCF7	0.926931644	1
SULFAMERAZINE	PC3	0.927172623	1
RIMEXOLONE	MCF7	0.927266217	1
RAUBASINE	PC3	0.927408908	1
DEXAMETHASONE	PC3	0.92743325	1
CAY-10397	PC3	0.927600113	1
PIRLINDOLE	MCF7	0.927766806	1
DACARBAZINE	PC3	0.927880461	1
TOLAZOLINE	MCF7	0.92793333	1
TROLEANDOMYCIN	PC3	0.92793333	1
SULFACETAMIDE	MCF7	0.927998138	1
TETRACYCLINE	HL60	0.928115729	1
GANCICLOVIR	PC3	0.928233236	1
SULINDAC	PC3	0.928265869	1
PIRENZEPINE	MCF7	0.928597728	1
TILETAMINE	MCF7	0.928763403	1
ETAMIVAN	MCF7	0.928928907	1
DIOXYBENZONE	PC3	0.929170227	1
BROMPHENIRAMINE	MCF7	0.929424397	1
RITODRINE	HL60	0.929636679	1

DEBRISOQUINE	MCF7	0.929753871	1
LEVOCABASTINE	MCF7	0.9302468	1
DIFLUNISAL	HL60	0.930333795	1
OXETACAINE	MCF7	0.930574564	1
GEFITINIB	HL60	0.930796828	1
TRIDIHEXETHYL	MCF7	0.930901643	1
(-)-ISOPRENALINE	PC3	0.93102783	1
XYLOMETAZOLINE	MCF7	0.931064925	1
ORCIPRENALINE	MCF7	0.931228036	1
ISOTRETINOIN	PC3	0.931258489	1
ACETYLSALICYLSALICYLIC ACID	HL60	0.931488804	1
LIDOCAINE	MCF7	0.931716336	1
NADOLOL	HL60	0.931718776	1
RITODRINE	MCF7	0.932203088	1
LAUDANOSINE	MCF7	0.93268829	1
CITALOPRAM	PC3	0.932849678	1
ETAMIVAN	PC3	0.933091373	1
CETIRIZINE	PC3	0.933205195	1
N-ACETYLMURAMIC ACID	HL60	0.933318931	1
AMPHOTERICIN B	HL60	0.933999527	1
MIMOSINE	MCF7	0.93413456	1
NIRIDAZOLE	MCF7	0.934613531	1
CHLORPROPAMIDE	MCF7	0.934758589	1
CYTISINE	HL60	0.935014567	1
METHOTREXATE	HL60	0.936198786	1
PIZOTIFEN	HL60	0.936245596	1
PINACIDIL	MCF7	0.936670958	1
BUTYL HYDROXYBENZOATE	MCF7	0.936699524	1
ETOMIDATE	MCF7	0.936984864	1
RIBOSTAMYCIN	PC3	0.937244938	1
BICUCULLINE	PC3	0.93746605	1
CALCIUM PANTOTHENATE	MCF7	0.937766561	1
CEFACLOR	MCF7	0.937922373	1
FENOFIBRATE	MCF7	0.93823347	1
STROPHANTHIDIN	PC3	0.938346984	1
AMPICILLIN	PC3	0.938456705	1
CINOXACIN	PC3	0.938566338	1
TETRAETHYLENEPENTAMINE	ssMCF7	0.939003988	1
AMINOPHENAZONE	MCF7	0.939008131	1
SPIRAMYCIN	PC3	0.93931676	1
HEXESTROL	PC3	0.939549065	1
NIZATIDINE	HL60	0.93987505	1
NORDIHYDROGUAIARETIC ACID	MCF7	0.940028034	1
PRESTWICK-1083	MCF7	0.940308458	1
SEMUSTINE	PC3	0.940544199	1
IVERMECTIN	MCF7	0.940696832	1
PYRIDOXINE	PC3	0.940955909	1
NIPECOTIC ACID	MCF7	0.941153663	1
IOHEXOL	PC3	0.941278436	1
METICRANE	MCF7	0.941457328	1
METHYLDOPA	PC3	0.9418142	1
NIFLUMIC ACID	PC3	0.941921086	1
TETRAHYDROALSTONINE	MCF7	0.94206252	1
CHLORTALIDONE	PC3	0.942347737	1
PREDNISONE	PC3	0.942514541	1
IMIPENEM	MCF7	0.942814995	1
MYOSMINE	MCF7	0.943088769	1
BENPERIDOL	MCF7	0.943114734	1
HESPERIDIN	MCF7	0.943413756	1
MEBENDAZOLE	MCF7	0.943712062	1
CAPTOPRIL	PC3	0.944158173	1

YOHIMBINE	MCF7	0.944184241	1
TROLEANDOMYCIN	MCF7	0.944249958	1
MOXISYLYTE	MCF7	0.944306518	1
CP-319743	PC3	0.944454683	1
CORYNANTHINE	PC3	0.944459521	1
FLUCLOXACILLIN	PC3	0.94487757	1
CAMPTOTHECIN	MCF7	0.944898097	1
TERFENADINE	HL60	0.94519016	1
RILUZOLE	MCF7	0.945339891	1
FLUDROCORTISONE	MCF7	0.945375421	1
PIPENZOLATE BROMIDE	MCF7	0.945486794	1
HESPERETIN	HL60	0.945501939	1
OXYBENZONE	HL60	0.945812906	1
PROPOFOL	MCF7	0.945926421	1
ARECOLINE	PC3	0.946638172	1
PROCAINE	MCF7	0.946655515	1
COPPER SULFATE	ssMCF7	0.946740923	1
AMIKACIN	MCF7	0.947380078	1
SR-95531	HL60	0.947457639	1
CHLORPROPAMIDE	HL60	0.947559664	1
STREPTOMYCIN	PC3	0.947865193	1
BETA-ESCIN	MCF7	0.947883002	1
CYCLOSERINE	MCF7	0.948100097	1
BENZOCAINE	MCF7	0.948243555	1
IMIPENEM	PC3	0.948574907	1
AMOXICILLIN	MCF7	0.948815562	1
AG-012559	MCF7	0.948877702	1
PHENELZINE	PC3	0.948958107	1
THIETHYLPERAZINE	HL60	0.948978452	1
PICROTOXININ	MCF7	0.949384647	1
DEMECARIUM BROMIDE	HL60	0.949581028	1
EUCATROPINE	PC3	0.949668093	1
SAQUINAVIR	HL60	0.950080658	1
COTININE	MCF7	0.950149972	1
DIPROPHYLLINE	MCF7	0.950232785	1
CP-863187	PC3	0.950934521	1
METHYLERGOMETRINE	HL60	0.951073038	1
FOLIOSIDINE	MCF7	0.9511806	1
HYDROCOTARNINE	MCF7	0.951909223	1
SULFADIMETHOXINE	PC3	0.952047729	1
NAPELLINE	PC3	0.952544349	1
MOMETASONE	PC3	0.952641698	1
CYTISINE	MCF7	0.952874883	1
LOPERAMIDE	HL60	0.953127054	1
IOHEXOL	MCF7	0.953285961	1
MEPRYLCAINE	MCF7	0.953559087	1
BIOTIN	MCF7	0.953898806	1
DILOXANIDE	PC3	0.954090816	1
METHAPYRILENE	HL60	0.954186682	1
CHLORCYCLIZINE	MCF7	0.954212694	1
DEMECARIUM BROMIDE	MCF7	0.954509176	1
MERBROMIN	MCF7	0.954644159	1
FENBENDAZOLE	MCF7	0.954855135	1
FLUDROXYCORTIDE	MCF7	0.954913567	1
CONESSINE	PC3	0.95523506	1
KAEMPFEROL	PC3	0.955707866	1
PHENTOLAMINE	MCF7	0.955875116	1
3-HYDROXY-DL-KYNURENINE	PC3	0.956084429	1
DIHYDROSTREPTOMYCIN	PC3	0.956116662	1
YOHIMBIC ACID	PC3	0.956178336	1
ACETYLSALICYLIC ACID	PC3	0.956249404	1

PROCAINAMIDE	MCF7	0.956249404	1
PENTETIC ACID	HL60	0.956272149	1
LOMEFLOXACIN	HL60	0.956365869	1
FOSFOSAL	MCF7	0.95638196	1
STACHYDRINE	PC3	0.956459495	1
PRAMOCAINE	MCF7	0.956508536	1
ETHISTERONE	PC3	0.956514328	1
GABAPENTIN	MCF7	0.956646508	1
MOLSIDOMINE	PC3	0.957019283	1
HELIOTRINE	MCF7	0.957047058	1
AZACYCLONOL	MCF7	0.957173354	1
MINAPRINE	MCF7	0.957304596	1
CLOZAPINE	PC3	0.957315145	1
VANCOMYCIN	PC3	0.957483192	1
CEFOTETAN	MCF7	0.957668098	1
CARBAMAZEPINE	MCF7	0.957672029	1
SC-19220	PC3	0.957697195	1
ANDROSTERONE	MCF7	0.957827685	1
DEXPANTHENOL	MCF7	0.9580881	1
TOLMETIN	MCF7	0.958864821	1
RANITIDINE	HL60	0.959042885	1
PHA-00665752	MCF7	0.959133783	1
6-BENZYLAMINOPURINE	PC3	0.959586852	1
SC-58125	MCF7	0.959634742	1
RESVERATROL	MCF7	0.959692844	1
ACEPROMAZINE	MCF7	0.9597624	1
METANEPHRINE	PC3	0.960144236	1
CHLOROPYRAMINE	PC3	0.960396412	1
ZALCITABINE	MCF7	0.960524365	1
DIENESTROL	MCF7	0.960650695	1
NALOXONE	HL60	0.96075375	1
VORINOSTAT	MCF7	0.960860205	1
PIRENPERONE	PC3	0.960931849	1
TRIFLUPROMAZINE	HL60	0.961020755	1
PREDNISOLONE	MCF7	0.96108843	1
SALBUTAMOL	MCF7	0.961154113	1
ETYNODIOL	PC3	0.961286905	1
DEMECOLCINE	MCF7	0.961640439	1
PHENAZONE	PC3	0.961904588	1
NOMEGESTROL	MCF7	0.962275663	1
PHA-00816795	PC3	0.962522797	1
HYDROCHLOROTHIAZIDE	PC3	0.962769166	1
DIMENHYDRINATE	PC3	0.963039306	1
PEMPIDINE	PC3	0.963557569	1
BUFEXAMAC	MCF7	0.963625427	1
FENBUFEN	PC3	0.963746983	1
8-AZAGUANINE	MCF7	0.963868347	1
CEFADROXIL	PC3	0.963901159	1
ACONITINE	MCF7	0.963989519	1
FINASTERIDE	PC3	0.964110499	1
BISOPROLOL	MCF7	0.964231286	1
CEFACLOR	PC3	0.964413664	1
OCTOPAMINE	MCF7	0.964472285	1
COTININE	HL60	0.964498745	1
ETOFENAMATE	MCF7	0.96519066	1
PROMAZINE	MCF7	0.965282634	1
PYRITHYLDIONE	MCF7	0.965428576	1
ENILCONAZOLE	PC3	0.965679766	1
CLEBOPRIDE	MCF7	0.965784002	1
SULFABENZAMIDE	HL60	0.966096975	1
PIPERACILLIN	MCF7	0.966151974	1

MEGESTROL	PC3	0.966263182	1
THIOGUANOSINE	MCF7	0.966372514	1
FOLIC ACID	PC3	0.966511767	1
15-DELTA PROSTAGLANDIN J2	MCF7	0.966574231	1
THEOBROMINE	PC3	0.966594435	1
HYOSCYAMINE	PC3	0.966606564	1
CARCININE	HL60	0.966677006	1
MEPENZOLATE BROMIDE	MCF7	0.966956185	1
NALOXONE	MCF7	0.966963422	1
SCOULERINE	PC3	0.96725229	1
PSEUDOPELLETIERINE	HL60	0.967497385	1
TIMOLOL	PC3	0.967741605	1
RACECADOTRIL	MCF7	0.967765175	1
N-ACETYL-L-ASPARTIC ACID	HL60	0.967822817	1
FLUOXETINE	PC3	0.968065871	1
ERGOCALCIFEROL	PC3	0.968146694	1
N6-METHYLADENOSINE	MCF7	0.968223178	1
BUMETANIDE	PC3	0.96822742	1
BRETYLIUM TOSILATE	PC3	0.968388579	1
RIBOFLAVIN	PC3	0.968469013	1
ORPHENADRINE	MCF7	0.968697179	1
VITEXIN	HL60	0.968869719	1
PRESTWICK-857	MCF7	0.969129829	1
ISOCORYDINE	HL60	0.969267985	1
CLOFAZIMINE	PC3	0.969347345	1
RILUZOLE	HL60	0.969426608	1
ROTENONE	PC3	0.969466602	1
STACHYDRINE	HL60	0.96958484	1
REMOXIPRIDE	MCF7	0.96969014	1
VALDECOXIB	MCF7	0.96974268	1
MYRICETIN	PC3	0.969821454	1
HARMAN	MCF7	0.969912895	1
TRIFLUOPERAZINE	MCF7	0.970123075	1
PHENOXYBENZAMINE	MCF7	0.97034135	1
OXAMIC ACID	MCF7	0.970448116	1
METAMPICILLIN	PC3	0.970686363	1
PNU-0230031	PC3	0.970822452	1
RIBOSTAMYCIN	MCF7	0.971014906	1
NIFENAZONE	HL60	0.971222615	1
PRESTWICK-860	MCF7	0.971450211	1
PACLITAXEL	HL60	0.971606179	1
BIPERIDEN	MCF7	0.971811292	1
BUTEIN	PC3	0.972139033	1
GLY-HIS-LYS	PC3	0.972204414	1
PRACTOLOL	HL60	0.972214761	1
BUTIROSIN	PC3	0.972516686	1
RESVERATROL	PC3	0.972524686	1
ETICLOPRIDE	HL60	0.97281703	1
CYPROTERONE	MCF7	0.97284318	1
NETILMICIN	PC3	0.97289187	1
TRIBENOSIDE	MCF7	0.97294895	1
ACACETIN	MCF7	0.972987373	1
ELDELINE	PC3	0.973265071	1
BENZATHINE BENZYLPENICILLIN	MCF7	0.973474829	1
THALIDOMIDE	MCF7	0.973548133	1
PRIMAQUINE	MCF7	0.973579411	1
CALMIDAZOLIUM	MCF7	0.973683795	1
OXOLAMINE	PC3	0.973855775	1
GW-8510	PC3	0.973995756	1
SUXIBUZONE	MCF7	0.974202738	1
CEFTAZIDIME	MCF7	0.974222048	1

FLUPENTIXOL	MCF7	0.974305932	1
ATROPINE METHONITRATE	HL60	0.97458584	1
DOXYCYCLINE	MCF7	0.9746437	1
AMPYRONE	MCF7	0.974716718	1
COBALT CHLORIDE	MCF7	0.974810733	1
QUINPIROLE	HL60	0.974875085	1
15-DELTA PROSTAGLANDIN J2	SKMEL5	0.974947147	1
PRALIDOXIME	MCF7	0.975225725	1
PIRETANIDE	PC3	0.975305967	1
METERGOLINE	MCF7	0.975326929	1
CYCLIZINE	PC3	0.975377432	1
MIANSERIN	PC3	0.975427933	1
TAMOXIFEN	MCF7	0.975436865	1
GLAFENINE	PC3	0.975448797	1
ETIOCHOLANOLONE	PC3	0.975629345	1
LOPERAMIDE	MCF7	0.97576087	1
MIDODRINE	HL60	0.975804129	1
CANRENOIC ACID	PC3	0.975829958	1
MEMANTINE	MCF7	0.975829958	1
PENBUTOLOL	HL60	0.975945564	1
THIAMINE	MCF7	0.976129381	1
TREMORINE	PC3	0.976227235	1
BENZETHONIUM CHLORIDE	MCF7	0.97622879	1
4-HYDROXYPHENAZONE	HL60	0.976297403	1
NAPROXEN	HL60	0.976327998	1
4,5-DIANILINOPHTHALIMIDE	MCF7	0.976437439	1
AMINOHIPPURIC ACID	MCF7	0.976525815	1
QUERCETIN	MCF7	0.976565685	1
ADIPIODONE	HL60	0.976577076	1
ASTEMIZOLE	MCF7	0.97682104	1
BERGENIN	HL60	0.976855148	1
DOSULEPIN	MCF7	0.977114462	1
HOMOSALATE	PC3	0.977114462	1
NIFUROXAZIDE	MCF7	0.977211869	1
NILUTAMIDE	PC3	0.977269253	1
NOVOBIOCIN	ssMCF7	0.977406486	1
PRESTWICK-983	HL60	0.977474952	1
COLCHICINE	MCF7	0.977529447	1
FLUDROCORTISONE	HL60	0.977543318	1
PROCARBAZINE	MCF7	0.977599491	1
CEPHAELINE	HL60	0.977611584	1
DIOXYBENZONE	MCF7	0.977983902	1
MOXONIDINE	MCF7	0.978221456	1
AZATHIOPRINE	MCF7	0.978479426	1
TESTOSTERONE	PC3	0.978556756	1
METYRAPONE	MCF7	0.978648881	1
CHLORAMPHENICOL	MCF7	0.978756728	1
CHLOROQUINE	HL60	0.979021948	1
CISAPRIDE	MCF7	0.979117825	1
CANADINE	MCF7	0.979396772	1
BIPERIDEN	PC3	0.979416753	1
BENDROFLUMETHIAZIDE	PC3	0.979489351	1
NIFUROXAZIDE	PC3	0.979742982	1
ZAPRINAST	HL60	0.979807925	1
AMODIAQUINE	MCF7	0.979857645	1
CP-944629	PC3	0.979949214	1
MEDRYSONE	MCF7	0.980246571	1
ISOPROPAMIDE IODIDE	MCF7	0.980313465	1
MOLSIDOMINE	HL60	0.980323828	1
MIDECAMYCIN	PC3	0.980584528	1
DIPERODON	MCF7	0.980642979	1

IRINOTECAN	MCF7	0.980674477	1
METAMPICILLIN	HL60	0.980706505	1
THIORIDAZINE	MCF7	0.980992223	1
MONASTROL	PC3	0.981336191	1
BUTEIN	MCF7	0.981398602	1
CROTAMITON	PC3	0.981460911	1
NADOLOL	MCF7	0.981474891	1
VINPOCETINE	PC3	0.981523118	1
FLUOCINONIDE	MCF7	0.981631023	1
2,6-DIMETHYLPIPERIDINE	PC3	0.981738041	1
ISOXICAM	HL60	0.981832631	1
DISOPYRAMIDE	PC3	0.981894229	1
REPAGLINIDE	MCF7	0.98199936	1
APOMORPHINE	MCF7	0.982086059	1
PHENTOLAMINE	PC3	0.982086059	1
CEFAZOLIN	HL60	0.982261677	1
HYDROQUININE	HL60	0.982383346	1
DIHYDROSTREPTOMYCIN	MCF7	0.982430821	1
SEMUSTINE	MCF7	0.982516502	1
HYMECROMONE	PC3	0.98262546	1
PROPYLTHIOURACIL	PC3	0.982865944	1
AMANTADINE	PC3	0.982925809	1
MIDODRINE	PC3	0.983110558	1
SOLANINE	MCF7	0.983194606	1
DYDROGESTERONE	PC3	0.983282861	1
LEVAMISOLE	PC3	0.983401061	1
NABUMETONE	MCF7	0.983445526	1
AG-028671	MCF7	0.983694773	1
PRESTWICK-1103	PC3	0.983869772	1
DROFENINE	MCF7	0.983941845	1
PIRACETAM	HL60	0.984101672	1
PHA-00846566E	PC3	0.98426863	1
ETOPOSIDE	PC3	0.984331934	1
LETROZOLE	MCF7	0.984511566	1
TOMELUKAST	MCF7	0.984674251	1
ALBENDAZOLE	MCF7	0.985150367	1
MAPROTILINE	HL60	0.98545862	1
15(S)-15-METHYLPROSTAGLANDIN E2	MCF7	0.985620837	1
CYPROTERONE	PC3	0.985897784	1
WITHAFERIN A	MCF7	0.985930368	1
CYCLIC ADENOSINE MONOPHOSPHATE	HL60	0.986060772	1
DAUNORUBICIN	PC3	0.986383964	1
LUMICOLCHICINE	MCF7	0.986437469	1
PRESTWICK-682	MCF7	0.986464112	1
ACEPROMAZINE	PC3	0.98649087	1
PACLITAXEL	MCF7	0.986616234	1
LEVODOPA	HL60	0.986809111	1
FLUPHENAZINE	MCF7	0.986817319	1
PRAZOSIN	MCF7	0.98706515	1
N-ACETYL-L-LEUCINE	PC3	0.987123634	1
SCOULERINE	HL60	0.987227648	1
METOPROLOL	PC3	0.987485873	1
NISOXETINE	MCF7	0.987861827	1
MG-132	MCF7	0.988245016	1
FLUTICASONE	MCF7	0.98828768	1
DIMETHADIONE	MCF7	0.98835793	1
CISAPRIDE	PC3	0.988443514	1
BEPRIDIL	MCF7	0.988567432	1
DIAZOXIDE	PC3	0.988775065	1
BCB000039	MCF7	0.988980817	1
OXYBENZONE	PC3	0.989029039	1

CAPSAICIN	PC3	0.989125172	1
PIROMIDIC ACID	PC3	0.989173082	1
CLOXACILLIN	MCF7	0.989252266	1
2-AMINOBENZENESULFONAMIDE	HL60	0.989363682	1
BETULIN	HL60	0.989552617	1
OXAMNIQUINE	MCF7	0.98958688	1
FLUCLOXACILLIN	MCF7	0.989719267	1
ROXITHROMYCIN	PC3	0.989739885	1
VANOXERINE	PC3	0.989786442	1
TRIMETAZIDINE	MCF7	0.989850821	1
TRANYLCYPROMINE	HL60	0.990200759	1
GUAIFENESIN	MCF7	0.99020592	1
BLEBBISTATIN	MCF7	0.990240475	1
LASALOCID	MCF7	0.990432483	1
PROPOXYCAINE	HL60	0.990472274	1
BACITRACIN	MCF7	0.990496069	1
FURALTADONE	MCF7	0.990519072	1
CARISOPRODOL	HL60	0.990740029	1
PARTHENOLIDE	MCF7	0.990748319	1
CHLORPROMAZINE	MCF7	0.990934033	1
DEFEROXAMINE	MCF7	0.990935241	1
PIRENPERONE	MCF7	0.991130324	1
COPPER SULFATE	PC3	0.991134605	1
CHLORHEXIDINE	MCF7	0.991242781	1
CHLORPHENAMINE	HL60	0.991264246	1
ACEMETACIN	PC3	0.991435636	1
NYSTATIN	PC3	0.991520702	1
MIFEPRISTONE	HL60	0.991647516	1
TETRACAINE	MCF7	0.99166444	1
BUTAMBEN	PC3	0.991815133	1
PRESTWICK-675	MCF7	0.991898312	1
IPRONIAZID	HL60	0.992186134	1
ACETOHEXAMIDE	MCF7	0.992191473	1
ASTEMIZOLE	PC3	0.992248982	1
DIHYDROSTREPTOMYCIN	HL60	0.992267424	1
MINOCYCLINE	PC3	0.992348294	1
NILUTAMIDE	HL60	0.992388572	1
MEPHENYTOIN	HL60	0.992428744	1
CYCLOBENZAPRINE	PC3	0.992468811	1
CALCIUM FOLINATE	HL60	0.992508773	1
ERYTHROMYCIN	HL60	0.992588382	1
QUERCETIN	PC3	0.992628029	1
BENZYLPENICILLIN	PC3	0.992746339	1
HYCANTHONE	HL60	0.992902612	1
ETODOLAC	MCF7	0.992977475	1
CICLOPIROX	PC3	0.993095586	1
DYCLONINE	PC3	0.99324807	1
ACICLOVIR	HL60	0.993285928	1
ETHISTERONE	MCF7	0.993308731	1
DIETHYLCARBAMAZINE	MCF7	0.993355008	1
PYRIDOXINE	HL60	0.993398868	1
PENBUTOLOL	MCF7	0.993408098	1
PRAZIQUANTEL	HL60	0.993436305	1
U0125	PC3	0.993584995	1
GLIPIZIDE	HL60	0.993658708	1
CEFOPERAZONE	MCF7	0.993768486	1
TOCAINIDE	MCF7	0.993773825	1
NOCODAZOLE	PC3	0.99387642	1
FELBINAC	PC3	0.993877314	1
ISONIAZID	MCF7	0.99397817	1
BUPIVACAINE	PC3	0.994056583	1

BCB000038	PC3	0.994129211	1
RESERPINE	MCF7	0.994198098	1
TELENZEPINE	MCF7	0.99427835	1
CIPROFLOXACIN	HL60	0.994303125	1
DEHYDROCHOLIC ACID	MCF7	0.99432764	1
LISURIDE	HL60	0.994476055	1
VINBURNINE	PC3	0.994510324	1
BROMPERIDOL	MCF7	0.994522688	1
PHENFORMIN	PC3	0.994570921	1
AMANTADINE	HL60	0.994646343	1
MICONAZOLE	MCF7	0.994666752	1
MONENSIN	HL60	0.994713717	1
LEFLUNOMIDE	HL60	0.994747246	1
DL-ALPHA TOCOPHEROL	HL60	0.994813985	1
AMYLOCAINE	PC3	0.994855875	1
STREPTOZOCIN	PC3	0.994880302	1
FAMPROFAZONE	PC3	0.994902626	1
CARBACHOL	MCF7	0.994949166	1
HEXYLCAINE	HL60	0.995173483	1
CHLORMEZANONE	MCF7	0.995178686	1
DIFENIDOL	MCF7	0.995223954	1
BRINZOLAMIDE	PC3	0.995269303	1
RICININE	MCF7	0.995358487	1
ONDANSETRON	HL60	0.995458081	1
MEVALOLACTONE	PC3	0.995489173	1
ATRACTYLOSIDE	PC3	0.995491111	1
MONORDEN	SKMEL5	0.995551039	1
PICOTAMIDE	PC3	0.995578467	1
TETRANDRINE	HL60	0.995581812	1
EPIANDROSTERONE	MCF7	0.995621826	1
SENECIPHYLLINE	HL60	0.995643041	1
BETA-ESCIN	PC3	0.995664973	1
SULFASALAZINE	HL60	0.995703846	1
PROCHLORPERAZINE	MCF7	0.995818709	1
HYDROXYACHILLIN	HL60	0.995913319	1
MITOXANTRONE	MCF7	0.995961057	1
HALOPERIDOL	PC3	0.9961016	1
GOSSYPOL	PC3	0.996125575	1
CIMETIDINE	HL60	0.996146344	1
ATROPINE OXIDE	HL60	0.996174994	1
LEVOMEPROMAZINE	PC3	0.996206558	1
DICYCLOVERINE	HL60	0.996231975	1
DIHYDROERGOCRISTINE	PC3	0.996260306	1
NISOXETINE	PC3	0.996344662	1
DANTROLENE	MCF7	0.996424801	1
HOMOCHLORCYCLIZINE	HL60	0.996455647	1
TTNPB	PC3	0.996510501	1
SULCONAZOLE	MCF7	0.996560456	1
LITHOCHOLIC ACID	HL60	0.99656493	1
FLUSPIRILENE	PC3	0.996778388	1
DYCLONINE	MCF7	0.996786811	1
EQUILIN	MCF7	0.996797809	1
ADENOSINE PHOSPHATE	HL60	0.997085797	1
ESCULIN	PC3	0.997110722	1
AMITRIPTYLINE	MCF7	0.997150367	1
PODOPHYLLOTOXIN	PC3	0.997184857	1
DIPIVEFRINE	HL60	0.997209355	1
MECAMYLAMINE	PC3	0.997233747	1
PIPERACILLIN	PC3	0.997250935	1
TENOXICAM	HL60	0.997377859	1
PENTOLONIUM	PC3	0.997386492	1

TRAZODONE	MCF7	0.997419848	1
THIOSTREPTON	MCF7	0.99748592	1
PROMETHAZINE	PC3	0.997495019	1
HYDRALAZINE	HL60	0.99751813	1
DIPERODON	HL60	0.997541136	1
DROPERIDOL	PC3	0.997632089	1
(+/-)-CATECHIN	HL60	0.997699183	1
PIZOTIFEN	PC3	0.997873402	1
PRESTWICK-972	HL60	0.997915888	1
METENEPROST	PC3	0.997924507	1
DIMETHYLOXALYLGLYCINE	PC3	0.997936971	1
SC-58125	HL60	0.997957947	1
INDOPROFEN	HL60	0.998020235	1
PRESTWICK-983	MCF7	0.998070996	1
TRIPROLIDINE	PC3	0.99808156	1
KANAMYCIN	HL60	0.998201324	1
SECURININE	PC3	0.99824039	1
METAMIZOLE SODIUM	PC3	0.9982671	1
CHLORAMBUCIL	PC3	0.998294259	1
NICARDIPINE	HL60	0.998336184	1
DEXIBUPROFEN	HL60	0.998355022	1
MEPHENTERMINE	HL60	0.998373752	1
CLEMASTINE	MCF7	0.998426845	1
HYMECROMONE	HL60	0.998429303	1
NARINGENIN	HL60	0.998465801	1
ATROPINE	HL60	0.99848389	1
BUPROPION	PC3	0.998501872	1
DISOPYRAMIDE	MCF7	0.998503828	1
NYSTATIN	HL60	0.998537514	1
SULPIRIDE	HL60	0.998572728	1
AMITRIPTYLINE	PC3	0.998578884	1
MEBHYDROLIN	HL60	0.998590175	1
SULFADIMETHOXINE	HL60	0.998607514	1
MEBENDAZOLE	HL60	0.998725892	1
IOPROMIDE	PC3	0.998758751	1
DOMPERIDONE	MCF7	0.998769609	1
DOBUTAMINE	HL60	0.998791181	1
DOMPERIDONE	PC3	0.998870381	1
PRESTWICK-685	HL60	0.998885899	1
OXYTETRACYCLINE	HL60	0.998901311	1
PIPENZOLATE BROMIDE	PC3	0.998916615	1
PF-01378883-00	MCF7	0.998925246	1
PIPERLONGUMINE	MCF7	0.998946902	1
PROGUANIL	HL60	0.99897676	1
PIPERINE	HL60	0.99900619	1
BAMBUTEROL	HL60	0.999020744	1
CORTICOSTERONE	HL60	0.999063763	1
DEFEROXAMINE	PC3	0.999068613	1
DILAZEP	HL60	0.999077888	1
TETRACAINE	HL60	0.99911962	1
KETANSERIN	HL60	0.999160387	1
TRETINOIN	HL60	0.99918671	1
HEXETIDINE	HL60	0.999200189	1
THIAMINE	HL60	0.999239025	1
CEFADROXIL	HL60	0.99926438	1
CEFOPERAZONE	PC3	0.999276897	1
BENZOCAINE	HL60	0.999301608	1
TOLMETIN	HL60	0.999313802	1
TRANEXAMIC ACID	HL60	0.999325889	1
ZUCLOPENTHIXOL	MCF7	0.999345982	1
PRESTWICK-984	HL60	0.999361507	1

PENTOXYVERINE	MCF7	0.999362639	1
PARTHENOLIDE	PC3	0.999373166	1
PODOPHYLLOTOXIN	HL60	0.999418725	1
PRESTWICK-682	HL60	0.99944086	1
MONOBENZONE	MCF7	0.999442703	1
AMPICILLIN	HL60	0.999451767	1
METHANTHELINIUM BROMIDE	HL60	0.999473259	1
CLENBUTEROL	HL60	0.999483843	1
ARECOLINE	MCF7	0.999488165	1
HALOFANTRINE	HL60	0.999504691	1
FLUCLOXACILLIN	HL60	0.999514953	1
CANADINE	HL60	0.999525109	1
MEGESTROL	HL60	0.999554931	1
LOMUSTINE	PC3	0.999559641	1
DIPHEMANIL METILSULFATE	HL60	0.999564657	1
FUSIDIC ACID	HL60	0.999583786	1
GLAFENINE	MCF7	0.999586728	1
DIPROPHYLLINE	HL60	0.99959319	1
PIMETHIXENE	MCF7	0.999612955	1
ISOMETHEPTENE	HL60	0.999620757	1
TRIMIPRAMINE	HL60	0.999629731	1
PRIMAQUINE	HL60	0.999638598	1
TRIMETHOBENZAMIDE	HL60	0.999656009	1
ANDROSTERONE	PC3	0.999664554	1
ADIPIODONE	PC3	0.999697658	1
ATRACTYLOSIDE	HL60	0.999705666	1
THIAMPHENICOL	HL60	0.999729044	1
CLORGILINE	HL60	0.999736621	1
ASIATICOSIDE	HL60	0.999751455	1
KARAKOLINE	HL60	0.999765858	1
CARBARSONE	HL60	0.999772899	1
PRIMIDONE	HL60	0.999786658	1
SR-95639A	HL60	0.999812887	1
MONOCROTALINE	HL60	0.999819175	1
VIGABATRIN	HL60	0.999825356	1
RILMENIDINE	PC3	0.999843255	1
AH-6809	PC3	0.999844734	1
DIPHENHYDRAMINE	HL60	0.999849006	1
GUANABENZ	PC3	0.999852798	1
ALPHA-ERGOCRYPTINE	HL60	0.999860186	1
ALFUZOSIN	HL60	0.999870936	1
HYCANTHONE	PC3	0.99987615	1
ISOFLUPREDONE	HL60	0.999881256	1
PROPIDIUM IODIDE	HL60	0.999886255	1
LEVOPROPOXYPHENE	HL60	0.999891146	1
GUANABENZ	HL60	0.99989593	1
NITROFURAL	HL60	0.999900607	1
CEFMETAZOLE	HL60	0.999905176	1
IODIXANOL	HL60	0.999913991	1
RILMENIDINE	HL60	0.999918238	1
CICLOPIROX	MCF7	0.99992731	1
AMITRIPTYLINE	HL60	0.999930332	1
LUMICOLCHICINE	HL60	0.999934149	1
NOCODAZOLE	MCF7	0.999935472	1
RAMIFENAZONE	HL60	0.999937858	1
CYCLOBENZAPRINE	HL60	0.999941459	1
PROCARBAZINE	HL60	0.999944954	1
ALEXIDINE	HL60	0.99994834	1
ISOCORYDINE	PC3	0.999951619	1
NADIDE	HL60	0.999954791	1
NOSCAPINE	HL60	0.999960811	1

ADIPHENINE	HL60	0.999966402	1
BIPERIDEN	HL60	0.999969036	1
CHLOROGENIC ACID	HL60	0.999971563	1
BOLDINE	HL60	0.999973982	1
HOMATROPINE	HL60	0.999976293	1
MERBROMIN	HL60	0.999980594	1
FUROSEMIDE	HL60	0.999982583	1
IOCETAMIC ACID	HL60	0.999984464	1
PHENETICILLIN	HL60	0.999986238	1
VIOMYCIN	HL60	0.999987905	1
CHICAGO SKY BLUE 6B	HL60	0.999989464	1
3-HYDROXY-DL-KYNURENINE	HL60	0.999990915	1
TRIBENOSIDE	HL60	0.999992259	1
THIAMAZOLE	HL60	0.999993495	1
OZAGREL	HL60	0.999994624	1
DOSULEPIN	HL60	0.999995646	1
CYCLOPENTHIAZIDE	HL60	0.99999656	1
METFORMIN	HL60	0.999997366	1
LACTOBIONIC ACID	HL60	0.999998065	1
CEFOTIAM	HL60	0.999998656	1
CARBENOXOLONE	HL60	0.99999914	1
DAPSONE	HL60	0.999999516	1
STREPTOZOCIN	HL60	0.999999785	1
ENOXACIN	HL60	1	1
