# Supplementary material for: The Diagnostic and Prognostic Values of HOXA Gene Family in Kidney Clear Cell Renal Cell Carcinoma
Source: J Oncol. 2022 Mar 16;2022:1762637. doi: 10.1155/2022/1762637 (PMC8942704; doi:10.1155/2022/1762637)
Supplement: Supplementary Materials — Supplementary Figure 1. Flow chart of data collection and subsequent model establishment in our study; Supplementary Figure 2. The TNM stage and clinical grade measured by the time-dependent receiver-operating characteristic curves at 1, 3, and 5 years. Supplementary Figure 3. The Kaplan–Meier survival curves of evaluating single prognostic roles of the prognosis-related HOXA genes. Supplementary Figure 4. A more detailed machine learning model construction process for early screening classification. Supplementary Figure 5. Custom scripts for prognostic model and machine learning model construction in this study; Table S1: differentially expressed genes between KIRC samples and paired normal samples, including the differential expression HOXA genes; Table S2: the training set, the test set, and the external validation set for machine learning model; Table S3: small molecule drugs by querying the CMAP and DGIdb databases; and Table S4: a detailed summary of the different roles of HOXA family members in KIRC. [file 1762637.f1.zip › 1762637.f1/Supplementary Table S4 (2).pdf]

Gene_name	Ensemble	Location	Alteration	Role	Ref.
HOXA1	ENSG00000105991	Chr 7:27092993-27096000	Unknown	-	-
HOXA2	ENSG00000105996	Chr 7:27100354-27102686	Downregulated	-	-
HOXA3	ENSG00000105997	Chr 7:27106184-27152583	Upregulated	Increase cell invasion and metastasis through partially the FAK/YAP signaling pathway	He C, Chen ZY, Li Y, et al. miR-10b suppresses cell invasion and metastasis through targeting HOXA3 regulated by FAK/YAP signaling pathway in clear-cell renal cell carcinoma. BMC Nephrol. 2019 Apr 11;20(1):127.
HOXA4	ENSG00000197576	Chr 7:27128507-27130780	Upregulated	-	-
HOXA5	ENSG00000106004	Chr 7:27141052-27143681	Downregulated	Show high methylation level and may initiate cancer and promote progression	Yoo KH, Park YK, Kim HS, et al. Epigenetic inactivation of HOXA5 and MSH2 gene in clear cell renal cell carcinoma. Pathol Int. 2010 Oct;60(10):661-6.
HOXA6	ENSG00000106006	Chr 7:27145396-27150603	Downregulated	Inhibit cell proliferation and induce apoptosis by suppressing the PI3K/Akt signaling pathway	Wu F, Wu S, Tong H, et al. HOXA6 inhibits cell proliferation and induces apoptosis by suppressing the PI3K/Akt signaling pathway in clear cell renal cell carcinoma. Int J Oncol. 2019 Jun;54(6):2095-2105.
HOXA7	ENSG00000122592	Chr 7:27153716-27157936	Downregulated	-	-
HOXA9	ENSG00000078399	Chr 7:27162438-27175180	Downregulated	Show high promoter methylation level and its expression level is related to tumor aggressiveness	Pires-Luís AS, Costa-Pinheiro P, Ferreira MJ, et al. Identification of clear cell renal cell carcinoma and oncocytoma using a three-gene promoter methylation panel. J Transl Med. 2017 Jun 29;15(1):149.
HOXA10	ENSG00000253293	Chr 7:27170592-27180261	Unknown	-	-
HOXA11	ENSG00000005073	Chr 7:27181157-27185232	Downregulated	Function as a tumor suppressor, significantly inhibit cell proliferation, colony formation, cell migration and invasion and induce KIRC cells apoptosis.	Wang L, Cui Y, Sheng J, et al. Epigenetic inactivation of HOXA11, a novel functional tumor suppressor for renal cell carcinoma, is associated with RCC TNM classification. Oncotarget. 2017 Mar 28;8(13):21861-21870.
HOXA13	ENSG00000106031	Chr 7:27193503-27200091	Upregulated	Function as a novel oncogene, promote cell proliferation, increase the protein level of p53 and decrease the protein level of cyclin D1 in KIRC cells.	Cui Y, Yan M, Zhang C, et al. Comprehensive analysis of the HOXA gene family identifies HOXA13 as a novel oncogenic gene in kidney renal clear cell carcinoma. J Cancer Res Clin Oncol. 2020
