# Supplementary figures and images for: The Diagnostic and Prognostic Values of HOXA Gene Family in Kidney Clear Cell Renal Cell Carcinoma
Source: J Oncol. 2022 Mar 16;2022:1762637. doi: 10.1155/2022/1762637 (PMC8942704; doi:10.1155/2022/1762637)

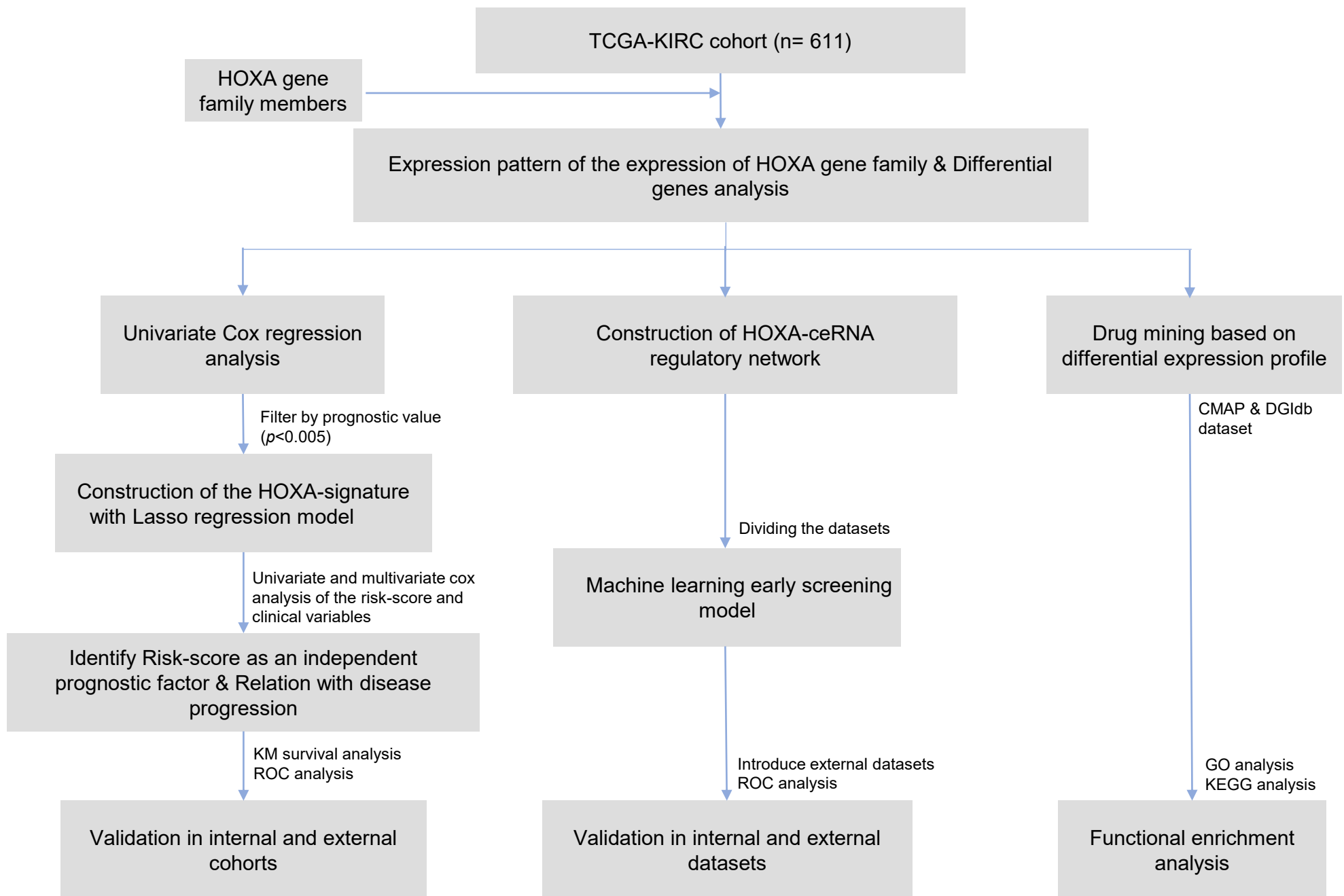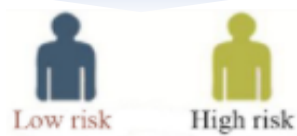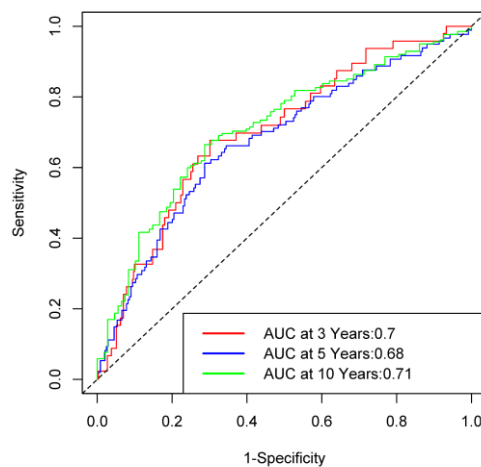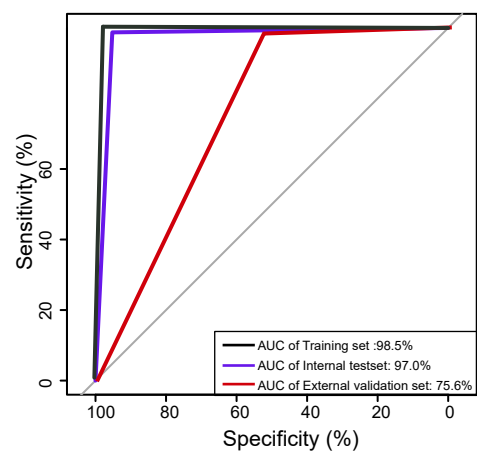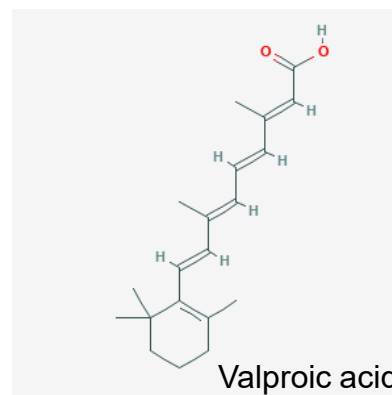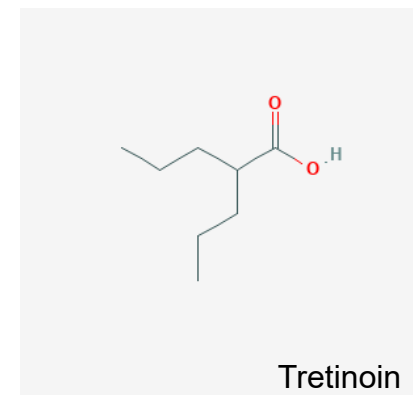

Supplement: Supplementary Materials — Supplementary Figure 1. Flow chart of data collection and subsequent model establishment in our study; Supplementary Figure 2. The TNM stage and clinical grade measured by the time-dependent receiver-operating characteristic curves at 1, 3, and 5 years. Supplementary Figure 3. The Kaplan–Meier survival curves of evaluating single prognostic roles of the prognosis-related HOXA genes. Supplementary Figure 4. A more detailed machine learning model construction process for early screening classification. Supplementary Figure 5. Custom scripts for prognostic model and machine learning model construction in this study; Table S1: differentially expressed genes between KIRC samples and paired normal samples, including the differential expression HOXA genes; Table S2: the training set, the test set, and the external validation set for machine learning model; Table S3: small molecule drugs by querying the CMAP and DGIdb databases; and Table S4: a detailed summary of the different roles of HOXA family members in KIRC. [file 1762637.f1.zip › 1762637.f1/Supplementary Figure 1 (2).pdf]

Grade

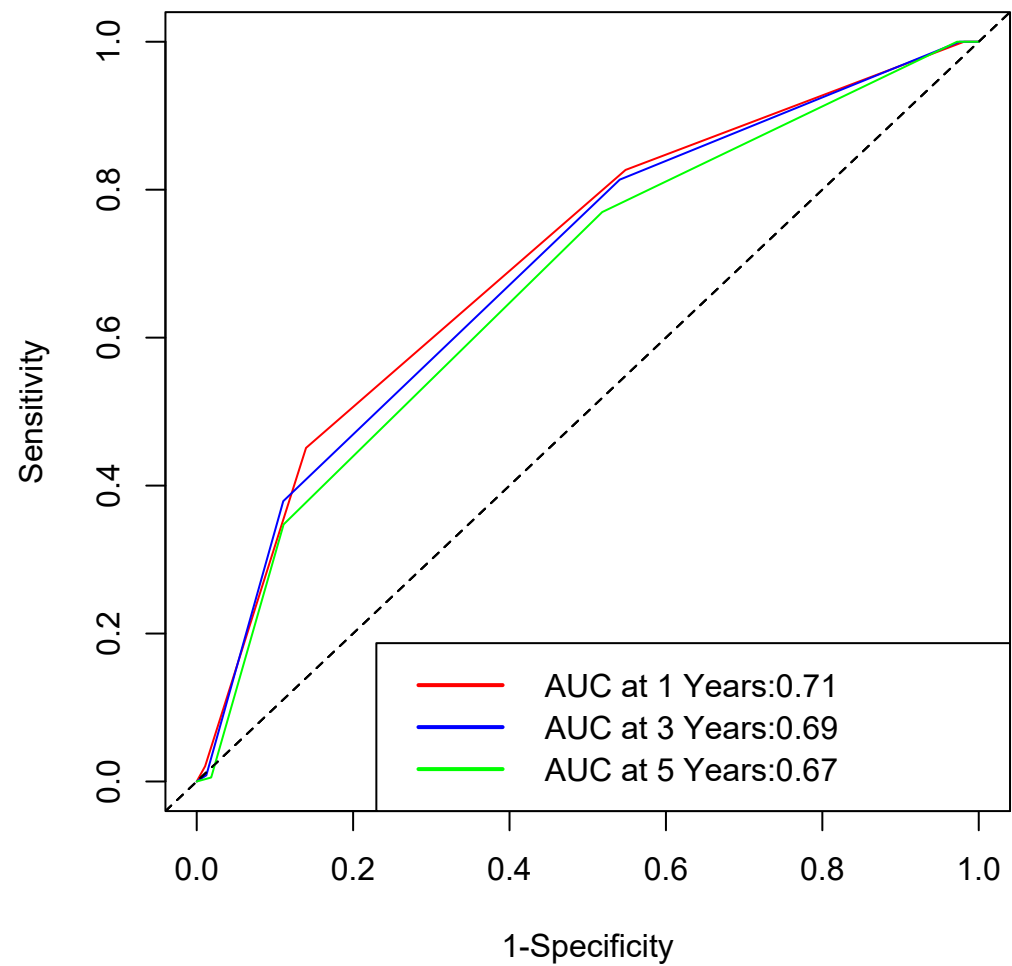

TNM

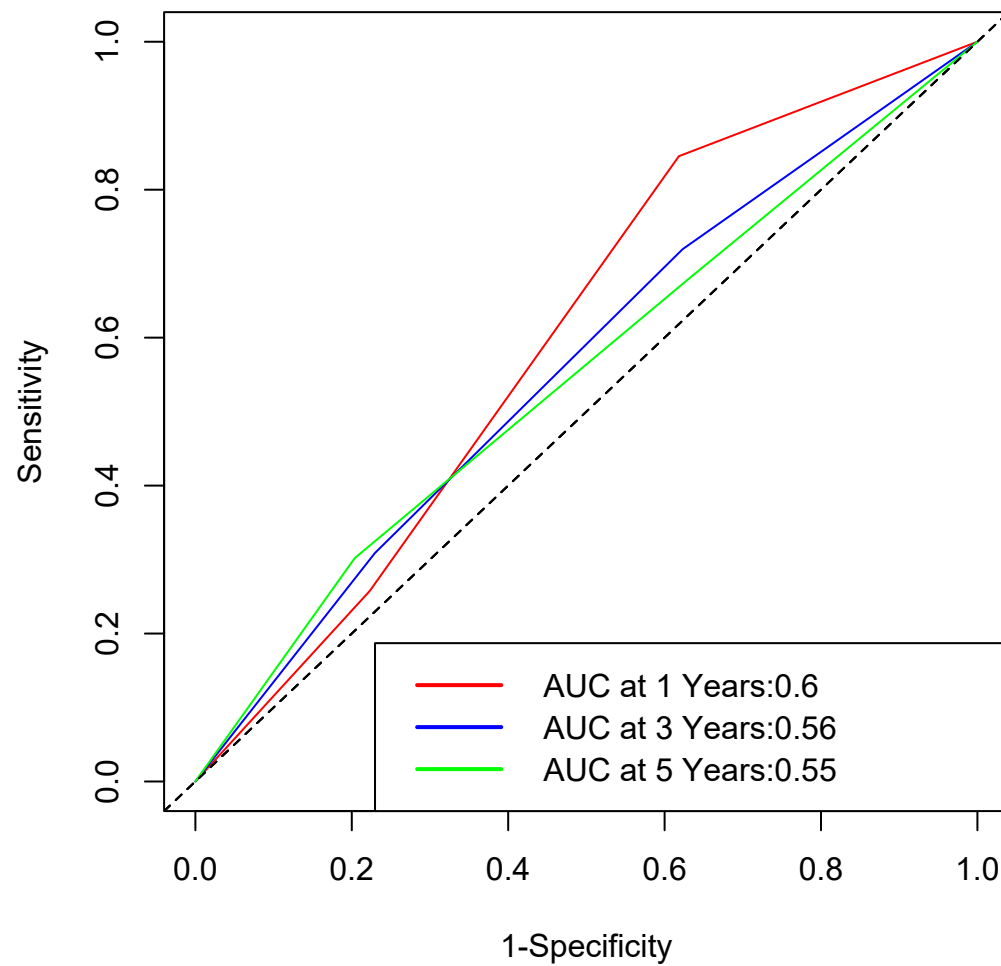

Supplement: Supplementary Materials — Supplementary Figure 1. Flow chart of data collection and subsequent model establishment in our study; Supplementary Figure 2. The TNM stage and clinical grade measured by the time-dependent receiver-operating characteristic curves at 1, 3, and 5 years. Supplementary Figure 3. The Kaplan–Meier survival curves of evaluating single prognostic roles of the prognosis-related HOXA genes. Supplementary Figure 4. A more detailed machine learning model construction process for early screening classification. Supplementary Figure 5. Custom scripts for prognostic model and machine learning model construction in this study; Table S1: differentially expressed genes between KIRC samples and paired normal samples, including the differential expression HOXA genes; Table S2: the training set, the test set, and the external validation set for machine learning model; Table S3: small molecule drugs by querying the CMAP and DGIdb databases; and Table S4: a detailed summary of the different roles of HOXA family members in KIRC. [file 1762637.f1.zip › 1762637.f1/Supplementary Figure 2 (1).pdf]

Overall Survival

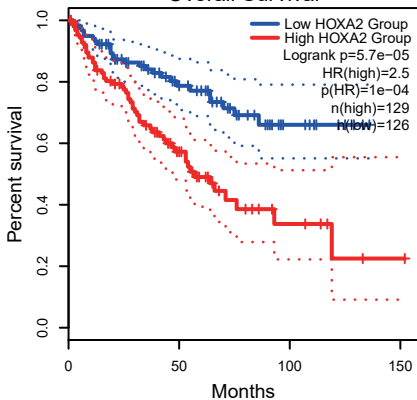

Overall Survival

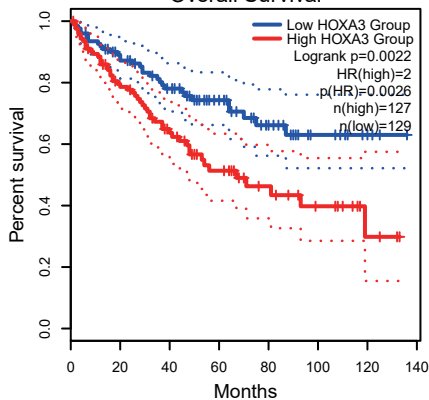

Overall Survival

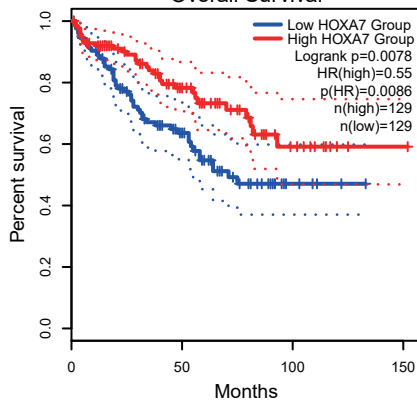

Overall Survival

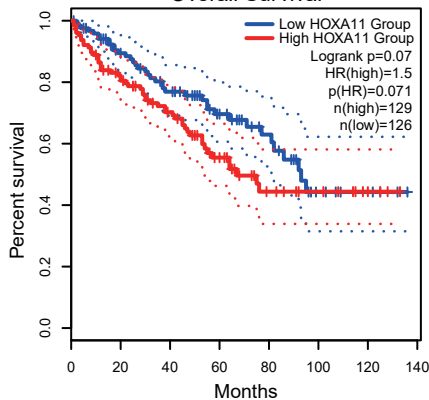

Overall Survival

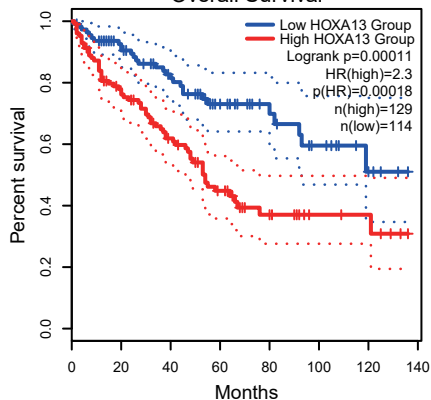

Supplement: Supplementary Materials — Supplementary Figure 1. Flow chart of data collection and subsequent model establishment in our study; Supplementary Figure 2. The TNM stage and clinical grade measured by the time-dependent receiver-operating characteristic curves at 1, 3, and 5 years. Supplementary Figure 3. The Kaplan–Meier survival curves of evaluating single prognostic roles of the prognosis-related HOXA genes. Supplementary Figure 4. A more detailed machine learning model construction process for early screening classification. Supplementary Figure 5. Custom scripts for prognostic model and machine learning model construction in this study; Table S1: differentially expressed genes between KIRC samples and paired normal samples, including the differential expression HOXA genes; Table S2: the training set, the test set, and the external validation set for machine learning model; Table S3: small molecule drugs by querying the CMAP and DGIdb databases; and Table S4: a detailed summary of the different roles of HOXA family members in KIRC. [file 1762637.f1.zip › 1762637.f1/Supplementary Figure 3 (1).pdf]
